# iMiRNA-SSF: Improving the Identification of MicroRNA Precursors by Combining Negative Sets with Different Distributions

**DOI:** 10.1038/srep19062

**Published:** 2016-01-12

**Authors:** Junjie Chen, Xiaolong Wang, Bin Liu

**Affiliations:** 1 School of Computer Science and Technology, Harbin Institute of Technology Shenzhen Graduate School, Shenzhen, Guangdong, China; 2 Key Laboratory of Network Oriented Intelligent Computation, Harbin Institute of Technology Shenzhen Graduate School, Shenzhen, Guangdong, China

## Abstract

The identification of microRNA precursors (pre-miRNAs) helps in understanding regulator in biological processes. The performance of computational predictors depends on their training sets, in which the negative sets play an important role. In this regard, we investigated the influence of benchmark datasets on the predictive performance of computational predictors in the field of miRNA identification, and found that the negative samples have significant impact on the predictive results of various methods. We constructed a new benchmark set with different data distributions of negative samples. Trained with this high quality benchmark dataset, a new computational predictor called iMiRNA-SSF was proposed, which employed various features extracted from RNA sequences. Experimental results showed that iMiRNA-SSF outperforms three state-of-the-art computational methods. For practical applications, a web-server of iMiRNA-SSF was established at the website http://bioinformatics.hitsz.edu.cn/iMiRNA-SSF/.

MicroRNAs (miRNAs) are a class of evolutionally conserved, single-stranded, small (approximately 19–23 nucleotides), endogenously expressed and non-protein-coding RNAs that act as post-transcriptional regulators of gene expression in a broad range of animals, plants and viruses^1^,^2^,^3^,^4^. MiRNAs play an important role as a regulator in biological process^5^. The aberrant expressions have been observed in many cancers^6^,^7^,^8^,^9^ and several miRNAs have been convincingly proved to play important roles in carcinogenesis^10^. The protein architecture in different programmed cell death (PCD) subroutines has been explored, but the global network organization of the noncoding RNA (ncRNA)-mediated cell death system is limited and ambiguous^11^,^12^. Thus, the discovery of human miRNAs regulation is an important task.

As traditional experimental methods for miRNA identification are time and money consuming, recently more attention has been paid to the development of computational approaches. Because miRNAs are short, the traditional feature engineering approaches^13^,^14^,^15^ are usually failed to extract features based on their sequences and structures, and therefore, computational approaches usually identify the precursors of miRNAs (pre-miRNAs) instead of miRNA. A variety of software tools for this purpose have been proposed. As shown in previous studies, extracting useful features are important for constructing a computational predictor^16^. Various features and machine learning techniques have been proposed to predict miRNAs. Triplet-SVM^17^ incorporated a local contiguous sequence-structure composition feature and utilized SVM to construct the predictor. MiPred^18^ identified the human pre-miRNAs by using an RF classifier with a combined feature set, including local contiguous sequence-structure (Triplet-SS), minimum of free energy feature (MFE) and *P*-value of randomization test feature (*P*-value). Compared with Triplet-SVM, MiPred improved the performance by nearly 10% in terms of accuracy. MiRanalyzer^19^ employed an RF classifier trained with a variety of features associated with nucleotide sequence, structure and energy. Wei, L. *et al.*^20^ proposed a SVM-based method called miRNApre using the local contiguous structure-sequence composition feature, primary sequence composition feature, and MFE. Recently some predictors have been proposed based on the predicted secondary structure of RNA sequences, such as iMiRNA-PseDPC^21^, iMcRNA-PseSSC^22^, miRNA-dis^23^, deKmer^24^, etc. These methods using different features and classifiers treat the pre-miRNA identification problem as a binary classification problem. Currently, the widely used classification algorithms include Support Vector Machine (SVM)^17^,^25^, Hidden Markov Model (HMM)^26^, Random Forest (RF)^18^, and Naive Bayes (NB)^27^. The widely used features of characterizing pre-miRNAs include stem-loop hairpin structures^28^,^29^, MFE of the pre-miRNAs, and *P*-value of randomization test^18^,^19^,^20^,^30^. Because the importance of the features for constructing a predictor, recently, some web-servers or stand-alone tools were proposed to extract the features from RNA sequences, such as Pse-in-One^31^, and repRNA^32^. MiPred^18^ identified the human pre-miRNAs by combining Triplet-SS, MFE and *P*-value, in which MFE and *P*-value were the top 2 most important features. MiRanalyzer^19^ was trained with a variety of features, in which MFE was the secondary most important feature. miRNApre was built based on Triplet-SS, primary sequence composition feature, and MFE. However, based on the feature analysis of miRNApre, MFE feature cannot improve the performance. Therefore, it is interesting to explore the reasons for the different discriminative power of the same feature in different predictors. Furthermore, there are several other challenging problems should be solved in this filed:

1. Many features have been proposed to characterize the pre-miRNAs, but their discriminative power is not investigated. Some features showed strong discriminative power in some predictors, while in other predictors, they only showed limited discriminative power, for example MFE played an important role in Triplet-SVM, but it almost had no contribution to the discriminative power of miRNApre. Therefore, the most discriminative features and their combinations for miRNA identification should be investigated.
2. The existing benchmark datasets are too small to reflect the statistical profile. Most of these datasets only contain several hundreds of real pre-miRNA samples and pseudo pre-miRNA samples. It is necessary to construct an updated benchmark dataset to fairly evaluate the performance of different methods.
3. Most of these methods performed well in cross validation test, but they showed much lower performance on independent testing sets. This is because the samples in the training set are not representative enough, especially for the pseudo pre-miRNA samples (negative samples). There is no golden standard to select or construct the negative samples^33^,^34^.

To solve these problems, we investigated the distributions of various benchmark datasets, and found that they had large variance, especially for the distributions of negative samples. A series of controlled experiments were conducted to find out how the performance were impacted on different distributions of negative samples. The results showed the negative samples were not representative enough. Therefore, the key to improve predictive performance was to construct an high quality benchmark dataset for miRNA identification. In this regard, a new benchmark dataset was constructed, in which the positive samples were extracted from the miRBase^35^,^36^,^37^, and the negative samples were selected from existing datasets with different data distributions. Finally, we proposed a new computational method for pre-miRNA identification, called iMiRNA-SSF, which employed the sequence and structure features trained with the updated benchmark dataset. The web-server of iMiRNA-SSF can be accessed at http://bioinformatics.hitsz.edu.cn/iMiRNA-SSF/.

## Results

### Negative samples have significant impact on the discriminative power of features

As reported in literatures, MFE and *P*-value were the top 2 most important features in MiPred^18^, but they were not so important in miRNApre^20^ (out of top 10 features). The main difference between these two methods was their negative samples in benchmark datasets. The negative samples of MiPred were collected from the protein coding regions with parameter filtering method, while the negative samples of miRNApre were collected by multi-level process. For more details, please refer to^17^,^20^.

Our hypothesis was that the different discriminative power of the same method was caused by the negative samples. In order to validate this hypothesis, two datasets 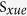 and 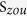 were constructed with the same positive set and the different negative sets:


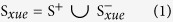



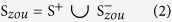


where the 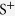, 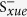 and 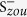 are the same as the subsets in Equation (4). The dataset 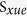 is union of 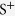 and 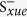; 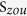 is union of 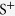 and 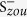.

We investigated the discriminative power of all features mentioned in Method section on datasets 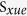 and 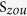 by assessing their information gain related to the classes. The higher information gain value^38^ means the related feature is more powerful. The top 20 most important features on the two datasets were shown in Table 1(A,B), respectively.

MFE and *P*-value were the top 2 most important features on 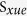. However, *P*-value was only ranked at 20^*th*^ on 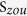 and MFE was ranked out of top 20. We also found that 14 of the top 20 most important features on 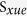 belonged to local triplet sequence-structure features (Triplet-SS) category, but only 4 features belonged to primary sequence features (3-gram) category. In contrast, for 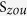, only 5 of the top 20 most important features belonged to Triplet-SS category, and 14 features belonged to 3-gram category. The structure features are more powerful than sequence features on 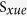 database, but it is not the case on 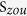 database. The results showed that the negative samples have significant impact on the discriminative power of features.

**Figure 1:**
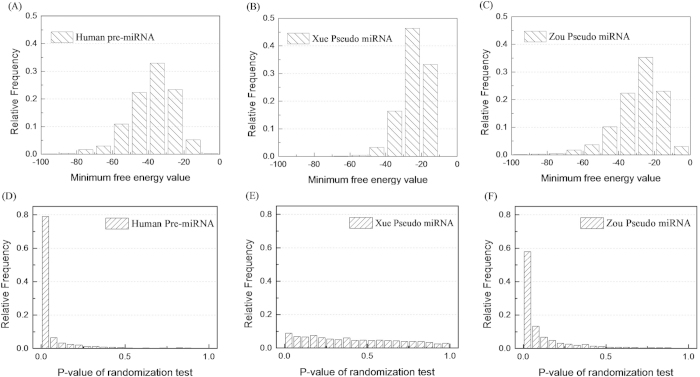
The distributions of MFE and *P*-value in the positive set and two negative sets. (**A**), (**B**) and (**C**) are the comparison of distributions of MFE on 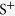, 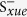 and 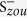, respectively. (**D**, **E**) and (**F**) are the comparison of distributions of *P*-value on 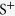, 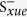 and 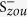, respectively.

We took MFE and *P*-value as examples to analyse the reasons. Their distributions on positive and negative samples of 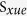 and 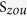 were calculated, and the results were shown in Fig. 1. The distributions of MFE and *P*-value are very similar between 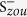 and 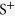, but they are different between 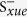 and 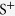. A feature has more discriminative power if its distribution has variance on positive and negative sets. This is why MFE and *P*-value show powerful discriminability on 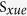 database, but it is not the case on 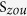 database.

### Importance of negative samples for training a classifier

The different distributions of negative samples have significant impact on the performance of a trained classifier. However, how does it come into being and how to avoid this problem?

We conducted the controlled experiments, employing all features (Triplet-SS, MFE, *P*-value and *N*-gram). The training sets and testing sets were constructed:


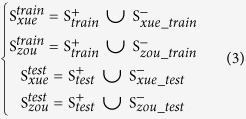


where 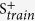 and 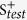 are disjoint subsets of 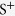, in which respectively contain 1312 and 300 human pre-miRNAs; 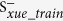 and 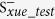 are disjoint subsets of 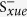, in which respectively contain 1312 and 300 Xue pseudo pre-miRNAs; 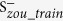 and 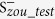 are disjoint subsets of 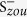, in which respectively contain 1142 and 300 Zou pseudo pre-miRNAs. The numbers of samples in each dataset were carefully chosen to avoid bias.

The prediction results were listed in Table 2. The cross validation results were achieved by leave-one-out strategy on 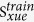 and 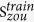, whereas the independent testing results were achieved by testing on 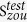 and 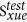. In term of Table 2, both two predictors performed well in cross validation test, achieved 87.69% and 98.57% accuracies, respectively. But they showed much lower performance on the independent testing dataset, especially the performance of the classifier trained on 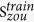 and tested on 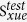 dropped to 51.17% from 98.57% in term of accuracy.

**Figure 2:**
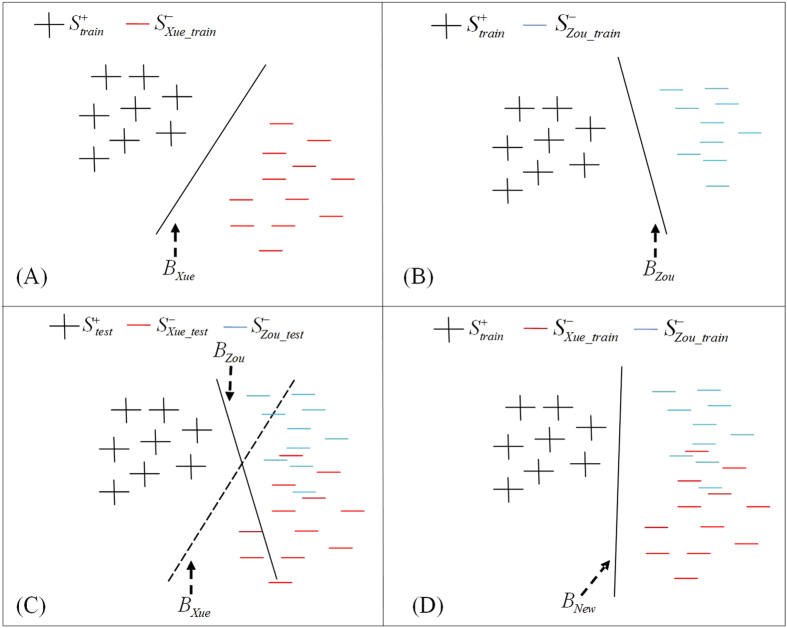
Importance of negative sample distribution for a SVM classifier decision boundary. *B*_*xue*_ is the generated decision boundary based on 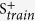 and 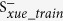; *B*_*zou*_ is the generated decision boundary based on 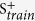 and 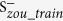; *B*_*New*_is the generated decision boundary based on 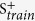, 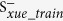 and 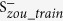.

For a SVM-based method, it generates a decision boundary that separates the positive samples from the negative ones. The generated decision boundaries based on different datasets are significant difference. As shown in Fig. 2(A,B), the two generated decision boundaries built on two datasets with different distributions are different. When using a decision boundary to classify samples in another dataset, the majority of samples can’t fall on their own categories. As shown in Fig. 2(C), if samples in 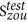 as test samples, the decision boundary *B*_*xue*_ performs badly to classify them into two classes. If samples in 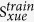 as test sample, the same is to *B*_*zou*_. But if we merge 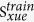 and 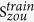 into one dataset, the generated new decision boundary *B*_*New*_ based on the new dataset can improve the predictive performance significantly. As shown in Fig. 2(D), the new decision boundary *B*_*New*_ can separate all samples correctly. It indicates that new decision boundary *B*_*New*_ is more general and outperforms *B*_*xue*_ and *B*_*zou*_.

### A new predictor built on updated benchmark dataset

We constructed a new benchmark set with different data distributions of negative samples, including real human pre-miRNAs as positive set, Xue pseudo pre-miRNAs 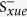 and Zou pseudo pre-miRNAs 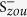 as negative sets. Trained with this high quality benchmark dataset, a new computational predictor called iMiRNA-SSF was proposed. Four kinds of features were employed to investigate that if they could be combined to improve performance of iMiRNA-SSF, including Triplet-SS, MFE, *P*-value and *N*-gram. The performance was obtained by using LibSVM algorithm with leave-one-out crossing validation on updated benchmark dataset. As shown in Table 3, the best performance (ACC = 90.42%, MCC = 0.79) was achieved with the combination of the four kinds of features. Triplet-SS is a local triplet sequence-structure-based feature; MFE and *P*-value are features based on the on minimum of free energy of the secondary structure; *N*-gram is a sequence-based feature considering the local sequence composition information. These features describe the characteristics of pre-miRNA from different aspects. Therefore the predictive performance of iMiRNA-SS can be further enhanced by combining all of features.

Furthermore, the importance of all features was also investigated. *P*-value and MFE features are the most discriminative, followed by the local triplet sequence-structure features and the primary sequence based features. The results were shown in Table 4.

### Comparison with other methods

Three state-of-the-art methods Triplet-SVM^17^, MiPred^18^ and miRNApre^20^ were selected to compare with the proposed iMiRNA-SSF. MiPred is a classifier using Random Forest algorithm combined with Triplet-SS, MFE, and *P*-value features. miRNApre employed the SVM algorithm with Triplet-SS, *N*-gram, MFE features. As mentioned in the introduction section, the reported accuracy of these methods were based on small datasets containing only several hundreds of samples without removing redundant sequences, thus, their performance might be overestimated. In order to make a fair comparison among these methods, all these methods were evaluated on the same updated benchmark dataset via leave-one-out crossing validation. Their predictive results were shown in Table 5.

**Figure 3:**
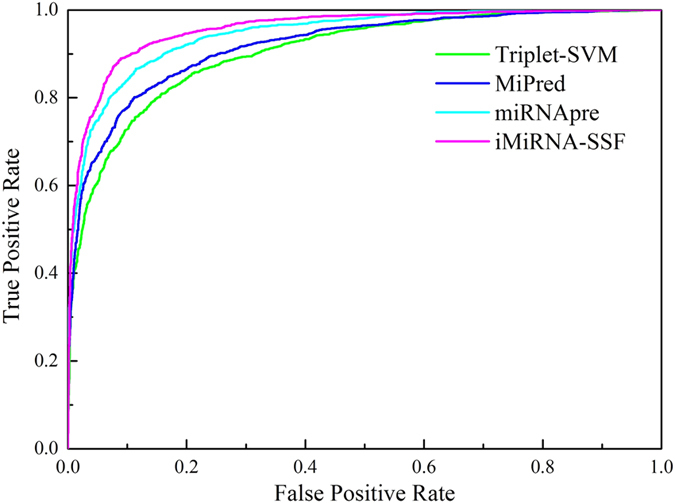
A graphical illustration to show the performance of different methods by the receiver operating characteristic (ROC) curves.

To further illustrate the comparison, receiver operating characteristic (ROC) scores of different methods were provided in Fig. 3. The ROC scores of Triplet-SVM, MiPred, miRNApre and iMiRNA-SSF are 0.90, 0.92, 0.94 and 0.96, respectively. iMiRNA-SSF outperforms the other three state-of-the-art methods.

### Web-server description

For the convenience of the vast majority of experimental scientists, we provided a simple guide on how to use the iMiRNA-SSF web-server. It is available at http://bioinformatics.hitsz.edu.cn/iMiRNA-SSF/.

**Figure 4:**
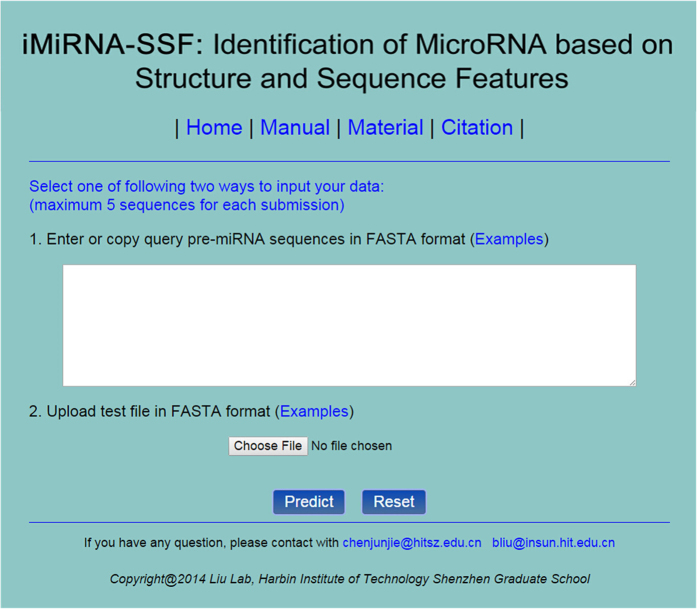
The homepage of iMiRNA-SSF webserver. The users can input their test data through two ways. One way is to copy their query to the text area, and the other is to upload their test file in FASTA format.

**Step 1:** The homepage was shown in Fig. 4. The users can input their test data through two ways. One way is to copy pre-miRNA sequences in FASTA format into text area. The other way is to upload test file. Example sequences can be found by clicking on the Example link.

**Step 2:** Click on the prediction button to submit. iMiRNA-SSF will decide whether the test sequences are real human pre-miRNA sequences or not. Note that the computational cost of *P*-value feature is expensive, because for each query sequence we need to predict the secondary structures of its random shuffled sequences for 1000 times via running Vienna RNA software.

**Figure 5:**
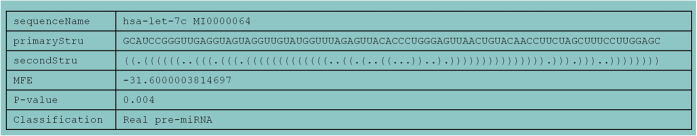
An example of prediction result. If the classification is predicted to Real pre-miRNA, it indicates the query most probably is a pre-miRNA. Some useful information is also provided, including second structure, MFE and P-value.

**Step 3:** An output example was shown in Fig. 5. If the classification is predicted to Real pre-miRNA, it indicates the query most probably is a pre-miRNA. Besides the predictive classification, we output other useful information, including the secondary structure, MFE and *P*-value.

## Discussion

By exploring two datasets that were constructed with the same positive set and different negative sets, we found that negative samples have significant impact on the predictive results of various methods. Therefore, we constructed an updated benchmark set with different data distributions of negative samples. A new predictor called iMiRNA-SSF was proposed, which was trained with this high quality benchmark dataset. Experimental results showed that iMiRNA-SSF achieved an accuracy of 90.42%, an MCC of 0.79 and an ROC score of 0.96, outperforming three state-of-the-art computational methods, including Triplet-SVM, MiPred, and miRNApre. Furthermore, the discriminative power of employed features was investigated on an updated benchmark. The results showed that structure features are more discriminative than the sequence features for pre-miRNA identification.

As shown in this study, the quality of the training samples is very important for improving the predictive performance of a computational predictor. The proposed framework of combining samples with different distributions can be applied to other important tasks in the field of bioinformatics, such as DNA binding protein identification^39^,^40^, protein remote homology detection^41^,^42^, enhancers and their strength prediction^43^, etc. Therefore, in our future studies, we will focus on applying the proposed framework to improve the performance of these problems.

## Method

### Datasets

Our benchmark dataset for pre-miRNA identification (see the Supplementary information) consists of real human pre-miRNAs as positive set and two pseudo pre-miRNAs subsets as negative set. The pre-miRNAs sharing sequence similarity more than 80% were removed using the CD-HIT software^44^ to get rid of redundancy and avoid bias. The benchmark dataset can be formulated as:


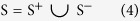



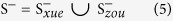


where the positive samples set 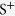 contains 1612 human miRNA precursors, which were selected from the 1872 reported Homo sapiens pre-miRNA entries downloaded from the miRBase^36^,^37^; the negative samples set 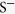
is the union of 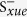 and 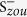; the 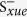 contains 1612 Xue pseudo miRNAs, which were selected from the 8494 pre-miRNA-like hairpins^17^; the 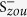 contains 1442 Zou pseudo miRNAs^20^. As miRNAs locate in the untranslated regions or intragenic regions, both 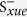 and 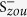 were collected from the protein coding regions. The main difference between them is that they were constructed based on different techniques. The 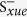 was collected by the widely accepted characteristics and the 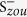 was collected by a multi-level negative sample selection technique. For more information, please refer to^17^,^20^.

### Features for characterizing microRNA precursors

Various sequence-based features were used in this study, including primary sequence features, minimum free energy feature, *P*-value randomization test feature and local triplet sequence-structure features, which were described as followings:

#### Primary sequence features (N-gram)

For a given RNA sequence **R**:


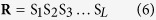


where S_*i*_
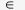
{Adenine (A), Cytosine (C), Guanine (G), Uracil (U)}; S_1_ denotes the nucleic acid residue at sequence position 1, S_2_ denotes the nucleic acid residue at position 2, and so on. The sequence pattern S_*i* + 1_S_*i* + 2_S_*i* + 3_…S_*i* + *N*_ is called *N*-gram. *N*-grams refer to all the possible sub-sequences. The different kinds of *N*-grams are 4^*n*^ (*n* is the length of the *N*-gram). Following previous studies^17^, we set *n* as 3 and the number of different 3-grams is 64 (4^3^).

#### Minimum of free energy feature (MFE)

The MFE describes the stability of a RNA secondary structure. Some evidences showed that miRNAs have lower folding free energies than random sequences^45^. The MFE of the secondary structure was predicted by the Vienna RNA software package (released 2.1.6)^46^ with default parameters.

#### P-value of randomization test feature (P-value)

In order to determine if the MFE value is significantly different from that of random sequences, a Monte Carlo randomization test was used^47^. The process can be summarized as follow:

1. Infer MFE value of the original sequence.
2. Randomize the order of the nucleotides of the original sequence while keeping the dinucleotide distribution (or frequencies) constant^48^. Then infer the MFE value of the shuffled sequence.
3. Repeat step 2 for 999 times to build the distribution of random sequence MFE values.
4. Denote *Num* as the number of shuffled sequences that their MFE value is not greater than the original sequence MFE value, then *P*-value can be computed based on:

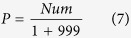


#### Local triplet sequence-structure features (Triplet-SS)

In the predicted secondary structure, there are only two statuses for each nucleotide, paired or unpaired, represented as brackets “(” or “)” and dots “.”, respectively. The left bracket “(” means that the paired nucleotide is located near the 5′-end and the right bracket “)” means one nucleotide can be paired with another at the 3′-end. When the sequences were represented as vectors, we didn’t distinguish these two situations and used “(” for both situations. For any 3 adjacent nucleotides, there are 8 (2^3^) possible structure compositions: “(((”, “((.”, “(..”, “(.(”, “.((”, “.(.”, “..(” and “…”. Considering the middle nucleotide among the three adjacent nucleotides, there are 32 (4 × 8) possible sequence-structure combinations, which they can be denoted as “U(((”, “A((.”, etc.. The occurrence frequencies of all 32 possible triplet elements were counted along the stem portions of a hairpin segment. Details of the 32 sequence-structure features can be found in^17^.

### Support Vector Machine

Support Vector Machine (SVM) is a supervised machine learning technique based on statistical theory for classification task^49^. Given a set of fixed length vectors with positive or negative labels, SVM can learn an optimal hyper plane to discriminate the two classes. New test samples can be classified based on the learned classification rule. SVM has exhibited excellent performance in practice and has a strong theoretical foundation of statistical learning.

In this study, the LibSVM algorithm was employed, which is an integrated software tool for SVM classification and regression. The kernel function was set as Radial Basis Function (RBF). The two parameters 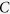 and 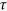 were set as 11 and −9 respectively, which were optimized by using the grid tool in LibSVM package^49^.

### Leave one out cross validation

Three test validation methods, including independent dataset test, sub-sampling (or K-fold cross-validation) test and leave-one-out test, are often used to evaluate the performance of a predictor. Among these three methods, the leave-one-out test is deemed the least arbitrary and most objective as elucidated in^49^,^50^,^51^. It has been widely recognized and adopted by investigators to examine the quality of various predictors. In the leave-one-out test, each sequence in the benchmark dataset is in turn singled out as an independent test sample and all the rule-parameters are calculated with the whole benchmark dataset.

### Measurement

For a prediction problem, a classifier can predict an individual instance into the following four categories: false positive (FP), true positive (TP), false negative (FN) and true negative (TN). As shown in previous studies^52^,^53^, the total prediction accuracy (ACC), Specificity (Sp), Sensitivity (Sn) and Mathew’s correlation coefficient (MCC) for assessment of the prediction system are given by:


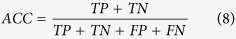



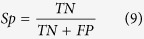



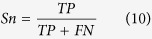



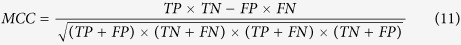


The receiver operating characteristic (ROC) score^54^ was also employed to evaluate the performance of different methods. Because it can evaluate the trade-off between specificity and sensitivity. An ROC score is the normalized area under a curve that is plotted with true positives as a function of false positives for varying classification thresholds. An ROC score of 1 indicates a perfect separation of positive samples from negative samples, whereas an ROC score of 0.5 denotes that random separation.

## Additional Information

**How to cite this article**: Chen, J. *et al.* iMiRNA-SSF: Improving the Identification of MicroRNA Precursors by Combining Negative Sets with Different Distributions. *Sci. Rep.* **6**, 19062; doi: 10.1038/srep19062 (2016).

## Supplementary Material

Supplementary S1

## Figures and Tables

**Table 1:** The importance of top 20 features.

(A)			(B)		
Features	*IG(c, x)*^a^	Rank	Features	*IG(c, x)*^a^	Rank
P-value	313.37	1	CGA	127.01	1
MFE	148.71	2	GCU	99.65	2
A(((	82.41	3	ACC	89.44	3
U(((	56.77	4	UGC	88.69	4
A…	47.77	5	GAC	72.01	5
C…	47.01	6	ACG	62.12	6
U…	39.56	7	CUG	60.67	7
C(((	29.03	8	UGG	60.03	8
G(((	26.95	9	A…	56.71	9
A..(	22.20	10	U(((	50.33	10
A(.(	20.68	11	CCG	50.26	11
G…	20.06	12	UCG	37.43	12
GGG	18.62	13	G(((	34.71	13
C(.(	17.66	14	GCA	34.62	14
U..(	15.29	15	CGU	34.27	15
CUA	14.15	16	GGC	30.87	16
G.((	13.37	17	C…	30.81	17
G(..	13.16	18	C..(	25.43	18
UAG	13.11	19	AGC	24.63	19
CCG	13.10	20	P-value	24.16	20

(A) and (B) are the ranking of top 20 most important features on 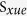 and 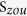, respectively.

^a^*IG(c, x)*: The information gain of features is a feature selection method used in many fields. In general terms, the expected information gain is the change in information entropy *H* from a prior state to a state that takes some information. The higher the information gain value means the feature is more discriminative.

**Table 2:** The comparison with cross validation and independent testing in controlled experiments with two datasets that have different distributions of negative samples.

Training dataset	Testing dataset	ACC		MCC	
		cross validation^a^	independent testing^b^	cross validation^a^	independent testing^b^
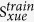	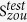	87.69%	77.83%	0.75	0.59
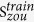	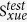	98.57%	51.17%	0.97	0.07

^a^The results were computed with leave-one-out cross validation strategy.

^b^The results were computed on independent test dataset.

**Table 3:** The performance of iMiRNA-SSF on an updated benchmark 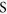 with different features combination.

Features	ACC	Sn	Sp	MCC
Triplet-SS	83.99%	79.09%	86.28%	0.64
Triplet-SS, MFE, P-value	86.05%	81.55%	88.23%	0.69
Triplet-SS, MFE,P-value, N-gram	**90.42%**	**85.89%**	**92.84%**	**0.79**

Note: The performance was assessed by leave-one-out crossing validation.

**Table 4:** The ranking of top 20 important features in the updated benchmark dataset.

Features	*IG(c, x)*^a^	Rank
P-value	166.6647	1
MFE	110.099	2
U(((	93.73114	3
A…	76.72718	4
A(((	62.0189	5
C…	55.73455	6
CGA	53.6649	7
G(((	42.86916	8
A..(	35.29512	9
CCG	30.7314	10
U…	28.18673	11
C(((	26.42756	12
A(..	25.07459	13
GGG	24.51414	14
UCG	24.0945	15
ACC	22.93585	16
CUG	21.95057	17
GAC	21.93206	18
UGC	21.79752	19
C(..	21.2629	20

^a^*IG(c, x)*: The information gain of features is a feature selection method used in many fields. In general terms, the expected information gain is the change in information entropy *H* from a prior state to a state that takes some information. The higher the information gain value means the feature is more discriminative.

**Table 5:** The performance comparison of different methods.

Method	Acc(%)	Sn(%)	Sp(%)	MCC	ROC
Triplet-SVM	83.99%	79.09%	86.28%	0.64	0.90
MiPred	86.05%	81.55%	88.23%	0.69	0.92
miRNApre	88.36%	84.82%	90.10%	0.74	0.94
iMiRNA-SSF	**90.42%**	**85.89%**	**92.84%**	**0.79**	**0.96**

All the methods were evaluated on the same updated benchmark dataset via leave-one-out crossing validation.

Note: Since the number of positive samples is not equal to the number of negative samples, we set the penalty factors that positive samples weight is 2 and the negative samples weight is 1.
