# Supplementary material for: iMiRNA-SSF: Improving the Identification of MicroRNA Precursors by Combining Negative Sets with Different Distributions
Source: Sci Rep. 2016 Jan 12;6:19062. doi: 10.1038/srep19062 (PMC4709562; doi:10.1038/srep19062)
Supplement: Supplementary S1 [file srep19062-s1.pdf]

Supplementary Information for

iMiRNA-SSF: Improving the Identification of MicroRNA Precursors by Combining Negative Sets with Different Distributions

Junjie Chen, Xiaolong Wang, and Bin Liu

Supplementary S1. The benchmark dataset S contains 4666 sequences, which are classified into subset S^+ with 1612 Human pre-microRNAs (positive samples) and subset S^- with 1612 Xue pseudo miRNAs and 1442 Zou pseudo miRNAs (negative samples).

(1). 1612 Human pre-microRNAs
>hsa-let-7c MI0000064
GCAUCCGGGUUGAGGUAGUAGGUUGUAUGGUUUAGAGUUACACCCUGGGAGUUAACUGUACAACC
UUCUAGCUUUCCUUGGAGC
>hsa-let-7d MI0000065
CCUAGGAAGAGGUAGUAGGUUGCAUAGUUUUAGGGCAGGGAUUUUGCCCACAAGGAGGUAACUAU
ACGACCUGCUGCCUUUCUUAGG
>hsa-let-7e MI0000066
CCCGGGCUGAGGUAGGAGGUUGUAUAGUUGAGGAGGACACCCAAGGAGAUCACUAUACGGCCUCC
UAGCUUUCCCCAGG
>hsa-let-7f-1 MI0000067
UCAGAGUGAGGUAGUAGAUUGUAUAGUUGUGGGGUAGUGAUUUUACCCUGUUCAGGAGAUAACUA
UACAAUCUAUUGCCUUCCCUGA
>hsa-let-7f-2 MI0000068
UGUGGGAUGAGGUAGUAGAUUGUAUAGUUUUAGGGUCAUACCCCAUCUUGGAGAUAACUAUACAG
UCUACUGUCUUUCCCACG
>hsa-let-7g MI0000433
AGGCUGAGGUAGUAGUUUGUACAGUUUGAGGGUCUAUGAUACCACCCGGUACAGGAGAUAACUGU
ACAGGCCACUGCCUUGCCA
>hsa-let-7i MI0000434
CUGGCUGAGGUAGUAGUUUGUGCUGUUGGUCGGGUUGUGACAUUGCCCGCUGUGGAGAUAACUGC
GCAAGCUACUGCCUUGCUA
>hsa-mir-1-2 MI0000437
ACCUACUCAGAGUACAUACUUCUUUAUGUACCCAUAUGAACAUACAAUGCUAUGGAAUGUAAAGA
AGUAUGUAUUUUUGGUAGGC
>hsa-mir-7-1 MI0000263
UUGGAUGUUGGCCUAGUUCUGUGUGGAAGACUAGUGAUUUUGUUGUUUUUAGAUAACUAAAUCGA
CAACAAAUCACAGUCUGCCAUAUGGCACAGGCCAUGCCUCUACAG
>hsa-mir-7-2 MI0000264
CUGGAUACAGAGUGGACCGGCUGGCCCCAUCUGGAAGACUAGUGAUUUUGUUGUUGUCUUACUGC
GCUCAACAACAAAUCCCAGUCUACCUAAUGGUGCCAGCCAUCGCA
>hsa-mir-7-3 MI0000265
AGAUUAGAGUGGCUGUGGUCUAGUGCUGUGUGGAAGACUAGUGAUUUUGUUGUUCUGAUGUACUA
CGACAACAAGUCACAGCCGGCCUCAUAGCGCAGACUCCCUUCGAC
>hsa-mir-9-1 MI0000466
CGGGGUUGGUUGUUAUCUUUGGUUAUCUAGCUGUAUGAGUGGUGUGGAGUCUUCAUAAAGCUAGA
UAACCGAAAGUAAAAAUAACCCCA
>hsa-mir-9-3 MI0000468
GGAGGCCCGUUUCUCUCUUUGGUUAUCUAGCUGUAUGAGUGCCACAGAGCCGUCAUAAAGCUAGA
UAACCGAAAGUAGAAAUGAUUCUCA

>hsa-mir-10a MI0000266
GAUCUGUCUGUCUUCUGUAUAUACCCUGUAGAUCCGAAUUUGUGUAAGGAAUUUUGUGGUCACAA
AUUCGUAUCUAGGGGAAUAUGUAGUUGACAUAAACACUCCGCUCU
>hsa-mir-10b MI0000267
CCAGAGGUUGUAACGUUGUCUAUAUAUACCCUGUAGAACCGAAUUUGUGUGGUAUCCGUAUAGUC
ACAGAUUCGAUUCUAGGGGAAUAUAUGGUCGAUGCAAAAACUUCA
>hsa-mir-15a MI0000069
CCUUGGAGUAAAGUAGCAGCACAUAAUGGUUUGUGGAUUUUGAAAAGGUGCAGGCCAUAUUGUGC
UGCCUCAAAAAUACAAGG
>hsa-mir-15b MI0000438
UUGAGGCCUUAAAGUACUGUAGCAGCACAUCAUGGUUUACAUGCUACAGUCAAGAUGCGAAUCAU
UAUUUGCUGCUCUAGAAAUUUAAGGAAAUUCAU
>hsa-mir-16-1 MI0000070
GUCAGCAGUGCCUUAGCAGCACGUAAAUAUUGGCGUUAAGAUUCUAAAAUUAUCUCCAGUAUUAA
CUGUGCUGCUGAAGUAAGGUUGAC
>hsa-mir-16-2 MI0000115
GUUCCACUCUAGCAGCACGUAAAUAUUGGCGUAGUGAAAUAUAUAUUAAACACCAAUAUUACUGU
GCUGCUUUAGUGUGAC
>hsa-mir-17 MI0000071
GUCAGAAUAAUGUCAAAGUGCUUACAGUGCAGGUAGUGAUAUGUGCAUCUACUGCAGUGAAGGCA
CUUGUAGCAUUAUGGUGAC
>hsa-mir-18a MI0000072
UGUUCUAAGGUGCAUCUAGUGCAGAUAGUGAAGUAGAUUAGCAUCUACUGCCCUAAGUGCUCCUU
CUGGCA
>hsa-mir-19a MI0000073
GCAGUCCUCUGUUAGUUUUGCAUAGUUGCACUACAAGAAGAAUGUAGUUGUGCAAAUCUAUGCAA
AACUGAUGGUGGCCUGC
>hsa-mir-19b-2 MI0000075
ACAUUGCUACUUACAAUUAGUUUUGCAGGUUUGCAUUUCAGCGUAUAUAUGUAUAUGUGGCUGUG
CAAAUCCAUGCAAAACUGAUUGUGAUAAUGU
>hsa-mir-20a MI0000076
GUAGCACUAAAGUGCUUAUAGUGCAGGUAGUGUUUAGUUAUCUACUGCAUUAUGAGCACUUAAAG
UACUGC
>hsa-mir-20b MI0001519
AGUACCAAAGUGCUCAUAGUGCAGGUAGUUUUGGCAUGACUCUACUGUAGUAUGGGCACUUCCAG
UACU
>hsa-mir-21 MI0000077
UGUCGGGUAGCUUAUCAGACUGAUGUUGACUGUUGAAUCUCAUGGCAACACCAGUCGAUGGGCUG
UCUGACA
>hsa-mir-22 MI0000078
GGCUGAGCCGCAGUAGUUCUUCAGUGGCAAGCUUUAUGUCCUGACCCAGCUAAAGCUGCCAGUUG
AAGAACUGUUGCCCUCUGCC
>hsa-mir-23a MI0000079
GGCCGGCUGGGGUUCCUGGGGAUGGGAUUUGCUUCCUGUCACAAAUCACAUUGCCAGGGAUUUCC

AACCGACC
>hsa-mir-23b MI0000439
CUCAGGUGCUCUGGCUGCUUGGGUUCCUGGCAUGCUGAUUUGUGACUUAAGAUUAAAAUCACAUU
GCCAGGGAUUACCACGCAACCACGACCUUGGC
>hsa-mir-23c MI0016010
AGUGACUUUCCAGGUGUCACACAGUGAGUGGCAUAAUCAGAGUACAAUUUGAGUCAUGCCCAUAC
AUCACAUUGCCAGUGAUUACCCAAGGAAAGUGACG
>hsa-mir-24-2 MI0000081
CUCUGCCUCCCGUGCCUACUGAGCUGAAACACAGUUGGUUUGUGUACACUGGCUCAGUUCAGCAG
GAACAGGG
>hsa-mir-25 MI0000082
GGCCAGUGUUGAGAGGCGGAGACUUGGGCAAUUGCUGGACGCUGCCCUGGGCAUUGCACUUGUCU
CGGUCUGACAGUGCCGGCC
>hsa-mir-26a-1 MI0000083
GUGGCCUCGUUCAAGUAAUCCAGGAUAGGCUGUGCAGGUCCCAAUGGGCCUAUUCUUGGUUACUU
GCACGGGGACGC
>hsa-mir-26a-2 MI0000750
GGCUGUGGCUGGAUUCAAGUAAUCCAGGAUAGGCUGUUUCCAUCUGUGAGGCCUAUUCUUGAUUA
CUUGUUUCUGGAGGCAGCU
>hsa-mir-26b MI0000084
CCGGGACCCAGUUCAAGUAAUUCAGGAUAGGUUGUGUGCUGUCCAGCCUGUUCUCCAUUACUUGG
CUCGGGGACCGG
>hsa-mir-27a MI0000085
CUGAGGAGCAGGGCUUAGCUGCUUGUGAGCAGGGUCCACACCAAGUCGUGUUCACAGUGGCUAAG
UUCCGCCCCCCAG
>hsa-mir-27b MI0000440
ACCUCUCUAACAAGGUGCAGAGCUUAGCUGAUUGGUGAACAGUGAUUGGUUUCCGCUUUGUUCAC
AGUGGCUAAGUUCUGCACCUGAAGAGAAGGUG
>hsa-mir-28 MI0000086
GGUCCUUGCCCUCAAGGAGCUCACAGUCUAUUGAGUUACCUUUCUGACUUUCCCACUAGAUUGUG
AGCUCCUGGAGGGCAGGCACU
>hsa-mir-29b-1 MI0000105
CUUCAGGAAGCUGGUUUCAUAUGGUGGUUUAGAUUUAAAUAGUGAUUGUCUAGCACCAUUUGAAA
UCAGUGUUCUUGGGGG
>hsa-mir-29c MI0000735
AUCUCUUACACAGGCUGACCGAUUUCUCCUGGUGUUCAGAGUCUGUUUUUGUCUAGCACCAUUUG
AAAUCGGUUAUGAUGUAGGGGGA
>hsa-mir-30b MI0000441
ACCAAGUUUCAGUUCAUGUAAACAUCCUACACUCAGCUGUAAUACAUGGAUUGGCUGGGAGGUGG
AUGUUUACUUCAGCUGACUUGGA
>hsa-mir-30c-1 MI0000736
ACCAUGCUGUAGUGUGUGUAAACAUCCUACACUCUCAGCUGUGAGCUCAAGGUGGCUGGGAGAGG
GUUGUUUACUCCUUCUGCCAUGGA
>hsa-mir-30d MI0000255

GUUGUUGUAAACAUCCCCGACUGGAAGCUGUAAGACACAGCUAAGCUUUCAGUCAGAUGUUUGCU
GCUAC
>hsa-mir-30e MI0000749
GGGCAGUCUUUGCUACUGUAAACAUCCUUGACUGGAAGCUGUAAGGUGUUCAGAGGAGCUUUCAG
UCGGAUGUUUACAGCGGCAGGCUGCCA
>hsa-mir-31 MI0000089
GGAGAGGAGGCAAGAUGCUGGCAUAGCUGUUGAACUGGGAACCUGCUAUGCCAACAUAUUGCCAU
CUUUCC
>hsa-mir-32 MI0000090
GGAGAUAUUGCACAUUACUAAGUUGCAUGUUGUCACGGCCUCAAUGCAAUUUAGUGUGUGUGAUA
UUUUC
>hsa-mir-33a MI0000091
CUGUGGUGCAUUGUAGUUGCAUUGCAUGUUCUGGUGGUACCCAUGCAAUGUUUCCACAGUGCAUC
ACAG
>hsa-mir-33b MI0003646
GCGGGCGGCCCCGCGGUGCAUUGCUGUUGCAUUGCACGUGUGUGAGGCGGGUGCAGUGCCUCGGC
AGUGCAGCCCGGAGCCGGCCCCUGGCACCAC
>hsa-mir-34a MI0000268
GGCCAGCUGUGAGUGUUUCUUUGGCAGUGUCUUAGCUGGUUGUUGUGAGCAAUAGUAAGGAAGCA
AUCAGCAAGUAUACUGCCCUAGAAGUGCUGCACGUUGUGGGGCCC
>hsa-mir-34b MI0000742
GUGCUCGGUUUGUAGGCAGUGUCAUUAGCUGAUUGUACUGUGGUGGUUACAAUCACUAACUCCAC
UGCCAUCAAAACAAGGCAC
>hsa-mir-34c MI0000743
AGUCUAGUUACUAGGCAGUGUAGUUAGCUGAUUGCUAAUAGUACCAAUCACUAACCACACGGCCA
GGUAAAAAGAUU
>hsa-mir-92a-1 MI0000093
CUUUCUACACAGGUUGGGAUCGGUUGCAAUGCUGUGUUUCUGUAUGGUAUUGCACUUGUCCCGGC
CUGUUGAGUUUGG
>hsa-mir-92a-2 MI0000094
UCAUCCCUGGGUGGGGAUUUGUUGCAUUACUUGUGUUCUAUAUAAAGUAUUGCACUUGUCCCGGC
CUGUGGAAGA
>hsa-mir-92b MI0003560
CGGGCCCCGGGCGGGCGGGAGGGACGGGACGCGGUGCAGUGUUGUUUUUUCCCCCGCCAAUAUUG
CACUCGUCCCGGCCUCCGGCCCCCCCGGCCC
>hsa-mir-93 MI0000095
CUGGGGGCUCCAAAGUGCUGUUCGUGCAGGUAGUGUGAUUACCCAACCUACUGCUGAGCUAGCAC
UUCCCGAGCCCCCGG
>hsa-mir-95 MI0000097
AACACAGUGGGCACUCAAUAAAUGUCUGUUGAAUUGAAAUGCGUUACAUUCAACGGGUAUUUAUU
GAGCACCCACUCUGUG
>hsa-mir-96 MI0000098
UGGCCGAUUUUGGCACUAGCACAUUUUUGCUUGUGUCUCUCCGCUCUGAGCAAUCAUGUGCAGUG
CCAAUAUGGGAAA

>hsa-mir-98 MI0000100
AGGAUUCUGCUCAUGCCAGGGUGAGGUAGUAAGUUGUAUUGUUGUGGGGUAGGGAUAUUAGGCCC
CAAUUAGAAGAUAACUAUACAACUUACUACUUUCCCUGGUGUGUGGCAUAUUCA
>hsa-mir-99a MI0000101
CCCAUUGGCAUAAACCCGUAGAUCCGAUCUUGUGGUGAAGUGGACCGCACAAGCUCGCUUCUAUG
GGUCUGUGUCAGUGUG
>hsa-mir-99b MI0000746
GGCACCCACCCGUAGAACCGACCUUGCGGGGCCUUCGCCGCACACAAGCUCGUGUCUGUGGGUCC
GUGUC
>hsa-mir-100 MI0000102
CCUGUUGCCACAAACCCGUAGAUCCGAACUUGUGGUAUUAGUCCGCACAAGCUUGUAUCUAUAGG
UAUGUGUCUGUUAGG
>hsa-mir-101-1 MI0000103
UGCCCUGGCUCAGUUAUCACAGUGCUGAUGCUGUCUAUUCUAAAGGUACAGUACUGUGAUAACUG
AAGGAUGGCA
>hsa-mir-101-2 MI0000739
ACUGUCCUUUUUCGGUUAUCAUGGUACCGAUGCUGUAUAUCUGAAAGGUACAGUACUGUGAUAAC
UGAAGAAUGGUGGU
>hsa-mir-103b-1 MI0007261
UCAUAGCCCUGUACAAUGCUGCUUGAUCCAUAUGCAACAAGGCAGCACUGUAAAGAAGCCGA
>hsa-mir-105-1 MI0000111
UGUGCAUCGUGGUCAAAUGCUCAGACUCCUGUGGUGGCUGCUCAUGCACCACGGAUGUUUGAGCA
UGUGCUACGGUGUCUA
>hsa-mir-106a MI0000113
CCUUGGCCAUGUAAAAGUGCUUACAGUGCAGGUAGCUUUUUGAGAUCUACUGCAAUGUAAGCACU
UCUUACAUUACCAUGG
>hsa-mir-106b MI0000734
CCUGCCGGGGCUAAAGUGCUGACAGUGCAGAUAGUGGUCCUCUCCGUGCUACCGCACUGUGGGUA
CUUGCUGCUCCAGCAGG
>hsa-mir-107 MI0000114
CUCUCUGCUUUCAGCUUCUUUACAGUGUUGCCUUGUGGCAUGGAGUUCAAGCAGCAUUGUACAGG
GCUAUCAAAGCACAGA
>hsa-mir-122 MI0000442
CCUUAGCAGAGCUGUGGAGUGUGACAAUGGUGUUUGUGUCUAAACUAUCAAACGCCAUUAUCACA
CUAAAUAGCUACUGCUAGGC
>hsa-mir-124-2 MI0000444
AUCAAGAUUAGAGGCUCUGCUCUCCGUGUUCACAGCGGACCUUGAUUUAAUGUCAUACAAUUAAG
GCACGCGGUGAAUGCCAAGAGCGGAGCCUACGGCUGCACUUGAA
>hsa-mir-125a MI0000469
UGCCAGUCUCUAGGUCCCUGAGACCCUUUAACCUGUGAGGACAUCCAGGGUCACAGGUGAGGUUC
UUGGGAGCCUGGCGUCUGGCC
>hsa-mir-125b-1 MI0000446
UGCGCUCCUCUCAGUCCCUGAGACCCUAACUUGUGAUGUUUACCGUUUAAAUCCACGGGUUAGGC
UCUUGGGAGCUGCGAGUCGUGCU

```
>hsa-mir-125b-2 MI0000470
ACCAGACUUUUCCUAGUCCCUGAGACCCUAACUUGUGAGGUAUUUUAGUAACAUCACAAGUCAGG
CUCUUGGGACCUAGGCGGAGGGGA
>hsa-mir-126 MI0000471
CGCUGGCGACGGGACAUUAUUACUUUUGGUACGCGCUGUGACACUUCAAACUCGUACCGUGAGUA
AUAAUGCGCCGUCCACGGCA
>hsa-mir-127 MI0000472
UGUGAUCACUGUCUCCAGCCUGCUGAAGCUCAGAGGGCUCUGAUUCAGAAAGAUCAUCGGAUCCG
UCUGAGCUUGGCUGGUCGGAAGUCUCAUCAUC
>hsa-mir-128-1 MI0000447
UGAGCUGUUGGAUUCGGGGCCGUAGCACUGUCUGAGAGGUUUACAUUUCUCACAGUGAACCGGUC
UCUUUUUCAGCUGCUUC
>hsa-mir-128-2 MI0000727
UGUGCAGUGGGAAGGGGGGCCGAUACACUGUACGAGAGUGAGUAGCAGGUCUCACAGUGAACCGG
UCUCUUUCCCUACUGUGUC
>hsa-mir-129-2 MI0000473
UGCCCUUCGCGAAUCUUUUUGCGGUCUGGGCUUGCUGUACAUAACUCAAUAGCCGGAAGCCCUUA
CCCCAAAAAGCAUUUGCGGAGGGCG
>hsa-mir-130a MI0000448
UGCUGCUGGCCAGAGCUCUUUUCACAUUGUGCUACUGUCUGCACCUGUCACUAGCAGUGCAAUGU
UAAAAGGGCAUUGGCCGUGUAGUG
>hsa-mir-130b MI0000748
GGCCUGCCCGACACUCUUUCCCUGUUGCACUACUAUAGGCCGCUGGGAAGCAGUGCAAUGAUGAA
AGGGCAUCGGUCAGGUC
>hsa-mir-132 MI0000449
CCGCCCCCGCGUCUCCAGGGCAACCGUGGCUUUCGAUUGUUACUGUGGGAACUGGAGGUAACAGU
CUACAGCCAUGGUCGCCCCGCAGCACGCCCACGCGC
>hsa-mir-133a-2 MI0000451
GGGAGCCAAAUGCUUUGCUAGAGCUGGUAAAAUGGAACCAAAUCGACUGUCCAAUGGAUUUGGUC
CCCUUCAACCAGCUGUAGCUGUGCAUUGAUGGCGCCG
>hsa-mir-133b MI0000822
CCUCAGAAGAAAGAUGCCCCCUGCUCUGGCUGGUCAAACGGAACCAAGUCCGUCUUCCUGAGAGG
UUUGGUCCCCUUCAACCAGCUACAGCAGGGCUGGCAAUGCCCAGUCCUUGGAGA
>hsa-mir-134 MI0000474
CAGGGUGUGUGACUGGUUGACCAGAGGGGCAUGCACUGUGUUCACCCUGUGGGCCACCUAGUCAC
CAACCCUC
>hsa-mir-135a-1 MI0000452
AGGCCUCGCUGUUCUCUAUGGCUUUUUAUUCCUAUGUGAUUCUACUGCUCACUCAUAUAGGGAUU
GGAGCCGUGGCGCACGGCGGGGACA
>hsa-mir-135a-2 MI0000453
AGAUAAAUUCACUCUAGUGCUUUAUGGCUUUUUAUUCCUAUGUGAUAGUAAUAAAGUCUCAUGUA
GGGAUGGAAGCCAUGAAAUACAUUGUGAAAAAUCA
>hsa-mir-135b MI0000810
CACUCUGCUGUGGCCUAUGGCUUUUCAUUCCUAUGUGAUUGCUGUCCCAAACUCAUGUAGGGCUA
```

AAAGCCAUGGGCUACAGUGAGGGGCGAGCUCC
>hsa-mir-136 MI0000475
UGAGCCCUCGGAGGACUCCAUUUGUUUUGAUGAUGGAUUCUUAUGCUCCAUCAUCGUCUCAAAUG
AGUCUUCAGAGGGUUCU
>hsa-mir-137 MI0000454
GGUCCUCUGACUCUCUUCGGUGACGGGUAUUCUUGGGUGGAUAAUACGGAUUACGUUGUUAUUGC
UUAAGAAUACGCGUAGUCGAGGAGAGUACCAGCGGCA
>hsa-mir-138-1 MI0000476
CCCUGGCAUGGUGUGGUGGGGCAGCUGGUGUUGUGAAUCAGGCCGUUGCCAAUCAGAGAACGGCU
ACUUCACAACACCAGGGCCACACCACACUACAGG
>hsa-mir-138-2 MI0000455
CGUUGCUGCAGCUGGUGUUGUGAAUCAGGCCGACGAGCAGCGCAUCCUCUUACCCGGCUAUUUCA
CGACACCAGGGUUGCAUCA
>hsa-mir-139 MI0000261
GUGUAUUCUACAGUGCACGUGUCUCCAGUGUGGCUCGGAGGCUGGAGACGCGGCCCUGUUGGAGU
AAC
>hsa-mir-140 MI0000456
UGUGUCUCUCUCUGUGUCCUGCCAGUGGUUUUACCCUAUGGUAGGUUACGUCAUGCUGUUCUACC
ACAGGGUAGAACCACGGACAGGAUACCGGGGCACC
>hsa-mir-141 MI0000457
CGGCCGGCCCUGGGUCCAUCUUCCAGUACAGUGUUGGAUGGUCUAAUUGUGAAGCUCCUAACACU
GUCUGGUAAAGAUGGCUCCCGGGUGGGUUC
>hsa-mir-142 MI0000458
GACAGUGCAGUCACCCAUAAAGUAGAAAGCACUACUAACAGCACUGGAGGGUGUAGUGUUUCCUA
CUUUAUGGAUGAGUGUACUGUG
>hsa-mir-143 MI0000459
GCGCAGCGCCCUGUCUCCCAGCCUGAGGUGCAGUGCUGCAUCUCUGGUCAGUUGGGAGUCUGAGA
UGAAGCACUGUAGCUCAGGAAGAGAGAAGUUGUUCUGCAGC
>hsa-mir-144 MI0000460
UGGGGCCCUGGCUGGGAUAUCAUCAUAUACUGUAAGUUUGCGAUGAGACACUACAGUAUAGAUGA
UGUACUAGUCCGGGCACCCCC
>hsa-mir-145 MI0000461
CACCUUGUCCUCACGGUCCAGUUUUCCCAGGAAUCCCUUAGAUGCUAAGAUGGGGAUUCCUGGAA
AUACUGUUCUUGAGGUCAUGGUU
>hsa-mir-146a MI0000477
CCGAUGUGUAUCCUCAGCUUUGAGAACUGAAUUCCAUGGGUUGUGUCAGUGUCAGACCUCUGAAA
UUCAGUUCUUCAGCUGGGAUAUCUCUGUCAUCGU
>hsa-mir-146b MI0003129
CCUGGCACUGAGAACUGAAUUCCAUAGGCUGUGAGCUCUAGCAAUGCCCUGUGGACUCAGUUCUG
GUGCCCGG
>hsa-mir-147b MI0005544
UAUAAAUCUAGUGGAAACAUUUCUGCACAAACUAGAUUCUGGACACCAGUGUGCGGAAAUGCUUC
UGCUACAUUUUUAGG
>hsa-mir-148a MI0000253

GAGGCAAAGUUCUGAGACACUCCGACUCUGAGUAUGAUAGAAGUCAGUGCACUACAGAACUUUGU
CUC
>hsa-mir-148b MI0000811
CAAGCACGAUUAGCAUUUGAGGUGAAGUUCUGUUAUACACUCAGGCUGUGGCUCUCUGAAAGUCA
GUGCAUCACAGAACUUUGUCUCGAAAGCUUUCUA
>hsa-mir-149 MI0000478
GCCGGCGCCCGAGCUCUGGCUCCGUGUCUUCACUCCCGUGCUUGUCCGAGGAGGGAGGGAGGGAC
GGGGGCUGUGCUGGGGCAGCUGGA
>hsa-mir-150 MI0000479
CUCCCCAUGGCCCUGUCUCCCAACCCUUGUACCAGUGCUGGGCUCAGACCCUGGUACAGGCCUGG
GGGACAGGGACCUGGGGAC
>hsa-mir-151a MI0000809
UUUCCUGCCCUCGAGGAGCUCACAGUCUAGUAUGUCUCAUCCCCUACUAGACUGAAGCUCCUUGA
GGACAGGGAUGGUCAUACUCACCUC
>hsa-mir-151b MI0003772
ACCUCUGAUGUGUCAGUCUCUCUUCAGGGCUCCCGAGACACAGAAACAGACACCUGCCCUCGAGG
AGCUCACAGUCUAGACAAACAAACCCAGGGU
>hsa-mir-152 MI0000462
UGUCCCCCCCGGCCCAGGUUCUGUGAUACACUCCGACUCGGGCUCUGGAGCAGUCAGUGCAUGAC
AGAACUUGGGCCCGGAAGGACC
>hsa-mir-153-1 MI0000463
CUCACAGCUGCCAGUGUCAUUUUUGUGAUCUGCAGCUAGUAUUCUCACUCCAGUUGCAUAGUCAC
AAAAGUGAUCAUUGGCAGGUGUGGC
>hsa-mir-154 MI0000480
GUGGUACUUGAAGAUAGGUUAUCCGUGUUGCCUUCGCUUUAUUUGUGACGAAUCAUACACGGUUG
ACCUAUUUUUCAGUACCAA
>hsa-mir-155 MI0000681
CUGUUAAUGCUAAUCGUGAUAGGGGUUUUUGCCUCCAACUGACUCCUACAUAUUAGCAUUAACAG
>hsa-mir-181a-1 MI0000289
UGAGUUUUGAGGUUGCUUCAGUGAACAUUCAACGCUGUCGGUGAGUUUGGAAUUAAAAUCAAAAC
CAUCGACCGUUGAUUGUACCCUAUGGCUAACCAUCAUCUACUCCA
>hsa-mir-181a-2 MI0000269
AGAAGGGCUAUCAGGCCAGCCUUCAGAGGACUCCAAGGAACAUUCAACGCUGUCGGUGAGUUUGG
GAUUUGAAAAAACCACUGACCGUUGACUGUACCUUGGGGUCCUUA
>hsa-mir-181b-1 MI0000270
CCUGUGCAGAGAUUAUUUUUUAAAAGGUCACAAUCAACAUUCAUUGCUGUCGGUGGGUUGAACUG
UGUGGACAAGCUCACUGAACAAUGAAUGCAACUGUGGCCCCGCUU
>hsa-mir-181b-2 MI0000683
CUGAUGGCUGCACUCAACAUUCAUUGCUGUCGGUGGGUUUGAGUCUGAAUCAACUCACUGAUCAA
UGAAUGCAAACUGCGGACCAAACA
>hsa-mir-181c MI0000271
CGGAAAAUUUGCCAAGGGUUUGGGGGAACAUUCAACCUGUCGGUGAGUUUGGGCAGCUCAGGCAA
ACCAUCGACCGUUGAGUGGACCCUGAGGCCUGGAAUUGCCAUCCU
>hsa-mir-181d MI0003139

```
GUCCCCUCCCCUAGGCCACAGCCGAGGUCACAAUCAACAUUCAUUGUUGUCGGUGGGUUGUGAGG
ACUGAGGCCAGACCCACCGGGGGAUGAAUGUCACUGUGGCUGGGCCAGACACGGCUUAAGGGGAA
UGGGGAC
>hsa-mir-182 MI0000272
GAGCUGCUUGCCUCCCCCCGUUUUUGGCAAUGGUAGAACUCACACUGGUGAGGUAACAGGAUCCG
GUGGUUCUAGACUUGCCAACUAUGGGGCGAGGACUCAGCCGGCAC
>hsa-mir-183 MI0000273
CCGCAGAGUGUGACUCCUGUUCUGUGUAUGGCACUGGUAGAAUUCACUGUGAACAGUCUCAGUCA
GUGAAUUACCGAAGGGCCAUAAACAGAGCAGAGACAGAUCCACGA
>hsa-mir-184 MI0000481
CCAGUCACGUCCCCUUAUCACUUUUCCAGCCCAGCUUUGUGACUGUAAGUGUUGGACGGAGAACU
GAUAAGGGUAGGUGAUUGA
>hsa-mir-185 MI0000482
AGGGGGCGAGGGAUUGGAGAGAAAGGCAGUUCCUGAUGGUCCCCUCCCCAGGGGCUGGCUUUCCU
CUGGUCCUUCCCUCCCA
>hsa-mir-186 MI0000483
UGCUUGUAACUUUCCAAAGAAUUCUCCUUUUGGGCUUUCUGGUUUUAUUUUAAGCCCAAAGGUGA
AUUUUUUGGGAAGUUUGAGCU
>hsa-mir-187 MI0000274
GGUCGGGCUCACCAUGACACAGUGUGAGACCUCGGGCUACAACACAGGACCCGGGCGCUGCUCUG
ACCCCUCGUGUCUUGUGUUGCAGCCGGAGGGACGCAGGUCCGCA
>hsa-mir-188 MI0000484
UGCUCCCUCUCUCACAUCCCUUGCAUGGUGGAGGGUGAGCUUUCUGAAAACCCCUCCCACAUGCA
GGGUUUGCAGGAUGGCGAGCC
>hsa-mir-190a MI0000486
UGCAGGCCUCUGUGUGAUAUGUUUGAUAUAUUAGGUUGUUAUUUAAUCCAACUAUAUAUCAAACA
UAUUCCUACAGUGUCUUGCC
>hsa-mir-190b MI0005545
UGCUUCUGUGUGAUAUGUUUGAUAUUGGGUUGUUUAAUUAGGAACCAACUAAAUGUCAAACAUAU
UCUUACAGCAGCAG
>hsa-mir-191 MI0000465
CGGCUGGACAGCGGGCAACGGAAUCCCAAAAGCAGCUGUUGUCUCCAGAGCAUUCCAGCUGCGCU
UGGAUUUCGUCCCCUGCUCUCCUGCCU
>hsa-mir-192 MI0000234
GCCGAGACCGAGUGCACAGGGCUCUGACCUAUGAAUUGACAGCCAGUGCUCUCGUCUCCCCUCUG
GCUGCCAAUUCCAUAGGUCACAGGUAUGUUCGCCUCAAUGCCAGC
>hsa-mir-193a MI0000487
CGAGGAUGGGAGCUGAGGGCUGGGUCUUUGCGGGCGAGAUGAGGGUGUCGGAUCAACUGGCCUAC
AAAGUCCCAGUUCUCGGCCCCCG
>hsa-mir-193b MI0003137
GUGGUCUCAGAAUCGGGGUUUUGAGGGCGAGAUGAGUUUAUGUUUUAUCCAACUGGCCCUCAAAG
UCCCGCUUUUGGGGUCAU
>hsa-mir-194-1 MI0000488
AUGGUGUUAUCAAGUGUAACAGCAACUCCAUGUGGACUGUGUACCAAUUUCCAGUGGAGAUGCUG
```

UUACUUUUGAUGGUUACCAA
>hsa-mir-194-2 MI0000732
UGGUUCCCGCCCCCUGUAACAGCAACUCCAUGUGGAAGUGCCCACUGGUUCCAGUGGGGCUGCUG
UUAUCUGGGGCGAGGGCCAG
>hsa-mir-195 MI0000489
AGCUUCCCUGGCUCUAGCAGCACAGAAAUAUUGGCACAGGGAAGCGAGUCUGCCAAUAUUGGCUG
UGCUGCUCCAGGCAGGGUGGUG
>hsa-mir-196a-1 MI0000238
GUGAAUUAGGUAGUUUCAUGUUGUUGGGCCUGGGUUUCUGAACACAACAACAUUAAACCACCCGA
UUCAC
>hsa-mir-196a-2 MI0000279
UGCUCGCUCAGCUGAUCUGUGGCUUAGGUAGUUUCAUGUUGUUGGGAUUGAGUUUUGAACUCGGC
AACAAGAAACUGCCUGAGUUACAUCAGUCGGUUUUCGUCGAGGGC
>hsa-mir-196b MI0001150
ACUGGUCGGUGAUUUAGGUAGUUUCCUGUUGUUGGGAUCCACCUUUCUCUCGACAGCACGACACU
GCCUUCAUUACUUCAGUUG
>hsa-mir-197 MI0000239
GGCUGUGCCGGGUAGAGAGGGCAGUGGGAGGUAAGAGCUCUUCACCCUUCACCACCUUCUCCACC
CAGCAUGGCC
>hsa-mir-198 MI0000240
UCAUUGGUCCAGAGGGGAGAUAGGUUCCUGUGAUUUUUCCUUCUUCUCUAUAGAAUAAAUGA
>hsa-mir-199a-2 MI0000281
AGGAAGCUUCUGGAGAUCCUGCUCCGUCGCCCCAGUGUUCAGACUACCUGUUCAGGACAAUGCCG
UUGUACAGUAGUCUGCACAUUGGUUAGACUGGGCAAGGGAGAGCA
>hsa-mir-199b MI0000282
CCAGAGGACACCUCCACUCCGUCUACCCAGUGUUUAGACUAUCUGUUCAGGACUCCCAAAUUGUA
CAGUAGUCUGCACAUUGGUUAGGCUGGGCUGGGUUAGACCCUCGG
>hsa-mir-200a MI0000737
CCGGGCCCCUGUGAGCAUCUUACCGGACAGUGCUGGAUUUCCCAGCUUGACUCUAACACUGUCUG
GUAACGAUGUUCAAAGGUGACCCGC
>hsa-mir-200b MI0000342
CCAGCUCGGGCAGCCGUGGCCAUCUUACUGGGCAGCAUUGGAUGGAGUCAGGUCUCUAAUACUGC
CUGGUAAUGAUGACGGCGGAGCCCUGCACG
>hsa-mir-200c MI0000650
CCCUCGUCUUACCCAGCAGUGUUUGGGUGCGGUUGGGAGUCUCUAAUACUGCCGGGUAAUGAUGG
AGG
>hsa-mir-202 MI0003130
CGCCUCAGAGCCGCCCGCCGUUCCUUUUUCCUAUGCAUAUACUUCUUUGAGGAUCUGGCCUAAAG
AGGUAUAGGGCAUGGGAAAACGGGGCGGUCGGGUCCUCCCCAGCG
>hsa-mir-203a MI0000283
GUGUUGGGGACUCGCGCGCUGGGUCCAGUGGUUCUUAACAGUUCAACAGUUCUGUAGCGCAAUUG
UGAAAUGUUUAGGACCACUAGACCCGGCGGGCGCGGCGACAGCGA
>hsa-mir-203b MI0017343
GCGCCCGCCGGGUCUAGUGGUCCUAAACAUUUCACAAUUGCGCUACAGAACUGUUGAACUGUUAA

GAACCACUGGACCCAGCGCGC
>hsa-mir-204 MI0000284
GGCUACAGUCUUUCUUCAUGUGACUCGUGGACUUCCCUUUGUCAUCCUAUGCCUGAGAAUAUAUG
AAGGAGGCUGGGAAGGCAAAGGGACGUUCAAUUGUCAUCACUGGC
>hsa-mir-205 MI0000285
AAAGAUCCUCAGACAAUCCAUGUGCUUCUCUUGUCCUUCAUUCCACCGGAGUCUGUCUCAUACCC
AACCAGAUUUCAGUGGAGUGAAGUUCAGGAGGCAUGGAGCUGACA
>hsa-mir-206 MI0000490
UGCUUCCCGAGGCCACAUGCUUCUUUAUAUCCCCAUAUGGAUUACUUUGCUAUGGAAUGUAAGGA
AGUGUGUGGUUUCGGCAAGUG
>hsa-mir-208a MI0000251
UGACGGGCGAGCUUUUGGCCCGGGUUAUACCUGAUGCUCACGUAUAAGACGAGCAAAAAGCUUGU
UGGUCA
>hsa-mir-208b MI0005570
CCUCUCAGGGAAGCUUUUUGCUCGAAUUAUGUUUCUGAUCCGAAUAUAAGACGAACAAAAGGUUU
GUCUGAGGGCAG
>hsa-mir-210 MI0000286
ACCCGGCAGUGCCUCCAGGCGCAGGGCAGCCCCUGCCCACCGCACACUGCGCUGCCCCAGACCCA
CUGUGCGUGUGACAGCGGCUGAUCUGUGCCUGGGCAGCGCGACCC
>hsa-mir-211 MI0000287
UCACCUGGCCAUGUGACUUGUGGGCUUCCCUUUGUCAUCCUUCGCCUAGGGCUCUGAGCAGGGCA
GGGACAGCAAAGGGGUGCUCAGUUGUCACUUCCCACAGCACGGAG
>hsa-mir-212 MI0000288
CGGGGCACCCCGCCCGGACAGCGCGCCGGCACCUUGGCUCUAGACUGCUUACUGCCCGGGCCGCC
CUCAGUAACAGUCUCCAGUCACGGCCACCGACGCCUGGCCCCGCC
>hsa-mir-214 MI0000290
GGCCUGGCUGGACAGAGUUGUCAUGUGUCUGCCUGUCUACACUUGCUGUGCAGAACAUCCGCUCA
CCUGUACAGCAGGCACAGACAGGCAGUCACAUGACAACCCAGCCU
>hsa-mir-215 MI0000291
AUCAUUCAGAAAUGGUAUACAGGAAAAUGACCUAUGAAUUGACAGACAAUAUAGCUGAGUUUGUC
UGUCAUUUCUUUAGGCCAAUAUUCUGUAUGACUGUGCUACUUCAA
>hsa-mir-216a MI0000292
GAUGGCUGUGAGUUGGCUUAAUCUCAGCUGGCAACUGUGAGAUGUUCAUACAAUCCCUCACAGUG
GUCUCUGGGAUUAUGCUAAACAGAGCAAUUUCCUAGCCCUCACGA
>hsa-mir-216b MI0005569
GCAGACUGGAAAAUCUCUGCAGGCAAAUGUGAUGUCACUGAGGAAAUCACACACUUACCCGUAGA
GAUUCUACAGUCUGACA
>hsa-mir-217 MI0000293
AGUAUAAUUAUUACAUAGUUUUUGAUGUCGCAGAUACUGCAUCAGGAACUGAUUGGAUAAGAAUC
AGUCACCAUCAGUUCCUAAUGCAUUGCCUUCAGCAUCUAAACAAG
>hsa-mir-218-1 MI0000294
GUGAUAAUGUAGCGAGAUUUUCUGUUGUGCUUGAUCUAACCAUGUGGUUGCGAGGUAUGAGUAAA
ACAUGGUUCCGUCAAGCACCAUGGAACGUCACGCAGCUUUCUACA
>hsa-mir-218-2 MI0000295

GACCAGUCGCUGCGGGGCUUUCCUUUGUGCUUGAUCUAACCAUGUGGUGGAACGAUGGAAACGGA
ACAUGGUUCUGUCAAGCACCGCGGAAAGCACCGUGCUCUCCUGCA
>hsa-mir-219a-1 MI0000296
CCGCCCCGGGCCGCGGCUCCUGAUUGUCCAAACGCAAUUCUCGAGUCUAUGGCUCCGGCCGAGAG
UUGAGUCUGGACGUCCCGAGCCGCCGCCCCCAAACCUCGAGCGGG
>hsa-mir-219a-2 MI0000740
ACUCAGGGGCUUCGCCACUGAUUGUCCAAACGCAAUUCUUGUACGAGUCUGCGGCCAACCGAGAA
UUGUGGCUGGACAUCUGUGGCUGAGCUCCGGG
>hsa-mir-219b MI0017299
GGAGCUCAGCCACAGAUGUCCAGCCACAAUUCUCGGUUGGCCGCAGACUCGUACAAGAAUUGCGU
UUGGACAAUCAGUGGCGAAGCCC
>hsa-mir-221 MI0000298
UGAACAUCCAGGUCUGGGGCAUGAACCUGGCAUACAAUGUAGAUUUCUGUGUUCGUUAGGCAACA
GCUACAUUGUCUGCUGGGUUUCAGGCUACCUGGAAACAUGUUCUC
>hsa-mir-222 MI0000299
GCUGCUGGAAGGUGUAGGUACCCUCAAUGGCUCAGUAGCCAGUGUAGAUCCUGUCUUUCGUAAUC
AGCAGCUACAUCUGGCUACUGGGUCUCUGAUGGCAUCUUCUAGCU
>hsa-mir-223 MI0000300
CCUGGCCUCCUGCAGUGCCACGCUCCGUGUAUUUGACAAGCUGAGUUGGACACUCCAUGUGGUAG
AGUGUCAGUUUGUCAAAUACCCCAAGUGCGGCACAUGCUUACCAG
>hsa-mir-224 MI0000301
GGGCUUUCAAGUCACUAGUGGUUCCGUUUAGUAGAUGAUUGUGCAUUGUUUCAAAAUGGUGCCCU
AGUGACUACAAAGCCC
>hsa-mir-296 MI0000747
AGGACCCUUCCAGAGGGCCCCCCCUCAAUCCUGUUGUGCCUAAUUCAGAGGGUUGGGUGGAGGCU
CUCCUGAAGGGCUCU
>hsa-mir-297 MI0005775
UGUAUGUAUGUGUGCAUGUGCAUGUAUGUGUAUAUACAUAUAUAUGUAUUAUGUACUCAUAUAUC
A
>hsa-mir-298 MI0005523
UCAGGUCUUCAGCAGAAGCAGGGAGGUUCUCCCAGUGGUUUUCCUUGACUGUGAGGAACUAGCCU
GCUGCUUUGCUCAGGAGUGAGCU
>hsa-mir-299 MI0000744
AAGAAAUGGUUUACCGUCCCACAUACAUUUUGAAUAUGUAUGUGGGAUGGUAAACCGCUUCUU
>hsa-mir-300 MI0005525
UGCUACUUGAAGAGAGGUAAUCCUUCACGCAUUUGCUUUACUUGCAAUGAUUAUACAAGGGCAGA
CUCUCUCUGGGGAGCAAA
>hsa-mir-301a MI0000745
ACUGCUAACGAAUGCUCUGACUUUAUUGCACUACUGUACUUUACAGCUAGCAGUGCAAUAGUAUU
GUCAAAGCAUCUGAAAGCAGG
>hsa-mir-301b MI0005568
GCCGCAGGUGCUCUGACGAGGUUGCACUACUGUGCUCUGAGAAGCAGUGCAAUGAUAUUGUCAAA
GCAUCUGGGACCA
>hsa-mir-302a MI0000738

CCACCACUUAAACGUGGAUGUACUUGCUUUGAAACUAAAGAAGUAAGUGCUUCCAUGUUUUGGUG
AUGG
>hsa-mir-302b MI0000772
GCUCCCUUCAACUUUAACAUGGAAGUGCUUUCUGUGACUUUAAAAGUAAGUGCUUCCAUGUUUUA
GUAGGAGU
>hsa-mir-302c MI0000773
CCUUUGCUUUAACAUGGGGGUACCUGCUGUGUGAAACAAAAGUAAGUGCUUCCAUGUUUCAGUGG
AGG
>hsa-mir-302e MI0006417
UUGGGUAAGUGCUUCCAUGCUUCAGUUUCCUUACUGGUAAGAUGGAUGUAGUAAUAGCACCUACC
UUAUAGA
>hsa-mir-302f MI0006418
UCUGUGUAAACCUGGCAAUUUUCACUUAAUUGCUUCCAUGUUUAUAAAAGA
>hsa-mir-320a MI0000542
GCUUCGCUCCCCUCCGCCUUCUCUUCCCGGUUCUUCCCGGAGUCGGGAAAAGCUGGGUUGAGAGG
GCGAAAAAGGAUGAGGU
>hsa-mir-320b-1 MI0003776
AAUUAAUCCCUCUCUUUCUAGUUCUUCCUAGAGUGAGGAAAAGCUGGGUUGAGAGGGCAAACAAA
UUAACUAAUUAAUU
>hsa-mir-320b-2 MI0003839
UGUUAUUUUUUGUCUUCUACCUAAGAAUUCUGUCUCUUAGGCUUUCUCUUCCCAGAUUUCCCAAA
GUUGGGAAAAGCUGGGUUGAGAGGGCAAAAGGAAAAAAAAAGAAUUCUGUCUCUGACAUAAUUAG
AUAGGGAA
>hsa-mir-320c-1 MI0003778
UUUGCAUUAAAAAUGAGGCCUUCUCUUCCCAGUUCUUCCCAGAGUCAGGAAAAGCUGGGUUGAGA
GGGUAGAAAAAAAAUGAUGUAGG
>hsa-mir-323a MI0000807
UUGGUACUUGGAGAGAGGUGGUCCGUGGCGCGUUCGCUUUAUUUAUGGCGCACAUUACACGGUCG
ACCUCUUUGCAGUAUCUAAUC
>hsa-mir-324 MI0000813
CUGACUAUGCCUCCCCGCAUCCCCUAGGGCAUUGGUGUAAAGCUGGAGACCCACUGCCCCAGGUG
CUGCUGGGGGUUGUAGUC
>hsa-mir-325 MI0000824
AUACAGUGCUUGGUUCCUAGUAGGUGUCCAGUAAGUGUUUGUGACAUAAUUUGUUUAUUGAGGAC
CUCCUAUCAAUCAAGCACUGUGCUAGGCUCUGG
>hsa-mir-326 MI0000808
CUCAUCUGUCUGUUGGGCUGGAGGCAGGGCCUUUGUGAAGGCGGGUGGUGCUCAGAUCGCCUCUG
GGCCCUUCCUCCAGCCCCGAGGCGGAUUCA
>hsa-mir-328 MI0000804
UGGAGUGGGGGGGCAGGAGGGGCUCAGGGAGAAAGUGCAUACAGCCCCUGGCCCUCUCUGCCCUU
CCGUCCCCUG
>hsa-mir-329-2 MI0001726
GUGGUACCUGAAGAGAGGUUUUCUGGGUUUCUGUUUCUUUAUUGAGGACGAAACACACCUGGUUA
ACCUCUUUUCCAGUAUCAA

>hsa-mir-330 MI0000803
CUUUGGCGAUCACUGCCUCUCUGGGCCUGUGUCUUAGGCUCUGCAAGAUCAACCGAGCAAAGCAC
ACGGCCUGCAGAGAGGCAGCGCUCUGCCC
>hsa-mir-331 MI0000812
GAGUUUGGUUUUGUUUGGGUUUGUUCUAGGUAUGGUCCCAGGGAUCCCAGAUCAAACCAGGCCCC
UGGGCCUAUCCUAGAACCAACCUAAGCUC
>hsa-mir-335 MI0000816
UGUUUUGAGCGGGGGUCAAGAGCAAUAACGAAAAAUGUUUGUCAUAAACCGUUUUUCAUUAUUGC
UCCUGACCUCCUCUCAUUUGCUAUAUUCA
>hsa-mir-337 MI0000806
GUAGUCAGUAGUUGGGGGGUGGGAACGGCUUCAUACAGGAGUUGAUGCACAGUUAUCCAGCUCCU
AUAUGAUGCCUUUCUUCAUCCCCUUCAA
>hsa-mir-338 MI0000814
UCUCCAACAAUAUCCUGGUGCUGAGUGAUGACUCAGGCGACUCCAGCAUCAGUGAUUUUGUUGAA
GA
>hsa-mir-339 MI0000815
CGGGGCGGCCGCUCUCCCUGUCCUCCAGGAGCUCACGUGUGCCUGCCUGUGAGCGCCUCGACGAC
AGAGCCGGCGCCUGCCCCAGUGUCUGCGC
>hsa-mir-340 MI0000802
UUGUACCUGGUGUGAUUAUAAAGCAAUGAGACUGAUUGUCAUAUGUCGUUUGUGGGAUCCGUCUC
AGUUACUUUAUAGCCAUACCUGGUAUCUUA
>hsa-mir-342 MI0000805
GAAACUGGGCUCAAGGUGAGGGGUGCUAUCUGUGAUUGAGGGACAUGGUUAAUGGAAUUGUCUCA
CACAGAAAUCGCACCCGUCACCUUGGCCUACUUA
>hsa-mir-345 MI0000825
ACCCAAACCCUAGGUCUGCUGACUCCUAGUCCAGGGCUCGUGAUGGCUGGUGGGCCCUGAACGAG
GGGUCUGGAGGCCUGGGUUUGAAUAUCGACAGC
>hsa-mir-346 MI0000826
GGUCUCUGUGUUGGGCGUCUGUCUGCCCGCAUGCCUGCCUCUCUGUUGCUCUGAAGGAGGCAGGG
GCUGGGCCUGCAGCUGCCUGGGCAGAGCGG
>hsa-mir-361 MI0000760
GGAGCUUAUCAGAAUCUCCAGGGGUACUUUAUAAUUUCAAAAAGUCCCCCAGGUGUGAUUCUGAU
UUGCUUC
>hsa-mir-362 MI0000762
CUUGAAUCCUUGGAACCUAGGUGUGAGUGCUAUUUCAGUGCAACACACCUAUUCAAGGAUUCAAA
>hsa-mir-363 MI0000764
UGUUGUCGGGUGGAUCACGAUGCAAUUUUGAUGAGUAUCAUAGGAGAAAAAUUGCACGGUAUCCA
UCUGUAAACC
>hsa-mir-365b MI0000769
AGAGUGUUCAAGGACAGCAAGAAAAAUGAGGGACUUUCAGGGGCAGCUGUGUUUUCUGACUCAGU
CAUAAUGCCCCUAAAAAUCCUUAUUGUUCUUGCAGUGUGCAUCGGG
>hsa-mir-367 MI0000775
CCAUUACUGUUGCUAAUAUGCAACUCUGUUGAAUAUAAAUUGGAAUUGCACUUUAGCAAUGGUGA
UGG

>hsa-mir-369 MI0000777
UUGAAGGGAGAUCGACCGUGUUAUAUUCGCUUUAUUGACUUCGAAUAAUACAUGGUUGAUCUUUU
CUCAG
>hsa-mir-370 MI0000778
AGACAGAGAAGCCAGGUCACGUCUCUGCAGUUACACAGCUCACGAGUGCCUGCUGGGGUGGAACC
UGGUCUGUCU
>hsa-mir-371a MI0000779
GUGGCACUCAAACUGUGGGGGCACUUUCUGCUCUCUGGUGAAAGUGCCGCCAUCUUUUGAGUGUU
AC
>hsa-mir-371b MI0017393
GGUAACACUCAAAAGAUGGCGGCACUUUCACCAGAGAGCAGAAAGUGCCCCCACAGUUUGAGUGC
C
>hsa-mir-372 MI0000780
GUGGGCCUCAAAUGUGGAGCACUAUUCUGAUGUCCAAGUGGAAAGUGCUGCGACAUUUGAGCGUC
AC
>hsa-mir-373 MI0000781
GGGAUACUCAAAAUGGGGGCGCUUUCCUUUUUGUCUGUACUGGGAAGUGCUUCGAUUUUGGGGUG
UCCC
>hsa-mir-374a MI0000782
UACAUCGGCCAUUAUAAUACAACCUGAUAAGUGUUAUAGCACUUAUCAGAUUGUAUUGUAAUUGU
CUGUGUA
>hsa-mir-374b MI0005566
ACUCGGAUGGAUAUAAUACAACCUGCUAAGUGUCCUAGCACUUAGCAGGUUGUAUUAUCAUUGUC
CGUGUCU
>hsa-mir-374c MI0016684
ACACGGACAAUGAUAAUACAACCUGCUAAGUGCUAGGACACUUAGCAGGUUGUAUUAUAUCCAUC
CGAGU
>hsa-mir-375 MI0000783
CCCCGCGACGAGCCCCUCGCACAAACCGGACCUGAGCGUUUUGUUCGUUCGGCUCGCGUGAGGC
>hsa-mir-376b MI0002466
CAGUCCUUCUUUGGUAUUUAAAACGUGGAUAUUCCUUCUAUGUUUACGUGAUUCCUGGUUAAUCA
UAGAGGAAAAUCCAUGUUUUCAGUAUCAAAUGCUG
>hsa-mir-377 MI0000785
UUGAGCAGAGGUUGCCCUUGGUGAAUUCGCUUUAUUUAUGUUGAAUCACACAAAGGCAACUUUUG
UUUG
>hsa-mir-378a MI0000786
AGGGCUCCUGACUCCAGGUCCUGUGUGUUACCUAGAAAUAGCACUGGACUUGGAGUCAGAAGGCC
U
>hsa-mir-378b MI0014154
GGUCAUUGAGUCUUCAAGGCUAGUGGAAAGAGCACUGGACUUGGAGGCAGAAAGACC
>hsa-mir-378c MI0015825
GGAGGCCAUCACUGGACUUGGAGUCAGAAGAGUGGAGUCGGGUCAGACUUCAACUCUGACUUUGA
AGGUGGUGAGUGCCUC
>hsa-mir-378d-1 MI0016749

ACUGUUUCUGUCCUUGUUCUUGUUGUUAUUACUGGACUUGGAGUCAGAAACAGG
>hsa-mir-378d-2 MI0003840
GAAUGGUUACAAGGAGAGAACACUGGACUUGGAGUCAGAAAACUUUCAUCCAAGUCAUUCCCUGC
UCUAAGUCCCAUUUCUGUUCCAUGAGAUUGUUU
>hsa-mir-378e MI0016750
CUGACUCCAGUGUCCAGGCCAGGGGCAGACAGUGGACAGAGAACAGUGCCCAAGACCACUGGACU
UGGAGUCAGGACAU
>hsa-mir-378f MI0016756
GUCAGGUCCUGGACUCCCAUAGUUUUCAGGCUGCUAAACAACAGAACGAGCACUGGACUUGGAGC
CAGAAGUCUUGGG
>hsa-mir-378h MI0016808
ACAGGAACACUGGACUUGGUGUCAGAUGGGAUGAGCCCUGGCUCUGUUUCCUAGCAGCAAUCUGA
UCUUGAGCUAGUCACUGG
>hsa-mir-378i MI0016902
GGGAGCACUGGACUAGGAGUCAGAAGGUGGAGUUCUGGGUGCUGUUUUCCCACUCUUGGGCCCUG
GGCAUGUUCUG
>hsa-mir-378j MI0021273
AUGCAGUGAGUCGGGGAGGAACUGGAUUUGGAGCCAGAAGAACUGGUUCUAAUAUCUACUUCCCU
GUGUAGAGUUGGGAUUUGGGAUUAUAUGAGUUAAUAUACACCAA
>hsa-mir-379 MI0000787
AGAGAUGGUAGACUAUGGAACGUAGGCGUUAUGAUUUCUGACCUAUGUAACAUGGUCCACUAACU
CU
>hsa-mir-380 MI0000788
AAGAUGGUUGACCAUAGAACAUGCGCUAUCUCUGUGUCGUAUGUAAUAUGGUCCACAUCUU
>hsa-mir-381 MI0000789
UACUUAAAGCGAGGUUGCCCUUUGUAUAUUCGGUUUAUUGACAUGGAAUAUACAAGGGCAAGCUC
UCUGUGAGUA
>hsa-mir-382 MI0000790
UACUUGAAGAGAAGUUGUUCGUGGUGGAUUCGCUUUACUUAUGACGAAUCAUUCACGGACAACAC
UUUUUUCAGUA
>hsa-mir-383 MI0000791
CUCCUCAGAUCAGAAGGUGAUUGUGGCUUUGGGUGGAUAUUAAUCAGCCACAGCACUGCCUGGUC
AGAAAGAG
>hsa-mir-384 MI0001145
UGUUAAAUCAGGAAUUUUAAACAAUUCCUAGACAAUAUGUAUAAUGUUCAUAAGUCAUUCCUAGA
AAUUGUUCAUAAUGCCUGUAACA
>hsa-mir-409 MI0001735
UGGUACUCGGGGAGAGGUUACCCGAGCAACUUUGCAUCUGGACGACGAAUGUUGCUCGGUGAACC
CCUUUUCGGUAUCA
>hsa-mir-410 MI0002465
GGUACCUGAGAAGAGGUUGUCUGUGAUGAGUUCGCUUUUAUUAAUGACGAAUAUAACACAGAUGG
CCUGUUUUCAGUACC
>hsa-mir-411 MI0003675
UGGUACUUGGAGAGAUAGUAGACCGUAUAGCGUACGCUUUAUCUGUGACGUAUGUAACACGGUCC

ACUAACCCUCAGUAUCAAAUCCAUCCCCGAG
>hsa-mir-412 MI0002464
CUGGGGUACGGGGAUGGAUGGUCGACCAGUUGGAAAGUAAUUGUUUCUAAUGUACUUCACCUGGU
CCACUAGCCGUCCGUAUCCGCUGCAG
>hsa-mir-422a MI0001444
GAGAGAAGCACUGGACUUAGGGUCAGAAGGCCUGAGUCUCUCUGCUGCAGAUGGGCUCUCUGUCC
CUGAGCCAAGCUUUGUCCUCCCUGG
>hsa-mir-423 MI0001445
AUAAAGGAAGUUAGGCUGAGGGGCAGAGAGCGAGACUUUUCUAUUUUCCAAAAGCUCGGUCUGAG
GCCCCUCAGUCUUGCUUCCUAACCCGCGC
>hsa-mir-424 MI0001446
CGAGGGGAUACAGCAGCAAUUCAUGUUUUGAAGUGUUCUAAAUGGUUCAAAACGUGAGGCGCUGC
UAUACCCCCUCGUGGGGAAGGUAGAAGGUGGGG
>hsa-mir-425 MI0001448
GAAAGCGCUUUGGAAUGACACGAUCACUCCCGUUGAGUGGGCACCCGAGAAGCCAUCGGGAAUGU
CGUGUCCGCCCAGUGCUCUUUC
>hsa-mir-429 MI0001641
CGCCGGCCGAUGGGCGUCUUACCAGACAUGGUUAGACCUGGCCCUCUGUCUAAUACUGUCUGGUA
AAACCGUCCAUCCGCUGC
>hsa-mir-431 MI0001721
UCCUGCUUGUCCUGCGAGGUGUCUUGCAGGCCGUCAUGCAGGCCACACUGACGGUAACGUUGCAG
GUCGUCUUGCAGGGCUUCUCGCAAGACGACAUCCUCAUCACCAACGACG
>hsa-mir-432 MI0003133
UGACUCCUCCAGGUCUUGGAGUAGGUCAUUGGGUGGAUCCUCUAUUUCCUUACGUGGGCCACUGG
AUGGCUCCUCCAUGUCUUGGAGUAGAUCA
>hsa-mir-433 MI0001723
CCGGGGAGAAGUACGGUGAGCCUGUCAUUAUUCAGAGAGGCUAGAUCCUCUGUGUUGAGAAGGAU
CAUGAUGGGCUCCUCGGUGUUCUCCAGG
>hsa-mir-448 MI0001637
GCCGGGAGGUUGAACAUCCUGCAUAGUGCUGCCAGGAAAUCCCUAUUUCAUAUAAGAGGGGGCUG
GCUGGUUGCAUAUGUAGGAUGUCCCAUCUCCCAGCCCACUUCGUCA
>hsa-mir-449a MI0001648
CUGUGUGUGAUGAGCUGGCAGUGUAUUGUUAGCUGGUUGAAUAUGUGAAUGGCAUCGGCUAACAU
GCAACUGCUGUCUUAUUGCAUAUACA
>hsa-mir-449b MI0003673
UGACCUGAAUCAGGUAGGCAGUGUAUUGUUAGCUGGCUGCUUGGGUCAAGUCAGCAGCCACAACU
ACCCUGCCACUUGCUUCUGGAUAAAUUCUUCU
>hsa-mir-449c MI0003823
GCUGGGAUGUGUCAGGUAGGCAGUGUAUUGCUAGCGGCUGUUAAUGAUUUUAACAGUUGCUAGUU
GCACUCCUCUCUGUUGCAUUCAGAAGC
>hsa-mir-450a-2 MI0003187
CCAAAGAAAGAUGCUAAACUAUUUUUGCGAUGUGUUCCUAAUAUGUAAUAUAAAUGUAUUGGGGA
CAUUUUGCAUUCAUAGUUUUGUAUCAAUAAUAUGG
>hsa-mir-451a MI0001729

CUUGGGAAUGGCAAGGAAACCGUUACCAUUACUGAGUUUAGUAAUGGUAAUGGUUCUCUUGCUAU
ACCCAGA
>hsa-mir-451b MI0017360
UGGGUAUAGCAAGAGAACCAUUACCAUUACUAAACUCAGUAAUGGUAACGGUUUCCUUGCCAUUC
CCA
>hsa-mir-452 MI0001733
GCUAAGCACUUACAACUGUUUGCAGAGGAAACUGAGACUUUGUAACUAUGUCUCAGUCUCAUCUG
CAAAGAAGUAAGUGCUUUGC
>hsa-mir-454 MI0003820
UCUGUUUAUCACCAGAUCCUAGAACCCUAUCAAUAUUGUCUCUGCUGUGUAAAUAGUUCUGAGUA
GUGCAAUAUUGCUUAUAGGGUUUUGGUGUUUGGAAAGAACAAUGGGCAGG
>hsa-mir-455 MI0003513
UCCCUGGCGUGAGGGUAUGUGCCUUUGGACUACAUCGUGGAAGCCAGCACCAUGCAGUCCAUGGG
CAUAUACACUUGCCUCAAGGCCUAUGUCAUC
>hsa-mir-466 MI0014157
GUGUGUGUAUAUGUGUGUUGCAUGUGUGUAUAUGUGUGUAUAUAUGUACACAUACACAUACACGC
AACACACAUAUAUACAUGC
>hsa-mir-483 MI0002467
GAGGGGGAAGACGGGAGGAAAGAAGGGAGUGGUUCCAUCACGCCUCCUCACUCCUCUCCUCCCGU
CUUCUCCUCUC
>hsa-mir-484 MI0002468
AGCCUCGUCAGGCUCAGUCCCCUCCCGAUAAACCCCUAAAUAGGGACUUUCCCGGGGGGUGACCC
UGGCUUUUUUGGCG
>hsa-mir-485 MI0002469
ACUUGGAGAGAGGCUGGCCGUGAUGAAUUCGAUUCAUCAAAGCGAGUCAUACACGGCUCUCCUCU
CUUUUAGU
>hsa-mir-486 MI0002470
GCAUCCUGUACUGAGCUGCCCCGAGGCCCUUCAUGCUGCCCAGCUCGGGGCAGCUCAGUACAGGA
UAC
>hsa-mir-487b MI0003530
UUGGUACUUGGAGAGUGGUUAUCCCUGUCCUGUUCGUUUUGCUCAUGUCGAAUCGUACAGGGUCA
UCCACUUUUUCAGUAUCAA
>hsa-mir-488 MI0003123
GAGAAUCAUCUCUCCCAGAUAAUGGCACUCUCAAACAAGUUUCCAAAUUGUUUGAAAGGCUAUUU
CUUGGUCAGAUGACUCUC
>hsa-mir-489 MI0003124
GUGGCAGCUUGGUGGUCGUAUGUGUGACGCCAUUUACUUGAACCUUUAGGAGUGACAUCACAUAU
ACGGCAGCUAAACUGCUAC
>hsa-mir-490 MI0003125
UGGAGGCCUUGCUGGUUUGGAAAGUUCAUUGUUCGACACCAUGGAUCUCCAGGUGGGUCAAGUUU
AGAGAUGCACCAACCUGGAGGACUCCAUGCUGUUGAGCUGUUCACAAGCAGCGGACACUUCCA
>hsa-mir-491 MI0003126
UUGACUUAGCUGGGUAGUGGGGAACCCUUCCAUGAGGAGUAGAACACUCCUUAUGCAAGAUUCCC
UUCUACCUGGCUGGGUUGG

>hsa-mir-492 MI0003131
CAACUACAGCCACUACUACAGGACCAUCGAGGACCUGCGGGACAAGAUUCUUGGUGCCACCAUUG
AGAACGCCAGGAUUGUCCUGCAGAUCAACAAUGCUCAACUGGCUGCAGAUG
>hsa-mir-493 MI0003132
CUGGCCUCCAGGGCUUUGUACAUGGUAGGCUUUCAUUCAUUCGUUUGCACAUUCGGUGAAGGUCU
ACUGUGUGCCAGGCCCUGUGCCAG
>hsa-mir-494 MI0003134
GAUACUCGAAGGAGAGGUUGUCCGUGUUGUCUUCUCUUUAUUUAUGAUGAAACAUACACGGGAAA
CCUCUUUUUUAGUAUC
>hsa-mir-495 MI0003135
UGGUACCUGAAAAGAAGUUGCCCAUGUUAUUUUCGCUUUAUAUGUGACGAAACAAACAUGGUGCA
CUUCUUUUUCGGUAUCA
>hsa-mir-496 MI0003136
CCCAAGUCAGGUACUCGAAUGGAGGUUGUCCAUGGUGUGUUCAUUUUAUUUAUGAUGAGUAUUAC
AUGGCCAAUCUCCUUUCGGUACUCAAUUCUUCUUGGG
>hsa-mir-497 MI0003138
CCACCCCGGUCCUGCUCCCGCCCCAGCAGCACACUGUGGUUUGUACGGCACUGUGGCCACGUCCA
AACCACACUGUGGUGUUAGAGCGAGGGUGGGGGAGGCACCGCCGAGG
>hsa-mir-498 MI0003142
AACCCUCCUUGGGAAGUGAAGCUCAGGCUGUGAUUUCAAGCCAGGGGGCGUUUUUCUAUAACUGG
AUGAAAAGCACCUCCAGAGCUUGAAGCUCACAGUUUGAGAGCAAUCGUCUAAGGAAGUU
>hsa-mir-499a MI0003183
GCCCUGUCCCCUGUGCCUUGGGCGGGCGGCUGUUAAGACUUGCAGUGAUGUUUAACUCCUCUCCA
CGUGAACAUCACAGCAAGUCUGUGCUGCUUCCCGUCCCUACGCUGCCUGGGCAGGGU
>hsa-mir-499b MI0017396
GGAAGCAGCACAGACUUGCUGUGAUGUUCACGUGGAGAGGAGUUAAACAUCACUGCAAGUCUUAA
CAGCCGCC
>hsa-mir-502 MI0003186
UGCUCCCCCUCUCUAAUCCUUGCUAUCUGGGUGCUAGUGCUGGCUCAAUGCAAUGCACCUGGGCA
AGGAUUCAGAGAGGGGGAGCU
>hsa-mir-503 MI0003188
UGCCCUAGCAGCGGGAACAGUUCUGCAGUGAGCGAUCGGUGCUCUGGGGUAUUGUUUCCGCUGCC
AGGGUA
>hsa-mir-504 MI0003189
GCUGCUGUUGGGAGACCCUGGUCUGCACUCUAUCUGUAUUCUUACUGAAGGGAGUGCAGGGCAGG
GUUUCCCAUACAGAGGGC
>hsa-mir-505 MI0003190
GAUGCACCCAGUGGGGGAGCCAGGAAGUAUUGAUGUUUCUGCCAGUUUAGCGUCAACACUUGCUG
GUUUCCUCUCUGGAGCAUC
>hsa-mir-506 MI0003193
GCCACCACCAUCAGCCAUACUAUGUGUAGUGCCUUAUUCAGGAAGGUGUUACUUAAUAGAUUAAU
AUUUGUAAGGCACCCUUCUGAGUAGAGUAAUGUGCAACAUGGACAACAUUUGUGGUGGC
>hsa-mir-507 MI0003194
GUGCUGUGUGUAGUGCUUCACUUCAAGAAGUGCCAUGCAUGUGUCUAGAAAUAUGUUUUGCACCU

UUUGGAGUGAAAUAAUGCACAACAGAUAC
>hsa-mir-508 MI0003195
CCACCUUCAGCUGAGUGUAGUGCCCUACUCCAGAGGGCGUCACUCAUGUAAACUAAAACAUGAUU
GUAGCCUUUUGGAGUAGAGUAAUACACAUCACGUAACGCAUAUUUGGUGG
>hsa-mir-510 MI0003197
GUGGUGUCCUACUCAGGAGAGUGGCAAUCACAUGUAAUUAGGUGUGAUUGAAACCUCUAAGAGUG
GAGUAACAC
>hsa-mir-511 MI0003127
CAAUAGACACCCAUCGUGUCUUUUGCUCUGCAGUCAGUAAAUAUUUUUUUGUGAAUGUGUAGCAA
AAGACAGAAUGGUGGUCCAUUG
>hsa-mir-512-2 MI0003141
GGUACUUCUCAGUCUGUGGCACUCAGCCUUGAGGGCACUUUCUGGUGCCAGAAUGAAAGUGCUGU
CAUAGCUGAGGUCCAAUGACUGAGGCGAGCACC
>hsa-mir-513a-1 MI0003191
GGGAUGCCACAUUCAGCCAUUCAGCGUACAGUGCCUUUCACAGGGAGGUGUCAUUUAUGUGAACU
AAAAUAUAAAUUUCACCUUUCUGAGAAGGGUAAUGUACAGCAUGCACUGCAUAUGUGGUGUCCC
>hsa-mir-514a-1 MI0003198
AACAUGUUGUCUGUGGUACCCUACUCUGGAGAGUGACAAUCAUGUAUAAUUAAAUUUGAUUGACA
CUUCUGUGAGUAGAGUAACGCAUGACACGUACG
>hsa-mir-518c MI0003159
GCGAGAAGAUCUCAUGCUGUGACUCUCUGGAGGGAAGCACUUUCUGUUGUCUGAAAGAAAACAAA
GCGCUUCUCUUUAGAGUGUUACGGUUUGAGAAAAGC
>hsa-mir-519d MI0003162
UCCCAUGCUGUGACCCUCCAAAGGGAAGCGCUUUCUGUUUGUUUUCUCUUAAACAAAGUGCCUCC
CUUUAGAGUGUUACCGUUUGGGA
>hsa-mir-532 MI0003205
CGACUUGCUUUCUCUCCUCCAUGCCUUGAGUGUAGGACCGUUGGCAUCUUAAUUACCCUCCCACA
CCCAAGGCUUGCAAAAAAGCGAGCCU
>hsa-mir-541 MI0005539
ACGUCAGGGAAAGGAUUCUGCUGUCGGUCCCACUCCAAAGUUCACAGAAUGGGUGGUGGGCACAG
AAUCUGGACUCUGCUUGUG
>hsa-mir-542 MI0003686
CAGAUCUCAGACAUCUCGGGGAUCAUCAUGUCACGAGAUACCAGUGUGCACUUGUGACAGAUUGA
UAACUGAAAGGUCUGGGAGCCACUCAUCUUCA
>hsa-mir-544a MI0003515
AUUUUCAUCACCUAGGGAUCUUGUUAAAAAGCAGAUUCUGAUUCAGGGACCAAGAUUCUGCAUUU
UUAGCAAGUUCUCAAGUGAUGCUAAU
>hsa-mir-544b MI0014159
GGAAUUUUGUUAAAAUGCAGAAUCCAUUUCUGUAGCUCUGAGACUAGACCUGAGGUUGUGCAUUU
CUAACAAAGUGCC
>hsa-mir-545 MI0003516
CCCAGCCUGGCACAUUAGUAGGCCUCAGUAAAUGUUUAUUAGAUGAAUAAAUGAAUGACUCAUCA
GCAAACAUUUAUUGUGUGCCUGCUAAAGUGAGCUCCACAGG
>hsa-mir-548a-1 MI0003593

UGCAGGGAGGUAUUAAGUUGGUGCAAAAGUAAUUGUGAUUUUUGCCAUUAAAAGUAACGACAAAA
CUGGCAAUUACUUUUGCACCAAACCUGGUAUU
>hsa-mir-548a-3 MI0003612
CCUAGAAUGUUAUUAGGUCGGUGCAAAAGUAAUUGCGAGUUUUACCAUUACUUUCAAUGGCAAAA
CUGGCAAUUACUUUUGCACCAACGUAAUACUU
>hsa-mir-548al MI0016851
GGUCGGUGCAAAAGUAAUUGCUGUUUUUGCCAUUAAAAAUAAUGGCAUUAAAAGUAAUGGCAAAA
ACGGCAAUGACUUUUGUACCAAUCUAAUAUCU
>hsa-mir-548ao MI0017871
AACUAUUCUUAGGUUGAUGCAGAAGUAACUACGGUUUUUGCAGUUGAAAGUAAUGGCAAAGACCG
UGACUACUUUUGCAACAGCCUAAUAGUUUCU
>hsa-mir-548b MI0003596
CAGACUAUAUAUUUAGGUUGGCGCAAAAGUAAUUGUGGUUUUGGCCUUUAUUUUCAAUGGCAAGA
ACCUCAGUUGCUUUUGUGCCAACCUAAUACUU
>hsa-mir-548d-1 MI0003668
AAACAAGUUAUAUUAGGUUGGUGCAAAAGUAAUUGUGGUUUUUGCCUGUAAAAGUAAUGGCAAAA
ACCACAGUUUCUUUUGCACCAGACUAAUAAAG
>hsa-mir-548f-2 MI0006375
UAAUAACUAUUAGGUUGGUGCGAACAUAAUUGCAGUUUUUAUCAUUACUUUUAAUGGCAAAAACU
GUAAUUACUUUUGCACCAACCUAAUAUUUUAGU
>hsa-mir-548h-3 MI0006413
UCUGAUUCUGCAUGUAUUAGGUUGGUGCAAAAGUAAUCGCGGUUUUUGUCAUUGAAAGUAAUAGC
AAAAACUGCAAUUACUUUUGCACCAACCUAAAAGUAGUCACUGUCUUCAGAUA
>hsa-mir-548h-4 MI0006414
GCUAUUAGGUUGGUGCAAAAGUAAUCGCGGUUUUUGUCAUUACUUUAAUUACUUUACGUUUCAUU
AAUGACAAAAACCGCAAUUACUUUUGCACCAACCUAAUACUUGCUA
>hsa-mir-548i-1 MI0006421
CAGAUGGCUCUGAAGUUUGCACCCUAUUAGGUUGGUGCAAAAGUAAUUGCGGAUUUUGCCAUUAA
AAGUAAUGGCAAAAAUAGCAAUUAUUUUUGUACCAGCCUAGUAUCUUUUCUCCUUCUACCAAACU
UUGUCCCUGAGCCAUCUCA
>hsa-mir-548j MI0006345
GGGCAGCCAGUGAAUAGUUAGCUGGUGCAAAAGUAAUUGCGGUCUUUGGUAUUACUUUCAGUGGC
AAAAACUGCAUUACUUUUGCACCAGCCUACUAGAACGCUGAGUUCAG
>hsa-mir-548k MI0006354
CUUUUCUCAAGUAUUGCUGUUAGGUUGGUGCAAAAGUACUUGCGGAUUUUGCUUUACUUUUAAUG
GCAAAAACCGCAAUUAUUUUUGCUUCAACCUAAUAUGAUGCAAAAUUGGCU
>hsa-mir-548o MI0006402
UGGUGAAAAUGUGUUGAUUGUAAUGGUUCCUAUUCUGAUCAAUAAACAUGGUUUGAGCCUAGUUA
CAAUGAUCUAAAAUUCACGGUCCAAAACUGCAGUUACUUUUGCACCAAC
>hsa-mir-548q MI0010637
AUAUUAGGCUGGUGCAAAAGUAAUGGCGGUUUUUGCCAUUACUUUUCAUUUUUACCAUUAAAAGU
AAUGGCAAAAAGCAUGAUUACUUUUUCACCAACCU
>hsa-mir-548y MI0016595
GCCUAAACUAUUAGGUUGGUGCAAAAGUAAUCACUGUUUUUGCCAUUACUCUCAGUGGCAAAAAC

CGUGAUUACUUUUGCACCAACCUAGUAACACCUUCACUGUGGGGG
>hsa-mir-549a MI0003679
AGACAUGCAACUCAAGAAUAUAUUGAGAGCUCAUCCAUAGUUGUCACUGUCUCAAAUCAGUGACA
ACUAUGGAUGAGCUCUUAAUAUAUCCCAGGC
>hsa-mir-550a-1 MI0003600
UGAUGCUUUGCUGGCUGGUGCAGUGCCUGAGGGAGUAAGAGCCCUGUUGUUGUAAGAUAGUGUCU
UACUCCCUCAGGCACAUCUCCAACAAGUCUCU
>hsa-mir-550b-1 MI0016686
AGAGACUUGUUGGAGAUGUGCCUGAGGGAGUAAGACACUAUCUUACAACAACAGGGCUCUUACUC
CCUCAGGCACUGCACCAGCCAGCAAAGCAUCA
>hsa-mir-551a MI0003556
GGGGACUGCCGGGUGACCCUGGAAAUCCAGAGUGGGUGGGGCCAGUCUGACCGUUUCUAGGCGAC
CCACUCUUGGUUUCCAGGGUUGCCCUGGAAA
>hsa-mir-551b MI0003575
AGAUGUGCUCUCCUGGCCCAUGAAAUCAAGCGUGGGUGAGACCUGGUGCAGAACGGGAAGGCGAC
CCAUACUUGGUUUCAGAGGCUGUGAGAAUAA
>hsa-mir-552 MI0003557
AACCAUUCAAAUAUACCACAGUUUGUUUAACCUUUUGCCUGUUGGUUGAAGAUGCCUUUCAACAG
GUGACUGGUUAGACAAACUGUGGUAUAUACA
>hsa-mir-553 MI0003558
CUUCAAUUUUAUUUUAAAACGGUGAGAUUUUGUUUUGUCUGAGAAAAUCUCGCUGUUUUAGACUG
AGG
>hsa-mir-554 MI0003559
ACCUGAGUAACCUUUGCUAGUCCUGACUCAGCCAGUACUGGUCUUAGACUGGUGAUGGGUCAGGG
UUCAUAUUUUGGCAUCUCUCUCUGGGCAUCU
>hsa-mir-555 MI0003561
GGAGUGAACUCAGAUGUGGAGCACUACCUUUGUGAGCAGUGUGACCCAAGGCCUGUGGACAGGGU
AAGCUGAACCUCUGAUAAAACUCUGAUCUAU
>hsa-mir-556 MI0003562
GAUAGUAAUAAGAAAGAUGAGCUCAUUGUAAUAUGAGCUUCAUUUAUACAUUUCAUAUUACCAUU
AGCUCAUCUUUUUUAUUACUACCUUCAACA
>hsa-mir-557 MI0003563
AGAAUGGGCAAAUGAACAGUAAAUUUGGAGGCCUGGGGCCCUCCCUGCUGCUGGAGAAGUGUUUG
CACGGGUGGGCCUUGUCUUUGAAAGGAGGUGGA
>hsa-mir-558 MI0003564
GUGUGUGUGUGUGUGUGUGUGGUUAUUUUGGUAUAGUAGCUCUAGACUCUAUUAUAGUUUCCUGAGC
UGCUGUACCAAAAUACCACAAACGGGCUG
>hsa-mir-559 MI0003565
GCUCCAGUAACAUCUUAAAGUAAAUAUGCACCAAAAUUACUUUUGGUAAAUACAGUUUUGGUGCA
UAUUUACUUUAGGAUGUUACUGGAGCUCCCA
>hsa-mir-561 MI0003567
CUUCAUCCACCAGUCCUCCAGGAACAUCAAGGAUCUUAAACUUUGCCAGAGCUACAAAGGCAAAG
UUUAAGAUCCUUGAAGUUCCUGGGGGAACCAU
>hsa-mir-562 MI0003568

AGUGAAAUUGCUAGGUCAUAUGGUCAGUCUACUUUUAGAGUAAUUGUGAAACUGUUUUUCAAAGU
AGCUGUACCAUUUGCACUCCCUGUGGCAAU
>hsa-mir-563 MI0003569
AGCAAAGAAGUGUGUUGCCCUCUAGGAAAUGUGUGUUGCUCUGAUGUAAUUAGGUUGACAUACGU
UUCCCUGGUAGCCA
>hsa-mir-564 MI0003570
CGGGCAGCGGGUGCCAGGCACGGUGUCAGCAGGCAACAUGGCCGAGAGGCCGGGGCCUCCGGGCG
GCGCCGUGUCCGCGACCGCGUACCCUGAC
>hsa-mir-566 MI0003572
GCUAGGCGUGGUGGCGGGCGCCUGUGAUCCCAACUACUCAGGAGGCUGGGGCAGCAGAAUCGCUU
GAACCCGGGAGGCGAAGGUUGCAGUGAGC
>hsa-mir-567 MI0003573
GGAUUCUUAUAGGACAGUAUGUUCUUCCAGGACAGAACAUUCUUUGCUAUUUUGUACUGGAAGAA
CAUGCAAAACUAAAAAAAAAAAAAAGUUAUUGCU
>hsa-mir-568 MI0003574
GAUAUACACUAUAUUAUGUAUAAAUGUAUACACACUUCCUAUAUGUAUCCACAUAUAUAUAGUGU
AUAUAUUAUACAUGUAUAGGUGUGUAUAUG
>hsa-mir-569 MI0003576
GGUAUUGUUAGAUUAAUUUUGUGGGACAUUAACAACAGCAUCAGAAGCAACAUCAGCUUUAGUUA
AUGAAUCCUGGAAAGUUAAGUGACUUUAUUU
>hsa-mir-571 MI0003578
CCUCAGUAAGACCAAGCUCAGUGUGCCAUUUCCUUGUCUGUAGCCAUGUCUAUGGGCUCUUGAGU
UGGCCAUCUGAGUGAGGGCCUGCUUAUUCUA
>hsa-mir-572 MI0003579
GUCGAGGCCGUGGCCCGGAAGUGGUCGGGGCCGCUGCGGGCGGAAGGGCGCCUGUGCUUCGUCCG
CUCGGCGGUGGCCCAGCCAGGCCCGCGGGA
>hsa-mir-573 MI0003580
UUUAGCGGUUUCUCCCUGAAGUGAUGUGUAACUGAUCAGGAUCUACUCAUGUCGUCUUUGGUAAA
GUUAUGUCGCUUGUCAGGGUGAGGAGAGUUUUUG
>hsa-mir-574 MI0003581
GGGACCUGCGUGGGUGCGGGCGUGUGAGUGUGUGUGUGUGAGUGUGUGUCGCUCCGGGUCCACGC
UCAUGCACACACCCACACGCCCACACUCAGG
>hsa-mir-575 MI0003582
AAUUCAGCCCUGCCACUGGCUUAUGUCAUGACCUUGGGCUACUCAGGCUGUCUGCACAAUGAGCC
AGUUGGACAGGAGCAGUGCCACUCAACUC
>hsa-mir-576 MI0003583
UACAAUCCAACGAGGAUUCUAAUUUCUCCACGUCUUUGGUAAUAAGGUUUGGCAAAGAUGUGGAA
AAAUUGGAAUCCUCAUUCGAUUGGUUAUAACCA
>hsa-mir-577 MI0003584
UGGGGGAGUGAAGAGUAGAUAAAAUAUUGGUACCUGAUGAAUCUGAGGCCAGGUUUCAAUACUUU
AUCUGCUCUUCAUUUCCCCAUAUCUACUUAC
>hsa-mir-578 MI0003585
AGAUAAAUCUAUAGACAAAAUACAAUCCCGGACAACAAGAAGCUCCUAUAGCUCCUGUAGCUUCU
UGUGCUCUAGGAUUGUAUUUUGUUUAUAUAU

>hsa-mir-579 MI0003586
CAUAUUAGGUUAAUGCAAAAGUAAUCGCGGUUUGUGCCAGAUGACGAUUUGAAUUAAUAAAUUCA
UUUGGUAUAAACCGCGAUUAUUUUUGCAUCAAC
>hsa-mir-580 MI0003587
AUAAAAUUUCCAAUUGGAACCUAAUGAUUCAUCAGACUCAGAUAUUUAAGUUAACAGUAUUUGAG
AAUGAUGAAUCAUUAGGUUCCGGUCAGAAAUU
>hsa-mir-581 MI0003588
GUUAUGUGAAGGUAUUCUUGUGUUCUCUAGAUCAGUGCUUUUAGAAAAUUUGUGUGAUCUAAAGA
ACACAAAGAAUACCUACACAGAACCACCUGC
>hsa-mir-582 MI0003589
AUCUGUGCUCUUUGAUUACAGUUGUUCAACCAGUUACUAAUCUAACUAAUUGUAACUGGUUGAAC
AACUGAACCCAAAGGGUGCAAAGUAGAAACAUU
>hsa-mir-583 MI0003590
AACUCACACAUUAACCAAAGAGGAAGGUCCCAUUACUGCAGGGAUCUUAGCAGUACUGGGACCUA
CCUCUUUGGU
>hsa-mir-584 MI0003591
UAGGGUGACCAGCCAUUAUGGUUUGCCUGGGACUGAGGAAUUUGCUGGGAUAUGUCAGUUCCAGG
CCAACCAGGCUGGUUGGUCUCCCUGAAGCAAC
>hsa-mir-585 MI0003592
UGGGGUGUCUGUGCUAUGGCAGCCCUAGCACACAGAUACGCCCAGAGAAAGCCUGAACGUUGGGC
GUAUCUGUAUGCUAGGGCUGCUGUAACAA
>hsa-mir-586 MI0003594
AUGGGGUAAAACCAUUAUGCAUUGUAUUUUUAGGUCCCAAUACAUGUGGGCCCUAAAAAUACAAU
GCAUAAUGGUUUUUCACUCUUUAUCUUCUUAU
>hsa-mir-587 MI0003595
CUCCUAUGCACCCUCUUUCCAUAGGUGAUGAGUCACAGGGCUCAGGGAAUGUGUCUGCACCUGUG
ACUCAUCACCAGUGGAAAGCCCAUCCCAUAU
>hsa-mir-588 MI0003597
AGCUUAGGUACCAAUUUGGCCACAAUGGGUUAGAACACUAUUCCAUUGUGUUCUUACCCACCAUG
GCCAAAAUUGGGCCUAAG
>hsa-mir-589 MI0003599
UCCAGCCUGUGCCCAGCAGCCCCUGAGAACCACGUCUGCUCUGAGCUGGGUACUGCCUGUUCAGA
ACAAAUGCCGGUUCCCAGACGCUGCCAGCUGGCC
>hsa-mir-590 MI0003602
UAGCCAGUCAGAAAUGAGCUUAUUCAUAAAAGUGCAGUAUGGUGAAGUCAAUCUGUAAUUUUAUG
UAUAAGCUAGUCUCUGAUUGAAACAUGCAGCA
>hsa-mir-591 MI0003603
UCUUAUCAAUGAGGUAGACCAUGGGUUCUCAUUGUAAUAGUGUAGAAUGUUGGUUAACUGUGGAC
UCCCUGGCUCUGUCUCAAAUCUACUGAUUC
>hsa-mir-592 MI0003604
UAUUAUGCCAUGACAUUGUGUCAAUAUGCGAUGAUGUGUUGUGAUGGCACAGCGUCAUCACGUGG
UGACGCAACAUCAUGACGUAAGACGUCACAAC
>hsa-mir-593 MI0003605
CCCCCAGAAUCUGUCAGGCACCAGCCAGGCAUUGCUCAGCCCGUUUCCCUCUGGGGGAGCAAGGA

GUGGUGCUGGGUUUGUCUCUGCUGGGGUUUCUCCU
>hsa-mir-595 MI0003607
ACGGAAGCCUGCACGCAUUUAACACCAGCACGCUCAAUGUAGUCUUGUAAGGAACAGGUUGAAGU
GUGCCGUGGUGUGUCUGGAGGAAGCGCCUGU
>hsa-mir-596 MI0003608
AGCACGGCCUCUCCGAAGCCUGCCCGGCUCCUCGGGAACCUGCCUCCCGCAUGGCAGCUGCUGCC
CUUCGGAGGCCG
>hsa-mir-597 MI0003609
UACUUACUCUACGUGUGUGUCACUCGAUGACCACUGUGAAGACAGUAAAAUGUACAGUGGUUCUC
UUGUGGCUCAAGCGUAAUGUAGAGUACUGGUC
>hsa-mir-598 MI0003610
GCUUGAUGAUGCUGCUGAUGCUGGCGGUGAUCCCGAUGGUGUGAGCUGGAAAUGGGGUGCUACGU
CAUCGUUGUCAUCGUCAUCAUCAUCAUCCGAG
>hsa-mir-599 MI0003611
AAAGACAUGCUGUCCACAGUGUGUUUGAUAAGCUGACAUGGGACAGGGAUUCUUUUCACUGUUGU
GUCAGUUUAUCAAACCCAUACUUGGAUGAC
>hsa-mir-600 MI0003613
AAGUCACGUGCUGUGGCUCCAGCUUCAUAGGAAGGCUCUUGUCUGUCAGGCAGUGGAGUUACUUA
CAGACAAGAGCCUUGCUCAGGCCAGCCCUGCCC
>hsa-mir-601 MI0003614
UGCAUGAGUUCGUCUUGGUCUAGGAUUGUUGGAGGAGUCAGAAAAACUACCCCAGGGAUCCUGAA
GUCCUUUGGGUGGA
>hsa-mir-602 MI0003615
UUCUCACCCCCGCCUGACACGGGCGACAGCUGCGGCCCGCUGUGUUCACUCGGGCCGAGUGCGUC
UCCUGUCAGGCAAGGGAGAGCAGAGCCCCCCUG
>hsa-mir-604 MI0003617
AGAGCAUCGUGCUUGACCUUCCACGCUCUCGUGUCCACUAGCAGGCAGGUUUUCUGACACAGGCU
GCGGAAUUCAGGACAGUGCAUCAUGGAGA
>hsa-mir-605 MI0003618
GCCCUAGCUUGGUUCUAAAUCCCAUGGUGCCUUCUCCUUGGGAAAAACAGAGAAGGCACUAUGAG
AUUUAGAAUCAAGUUAGG
>hsa-mir-606 MI0003619
UGUAUCCUUGGUUUUUAGUAGUUUUACUAUGAUGAGGUGUGCCAUCCACCCCAUCAUAGUAAACU
ACUGAAAAUCAAAGAUACAAGUGCCUGACCA
>hsa-mir-607 MI0003620
UUGCCUAAAGUCACACAGGUUAUAGAUCUGGAUUGGAACCCAGGGAGCCAGACUGCCUGGGUUCA
AAUCCAGAUCUAUAACUUGUGUGACUUUGGG
>hsa-mir-608 MI0003621
GGGCCAAGGUGGGCCAGGGGUGGUGUUGGGACAGCUCCGUUUAAAAAGGCAUCUCCAAGAGCUUC
CAUCAAAGGCUGCCUCUUGGUGCAGCACAGGUAGA
>hsa-mir-609 MI0003622
UGCUCGGCUGUUCCUAGGGUGUUUCUCUCAUCUCUGGUCUAUAAUGGGUUAAAUAGUAGAGAUGA
GGGCAACACCCUAGGAACAGCAGAGGAACC
>hsa-mir-610 MI0003623

UCUAUUUGUCUUAGGUGAGCUAAAUGUGUGCUGGGACACAUUUGAGCCAAAUGUCCCAGCACACA
UUUAGCUCACAUAAGAAAAAUGGACUCUAGU
>hsa-mir-611 MI0003624
AAAAUGGUGAGAGCGUUGAGGGGAGUUCCAGACGGAGAUGCGAGGACCCCUCGGGGUCUGACCCA
CA
>hsa-mir-612 MI0003625
UCCCAUCUGGACCCUGCUGGGCAGGGCUUCUGAGCUCCUUAGCACUAGCAGGAGGGGCUCCAGGG
GCCCUCCCUCCAUGGCAGCCAGGACAGGACUCUCA
>hsa-mir-613 MI0003626
GGUGAGUGCGUUUCCAAGUGUGAAGGGACCCUUCCUGUAGUGUCUUAUAUACAAUACAGUAGGAA
UGUUCCUUCUUUGCCACUCAUACACCUUUA
>hsa-mir-614 MI0003627
UCUAAGAAACGCAGUGGUCUCUGAAGCCUGCAGGGGCAGGCCAGCCCUGCACUGAACGCCUGUUC
UUGCCAGGUGGCAGAAGGUUGCUGC
>hsa-mir-615 MI0003628
CUCGGGAGGGGCGGGAGGGGGGUCCCCGGUGCUCGGAUCUCGAGGGUGCUUAUUGUUCGGUCCGA
GCCUGGGUCUCCCUCUUCCCCCCAACCCCCC
>hsa-mir-616 MI0003629
UUAGGUAAUUCCUCCACUCAAAACCCUUCAGUGACUUCCAUGACAUGAAAUAGGAAGUCAUUGGA
GGGUUUGAGCAGAGGAAUGACCUGUUUUAAAA
>hsa-mir-617 MI0003631
CAUCAUAAGGAGCCUAGACUUCCCAUUUGAAGGUGGCCAUUUCCUACCACCUUCAAAUGGUAAGU
CCAGGCUCCUUCUGAUUCAAUAAAUGAGGAGC
>hsa-mir-618 MI0003632
CUCUUGUUCACAGCCAAACUCUACUUGUCCUUCUGAGUGUAAUUACGUACAUGCAGUAGCUCAGG
AGACAAGCAGGUUUACCCUGUGGAUGAGUCUGA
>hsa-mir-619 MI0003633
CGCCCACCUCAGCCUCCCAAAAUGCUGGGAUUACAGGCAUGAGCCACUGCGGUCGACCAUGACCU
GGACAUGUUUGUGCCCAGUACUGUCAGUUUGCAG
>hsa-mir-620 MI0003634
AUAUAUAUCUAUAUCUAGCUCCGUAUAUAUAUAUAUAUAUAUAUAGAUAUCUCCAUAUAUAUGGA
GAUAGAUAUAGAAAUAAAACAAGCAAAGAA
>hsa-mir-621 MI0003635
UAGAUUGAGGAAGGGGCUGAGUGGUAGGCGGUGCUGCUGUGCUCUGAUGAAGACCCAUGUGGCUA
GCAACAGCGCUUACCUUUUGUCUCUGGGUCC
>hsa-mir-622 MI0003636
AGAGAAGCUGGACAAGUACUGGUCUCAGCAGAUUGAGGAGAGCACCACAGUGGUCAUCACACAGU
CUGCUGAGGUUGGAGCUGCUGAGAUGACACU
>hsa-mir-623 MI0003637
GUACACAGUAGAAGCAUCCCUUGCAGGGGCUGUUGGGUUGCAUCCUAAGCUGUGCUGGAGCUUCC
CGAUGUACUCUGUAGAUGUCUUUGCACCUUCUG
>hsa-mir-624 MI0003638
AAUGCUGUUUCAAGGUAGUACCAGUACCUUGUGUUCAGUGGAACCAAGGUAAACACAAGGUAUUG
GUAUUACCUUGAGAUAGCAUUACACCUAAGUG

>hsa-mir-625 MI0003639
AGGGUAGAGGGAUGAGGGGGAAAGUUCUAUAGUCCUGUAAUUAGAUCUCAGGACUAUAGAACUUU
CCCCCUCAUCCCUCUGCCCU
>hsa-mir-626 MI0003640
ACUGAUAUAUUUGUCUUAUUUGAGAGCUGAGGAGUAUUUUUAUGCAAUCUGAAUGAUCUCAGCUG
UCUGAAAAUGUCUUCAAUUUUAAAGGCUU
>hsa-mir-627 MI0003641
UACUUAUUACUGGUAGUGAGUCUCUAAGAAAAGAGGAGGUGGUUGUUUUCCUCCUCUUUUCUUUG
AGACUCACUACCAAUAAUAAGAAAUACUACUA
>hsa-mir-628 MI0003642
AUAGCUGUUGUGUCACUUCCUCAUGCUGACAUAUUUACUAGAGGGUAAAAUUAAUAACCUUCUAG
UAAGAGUGGCAGUCGAAGGGAAGGGCUCAU
>hsa-mir-629 MI0003643
UCCCUUUCCCAGGGGAGGGGCUGGGUUUACGUUGGGAGAACUUUUACGGUGAACCAGGAGGUUCU
CCCAACGUAAGCCCAGCCCCUCCCCUCUGCCU
>hsa-mir-630 MI0003644
AACUUAACAUCAUGCUACCUCUUUGUAUCAUAUUUUGUUAUUCUGGUCACAGAAUGACCUAGUAU
UCUGUACCAGGGAAGGUAGUUCUUAACUAUAU
>hsa-mir-631 MI0003645
GUGGGGAGCCUGGUUAGACCUGGCCCAGACCUCAGCUACACAAGCUGAUGGACUGAGUCAGGGGC
CACACUCUCC
>hsa-mir-632 MI0003647
CGCCUCCUACCGCAGUGCUUGACGGGAGGCGGAGCGGGGAACGAGGCCGUCGGCCAUUUUGUGUC
UGCUUCCUGUGGGACGUGGUGGUAGCCGU
>hsa-mir-633 MI0003648
AACCUCUCUUAGCCUCUGUUUCUUUAUUGCGGUAGAUACUAUUAACCUAAAAUGAGAAGGCUAAU
AGUAUCUACCACAAUAAAAUUGUUGUGAGGAUA
>hsa-mir-634 MI0003649
AAACCCACACCACUGCAUUUUGGCCAUCGAGGGUUGGGGCUUGGUGUCAUGCCCCAAGAUAACCA
GCACCCCAACUUUGGACAGCAUGGAUUAGUCU
>hsa-mir-635 MI0003650
CAGAGAGGAGCUGCCACUUGGGCACUGAAACAAUGUCCAUUAGGCUUUGUUAUGGAAACUUCUCC
UGAUCAUUGUUUUGUGUCCAUUGAGCUUCCAAU
>hsa-mir-636 MI0003651
UGGCGGCCUGGGCGGGAGCGCGCGGGCGGGGCCGGCCCCGCUGCCUGGAAUUAACCCCGCUGUGC
UUGCUCGUCCCGCCCGCAGCCCUAGGCGGCGUCG
>hsa-mir-637 MI0003652
UGGCUAAGGUGUUGGCUCGGGCUCCCCACUGCAGUUACCCUCCCCUCGGCGUUACUGAGCACUGG
GGGCUUUCGGGCUCUGCGUCUGCACAGAUACUUC
>hsa-mir-638 MI0003653
GUGAGCGGGCGCGGCAGGGAUCGCGGGCGGGUGGCGGCCUAGGGCGCGGAGGGCGGACCGGGAAU
GGCGCGCCGUGCGCCGCCGGCGUAACUGCGGCGCU
>hsa-mir-639 MI0003654
UGGCCGACGGGGCGCGCGCGGCCUGGAGGGGCGGGGCGGACGCAGAGCCGCGUUUAGUCUAUCGC

UGCGGUUGCGAGCGCUGUAGGGAGCCUGUGCUG
>hsa-mir-640 MI0003655
GUGACCCUGGGCAAGUUCCUGAAGAUCAGACACAUCAGAUCCCUUAUCUGUAAAAUGGGCAUGAU
CCAGGAACCUGCCUCUACGGUUGCCUUGGGG
>hsa-mir-641 MI0003656
UGGGUGAAAGGAAGGAAAGACAUAGGAUAGAGUCACCUCUGUCCUCUGUCCUCUACCUAUAGAGG
UGACUGUCCUAUGUCUUUCCUUCCUCUUACCCCU
>hsa-mir-642a MI0003657
AUCUGAGUUGGGAGGGUCCCUCUCCAAAUGUGUCUUGGGGUGGGGGAUCAAGACACAUUUGGAGA
GGGAACCUCCCAACUCGGCCUCUGCCAUCAUU
>hsa-mir-643 MI0003658
ACCAAGUGAUAUUCAUUGUCUACCUGAGCUAGAAUACAAGUAGUUGGCGUCUUCAGAGACACUUG
UAUGCUAGCUCAGGUAGAUAUUGAAUGAAAAA
>hsa-mir-644a MI0003659
UUUUUUUUUAGUAUUUUUCCAUCAGUGUUCAUAAGGAAUGUUGCUCUGUAGUUUUCUUAUAGUGU
GGCUUUCUUAGAGCAAAGAUGGUUCCCUA
>hsa-mir-645 MI0003660
CAGUUCCUAACAGGCCUCAGACCAGUACCGGUCUGUGGCCUGGGGGUUGAGGACCCCUGCUCUAG
GCUGGUACUGCUGAUGCUUAAAAAGAGAG
>hsa-mir-646 MI0003661
GAUCAGGAGUCUGCCAGUGGAGUCAGCACACCUGCUUUUCACCUGUGAUCCCAGGAGAGGAAGCA
GCUGCCUCUGAGGCCUCAGGCUCAGUGGC
>hsa-mir-647 MI0003662
AGGAAGUGUUGGCCUGUGGCUGCACUCACUUCCUUCAGCCCCAGGAAGCCUUGGUCGGGGGCAGG
AGGGAGGGUCAGGCAGGGCUGGGGGCCUGAC
>hsa-mir-648 MI0003663
AUCACAGACACCUCCAAGUGUGCAGGGCACUGGUGGGGGCCGGGGCAGGCCCAGCGAAAGUGCAG
GACCUGGCACUUAGUCGGAAGUGAGGGUG
>hsa-mir-649 MI0003664
GGCCUAGCCAAAUACUGUAUUUUUGAUCGACAUUUGGUUGAAAAAUAUCUAUGUAUUAGUAAACC
UGUGUUGUUCAAGAGUCCACUGUGUUUUGCUG
>hsa-mir-650 MI0003665
CAGUGCUGGGGUCUCAGGAGGCAGCGCUCUCAGGACGUCACCACCAUGGCCUGGGCUCUGCUCCU
CCUCACCCUCCUCACUCAGGGCACAGGUGAU
>hsa-mir-651 MI0003666
AAUCUAUCACUGCUUUUUAGGAUAAGCUUGACUUUUGUUCAAAUAAAAAUGCAAAAGGAAAGUGU
AUCCUAAAAGGCAAUGACAGUUUAAUGUGUUU
>hsa-mir-652 MI0003667
ACGAAUGGCUAUGCACUGCACAACCCUAGGAGAGGGUGCCAUUCACAUAGACUAUAAUUGAAUGG
CGCCACUAGGGUUGUGCAGUGCACAACCUACAC
>hsa-mir-653 MI0003674
UUCAUUCCUUCAGUGUUGAAACAAUCUCUACUGAACCAGCUUCAAACAAGUUCACUGGAGUUUGU
UUCAAUAUUGCAAGAAUGAUAAGAUGGAAGC
>hsa-mir-654 MI0003676

GGGUAAGUGGAAAGAUGGUGGGCCGCAGAACAUGUGCUGAGUUCGUGCCAUAUGUCUGCUGACCA
UCACCUUUAGAAGCCC
>hsa-mir-655 MI0003677
AACUAUGCAAGGAUAUUUGAGGAGAGGUUAUCCGUGUUAUGUUCGCUUCAUUCAUCAUGAAUAAU
ACAUGGUUAACCUCUUUUUGAAUAUCAGACUC
>hsa-mir-656 MI0003678
CUGAAAUAGGUUGCCUGUGAGGUGUUCACUUUCUAUAUGAUGAAUAUUAUACAGUCAACCUCUUU
CCGAUAUCGAAUC
>hsa-mir-657 MI0003681
GUGUAGUAGAGCUAGGAGGAGAGGGUCCUGGAGAAGCGUGGACCGGUCCGGGUGGGUUCCGGCAG
GUUCUCACCCUCUCUAGGCCCCAUUCUCCUCUG
>hsa-mir-658 MI0003682
GCUCGGUUGCCGUGGUUGCGGGCCCUGCCCGCCCGCCAGCUCGCUGACAGCACGACUCAGGGCGG
AGGGAAGUAGGUCCGUUGGUCGGUCGGGAACGAGG
>hsa-mir-659 MI0003683
UACCGACCCUCGAUUUGGUUCAGGACCUUCCCUGAACCAAGGAAGAGUCACAGUCUCUUCCUUGG
UUCAGGGAGGGUCCCCAACAAUGUCCUCAUGG
>hsa-mir-660 MI0003684
CUGCUCCUUCUCCCAUACCCAUUGCAUAUCGGAGUUGUGAAUUCUCAAAACACCUCCUGUGUGCA
UGGAUUACAGGAGGGUGAGCCUUGUCAUCGUG
>hsa-mir-661 MI0003669
GGAGAGGCUGUGCUGUGGGGCAGGCGCAGGCCUGAGCCCUGGUUUCGGGCUGCCUGGGUCUCUGG
CCUGCGCGUGACUUUGGGGUGGCU
>hsa-mir-662 MI0003670
GCUGUUGAGGCUGCGCAGCCAGGCCCUGACGGUGGGGUGGCUGCGGGCCUUCUGAAGGUCUCCCA
CGUUGUGGCCCAGCAGCGCAGUCACGUUGC
>hsa-mir-663b MI0006336
GGUGCCGAGGGCCGUCCGGCAUCCUAGGCGGGUCGCUGCGGUACCUCCCUCCUGUCUGUGGCGGU
GGGAUCCCGUGGCCGUGUUUUCCUGGUGGCCCGGCCGUGCCUGAGGUUUC
>hsa-mir-664a MI0006442
GAACAUUGAAACUGGCUAGGGAAAAUGAUUGGAUAGAAACUAUUAUUCUAUUCAUUUAUCCCCAG
CCUACAAAAUGAAAAAA
>hsa-mir-665 MI0005563
UCUCCUCGAGGGGUCUCUGCCUCUACCCAGGACUCUUUCAUGACCAGGAGGCUGAGGCCCCUCAC
AGGCGGC
>hsa-mir-668 MI0003761
GGUAAGUGCGCCUCGGGUGAGCAUGCACUUAAUGUGGGUGUAUGUCACUCGGCUCGGCCCACUAC
C
>hsa-mir-670 MI0003933
GUUUAGGGGUGGACCUGAUGUCCCUGAGUGUAUGUGGUGAACCUGAAUUUGCCUUGGGUUUCCUC
AUAUUCAUUCAGGAGUGUCAGUUGCCCCUUCAC
>hsa-mir-671 MI0003760
GCAGGUGAACUGGCAGGCCAGGAAGAGGAGGAAGCCCUGGAGGGGCUGGAGGUGAUGGAUGUUUU
CCUCCGGUUCUCAGGGCUCCACCUCUUUCGGGCCGUAGAGCCAGGGCUGGUGC

>hsa-mir-675 MI0005416
CCCAGGGUCUGGUGCGGAGAGGGCCCACAGUGGACUUGGUGACGCUGUAUGCCCUCACCGCUCAG
CCCCUGGG
>hsa-mir-676 MI0016436
GCAUGACUCUUCAACCUCAGGACUUGCAGAAUUAAUGGAAUGCUGUCCUAAGGUUGUUGAGUUGU
GC
>hsa-mir-708 MI0005543
AACUGCCCUCAAGGAGCUUACAAUCUAGCUGGGGGUAAAUGACUUGCACAUGAACACAACUAGAC
UGUGAGCUUCUAGAGGGCAGGGA
>hsa-mir-711 MI0012488
ACUGACUUUGAGUCUCUCCUCAGGGUGCUGCAGGCAAAGCUGGGGACCCAGGGAGAGACGUAAGU
GAGGGGAGAUG
>hsa-mir-718 MI0012489
GGCCGCGGCGCGCAAGAUGGCGGCGGGCCCGGGCACCGCCCCUUCCGCCCCGCCGGGCGUCGCAC
GAGGC
>hsa-mir-744 MI0005559
UUGGGCAAGGUGCGGGGCUAGGGCUAACAGCAGUCUUACUGAAGGUUUCCUGGAAACCACGCACA
UGCUGUUGCCACUAACCUCAACCUUACUCGGUC
>hsa-mir-758 MI0003757
GCCUGGAUACAUGAGAUGGUUGACCAGAGAGCACACGCUUUAUUUGUGCCGUUUGUGACCUGGUC
CACUAACCCUCAGUAUCUAAUGC
>hsa-mir-759 MI0004065
UAAUAAAUUAAAUGCCUAAACUGGCAGAGUGCAAACAAUUUUGACUCAGAUCUAAAUGUUUGCAC
UGGCUGUUUAAACAUUUAAUUUGUUA
>hsa-mir-760 MI0005567
GGCGCGUCGCCCCCCUCAGUCCACCAGAGCCCGGAUACCUCAGAAAUUCGGCUCUGGGUCUGUGG
GGAGCGAAAUGCAAC
>hsa-mir-761 MI0003941
GGAGGAGCAGCAGGGUGAAACUGACACAGUUCUGGUGAGUUUCACUUUGCUGCUCCUCC
>hsa-mir-762 MI0003892
GGCCCGGCUCCGGGUCUCGGCCCGUACAGUCCGGCCGGCCAUGCUGGCGGGGCUGGGGCCGGGGC
CGAGCCCGCGGCGGGGCC
>hsa-mir-764 MI0003944
AAUCUAGGAGGCAGGUGCUCACUUGUCCUCCUCCAUGCUUGGAAAAUGCAGGGAGGAGGCCAUAG
UGGCAACUGUUACCAUGAUU
>hsa-mir-765 MI0005116
UUUAGGCGCUGAUGAAAGUGGAGUUCAGUAGACAGCCCUUUUCAAGCCCUACGAGAAACUGGGGU
UUCUGGAGGAGAAGGAAGGUGAUGAAGGAUCUGUUCUCGUGAGCCUGAA
>hsa-mir-766 MI0003836
GCAUCCUCAGGACCUGGGCUUGGGUGGUAGGAGGAAUUGGUGCUGGUCUUUCAUUUUGGAUUUGA
CUCCAGCCCCACAGCCUCAGCCACCCCAGCCAAUUGUCAUAGGAGC
>hsa-mir-767 MI0003763
GCUUUUAUAUUGUAGGUUUUUGCUCAUGCACCAUGGUUGUCUGAGCAUGCAGCAUGCUUGUCUGC
UCAUACCCCAUGGUUUCUGAGCAGGAACCUUCAUUGUCUACUGC

>hsa-mir-769 MI0003834
GCCUUGGUGCUGAUUCCUGGGCUCUGACCUGAGACCUCUGGGUUCUGAGCUGUGAUGUUGCUCUC
GAGCUGGGAUCUCCGGGGUCUUGGUUCAGGGCCGGGGCCUCUGGGUUCCAAGC
>hsa-mir-770 MI0005118
AGGAGCCACCUUCCGAGCCUCCAGUACCACGUGUCAGGGCCACAUGAGCUGGGCCUCGUGGGCCU
GAUGUGGUGCUGGGGCCUCAGGGGUCUGCUCUU
>hsa-mir-802 MI0003906
GUUCUGUUAUUUGCAGUCAGUAACAAAGAUUCAUCCUUGUGUCCAUCAUGCAACAAGGAGAAUCU
UUGUCACUUAGUGUAAUUAAUAGCUGGAC
>hsa-mir-873 MI0005564
GUGUGCAUUUGCAGGAACUUGUGAGUCUCCUAUUGAAAAUGAACAGGAGACUGAUGAGUUCCCGG
GAACACCCACAA
>hsa-mir-874 MI0005532
UUAGCCCUGCGGCCCCACGCACCAGGGUAAGAGAGACUCUCGCUUCCUGCCCUGGCCCGAGGGAC
CGACUGGCUGGGC
>hsa-mir-875 MI0005541
UUAGUGGUACUAUACCUCAGUUUUAUCAGGUGUUCUUAAAAUCACCUGGAAACACUGAGGUUGUG
UCUCACUGAAC
>hsa-mir-876 MI0005542
UGAAGUGCUGUGGAUUUCUUUGUGAAUCACCAUAUCUAAGCUAAUGUGGUGGUGGUUUACAAAGU
AAUUCAUAGUGCUUCA
>hsa-mir-877 MI0005561
GUAGAGGAGAUGGCGCAGGGGACACGGGCAAAGACUUGGGGGUUCCUGGGACCCUCAGACGUGUG
UCCUCUUCUCCCUCCUCCCAG
>hsa-mir-885 MI0005560
CCGCACUCUCUCCAUUACACUACCCUGCCUCUUCUCCAUGAGAGGCAGCGGGGUGUAGUGGAUAG
AGCACGGGU
>hsa-mir-887 MI0005562
GUGCAGAUCCUUGGGAGCCCUGUUAGACUCUGGAUUUUACACUUGGAGUGAACGGGCGCCAUCCC
GAGGCUUUGCACAG
>hsa-mir-888 MI0005537
GGCAGUGCUCUACUCAAAAAGCUGUCAGUCACUUAGAUUACAUGUGACUGACACCUCUUUGGGUG
AAGGAAGGCUCA
>hsa-mir-889 MI0005540
GUGCUUAAAGAAUGGCUGUCCGUAGUAUGGUCUCUAUAUUUAUGAUGAUUAAUAUCGGACAACCA
UUGUUUUAGUAUCC
>hsa-mir-890 MI0005533
GGAAGUGCCCUACUUGGAAAGGCAUCAGUUGCUUAGAUUACAUGUAACUAUUCCCUUUCUGAGUA
GAGUAAGUCUUA
>hsa-mir-891a MI0005524
CCUUAAUCCUUGCAACGAACCUGAGCCACUGAUUCAGUAAAAUACUCAGUGGCACAUGUUUGUUG
UGAGGGUCAAAAGA
>hsa-mir-892b MI0005538
UGCAAUGCCCUACUCAGAAAGGUGCCAUUUAUGUAGAUUUUAUGUCACUGGCUCCUUUCUGGGUA

GAGCAAGGCUCA
>hsa-mir-920 MI0005712
GUAGUUGUUCUACAGAAGACCUGGAUGUGUAGGAGCUAAGACACACUCCAGGGGAGCUGUGGAAG
CAGUAACACG
>hsa-mir-921 MI0005713
ACUAGUGAGGGACAGAACCAGGAUUCAGACUCAGGUCCAUGGGCCUGGAUCACUGG
>hsa-mir-922 MI0005714
AUGGCGUUUUCCCUCUCCCUGUCCUGGACUGGGGUCAGACUGUGCCCCGAGGAGAAGCAGCAGAG
AAUAGGACUACGUCAU
>hsa-mir-924 MI0005716
AAUAGAGUCUUGUGAUGUCUUGCUUAAGGGCCAUCCAACCUAGAGUCUACAAC
>hsa-mir-933 MI0005755
ACUUGGGUCAGUUCAGAGGUCCUCGGGGCGCGCGUCGAGUCAGCCGUGUGCGCAGGGAGACCUCU
CCCACCCACAGU
>hsa-mir-934 MI0005756
AGAAAUAAGGCUUCUGUCUACUACUGGAGACACUGGUAGUAUAAAACCCAGAGUCUCCAGUAAUG
GACGGGAGCCUUAUUUCU
>hsa-mir-935 MI0005757
GGCGGGGGCGCGGGCGGCAGUGGCGGGAGCGGCCCCUCGGCCAUCCUCCGUCUGCCCAGUUACCG
CUUCCGCUACCGCCGCCGCUCCCGCU
>hsa-mir-936 MI0005758
UCAAGGCCACUGGGACAGUAGAGGGAGGAAUCGCAGAAAUCACUCCAGGAGCAACUGAGAGACCU
UGCUUCUACUUUACCAGGUCCUGCUGGCCCAGA
>hsa-mir-937 MI0005759
AGCACUGCCCCCGGUGAGUCAGGGUGGGGCUGGCCCCCUGCUUCGUGCCCAUCCGCGCUCUGACU
CUCUGCCCACCUGCAGGAGCU
>hsa-mir-938 MI0005760
GAAGGUGUACCAUGUGCCCUUAAAGGUGAACCCAGUGCACCUUCAUGAACCGUGGUACACCUUUA
AGAACUUGGUAUGCCUUC
>hsa-mir-939 MI0005761
UGUGGGCAGGGCCCUGGGGAGCUGAGGCUCUGGGGGUGGCCGGGGCUGACCCUGGGCCUCUGCUC
CCCAGUGUCUGACCGCG
>hsa-mir-940 MI0005762
GUGAGGUGUGGGCCCGGCCCCAGGAGCGGGGCCUGGGCAGCCCCGUGUGUUGAGGAAGGAAGGCA
GGGCCCCCGCUCCCCGGGCCUGACCCCAC
>hsa-mir-941-1 MI0005763
UGUGGACAUGUGCCCAGGGCCCGGGACAGCGCCACGGAAGAGGACGCACCCGGCUGUGUGCACAU
GUGCCCA
>hsa-mir-942 MI0005767
AUUAGGAGAGUAUCUUCUCUGUUUUGGCCAUGUGUGUACUCACAGCCCCUCACACAUGGCCGAAA
CAGAGAAGUUACUUUCCUAAU
>hsa-mir-943 MI0005768
GGGACGUUCUGAGCUCGGGGUGGGGGACGUUUGCCGGUCACUGCUGCUGGCGCCCUGACUGUUGC
CGUCCUCCAGCCCCACUCAAAGGCAUCCC

>hsa-mir-944 MI0005769
GUUCCAGACACAUCUCAUCUGAUAUACAAUAUUUUCUUAAAUUGUAUAAAGAGAAAUUAUUGUAC
AUCGGAUGAGCUGUGUCUGGGAU
>hsa-mir-1178 MI0006271
GCGUUGGCUGGCAGAGGAAGGGAAGGGUCCAGGGUCAGCUGAGCAUGCCCUCAGGUUGCUCACUG
UUCUUCCCUAGAAUGUCAGGUGAUGU
>hsa-mir-1179 MI0006272
GGCUGGAAAGGAAGAAGCAUUCUUUCAUUGGUUGGUGUGUAUUGCCUUGUCAACCAAUAAGAGGA
UGCCAUUUAUCCUUUUCUGACUAGCU
>hsa-mir-1180 MI0006273
GCUGCUGGACCCACCCGGCCGGGAAUAGUGCUCCUGGUUGUUUCCGGCUCGCGUGGGUGUGUCGG
CGGC
>hsa-mir-1181 MI0006274
UCCACUGCUGCCGCCGUCGCCGCCACCCGAGCCGGAGCGGGCUGGGCCGCCAAGGCAAGAUGGUG
GACUACAGCGUGUGGG
>hsa-mir-1182 MI0006275
GGGACUUGUCACUGCCUGUCUCCUCCCUCUCCAGCAGCGACUGGAUUCUGGAGUCCAUCUAGAGG
GUCUUGGGAGGGAUGUGACUGUUGGGAAGCCC
>hsa-mir-1183 MI0006276
AUUAUUCAAAUGCUCGGAGACACAGAACAUUAGAGAAGACAGGAGUUCACUGUAGGUGAUGGUGA
GAGUGGGCAUGGAGCAGGAGUGCC
>hsa-mir-1184-1 MI0006277
CUUGCAGAACGAGGUGAAGGAGGUGGUUCUGCUCAGCAGUCAACAGUGGCCACAUCUCCACCUGC
AGCGACUUGAUGGCUUCCGUGUCCUUUUCGUGGG
>hsa-mir-1185-1 MI0003844
UUUGGUACUUGAAGAGAGGAUACCCUUUGUAUGUUCACUUGAUUAAUGGCGAAUAUACAGGGGGA
GACUCUUAUUUGCGUAUCAAA
>hsa-mir-1193 MI0014205
GUAGCUGAGGGGAUGGUAGACCGGUGACGUGCACUUCAUUUACGAUGUAGGUCACCCGUUUGACU
AUCCACCAGCGCC
>hsa-mir-1197 MI0006656
ACUUCCUGGUAUUUGAAGAUGCGGUUGACCAUGGUGUGUACGCUUUAUUUGUGACGUAGGACACA
UGGUCUACUUCUUCUCAAUAUCA
>hsa-mir-1199 MI0020340
AGCCUGCGCCGGAGCCGGGGCCUGAGCCCGGGCCGCGCAGGCCGUGAACUCGUCGAGCUGCGCGU
GCGGCCGGUGCUCAACCUGCCGGGUCCUGGCCCCGCGCUCCCGCGCGCCCUGGA
>hsa-mir-1200 MI0006332
UGCUACUUCUCCUGAGCCAUUCUGAGCCUCAAUCACUUGCCAGAGAGAUUGGUUCAGGAAUUUGU
CAGGGAUAGCC
>hsa-mir-1202 MI0006334
CCUGCUGCAGAGGUGCCAGCUGCAGUGGGGGAGGCACUGCCAGGGCUGCCCACUCUGCUUAGCCA
GCAGGUGCCAAGAACAGG
>hsa-mir-1203 MI0006335
UCCUCCCCGGAGCCAGGAUGCAGCUCAAGCCACAGCAGGGUGUUUAGCGCUCUUCAGUGGCUCCA

GAUUGUGGCGCUGGUGCAGG
>hsa-mir-1204 MI0006337
ACCUCGUGGCCUGGUCUCCAUUAUUUGAGAUGAGUUACAUCUUGGAGGUGAGGACGUGCCUCGUG
GU
>hsa-mir-1205 MI0006338
GAAGGCCUCUGCAGGGUUUGCUUUGAGGUACUUCCUUCCUGUCAACCCUGUUCUGGAGUCUGU
>hsa-mir-1206 MI0006339
CAGUGUUCAUGUAGAUGUUUAAGCUCUUGCAGUAGGUUUUUGCAAGCUAGUGAACGCUG
>hsa-mir-1207 MI0006340
GCAGGGCUGGCAGGGAGGCUGGGAGGGGCUGGCUGGGUCUGGUAGUGGGCAUCAGCUGGCCCUCA
UUUCUUAAGACAGCACUUCUGU
>hsa-mir-1208 MI0006341
CACCGGCAGAAUCACUGUUCAGACAGGCGGAGACGGGUCUUUCUCGCCCUCUGAUGAGUCACCAC
UGUGGUGG
>hsa-mir-1224 MI0003764
GUGAGGACUCGGGAGGUGGAGGGUGGUGCCGCCGGGGCCGGGCGCUGUUUCAGCUCGCUUCUCCC
CCCACCUCCUCUCUCCUCAG
>hsa-mir-1225 MI0006311
GUGGGUACGGCCCAGUGGGGGGGAGAGGGACACGCCCUGGGCUCUGCCCAGGGUGCAGCCGGACU
GACUGAGCCCCUGUGCCGCCCCCAG
>hsa-mir-1226 MI0006313
GUGAGGGCAUGCAGGCCUGGAUGGGGCAGCUGGGAUGGUCCAAAAGGGUGGCCUCACCAGCCCUG
UGUUCCCUAG
>hsa-mir-1227 MI0006316
GUGGGGCCAGGCGGUGGUGGGCACUGCUGGGGUGGGCACAGCAGCCAUGCAGAGCGGGCAUUUGA
CCCCGUGCCACCCUUUUCCCCAG
>hsa-mir-1228 MI0006318
GUGGGCGGGGGCAGGUGUGUGGUGGGUGGUGGCCUGCGGUGAGCAGGGCCCUCACACCUGCCUCG
CCCCCCAG
>hsa-mir-1229 MI0006319
GUGGGUAGGGUUUGGGGGAGAGCGUGGGCUGGGGUUCAGGGACACCCUCUCACCACUGCCCUCCC
ACAG
>hsa-mir-1231 MI0006321
GUCAGUGUCUGGGCGGACAGCUGCAGGAAAGGGAAGACCAAGGCUUGCUGUCUGUCCAGUCUGCC
ACCCUACCCUGUCUGUUCUUGCCACAG
>hsa-mir-1233-1 MI0006323
GUGAGUGGGAGGCCAGGGCACGGCAGGGGGAGCUGCAGGGCUAUGGGAGGGGCCCCAGCGUCUGA
GCCCUGUCCUCCCGCAG
>hsa-mir-1234 MI0006324
GUGAGUGUGGGGUGGCUGGGGGGGGGGGGGGGGGCCGGGGACGGCUUGGGCCUGCCUAGUCGGC
CUGACCACCCACCCCACAG
>hsa-mir-1236 MI0006326
GUGAGUGACAGGGGAAAUGGGGAUGGACUGGAAGUGGGCAGCAUGGAGCUGACCUUCAUCAUGGC
UUGGCCAACAUAAUGCCUCUUCCCCUUGUCUCUCCAG

>hsa-mir-1237 MI0006327
GUGGGAGGGCCCAGGCGCGGGCAGGGGUGGGGGUGGCAGAGCGCUGUCCCGGGGGCGGGGCCGAA
GCGCGGCGACCGUAACUCCUUCUGCUCCGUCCCCCAG
>hsa-mir-1238 MI0006328
GUGAGUGGGAGCCCCAGUGUGUGGUUGGGGCCAUGGCGGGUGGGCAGCCCAGCCUCUGAGCCUUC
CUCGUCUGUCUGCCCCAG
>hsa-mir-1243 MI0006373
CUAAAACUGGAUCAAUUAUAGGAGUGAAAUAAAGGUCCAUCUCCUGCCUAUUUAUUACUUUGCUU
UGGUAAUAAAUCUAUUUUUAAAAGAACC
>hsa-mir-1244-1 MI0006379
AUCUUAUUCCGAGCAUUCCAGUAACUUUUUUGUGUAUGUACUUAGCUGUACUAUAAGUAGUUGGU
UUGUAUGAGAUGGUUAAAAA
>hsa-mir-1245a MI0006380
AUUUAUGUAUAGGCCUUUAGAUCAUCUGAUGUUGAAUACUCUUUAAGUGAUCUAAAGGCCUACAU
AUAAA
>hsa-mir-1245b MI0017431
UUUAUAUGUAGGCCUUUAGAUCACUUAAAGAGUAUUCAACAUCAGAUGAUCUAAAGGCCUAUACA
UAAA
>hsa-mir-1246 MI0006381
UGUAUCCUUGAAUGGAUUUUUGGAGCAGGAGUGGACACCUGACCCAAAGGAAAUCAAUCCAUAGG
CUAGCAAU
>hsa-mir-1247 MI0006382
CCGCUUGCCUCGCCCAGCGCAGCCCCGGCCGCUGGGCGCACCCGUCCCGUUCGUCCCCGGACGUU
GCUCUCUACCCCGGGAACGUCGAGACUGGAGCGCCCGAACUGAGCCACCUUCGCGGACCCCGAGA
GCGGCG
>hsa-mir-1248 MI0006383
UUUACCUUCUUGUAUAAGCACUGUGCUAAAAUUGCAGACACUAGGACCAUGUCUUGGUUUUUGCA
AUAAUGCUAGCAGAGUACACACAAGAAGAAAAGUAACAGCA
>hsa-mir-1249 MI0006384
GGGAGGAGGGAGGAGAUGGGCCAAGUUCCCUCUGGCUGGAACGCCCUUCCCCCCCUUCUUCACCU
G
>hsa-mir-1250 MI0006385
CUGUCCCGCUGGCCUGGCAGGUGACGGUGCUGGAUGUGGCCUUUUUGCCUUUUCUAAAGGCCACA
UUUUCCAGCCCAUUCAACCUUCCAGAGCCCUCUGAAGUGGCCACAGGC
>hsa-mir-1251 MI0006386
GUGGACUCUAGCUGCCAAAGGCGCUUCUCCUUCUGAACAGAGCGCUUUGCUCAGCCAGUGUAGAC
AUGGC
>hsa-mir-1252 MI0006434
AGAAAGAAGGAAAUUGAAUUCAUUUAGAAAAGAGAAUUCCAAAUGAGCUUAAUUUCCUUUUUUCU
>hsa-mir-1253 MI0006387
AGCAGCAAGAGAUAGAAUCCAAAAGAGAAGAAGAUCAGCCUGCAGAUGUGGACUGCUAAAUGCAG
GCUGAUCUUCUCCCCUUUGGGAUUCUCUUAUGAGAAGCCA
>hsa-mir-1254-1 MI0006388
GGUGGGAGGAUUGCUUGAGCCUGGAAGCUGGAGCCUGCAGUGAACUAUCAUUGUGCCACUGUACU

CCAGCCUAGGCAACAAAAUGAAAUCCUGUCUA
>hsa-mir-1255a MI0006389
AUUGGAAAUCCUUUGAGUUGCUUCUCAAGGAUGAGCAAAGAAAGUAGAUUUUUUAGAUUCUAAAG
AAACUAUCUUCUUUGCUCAUCCUUGAGAAGCAACUCCUUAUCCAUUAA
>hsa-mir-1255b-1 MI0006435
UACGGAUGAGCAAAGAAAGUGGUUUCUUAAAAUGGAAUCUACUCUUUGUGAAGAUGCUGUGAA
>hsa-mir-1255b-2 MI0006436
UCUUACGGAUGAGCAAAGAAAGUGGUUUGCGCCUCAAGAAACCACUUUCUUUGCUCAUCCAUAAG
GA
>hsa-mir-1256 MI0006390
AGUCAGCCUGUUGAAGCUUUGAAGCUUUGAUGCCAGGCAUUGACUUCUCACUAGCUGUGAAAGUC
CUAGCUAAAGAGAAGUCAAUGCAUGACAUCUUGUUUCAAUAGAUGGCUGUUUCA
>hsa-mir-1257 MI0006391
GCCCUGGGCUUGUGCUUGGGGAGUGAAUGAUGGGUUCUGACCCCCAUGCACCCCUGUGGGCCCCU
GGCAUCACUGGCCCCAUCCUUCACCCCUGCCAACCACGCUUGCCCUGUGCCU
>hsa-mir-1258 MI0006392
CUGUGGCUUCCACGACCUAAUCCUAACUCCUGCGAGUCCCUGGAGUUAGGAUUAGGUCGUGGAAG
CCACAGGA
>hsa-mir-1260a MI0006394
ACCUUUCCAGCUCAUCCCACCUCUGCCACCAAAACACUCAUCGCGGGGUCAGAGGGAGUGCCAAA
AAAGGUAA
>hsa-mir-1260b MI0014197
UCUCCGUUUAUCCCACCACUGCCACCAUUAUUGCUACUGUUCAGCAGGUGCUGCUGGUGGUGAUG
GUGAUAGUCUGGUGGGGGCGGUGG
>hsa-mir-1261 MI0006396
UGCUAUGGAUAAGGCUUUGGCUUAUGGGGAUAUUGUGGUUGAUCUGUUCUAUCCAGAUGACUGAA
ACUUUCUCCAUAGCAGC
>hsa-mir-1262 MI0006397
AUCUACAAUGGUGAUGGGUGAAUUUGUAGAAGGAUGAAAGUCAAAGAAUCCUUCUGGGAACUAAU
UUUUGGCCUUCAACAAGAAUUGUGAUAU
>hsa-mir-1263 MI0006398
CUACCCCAAAAUAUGGUACCCUGGCAUACUGAGUAUUUUAAUACUGGCAUACUCAGUAUGCCAUG
UUGCCAUAUUUUGGGGUAGCA
>hsa-mir-1264 MI0003758
AGGUCCUCAAUAAGUAUUUGUUGAAAGAAUAAAUAAACCAACAAGUCUUAUUUGAGCACCUGUUA
UGUG
>hsa-mir-1265 MI0006401
AUGGUUUGGGACUCAGGAUGUGGUCAAGUGUUGUUAAGGCAUGUUCAGGAACAAUACUUGACCAC
AUUUUGAAUUCCAAACCAUAU
>hsa-mir-1266 MI0006403
ACAGGUAGUGUCCCUCAGGGCUGUAGAACAGGGCUGGGAUUACUAAAGCCCUGUUCUAUGCCCUG
AGGGACACUGAGCAUGUCA
>hsa-mir-1267 MI0006404
CUCCCAAAUCUCCUGUUGAAGUGUAAUCCCCACCUCCAGCAUUGGGGAUUACAUUUCAACAUGAG

AUUUGGAUGAGGA
>hsa-mir-1268a MI0006405
UAGCCGGGCGUGGUGGUGGGGGCCUGUGGUCCCAGCUACUUUGGAGGCUGAG
>hsa-mir-1268b MI0016748
ACCCGGGCGUGGUGGUGGGGGUGGGUGCCUGUAAUUCCAGCUAGUUGGGA
>hsa-mir-1269a MI0006406
UGGAUUGCCUAGACCAGGGAAGCCAGUUGGCAUGGCUCAGUCCAAGUCUGACCACCUGAGGAAUG
CCUGGACUGAGCCGUGCUACUGGCUUCCCUGGUCUCCAGC
>hsa-mir-1269b MI0016888
UGAGGUUUCUGGACUGAGCCAUGCUACUGGCUUCUCUGGUUCUCCAGCUUACAGAUGGCUUAUCA
UGGGACCUCU
>hsa-mir-1270-1 MI0006407
CACAGAGUUAUACUGGAGAUAUGGAAGAGCUGUGUUGGGUAUAAGUAACAGGCUUUUCUUUAUCU
UCUAUGUGGCUCUUUGCA
>hsa-mir-1271 MI0003814
CACCCAGAUCAGUGCUUGGCACCUAGCAAGCACUCAGUAAAUAUUUGUUGAGUGCCUGCUAUGUG
CCAGGCAUUGUGCUGAGGGCU
>hsa-mir-1272 MI0006408
CCAGAUCAGAUCUGGGUGCGAUGAUGAUGGCAGCAAAUUCUGAAAACGUGCUCAGUGUCUUUAUA
ACAGGAAAGCCGUAAACUUAGAAAUGUAGGCUGCAGCUCGUGUGCUCUGUGGUCUGGGCUGGUA
>hsa-mir-1273a MI0006409
UGAGGCAGGAGAAUUGCUUGAACCCGGGUGGUGGAGGUUGCAGUGAGCCAAGAUUGCGCCACUGC
ACUCCAGCCUGGGCGACAAAGCAAGACUCUUUCUUGGA
>hsa-mir-1273c MI0014171
UGCAGCCUGGGCGACAAAACGAGACCCUGUCUUUUUUUUUUUCUGAGACAGAGUCUCGUUCUGUU
GCCCAAGCUGGA
>hsa-mir-1273f MI0018002
AGGUGGGAGGAUUGCUUGAGCCUGGGAGAUGGAGGUUGCAGUGAGCUGAGAUCACGCAACUGCAC
CCCCAGCCUGGGCCAUAGAGUCAGUCCUUGUCUC
>hsa-mir-1273g MI0018003
GAGGUGGGAGGAUUGCUUGAGUCAGGGUGGUUGAGGCUGCAGUAAGUUGUGAUCAUACCACUGCA
CUCCAGCCUGAGUGACAGAGCAAGACCUUGUCUCA
>hsa-mir-1273h MI0025512
UACUUGGGUGACUAAGGCAGGAUUGCUUGAGCCUGGGAGGUCAAGGCUGCAGUGUCGUGGUCACA
GCUUGCUGCAGACUCGACCUCCCAGGCUUAAGCAAUCCUCCUGCUCGAGUG
>hsa-mir-1275 MI0006415
CCUCUGUGAGAAAGGGUGUGGGGGAGAGGCUGUCUUGUGUCUGUAAGUAUGCCAAACUUAUUUUC
CCCAAGGCAGAGGGA
>hsa-mir-1276 MI0006416
CCCCAGCUAGGUAAAGAGCCCUGUGGAGACACCUGGAUUCAGAGAACAUGUCUCCACUGAGCACU
UGGGCCUUGAUGGCGGCU
>hsa-mir-1277 MI0006419
ACCUCCCAAAUAUAUAUAUAUAUGUACGUAUGUGUAUAUAAAUGUAUACGUAGAUAUAUAUGUAU
UUUUGGUGGGUUU

>hsa-mir-1278 MI0006425
AUUUGCUCAUAGAUGAUAUGCAUAGUACUCCCAGAACUCAUUAAGUUGGUAGUACUGUGCAUAUC
AUCUAUGAGCGAAUAG
>hsa-mir-1279 MI0006426
AUAUUCACAAAAAUUCAUAUUGCUUCUUUCUAAUGCCAAGAAAGAAGAGUAUAAGAACUUCC
>hsa-mir-1282 MI0006429
CCUUCUUCUCGUUUGCCUUUUUCUGCUUCUGCUGCAUGAUCUCCGAGUCCCUGGGGGUAGAGAUG
AUGGGGCACUGGGAGGUACCAGAGGGCAAAAAGGAC
>hsa-mir-1284 MI0006431
AUUUUGAUAUAUAAGCCAGUUUAAUGUUUUCUAUACAGACCCUGGCUUUUCUUAAAUUUUAUAUA
UUGGAAAGCCCAUGUUUGUAUUGGAAACUGCUGGUUUCUUUCAUACUGAAAAUCU
>hsa-mir-1285-1 MI0006346
UGUAGAGAUAGGAUCUCACUUUGUUGCCCAGGCUGGUCUCAAACUCCUGGUCUGGGCAACAAAGU
GAGACCUUAUCUCUACAAG
>hsa-mir-1285-2 MI0006347
UUUGGGAGGCCGAGGCUGGUGCAUCACUUGAGCCCAGCAAUUUGAGACCAAUCUGGGCAACAAAG
UGAGACCUCCGUCUCUACAAAGA
>hsa-mir-1286 MI0006348
UGUCCUCUGGGGACUCAGCUUGCUCUGGCUGCUGGAUUGAAUUAGCUGCAGGACCAAGAUGAGCC
CUUGGUGGAGACA
>hsa-mir-1287 MI0006349
GUUGUGCUGUCCAGGUGCUGGAUCAGUGGUUCGAGUCUGAGCCUUUAAAAGCCACUCUAGCCACA
GAUGCAGUGAUUGGAGCCAUGACAA
>hsa-mir-1288 MI0006432
GAGGGUGUUGAUCAGCAGAUCAGGACUGUAACUCACCAUAGUGGUGGACUGCCCUGAUCUGGAGA
CCACUGCCUU
>hsa-mir-1289-1 MI0006350
UUCUCAAUUUUUAGUAGGAAUUAAAAACAAAACUGGUAAAUGCAGACUCUUGGUUUCCACCCCCA
GAGAAUCCCUAAACCGGGGGUGGAGUCCAGGAAUCUGCAUUUUAGAAAGUACCCAGGGUGAUUCU
GAUAAUUGGGAACA
>hsa-mir-1289-2 MI0006351
CCACGGUCCUAGUUAAAAAGGCACAUUCCUAGACCCUGCCUCAGAACUACUGAACAGAGUCACUG
GGUGUGGAGUCCAGGAAUCUGCAUUUUUACCCCUAUCGCCCCCGCC
>hsa-mir-1290 MI0006352
GAGCGUCACGUUGACACUCAAAAAGUUUCAGAUUUUGGAACAUUUCGGAUUUUGGAUUUUUGGAU
CAGGGAUGCUCAA
>hsa-mir-1291 MI0006353
GGUAGAAUUCCAGUGGCCCUGACUGAAGACCAGCAGUUGUACUGUGGCUGUUGGUUUCAAGCAGA
GGCCUAAAGGACUGUCUUCCUG
>hsa-mir-1292 MI0006433
CCUGGGAACGGGUUCCGGCAGACGCUGAGGUUGCGUUGACGCUCGCGCCCCGGCUCCCGUUCCAG
G
>hsa-mir-1293 MI0006355
AGGUUGUUCUGGGUGGUCUGGAGAUUUGUGCAGCUUGUACCUGCACAAAUCUCCGGACCACUUAG

UCUUUA
>hsa-mir-1294 MI0006356
CACCUAAUGUGUGCCAAGAUCUGUUCAUUUAUGAUCUCACCGAGUCCUGUGAGGUUGGCAUUGUU
GUCUGGCAUUGUCUGAUAUACAACAGUGCCAACCUCACAGGACUCAGUGAGGUGAAACUGAGGAU
UAGGAAGGUGUA
>hsa-mir-1295a MI0006357
AGGACAUUUUGCCCAGAUCCGUGGCCUAUUCAGAAAUGUGGCCUGUGAUUAGGCCGCAGAUCUGG
GUGAAAUGUCCUCC
>hsa-mir-1295b MI0019146
CACCCAGAUCUGCGGCCUAAUCACAGGCCACAUUUCUGAAUAGGCCACGGAUCUGGGCAA
>hsa-mir-1296 MI0003780
ACCUACCUAACUGGGUUAGGGCCCUGGCUCCAUCUCCUUUAGGAAAACCUUCUGUGGGGAGUGGG
GCUUCGACCCUAACCCAGGUGGGCUGU
>hsa-mir-1297 MI0006358
UGUUUAUCUCUAGGGUUGAUCUAUUAGAAUUACUUAUCUGAGCCAAAGUAAUUCAAGUAAUUCAG
GUGUAGUGAAAC
>hsa-mir-1298 MI0003938
AGACGAGGAGUUAAGAGUUCAUUCGGCUGUCCAGAUGUAUCCAAGUACCCUGUGUUAUUUGGCAA
UAAAUACAUCUGGGCAACUGACUGAACUUUUCACUUUUCAUGACUCA
>hsa-mir-1299 MI0006359
CCUCAUGGCAGUGUUCUGGAAUCCUACGUGAGGGACAAUCAUUCAGACCCACGUAGCAGUGUUCU
GGAAUUCUGUGUGAGGGA
>hsa-mir-1301 MI0003815
GGAUUGUGGGGGGUCGCUCUAGGCACCGCAGCACUGUGCUGGGGAUGUUGCAGCUGCCUGGGAGU
GACUUCACACAGUCCUC
>hsa-mir-1302-1 MI0006362
CAGAAAGCCCAGUUAAAUUUGAAUUUCAAGUAAACAAUGAAUAAUUGUGUAUGUAAGAAUAUCCC
AUACAAUAUUUGGGACAUACUUAUGCUAAAAAUUAUUCCUUGCUUAUCUGAAAUUCAAAUGUAAC
UAGGAUUCCUGUA
>hsa-mir-1302-10 MI0015979
GGAUGCCCAGCUAGUUUGAAUUUUAGAUAAACAACGAAUAAUUUCGUAGCAUAAAUAUGUCCCAA
GCUUAGUUUGGGACAUACUUAUGCUAAAAAACAUUAUUGGUUGUUUAUCUGAGAUUCAGAAUUAA
GCAUUUUA
>hsa-mir-1302-4 MI0006365
AAUGCAGAAGCACAGCUAAAAUUUGAAUUUCAGAUAAACAAAUUUUUCUUAGAAUAAGUAUGUCU
CCAUGCAACAUUUGGGACAUACUUAUGCUAAAAUAUUAUUUGUGUUUCAUCUGAAAUUCAAAUUC
AACUGGACAUCCUGUAUUUU
>hsa-mir-1302-5 MI0006366
UGCCCGGCCUCCCAUUAAAUUGGUUUUUCAGACAAAUCACAAAUUUGUUUAGGUAUAAGUAUAUC
CCAUGUAAUCUUUGGGACAUACUUAUGCUAAAAUAAUUGUUCCUUGUUGAUUGGAAAUUUUAAUU
UUAAUUAGGUGUCCUGUAUU
>hsa-mir-1302-6 MI0006367
AACAAAUAAUUUGGUAAUAUAUGUAUGGCCCACACAAUAUUUAGGACAACAAUAUUUGGGACAUA
CUUAUGCUAAAAAAGUAUUUGUUGA

>hsa-mir-1302-8 MI0006369
CCCAUUUAAACUUGAAUUUCAUAUAAACACCGUAAUUUUCAGCAUUAGUGUAUCACAUGCAGUAU
UUGGGACAUACUUAUGCUAAAAAAUUAGGUGGUGUUGAUCUGAAAUUCCAGUGUAGAUGGGCA
>hsa-mir-1303 MI0006370
GGCUGGGCAACAUAGCGAGACCUCAACUCUACAAUUUUUUUUUUUUAAAUUUUAGAGACGGGGU
CUUGCUCUGUUGCCAGGCUUU
>hsa-mir-1304 MI0006371
AAACACUUGAGCCCAGCGGUUUGAGGCUACAGUGAGAUGUGAUCCUGCCACAUCUCACUGUAGCC
UCGAACCCCUGGGCUCAAGUGAUUCA
>hsa-mir-1305 MI0006372
AAGAUCCUGCUGUUUCUACCAUUAGUUUUGAAUGUUUAUUGUAAAGAUACUUUUCAACUCUAAUG
GGAGAGACAGCAGGAUUCUCC
>hsa-mir-1306 MI0006443
GUGAGCAGUCUCCACCACCUCCCCUGCAAACGUCCAGUGGUGCAGAGGUAAUGGACGUUGGCUCU
GGUGGUGAUGGACAGUCCGA
>hsa-mir-1307 MI0006444
CAUCAAGACCCAGCUGAGUCACUGUCACUGCCUACCAAUCUCGACCGGACCUCGACCGGCUCGUC
UGUGUUGCCAAUCGACUCGGCGUGGCGUCGGUCGUGGUAGAUAGGCGGUCAUGCAUACGAAUUUU
CAGCUCUUGUUCUGGUGAC
>hsa-mir-1321 MI0006652
ACAUUAUGAAGCAAGUAUUAUUAUCCCUGUUUUACAAAUAAGGAAAUAAACUCAGGGAGGUGAAU
GUGAUCAAAGAUAG
>hsa-mir-1322 MI0006653
AGUAUCAUGAAUUAGAAACCUACUUAUUACAUAGUUUACAUAAGAAGCGUGAUGAUGCUGCUGAU
GCUGUA
>hsa-mir-1323 MI0003786
ACUGAGGUCCUCAAAACUGAGGGGCAUUUUCUGUGGUUUGAAAGGAAAGUGCACCCAGUUUUGGG
GAUGUCAA
>hsa-mir-1324 MI0006657
CCUGAAGAGGUGCAUGAAGCCUGGUCCUGCCCUCACUGGGAACCCCCUUCCCUCUGGGUACCAGA
CAGAAUUCUAUGCACUUUCCUGGAGGCUCCA
>hsa-mir-1343 MI0017320
GCUGGCGUCGGUGCUGGGGAGCGGCCCCCGGGUGGGCCUCUGCUCUGGCCCCUCCUGGGGCCCGC
ACUCUCGCUCUGGGCCCGC
>hsa-mir-1468 MI0003782
GGUGGGUGGUUUCUCCGUUUGCCUGUUUCGCUGAUGUGCAUUCAACUCAUUCUCAGCAAAAUAAG
CAAAUGGAAAAUUCGUCCAUC
>hsa-mir-1470 MI0007075
GCCCUCCGCCCGUGCACCCCGGGGCAGGAGACCCCGCGGGACGCGCCGAGGUAGGGGGGAC
>hsa-mir-1471 MI0007076
GCCCGCGUGUGGAGCCAGGUGUAGAGGCGGAGCACAGCUGGCUCUAAUUUGAGGGGC
>hsa-mir-1537 MI0007258
ACAGCUGUAAUUAGUCAGUUUUCUGUCCUGUCCACACAGAAAACCGUCUAGUUACAGUUGU
>hsa-mir-1538 MI0007259

GGGAACAGCAGCAACAUGGGCCUCGCUUCCUGCCGGCGCGGCCCGGGCUGCUGCUGUUCCU
>hsa-mir-1539 MI0007260
GGCUCUGCGGCCUGCAGGUAGCGCGAAAGUCCUGCGCGUCCCAGAUGCCC
>hsa-mir-1587 MI0016905
UUUGGGCUGGGCUGGGUUGGGCAGUUCUUCUGCUGGACUCACCUGUGACCAGC
>hsa-mir-1825 MI0008193
AGAGACUGGGGUGCUGGGCUCCCCUAGACUAGGACUCCAGUGCCCUCCUCUCC
>hsa-mir-1827 MI0008195
UCAGCAGCACAGCCUUCAGCCUAAAGCAAUGAGAAGCCUCUGAAAGGCUGAGGCAGUAGAUUGAA
U
>hsa-mir-1908 MI0008329
CGGGAAUGCCGCGGCGGGGACGGCGAUUGGUCCGUAUGUGUGGUGCCACCGGCCGCCGGCUCCGC
CCCGGCCCCCGCCCC
>hsa-mir-1909 MI0008330
CAUCCAGGACAAUGGUGAGUGCCGGUGCCUGCCCUGGGGCCGUCCCUGCGCAGGGGCCGGGUGCU
CACCGCAUCUGCCCC
>hsa-mir-1910 MI0008331
UGUCCCUUCAGCCAGUCCUGUGCCUGCCGCCUUUGUGCUGUCCUUGGAGGGAGGCAGAAGCAGGA
UGACAAUGAGGGCAA
>hsa-mir-1911 MI0008332
UCGGCAUCUGCUGAGUACCGCCAUGUCUGUUGGGCAUCCACAGUCUCCCACCAGGCAUUGUGGUC
UCCGCUGACGCUUUG
>hsa-mir-1912 MI0008333
CUCUAGGAUGUGCUCAUUGCAUGGGCUGUGUAUAGUAUUAUUCAAUACCCAGAGCAUGCAGUGUG
AACAUAAUAGAGAUU
>hsa-mir-1913 MI0008334
ACCUCUACCUCCCGGCAGAGGAGGCUGCAGAGGCUGGCUUUCCAAAACUCUGCCCCCUCCGCUGC
UGCCAAGUGGCUGGU
>hsa-mir-1914 MI0008335
CGUGUGAGCCCGCCCUGUGCCCGGCCCACUUCUGCUUCCUCUUAGCGCAGGAGGGGUCCCGCACU
GGGAGGGGCCCUCAC
>hsa-mir-1915 MI0008336
UGAGAGGCCGCACCUUGCCUUGCUGCCCGGGCCGUGCACCCGUGGGCCCCAGGGCGACGCGGCGG
GGGCGGCCCUAGCGA
>hsa-mir-1972-1 MI0009982
UAUAGGCAUGUGCCACCACACCUGGCUUAAAUGUGUCAUUUAAAAAUUCAGGCCAGGCACAGUGG
CUCAUGCCUGUA
>hsa-mir-1973 MI0009983
UAUGUUCAACGGCCAUGGUAUCCUGACCGUGCAAAGGUAGCAUA
>hsa-mir-1976 MI0009986
GCAGCAAGGAAGGCAGGGGUCCUAAGGUGUGUCCUCCUGCCCUCCUUGCUGU
>hsa-mir-2052 MI0010486
CUGUUUUGAUAACAGUAAUGUCCCUUUAGUUCAAAGUUACCAGCUAUCAAAACAA
>hsa-mir-2053 MI0010487

CUUGCCAUGUAAAUACAGAUUUAAUUAACAUUUGCAACCUGUGAAGAUGCAAAACUUUAAGUGUU
AAUUAAACCUCUAUUUACAUAGCAAG
>hsa-mir-2054 MI0010488
CUGUAAUAUAAAUUUAAUUUAUUCUCUAUCAUUAAAAAAUGUAUUACAG
>hsa-mir-2110 MI0010629
CAGGGGUUUGGGGAAACGGCCGCUGAGUGAGGCGUCGGCUGUGUUUCUCACCGCGGUCUUUUCCU
CCCACUCUUG
>hsa-mir-2113 MI0003939
UUUUCAAAGCAAUGUGUGACAGGUACAGGGACAAAUCCCGUUAAUAAGUAAGAGGAUUUGUGCUU
GGCUCUGUCACAUGCCACUUUGAAAA
>hsa-mir-2114 MI0010633
CCUCCAUGCUCCUAGUCCCUUCCUUGAAGCGGUCGGAUAAUCACAUGACGAGCCUCAAGCAAGGG
ACUUCAAGCUGGUGG
>hsa-mir-2115 MI0010634
ACUGUCAUCCCACUGCUUCCAGCUUCCAUGACUCCUGAUGGAGGAAUCACAUGAAUUCAUCAGAA
UUCAUGGAGGCUAGAAGCAGUAUGAGGAUCAUUUA
>hsa-mir-2116 MI0010635
GACCUAGGCUAGGGGUUCUUAGCAUAGGAGGUCUUCCCAUGCUAAGAAGUCCUCCCAUGCCAAGA
ACUCCCAGACUAGGA
>hsa-mir-2117 MI0010636
GCUCUGAUUUACUUCUGUCCGGCAUGGUGAACAGCAGGAUUGGCUGUAGCUGUUCUCUUUGCCAA
GGACAGAUCUGAUCU
>hsa-mir-2276 MI0011282
GUGUUCUUCCAGUCCGCCCUCUGUCACCUUGCAGACGGCUUUCUCUCCGAAUGUCUGCAAGUGUC
AGAGGCGAGGAGUGGCAGCUGCAU
>hsa-mir-2277 MI0011284
GUGCUUCCUGCGGGCUGAGCGCGGGCUGAGCGCUGCCAGUCAGCGCUCACAUUAAGGCUGACAGC
GCCCUGCCUGGCUCGGCCGGCGAAGCUC
>hsa-mir-2278 MI0011285
GUGCUGCAGGUGUUGGAGAGCAGUGUGUGUUGCCUGGGGACUGUGUGGACUGGUAUCACCCAGAC
AGCUUGCACUGACUCCAGACCCUGCCGUCAU
>hsa-mir-2355 MI0015873
CAGACGUGUCAUCCCCAGAUACAAUGGACAAUAUGCUAUUAUAAUCGUAUGGCAUUGUCCUUGCU
GUUUGGAGAUAAUACUGCUGAC
>hsa-mir-2392 MI0016870
AUGGUCCCUCCCAAUCCAGCCAUUCCUCAGACCAGGUGGCUCCCGAGCCACCCCAGGCUGUAGGA
UGGGGGUGAGAGGUGCUAG
>hsa-mir-2467 MI0017432
GGACAGGCACCUGAGGCUCUGUUAGCCUUGGCUCUGGGUCCUGCUCCUUAGAGCAGAGGCAGAGA
GGCUCAGGGUCUGUCU
>hsa-mir-2681 MI0012062
GCCCCCUUUUCACGCAUUUGUGUUUUACCACCUCCAGGAGACUGCCCAAAGACUCUUCAGUAUCA
UGGAGUUGGUAAAGCACAGAUGCAUGAAUAAUUCAACGUG
>hsa-mir-2682 MI0012063

ACCUUCCUGAAAGAGGUUGGGGCAGGCAGUGACUGUUCAGACGUCCAAUCUCUUUGGGACGCCUC
UUCAGCGCUGUCUUCCCUGCCUCUGCCUUUAGGACGAGUCUCAAA
>hsa-mir-2861 MI0013006
GGCGCCUCUGCAGCUCCGGCUCCCCCUGGCCUCUCGGGAACUACAAGUCCCAGGGGGCCUGGCGG
UGGGCGGCGGGCGGAAGAGGCGGGG
>hsa-mir-2909 MI0013083
GGUGUUAGGGCCAACAUCUCUUGGUCUUUCCCCUGUGGUCCCAAGAUGGCUGUUGCAACUUAACG
CCAU
>hsa-mir-3064 MI0017375
GGUCUGGCUGUUGUGGUGUGCAAAACUCCGUACAUUGCUAUUUUGCCACACUGCAACACCUUACA
G
>hsa-mir-3065 MI0014228
CUGCCCUCUUCAACAAAAUCACUGAUGCUGGAGUCGCCUGAGUCAUCACUCAGCACCAGGAUAUU
GUUGGAGAGGACAG
>hsa-mir-3074 MI0014181
GCUCGACUCCUGUUCCUGCUGAACUGAGCCAGUGUGUAAAAUGAGAACUGAUAUCAGCUCAGUAG
GCACCGGAGGGCGGGU
>hsa-mir-3115 MI0014127
UCUGAAUAUGGGUUUACUAGUUGGUGGUGAAUUCAUGAGUCGCCAACUAUUAGGCCUUUAUGUCC
AGA
>hsa-mir-3116-1 MI0014128
CUUUAUUGAGUCCCUACUAUGUUCCAGGCACUGGGUAUCGUAGGUGCCUGGAACAUAGUAGGGAC
UCAAUAAAG
>hsa-mir-3117 MI0014130
CCCUAAAGGGCCAGACACUAUACGAGUCAUAUAAGGGAAGGCAUUAUAGGACUCAUAUAGUGCCA
GGUGUUUUGUGGG
>hsa-mir-3118-5 MI0014243
CACACAUACAAUAAUAUUCAUAAUGCAAUCACACACAAUCACCAUGUGACUGCAUUAUGAAAAUU
CUUCUAGUGUG
>hsa-mir-3119-1 MI0014134
AUUAACUCUGGCUUUUAACUUUGAUGGCAAAGGGGUAGCUAAACAAUCUAUGUCUUUGCCAUCAA
AGUUAAAAGCCAUAGUUAAU
>hsa-mir-3120 MI0014136
GUCAUGUGACUGCCUGUCUGUGCCUGCUGUACAGGUGAGCGGAUGUUCUGCACAGCAAGUGUAGA
CAGGCAGACACAUGAC
>hsa-mir-3121 MI0014137
AAAUGGUUAUGUCCUUUGCCUAUUCUAUUUAAGACACCCUGUACCUUAAAUAGAGUAGGCAAAGG
ACAGAAACAUUU
>hsa-mir-3122 MI0014138
ACCAGCUCUGUUGGGACAAGAGGACGGUCUUCUUUUGGAAGGAAGACCAUCAUCUUGUCCGAAGA
GAGCUGGU
>hsa-mir-3123 MI0014139
AUGGAUUUGAUUGAAUGAUUCUCCCAUUUCCACAUGGAGAGUGGAGCCCAGAGAAUUGUUUAAUC
AUGUAUCCAU

>hsa-mir-3124 MI0014140
GCGGGCUUCGCGGGCGAAGGCAAAGUCGAUUUCCAAAAGUGACUUUCCUCACUCCCGUGAAGUCG
GC
>hsa-mir-3125 MI0014142
GAGAAUGGGUAGAGGAAGCUGUGGAGAGAACUCACGGUGCCUGUGGUUCGAGAUCCCCGCCUUCC
UCCUCCUUUCCUC
>hsa-mir-3126 MI0014143
AUGAUUAUAUGAGGGACAGAUGCCAGAAGCACUGGUUAUGAUUUGCAUCUGGCAUCCGUCACACA
GAUAAUUAU
>hsa-mir-3127 MI0014144
GGCCAGGCCCAUCAGGGCUUGUGGAAUGGGAAGGAGAAGGGACGCUUCCCCUUCUGCAGGCCUGC
UGGGUGUGGCU
>hsa-mir-3128 MI0014145
UUCCUCUGGCAAGUAAAAAACUCUCAUUUUCCUUAAAAAAUGAGAGUUUUUUACUUGCAAUAGGA
A
>hsa-mir-3129 MI0014146
GUACUUGGGCAGUAGUGUAGAGAUUGGUUUGCCUGUUAAUGAAUUCAAACUAAUCUCUACACUGC
UGCCCAAGAGC
>hsa-mir-3130-1 MI0014147
CUUGUCAUGUCUUACCCAGUCUCCGGUGCAGCCUGUUGUCAAGGCUGCACCGGAGACUGGGUAAG
ACAUGACAAG
>hsa-mir-3131 MI0014151
GAGUCGAGGACUGGUGGAAGGGCCUUUCCCCUCAGACCAAGGCCCUGGCCCCAGCUUCUUCUC
>hsa-mir-3132 MI0014152
GGUGGGAUGGGUAGAGAAGGAGCUCAGAGGACGGUGCGCCUUGUUUCCCUUGAGCCCUCCCUCUC
UCAUCCCACC
>hsa-mir-3133 MI0014153
CAGAAAUUGUAAAGAACUCUUAAAACCCAAUAGUAAAAAGACAACCUGUUGAGUUUUAAGAGUUC
UUUAUAUAUUCUG
>hsa-mir-3134 MI0014155
UGUAUCCAAUGUGUAGUCUUUUAUCCCUCACAUGGAGUAAAAUAUGAUGGAUAAAAGACUACAUA
UUGGGUACA
>hsa-mir-3135a MI0014156
UCACUUUGGUGCCUAGGCUGAGACUGCAGUGGUGCAAUCUCAGUUCACUGCAGCCUUGACCUCCU
GGGCUCAGGUGA
>hsa-mir-3135b MI0016809
UGCCCAGGCUGGAGCGAGUGCAGUGGUGCAGUCAGUCCUAGCUCACUGCAGCCUCGAACUCCUGG
GCU
>hsa-mir-3136 MI0014158
AAUAUGAAACUGACUGAAUAGGUAGGGUCAUUUUUCUGUGACUGCACAUGGCCCAACCUAUUCAG
UUAGUUCCAUAUU
>hsa-mir-3137 MI0014160
UACAGGUCUGUAGCCUGGGAGCAAUGGGGUGUAUGGUAUAGGGGUAGCCUCGUGCUCCUGGGCUA
CAAACCUGUA

>hsa-mir-3138 MI0014161
CCCUCCUCGGCACUUCCCCCACCUCACUGCCCGGGUGCCCACAAGACUGUGGACAGUGAGGUAGA
GGGAGUGCCGAGGAGGG
>hsa-mir-3139 MI0014162
GGCUCAGAGUAGGAGCUCAACAGAUGCCUGUUGACUGAAUAAUAAACAGGUAUCGCAGGAGCUUU
UGUUAUGUGCC
>hsa-mir-3140 MI0014163
CCUCUUGAGGUACCUGAAUUACCAAAAGCUUUAUGUAUUCUGAAGUUAUUGAAAAUAAGAGCUUU
UGGGAAUUCAGGUAGUUCAGGAGUG
>hsa-mir-3141 MI0014165
UCACCCGGUGAGGGCGGGUGGAGGAGGAGGGUCCCCACCAUCAGCCUUCACUGGGACGGGA
>hsa-mir-3142 MI0014166
UUCAGAAAGGCCUUUCUGAACCUUCAGAAAGGCUGCUGAAUCUUCAGAAAGGCCUUUCUGAACCU
UCAGAAAGGCUGCUGAA
>hsa-mir-3143 MI0014167
UAGAUAACAUUGUAAAGCGCUUCUUUCGCGGUUGGGCUGGAGCAACUCUUUACAAUGUUUCUA
>hsa-mir-3144 MI0014169
AACUACACUUUAAGGGGACCAAAGAGAUAUAUAGAUAUCAGCUACCUAUAUACCUGUUCGGUCUC
UUUAAAGUGUAGUU
>hsa-mir-3145 MI0014170
UAUAUGAGUUCAACUCCAAACACUCAAAACUCAUUGUUGAAUGGAAUGAGAUAUUUUGAGUGUUU
GGAAUUGAACUCGUAUA
>hsa-mir-3146 MI0014172
GCUAAGUCCCUUCUUUCUAUCCUAGUAUAACUUGAAGAAUUCAAAUAGUCAUGCUAGGAUAGAAA
GAAUGGGACUUGGC
>hsa-mir-3147 MI0014173
GUCCGGGUUGGGCAGUGAGGAGGGUGUGACGCCGCGAAGUGCACCUCGCCCUUGUCCAACUCGGA
C
>hsa-mir-3148 MI0014175
GAGUUAAGAUGGAAAAAACUGGUGUGUGCUUAUUGAUGUAGCCAACAAGCAUACAUCAGUUUUUU
CCAACUUAACUC
>hsa-mir-3149 MI0014176
AUACAUACAUGUACACACACAUGUCAUCCACACACAUACAUAUAUAUAUGUUUGUAUGGAUAUGU
GUGUGUAUGUGUGUGUAU
>hsa-mir-3150b MI0016426
GAGGGAAAGCAGGCCAACCUCGAGGAUCUCCCCAGCCUUGGCGUUCAGGUGCUGAGGAGAUCGUC
GAGGUUGGCCUGCUUCCCCUC
>hsa-mir-3151 MI0014178
GGGGUGAUGGGUGGGGCAAUGGGAUCAGGUGCCUCAAAGGGCAUCCCACCUGAUCCCACAGCCCA
CCUGUCACCCC
>hsa-mir-3152 MI0014179
GUGCAGAGUUAUUGCCUCUGUUCUAACACAAGACUAGGCUUCCCUGUGUUAGAAUAGGGGCAAUA
ACUCUGCAC
>hsa-mir-3153 MI0014180

GACAAAUUUUAAAUGUCCCUGUCCCCUUCCCCCCAAUUAAAGUAGAUUGGGGGAAAGCGAGUAGG
GACAUUUAAAAUUUGUU
>hsa-mir-3154 MI0014182
GGCCCCUCCUUCUCAGCCCCAGCUCCCGCUCACCCCUGCCACGUCAAAGGAGGCAGAAGGGGAGU
UGGGAGCAGAGAGGGGACC
>hsa-mir-3155a MI0014183
UCCGGGCAUCACCUCCCACUGCAGAGCCUGGGGAGCCGGACAGCUCCCUUCCCAGGCUCUGCAGU
GGGAACUGAUGCCUGGA
>hsa-mir-3156-2 MI0014230
UGCAGAAGAAAGAUCUGGAAGUGGGAGACACUUUCACUAUAUAUAGUGGCUCCCACUUCCAGAUC
UUUCUCUCUGUA
>hsa-mir-3157 MI0014185
GGGAAGGGCUUCAGCCAGGCUAGUGCAGUCUGCUUUGUGCCAACACUGGGGUGAUGACUGCCCUA
GUCUAGCUGAAGCUUUUCCC
>hsa-mir-3158-1 MI0014186
AUUCAGGCCGGUCCUGCAGAGAGGAAGCCCUUCUGCUUACAGGUAUUGGAAGGGCUUCCUCUCUG
CAGGACCGGCCUGAAU
>hsa-mir-3159 MI0014188
CCAAAGUCCUAGGAUUACAAGUGUCGGCCACGGGCUGGGCACAGUGGCUCACGCCUGUAAUCCCA
GCAUUUUGG
>hsa-mir-3160-1 MI0014189
GGACCUGCCCUGGGCUUUCUAGUCUCAGCUCUCCUCCAGCUCAGCUGGUCAGGAGAGCUGAGACU
AGAAAGCCCAGGGCAGGUUC
>hsa-mir-3161 MI0014191
CCUCGAGAGCUGAUAAGAACAGAGGCCCAGAUUGAAGUUGAAUAGUGCUGGGCCUUUGUUUUUAC
CAAGUUCCCUGG
>hsa-mir-3162 MI0014192
CUGACUUUUUUAGGGAGUAGAAGGGUGGGGAGCAUGAACAAUGUUUCUCACUCCCUACCCCUCCA
CUCCCCAAAAAAGUCAG
>hsa-mir-3163 MI0014193
UUCCUCAUCUAUAAAAUGAGGGCAGUAAGACCUUCCUUCCUUGUCUUACUACCCCCAUUUUAUAG
AUGAGGAA
>hsa-mir-3164 MI0014194
CUUGGAAACUGUGACUUUAAGGGAAAUGGCGCACAGCAGACCCUGCAAUCAUGCCGUUUUGCUUG
AAGUCGCAGUUUCCCAGG
>hsa-mir-3165 MI0014195
CCGGUGGCAAGGUGGAUGCAAUGUGACCUCAACUCUUGGUCCUCUGAGGUCACAUUGUAUCCACC
UUACCACUGG
>hsa-mir-3166 MI0014196
AAAUUUUUUUGAGGCCAGUAGGCAUUGUCUGCGUUAGGAUUUCUGUAUCAUCCUCCUAACGCAGA
CAAUGCCUACUGGCCUAAGAAAAAUUU
>hsa-mir-3167 MI0014198
GGCUGUGGAGGCACCAGUAUUUCUGAAAUUCUUUUUUCUGAAAUUCUUCAGGAAGGAUUUCAGAA
AUACUGGUGUCCCGACAGCC

>hsa-mir-3168 MI0014199
AAGAUCAUGAGUUCUACAGUCAGACAGCCUGAGUUGGAGGCUCAUCUUCACUUCUUGCUGUGUGA
CCCUGGGCCAGUGACUU
>hsa-mir-3169 MI0014200
AUGUGAAAACAUAGGACUGUGCUUGGCACAUAGCACAAAGUCUUAUGGUACUGUGUGCCAAGCAU
AGUCCUGUGUUUUUACAU
>hsa-mir-3170 MI0014201
CUGGUAACACUGGGGUUCUGAGACAGACAGUGUUAGCUCCAGAAGCAUUGCCUGUCUUAGAACCC
CUAUGUUACCAG
>hsa-mir-3171 MI0014202
UAUAUAUAGAGAUGUAUGGAAUCUGUAUAUAUCUAUAUAUAUGUGUAUAUAUAGAUUCCAUAAAU
CUAUAUAUG
>hsa-mir-3173 MI0014204
UCCCUGCCCUGCCUGUUUUCUCCUUUGUGAUUUUAUGAGAACAAAGGAGGAAAUAGGCAGGCCAG
GGA
>hsa-mir-3174 MI0014208
GUUACCUGGUAGUGAGUUAGAGAUGCAGAGCCCUGGGCUCCUCAGCAAACCUACUGGAUCUGCAU
UUUAAUUCACAUGCAUGGUAAU
>hsa-mir-3175 MI0014209
CCUGGGGGGCGGGGAGAGAACGCAGUGACGUCUGGCCGCGUGCGCAUGUCGGGCGCUUUCUCCUC
CCCCUACCCAGG
>hsa-mir-3176 MI0014210
UGGCCUCUCCAGUCUGCAGCUCCCGGCAGCCUCGGGCCACACUCCCGGGAUCCCCAGGGACUGGC
CUGGGACUACCGGGGGUGGCGGCCG
>hsa-mir-3177 MI0014211
CCACGUGCCAUGUGUACACACGUGCCAGGCGCUGUCUUGAGACAUUCGCGCAGUGCACGGCACUG
GGGACACGUGGCACUGG
>hsa-mir-3178 MI0014212
GAGGCUGGGCGGGGCGCGGCCGGAUCGGUCGAGAGCGUCCUGGCUGAUGACGGUCUCCCGUGCCC
ACGCCCCAAACGCAGUCUC
>hsa-mir-3179-1 MI0014213
CAGGAUCACAGACGUUUAAAUUACACUCCUUCUGCUGUGCCUUACAGCAGUAGAAGGGGUGAAAU
UUAAACGUCUGUGAUCCUG
>hsa-mir-3180-1 MI0014214
CAGUGCGACGGGCGGAGCUUCCAGACGCUCCGCCCCACGUCGCAUGCGCCCCGGGAAAGCGUGGG
GCGGAGCUUCCGGAGGCCCCGCCCUGCUG
>hsa-mir-3180-4 MI0016408
GCUCCGCCCCACGUCGCAUGCGCCCCGGGAACGCGUGGGGCGGAGCUUCCGGAGGCCCCGCUCUG
CUGCCGACCCUGUGGAGCGGAGGGUGAAGCCUCCGGAUGCCAGUCCCUCAUCGCUGGCCUGGUCG
CGCUGUGGCGAAGGGGGCGGAGC
>hsa-mir-3181 MI0014223
CGGCGACCAUCGGGCCCUCGGCGCCGGCCCGUUAGUUGCCCGGGCCCGAGCCGGCCGGGCCCGCG
GGUUGCCG
>hsa-mir-3182 MI0014224

GCUGCUUCUGUAGUGUAGUCCGUGCAUCCGCCCUUCGAUGCUUGGGUUGGAUCAUAGAGCAGU
>hsa-mir-3183 MI0014225
CUCUGCCCUGCCUCUCUCGGAGUCGCUCGGAGCAGUCACGUUGACGGAAUCCUCCGGCGCCUCCU
CGAGGGAGGAGAGGCAGGG
>hsa-mir-3184 MI0014226
AAGCAAGACUGAGGGGCCUCAGACCGAGCUUUUGGAAAAUAGAAAAGUCUCGCUCUCUGCCCCUC
AGCCUAACUU
>hsa-mir-3185 MI0014227
GAAUGGAAGAAGAAGGCGGUCGGUCUGCGGGAGCCAGGCCGCAGAGCCAUCCGCCUUCUGUCCAU
GUC
>hsa-mir-3186 MI0014229
AGCCUGCGGUUCCAACAGGCGUCUGUCUACGUGGCUUCAACCAAGUUCAAAGUCACGCGGAGAGA
UGGCUUUGGAACCAGGGGCU
>hsa-mir-3187 MI0014231
GCUGGCCCUGGGCAGCGUGUGGCUGAAGGUCACCAUGUUCUCCUUGGCCAUGGGGCUGCGCGGGG
CCAGC
>hsa-mir-3188 MI0014232
GGCGCCUCCUGCUCUGCUGUGCCGCCAGGGCCUCCCCUAGCGCGCCUUCUGGAGAGGCUUUGUGC
GGAUACGGGGCUGGAGGCCU
>hsa-mir-3189 MI0014233
GCCUCAGUUGCCCCAUCUGUGCCCUGGGUAGGAAUAUCCUGGAUCCCCUUGGGUCUGAUGGGGUA
GCCGAUGC
>hsa-mir-3190 MI0014235
CUGGGGUCACCUGUCUGGCCAGCUACGUCCCCACGGCCCUUGUCAGUGUGGAAGGUAGACGGCCA
GAGAGGUGACCCCGG
>hsa-mir-3191 MI0014236
GGGGUCACCUCUCUGGCCGUCUACCUUCCACACUGACAAGGGCCGUGGGGACGUAGCUGGCCAGA
CAGGUGACCCC
>hsa-mir-3192 MI0014237
GGAAGGGAUUCUGGGAGGUUGUAGCAGUGGAAAAAGUUCUUUUCUUCCUCUGAUCGCCCUCUCAG
CUCUUUCCUUCU
>hsa-mir-3193 MI0014238
UCCUGCGUAGGAUCUGAGGAGUGGACGAGUCUCAUUACCCAGCUCCUGAGCAGGA
>hsa-mir-3194 MI0014239
AGGUGGCAGGGCCAGCCACCAGGAGGGCUGCGUGCCACCCGGGCAGCUCUGCUGCUCACUGGCAG
UGUCACCU
>hsa-mir-3195 MI0014240
CCGCAGCCGCCGCGCCGGGCCCGGGUUGGCCGCUGACCCCCGCGGGGCCCCCGGCGGCCGGGGCG
GGGGCGGGGGCUGCCCCGG
>hsa-mir-3196 MI0014241
GGGUGGGGGCGGGGCGGCAGGGGCCUCCCCCAGUGCCAGGCCCCAUUCUGCUUCUCUCCCAGCU
>hsa-mir-3197 MI0014245
GGCGAGGGGAGGCGCAGGCUCGGAAAGGCGCGCGAGGCUCCAGGCUCCUUCCCGAUCCACCGCUC
UCCUCGCU

>hsa-mir-3198-1 MI0014246
GACUGUGCUCUCACUGUUCACCCAGCACUAGCAGUACCAGACGGUUCUGUGGAGUCCUGGGGAAU
GGAGAGAGCACAGUC
>hsa-mir-3199-1 MI0014247
GGUGACUCCAGGGACUGCCUUAGGAGAAAGUUUCUGGAAGUUCUGACAUUCCAGAAACUUUCUCC
UAAGGCAGUCCCUGGGAGUCACU
>hsa-mir-3200 MI0014249
GGUGGUCGAGGGAAUCUGAGAAGGCGCACAAGGUUUGUGUCCAAUACAGUCCACACCUUGCGCUA
CUCAGGUCUGCUCGUGCCCU
>hsa-mir-3201 MI0014250
GGGAUAUGAAGAAAAAUAAGAGGCUAGGAUUGCCUCUUAUUUUUACAUGCCC
>hsa-mir-3202-1 MI0014252
UAUUAAUAUGGAAGGGAGAAGAGCUUUAAUGAUUGGAGUCAUUUUCAGAGCAUUAAAGCUCUUCU
CCCUUCCAUAUUAAUG
>hsa-mir-3529 MI0017351
GGCACCAUUAGGUAGACUGGGAUUUGUUGUUGAGCGCAGUAAGACAACAACAAAAUCACUAGUCU
UCCAGAUGGGGCC
>hsa-mir-3591 MI0017383
CAGUAGCUAUUUAGUGUGAUAAUGGCGUUUGAUAGUUUAGACACAAACACCAUUGUCACACUCCA
CAGCUCUG
>hsa-mir-3605 MI0015995
ACUUUAUACGUGUAAUUGUGAUGAGGAUGGAUAGCAAGGAAGCCGCUCCCACCUGACCCUCACGG
CCUCCGUGUUACCUGUCCUCUAGGUGGGACGCUCG
>hsa-mir-3606 MI0015996
UUGUUGCUAUCUAGGUUAGUGAAGGCUAUUUUAAUUUUUUUAAAAUUUCUUUCACUACUUAGG
>hsa-mir-3607 MI0015997
AAGGUUGCGGUGCAUGUGAUGAAGCAAAUCAGUAUGAAUGAAUUCAUGAUACUGUAAACGCUUUC
UGAUGUACUACUCA
>hsa-mir-3609 MI0015999
GUAACAGUAACUUUUAUUCUCAUUUUCCUUUUCUCUACCUUGUAGAGAAGCAAAGUGAUGAGUAA
UACUGGCUGGAGCCC
>hsa-mir-3610 MI0016000
AAGAGCCGCGGCGUAACGGCAGCCAUCUUGUUUGUUUGAGUGAAUCGGAAAGGAGGCGCCGGCUG
UGGCGGCG
>hsa-mir-3611 MI0016001
AGCAGGUCUAAUAAGAAUUUCUUUUUCUUCACAAUUAUGAAAGAAAAGAAAUUGUGAAGAAAGAA
AUUCUUACUAGUUUUGCU
>hsa-mir-3612 MI0016002
GGGACUGGGGAUGAGGAGGCAUCUUGAGAAAUGGAAGGAAUGGGAUCUACUUCCAGUUCACUAGA
GGCGUCCUGACACCCCUAGCUC
>hsa-mir-3613 MI0016003
UGGUUGGGUUUGGAUUGUUGUACUUUUUUUUUUGUUCGUUGCAUUUUUAGGAACAAAAAAAAAAG
CCCAACCCUUCACACCACUUCA
>hsa-mir-3614 MI0016004

GGUUCUGUCUUGGGCCACUUGGAUCUGAAGGCUGCCCCUUUGCUCUCUGGGGUAGCCUUCAGAUC
UUGGUGUUUUGAAUUCUUACU
>hsa-mir-3615 MI0016005
GACUCUGGGACGCUCAGACGCCGCGCGGGGCGGGGAUUGGUCUGUGGUCCUCUCUCGGCUCCUCG
CGGCUCGCGGCGGCCGACGGUU
>hsa-mir-3616 MI0016006
UGUCACUCCGCCAGCAUCAUGAAGUGCACUCAUGAUAUGUUUGCCCCAUCAGCGUGUCACGAGGG
CAUUUCAUGAUGCAGGCGGGGUUGGCA
>hsa-mir-3617 MI0016007
AGGUCAUAGAAAGACAUAGUUGCAAGAUGGGAUUAGAAACCAUAUGUCUCAUCAGCACCCUAUGU
CCUUUCUCUGCCCU
>hsa-mir-3618 MI0016008
UAAGCUGAGUGCAUUGUGAUUUCCAAUAAUUGAGGCAGUGGUUCUAAAAGCUGUCUACAUUAAUG
AAAAGAGCAAUGUGGCCAGCUUG
>hsa-mir-3619 MI0016009
ACGGCAUCUUUGCACUCAGCAGGCAGGCUGGUGCAGCCCGUGGUGGGGGACCAUCCUGCCUGCUG
UGGGGUAAGGACGGCUGU
>hsa-mir-3620 MI0016011
GUGAGGUGGGGGCCAGCAGGGAGUGGGCUGGGCUGGGCUGGGCCAAGGUACAAGGCCUCACCCUG
CAUCCCGCACCCAG
>hsa-mir-3621 MI0016012
GUGAGCUGCUGGGGACGCGGGUCGGGGUCUGCAGGGCGGUGCGGCAGCCGCCACCUGACGCCGCG
CCUUUGUCUGUGUCCCACAG
>hsa-mir-3622b MI0016014
AGUGAUAUAAUAGAGGGUGCACAGGCAUGGGAGGUCAGGUGAGCUCAGCUCCCUGCCUCACCUGA
GCUCCCGUGCCUGUGCACCCUCUAUUGGCU
>hsa-mir-3646 MI0016046
UUCAGUAGGUUGGGUUCAUUUCAUUUUCAUGACAACCCUAUAUGGGAAAAUGUUGUGAAAAUGAA
AUGAGCCCAGCCCAUUGAA
>hsa-mir-3648 MI0016048
CGCGACUGCGGCGGCGGUGGUGGGGGGAGCCGCGGGGAUCGCCGAGGGCCGGUCGGCCGCCCCGG
GUGCCGCGCGGUGCCGCCGGCGGCGGUGAGGCCCCGCGCGUGUGUCCCGGCUGCGGUCGGCCGCG
CUCGAGGGGUCCCCGUGGCGUCCCCUUCCCCGCCGGCCGCCUUUCUCGCG
>hsa-mir-3649 MI0016049
GCUUGGAACAGGCACCUGUGUGUGCCCAAGUGUUUCUAGCAAACACAGGGACCUGAGUGUCUAAG
C
>hsa-mir-3650 MI0016050
UCAAGGUGUGUCUGUAGAGUCCUGACUGCGUGCCAGGGGCUCUGUCUGGCACAUUUCUGA
>hsa-mir-3651 MI0016051
GAUUCGAUGGGCCAUAGCAAUCCUGUGAUUUAUGCAUGGAGGCUGCUUCUCCUCAGCAGCUGCCA
UAGCCCGGUCGCUGGUACAUGAUUC
>hsa-mir-3652 MI0016052
CGGCUGGAGGUGUGAGGAUCCGAACCCAGGGGUGGGGGGUGGAGGCGGCUCCUGCGAUCGAAGGG
GACUUGAGACUCACCGGCCGCACGCCAUGAGGGCCCUGUGGGUGCUGGGCCUCUGCUGCGUCCUG

C
>hsa-mir-3653 MI0016053
UCCCUGGGGACCCCUGGCAGCCCCUCCUGAUGAUUCUUCUUCCUGAGCACGCUCAUGAUGAGCAA
ACUGAGCCUCUAAGAAGUUGACUGAAGGGGCUGCUUCCCCAAGGA
>hsa-mir-3654 MI0016054
UUCAUGAGCUGCAAUCUCAUCACUGGAAUGUUCCAGCGACUGGACAAGCUGAGGAA
>hsa-mir-3655 MI0016055
GCUUGUCGCUGCGGUGUUGCUGUUGGAGACUCGAUUGUUGGUGACAGCGAAAGAACGAUAACAAA
AUGCCGGAGCGAGAUAGU
>hsa-mir-3656 MI0016056
CUUUCGGCCAGCGGGACGGCAUCCGAGGUGGGCUAGGCUCGGGCCCGUGGCGGGUGCGGGGGUGG
GAGG
>hsa-mir-3657 MI0016057
UGUGUCCCAUAAUUAAAUAAUGAAAUCUGAAAUCACCAAUAAUGGGACACUAAUGUGAUUAAUGU
UGUUGUGUCCCAUUAUUGGUGAUUUCAGAUUUCAUAUAUGAUUAAGGACAUA
>hsa-mir-3658 MI0016058
UAUUUAAGAAAACACCAUGGAGAUGAAAUGCCUUUGAUUUUUUUUUCUUUUUGUA
>hsa-mir-3659 MI0016060
UCUACAAGCAGAUACAAGGAUGCCCUUGUACACAACACACGUGCUGCUUGUAUAGACAUGAGUGU
UGUCUACGAGGGCAUCCUUGUGUCUGUGUGUGUG
>hsa-mir-3660 MI0016061
GAAAGAAGAACUGGACAAAAUUAAAAUGCUCUUCUGUCAUUGUAAUAGUUCAUAUGGGCACUGAC
AGGAGAGCAUUUUGACUUUGUCAAGUGUGUCUGCU
>hsa-mir-3661 MI0016062
CACCUUCUCGCAGAGGCUCUUGACCUGGGACUCGGACAGCUGCUUGCACUCGUUCAGCUGCUCGA
UCCACUGGUCCAGCUCCUUGGUGAACACCUU
>hsa-mir-3662 MI0016063
UGUGUUUUCCUCAACGCUCACAGUUACACUUCUUACUCUCAAUCCAUUCAUAUUGAAAAUGAUGA
GUAGUGACUGAUGAAGCACAAAUCAGCCAA
>hsa-mir-3663 MI0016064
CCCGGGACCUUGGUCCAGGCGCUGGUCUGCGUGGUGCUCGGGUGGAUAAGUCUGAUCUGAGCACC
ACACAGGCCGGGCGCCGGGACCAAGGGGGCUC
>hsa-mir-3664 MI0016065
CUGUAAACUUGAAGGUAGGGAACUCUGUCUUCACUCAUGAGUACCUUCCAACACGAGCUCUCAGG
AGUAAAGACAGAGUUCCCUACCUUCAAUGUGGAU
>hsa-mir-3665 MI0016066
GCGGGCGGCGGCGGCGGCAGCAGCAGCAGGUGCGGGGCGGCGGCCGCGCUGGCCGCUCGACUCCG
CAGCUGCUCGUUCUGCUUCUCCAGCUUGCGCACCAGCUCC
>hsa-mir-3666 MI0016067
AGUAAGGUCCGUCAGUUGUAAUGAGACCCAGUGCAAGUGUAGAUGCCGACUCCGUGGCAGAGUUC
AGCGUUUCACACUGCCUGGUCUCUGUCACUCUAUUGAAUUAGAUUG
>hsa-mir-3667 MI0016068
UGAGGAUGAAAGACCCAUUGAGGAGAAGGUUCUGCUGGCUGAGAACCUUCCUCUCCAUGGGUCUU
UCAUCCUCA

>hsa-mir-3668 MI0016069
AUAUAUGAAAUGUAGAGAUUGAUCAAAAUAGUUUCUAUCAAAAUAGUUUGAUCAAUCUCUGCAA
UUUUAUAUAU
>hsa-mir-3670-1 MI0016071
UCUAGACUGGUAUAGCUGCUUUUGGAGCCUCACCUGCUGAGAGCUCACAGCUGUCCUUCUCUAGA
>hsa-mir-3671 MI0016072
AUGUUAUUGCUGCUGCUGUCACAUUUACAUGAAAAUAAAAUGUAAAUUAUUUUAUUUCUAUCAAA
UAAGGACUAGUCUGCAGUGAUAU
>hsa-mir-3672 MI0016073
UCUUUGUGAUUACCAUGAGACUCAUGUAAAACAUCUUAGACUAUUACAAGAUGUUUUAUGAGUCU
CAUGAUAAUCACAAAGA
>hsa-mir-3673 MI0016074
AUAUAUAUAUAUGGAAUGUAUAUACGGAAUAUAUAUAUAUAUGGAAUGUAUAUACGGAAUAUAUA
UAUAUAUGGAAUGUAUAUACGGAAUAUAUAUAUAUAU
>hsa-mir-3674 MI0016075
ACAUCACUAUUGUAGAACCUAAGAUUGGCCGUUUGAGAUGUCCUUUCAAGUUUUUGCAUUUCUGA
UGU
>hsa-mir-3675 MI0016076
GGAUGAUAAGUUAUGGGGCUUCUGUAGAGAUUUCUAUGAGAACAUCUCUAAGGAACUCCCCCAAA
CUGAAUUC
>hsa-mir-3677 MI0016078
GGCAGUGGCCAGAGCCCUGCAGUGCUGGGCAUGGGCUUCUCGUGGGCUCUGGCCACGGCC
>hsa-mir-3678 MI0016079
GAAUCCGGUCCGUACAAACUCUGCUGUGUUGAAUGAUUGGUGAGUUUGUUUGCUCAUUGAUUGAA
UCACUGCAGAGUUUGUACGGACCGGAUUC
>hsa-mir-3679 MI0016080
CGUGGUGAGGAUAUGGCAGGGAAGGGGAGUUUCCCUCUAUUCCCUUCCCCCCAGUAAUCUUCAUC
AUG
>hsa-mir-3680-1 MI0016081
AAAUUUAAGGAGGGACUCACUCACAGGAUUGUGCAAAUGCAAAGUUGGCUUUUGCAUGACCCUGG
GAGUAGGUGCCUCCUUAAAUUU
>hsa-mir-3681 MI0016082
ACUUCCAGUAGUGGAUGAUGCACUCUGUGCAGGGCCAACUGUGCACACAGUGCUUCAUCCACUAC
UGGAAGU
>hsa-mir-3682 MI0016083
UAAGUUAUAUAUGUCUACUUCUACCUGUGUUAUCAUAAUAAAGGUGUCAUGAUGAUACAGGUGGA
GGUAGAAAUAUAUAACUUA
>hsa-mir-3683 MI0016084
GGGUGUACACCCCCUGCGACAUUGGAAGUAGUAUCAUCUCUCCCUUGGAUGCUACGAACAAUAUC
ACAGAAGGUGUACACCC
>hsa-mir-3684 MI0016085
AAUCUAAAGGACCUGUACUAGGUUUAACAUGUUGAGCAUUACUCAUGUUAGACCUAGUACACGUC
CUUUAGAUU
>hsa-mir-3685 MI0016086

GUACAUUUCCUACCCUACCUGAAGACUUGAGAUUAUAGUCUUUGGGGGGAUGGGCAAAGUAC
>hsa-mir-3686 MI0016087
CUCACCUCAUUCAUUUACCUUCUCUUACAGAUCACUUUUCUGCACUGGACAGUGAUCUGUAAGAG
AAAGUAAAUGAAAGAGGUGAG
>hsa-mir-3687 MI0016088
CGCGCGUGCGCCCGAGCGCGGCCCGGUGGUCCCUCCCGGACAGGCGUUCGUGCGACGUGUG
>hsa-mir-3688-1 MI0016089
UCUUCACUUUCAAGAGUGGCAAAGUCUUUCCAUAUGUAUGUAUGUAUGUCUGUUACACAUAUGGA
AAGACUUUGCCACUCUUUAAAGUGAAGA
>hsa-mir-3689b MI0016411
GAUCCUGUGCUCCCUGGGGGGUCUGAUCCUGUGCUUCCUGGGAGGUGUGAUAUCAUGGUUCCUGG
GAGGUGUGAUCCCGUGCUUCCUGGGAGGUGUGAUAUUGUGGUUCCUGGGAGGUGUGAUCCCGUGC
UCCCUGGGAGGUGUGAUC
>hsa-mir-3689d-2 MI0016835
ACUGGGAGGUGUGAUCUCACACUCGCUGGGAGGUGUGCUAUCGUCUUCCCUGGGAGGUGUGAUCC
UGUUCUUCCUGAGCG
>hsa-mir-3689f MI0016837
AGGUGUGAUAUCGUGCUUCCUGGGACGUGUGAUGCUGUGCUUCCUGGGAGGUGUGAUCCCACACU
C
>hsa-mir-3690-1 MI0016091
CCCAUCUCCACCUGGACCCAGCGUAGACAAAGAGGUGUUUCUACUCCAUAUCUACCUGGACCCAG
UGUAGAUGGG
>hsa-mir-3691 MI0016092
UUGAGGCACUGGGUAGUGGAUGAUGGAGACUCGGUACCCACUGCUGAGGGUGGGGACCAAGUCUG
CGUCAUCCUCUCCUCAGUGCCUCAA
>hsa-mir-3692 MI0016093
CCAUUCCUGCUGGUCAGGAGUGGAUACUGGAGCAAUAGAUACAGUUCCACACUGACACUGCAGAA
GUGG
>hsa-mir-3713 MI0016134
GGUAUCCGUUUGGGGAUGGUUUCACUAUCCCCAGAUGGAUACCAA
>hsa-mir-3714 MI0016135
GAAGGCAGCAGUGCUCCCCUGUGACGUGCUCCAUCACCGGGCAGGGAAGACACCGCUGCCACCUC
>hsa-mir-3907 MI0016410
GGGUUGGAAAGCUGUAGGUGUGGAGGGGCAUGGAUACGGGGGCCAUGAGGGUGGGGUCCAGGCUG
GACCAGGCCUGCCCUGAGUCCCCCAGCAGGUGCUCCAGGCUGGCUCACACCCUCUGCCUCUCUCU
CUUCCUUCCUGGCCCCAACCC
>hsa-mir-3908 MI0016412
GCCUGAGCAAUGUAGGUAGACUGUUUCUAAAAAAAUAAAAAGUUAAAAAAAUUUAUGUUAACGUG
UAAUGUGUUUACUAAUUUUUUUUUUUUUUUUUGGAGACAGAGUCUCCCUCUGUCGCCAGGC
>hsa-mir-3909 MI0016413
GGUAUGCUGUUGCGCUGUCCUUCCUCUGGGGAGCAGGCUCCGGGGGACAGGGAAAGCACACAAG
GAACUUGUCCUCUAGGGCCUGCAGUCUCAUGGGAGAGUGACAUGCACCAGGACC
>hsa-mir-3910-1 MI0016414
CUUUUGCUGUCAGUUUUUCUGUUGCUUGUCUUGGUUUUAUGCCUUUUAUAUCAAGGCACAUAAAA

GGCAUAAAACCAAGACAAGCAACAAAAAAAGGAUUGAUCACAGAAG
>hsa-mir-3911 MI0016415
GGGUGAGGAUGUGUGUGGAUCCUGGAGGAGGCAGAGAAGACAGUGAGCUUGCCAGUUCUGGUUUC
CAACACUUCCUUUCCUGCGCUUCUCGAUUCCCAGAUCUGCACCC
>hsa-mir-3912 MI0016416
AGAGAGGAAUGAACAGUUAAAUUAUAACAUGUCCAUAUUAUGGGUUAGUUGUGGACACAUACUAA
CGCAUAAUAUGGACAUGUUAUAAUUUAACUGUUCCUUUCU
>hsa-mir-3913-1 MI0016417
UUGUUUAUAAUAAACUGAAAUAUUUGGGACUGAUCUUGAUGUCUGCCAAAACCUUGGCAGACAUC
AAGAUCAGUCCCAAAUAUUUCAGUUUAUUAUAGACAG
>hsa-mir-3914-1 MI0016419
UGGACUUCAGAUUUAACUUCUCAUUUUCUGGUUCCUUCUAAUGAGUAUGCUUAACUUGGUAGAAG
GAACCAGAAAAUGAGAAGUUGAGUAGGAACUCUA
>hsa-mir-3915 MI0016420
CAAGUUGGCACUGUAGAAUAUUGAGGAAAAGAUGGUCUUAUUGCAAAGAUUUUCAAUAAGACCAU
CCUUUCCUCAAUAUUCUGUGGUGUCAUCUUUG
>hsa-mir-3916 MI0016422
AUCCCAGAGAAGAAGGAAGAAGAGGAAGAAAUGGCUGGUUCUCAGGUGAAUGUGUCUGGGUUCAG
GGGAUGUGUCUCCUCUUUUCUUCUGGGAU
>hsa-mir-3917 MI0016423
GGCGCUUUUGUGCGCGCCCGGGUCUGUUGGUGCUCAGAGUGUGGUCAGGCGGCUCGGACUGAGCA
GGUGGGUGCGGGGCUCGGAGGAGGCGGC
>hsa-mir-3918 MI0016424
AGGCGGUUAAGCCAUGGGACAGGGCCGCAGAUGGAGACUGCUCAAGGUCAAAGGGGUCUCCAGCU
GGGACCCUGCACCUGGUUCGUAGCCCCU
>hsa-mir-3919 MI0016425
CCUGAGCACCAUUUACUGAGUCCUUUGUUCUCUACUAGUUUGUAGUAGUUCGUAGCAGAGAACAA
AGGACUCAGUAAAUGGUGCUCAGG
>hsa-mir-3920 MI0016427
ACUGAGUGAGGGAGUCAGAGAGUUAAGAGAAUUAGUACAGGUGAGAUUGUACUGAUUAUCUUAAC
UCUCUGACCCCCUCACUCAGU
>hsa-mir-3921 MI0016428
CCUAGCCCAGUACAAGGCAUAUGGUACUCAAGAGACUUAGAAAUCCCUAAGUCUCUGAGUACCAU
AUGCCUUGUACUGGGCUAGG
>hsa-mir-3922 MI0016429
GGAAGAGUCAAGUCAAGGCCAGAGGUCCCACAGCAGGGCUGGAAAGCACACCUGUGGGACUUCUG
GCCUUGACUUGACUCUUUC
>hsa-mir-3923 MI0016430
GGUAGAGUGAGCUCUAAUCCAAUAUUACUAGCUUCUUUAUAAGAAGAGGAAACUAGUAAUGUUGG
AUUAGGGCUCACUCUACU
>hsa-mir-3924 MI0016432
UAAAUGAAAAAGUAGUAGUCAAAUAUGCAGAUCUAUGUCAUAUAUACAGAUAUGUAUAUGUGACU
GCUACUUUUUUGUUUA
>hsa-mir-3925 MI0016433

GUGGGAAUAGCAAGAGAACUGAAAGUGGAGCCUGUCACAUCUCCAGACUCCAGUUUUAGUUCUCU
UGCUAUUUCCAC
>hsa-mir-3926-1 MI0016434
AAAAUGGAGCUGGCCAAAAAGCAGGCAGAGACUUUAAAAGCGUCUCUGCCUGCUUUUUGGCCAGC
UCCGUUUU
>hsa-mir-3927 MI0016435
UGCCAAUGCCUAUCACAUAUCUGCCUGUCCUAUGACAAACAUGGCAGGUAGAUAUUUGAUAGGCA
UUGGCA
>hsa-mir-3928 MI0016438
GCUGAAGCUCUAAGGUUCCGCCUGCGGGCAGGAAGCGGAGGAACCUUGGAGCUUCGGC
>hsa-mir-3929 MI0016439
AGUGGCUCACACCAGUAAUCCCAGCACUUUGGGAGGCUGAUGUGAGUAGACCACU
>hsa-mir-3934 MI0016590
CACAGCCCUUCCUGUCCCCAGUUUUCAGGUGUGGAAACUGAGGCAGGAGGCAGUGAAGUAACUUG
CUCAGGUUGCACAGCUGGGAAGUGGAGCAGGGAUUUGAAUCC
>hsa-mir-3935 MI0016591
GGAUGUGUUCCUGUCCCAGAAGGAGCUGAUGGUUGUAUCUAUGAAGGUAAGCAUUUUUGUAGAUA
CGAGCACCAGCCACCCUAAGCAAAGGCAGAGAAUGCUUA
>hsa-mir-3936 MI0016592
AUGAUUCAGAGCAUCUGUCCAGUGUCUGCUGUAGAUCCCUCAAAUCCGUGUUUGGACGCUUCUGG
UAAGGGGUGUAUGGCAGAUGCACCCGACAGAUGCACUUGGCAGCA
>hsa-mir-3937 MI0016593
AGAAGAAUGCCCAACCAGCCCUCAGUUGCUACAGUUCCCUGUUGUUUCAGCUCGACAACAACAGG
CGGCUGUAGCAAUGGGGGGCUGGAUGGGCAUCUCAAUGUGC
>hsa-mir-3938 MI0016594
AGGAAUUUUUAACCCGAUCACUAGAUUAUCUACAAGGGAAUUUUUUUUAAUUUAAAAAAUUCCC
UUGUAGAUAACCCGGUGGUCAGGUUGGAUGGCUCCAUG
>hsa-mir-3939 MI0016596
CUGGCUUCCAAAGGCCUCUGUGUGUUCCUGUAUGUGGGCGUGCACGUACCUGUCACAUGUGUACG
CGCAGACCACAGGAUGUCCACACUGGCUUCCAAACACAUCU
>hsa-mir-3940 MI0016597
GCUUAUCGAGGAAAAGAUCGAGGUGGGUUGGGGCGGGCUCUGGGGAUUUGGUCUCACAGCCCGGA
UCCCAGCCCACUUACCUUGGUUACUCUCCUUCCUUCU
>hsa-mir-3941 MI0016598
GAGUCAGAAUUCUCAUCAGGCUGUGAUGCUCAGUUGUGUGUAGAUUGAAAGCCCUAAUUUUACAC
ACAACUGAGGAUCAUAGCCUGAUGGUUCCUUUUUGUUU
>hsa-mir-3942 MI0016599
UCUUCAGUAUGACACCUCAAAGAAGCAAUACUGUUACCUGAAAUAGGCUGCGAAGAUAACAGUAU
UUCAGAUAACAGUAUUACAUCUUUGAAGUGUCAUAUUCACUGAC
>hsa-mir-3943 MI0016600
CACACAGACGGCAGCUGCGGCCUAGCCCCCAGGCUUCACUUGGCGUGGACAACUUGCUAAGUAAA
GUGGGGGGUGGGCCACGGCUGGCUCCUACCUGGAC
>hsa-mir-3944 MI0016601
UCCACCCAGCAGGCGCAGGUCCUGUGCAGCAGGCCAACCGAGAAGCGCCUGCGUCUCCCAUUUUC

GGGCUGGCCUGCUGCUCCGGACCUGUGCCUGAUCUUAAUGCUG
>hsa-mir-3945 MI0016602
GAUGUUGAUGCACGUGACGGGGAGGGCAUAGGAGAGGGUUGAUAUAAAAUGCAAUUACAGCCUCU
UAUGCUUUCCAAAGUGGGGGAUGAUUCAAUGAU
>hsa-mir-3960 MI0016964
GGCGCCCCGGCUCCCCGCGCCCCCGAUCGGGGCCGCCGCUAGUAGUGGCGGCGGCGGAGGCGGGG
GCAGCGGCGGCGGCGGCGGAGGCGCC
>hsa-mir-3972 MI0016990
GCCCAUUUGCCUUGGCUUGGGGUGGCAGUCCUGUGGGAAUGAGAGAUGCCAAACUGGACCUGCCA
GCCCCGUUCCAGGGCACAGCAU
>hsa-mir-3973 MI0016991
GCCCAGGGUAGCUCUCUGUAUUGCUUGUUACAUUUUAGGAUUGCUUGCCCCUUGCUCCAAUGGUG
CAGGCAGAAGAAAUGCAAACAAAGUACAGCAUUAGCCUUAGC
>hsa-mir-3974 MI0016992
GUUCAGGGAAAAGGUCAUUGUAAGGUUAAUGCACCCAUAUUUUAAUAUCAAACUAUGACAAAUUU
GACUACAGCCUUUCCGUACCCCUGCCAAAAC
>hsa-mir-3975 MI0016993
CAUGUAGGUGAGUGAUUGCUAUUUCAAAAGACUAGAGGUAGGAAAUGAGGCUAAUGCACUACUUC
ACAUG
>hsa-mir-3976 MI0016994
UGAGGGAUAUAGAGAGCAGGAAGAUUAAUGUCAUAUUGGAGUUGGACUGCAGGGCUUCCUUUACA
CAAUAAAUAUUGUAUGAAGUGCUGAUGUAACCUUUACUGCAGCAUGACAUGGGAUUUGGCUGUUU
UUAUGGCUC
>hsa-mir-3977 MI0016995
UUGUGCUUCAUCGUAAUUAACCUUAAGUGGUUCGGGUAAAUCACUUUAAUUUGUUAUGUGUUGGC
AGAAU
>hsa-mir-3978 MI0016996
UCAGUGGAAAGCAUGCAUCCAGGGUGUGGAGCCAAAAUUAGAAGGGCCAAAAUUCUACCUGGCCC
ACUACCACAGCAACCUUGGGCAUCGUUUUCUUUUGA
>hsa-mir-4251 MI0015861
CACGUCCUCCAGCUUUUUUCCUUAGUGGCCAAUUCCUGAGAAAAGGGCCAACGUGCUUCCA
>hsa-mir-4252 MI0015864
UGGGGGGCUGGCAGCUCAUCAGUCCAGGCCAUCUGGCCACUGAGUCAGCACCAGCGCCCAAUC
>hsa-mir-4253 MI0015860
CCAGCCAUCGCCCUUGAGGGGCCCUAGGACUUACUUGUGCAGGGCAUGUCCAGGGGGUCCAGGUC
UGC
>hsa-mir-4254 MI0015862
CUUGGGAGGAGGGUGGGGUGGCUCCUCUGCAGUGAGUAGGUCUGCCUGGAGCUACUCCACCAUCU
CCCCCAGCCCC
>hsa-mir-4255 MI0015863
GAGCAUCCUUCAGUGUUCAGAGAUGGAGUCAGUAUUGGUCUGGCCAUUUUUAGGGCAAAGAGGCA
GCAUCAU
>hsa-mir-4256 MI0015855
UGUUCCAUUUAUCUGACCUGAUGAAGGUCUCCUGGCAUUGAUUAGGUCUGAUGAUCCAUUUCUG

>hsa-mir-4257 MI0015856
GGCUUAGAAACAGUCCCUAGGUAGGAUUUGGGGAGGAGCUAAGAAGCCCCUACAGGGCCCAGAGG
UGGGGACUGAGCCUUAGUUGG
>hsa-mir-4258 MI0015857
ACGCCCCCCGCCCCGCCACCGCCUUGGAGGCUGACCUCUUACUUUCGGUCGGUCUUCUUCCCUGG
GCUUGGUUUGGGGGCGGGGGAGUGUC
>hsa-mir-4259 MI0015858
GAUGGGCCCCUUGUGUCCUGAAUUGGGUGGGGGCUCUGAGUGGGGAAAGUGGGGGCCUAGGGGAG
GUCACAGUUGGGUCUAGGGGUCAGGAGGGCCCAGGA
>hsa-mir-4260 MI0015859
AACAAGGUGACUUGGGGCAUGGAGUCCCACUUCCUGGAGCCCACACCCCAGCUUGUCACACACCA
AC
>hsa-mir-4261 MI0015868
GGUGGAAGUGGGUUCCUCCCAGUUCCUGAGACAGGAAACAGGGACCCAGGAGACCAGC
>hsa-mir-4262 MI0015872
GAAAGCUGCAGGUGCUGAUGUUGGGGGGACAUUCAGACUACCUGCAGCAGAGCC
>hsa-mir-4263 MI0015876
AUAGUGCUCUUCAGGGUUUUACUUGGGAGAUUGGAGUGGCCAGUGUUCCUAAACAAUUCUAAGUG
CCUUGGCCCACAACAUAC
>hsa-mir-4264 MI0015877
AAAGCUGGAUACUCAGUCAUGGUCAUUGUAACAUGAUAGUGACAGGUACUGGGUAAGACUGCAUA
G
>hsa-mir-4265 MI0015869
UGCAGUGGGUUGGAGCUUCAGCCUACACCUGUAAAGAAUUGGUCAGCCUGGGGACUGGUGAUCUC
UGCAGCUGUGGGCUCAGCUCUGGGCUGGGCCUGG
>hsa-mir-4266 MI0015870
CCACUGCUGGCCGGGGCCCCUACUCAAGGCUAGGAGGCCUUGGCCAAGGACAGUC
>hsa-mir-4267 MI0015871
CUCAGCAGGCUCCAGCUCGGUGGCACUGGGGGAAGGCUCCAGACCCCAGCCUCUGUCAUCCCUGC
AUGGAGCCCACAUCUCC
>hsa-mir-4268 MI0015874
AUGCACAUCAGGUUCUAGAGGUUUUGCCCUAGCGGCUCCUCCUCUCAGGAUGUGAUGUCACCUG
>hsa-mir-4269 MI0015875
ACAGCGCCCUGCAGGCACAGACAGCCCUGGCUUCUGCCUCUUUCUUUGUGGAAGCCACUCUGUCA
GGCCUGGGAUGGAGGGGCA
>hsa-mir-4270 MI0015878
ACAAAUAGCUUCAGGGAGUCAGGGGAGGGCAGAAAUAGAUGGCCUUCCCCUGCUGGGAAGAAAGU
GGGUC
>hsa-mir-4271 MI0015879
AAAUCUCUCUCCAUAUCUUUCCUGCAGCCCCCAGGUGGGGGGGAAGAAAAGGUGGGGAAUUAGAU
UC
>hsa-mir-4272 MI0015880
UUUUCUGCACAAAUUAAUCAGUUAAUGCAUAGAAAGCAUUCAACUAGUGAUUGUGUUAUAAGAG
>hsa-mir-4273 MI0015881

UCCCCUGUGUGUGUUCUCUGAUGGACAGUAAGCCUUGACUUAUGGCUAAAUGCUUCUUCACAAUG
GUCACAUGCAUAGGGCUUU
>hsa-mir-4274 MI0015884
GGGGCAUUUAGGGUAACUGAGCUGCUGCCGGGGCCUGGCGCUCCUCUACCUUGUCAGGUGACCCA
GCAGUCCCUCCCCCUGCAUGGUGCCC
>hsa-mir-4275 MI0015883
ACAUUUUUGUCCAAUUACCACUUCUUUUUGCCACCUGAGCACAGUCAGCAGUCAGCAUAAAAAAG
UGAUAAUGGGAAGUUAAUGUCU
>hsa-mir-4276 MI0015882
CACAGUCUGACUCAGUGACUCAUGUGCUGGCAGUGGCCACGUAAAUAGAGCUACUGUGUCUGAAA
GCAAU
>hsa-mir-4277 MI0015886
CUGGGUCGAGGCAGUUCUGAGCACAGUACACUGGGCUGCCCCCACUGCCCAGUGCCCUGCUCAGC
UCAAGUCCUUGUGCCCCUC
>hsa-mir-4278 MI0015888
AUCUAACACCAGGAGAAUCCCAUAGAACAUUGACAUCAACACUAGGGGGUUUGCCCUUGUGGGGA
AGAA
>hsa-mir-4279 MI0015887
UGCUCUGUGGAGCUGAGGAGCAGAUUCUCUCUCUCUCCUCCCGGCUUCACCUCCUGAG
>hsa-mir-4280 MI0015889
AAUCAGGGUGGAGUGUAGUUCUGAGCAGAGCCUUAAAGGAUGAGGUAUGUUCAAGACUGAAUGAC
ACCUUUGUGAU
>hsa-mir-4281 MI0015885
GCUGGGGGUCCCCCGACAGUGUGGAGCUGGGGCCGGGUCCCGGGGAGGGGGGUUCUGGGCAG
>hsa-mir-4282 MI0015890
GGUGAAGUUCCAGGGGAAGAUUUUAGUAUGCCACAUUUCUAAAAUUUGCAUCCAGGAACAUCAUC
CU
>hsa-mir-4283-1 MI0015892
ACUCUGAUCCUGGGGCUCAGCGAGUUUGCAAGGGGUGUUUCUGUCCAUGGUCAGGCUUGCCAGCC
UUGGUCCUUGGGCCC
>hsa-mir-4284 MI0015893
GUUCUGUGAGGGGCUCACAUCACCCCAUCAAAGUGGGGACUCAUGGGGAGAGGGGGUAGUUAGGA
GCUUUGAUAGAGGCGG
>hsa-mir-4285 MI0015891
AUUAGCUGGGGCGGCGAGUCCGACUCAUCAAUAUUUUAAGGAAUGACCCGGCCUUGGGGUGCGGA
AUUGCUGCGCGGGCGGGGGC
>hsa-mir-4286 MI0015894
UACUUAUGGCACCCCACUCCUGGUACCAUAGUCAUAAGUUAGGAGAUGUUAGAGCUGUGAGUACC
AUGACUUAAGUGUGGUGGCUUAAACAUG
>hsa-mir-4287 MI0015895
UAGUUCUUUUUCUCCCUUGAGGGCACUUUUCAGUUCCUGAGAUCAAUGUGGUCCCUACUGGGGAG
ACCAUAGGAGCCC
>hsa-mir-4288 MI0015896
AUGGAGGUGGAGAGUCAUCAGCAGCACUGAGCAGGCAGUGUUGUCUGCUGAGUUUCCACGUCAUU

UG
>hsa-mir-4289 MI0015898
CCUUGGGAGGGCAUUGUGCAGGGCUAUCAGGCAGUUUCCUGGGCCCUGUCUGCAGAGCCUAAACA
GAUCA
>hsa-mir-4290 MI0015899
GCCACCAAGAAGGUGAAGGGAGGGUCAGUCCCAAUCUGAAUCCCACCAAAAUAGGUGGUAGAGGG
UUGCCCUCCUUUCUUCCCUCACCUCUGACC
>hsa-mir-4291 MI0015900
CGCCGGGGGCUUCAGCAGGAACAGCUGGGUGGAGGCAGAGCUGUUCUGCUGUGGCUGCAGCCCUG
>hsa-mir-4292 MI0015897
GAGACACCAGAAGGCCACCUGCUUAGGAGGCCAGAGGUGCCCCUGGGCCGGCCUUGGUGAGGGGC
CC
>hsa-mir-4293 MI0015826
AGAGACACCUGUUCCUUGGGAAGCUGGUGACAUUGCUAAUUCAUUUCACACCAGCCUGACAGGAA
CAGCCUGACUGAA
>hsa-mir-4294 MI0015827
CCGAUGCCUCGGGAGUCUACAGCAGGGCCAUGUCUGUGAGGGCCCAAGGGUGCAUGUGUCUCCCA
GGUUUCGGUGC
>hsa-mir-4295 MI0015822
CUUUGUGGAACAGUGCAAUGUUUUCCUUGCCUGUGGCAAGACCACUUCGGUUCAAGGCUAAGAAA
CUAGACUGUUCCUACAGAGA
>hsa-mir-4296 MI0015823
UUGGGCUUUGAUGUGGGCUCAGGCUCAGAGGGCUGAAGUGGUUGUGGGGAGGGGCUUCUGGGGAC
UGUGUCCAUGUCUCUGUCGUUUU
>hsa-mir-4297 MI0015824
AGCACGCACGUGCCUUCCUGUCUGUGCCUGCCUUCGAAGUGCACGGCAGGGCCAGGACGGGUCGC
UGUGGGUGGGG
>hsa-mir-4298 MI0015830
GGGGAGGUACCUGGGACAGGAGGAGGAGGCAGCCUUGCCUCAGAAACCAAACUGUCAAAAGUGUA
GGUUCCAC
>hsa-mir-4299 MI0015829
GGGUUCUGACCAAUCAUGUUACAGUGUUUUCUCCUUUAGAGAGAGCUGGUGACAUGAGAGGCAGA
AAAAGGA
>hsa-mir-4300 MI0015831
UGAGUUUAGAAGAGGGCCAGCUAAAUCAGCAGAGACAUGAGGUGAUCAAAAACCUUUUUUCAAAG
CAGUGGGAGCUGGACUACUUCUGAACCAAUA
>hsa-mir-4301 MI0015828
ACCAGCCACCUCCCACUACUUCACUUGUGAACAUUGCAUUCGUGGAGGGUGGCAGGUGCAGCUCU
G
>hsa-mir-4302 MI0015833
UCAGGAGGGACCAGUGUGGCUCAGCGAGGUGGCUGAGUUUACUUAAGGUAUUGGAAUGAG
>hsa-mir-4303 MI0015834
AGAAAAUAGCUUCUGAGCUGAGGACAGCUUGCUCUGCUUUUCUUUAGCUUAGGAGCUAACCAUGG
U

>hsa-mir-4304 MI0015832
AGAGAAGUGGCCGGCAUGUCCAGGGCAUCCCCAUUGCUCUGUGACUGCUGCCAUCCUUCUCC
>hsa-mir-4305 MI0015835
CUGCCUUAGACCUAGACACCUCCAGUUCUGGGUUCUUAGAGGCCUAAUCCUCUACAAACUCAGUU
UUCAGACUGUGAGGGAAAUUCUCUGUCUUAUUGCUUU
>hsa-mir-4306 MI0015836
AAGCUGCUUAGUGUCCUUAGAGUCUCCAGAGGCAUCCCUAACCCAGAAUCUUUUGACUGUCCUCU
GGAGAGAAAGGCAGUAGGUCUGUACC
>hsa-mir-4307 MI0015838
UCAGAAGAAAAAACAGGAGAUAAAGUUUGUGAUAAUGUUUGUCUAUAUAGUUAUGAAUGUUUUUU
CCUGUUUCCUUCAGGGCCA
>hsa-mir-4308 MI0015839
UAUGGGUUCAGAGGGAACUCCAUUGGACAGAAAUUUCCUUUUGAGGAAAUCUUUCCCUGGAGUUU
CUUCUUACCUUUUUCC
>hsa-mir-4309 MI0015837
UCUGGGGGUUCUGGAGUCUAGGAUUCCAGGAUCUGGGUUUUGAGGUCUUGGGUUGUAGGGUCUGC
GGUUUGAAGCCCCUCUUG
>hsa-mir-4310 MI0015840
UGGCGUCUGGGGCCUGAGGCUGCAGAACAUUGCAGCAUUCAUGUCCCACCCCCACCA
>hsa-mir-4311 MI0015841
UCAGAGAGGGGAAAGAGAGCUGAGUGUGACCUGGAGCAGCUCAGGAGGGCUUCCUGGGUGAGGUG
GCAGGUUACAGGUUCGAUCUUUGGCCCUCAGAUUC
>hsa-mir-4312 MI0015842
GAAAGGUUGGGGGCACAGAGAGCAAGGAGCCUUCCCCAGAGGAGUCAGGCCUUGUUCCUGUCCCC
AUUCCUCAGAG
>hsa-mir-4313 MI0015843
GAUCAGGCCCAGCCCCCUGGCCCCAAACCCUGCAGCCCCAGCUGGAGGAUGAGGAGAUGCUGGGC
UUGGGUGGGGGAAUCAGGGGUGUAAAGGGGCCUGCU
>hsa-mir-4314 MI0015846
GGCCAUUCCUCUCUGGGAAAUGGGACAGGUAGUGGCCACAGUGAGAAAGCUGGCCUGUCCUUCUG
CCCCAGGGCCCAGAGUCUGUGACUGGA
>hsa-mir-4315-1 MI0015844
UGGGCUUUGCCCGCUUUCUGAGCUGGACCCUCUCUCUACCUCUGGUGCAGAACUACAGCGGAAGG
AAUCUCUG
>hsa-mir-4316 MI0015845
AGUGGCCCAGGGUGAGGCUAGCUGGUGUGGUCACCCACUCUCCAGCCCAGCCCCAAUCCCACCAC
AACCAC
>hsa-mir-4317 MI0015850
AAAAGGCGAGACAUUGCCAGGGAGUUUAUUUUGUAGCUCUCUUGAUAAAAUGUUUUAGCAAACAC
>hsa-mir-4318 MI0015847
GCUUCUUAAUUAUGUCAUAAACCCACUGUGGACAAGGGCCUUGUCUUAGACAGUCACUGUGGGUA
CAUGCUAGGUGCUCAA
>hsa-mir-4319 MI0015848
UUGGCUUGAGUCCCUGAGCAAAGCCACUGGGAAUGCUCCCUGAGGACGUUAUAUGAGUGCUCAGC

UCAUGGGGCUAUGAUGGUCA
>hsa-mir-4320 MI0015849
GACAUGUGGGGUUUGCUGUAGACAUUUCAGAUAACUCGGGAUUCUGUAGCUUCCUGGCAACUUUG
>hsa-mir-4321 MI0015852
CUGGUCUCCGCAGAGCCUCUGCCCCUCCCGAGACACCCGCUACCUGGUGUUAGCGGUGGACCGCC
CUGCGGGGGCCUGGC
>hsa-mir-4322 MI0015851
ACCGCGAGUUCCGCGCCUGGCCGUGUCGCCCCACGAGGGGGACUGUGGGCUCAGCGCGUGGGGCC
CGGAGCAU
>hsa-mir-4323 MI0015853
CGGGGCCCAGGCGGGCAUGUGGGGUGUCUGGAGACGCCAGGCAGCCCCACAGCCUCAGACCUCGG
GCAC
>hsa-mir-4324 MI0015854
CGGCCCCUUUGUUAAGGGUCUCAGCUCCAGGGAACUUUAAAACCCUGAGACCCUAACCUUAAAGG
UGCUGCA
>hsa-mir-4325 MI0015865
GGGGAAGAUGUUGCACUUGUCUCAGUGAGAGAUGCUUCUAGAUCCAGGAGGCAGACCUCAAGGAU
GGAGAGAAGGCAGAUCCUUUGAGAU
>hsa-mir-4326 MI0015866
GCUGCUCUGCUGUUCCUCUGUCUCCCAGACUCUGGGUGGAUGGAGCAGGUCGGGGGCCA
>hsa-mir-4327 MI0015867
GGCCUGGGUAGGCUUGCAUGGGGGACUGGGAAGAGACCAUGAACAGGUUAGUCCAGGGAGUUCUC
AUCAAGCCUUUACUCAGUAG
>hsa-mir-4328 MI0015904
AACAGUUGAGUCCUGAGAACCAUUGAGAACCAGUUUUCCCAGGAUUAACUGUUCCG
>hsa-mir-4329 MI0015901
UAGAGAGGAAGGUGUACCAGGGUUUUGGAGUUUUUUUUCCUCCUGAGACCCUAGUUCCACAUUC
UGGAGC
>hsa-mir-4330 MI0015902
AAUUGUCAGCAGGCAAUUAUCUGAGGAUGCAGGAGAGGAAGGGGGCUUCUUUUUGACGCCUACUU
CAUCAGCUGCUCCUCAGAUCAGAGCCUUGCAGGUCAGGCC
>hsa-mir-4417 MI0016753
GAAAACAACCAGGUGGGCUUCCCGGAGGGCGGAACACCCAGCCCCAGCAUCCAGGGCUCACCUAC
CACGUUUG
>hsa-mir-4418 MI0016754
UGGUUUUUGCUCUGAGUGACCGUGGUGGUUGUGGGAGUCACUGCAGGACUCAGCAGGAAUUC
>hsa-mir-4419a MI0016755
UGGUGGUGUGUGCCUGUAGUCUUAGCUACUCGGGAGGCUGAGGGAGGAGACUGCAGUGAGUGGAG
GUCACGCCACUG
>hsa-mir-4419b MI0016861
CUCAGGCUCAGUGGUGCAUGCUUAUAGUCCCAGCCACUCUGGAGGCUGAAGGAAGAUGGCUUGAG
CCU
>hsa-mir-4420 MI0016757
CUCUUGGUAUGAACAUCUGUGUGUUCAUGUCUCUCUGUGCACAGGGGACGAGAGUCACUGAUGUC

UGUAGCUGAGAC
>hsa-mir-4421 MI0016758
CUGGGUCUCCUUUCUGCUGAGAGUUGAACACUUGUUGGGACAACCUGUCUGUGGAAAGGAGCUAC
CUAC
>hsa-mir-4422 MI0016759
AGUUCUUCUGCAGACAAAAGCAUCAGGAAGUACCCACCAUGUACCAGUGGGCCCUUCUUGAUGCU
CUUGAUUGCAGAGGAGCC
>hsa-mir-4423 MI0016760
AUCAUGUACUGCAGUUGCCUUUUUGUUCCCAUGCUGUUUAAGCCUAGCAUAGGCACCAAAAAGCA
ACAACAGUAUGUGAA
>hsa-mir-4424 MI0016763
CUUACAUCACACACAGAGUUAACUCAAAAUGGACUAAUUUUUCCACUAGUUAGUCCAUUUCAAGU
UAACUCUGUGUGUGAUGUAGU
>hsa-mir-4425 MI0016764
GUGCUUUACAUGAAUGGUCCCAUUGAAUCCCAACAGCUUUGCGAAGUGUUGUUGGGAUUCAGCAG
GACCAUUCGUGUAAAGUAA
>hsa-mir-4426 MI0016765
AGUUGGAAGAUGGACGUACUUUGUCUGACUACAAUAUUCAAAAGGAGUCUACUCUUCAUCUUG
>hsa-mir-4427 MI0016766
GAAGCCUCUUGGGGCUUAUUUAGACAAUGGUUUCAUCAUUUCGUCUGAAUAGAGUCUGAAGAGUC
UUU
>hsa-mir-4428 MI0016767
UUGGCAGGUGCCAUGUUGCCUGCUCCUUACUGUACACGUGGCUGGCAAGGAGACGGGAACAUGGA
GCCGCCAU
>hsa-mir-4429 MI0016768
AGGGAGAAAAGCUGGGCUGAGAGGCGACUGGUGUCUAAUUUGUUUGUCUCUCCAACUCAGACUGC
CUGGCCCA
>hsa-mir-4431 MI0016771
UGGUUUGCGACUCUGAAAACUAGAAGGUUUAUGACUGGGCAUUUCUCACCCAAUGCCCAAUAUUG
AACUUUCUAGUUGUCAGAGUCAUUAACCC
>hsa-mir-4432 MI0016772
GCAUCUUGCAGAGCCGUUCCAAUGCGACACCUCUAGAGUGUCAUCCCCUAGAAUGUCACCUUGGA
AAGACUCUGCAAGAUGCCU
>hsa-mir-4433b MI0025511
UGUGUUCCCUAUCCUCCUUAUGUCCCACCCCCACUCCUGUUUGAAUAUUUCACCAGAAACAGGAG
UGGGGGGUGGGACGUAAGGAGGAUGGGGGAAAGAACA
>hsa-mir-4434 MI0016774
UCACUUUAGGAGAAGUAAAGUAGAACUUUGGUUUUCAACUUUUCCUACAGUGU
>hsa-mir-4435-1 MI0016775
AGGCAGCAAAUGGCCAGAGCUCACACAGAGGGAUGAGUGCACUUCACCUGCAGUGUGACUCAGCA
GGCCAACAGAUGCUA
>hsa-mir-4436a MI0016776
GCCUCACUUUUCCACUUAUGCCUGCCCUGCCCCUCGAAUCUGCUCCACGAUUUGGGCAGGACAGG
CAGAAGUGGAUAAGUGAGGA

>hsa-mir-4436b-1 MI0017425
GUGUCCUCACUUGUCCACUUCUGCCUGCCCUGCCCAAAUGGUGGAGCAGAUUCGAGGGGCAGGGC
AGGAAGAAGUGGACAAGUGAGGCCAU
>hsa-mir-4437 MI0016778
ACUUUGUGCAUUGGGUCCACAAGGAGGGGAUGACCCUUGUGGGCUCAGGGUACAAAGGUU
>hsa-mir-4438 MI0016781
UAAGUGUAAACUUAAGGACUGUCUUUUCUAAGCCUGUGCCUUGCCUUUCCUUUGGCACAGGCUUA
GAAAAGACAGUCUUUAAGUUUACACUUC
>hsa-mir-4439 MI0016782
CCAGUGACUGAUACCUUGGAGGCAUUUUAUCUAAGAUACACACAAAGCAAAUGCCUCUAAGGUAU
CAGUUUACCAGGCCA
>hsa-mir-4440 MI0016783
CUCUCACCAAGCAAGUGCAGUGGGGCUUGCUGGCUUGCACCGUGACUCCCUCUCACCAAGCAAGU
GUCGUGGGGCUUGCUGGCUUGCACUGUGAAGAU
>hsa-mir-4441 MI0016784
CAGAGUCUCCUUCGUGUACAGGGAGGAGACUGUACGUGAGAGAUAGUCAGAUCCGCAUGUUAGAG
CAGAGUCUCCUUCGUGUACAGGGAGGAGAUUGUAC
>hsa-mir-4442 MI0016785
GCGCCCUCCCUCUCUCCCCGGUGUGCAAAUGUGUGUGUGCGGUGUUAUGCCGGACAAGAGGGAGG
UG
>hsa-mir-4443 MI0016786
GGUGGGGGUUGGAGGCGUGGGUUUUAGAACCUAUCCCUUUCUAGCCCUGAGCA
>hsa-mir-4444-1 MI0016787
GUGACGACUGGCCCCGCCUCUUCCUCUCGGUCCCAUAUUGAACUCGAGUUGGAAGAGGCGAGUCC
GGUCUCAAA
>hsa-mir-4445 MI0016788
UUCCUGCAGAUUGUUUCUUUUGCCGUGCAAGUUUAAGUUUUUGCACGGCAAAAGAAACAAUCCAG
AGGGU
>hsa-mir-4446 MI0016789
CUGGUCCAUUUCCCUGCCAUUCCCUUGGCUUCAAUUUACUCCCAGGGCUGGCAGUGACAUGGGUC
AA
>hsa-mir-4447 MI0016790
GUUCUAGAGCAUGGUUUCUCAUCAUUUGCACUACUGAUACUUGGGGUCAGAUAAUUGUUUGUGGU
GGGGGCUGUUGUUUGCAUUGUAGGAU
>hsa-mir-4448 MI0016791
AGGAGUGACCAAAAGACAAGAGUGCGAGCCUUCUAUUAUGCCCAGACAGGGCCACCAGAGGGCUC
CUUGGUCUAGGGGUAAUGCCA
>hsa-mir-4449 MI0016792
AGCAGCCCUCGGCGGCCCGGGGGGCGGGCGGCGGUGCCCGUCCCGGGGCUGCGCGAGGCACAGGC
G
>hsa-mir-4450 MI0016795
UGUCUGGGGAUUUGGAGAAGUGGUGAGCGCAGGUCUUUGGCACCAUCUCCCCUGGUCCCUUGGCU
>hsa-mir-4451 MI0016797
UCUGUACCUCAGCUUUGCUCCCAACCAACCACUUCCACAUGUUUUGCUGGUAGAGCUGAGGACAG

C
>hsa-mir-4452 MI0016798
UGGAUCACUUGAGGCCAAGAGUGCAAGGCUGUAGUGUGCACAGCCUUGAAUUCUUGGCCUUAAGU
GAUCCC
>hsa-mir-4453 MI0016799
UGGAGAGCUUGGUCUGUAGCGGUUUCCUUCGGGGCAGGUGGGGACUGCUCCUUUGGGAGGAAGGA
GGAGGCCCAGGCCGCGUCUUCAGG
>hsa-mir-4454 MI0016800
CCGGAUCCGAGUCACGGCACCAAAUUUCAUGCGUGUCCGUGUGAAGAGACCACCA
>hsa-mir-4455 MI0016801
AGAAGGGUGUGUGUGUUUUUCCUGAGAAUAAGAGAAGGAAGGACAGCCAAAUUCUUCA
>hsa-mir-4456 MI0016802
AUGAACCUGGUGGCUUCCUUUUCUGGGAGGAAGUUAGGGUUCA
>hsa-mir-4457 MI0016803
GGAGUACUCCAGUCAAUACCGUGUGAGUUAGAAAAGCUCAAUUCACAAGGUAUUGACUGGCGUAU
UCA
>hsa-mir-4458 MI0016804
GAGCGCACAGAGGUAGGUGUGGAAGAAAGUGAAACACUAUUUUAGGUUUUAGUUACACUCUGCUG
UGGUGUGCUG
>hsa-mir-4459 MI0016805
ACCCAGGAGGCGGAGGAGGUGGAGGUUGCAGUGAGCCAAGAUCGUGGCACUGACUCCAGCCUGGG
G
>hsa-mir-4460 MI0016806
GUUUUUUGCCCAUAGUGGUUGUGAAUUUACCUUCUCCUCUUUGCAGUGAUAAAGGAGGUAAAUUC
ACAACCACUGUGGGCAGAAAC
>hsa-mir-4461 MI0016807
GAGUAGGCUUAGGUUAUGUACGUAGUCUAGGCCAUACGUGUUGGAGAUUGAGACUAGUAGGGCUA
GGCCUACUG
>hsa-mir-4462 MI0016810
CUUCCCAGCUGCCCUAAGUCAGGAGUGGCUUUCCUGACACGGAGGGUGGCUUGGGAAA
>hsa-mir-4463 MI0016811
AAUAGAUUAUUGGUCACCACCUCCAGUUUCUGAAUUUGUGAGACUGGGGUGGGGCCUGAGAAUUU
GC
>hsa-mir-4464 MI0016812
GGAACCUUAGUAAGGUUUGGAUAGAUGCAAUAAAGUAUGUCCACAGCUGAAAGGACAUACUUUAU
UGCAUGUAUCCAAACCUUACUAAUUCA
>hsa-mir-4465 MI0016816
CAUGUGUCCCCUGGCACGCUAUUUGAGGUUUACUAUGGAACCUCAAGUAGUCUGACCAGGGGACA
CAUGA
>hsa-mir-4466 MI0016817
ACGCGGGUGCGGGCCGGCGGGGUAGAAGCCACCCGGCCCGGCCCGGCCCGGCGA
>hsa-mir-4467 MI0016818
UGGUGGCGGCGGUAGUUAUGGGCUUCUCUUUCUCACCAGCAGCCCCUGGGCCGCCGCCUCCCU
>hsa-mir-4468 MI0016819

AGUCUUCUCCUGGGGCUUUGGUGGCUAUGGUUGACUGGGCCACUCAGAGCAGAAGGAUGAGAUG
>hsa-mir-4469 MI0016820
CCGACGCGGAGAGCGGCUCUAGGUGGGUUUGGCGGCGGCGAGGACACCGCCGCUCCCUCUAGGGU
CGCUCGGAGCGUGA
>hsa-mir-4470 MI0016821
CGAGCCUCUUUCGGCUUUCCAGUUUGUCUCGGUCCUUUGGAACGUGGCAAACGUGGAAGCCGAGA
GGGCUCU
>hsa-mir-4471 MI0016822
CCAAAUUUAAAACUUAAACCUCUACUAAGUUUCCAUGAAAAGAACCCAUGGGAACUUAGUAGAGG
UUUAAGUUUUAAAUUUGA
>hsa-mir-4472-1 MI0016823
UGGCAGACCCUUGCUCUCUCACUCUCCCUAAUGGGGCUGAAGACAGCUCAGGGGCAGGGUGGGGG
GUGUUGUUUUUGUUU
>hsa-mir-4472-2 MI0016824
UGGUGGGGGUGGGGGGUGUUGUUUUUGUUUUUGAGACAGAGUCUUGCUCCGUCGCCCAGGCCGGA
GU
>hsa-mir-4473 MI0016825
AAGGAACAGGGGACACUUGUAAUGGAGAACACUAAGCUAUGGACUGCUAUGGACUGCUAGUGCUC
UCCGUUACAAGUAUCCCCUGUUACCU
>hsa-mir-4474 MI0016826
UUGCCUACCUUGUUAGUCUCAUGAUCAGACACAAAUAUGGCUCUUUGUGGCUGGUCAUGAGGCUA
ACAAGGUAGGCAC
>hsa-mir-4475 MI0016827
AUCUCAAUGAGUGUGUGGUUCUAAAUGACUCAUAGUCAAGGGACCAAGCAUUCAUUAUGAA
>hsa-mir-4476 MI0016828
AAAAGCCUGUCCCUAAGUCCCUCCCAGCCUUCCAGAGUUGGUGCCAGGAAGGAUUUAGGGACAGG
CUUUG
>hsa-mir-4477a MI0016829
UCCUCCUCCCAUCAAUCACAAAUGUCCUUAAUGGCAUUUAAGGAUUGCUAUUAAGGACAUUUGUG
AUUCACGGGAGGAGGU
>hsa-mir-4478 MI0016831
GGCCGAGGCUGAGCUGAGGAGCCUCCAAACCUGUAGACAGGGUCAUGCAGUACUAGGGGCGAGCC
UCAUCCCCUGCAGCCCUGGCC
>hsa-mir-4479 MI0016838
GAAACCAAGUCCGAGCGUGGCUGGCGCGGGAAAGUUCGGGAACGCGCGCGGCCGUGCUCGGAGCA
GCGCCA
>hsa-mir-4480 MI0016841
GCAGAGGUGAGUUGACCUCCACAGGGCCACCCAGGGAGUAAGUAGCCAAGUGGAAGUUACUUUAC
CUCUGU
>hsa-mir-4481 MI0016842
GGAGUGGGCUGGUGGUUUUUUAAGAGGAAGGGAGACCUAAGCUAGCACAUGAGCACGCUC
>hsa-mir-4482 MI0016843
AGUGAGCAACCCAGUGGGCUAUGGAAAUGUGUGGAAGAUGGCAUUUCUAUUUCUCAGUGGGGCUC
UUACC

>hsa-mir-4483 MI0016844
AAAAAACAACAUACUUAGUGCAUACCCAUAUAAUAUUAGGGGUGGUCUGUUGUUGUUUUUCU
>hsa-mir-4484 MI0016845
GGGUUUCCUCUGCCUUUUUUUCCAAUGAAAAUAACGAAACCUGUUAUUUCCCAUUGAGGGGGAAA
AAGGCGGGAGAAGCCCCA
>hsa-mir-4485 MI0016846
AGAGGCACCGCCUGCCCAGUGACAUGCGUUUAACGGCCGCGGUACCCUAACUGUGCA
>hsa-mir-4486 MI0016847
GCAUGCUGGGCGAGGCUGGCAUCUAGCACAGGCGGUAGAUGCUUGCUCUUGCCAUUGCAAUGA
>hsa-mir-4487 MI0016848
ACUGUCCUUCAGCCAGAGCUGGCUGAAGGGCAGAAGGGAACUGUCCUUCAGCCAGAGCUGGCUGA
AGGGCAGA
>hsa-mir-4488 MI0016849
GGUAGGGGGCGGGCUCCGGCGCUGGGACCCCACUAGGGUGGCGCCUUGGCCCCGCCCCGCCC
>hsa-mir-4489 MI0016850
GGGGGUGGGGCUAGUGAUGCAGGACGCUGGGGACUGGAGAAGUCCUGCCUGACCCUGUCCCA
>hsa-mir-4490 MI0016852
AUAGUUUCUGCAAUGCUCAAAUCUCUGGCCAAAGACCAGAACUUAAUGGUCUCUGGUAAGAGAUU
UGGGCAUAUUAGAAACUAA
>hsa-mir-4491 MI0016853
ACAUUUGGUCACACCAGUCCACAUUAACGUGGACCAGACAAUAUUAAUGUGGACUGGUGUGACCA
AAA
>hsa-mir-4492 MI0016854
CUGCAGCGUGCUUCUCCAGGCCCCGCGCGCGGACAGACACACGGACAAGUCCCGCCAGGGGCUGG
GCGCGCGCCAGCCGG
>hsa-mir-4493 MI0016855
CCAGAGAUGGGAAGGCCUUCCGGUGAUUAUCACAGCCAUGCCUUUACCUCCAGAAGGCCUUUCCA
UCUCUGUC
>hsa-mir-4494 MI0016856
AGUUUUAGUUACCCUGGUCAUCUGCAGUCUGAAAAUACAAAAUGGAAAAUUCCAGACUGUGGCUG
ACCAGAGGUAACUGAAACC
>hsa-mir-4495 MI0016857
AAGAAAUGUAAACAGGCUUUUUGCUCAGUGGAGUUAUUUUGAGCAAAAAGCUUAUUUACAUUUCU
G
>hsa-mir-4496 MI0016858
ACAUCAGCUCAUAUAAUCCUCGAAGCUGCCUUUAGAAAUGAGGAAACUGAAGCUGAGAGGG
>hsa-mir-4497 MI0016859
ACCUCCGGGACGGCUGGGCGCCGGCGGCCGGGAGAUCCGCGCUUCCUGAAUCCCGGCCGGCCCGC
CCGGCGCCCGUCCGCCCGCGGGUC
>hsa-mir-4498 MI0016860
AGGGCUGGGCUGGCAGGGCAAGUGCUGCAGAUCUUUGUCUAAGCAGCCCCUGCCUUGGAUCUCCC
A
>hsa-mir-4499 MI0016862
AAGACUGAGAGGAGGGAACUGGUGAGUUGUACAUAGAAAUGCUUUCUAACUCCUUGUCUCAGUCU

GUUU
>hsa-mir-4500 MI0016863
CAGGAGAGAAAGUACUGCCCAGAAGCUAAAGUGUAGAUCAAACGCAUAAUGGCUGAGGUAGUAGU
UUCUUGAACUU
>hsa-mir-4501 MI0016864
UAUGUGACCUCGGAUGAAUCACUGAAAUAUGUCUGAGCUUCUGUUUCAUCAGAUGUCACAUUUU
>hsa-mir-4502 MI0016865
AGCCUUUAGCAAGUUGUAAUCUUUUUGCUGAUGGAGGGUCUUGCCUCCAUGGGGAUGGCUGAUGA
UGAUGGUGCUGAAGGC
>hsa-mir-4503 MI0016866
ACAAUGUAGAUAUUUAAGCAGGAAAUAGAAUUUACAUAUAAAUUUCUAUUUGUUUCUAUUUCCUG
CUUAAAUAUCUACAUUGC
>hsa-mir-4504 MI0016867
CUAAGAUAAUGUCCUCCAGGUUCAUCUCUGUUGUCAUUUGUGGCAUGGACCAUUUGUGACAAUAG
AGAUGAACAUGGAGGAUAUUAUCUUAA
>hsa-mir-4505 MI0016868
GGAGGCUGGGCUGGGACGGACACCCGGCCUCCACUUUCUGUGGCAGGUACCUCCUCCAUGUCGGC
CCGCCUUG
>hsa-mir-4506 MI0016869
UGGCCUCUGCCAUCAGACCAUCUGGGUUCAAGUUUGGCUCCAUCUUUAUGAAAUGGGUGGUCUGA
GGCAAGUGGUCU
>hsa-mir-4507 MI0016871
UCUGGGCUGAGCCGAGCUGGGUUAAGCCGAGCUGGGUUGGGCUGGGCUGGGU
>hsa-mir-4508 MI0016872
AGGACCCAGCGGGGCUGGGCGCGCGGAGCAGCGCUGGGUGCAGCGCCUGCGCCGGCAGCUGCAAG
GGCCG
>hsa-mir-4509-1 MI0016873
CUUUAAUACUAUCUCAAACUAAAGGAUAUAGAAGGUUUUCCCUUUCUCUUGCCCUGAAACCUUCU
GUAUCCUUUAUUUUGAGAUAGUAUUAGAA
>hsa-mir-4510 MI0016876
GUGUAUGUGAGGGAGUAGGAUGUAUGGUUGUUAGAUAGACAACUACAAUCUUUUCUCACAACAGA
CAG
>hsa-mir-4511 MI0016877
AAAAAAAAGGGAAAGAAGAACUGUUGCAUUUGCCCUGCACUCAGUUUGCACAGGGUAAAUGCAAU
AGUUCUUCUUUCCCUUUUUUUA
>hsa-mir-4512 MI0016878
CUCAGCCCGGGCAAUAUAGUGAGACCUCGUCUCUACAAAAAAUUGAGACAGGGCCUCACUGUAUC
GCCCAGGCUGGA
>hsa-mir-4513 MI0016879
AUUCUAGGUGGGGAGACUGACGGCUGGAGGCCCAUAAGCUGUCUAAAACUUCGGCCCCCAGAUUU
CUGGUCUCCCCACUUCAGAAC
>hsa-mir-4514 MI0016880
GUUGAGACAGGCAGGAUUGGGGAAACAUCUUUUACCUCGUCUCUUGCCUGUUUUAGA
>hsa-mir-4515 MI0016881

GCGGGAGGUGUAACAGGACUGGACUCCCGGCAGCCCCAGGGCAGGGGCGUGGGGAGCUGGUCCUA
GCUCAGCGCUCCCGGA
>hsa-mir-4516 MI0016882
AGGGAGAAGGGUCGGGGCAGGGAGGGCAGGGCAGGCUCUGGGGUGGGGGGUCUGUGAGUCAGCCA
CGGCUCUGCCCACGUCUCCCC
>hsa-mir-4517 MI0016883
AGGUAAAUAUGAUGAAACUCACAGCUGAGGAGCUUAGCAAGUAGCUAAGGCCAGAGCUUGUGUUU
GGGUGGUGUGGCUG
>hsa-mir-4518 MI0016884
UGGGGGAAAAGUGCUGGGAUUGAUUAGUGAUGUCUGCUGGGGAACCGGGGCUCAGGGAUGAUAAC
UGUGCUGAGAAGCCCCCU
>hsa-mir-4519 MI0016885
AACCUCAGCAGUGCGCAGGGCUGCACUGUCUCCGUCUGCGGCCUGCAGUAAGCGGGUA
>hsa-mir-4520a MI0016886
GUGUGCCACCUGCGUGUUUUCUGUCCAAAUCAGAAAAGGAUUUGGACAGAAAACACGCAGGAAGA
AGGAA
>hsa-mir-4521 MI0016887
UCGGCUAAGGAAGUCCUGUGCUCAGUUUUGUAGCAUCAAAACUAGGAUUUCUCUUGUUAC
>hsa-mir-4522 MI0016889
GCGGGCGUUGCCUGGGGGCCUCGCAGGGGGAGAUCCAGCCCAGGCUGGUUCCGCUGACUCUGCCU
GUAGGCCGGUGGCGUCUUCUGG
>hsa-mir-4523 MI0016890
GCGGGGGACCGAGAGGGCCUCGGCUGUGUGAGGACUAGAGGCGGCCGAGGCCCGGGCCGGUUCCC
CCGA
>hsa-mir-4524a MI0016891
GAACGAUAGCAGCAUGAACCUGUCUCACUGCAGAAUUAUUUUGAGACAGGCUUAUGCUGCUAUCC
UUCA
>hsa-mir-4524b MI0019114
UAGCUGGGUGGAUGUGUUCUUUUGAAGGAUAGCAGCAUAAGCCUGUCUCAAAAUAAUUCUGCAGU
GAGACAGGUUCAUGCUGCUAUCGUUCCAAAGAGGAAGGGUAAUCACUGUC
>hsa-mir-4525 MI0016892
GUCAGAGGGGGGAUGUGCAUGCUGGUUGGGGUGGGCUGCCUGUGGACCAAUCAGCGUGCACUUCC
CCACCCUGAA
>hsa-mir-4526 MI0016893
UGCGGUGACAUCAGGGCCCAGUCCCUGCUGUCAUGCCCCAGGUGACGUGCUGGGCUGACAGCAGG
GCUGGCCGCUAACGUCACUGUC
>hsa-mir-4527 MI0016894
CCAGAAGUGGUCUGCAAAGAGAUGACUGUGAAUCCAAGAUCCACAUCAGCUCUGUGCUGCCUACA
UCUGA
>hsa-mir-4528 MI0016895
UAUUCUACUGAGAGUACAGAUCUUUAUAUAUAUGAUCAUUAUAUGUAUGAUGAGAUCAUUAUAUG
UAUGAUCUGGACACCCAGUAGAAUC
>hsa-mir-4529 MI0016896
AUGACAGGCCAUCAGCAGUCCAAUGAAGACAUGAAGACCCAAUGUCUUCAUUGGACUGCUGAUGG

CCCGUCACUGGGA
>hsa-mir-4530 MI0016897
CGACCGCACCCGCCCGAAGCUGGGUCAAGGAGCCCAGCAGGACGGGAGCGCGGCGC
>hsa-mir-4531 MI0016898
GCCUAGGAGUCCUUGGUCAGUGGGGACAUGGAGAAGGCUUCUGAGGA
>hsa-mir-4532 MI0016899
ACAGACCCCGGGGAGCCCGGCGGUGAAGCUCCUGGUAUCCUGGGUGUCUGA
>hsa-mir-4533 MI0016900
UGAGAAUGUGGAAGGAGGUUGCCGGACGCUGCUGGCUGCCUUCCAGCGUCCACUUCCCUUUCUCU
CUCUCC
>hsa-mir-4534 MI0016901
UGUGAAUGACCCCCUUCCAGAGCCAAAAUCACCAGGGAUGGAGGAGGGGUCUUGGGUACU
>hsa-mir-4535 MI0016903
AACUGGGUCCCAGUCUUCACAGUUGGUUUCUGACACGUGGACCUGGCUGGGACGAUGUG
>hsa-mir-4536-1 MI0016906
AUGUGGUAGAUAUAUGCACGAUAUAUAUACUGCCCUGCUUUUAUACAUACAUACAUACAUACCUA
UAUCGUGCAUAUAUCUACCACAU
>hsa-mir-4536-2 MI0019149
AUGUGGUAGAUAUAUGCACGAUAUAGGUAUGUAUGUAUGUAUGUAUAAAAGCAGGGCAGUAUAUA
UAUCGUGCAUAUAUCUACCACAU
>hsa-mir-4537 MI0016908
UGAGCCGAGCUGAGCUUAGCUGGGCUGAGCUAACCAGGGCUGGGCUGAGCUGGGCUGAGCUGAGC
UGAGC
>hsa-mir-4538 MI0016909
GAGCUUGGAUGAGCUGGGCUGAACUGGGCUGGGUUGAGCUGGGCUGGGCUGAGUUGAGCCAGGCU
GAUCUGGGCUGAG
>hsa-mir-4540 MI0016911
AAGCUGCAUGGACCAGGACUUGGCACCUUUGGCCUUAGUCCUGCCUGUAGGUUUA
>hsa-mir-4632 MI0017259
GAGGGCAGCGUGGGUGUGGCGGAGGCAGGCGUGACCGUUUGCCGCCCUCUCGCUGCUCUAG
>hsa-mir-4633 MI0017260
UGGCAAGUCUCCGCAUAUGCCUGGCUAGCUCCUCCACAAAUGCGUGUGGAGGAGCUAGCCAGGCA
UAUGCAGAGCGUCA
>hsa-mir-4634 MI0017261
GGACAAGGGCGGCGCGACCGGCCCGGGGCUCUUGGGCGGCCGCGUUUCCCCUCC
>hsa-mir-4635 MI0017262
CCGGGACUUUGUGGGUUCUGACCCCACUUGGAUCACGCCGACAACACUGGUCUUGAAGUCAGAAC
CCGCAAAGUCCUGG
>hsa-mir-4636 MI0017263
UAGAUUCAGAACUCGUGUUCAAAGCCUUUAGCCCAGCAAUGGGAGAGUGCUAAAGGCUUCAAGCA
CGAGUUCUGAAUCUA
>hsa-mir-4637 MI0017264
CCCUUACUUGGAUCUGCAAUUAGUAUUUUAAUCAUAGAUUGUAUUUAGUUAGUUUUUAAUACUAA
CUGCAGAUUCAAGUGAGGG

>hsa-mir-4638 MI0017265
GACUCGGCUGCGGUGGACAAGUCCGGCUCCAGAACCUGGACACCGCUCAGCCGGCCGCGGCAGGG
GUC
>hsa-mir-4639 MI0017266
UUGCUAAGUAGGCUGAGAUUGAUGUCAGGUUAUCCCCAAGCAUAACCUCACUCUCACCUUGCUUU
GCAG
>hsa-mir-4640 MI0017267
CUGUGGGCUGGGCCAGGGAGCAGCUGGUGGGUGGGAAGUAAGAUCUGACCUGGACUCCAUCCCAC
CCACCCCCUGUUUCCUGGCCCACAG
>hsa-mir-4641 MI0017268
GGGGGGCAGGGGGCAGAGGGCAUCAGAGGACAGCCGCCUGGUGCCCAUGCCAUACUUUUGCCUCA
G
>hsa-mir-4642 MI0017269
CACAACUGCAUGGCAUCGUCCCCUGGUGGCUGUGGCCUAGGGCAAGCCACAAAGCCACUCAGUGA
UGAUGCCAGCAGUUGUG
>hsa-mir-4643 MI0017270
GUGUGCCCUAGCAUUUAUAAUCAUGUGUUCAUUCACAUGAUCAUAAGUGGACACAUGACCAUAAA
UGCUAAAGCACAC
>hsa-mir-4644 MI0017271
GCGGCGGUGCUCUGCCUCUUUCUCCAUCCACCCUGGUCCAGGUCCACAGCAGUGGAGAGAGAAAA
GAGACAGAAGGAUGGCCGU
>hsa-mir-4645 MI0017272
UGAUAGGGAAACCAGGCAAGAAAUAUUGUCUCCUCAAGUUGCGACGAGACAGUAGUUCUUGCCUG
GUUUCUCUAUCA
>hsa-mir-4646 MI0017273
ACUGGGAAGAGGAGCUGAGGGACAUUGCGGAGAGGGUCUCACAUUGUCCCUCUCCCUUCCCAG
>hsa-mir-4647 MI0017274
CCAGGAGGGUGAAGAUGGUGCUGUGCUGAGGAAAGGGGAUGCAGAGCCCUGCCCAGCACCACCAC
CUCCUAUGCUCCUGG
>hsa-mir-4648 MI0017275
UGUGGGACUGCAAAUGGGAGCUCAGCACCUGCCUGCCACCCACGCAGACCAGCCCCUGCUCUGUU
CCCACAG
>hsa-mir-4649 MI0017276
UCUGGGCGAGGGGUGGGCUCUCAGAGGGGCUGGCAGUACUGCUCUGAGGCCUGCCUCUCCCCAG
>hsa-mir-4650-1 MI0017277
UUCUGUAGAGAUUAUCAGGCCUCUUUCUACCUUCCAAGGCUCAGAAGGUAGAAUGAGGCCUGACA
UAUCUGCAGGA
>hsa-mir-4651 MI0017279
CGGCGACGGCGGGGUGGGUGAGGUCGGGCCCCAAGACUCGGGGUUUGCCGGGCGCCUCAGUUCAC
CGCGGCCG
>hsa-mir-4652 MI0017280
UAUUGGACGAGGGGACUGGUUAAUAGAACUAACUAACCAGAACUAUUUUGUUCUGUUAACCCAUC
CCCUCAUCUAAUA
>hsa-mir-4653 MI0017281

UUGUCCAAUUCUCUGAGCAAGGCUUAACACCAAAGGGUUAAGGGUUUGCUCUGGAGUUAAGGGUU
GCUUGGAGAAUUGGAGAA
>hsa-mir-4654 MI0017282
CUGGCUGGUUGUGGGAUCUGGAGGCAUCUGGGGUUGGAAUGUGACCCCAGUCUCCUUUUCCCUCA
UCAUCUGCCAG
>hsa-mir-4655 MI0017283
CCAAGGGCACACCGGGGAUGGCAGAGGGUCGUGGGAAAGUGUUGACCCUCGUCAGGUCCCCGGGG
AGCCCCUGG
>hsa-mir-4656 MI0017284
AGGCUGGCGUGGGCUGAGGGCAGGAGGCCUGUGGCCGGUCCCAGGCCUCCUGCUUCCUGGGCUCA
GGCUCGGUUU
>hsa-mir-4657 MI0017285
AAUGUGGAAGUGGUCUGAGGCAUAUAGAGUAUAUGCCAAGAACACUACCAUAU
>hsa-mir-4658 MI0017286
GCUGCCCUUCACUCAGAGCAUCUACACCCACUACCGGUGAGUGUGGAUCCUGGAGGAAUCGUGGC
>hsa-mir-4659a MI0017287
GAAACUGCUGAAGCUGCCAUGUCUAAGAAGAAAACUUUGGAGAAAAAUUUUCUUCUUAGACAUGG
CAACGUCAACAGUUUC
>hsa-mir-4660 MI0017288
ACUCCUUCUGCAGCUCUGGUGGAAAAUGGAGAAGACUUUUCCUUUCCUCCAUCUCCCCCAGGGCC
UGGUGGAGU
>hsa-mir-4661 MI0017289
UUUACUCUGAACUAGCUCUGUGGAUCCUGACAGACAGCCUGAUAGACAGGAUCCACAGAGCUAGU
CCAGAGUAAA
>hsa-mir-4662b MI0017293
CACAAUUUCUAUUUAGCCAAUUGUCUAUCUUUAGGCAUUCAGAAUAGCUAAAGAUGGACAAUUGG
CUAAAUAGACACUGUG
>hsa-mir-4663 MI0017292
CUGUGGUGGAGCUGAGCUCCAUGGACGUGCAGUGGCAUCUGUCAUUGCUGCCUUCCUGGAGCUCA
GGCCCUUGCAG
>hsa-mir-4664 MI0017294
GUUGGGGGCUGGGGUGCCCACUCCGCAAGUUAUCACUGAGCGACUUCCGGUCUGUGAGCCCCGUC
CUCCGC
>hsa-mir-4665 MI0017295
CUCGAGGUGCUGGGGGACGCGUGAGCGCGAGCCGCUUCCUCACGGCUCGGCCGCGGCGCGUAGCC
CCCGCCACAUCGGG
>hsa-mir-4666a MI0017296
AUCACUUAAAUACAUGUCAGAUUGUAUGCCUACAAAAUCCCUCCAGACUGGCAUACAAUCUGACA
UGUAUUUAAGAGAU
>hsa-mir-4666b MI0019299
UGUCUAAAUUGCAUGUCAGAUUGUAAUUCCCAGGCCCUUCCUCCAAUACUGGGAAUUACAAUUUG
ACAUGCAAUUUAGACA
>hsa-mir-4667 MI0017297
UGACUGGGGAGCAGAAGGAGAACCCAAGAAAAGCUGACUUGGAGGUCCCUCCUUCUGUCCCCACA

G
>hsa-mir-4668 MI0017298
AGGGAAAAAAAAAAGGAUUUGUCUUGUAGCCAGGAUAUUGUUUUAAAGAAAAUCCUUUUUGUUUU
UCCAG
>hsa-mir-4669 MI0017300
GCCUCCCUUCACUUCCUGGCCAUCCAGGCAUCUGUGUCUGUGUCCGGGAAGUGGAGGAGGGC
>hsa-mir-4670 MI0017301
CUCUAGGAAGCGACCAUGAUGUAACUUCACAGACUCUCCAAAAGUCUGAAGUUACAUCAUGGUCG
CUUCCUAGAG
>hsa-mir-4671 MI0017302
UAUUUUAAGACCGAAGACUGUGCGCUAAUCUCUUAGCACUGAAGAUUAGUGCAUAGUCUUUGGUC
UCAAAAUA
>hsa-mir-4672 MI0017303
GGCUGCUUCUCGCCUCUGUCCAGCUGUGUGGCCUUGGACAAGCCUCUUGGUUACACAGCUGGACA
GAGGCACGAAACAGCC
>hsa-mir-4673 MI0017304
GUCCAGGCAGGAGCCGGACUGGACCUCAGGGAAGAGGCUGACCCGGCCCCUCUUGCGGC
>hsa-mir-4674 MI0017305
CCCAGGCGCCCGCUCCCGACCCACGCCGCGCCGCCGGGUCCCUCCUCCCCGGAGAGGCUGGGCUC
GGGACGCGCGGCUCAGCUCGGG
>hsa-mir-4675 MI0017306
CAUGAGAAAUCCUGCUGGUCAACCAUAGCCCUGGUCAGACUCUCCGGGGCUGUGAUUGACCAGCA
GGACUUCUCAUG
>hsa-mir-4676 MI0017307
UGAAUGAAAGAGCCAGUGGUGAGACAGUGAGUUGAUUACUUCUCACUGUUUCACCACUGGCUCUU
UGGUUCA
>hsa-mir-4677 MI0017308
GCAAAGCAGCAAUUGUUCUUUGGUCUUUCAGCCAUGACCUGACCUUCUGUCUGUGAGACCAAAGA
ACUACUUUGCUUGGC
>hsa-mir-4678 MI0017309
GGAAAAAACAAGGUAUUGUUCAGACUUAUGAUUUUUGGGGUCAAAGAUUCUGAGCAAUAACCUAU
UAAAAAACC
>hsa-mir-4679-2 MI0017311
UAUCUUUUUUCUGUGAUAGAGAUUCUUUGCUUUUUGUUUCUAACAAAGCAAAGAAUCUCUAUCAC
AGAAAAAAGACG
>hsa-mir-4680 MI0017312
UAUAAGAACUCUUGCAGUCUUAGAUGUUAUAAAAAUAUAUAUCUGAAUUGUAAGAGUUGUUAGCA
C
>hsa-mir-4681 MI0017313
GGCAACGGGAAUGCAGGCUGUAUCUGCAGGGCAUUGUGCUAACAGGUGCAGGCUGCAGACCUGUC
ACAGGCC
>hsa-mir-4682 MI0017314
UGCCCCUGGUCUGAGUUCCUGGAGCCUGGUCUGUCACUGGGGAAGUCCAGAGCUCCAAGGCUCAG
UGCCCAGGGGACGCA

>hsa-mir-4683 MI0017315
GACACGCAAGACGAGGCGGGCCUGGAGGUGCACCAGUUCUGGCCGCUGGUGGAGAUCCAGUGCUC
GCCCGAUCUCAAGUUC
>hsa-mir-4684 MI0017316
GCACCAGGGGUACCUCUCUACUGACUUGCAACAUACAUUUGUCUUGGUGUGUUGCAAGUCGGUGG
AGACGUACCCUUGGUGC
>hsa-mir-4685 MI0017317
UAGCCCAGGGCUUGGAGUGGGGCAAGGUUGUUGGUGAUAUGGCUUCCUCUCCCUUCCUGCCCUGG
CUAG
>hsa-mir-4686 MI0017318
GGCUUCCUGUAUCUGCUGGGCUUUCUGGUGUUGGCAGCCCAAGAUGACACCCUGGGCCCAGCAGG
AGCCAGAAGCC
>hsa-mir-4687 MI0017319
ACCUGAGGAGCCAGCCCUCCUCCCGCACCCAAACUUGGAGCACUUGACCUUUGGCUGUUGGAGGG
GGCAGGCUCGCGGGU
>hsa-mir-4688 MI0017321
GUCUACUCCCAGGGUGCCAAGCUGUUUCGUGUUCCCUCCCUAGGGGAUCCCAGGUAGGGGCAGCA
GAGGACCUGGGCCUGGAC
>hsa-mir-4689 MI0017322
GGUUUCUCCUUGAGGAGACAUGGUGGGGGCCGGUCAGGCAGCCCAUGCCAUGUGUCCUCAUGGAG
AGGCC
>hsa-mir-4690 MI0017323
GAGCAGGCGAGGCUGGGCUGAACCCGUGGGUGAGGAGUGCAGCCCAGCUGAGGCCUCUGC
>hsa-mir-4691 MI0017324
GGAGCACUCCCAGGUCCUCCAGGCCAUGAGCUGCGGCCCUGAUGUCUCUACUCCAGCCACGGACU
GAGAGUGCAUAGGAGUGUCC
>hsa-mir-4692 MI0017325
GUUUUACUUGAUACCCACACUGCCUGGGUGGGACACUCAGGCAGUGUGGGUAUCAGAUAAAAC
>hsa-mir-4693 MI0017326
GUUUAAAGAAUACUGUGAAUUUCACUGUCACAAAUUCAAAUAAAGUGAGAGUGGAAUUCACAGUA
UUUAAGGAAU
>hsa-mir-4694 MI0017327
CAAAUACAUAGGUGUUAUCCUAUCCAUUUGCCUCUCUCAGAAAAUAGAGUCAAAUGGACAGGAUA
ACACCUAUGUAUUUG
>hsa-mir-4695 MI0017328
CCUGCAGGAGGCAGUGGGCGAGCAGGCGGGGCAGCCCAAUGCCAUGGGCCUGAUCUCACCGCUGC
CUCCUUCCC
>hsa-mir-4696 MI0017329
CAAAGCCACUGCAAGACGGAUACUGUCAUCUAUUCCAGAAGAUGACAAUGUCCAUUUUGCAGUGG
CUUUG
>hsa-mir-4697 MI0017330
GGGCCCAGAAGGGGGCGCAGUCACUGACGUGAAGGGACCACAUCCCGCUUCAUGUCAGUGACUCC
UGCCCCUUGGUCU
>hsa-mir-4698 MI0017331

UGCUUCUCCUGGGGUCUUCCUCUACAUUUCCACCUAGACGGGCCUGGGUCAAAAUGUAGAGGAAG
ACCCCAGAAGGAGCA
>hsa-mir-4699 MI0017332
AGCAAUUGGAGAAGAUUGCAGAGUAAGUUCCUGAUUAAGAAAUGGAAUUUACUCUGCAAUCUUCU
CCAAUUGCU
>hsa-mir-4700 MI0017333
UCAGUGAGGUCUGGGGAUGAGGACAGUGUGUCCUGAAAUUCACAGGACUGACUCCUCACCCCAGU
GCACGAGGA
>hsa-mir-4701 MI0017334
CCUUGGCCACCACACCUACCCCUUGUGAAUGUCGGGCAAUGGGUGAUGGGUGUGGUGUCCACA
>hsa-mir-4703 MI0017336
UUAUGCAUAUUAGCAAUACAGUACAAAUAUAGUGUGUUUGAUUUGCACUGUAGUUGUAUUGUAUU
GCCACUCUGUAUAA
>hsa-mir-4704 MI0017337
CUUAUCCUAGACACUAGGCAUGUGAGUGAUUGUCUUCCUCACUCAAUCAGUCACAUAUCUAGUGU
CUAGAAUGAG
>hsa-mir-4705 MI0017338
CUCACAAGAUCAAUCACUUGGUAAUUGCUGUGAUAACAACUCAGCAAUUACCAAGUGAUUGGUUU
UGUGAG
>hsa-mir-4706 MI0017339
GCUACGGGGAGCGGGGAGGAAGUGGGCGCUGCUUCUGCGUUAUCUGGAAGGAGCAGCCCACUCCU
GUCCUGGGCUCUGUGGU
>hsa-mir-4707 MI0017340
GGUUCCGGAGCCCCGGCGCGGGCGGGUUCUGGGGUGUAGACGCUGCUGGCCAGCCCGCCCCAGCC
GAGGUUCUCGGCACC
>hsa-mir-4708 MI0017341
UUUAGGAGAGAGAUGCCGCCUUGCUCCUUGAACAGGAGGAGCAAGGCGGCAUCUCUCUGAUACUA
AA
>hsa-mir-4709 MI0017342
CUGCUUCAACAACAGUGACUUGCUCUCCAAUGGUAUCCAGUGAUUCGUUGAAGAGGAGGUGCUCU
GUAGCAG
>hsa-mir-4710 MI0017344
GACCGAGUGGGGUGAGGGCAGGUGGUUCUUCCCGAAGCAGCUCUCGCCUCUUCGUC
>hsa-mir-4711 MI0017345
AAAUGUGCAUCAGGCCAGAAGACAUGAGCCCUUUGGAAAGGUCUCGUGUCUUCUGGCUUGAUGCA
CAUUU
>hsa-mir-4712 MI0017346
GACAGGAUUCCAGUACAGGUCUCUCAUUUCCUUCAUGAUUAGGAAUACUACUUUGAAAUGAGAGA
CCUGUACUGUAUCUGUU
>hsa-mir-4713 MI0017347
GUCCCCAUUUUUCUCCCACUACCAGGCUCCCAUAAGGGUCGAAUGGGAUCCAGACAGUGGGAGAA
AAAUGGGGAC
>hsa-mir-4714 MI0017348
AUUUUGGCCAACUCUGACCCCUUAGGUUGAUGUCAGAAUGAGGUGUACCAACCUAGGUGGUCAGA

GUUGGCCAAAAU
>hsa-mir-4715 MI0017349
GGGGAAUGAAAGUUGGCUGCAGUUAAGGUGGCUAAUCAGCUGAUGGUGCCACCUUAACUGCAGCC
AAUUCUAAUUCCCC
>hsa-mir-4716 MI0017350
CAUACUUUGUCUCCAUGUUUCCUUCCCCCUUCUGUAUACAUGUAUACAGGAGGAAGGGGGAAGGA
AACAUGGAGACAAAGUGUG
>hsa-mir-4717 MI0017352
GGCAGUGUUUAGGCCACAGCCACCCAUGUGUAGGGGUGGCUACACAUGGGUGGCUGUGGCCUAAA
CACUGCC
>hsa-mir-4718 MI0017353
AGCUGUACCUGAAACCAAGCACCUGUUUGUGACUUGGCUUCAGUUACUAGC
>hsa-mir-4719 MI0017354
ACAAUGAUGACUUGUAUGUUAUAGAUUUGUGAUUACAUUAAAACUUAAAAUUUCACAAAUCUAUA
AUAUGCAGGUCAUCACUGU
>hsa-mir-4720 MI0017355
AAGCCUGGCAUAUUUGGUAUAACUUAAGCACCAGGUAAAAUCUGGUGCUUAAGUUGUACCAAGUA
UAGCCAAGUUU
>hsa-mir-4721 MI0017356
GGGCCUGGUCAUGGUCAAGCCAGGUUCCAUCAAGCCCCACCAGAAGGUGGAGGCCCAGGUGAGGG
CUCCAGGUGACGGUGGGCAGGGUU
>hsa-mir-4722 MI0017357
GGCAGGAGGGCUGUGCCAGGUUGGCUGGGCCAGGCCUGACCUGCCAGCACCUCCCUGCAG
>hsa-mir-4723 MI0017359
AGUUGGUGGGGGAGCCAUGAGAUAAGAGCACCUCCUAGAGAAUGUUGAACUAAAGGUGCCCUCUC
UGGCUCCUCCCCAAAG
>hsa-mir-4724 MI0017361
ACGCAAAAUGAACUGAACCAGGAGUGAGCUUCGUGUACAUUAUCUAUUAGAAAAUGAAGUACCUU
CUGGUUCAGCUAGUCCCUGUGCGU
>hsa-mir-4725 MI0017362
GUGUCUCUCUGGAGACCCUGCAGCCUUCCCACCCACCAGGGAGCUUUCCAUGGGCUGUGGGGAAG
GCGUCAGUGUCGGGUGAGGGAACAC
>hsa-mir-4726 MI0017363
AGGGCCAGAGGAGCCUGGAGUGGUCGGGUCGACUGAACCCAGGUUCCCUCUGGCCGCA
>hsa-mir-4727 MI0017364
AAUCUGCCAGCUUCCACAGUGGCAGAUUUUCCCAUAGUGGGAAGCUGGCAGAUUC
>hsa-mir-4728 MI0017365
GUGGGAGGGGAGAGGCAGCAAGCACACAGGGCCUGGGACUAGCAUGCUGACCUCCCUCCUGCCCC
AG
>hsa-mir-4729 MI0017366
UCUGUUUCCUCAUUUAUCUGUUGGGAAGCUAACUGUGACCUUAGCGUCCCAGCAGAUAAAUGAGG
AAACAGA
>hsa-mir-4730 MI0017367
CGCAGGCCUCUGGCGGAGCCCAUUCCAUGCCAGAUGCUGAGCGAUGGCUGGUGUGUGCUGCUCCA

CAGGCCUGGUG
>hsa-mir-4731 MI0017368
CCCUGCCAGUGCUGGGGGCCACAUGAGUGUGCAGUCAUCCACACACAAGUGGCCCCCAACACUGG
CAGGG
>hsa-mir-4732 MI0017369
GAGGGAGCUGUAGAGCAGGGAGCAGGAAGCUGUGUGUGUCCAGCCCUGACCUGUCCUGUUCUGCC
CCCAGCCCCUC
>hsa-mir-4733 MI0017370
GGUCGCUUAAAUCCCAAUGCUAGACCCGGUGGCAAUCAAGGUCUAGCCACCAGGUCUAGCAUUGG
GAUUUAAGCCC
>hsa-mir-4734 MI0017371
CUCGGGCCCGACCGCGCCGGCCCGCACCUCCCGGCCCGGAGCUGCGGGCUGCGGUCAGGGCGAUC
CCGGG
>hsa-mir-4735 MI0017372
UGCAGUGCCUAAUUUGAACACCUUCGGUAUUCAUCAAAAAUACCAAAGGUGCUCAAAUUAGACAU
UGCA
>hsa-mir-4736 MI0017373
AGGCAGGUUAUCUGGGCUGCCAUCUCCCACUGGCUGCUUGCCUGCCU
>hsa-mir-4737 MI0017374
CUGCACAGGAUGCGAGGAUGCUGACAGUGCCUCACAGCCGCACAGGACCGAGGAUGCUGACGGUG
CCUCACAGCCACACAG
>hsa-mir-4738 MI0017376
GGUCGCAUUUCUCCUUCUUACCAGCGCGUUUUCAGUUUCAUAGGGAAGCCUUUCCAUGAAACUGG
AGCGCCUGGAGGAGAAGGGGCC
>hsa-mir-4739 MI0017377
GGGAGGAAGAAGGGAGGAGGAGCGGAGGGGCCCUUGUCUUCCCAGAGCCUCUCCCUUCCUCCCCU
CCCCCUCCC
>hsa-mir-4740 MI0017378
GCCAAGGACUGAUCCUCUCGGGCAGGGAGUCAGAGGGGACCGCCCGAGAGGAUCCGUCCCUGC
>hsa-mir-4741 MI0017379
CGGGCGGGGCGGGUCCGGCCGCCUCCGAGCCCGGCCGGCAGCCCCCGGCCUUAAAGCGCGGGCUG
UCCGGAGGGGUCGGCUUUCCCACCG
>hsa-mir-4742 MI0017380
UCAGGCAAAGGGAUAUUUACAGAUACUUUUUAAAAUUUGUUUGAGUUGAGGCAGAUUAAAUAUCU
GUAUUCUCCUUUGCCUGCAG
>hsa-mir-4743 MI0017381
GCUGGCCGGAUGGGACAGGAGGCAUGAAUGAGCCAUCUUUCCAAUGCCUUUCUGUCUUUUCUGGU
CCAG
>hsa-mir-4744 MI0017382
GUAAUCACAUCUAAAGACUAGACUUCGCUAUGACCAGGCCAUAGUAAACAUCAUAGUAUGUCUAG
UCUUUAGGUUUGAUUAC
>hsa-mir-4745 MI0017384
GUGAGUGGGGCUCCCGGGACGGCGCCCGCCCUGGCCCUGGCCCGGCGACGUCUCACGGUCCC
>hsa-mir-4746 MI0017385

GUGUCUGUGCCGGUCCCAGGAGAACCUGCAGAGGCAUCGGGUCAGCGGUGCUCCUGCGGGCCGAC
ACUCAC
>hsa-mir-4747 MI0017386
AGGGAAGGAGGCUUGGUCUUAGCACGGGGUCUAAGGCCCGGGCUUUCCUCCCAG
>hsa-mir-4748 MI0017387
UGGCUGGCUGAGGUUUGGGGAGGAUUUGCUGGUGCUAGAGAGGAAAGCAGACCCUACCCAACCCC
ACGCCCUACUACAGCCA
>hsa-mir-4749 MI0017388
CCUGCGGGGACAGGCCAGGGCAUCUAGGCUGUGCACAGUGACGCCCCUCCUGCCCCCACAG
>hsa-mir-4750 MI0017389
CGCUCGGGCGGAGGUGGUUGAGUGCCGACUGGCGCCUGACCCACCCCCUCCCGCAG
>hsa-mir-4751 MI0017390
CCCGGAGCCAGAGGACCCGUAGCUGCUAGAAGGGCAGGGGUGUGGCUUCUGGGGGCUGGUCUUCA
GCUCUGGCG
>hsa-mir-4752 MI0017391
AGUGUCUCCUUGUGGAUCUCAAGGAUGUGCUUCCACAUAGCAGCAUGUUCUUCAGAUGGACAAGG
AGACACU
>hsa-mir-4753 MI0017392
AUAUCUACACAAGGCCAAAGGAAGAGAACAGAUAUAUCCACAGUACACUUGGCUGUUCUCUUUCU
UUAGCCUUGUGUAGAUAU
>hsa-mir-4754 MI0017394
ACGCGCCUGAUGCGGACCUGGGUUAGCGGAGUGAGGCCCAGUGGUCACCGCCGCCCUCCGCAGGU
CCAGGUUGCCGUGCGCAUGUGCCU
>hsa-mir-4755 MI0017395
AGAUUCAGCUUUCCCUUCAGAGCCUGGCUUUGGCAUCUAUGAAAGCCAGGCUCUGAAGGGAAAGU
UGAAUCU
>hsa-mir-4756 MI0017397
GGGAUAAAAUGCAGGGAGGCGCUCACUCUCUGCUGCCGAUUCUGCACCAGAGAUGGUUGCCUUCC
UAUAUUUUGUGUC
>hsa-mir-4757 MI0017398
UUCCAGCCCGAGGCCUCUGUGACGUCACGGUGUCUGCGGGAGGAGACCAUGACGUCACAGAGGCU
UCGCGCUCUGAG
>hsa-mir-4758 MI0017399
GGUGAGUGGGAGCCGGUGGGGCUGGAGUAAGGGCACGCCCGGGGCUGCCCCACCUGCUGACCACC
CUCCCC
>hsa-mir-4759 MI0017400
CAUUUAGGACUAGAUGUUGGAAUUAGACAGAAAAAAGUUAGACACAAAAAAUUGUGUCUAAUUCC
AACAUCUAGUCCUAAAUG
>hsa-mir-4760 MI0017401
GCCAUGGUGUUUAGAUUGAACAUGAAGUUAGAAUUCUUAAGUAUCAAAACUAAAUUCAUGUUCAA
UCUAAACCCCAUGGC
>hsa-mir-4761 MI0017402
GGACAAGGUGUGCAUGCCUGACCCGUUGUCAGACCUGGAAAAAGGGCCGGCUGUGGGCAGGGAGG
GCAUGCGCACUUUGUCC

>hsa-mir-4762 MI0017403
CUGAUACCCCAAAUCUUGAUCAGAAGCCUUGAUCAGAAGCUAGGAAGGCUUCUGAUCAAGAUUUG
UGGUGUCAAG
>hsa-mir-4763 MI0017404
CCUGUCCCUCCUGCCCUGCGCCUGCCCAGCCCUCCUGCUCUGGUGACUGAGGACCGCCAGGCAGG
GGCUGGUGCUGGGCGGGGGGCGGCGGG
>hsa-mir-4764 MI0017405
UCUUCCCCAUGGAUGUGGAAGGAGUUAUCUGUCACCAGUCAGAUAACUGUCACCAGUCAGUUAAC
UCCUUUCACACCCAUGGGGAAGA
>hsa-mir-4765 MI0017406
UGGUGAUUUUGAACGUAGCUAUCCACCACUCAGCCUGGAAAAAGCUGAGUGAUUGAUAGCUAUGU
UCAAAAUCACCA
>hsa-mir-4766 MI0017407
CUGAAGCUCCUUCUGAAAGAGCAGUUGGUGUUUAUUUUUUACUAAAUAGCAAUUGCUCUUUUGGA
AGGAACUUGAG
>hsa-mir-4767 MI0017408
ACAUGGGCCCGCGGGCGCUCCUGGCCGCCGCCCGACUUCGGGGCCAGCCGGGGGCAGAGCGCGCG
GGAGCCCGAGCGU
>hsa-mir-4768 MI0017409
AAACUUUGAUUCUCUCUGGAUCCCAUGGAUAUGGGAACUGUGAUGUCCAGGAGAUCCAGAGAGAA
UCAGAGUUU
>hsa-mir-4769 MI0017410
GAGGAGAGGUGGGAUGGAGAGAAGGUAUGAGCUAAAAAUCCCCAAGCUCUGCCAUCCUCCCUCCC
CUACUUCUCCCC
>hsa-mir-4770 MI0017411
GAGUUAUGGGGUCAUCUAUCCUUCCCUUGGAAAAUGAUCUGAGAUGACACUGUAGCUC
>hsa-mir-4771-1 MI0017412
GCUCUAGCCUAAUUUUAGAUCUGGUCUGCUUCAGUUUCACUCCAAGCAGACUUGACCUACAAUUA
GCCUAGAGC
>hsa-mir-4772 MI0017414
GUGAUUGCCUCUGAUCAGGCAAAAUUGCAGACUGUCUUCCCAAAUAGCCUGCAACUUUGCCUGAU
CAGAGGCAGUCAC
>hsa-mir-4773-1 MI0017415
UGCUCCCCAGCCUUUCUAUGCUCCUGUUCUGCUUUAUUUCAUCAAAGCAGAACAGGAGCAUAGAA
AGGCUGGGGAGCA
>hsa-mir-4774 MI0017417
UAUAUUGUUGUCUGGUAUGUAGUAGGUAAUAACUGACAAACAGACAAUUGCCUAACAUGUGCCAG
AAAACAACAUA
>hsa-mir-4775 MI0017418
AUUAAGCUUUUAAUUUUUUGUUUCGGUCACUCUUGAUAGCAGACAUUGACUGAAACAAAAAAUUA
AAAGCUUUAU
>hsa-mir-4776-1 MI0017419
CUAUAUGCAGUGGACCAGGAUGGCAAGGGCUCUCCUGAAAGGACAGUAGAGCCCUUGCCAUCCUG
GUCCACUGCAUAUAG

>hsa-mir-4777 MI0017421
UAGAAUAUUUCGGCAUUCUAGAUGAGAGAUAUAUAUAUACCUCAUAUGUAUAUGGUAUACCUCAU
CUAGAAUGCUGUAAUAUUCUA
>hsa-mir-4778 MI0017422
UCACAUGUCCAAUUCUGUAAAGGAAGAAGAGGUAAGAAGAAGUGAAGCCCUCUUCUUCCUUUGCA
GAGUUGAAUAUGUGG
>hsa-mir-4779 MI0017423
UAAAUGUCUUACUGCUUUUACUGUUCCCUCCUAGAGUCCAUUCUUUACUCUAGGAGGGAAUAGUA
AAAGCAGUAAGACAUUUA
>hsa-mir-4780 MI0017424
GGCCAGUGCCAGGGGGUCAGGCUCAAGGACCAGCCCAAAGGCCAGGCCUGACCCUUGAGCCUGAU
CCCUAGCACUGAUCCC
>hsa-mir-4781 MI0017426
AGGUGCACGCUCUAGCGGGGAUUCCAAUAUUGGGCCAAUUCCCCCAAUGUUGGAAUCCUCGCUAG
AGCGUGCACUU
>hsa-mir-4782 MI0017427
AUUGCCCAGUUCUGGAUAUGAAGACAAUCAAGAAAAGAUUUGGUGUUCUUGAUUGUCUUCAUAUC
UAGAACUGGGCAGU
>hsa-mir-4783 MI0017428
GGGAAAGCGGAGGGCGCGCCCAGCUCCCGGGCUGAUUGCGCUAACAGUGGCCCCGGUGUUGGGGC
GCGUCUGCCGCUGCCCC
>hsa-mir-4784 MI0017429
UGACUGGGCUGAGGAGAUGCUGGGACUGAGAGUGUCAUGGUGGAGCCUCCGUCCCUGCUCAUCCU
CUCCGCAUGUUG
>hsa-mir-4785 MI0017430
GUAGGUGGGGACGCGGCGGCGCUGCUCCUCCGCUGCCGCCGGGAGAGUCGGCGACGCCGCCAGCU
CCGCGCGC
>hsa-mir-4786 MI0017433
GGGCAUGGCCUGAGACCAGGACUGGAUGCACCACUCUCCCUGUGAUGAGGUGAAGCCAGCUCUGG
UCUGGGCCAUUUCAC
>hsa-mir-4787 MI0017434
CGGUCCAGACGUGGCGGGGGUGGCGGCGGCAUCCCGGACGGCCUGUGAGGGAUGCGCCGCCCACU
GCCCCGCGCCGCCUGACCG
>hsa-mir-4788 MI0017435
AAUGAAGGAUUACGGACCAGCUAAGGGAGGCAUUAGGAUCCUUAUUCUUGCCUCCCUUAGUUGGU
CCCUAAUCCUUCGUU
>hsa-mir-4789 MI0017436
CAUGCUACGUAUGUAUACACCUGAUAUGUGUAUGUGUAAAUACAUAUCCACACACAUAGCAGGUG
UAUAUAUAGGUAGCCUG
>hsa-mir-4790 MI0017437
CAAUGUGACAUCGCUUUACCAUUCAUGUUCACUGAAAGGUAGAUUUUAAAAACAUGAAUGGUAAA
GCGAUGUCACAUUG
>hsa-mir-4791 MI0017438
UAAGAACUGGAUAUGAUGACUGAAAUAAGCUCCAUAUCAAUGAGAAUUUCAAUGGGAUUAUGUGC

AGUCAAUGUCCAGUAAUUA
>hsa-mir-4792 MI0017439
GCAGCCCGGUGAGCGCUCGCUGGCCUGGCAGUGCGUCGGAAGAACAGGGCGGGUGGGGCCGCGCA
CAUCUCUGC
>hsa-mir-4793 MI0017440
UUUCUCCUCGCUGCCCGCACAUCCUGCUCCACAGGGCAGAGGGAGGCCAAGAAGACCUCUGCACU
GUGAGUUGGCUGGCUGGAGGAA
>hsa-mir-4794 MI0017441
UUUUAACAUCUGGCUAUCUCACGAGACUGUAUGUCCUAACAGUGCUUGUAGUCUCAUGAGAUAGC
CAGAUGUUAAAA
>hsa-mir-4795 MI0017442
UGAUAUGGAAGAAAUCCAGAAGUGGCUAAUAAUAUUGACACUAUAACAAUAAUGUCAAUAUUAUU
AGCCACUUCUGGAUUUAUGAAUCA
>hsa-mir-4796 MI0017443
UAAAUUUGUGUCUAUACUCUGUCACUUUACUUUUGGCCUCAAGUCAUUGCAGUAAAGUGGCAGAG
UAUAGACACAAAUUUA
>hsa-mir-4797 MI0017444
GACUCAGAAGACAGAGUGCCACUUACUGAAAGGUUUUUUCUCUCAGUAAGUGGCACUCUGUCUUC
UGAGUU
>hsa-mir-4798 MI0017445
AAGUACAACUUCGGUAUACUUUGUGAAUUGGCUUUUACAAAAGACCAACUCACGAAGUAUACCGA
AGUCAUACUU
>hsa-mir-4799 MI0017446
ACUGCUAAUAUCUAAAUGCAGCAUGCCAGUCCUGAGAUGCAGGGACUGGCAUGCUGCAUUUAUAU
AUUAGCAGU
>hsa-mir-4800 MI0017448
GGAGAAAGGAGUGGACCGAGGAAGGAAGGAAGGCAAGGCUGUCUGUCCAUCCGUCCGUCUGUCCA
CCUACCUGUCAGUCC
>hsa-mir-4801 MI0017449
UUGAGGCUUGGUUUUCUUAUGUGUAAAAUGUAAUAACAUUUCUUAUGUUUAAAACACUUUACACA
AGAAAACCAAGGCUCAA
>hsa-mir-4802 MI0017450
CUGACUGGCUUGUAUGGAGGUUCUAGACCAUGUUAGUGUUCAAGUCUACAUGGAUGGAAACCUUC
AAGCAGGCCAAGCAG
>hsa-mir-4803 MI0017451
AGUGGGAUUUAACAUAAUAGUGUGGAUUGAAUCACACACACAUUUCAACCCACACUAUGAUGUUA
AAUCCCAUU
>hsa-mir-4804 MI0017452
UCAGUGUAUUUGGACGGUAAGGUUAAGCAAGGUGCGUCGUAUCUUGCUUAACCUUGCCCUCGAAA
UACACUGA
>hsa-mir-4999 MI0017865
AUAGAAAAUAAAACACAUACUGCUGUAUUGUCAGGUAGUGAUAGGAUUUAUCACUACCUGACAAU
ACAGUAUGUGUUUGUUUUAUAUAUUU
>hsa-mir-5000 MI0017866

CUGAAGAGUAGAGUGUGUGGUCCCAGUUCAGAAGUGUUCCUGAGUAACUUGUGCUUAUAACUCAG
GACACUUCUGAACUUGGACCAUACAGGUCUCCCUGCUU
>hsa-mir-5001 MI0017867
AGCUCAGGGCGGCUGCGCAGAGGGCUGGACUCAGCGGCGGAGCUGGCUGCUGGCCUCAGUUCUGC
CUCUGUCCAGGUCCUUGUGACCCGCCCGCUCUCCU
>hsa-mir-5002 MI0017868
UCUUCCUCUCUGUCCUCUGGAAUUUGGUUUCUGAGGCACUUAGUAGGUGAUAGCAUGACUGACUG
CCUCACUGACCACUUCCAGAUGAGGGUUACUC
>hsa-mir-5003 MI0017869
AUGAGUUUGCUUUGUGUCAUCCUCACAACAACCUUGCAGGGUAGAGAUGAUUUUUCCUACUUUUC
UAGGUUGUUGGGGGCUGGGGCAGGGGGAACAGAG
>hsa-mir-5004 MI0017870
GGCACUUGCUUGGGGGUUAGUGAGGACAGGGCAAAUUCACGAGAUUGGGUUGUGCAGAGGCUGAC
ACUUGGAUUUUCCUGGGCCUCAGGACUUCCUUUCAGACAUGG
>hsa-mir-5006 MI0017873
AACCAUUAGGGGGCUGUGGUUUGCCAGGGCAGGAGGUGGAAGGGAGCCCCAUUUACAGUGGUAAC
UUCCUUUCCCUUUCCAUCCUGGCAGGCUUCAGAGAACUUUACCAG
>hsa-mir-5007 MI0017874
GGUAAACCUUGGUGACUAAUUAGAGUCUGGCUGAUAUGGUUUGACACAGAGCUAAAUCAUAUGAA
CCAAACUCUAAUUAGUCAAUAAUUUCUGUU
>hsa-mir-5008 MI0017876
GGGCUGACCCCUAGGGUCAGGUGAGGCCCUUGGGGCACAGUGGUGCCAUCUCCCCUGUGCUCCCA
GGGCCUCGCCUGUCCCUUGAGGUCGGCCC
>hsa-mir-5009 MI0017877
GACCAGAAGUGUUUUGGAUUUUGGACUUUUUCAGAUUUGGGGAUAUUUGCAUUAUACUUAUCCUA
AAUCUGAAAGUCCAAAACCUGAAAUGACCAAUAAG
>hsa-mir-5010 MI0017878
GAUCCAGGGAACCCUAGAGCAGGGGGAUGGCAGAGCAAAAUUCAUGGCCUACAGCUGCCUCUUGC
CAAACUGCACUGGAUUUUGUGUCUCCCAUUCCCCAGAGCUGUCUGAGGUGCUUUG
>hsa-mir-5011 MI0017879
AGAUGGUAUUGAGUGGAUGCUGUUAUAUAUACAGCCAUGCACUCUGUAGUUUGGGUACACAGUGC
AUGGCUGUAUAUAUAACACUAUCCAUUCAUCUUUCAGC
>hsa-mir-5047 MI0017932
GAAGCGCUUGCCUAGACGAGACACAGUGCAUAAAAACAACUUUUGGGGGACAGGUAUGUUUUCUU
GCAGCUGCGGUUGUAAGGUCUUGGCAAGACAAGCA
>hsa-mir-5087 MI0017976
AGCUUUCUACGGGUUUGUAGCUUUGCUGGCAUGUUAAGUGUUGUCCUACAGUCGCAAGCAUAAGA
AAGAGAAAGUA
>hsa-mir-5088 MI0017977
CCCAUCAGGGCUCAGGGAUUGGAUGGAGGUGAUGGGGGCAGGGGAUGGGUCUCACCCUCCCUUCU
UCCUGGGCCCUCAG
>hsa-mir-5089 MI0017978
AAGGACUUCAGUGGGAUUUCUGAGUAGCAUCCUUGGAAUCUGCACUCAAGGGAUGCUACUCGGAA
AUCCCACUGAAGUCCUUUU

>hsa-mir-5090 MI0017979
UCUGAGGUACCCGGGGCAGAUUGGUGUAGGGUGCAAAGCCUGCCCGCCCCCUAAGCCUUCUGCCC
CCAACUCCAGCCUGUCAGGA
>hsa-mir-5091 MI0017980
GACUGUGGCGACGGAGACGACAAGACUGUGCUGGUCGCGGGUUGUGGGGUUUAGGUCACCGGCAG
GGGUCUGGAGUCCCUGGAGGUUAGGGCU
>hsa-mir-5092 MI0017981
AUCCCAGAUCAGAUGCCAAAGCCAGUGGGGACUGGACAACAUGAUGAGCCCAAACCAAUCCACGC
UGAGCUUGGCAUCUGAUUUGGGA
>hsa-mir-5093 MI0017982
CCCGCCAGGUCCACAUGCCAGAGUGUCAACGUGACCCAGCCAGCCUCCUUCCUGAGCUAGGAGGA
UUAGGAAAUGAGGCUGGCUAGGAGCACAGCCAGGG
>hsa-mir-5094 MI0017983
AAAAGAAAAAAAUCAGUGAAUGCCUUGAACCUAACACACUGCCUUUUAUGUGGUAGGUACAGUGG
GCUCACUGAAACAUUCAACU
>hsa-mir-5095 MI0018001
CUGGGAUUACAGGCGUGAACCACCGCGCCCGGCCUAACUUUUAAGAAACGUCGGCCCGGGAGCGG
UGGCUCACGCCUGUAAUCCCAGC
>hsa-mir-5096 MI0018004
AGUAGAGGUGGGGUUUCACCAUGUUGGUCAGGCUGGUCUCAAACUCCUGACCUCAGGUGAUCCAU
CCACC
>hsa-mir-5100 MI0019116
CCAUGAGGAGCUGGCAGUGGGAUGGCCUGGGGGUAGGAGCGUGGCUUCUGGAGCUAGACCACAUG
GGUUCAGAUCCCAGCGGUGCCUCUAACUGGCCACAGGACCUUGGGCAGUCAGCU
>hsa-mir-5186 MI0018165
UCAGCCAGCUUAUGACUUGACCCUCUCACCUGAUUUCUACCAACCUUUCCUCAGCUGAUUUCUUU
CUGGGGAGAGAUUGGUAGAAAUCAGGUGAGAGGGUCAUGCCAUAAGCUGGCUAAC
>hsa-mir-5187 MI0018166
GACUAAGGGUGGGAUGAGGGAUUGAAGUGGAGCAGGAAUGCGCUUUUCUCCACUGAAUCCUCUUU
UCCUCAGGUGG
>hsa-mir-5188 MI0018167
GGGAGGCAUGGAAAUUUCUCUGGUUUCAAUGGGUACGAUUAUUGUAAGCAGGAUCCAUUCAAUAA
UCGGACCCAUUUAAACCGGAGAUUUUAAAAGACAGGAAUAGAAUCCCA
>hsa-mir-5189 MI0018168
GGCCCGCCUUUUAGGGGCCUCGCUGUCUGGGCACAGGCGGAUGGACAGGCUGGCCUCUGGAUGAC
CUGCCAACCGUCAGAGCCCAGACCCACGUGGCCUCAGUUGGGGACCAGG
>hsa-mir-5190 MI0018169
GGUCAUACCCUGGCUCCAGCCCUGUCACAUGGUUAAUGUUCCACAGCCAGUGACUGAGCUGGAGC
CAGGGCCACUGCCCC
>hsa-mir-5191 MI0018170
AGUUGGCCAGGACCCCAAGCCCCCAGCACUUCAUUCUUGCUGUCCUCUCCUGGUCUGGGAGGAUA
GAAGAGAGGAUAGGAAGAAUGAAGUGCUGGGCGCUUAGGGGGAUCCUGGCCAACU
>hsa-mir-5192 MI0018171
UUAGUUCCAGCCUCCUGGCUCACCUGGAACCAUUUCUCCUGGGAAGCAUGGUAGCCAGGAGAGUG

GAUUCCAGGUGGUGAGGGCUUGGUACU
>hsa-mir-5193 MI0018172
CCUAGGAAAGGCUGCUGGUAACUGGGAUGGGGGUUGGGGGGAGGUAAGAAGUCUCUGACUCCUCC
UCUACCUCAUCCCAGUUCCAUCACCUGAAGUGGACCUCUUGGGA
>hsa-mir-5194 MI0018173
AUUUCUUUGGGUUAACUUAAACUCAGCCCUUCUAGGCCCAUUCUUUUCACUCAGGAAUUGGAUAA
GCUUUUCUGAGGGGUUUGGAAUGGGAUGGCAGGGAGAGUCACCAGACACCAUGAA
>hsa-mir-5195 MI0018174
GAGCAAAAACCAGAGAACAACAUGGGAGCGUUCCUAACCCCUAAGGCAACUGGAUGGGAGACCUG
ACCCAUCCAGUUCUCUGAGGGGGCUCUUGUGUGUUCUACAAGGUUGUUCA
>hsa-mir-5196 MI0018175
UCUGAGGAGACCUGGGCUGUCAGAGGCCAGGGAAGGGGACGAGGGUUGGGGAACAGGUGGUUAGC
ACUUCAUCCUCGUCUCCCUCCCAGGUUAGAAGGGCCCCCCUCUCUGAAGG
>hsa-mir-5197 MI0018176
UAUGGGAUUCCACAGACAAUGAGUAUCAAUGGCACAAACUCAUUCUUGAAUUUUUGCCAGUUCAA
GAAGAGACUGAGUCAUCGAAUGCUCUAAAUGUCACUUCACCUCAUGU
>hsa-mir-5571 MI0019115
AUCUGACACAAAAUGUGAACCAAGCAAUUCUCAAAGGAGCCUCCCAGGAAAUUCACUUUAGGAAG
UCCUAGGAGGCUCCUCUGAGAGUUGCUAAAACAAAACAUUGAGAGUCC
>hsa-mir-5572 MI0019117
AGCCAGACAAGAGGGUCAUGGGGAGUCACUGUCAACCCAGAGCAGGCACUGCCCCUGCGACCAGC
CUGGGGCAUCGGUUGGGGUGCAGGGGUCUGCUGGUGAUGCUUUCCAUCUCUUUGCUUUGUCCUGA
UUGUAGC
>hsa-mir-5579 MI0019133
UAUGGUACUCCUUAAGCUAACAGGCCCCUGUCACCAUUAGCUUAAGGAGUACCAGAUC
>hsa-mir-5580 MI0019135
UGCUGGCUCAUUUCAUAUGUGUGCUGAGAAAAUUCACACAUAUGAAGUGAGCCAGCAC
>hsa-mir-5581 MI0019136
AGCCUUCCAGGAGAAAUGGAGACCCUAUACAUACCUGUUUCCAUGCCUCCUAGAAGUUCC
>hsa-mir-5582 MI0019138
UAGGCACACUUAAAGUUAUAGCUACAUCAGUUAUAACUAUAUCAGUUAAAACUUUAAGUGUGCCU
AGG
>hsa-mir-5583-1 MI0019139
AAACUAAUAUACCCAUAUUCUGGCUAGGUGAUCAUCAGAAUAUGGGUAUAUUAGUUUGG
>hsa-mir-5584 MI0019141
CAGGGAAAUGGGAAGAACUAGAUUUGAAUCCAGACCUUUAGUUCUUCCCUUUGCCCAAUU
>hsa-mir-5585 MI0019142
UGAAGUACCAGCUACUCGAGAGGUCAGAGGAUUGCUCCUGAAUAGCUGGGACUACAGGU
>hsa-mir-5586 MI0019143
UAUCCAGCUUGUUACUAUAUGCUUUUUAAAUGGGGCACAGAGUGACAAGCUGGUUAAAG
>hsa-mir-5587 MI0019144
AUGGUCACCUCCGGGACUCAGCCCUGUGCUGAGCCCCGGGCAGUGUGAUCAUC
>hsa-mir-5588 MI0019147
ACUGGCAUUAGUGGGACUUUUUUUUUUUUUUUUUUUAAUGUUAAAAGUCCCACUAAUGCCAGC

>hsa-mir-5589 MI0019148
GGCUGGGUGCUCUUGUGCAGUGAGCAACCUACACAACUGCACAUGGCAACCUAGCUCCCA
>hsa-mir-5590 MI0019150
UUGCCAUACAUAGACUUUAUUGUGUUGAUCAACAAUAAAGUUCAUGUAUGGCAA
>hsa-mir-5591 MI0019151
UGGGAGCUAAGCUAUGGGUAUACUGAGCUUAUGUAUGCAUCUGCAUACCCAUAGCUUAGCUCCCA
>hsa-mir-5680 MI0019280
GCAUUGGGUUAGCAGGUUAGCCCAGCAUUUCCCUUCCUGGACACACAGGAGGAGAAAUGCUGGAC
UAAUCUGCUAAUCCAAUGC
>hsa-mir-5681a MI0019281
AGUUUUUGAAGAGUAUUGCCACCCUUUCUAGUCCCUAUUAGACUAGAAAGGGUGGCAAUACCUCU
UCCAAAAACU
>hsa-mir-5682 MI0019282
GGCCCAUGGGUCUUAUCCUGCAAGGUGCUGCAGAGACGAGGCCUGUAGCACCUUGCAGGAUAAGG
UCUACUGGGCC
>hsa-mir-5683 MI0019284
GGAGCUUGUUACAGAUGCAGAUUCUCUGACUUCUUACUGCACCAGUGAAGUCAGGAUCUGCAUUU
GAAUAAGACCC
>hsa-mir-5684 MI0019285
GCUGAACUCUAGCCUGAGCAACAGAGUGAGAUGGUCUUGUUUUGUUGCCCAGGCUGGAGUCCAGU
>hsa-mir-5685 MI0019287
CUCUACAUCACAGCCCAGCAGUUAUCACGGGCCCCUCCCCUCAAUGGGCCCGUGAUAACUGCAGG
GCUGUGAUGUAGAG
>hsa-mir-5687 MI0019291
CCUCACUUAUCUGACUCUGAAAUCUUCUAAAUGGUACCCACUUUAUUUAGAACGUUUUAGGGUCA
AAUAAGUACAGG
>hsa-mir-5688 MI0019292
GAAACACUUUGCCUUUUUACAGGAGUUUAUUAUGUUUUGGACAUAGAAACAUAACAAACACCUGU
AAAACAGCAAAGUGUUUC
>hsa-mir-5689 MI0019294
AGCGUGGUAGCAUACACCUGUAGUCCUAGAUACUCAGGAGGGUGAGUAUCUAGGACUACAGGUGU
GUGCUACCACGCU
>hsa-mir-5690 MI0019295
CUUUUAAUUUCAGCUACUACCUCUAUUAGGAUUUGGGAGUUAUACUAAUAGAGGUAAUAGUUGAA
AUUAAGAG
>hsa-mir-5691 MI0019296
GGACAAGCUUGCUCUGAGCUCCGAGAAAGCUGACAGACAGCUGCUUGGUGUUCAGAGCUUGUCUG
UCC
>hsa-mir-5692a-2 MI0019298
UACAAAUAAUACCACAGUGGGUGUACCUCAUGUGUGUACACCCUGUGAUAUUAUUUGUA
>hsa-mir-5692b MI0019311
GAUAUUAUGAAUAAUAUCACAGUAGGUGUUCACACAUAAUGUGUACACCAUGUGUGUACACCCAU
GUGAUAUUUGAAGUAGUAUGUC
>hsa-mir-5692c-1 MI0019288

UAUAACAUUGUAUAUACCCACUGUGAUAUUAAGAGUAAUAGCUCUCUAGGUUAUUAUGAAUAAUA
UCACAGUAGGUGUACACAAUGUUGUA
>hsa-mir-5693 MI0019300
CUGGGAAGUUAGUUCAUUUCAGUCUGUGCUGUGAGCUAGCCAGCAGUGGCUCUGAAAUGAACUCA
AACUCUAG
>hsa-mir-5694 MI0019301
GCCAACUGCAGAUCAUGGGACUGUCUCAGCCCCAUAUGUAUCUGAAGGCUGAGAAGUCCCAUGAU
CCGCACUUGGC
>hsa-mir-5695 MI0019302
CAAGGCCUAUCUAUCUAGAUUCUUCUUGGCCUCUCUGAGCAUGCAUUCCUGAGACUCCAAGAAGA
AUCUAGACAGAUAGGCCUUG
>hsa-mir-5696 MI0019303
GUGCUCAUUUAAGUAGUCUGAUGCCUACUACUGAUGACAUACAAUGUAAGUGCUCAUUUAGGCGU
CAGACUACCUAAAUGAGCAC
>hsa-mir-5697 MI0019304
AGCAUAUUCUCAAGUAGUUUCAUGAUAAAGGGUGUAUGAGAGAUCAACCCUUUAUCAUGAAACGC
UUGAGGAUACGCU
>hsa-mir-5698 MI0019305
CUGUGCACCUGGGGGAGUGCAGUGAUUGUGGAAUGCAAAGUCCCACAAUCACUGUACUCCCCAGG
UGCACAG
>hsa-mir-5699 MI0019306
CUGUACCCCUGCCCCAACAAGGAAGGACAAGAGGUGUGAGCCACACACACGCCUGGCCUCCUGUC
UUUCCUUGUUGGAGCAGGGAUGUAG
>hsa-mir-5700 MI0019307
UUAAUUAAUGCAUUAAAUUAUUGAAGGCCCUUGGGCACCCCAGGCCUUCAAUAAUUUAAUGCAUU
UAUUGA
>hsa-mir-5701-1 MI0019308
GAUUGGACUUUAUUGUCACGUUCUGAUUGGUUAGCCUAAGACUUGUUCUGAUCCAAUCAGAACAU
GAAAAUAACGUCCAAUC
>hsa-mir-5702 MI0019309
GCCUCAACUCCUGGGAUAUGUUGCUGAUCCAACCUGAAAUCCUUCUGUAGGUUGAGUCAGCAACA
UAUCCCAUGACUUUUGGGU
>hsa-mir-5703 MI0019310
UUGCCGUCCCCUUCCUCGUCUUUUCCCCUCAGGAGAAGUCGGGAAGGUGGCGGCGG
>hsa-mir-5704 MI0019312
UGAUCUUGUUUAGGCCAUCAUCCCAUUAUGCUAAGUCCAUGGGCAAACAUAACAGGAUGAUGGCC
UAAACAAGACCA
>hsa-mir-5705 MI0019313
UCCCCAUUUACACAGGCCAUGAGCCCCGAAACACCCAUCCCAGGAUUGCUGAUGGGUGUUUCGGG
GCUCAUGGCCUGUGUAAAUGGGGA
>hsa-mir-5706 MI0019314
AGCUAGGUCUUCUGGAUAACAUGCUGAAGCUUCUACGUCAUUCAGCACUUGCUUCAGCAUGUUUU
CCAGAGGAUCUAGCU
>hsa-mir-5707 MI0019315

UGUAAGAACACGUUUGAAUGCUGUACAAGGCACAUAUGUGAACAUUGUACCACAUGUACAGCUUU
CAAACAUGCUCUUAUA
>hsa-mir-5708 MI0019316
AUUACAGACAUGAGCGACUGUGCCUGACCAAAAGUCAACAUUAAACAACAAAUCUUGGCCAGGCA
CAGUGGCUCAUGCCUGUAAU
>hsa-mir-5739 MI0019412
GGUUGGCUAUAACUAUCAUUUCCAAGGUUGUGCUUUUAGGAAAUGUUGGCUGUCCUGCGGAGAGA
GAAUGGGGAGCCAGG
>hsa-mir-6068 MI0020345
CCUGCGAGUCUCCGGCGGUGGCUUGUGGCUGAGUGUCACGCUGCUGGCGCAGGCUCGGCC
>hsa-mir-6069 MI0020346
UGGUGACCCCUGGGCUAGGGCCUGCUGCCCCCUGCCCAGUGCAGGAGGGUGGAGGGUCACUCCUU
AGGUGGUCCCAGUG
>hsa-mir-6070 MI0020347
GGGUCACUUCUAGCCUCUCCGGUUCCAGUCCCUGGAGCUCUGUGCCUUUGGGUCCUGGUGAGCUC
UUGUUGAUUGCAGUGGGAGAUGGGGAGAAUUGCCUUCA
>hsa-mir-6071 MI0020348
GUUUCUGCUGCCGGCCAAGGCUCUGGGUCCGGCUUACUCCCUAGCUUACUGUCCUCAGAACCCCC
GCCACCACAGAAG
>hsa-mir-6072 MI0020349
GGGUGGGGGCUGGUGCAGGGAUGGGCUUUGCUACUGCUCCUCAUCACACUGCACCUUAGCUGCUG
CCUGGA
>hsa-mir-6073 MI0020350
UAGAUGUUGGUCCAAACUGAAAGUUGAUGAGUCACUGUGCCUCUCGGGGUAGUGAGUUAUCAGCU
ACAGUGAGAGAGCAGUGUUUGGCC
>hsa-mir-6074 MI0020351
UACCAACCCCAUGGAAUUUUUACUCACCUUCAGUCAACUGAUUUGCUCUUUGGUGGAGAUAUUCA
GAGGCUAGGUGGAGAUAGAGGUAGCCUUGAGGGUGGGUGUGG
>hsa-mir-6075 MI0020352
GACACCACAUGCUCCUCCAGGCCUGCCUGCCCUCCAGGUCAUGUUCCAGUGUCCCACAGAUGCAG
CACCACGGCCCAGGCGGCAUUGGUGUCACC
>hsa-mir-6076 MI0020353
AGCAUGACAGAGGAGAGGUGGAGGUAGGCGAGAGUAAUAUAAUUUCUCCAGGAGAACAUCUGAGA
GGGGAAGUUGCUUUCCUGCCCUGGCCCUUUCACCCUCCUGAGUUUGGG
>hsa-mir-6077-1 MI0020354
CCUGCGUAGAAAGGGAAGAGCUGUACGGCCUUCGCGCUAGUUUGUUUGUGUUAAGGCUGACGCUC
CCUAUUUCUCCUCAGUG
>hsa-mir-6078 MI0020355
GCAGAGGAGAAAGAGCCACCGCCUGAGCUAGCUGUGGACAGAGGCAAAACUCUAACUUCAGCUGG
UUUUGAGUGAGAAGUGGGUCUCUGUUAAAAUGCUA
>hsa-mir-6079 MI0020356
UCCUAGACCUAGUAUCAGUGGCCAAAGAAACUUUUUUGGAAGCUUGGACCAACUAGCUGGCU
>hsa-mir-6080 MI0020357
GCCUGCGGCUCUGGGAAUGCCGGUCUGGGGCUGGUUGUCUAGUGCGGGCGUUCCCGUUCGCGGGG

A
>hsa-mir-6081 MI0020358
CCACCACGGUGCUGGCACCAGGGCCUCUGCCCCGUAGGACACCGAGGCUUAUGAAUAGGAGCAGU
GCCGGCCAAGGCGCCGGCACCAUCUUGGUGAU
>hsa-mir-6082 MI0020359
UUGAAGAAUACGUCUGGUUGAUCCAGUCCACUUAAUUCUGAGAAAGUCCACAGACUGGAUCGGGU
CUUGGAUUUUAGCUGGAUUCAUUGACUGGGCUAUAUUUGUCAUA
>hsa-mir-6083 MI0020360
GUAAAAAGAGUCCAGAGUCUGGGAAGGUGGAAAGGGAGCAGGAGCAUCGUCUUUAAGAGGGUCAG
GUACCUUUGCUCUUAUAUCAGAGGCUGUGGGCUUCCUACAG
>hsa-mir-6084 MI0020361
AGCGUCCAGGCUGGGCCGCAGGACCGGGCGCGGAGCCUCGCAGGGUCGGGCUCGGGCUCCCUAAC
CGUCUCCGCUUCUUCCGCCAGUCGGUGGCCGGGCUGGCGGCGCGG
>hsa-mir-6085 MI0020362
GUCUACCAGGUGUGGGCCCAGCUUUACAUAGUUCAUGCUGAGGCCGGGAUUUCAUGCAGAAAACU
GGUUGCAAAAGGUGCUGAAGGGGCUGGGGGAGCACAAGGGAGAAG
>hsa-mir-6086 MI0020363
AGGAGGUUGGGAAGGGCAGAGAUGAGCAUAAAGUUUUUGCCUUGUUUUUCUUUUU
>hsa-mir-6087 MI0020364
GGUGAGGCGGGGGGGCGAGCCCUGAGGGGCUCUCGCUUCUGGCGCCAAG
>hsa-mir-6088 MI0020365
AGAGAUGAAGCGGGGGGGCGGGGUCUUGCUCUAUUGCCUACGCUGAUCUCA
>hsa-mir-6089-1 MI0020366
CCCCGGGCCCGGCGUUCCCUCCCCUUCCGUGCGCCAGUGGAGGCCGGGGUGGGGCGGGGCGGGG
>hsa-mir-6090 MI0020367
CGCUGGGUCCGCGCGCCCUGGGCCGGGCGAUGUCCGCUUGGGGGAGCGAGGGGCGGGGCG
>hsa-mir-6124 MI0021258
GGGGAGGUAGGGAAAAGGAAGGGGGAGGAGAAGGUGAGACCAAUGUCCUGGGUGCCACUCCUGCC
CAGUGCCUCCCUUCCUCGUU
>hsa-mir-6125 MI0021259
GCUCUGGGGCGUGCCGCCGCCGUCGCUGCCACCUCCCCUACCGCUAGUGGAAGAAGAUGGCGGAA
GGCGGAGCGGCGGAUCUGGACACCCAGCGGU
>hsa-mir-6126 MI0021260
AGCCUGUGGGAAAGAGAAGAGCAGGGCAGGGUGAAGGCCCGGCGGAGACACUCUGCCCACCCCAC
ACCCUGCCUAUGGGCCACACAGCU
>hsa-mir-6127 MI0021271
ACUUUCUCCAUCUACCAAGAUGAGGGAGUGGGUGGGAGGUGGGAAGGCUGCCCCAAAUGGCCUCU
AACAUCCCUUCCAGUCUCCUCCUCCUCCUCCUCCUUCUUCUUCU
>hsa-mir-6128 MI0021272
AAGAAGCUUGUAGAUUUUUCUCCCUUACUAUCUAGAAUUAUAGGACUUCAGUCCAUGAUUUGGAA
AAAUUACUGGAAUUGGAGUCAAAAAUAAUUUGAAAAUUAGGAAU
>hsa-mir-6129 MI0021274
GUUCCUUCUAAGGAUUCCAGUGAGGGAGUUGGGUGUAUAGGAAGGAAAUCUUAUUGUCCAAGUUU
CCCUUUGAAACCUCUUAGAAUGGAAUAAAACUGCCCAAAUCAUA

>hsa-mir-6130 MI0021275
CAAUGAAUGUGGAAAUGCAGGCAUCCCUUCUAUAUACUGAUGUAAUUUUCCUUGGAUUUAUAUGC
AGUACUGAGGGAGUGGAUUGUAUGGUAGUUCUAUUUUUACAUUU
>hsa-mir-6131 MI0021276
UCCCGCAUUCCCUCUGCUUUGGUCAGGUGGUGCCCUCCUUCCAUGGGUAGAGCCAGAGAUGGUGG
GUUCUGGCUGGUCAGAUGGGAGUGGACAGAGACCCGGGGUCCUC
>hsa-mir-6132 MI0021277
UGCUAUUGUCUUACUGCUACAGCAGGGCUGGGGAUUGCAGUAUCCGCUGUUGCUGCUGCUCCCAG
UCCUGCCCCUGCUGCUACCUAGUCCAGCCUCACCGCAUCCCAGA
>hsa-mir-6133 MI0021278
GGAAUGUCACCUGUGUGUUUUCUCUGCAUGCCCUCUUCAUUGUUCUGCUGAAGACUGGUCUCUUC
AUGUGUGAGGGAGGAGGUUGGGUAUUGAGGGAAAACAGGGGGC
>hsa-mir-6134 MI0021279
UGGGCCUUGAUGUCAGUAGGGCUCAGUAGGGAACUGACCCUCUAUUUCCACCUGCAUAAAUGAAG
CUGAAUGAGGUGGUAGGAUGUAGAGCUGGUUGACACUCUGCUUU
>hsa-mir-6165 MI0021472
CAGCAGGUCAGCAGGAGGUGAGGGGAGAGGAUCCACCUGUCCUGUCCUGUCCUCUCCUGCCCUGU
CCUGGCUCCAGCCCCUCCC
>hsa-mir-6499 MI0022209
UCAGUCGGGCGCAAGAGCACUGCAGUUCUGUUGGGUGACAGCAGUGUUUGUUUUGCCCACAG
>hsa-mir-6500 MI0022211
CCUGCCUGCAGAAAGGAGCUAUCCACUCCAGGUGUCCUUUCUUCUGAGAGCUGGACACUUGUUGG
GAUGACCUGCCUGCAGGUAGG
>hsa-mir-6501 MI0022213
GGAGUUGCCAGGGCUGCCUUUGGUGACAGCAGCAGUAGAGUUGCCAGAGCAGCCUGCGGUAACAG
UA
>hsa-mir-6502 MI0022214
CAGAGUGGGAGCUCUAGAAAGAUUGUUGACCAAUCAUCUUAUUGACUAGACCAUCUUUCUAGAGU
AUAACUAUUUU
>hsa-mir-6503 MI0022215
AAUGGUCCCCCCAGGGAGGUCUGCAUUCAAAUCCCCAGAAGCUGAGGAUUAGGGGACUAGGAUGC
AGACCUCCCUGGGGGACCAUU
>hsa-mir-6504 MI0022216
GCAGUCUGGCUGUGCUGUAAUGCAGUCUGCACCCUGCUGCAUUACAGCACAGCCAUUCUCU
>hsa-mir-6505 MI0022217
GCAUUGGAAUAGGGGAUAUCUCAGCAUGUUGAGCCCUGUCUCUGGGGAGCUGACUUCUACCUCUU
CCAAAG
>hsa-mir-6506 MI0022218
GACUGGGAUGUCACUGAAUAUGGUGUUUGUGAGUUGAUUGACAUCGUAUCAGAGAUUCCAGACAC
A
>hsa-mir-6507 MI0022219
GGAGGGAAGAAUAGGAGGGACUUUGUAUUGUGGUUCAGUACCAUGCAAAGUCCUUCCUAUUUUUC
CCUCC
>hsa-mir-6508 MI0022220

UUCCUCUAGAAAUGCAUGACCCACCCUGAGUUUUGGUGGGCCAUGCAUUUCUAGAACUCC
>hsa-mir-6509 MI0022221
UUUUUGUGUGUGAAAUUAGGUAGUGGCAGUGGAACACUAUAUUAAUCAGGUUUCCACUGCCACUA
CCUAAUUUCUCAGAUGGAAA
>hsa-mir-6510 MI0022222
AGCAGCAGGGGAGAGAGAGGAGUCCUCUAGACACCGACUCUGUCUCCUGCAGAU
>hsa-mir-6511b-1 MI0022552
GGGACGGGGCCUGCAGGCAGAAGUGGGGCUGACAGGGCAGAGGGUUGCGCCCCCUCACCACCCCU
UCUGCCUGCAGCGGUGGGCU
>hsa-mir-6512 MI0022224
UAUGCUCUUACCAUUAGAAGAGCUGGAAGAAGGCUGAGGGAGAUGCCUUCUUCCAGCCCUUCUAA
UGGUAGGAGCAU
>hsa-mir-6513 MI0022225
GCUUUGGGAUUGACGCCACAUGUCUCAGGUCCCCAGCUGAGUCAAGUGUCAUCUGUCCCUAGGC
>hsa-mir-6514 MI0022226
UAUGGAGUGGACUUUCAGCUGGCAUUUACGAGUCAGAGUUCUUACAGAGCUGCCUGUUCUUCCAC
UCCAG
>hsa-mir-6515 MI0022227
CAUUGGAGGGUGUGGAAGACAUCUGGGCCAACUCUGAUCUCUUCAUCUACCCCCCAG
>hsa-mir-6516 MI0025513
UGGGUUUUGAAUUUGCAGUAACAGGUGUGAGCAUUCUAGCAGCAGUUUGAUGAUCAUGUAUGAUA
CUGCAAACAGGACCUA
>hsa-mir-6715a MI0022548
UGGGCUGCGUCCACAGGCACAGCCGGUUUGAGCAUUUUUACUGAAUUGCCAAACCAGUCGUGCCU
GUGGGCACAACCUU
>hsa-mir-6715b MI0022549
GGUUGUGCCCACAGGCACGACUGGUUUGGCAAUUCAGUAAAAAUGCUCAAACCGGCUGUGCCUGU
GGACGCAGCCCA
>hsa-mir-6716 MI0022550
GAGAGGCCAAGACCUUGGGAAUGGGGGUAAGGGCCUUCUGAGCCCAGGUCCGAACUCUCCAUUCC
UCUGCAGAGCGCUCU
>hsa-mir-6717 MI0022551
CUGGUGUUUGAGGCGAUGUGGGGAUGUAGAGACAACUUCCCAGUCUCAUUUCCUCAUCCUGCCAG
GCCACCAU
>hsa-mir-6718 MI0022553
AGUGAAUCCCUAGUGGUCAGAGGGCUUAUGAUAUAUUGUGAGAGCCAUGUCAUAAGCCUUUUGGC
CACUAGGGAUUCAAU
>hsa-mir-6719 MI0022554
GCCCUACUCUCAGGAGGAGGCUGAUGUCUUCAGAGCAGGGUUUGGUCUGUGCUUCUCUGACAUCA
GUGAUUCUCCUGCUGCUUUUAU
>hsa-mir-6720 MI0022555
UUGAGCGAGAGAUUGUGGCGCACCGAGUUCUUCCAGCCCUGGUAGGCGCCGCGGAAGAAGGGGAA
GCGCGCCUGCAGGAACUGGUAGAUCUCGCUGAG
>hsa-mir-6721 MI0022556

CCCUCAUCUCUGGGCAGGGGCUUAUUGUAGGAGUCUCUGAAGAGAGCUGUGGACUGACCUGCUUU
AACCCUUCCCCAGGUUCCCAUU
>hsa-mir-6722 MI0022557
GGCCUCAGGCAGGCGCACCCGACCACAUGCAUGGCUGGUGGCGGCGUGCAGGGGUCGGGUGGGCC
AGGCUGUGGGGCG
>hsa-mir-6723 MI0022558
AUGCAUCGGGAUAGUCCGAGUAACGUCGGGGCAUUCCGGAUAGGCCGAGAAAGUGUUGUGGGAAG
AAAGUUAGAUUUACGCCGAUGAAU
>hsa-mir-6724 MI0022559
CGCUGCGCUUCUGGGCCCGCGGCGGGCGUGGGGCUGCCCGGGCCGGUCGACCAGCGCGCCGUAGC
UCCCGAGGCCCGAGCCGCGACCCGCGG
>hsa-mir-6726 MI0022571
GGGGGCGGGAGCUGGGGUCUGCAGGUUCGCACUGAUGCCUGCUCGCCCUGUCUCCCGCUAG
>hsa-mir-6727 MI0022572
GGGUGCUCGGGGCAGGCGGCUGGGAGCGGCCCUCACAUUGAUGGCUCCUGCCACCUCCUCCGCAG
>hsa-mir-6728 MI0022573
CUAGAUUGGGAUGGUAGGACCAGAGGGGCUUACUGCCCUGUGGGGCUCUCUGGACCCAGUGCCAU
GCUUCUCUGCUCUGCUCUCCCCAG
>hsa-mir-6729 MI0022574
GAGGGUGGGCGAGGGCGGCUGAGCGGCUCCAUCCCCCGGCCUGCUCAUCCCCCUCGCCCUCUCAG
>hsa-mir-6730 MI0022575
CCGAAAGAAAGGUGGAGGGGUUGUCAGAGCUGCGGCUGCUCCACCCUGACACCCCAUCUGCCCUC
AG
>hsa-mir-6731 MI0022576
ACAGGUGGGAGAGCAGGGUAUUGUGGAAGCUCCAGGUGCCAACCACCUGCCUCUAUUCCCCACUC
UCCCCAG
>hsa-mir-6732 MI0022577
AGGCCUAGGGGGUGGCAGGCUGGCCAUCAGUGUGGGCUAACCCUGUCCUCUCCCUCCCAG
>hsa-mir-6733 MI0022578
GUGCUUGGGAAAGACAAACUCAGAGUUCCCUUCUUGUGAGCUCAGUGUCUGGAUUUCCUAG
>hsa-mir-6734 MI0022579
AGAACUUGAGGGGAGAAUGAGGUGGAGAAGCCCAGGUUCUGAAUCCCCUUCCCUCACUCUUCUCU
CAG
>hsa-mir-6735 MI0022580
GCAGCCAGGGCAGAGGGCACAGGAAUCUGAGGUGACUGGCACAGAAGACUCAGGCCUGUGGCUCC
UCCCUCAG
>hsa-mir-6736 MI0022581
CUGAGCUGGGUGAGGGCAUCUGUGGUUUGCUGGCUGCCUCAGCUCCUCUCUACCCACAG
>hsa-mir-6737 MI0022582
UUGGGUUGGGGUGGUCGGCCCUGGAGGGGGUUUGUUUGCUUAUUCCCCUCUGUGCUUCACCCCUA
CCCAG
>hsa-mir-6738 MI0022583
GAAGGCGAGGGGUAGAAGAGCACAGGGGUUCUGAUAAACCCUUCUGCCUGCAUUCUACUCCCAG
>hsa-mir-6739 MI0022584

GAAUGUGGGAAAGAGAAAGAACAAGUAAAAGGAAUUUUCAUUUUCCAGCCCCUAAUUGUUCUGUC
UUUCUCCCAG
>hsa-mir-6740 MI0022585
GAAAGAGUUUGGGAUGGAGAGAGGAGAAACUUGAGGUCUCUGGGAGUUGCUUAAACCAGUUGACC
GUAACCUGGCCAGAGAAUUCUGAUAGUGUCUUCUCUCCUCCCAAACAG
>hsa-mir-6741 MI0022586
AAUGGGUGGGUGCUGGUGGGAGCCGUGCCCUGGCCACUCAUUCGGCUCUCUCCCUCACCCUAG
>hsa-mir-6742 MI0022587
GAGGGAGUGGGGUGGGACCCAGCUGUUGGCCAUGGCGACAACACCUGGGUUGUCCCCUCUAG
>hsa-mir-6743 MI0022588
GGGUAAAGGGGCAGGGACGGGUGGCCCCAGGAAGAAGGGCCUGGUGGAGCCGCUCUUCUCCCUGC
CCACAG
>hsa-mir-6744 MI0022589
UCACGUGGAUGACAGUGGAGGCCUCCUGGAUCUCUAGGUCUCAGGGCCUCUCUUGUCAUCCUGCA
G
>hsa-mir-6745 MI0022590
GGUCCUUGAGGGCAGGAGCUGGGUCUUGGGACUGAUUGCCCCUAGUGGCUAGCCCUGGGUCUAGC
AGCCUAUGGCAGUGUCUGGUAAACACUGGUGGAGUCCUGGGUGGAAGAAGGUCUGGUUCUCA
>hsa-mir-6746 MI0022591
CUUGCCCGGGAGAAGGAGGUGGCCUGGAGAGCUGCUGUCUCCAGCCGCCGCCUGUCUCCACAG
>hsa-mir-6747 MI0022592
UUUGGAGGGGUGUGGAAAGAGGCAGAACAUUCGUUCACUUUCCUGCCUUCCUCUGCACCAG
>hsa-mir-6748 MI0022593
UGGUGUGUGGGUGGGAAGGACUGGAUUUGAAAUGGUCCCACUCCUGACGAUCCUGUCCCUGUCUC
CUACAG
>hsa-mir-6749 MI0022594
GGCCCUCGGGCCUGGGGUUGGGGGAGCUCUGUCCUGUCUCACUCAUUGCUCCUCCCCUGCCUGGC
CCAG
>hsa-mir-6750 MI0022595
GCUGUCAGGGAACAGCUGGGUGAGCUGCUGCCCCAGAGGCCCAGCAGGUGUCCAGAACUCACCCU
CUGCUCCCAG
>hsa-mir-6751 MI0022596
UCUUCUUGGGGGUGAGGUUGGUGUCUGGCCCCAGCAGCCCAGACUGAGCCUCUCUCUCUCCAG
>hsa-mir-6752 MI0022597
AUGGAGGGGGGUGUGGAGCCAGGGGGCCCAGGUCUACAGCUUCUCCCCGCUCCCUGCCCCCAUAC
UCCCAG
>hsa-mir-6753 MI0022598
CACCAGGGCAGAGCAGGGCUGAUCAUCUCACGUCAGAGAGAGGGGAAGGGGCUGCCCAGUGAGCC
CCCACAGGGCUCUACAUCUCCAGCUGGGCCUGGCUGGAGAUCCCAGGGUCCCUGAAGGCCCCCGC
CACCGUUCUGGUCUGUCUCUGCCCUGGCACCCAG
>hsa-mir-6754 MI0022599
GGCUGCCAGGGAGGCUGGUUUGGAGGAGUCUGGUGGCCUGUUCUCUUCACCUGCCUCUGCCUGCA
G
>hsa-mir-6755 MI0022600

UGUUUUAGGGUAGACACUGACAACGUUAUGUGUGGUCUUUAACCUGUUGUCAUGUUUUUUCCCUA
G
>hsa-mir-6756 MI0022601
ACCCUAGGGUGGGGCUGGAGGUGGGGCUGAGGCUGAGUCUUCCUCCCCUUCCUCCCUGCCCAG
>hsa-mir-6757 MI0022602
GGGCUUAGGGAUGGGAGGCCAGGAUGAAGAUUAAUCCCUAAUCCCCAACACUGGCCUUGCUAUCC
CCAG
>hsa-mir-6758 MI0022603
UGGGCUAGAGAGGGGAAGGAUGUGAUGUGAGCAGAUGGUUCUCACUCAUUCUCCUCUGUCCAG
>hsa-mir-6759 MI0022604
UAUUGUUGUGGGUGGGCAGAAGUCUGUUUUCUUCAUGGUUUUCUGACCUUUGCCUCUCCCCUCAG
>hsa-mir-6760 MI0022605
CAGUGCAGGGAGAAGGUGGAAGUGCAGAGUGGGCUCACCUCUCGCCCACACUGUCCCCUUCUCCC
CAG
>hsa-mir-6761 MI0022606
UCUGCUCUGAGAGAGCUCGAUGGCAGGUGCCUCCGUGUUGCCGAACCCUCCUACGCUGCUCUCUC
ACUCCAG
>hsa-mir-6762 MI0022607
AGAGCCGGGGCCAUGGAGCAGCCUGUGUAGACGGGGACCUGCCCUGCAUGGGCACCCCCUCACUG
GCUGCUUCCCUUGGUCUCCAG
>hsa-mir-6763 MI0022608
UUCUCCUGGGGAGUGGCUGGGGAGCAGACAGACCCAACCUCAUGCUCCCCGGCCUCUGCCCCCAG
>hsa-mir-6764 MI0022609
CUGACUCCCAGGGUCUGGUCAGAGUUGCUGAGUGGGUUGAUCUCUGGUCUUUCCUUGACAG
>hsa-mir-6765 MI0022610
GUGAGGCGGGGCCAGGAGGGUGUGUGGCGUGGGUGCUGCGGGGCCGUCAGGGUGCCUGCGGGACG
CUCACCUGGCUGGCCCGCCCAG
>hsa-mir-6766 MI0022611
AUGAGCGGGUGGGAGCAGAUCUUAUUGAGAGUUCCUUCUCCUGCUCCUGAUUGUCUUCCCCCACC
CUCACAG
>hsa-mir-6767 MI0022612
UGAAAUCGCAGACAGGGACACAUGGAGAACGCCCCCACCAGUUCCCACGUGCUUCUCUUUCCGCA
G
>hsa-mir-6768 MI0022613
CCAGGCACACAGGAAAAGCGGGGCCCUGGGUUCGGCUGCUACCCCAAAGGCCACAUUCUCCUGUG
CACACAG
>hsa-mir-6769a MI0022614
AGGCCAGGUGGGUAUGGAGGAGCCCUCAUAUGGCAGUUGGCGAGGGCCCAGUGAGCCCCUCUCUG
CUCUCCAG
>hsa-mir-6769b MI0022706
CUUCCUGGUGGGUGGGGAGGAGAAGUGCCGUCCUCAUGAGCCCCUCUCUGUCCCACCCAUAG
>hsa-mir-6770-1 MI0022615
UAUCCUGAGAAGGCACAGCUUGCACGUGACCUCCUGGGCCUGGCGGCUGUGUCUUCACAG
>hsa-mir-6771 MI0022616

GGUGCCUCGGGAGGGCAUGGGCCAGGCCACAUAAUGAGCCAAACCCCUGUCUACCCGCAG
>hsa-mir-6772 MI0022617
AGGCCUGGGUGUAGGCUGGAGCUGAGGACUGAGGCUCACCUUGCUCCUGACUCUGUGCCCACAG
>hsa-mir-6773 MI0022618
GGGAGUUGGGCCCAGGAGUAAACAGGAUUAGUGCUUAUGCUAAGAUGUCUUCACUGUCACUUCUC
UGCCCAUAG
>hsa-mir-6774 MI0022619
UGUGCACUUGGGCAGGAGGGACCCUGUAUGUCUCCCCGCAGCACCGUCAUCGUGUCCCUCUUGUC
CACAG
>hsa-mir-6775 MI0022620
GAACCUCGGGGCAUGGGGGAGGGAGGCUGGACAGGAGAGGGCUCACCCAGGCCCUGUCCUCUGCC
CCAG
>hsa-mir-6776 MI0022621
CGGGCUCUGGGUGCAGUGGGGGUUCCCACGCCGCGGCAACCACCACUGUCUCUCCCCAG
>hsa-mir-6777 MI0022622
UCAAGACGGGGAGUCAGGCAGUGGUGGAGAUGGAGAGCCCUGAGCCUCCACUCUCCUGGCCCCCA
G
>hsa-mir-6778 MI0022623
GUUCAAGUGGGAGGACAGGAGGCAGGUGUGGUUGGAGGAAGCAGCCUGAACCUGCCUCCCUGACA
UUCCACAG
>hsa-mir-6779 MI0022624
GAGCUCUGGGAGGGGCUGGGUUUGGCAGGACAGUUUCCAAGCCCUGUCUCCUCCCAUCUUCCAG
>hsa-mir-6780a MI0022625
GACACUUGGGAGGGAAGACAGCUGGAGAGUAUGGUCACAGCAGCAUCCUCCUCUGUUUUCUUUCC
UAG
>hsa-mir-6780b MI0022681
CAGCCUGGGGAAGGCUUGGCAGGGAAGACACAUGAGCAGUGCCUCCACUUCACGCCUCUCCCUUG
UCUCCUUUCCCUAG
>hsa-mir-6781 MI0022626
AACCCCGGGCCGGAGGUCAAGGGCGUCGCUUCUCCCUAAUGUUGCCUCUUUUCCACGGCCUCAG
>hsa-mir-6782 MI0022627
UGGGGUAGGGGUGGGGGAAUUCAGGGGUGUCGAACUCAUGGCUGCCACCUUUGUGUCCCCAUCCU
GCAG
>hsa-mir-6783 MI0022628
CCUGUUAGGGGAAAAGUCCUGAUCCGGGAACCCACAGCCCCGUUCCUGGGCUUCUCCUCUGUAG
>hsa-mir-6784 MI0022629
UACAGGCCGGGGCUUUGGGUGAGGGACCCCCGGAGUCUGUCACGGUCUCACCCCAACUCUGCCCC
AG
>hsa-mir-6785 MI0022630
CUCCCUGGGAGGGCGUGGAUGAUGGUGGGAGAGGAGCCCCACUGUGGAAGUCUGACCCCCACAUC
GCCCCACCUUCCCCAG
>hsa-mir-6786 MI0022631
GCCGGGUGGGGCGGGGCGGCCUCAGGAGGGGCCCAGCUCCCCUGGAUGUGCUGCGGUGGGGCCGG
AGGGGCGUCACGUGCACCCAAGUGACGCCCCUUCUGAUUCUGCCUCAG

>hsa-mir-6787 MI0022632
UCGGCUGGCGGGGGUAGAGCUGGCUGCAGGCCCGGCCCCUCUCAGCUGCUGCCCUCUCCAG
>hsa-mir-6788 MI0022633
GACGGCUGGGAGAAGAGUGGUGAAGAAGAGUAUUGAUUGUGCUGUUCGCCACUUCCCUCCCUGCA
G
>hsa-mir-6789 MI0022634
CGAGGUAGGGGCGUCCCGGGCGCGCGGGCGGGUCCCAGGCUGGGCCCCUCGGAGGCCGGGUGCUC
ACUGCCCCGUCCCGGCGCCCGUGUCUCCUCCAG
>hsa-mir-6790 MI0022635
GUGAGUGUGGAUUUGGCGGGGUUCGGGGGUUCCGACGGCGACCUCGGCGACCCCUCACUCACC
>hsa-mir-6791 MI0022636
CCAGACCCCUGGGGCUGGGCAGGCGGAAAGAGGUCUGAACUGCCUCUGCCUCCUUGGUCUCCGGC
AG
>hsa-mir-6792 MI0022637
GUAAGCAGGGGCUCUGGGUGAUGUGAGGAGCAACAGGCACCCUCCUCCACAGCCCCUGCUCAUUC
CU
>hsa-mir-6793 MI0022638
GUCACUGUGGGUUCUGGGUUGGGGUGAUACACAAGCCUGACCCUCCCCAACCCCUGCCCGCAG
>hsa-mir-6794 MI0022639
GGGCGCAGGGGGACUGGGGGUGAGCAGGCCCAGAACCCAGCUCGUGCUCACUCUCAGUCCCUCCC
UAG
>hsa-mir-6795 MI0022640
AGGGUUGGGGGGACAGGAUGAGAGGCUGUCUUCAUUCCCUCUUGACCACCCCUCGUUUCUUCCCC
CAG
>hsa-mir-6796 MI0022641
UUACCUUGUGGGGUUGGAGAGCUGGCUGGUCCAGCCCCUCAGAAGCUCUCCCCUCCCCGCAG
>hsa-mir-6797 MI0022642
CAGCCAGGAGGGAAGGGGCUGAGAACAGGACCUGUGCUCACUGGGGCCUGCAUGACCCUUCCCUC
CCCACAG
>hsa-mir-6798 MI0022643
GGCAGCCAGGGGGAUGGGCGAGCUUGGGCCCAUUCCUUUCCUUACCCUACCCCCCAUCCCCCUGU
AG
>hsa-mir-6799 MI0022644
GAGGAGGGGAGGUGUGCAGGGCUGGGGUCACUGACUCUGCUUCCCCUGCCCUGCAUGGUGUCCCC
ACAG
>hsa-mir-6800 MI0022645
ACCUGUAGGUGACAGUCAGGGGCGGGGUGUGGUGGGGCUGGGGCUGGCCCCCUCCUCACACCUCU
CCUGGCAUCGCCCCCAG
>hsa-mir-6801 MI0022646
UGGCCUGGUCAGAGGCAGCAGGAAAUGAGAGUUAGCCAGGAGCUUUGCAUACUCACCCCUGCCAC
UCACUGGCCCCCAG
>hsa-mir-6802 MI0022647
GAGGGCUAGGUGGGGGGCUUGAAGCCCCGAGAUGCCUCACGUCUUCACCCCUCUCACCUAAGCAG
>hsa-mir-6803 MI0022648

CUCCUCUGGGGGUGGGGGGCUGGGCGUGGUGGACAGCGAUGCAUCCCUCGCCUUCUCACCCUCAG
>hsa-mir-6804 MI0022649
GGAUGUGAGGGUGUCAGCAGGUGACGGUGGGGGCCACGCUGACAGCCGCACCUGCCUCUCACCCA
CAG
>hsa-mir-6805 MI0022650
UGGCCUAGGGGGCGGCUUGUGGAGUGUAUGGGCUGAGCCUUGCUCUGCUCCCCCGCCCCCAG
>hsa-mir-6806 MI0022651
UGCUCUGUAGGCAUGAGGCAGGGCCCAGGUUCCAUGUGAUGCUGAAGCUCUGACAUUCCUGCAG
>hsa-mir-6807 MI0022652
GUGAGCCAGUGGAAUGGAGAGGCUGUGGGCAGGGGGAGAUGUGAAGGAAAGAACUAGGACCCAUU
CAUCCACUGCAUUCCUGCUUGGCCCAG
>hsa-mir-6808 MI0022653
GGGGCCAGGCAGGGAGGUGGGACCAUGGGGGCCUUGCUGUGUGACCACCGUUCCUGCAG
>hsa-mir-6809 MI0022654
AAUGUUGGCAAGGAAAGAAGAGGAUCAUGUUUGCCCCCGUGGACAGCUCUCUGGUGUGCUCCUCC
UGCCAUCCUGCCCACCCCUGCAUAAUGCUGCUUCUCUUCUCUCCUUCCCAG
>hsa-mir-6810 MI0022655
CUGGGAUGGGGACAGGGAUCAGCAUGGCACAGAUCCAAUACCUUCUGUCCCCUGCUCCCUUGUUC
CCCAG
>hsa-mir-6811 MI0022656
UAUGCAGGCCUGUGUACAGCACUCAGGCAGUGCCAUGAGCCUGUGCUUGUCCCUGCAG
>hsa-mir-6812 MI0022657
UGAGGAUGGGGUGAGAUGGGGAGGAGCAGCCAGUCCUGUCUCACCGCUCUUCCCCUGACCCCAG
>hsa-mir-6813 MI0022658
GUAGGCAGGGGCUGGGGUUUCAGGUUCUCAGUCAGAACCUUGGCCCCUCUCCCCAG
>hsa-mir-6814 MI0022659
UUUCCUCCCAAGGGUGAGAUGCUGCCACCCAGCCCUGCAGAGCCCCUGACUCGCAUCCUUCCCUU
GGCAG
>hsa-mir-6815 MI0022660
CACUGUAGGUGGCGCCGGAGGAGUCAUUUCCCAUCACUAAUGGCUUCUCUUGCACACCCAG
>hsa-mir-6816 MI0022661
CCGAGUGGGGCGGGGCAGGUCCCUGCAGGGACUGUGACACUGAAGGACCUGCACCUUCGCCCACA
G
>hsa-mir-6817 MI0022662
AGGAUUCUGCCAUAGGAAGCUUGGAGUGGAACUGACCUGCCCCCUUUCUCUCUGACUCCAUGGCA
G
>hsa-mir-6818 MI0022663
CUAUUUUGUGUGAGUACAGAGAGCAUCUGAAUGGGUACAGUUGUUGUCUCUUGUUCCUCACACAG
>hsa-mir-6819 MI0022664
GAGGGUUGGGGUGGAGGGCCAAGGAGCUGGGUGGGGUGCCAAGCCUCUGUCCCCACCCCAG
>hsa-mir-6820 MI0022665
CCUUCUGCGGCAGAGCUGGGGUCACCAGCCCUCAUGUACUUGUGACUUCUCCCCUGCCACAG
>hsa-mir-6821 MI0022666
GUGCGUGGUGGCUCGAGGCGGGGGUGGGGGCCUCGCCCUGCUUGGGCCCUCCCUGACCUCUCCGC

UCCGCACAG
>hsa-mir-6822 MI0022667
UGGCCCAGGGAACCAGUUGGGGCUUCCGCUCUGCAGAGGCUCUAACUGGCUUUCCCUGCAG
>hsa-mir-6823 MI0022668
CAAGGUCAGGGUUGGUAGGGGUUGCUGUUGCUGUGAAAGCUGAGCCUCUCCUUCCCUCCAG
>hsa-mir-6824 MI0022669
GAGGUGUAGGGGAGGUUGGGCCAGGGAUGCCUUCACUGUGUCUCUCUGGUCUUGCCACCCCAG
>hsa-mir-6825 MI0022670
GGGCAUGGGGAGGUGUGGAGUCAGCAUGGGGCUAGGAGGCCCCGCGCUGACCCGCCUUCUCCGCA
G
>hsa-mir-6826 MI0022671
CUUGGUCAAUAGGAAAGAGGUGGGACCUCCUGGCUUUUCCUCUGCAGCAUGGCUCGGACCUAGUG
CAAUGUUUAAGCUCCCCUCUCUUUCCUGUUCAG
>hsa-mir-6827 MI0022672
UCUGGUGGGAGCCAUGAGGGUCUGUGCUGUCUCUGAGCACCGUCUCUUCUGUUCCCCAG
>hsa-mir-6828 MI0022673
GGCUCAGGAAGCAAGAGAACCCUGUGGUCUAACCUUUCCCAUCUGCUCUCUUGUUCCCAG
>hsa-mir-6829 MI0022674
CAGCGUGGGCUGCUGAGAAGGGGCAGGGUCCUCCAGCUCAUUCCUCCUGCCUCCUCCGUGGCCUC
AG
>hsa-mir-6830 MI0022675
GUGCCCCAAGGAAGGAGGCUGGACAUCCCUCAUCUGUUUCUCACUGGUGUCUUUCUUCUCUCCCU
UGCAG
>hsa-mir-6831 MI0022676
GUAGGUAGAGUGUGAGGAGGAGGUCUGAGCCCAUGUGUGGACCUAGGUCUGCUGUUAAACUGACU
AACUCCCACUCUACAG
>hsa-mir-6832 MI0022677
GGUGGAGUAGAGAGGAAAAGUUAGGGUCAGUGGCAGAGCCAGGCAGAUGCUGACCCUUUUUCUCU
UUCCCAG
>hsa-mir-6833 MI0022678
AAACGGUGUGGAAGAUGGGAGGAGAAAAAUCCCUGUUAACUUUCUCUCUCCACUUCCUCAG
>hsa-mir-6834 MI0022679
GUGAGGGACUGGGAUUUGUGGGGCGAGGAGGGACCUGUACUAGCCAUGGUUCUGAUCACAUAUGU
CCCAUCCCUCCAUCAG
>hsa-mir-6835 MI0022680
UGAUGAGGGGGUAGAAAGUGGCUGAAGCGAGAUGUUUGUCUAAAAGCACUUUUCUGUCUCCCAG
>hsa-mir-6836 MI0022682
GGCUCCGCAGGGCCCUGGCGCAGGCAUCCAGACAGCGGGCGAAUGCCUCCCCCGGCCCCGCAG
>hsa-mir-6837 MI0022683
GUGGGACCAGGGCCAGCAGGGAAUGUCAGGGCCACCCCUGACCUUCACUGUGACUCUGCUGCAG
>hsa-mir-6838 MI0022684
CAGGGAAGCAGCAGUGGCAAGACUCCUAGGUCACGGAAGUCCUGCUUCUGUUGCAG
>hsa-mir-6839 MI0022685
UGUAGUCUGGAUUGAAGAGACGACCCAAGCAGGCUUUGUGUGAGCAGUGAGGCUAUUUAUUCACU

UGGGUGCGAGCUCACACGAAGCCUGCUUGGGUUUUCUCUUCAAUCCAG
>hsa-mir-6840 MI0022686
UGACCACCCCCGGGCAAAGACCUGCAGAUCCCCUGUUAGAGACGGGCCCAGGACUUUGUGCGGGG
UGCCCA
>hsa-mir-6841 MI0022687
GUGUUUAGGGUACUCAGAGCAAGUUGUGAAACACAGGUGUUUUUUAACCUCACCUUGCAUCUGCA
UCCCCAG
>hsa-mir-6842 MI0022688
AGCCCUGGGGGUGGUCUCUAGCCAAGGCUCUGGGGUCUCACCCUUGGCUGGUCUCUGCUCCGCAG
>hsa-mir-6843 MI0022689
CCCCCAUUUCCUCAGGAAUAAAAGUGCAGCAGUGCCUGCUGUGGGGACAGCUGAGGGCAGUGAGG
CCCUGGGGAGCUGCUGCAGGCAGCAGGUGGGCGGGACGCCAGCAGGCUGUCUAGCUGUUCCCAUG
AUGGUCUCCUGUUCUCUGCAG
>hsa-mir-6844 MI0022690
GAACUUAAGAAUUUUGUAGAAAUCAAGCUAUUUGCUAAAAGUUCUUUGUUUUUAAUUCACAG
>hsa-mir-6845 MI0022691
AACUGCGGGGCCAGAGCAGAGAGCCCUUGCACACCACCAGCCUCUCCUCCCUGUGCCCCAG
>hsa-mir-6846 MI0022692
CAGGCUGGGGGCUGGAUGGGGUAGAGUAGGAGAGCCCACUGACCCCUUCUGUCUCCCUAG
>hsa-mir-6847 MI0022693
GACCCACAGAGGACAGUGGAGUGUGAGCUGGAAGGAGUGGGCCUGGCUCAUGUGUCUGUCCUCUU
CCAG
>hsa-mir-6848 MI0022694
GUCCCUGGGGGCUGGGAUGGGCCAUGGUGUGCUCUGAUCCCCCUGUGGUCUCUUGGCCCCCAGGA
ACUCC
>hsa-mir-6849 MI0022695
CCUGGGAGUGGAUAGGGGAGUGUGUGGAGAGAGCACUGAGCCUGCCCACCAGCCUGUGUCCACCU
CCAG
>hsa-mir-6850 MI0022696
GUGCGGAACGCUGGCCGGGGCGGGAGGGGAAGGGACGCCCGGCCGGAACGCCGCACUCACG
>hsa-mir-6851 MI0022697
CAGGGAGGAGGUGGUACUAGGGGCCAGCAACCUGAUUACCCCUCUUUGGCCCUUUGUACCCCUCC
AG
>hsa-mir-6852 MI0022698
UGCUGCCCUGGGGUUCUGAGGACAUGCUCUGACUCCCCUGAUGUCCUCUGUUCCUCAGGUGCUGG
G
>hsa-mir-6853 MI0022699
GGGAAAGCGUGGGAUGUCCAUGAAGUCAGGUGAUGGUGAUAAGGUCAAGGCCUGUUCAUUGGAAC
CCUGCGCAG
>hsa-mir-6854 MI0022700
AAAGCAAGCUCAGGUUUGAGAACUGCUGAUGUCAUCAGUCAUAACUUCUGCGUUUCUCCUCUUGA
GCAG
>hsa-mir-6855 MI0022701
GCUGCUUGGGGUUUGGGGUGCAGACAUUGCCAGAGGAUGGGCAGCAGACUGACCUUCAACCCCAC

AG
>hsa-mir-6856 MI0022702
UGGAAAAGAGAGGAGCAGUGGUGCUGUGGCAGUGGCAGAGGUCGCUACAGCCCUGUGAUCUUUCC
AG
>hsa-mir-6857 MI0022703
GCUUGUUGGGGAUUGGGUCAGGCCAGUGUUCAAGGGCCCCUCCUCUAGUACUCCCUGUUUGUGUU
CUGCCACUGACUGAGCUUCUCCCCACAG
>hsa-mir-6858 MI0022704
GUGAGGAGGGGCUGGCAGGGACCCCUCCAAGUUGGGGACGGCAGCCAGCCCCUGCUCACCCCUCG
CC
>hsa-mir-6859-1 MI0022705
UGUGGGAGAGGAACAUGGGCUCAGGACAGCGGGUGUCAGCUUGCCUGACCCCCAUGUCGCCUCUG
UAG
>hsa-mir-6860 MI0022707
GUUAAGCAUUGGGGAGUUUGGAGUCGGUGGGUGGAGCCAAACUGGGCAGGGCUGUGGUGAGUGAG
U
>hsa-mir-6861 MI0022708
GAGGCACUGGGUAGGUGGGGCUCCAGGGCUCCUGACACCUGGACCUCUCCUCCCCAGGCCCACA
>hsa-mir-6862-1 MI0022709
CGAAGCGGGCAUGCUGGGAGAGACUUUGUGAUUUGUCUCCAAAGCCUCACCCAGCUCUCUGGCCC
UCUAG
>hsa-mir-6863 MI0022710
AUUUAUGAGGCAGCAGAGUCUACAAGUAAAUCAUGAAUCCAGUUGAAAAUGUUAAUGAGGCCAUA
GACGUGGUGAAGGAUUGAGUGACCU
>hsa-mir-6864 MI0022711
GAAAGUUGAAGGGACAAGUCAGAUAUGCCAUUAUGUGGGUCAUGAGGUGGUGAGACUUCUCUCCC
UUCAG
>hsa-mir-6865 MI0022712
AUCCAUAGGUGGCAGAGGAGGGACUUCAGAUGCUUCAUGACACCCUCUUUCCCUACCGCCUACAG
>hsa-mir-6866 MI0022713
CCAUUUUAGAGGCUGGAAUAGAGAUUCUUGAGGCUUGGAAGAGUAAGGAUCCCUUUAUCUGUCCU
CUAG
>hsa-mir-6867 MI0022714
CCCGGUGUGUGUGUAGAGGAAGAAGGGAAGCUGGGAACCUGACUGCCUCUCCCUCUUUACCCACU
AG
>hsa-mir-6868 MI0022715
CCAGGACUGGCAGAACACUGAAGCAGCAGGCACCUGCUUCCUUCUGUUGUCUGUGCAG
>hsa-mir-6869 MI0022716
GUGAGUAGUGGCGCGCGGCGGCUCGGAGUACCUCUGCCGCCGCGCGCAUCGGCUCAGCAUGC
>hsa-mir-6870 MI0022717
CAAGGUGGGGGAGAUGGGGGUUGAACUUCAUUUCUCAUGCUCAUCCCCAUCUCCUUUCAG
>hsa-mir-6871 MI0022718
CUCCUCAUGGGAGUUCGGGGUGGUUGCUGGAGGUCAGCACCCUGUGGCUCCCACAG
>hsa-mir-6872 MI0022719

GUGGGUCUCGCAUCAGGAGGCAAGGCCAGGACCCGCUGACCCAUGCCUCCUGCCGCGGUCAG
>hsa-mir-6873 MI0022720
CCCAGCAGAGGGAAUACAGAGGGCAAUCAGGACUGGGUCAUUCUCUCUGUCUUUCUCUCUCAG
>hsa-mir-6874 MI0022721
GCCACAUGGAGCUGGAACCAGAUCAGGCUUUAAUGUUUGAAGUAAUGUCAGUUCUGCUGUUCUGA
CUCUAG
>hsa-mir-6875 MI0022722
GAGUCUGAGGGACCCAGGACAGGAGAAGGCCUAUGGUGAUUUGCAUUCUUCCUGCCCUGGCUCCA
UCCUCAG
>hsa-mir-6876 MI0022723
AGUUGCAGGAAGGAGACAGGCAGUUCAGGAGGUGGCACUGCUGUGUGUGAGCUGUCUGUGUUUUC
CUUCUCAG
>hsa-mir-6877 MI0022724
AGUUCAGGGCCGAAGGGUGGAAGCUGCUGGUGCUCAUCUCAGCCUCUGCCCUUGGCCUCCCCAG
>hsa-mir-6878 MI0022725
AUGAGAGGGAGAAAGCUAGAAGCUGAAGAUUCUGAAAAUCACUAACUGGCCUCUUCUUUCUCCUA
G
>hsa-mir-6879 MI0022726
CAGAGCAGGGCAGGGAAGGUGGGAGAGGGGCCCAGCUGACCCUCCUGUCACCCGCUCCUUGCCCA
G
>hsa-mir-6880 MI0022727
GAGGGUGGUGGAGGAAGAGGGCAGCUCCCAUGACUGCCUGACCGCCUUCUCUCCUCCCCCAG
>hsa-mir-6881 MI0022728
UGCACUGGGGUAAGGAUAGGAGGGUCAGGUCUGCAGCCUUGUAUCUGCUGAUCCUCUUUCGUCCU
UCCCACUCCAG
>hsa-mir-6882 MI0022729
GGCUUUACAAGUCAGGAGCUGAAGCAGCUGGAAUUCAAGCCCUGCUGCCUCUCCUCUUGCCUGCA
G
>hsa-mir-6883 MI0022730
CAGACAGGGAGGGUGUGGUAUGGAUGUGUUGACCCCUGAAGUGGUUCUGAUGACCUUUCCCUAUC
UCACUCUCCUCAG
>hsa-mir-6884 MI0022731
CCCGCAGAGGCUGAGAAGGUGAUGUUGGCUCAAGAAAGGGAGAUAGAUGGUAGCCCAUCACCUUU
CCGUCUCCCCUAG
>hsa-mir-6885 MI0022732
CCUGGAGGGGGGCACUGCGCAAGCAAAGCCAGGGACCCUGAGAGGCUUUGCUUCCUGCUCCCCUA
G
>hsa-mir-6886 MI0022733
CUUGGCCCGCAGGUGAGAUGAGGGCUCCUGGCGCUGAUGCCCUUCUCUCCUCCUGCCUCAG
>hsa-mir-6887 MI0022734
GAGAAUGGGGGGACAGAUGGAGAGGACACAGGCUGGCACUGAGGUCCCCUCCACUUUCCUCCUAG
>hsa-mir-6888 MI0022735
GUGGGAAGGAGAUGCUCAGGCAGAUCUGUCUCUGAUUGUUUCCAAGAUCUGUCUCGAUUGUUUCC
AG

>hsa-mir-6889 MI0022736
CUGUGUCGGGGAGUCUGGGGUCCGGAAUUCUCCAGAGCCUCUGUGCCCCUACUUCCCAG
>hsa-mir-6890 MI0022737
UGGGCCAUGGGGUAGGGCAGAGUAGGGCUGGAUGGUAGGGCCCACUGCCUAUGCCCCACAG
>hsa-mir-6891 MI0022738
GUAAGGAGGGGGAUGAGGGGUCAUAUCUCUUCUCAGGGAAAGCAGGAGCCCUUCAGCAGGGUCAG
GGCCCCUCAUCUUCCCCUCCUUUCCCAG
>hsa-mir-6892 MI0022739
GUAAGGGACCGGAGAGUAGGAAAAGCAGGGCUCAGGGCCAGAGAGACUGGGCAUAGAACUAAGGA
GGAUGGUGUCCUCCUGACUGCAUCUCUCUUCCCUCUCCCACCCCUUGCAG
>hsa-mir-6893 MI0022740
CCGGGCAGGCAGGUGUAGGGUGGAGCCCACUGUGGCUCCUGACUCAGCCCUGCUGCCUUCACCUG
CCAG
>hsa-mir-6894 MI0022741
CAAGAAGGAGGAUGGAGAGCUGGGCCAGACAUGCUCUUGCCUGCCCUCUUCCUCCAG
>hsa-mir-6895 MI0022742
CAGCUCAGGGCCAGGCACAGAGUAAGCAUCAAUAGCAUUGGCAAGUUGAACUGAGCUGUCUCUCG
CCCUUGGCCUUAG
>hsa-mir-7106 MI0022957
GCUUCUGGGAGGAGGGGAUCUUGGGAGUGAUCCCAACAGCUGAGCUCCCUGAAUCCCUGUCCCAG
>hsa-mir-7107 MI0022958
UGCCGUCGGCCUGGGGAGGAGGAAGGGCAAGUCCAAAGGUAUACAGUUGGUCUGUUCAUUCUCUC
UUUUUGGCCUACAAG
>hsa-mir-7108 MI0022959
GUGUGGCCGGCAGGCGGGUGGGCGGGGGCGGCCGGUGGGAACCCCGCCCCGCCCCGCGCCCGCAC
UCACCCGCCCGUCUCCCCACAG
>hsa-mir-7109 MI0022960
GUCUCCUGGGGGGAGGAGACCCUGCUCUCCCUGGCAGCAAGCCUCUCCUGCCCUUCCAGAUUAGC
>hsa-mir-7110 MI0022961
GGGGCUGGGGGUGUGGGGAGAGAGAGUGCACAGCCAGCUCAGGGAUUAAAGCUCUUUCUCUCUCU
CUCUCUCCCACUUCCCUGCAG
>hsa-mir-7111 MI0022962
CUGGGGGAGGAAGGACAGGCCAUCUGCUAUUCGUCCACCAACCUGACUUGAUCCUCUCUUCCCUC
CUCCCAG
>hsa-mir-7112-1 MI0022963
ACGGGCAGGGCAGUGCACCCUGCAGGUGAGAGCGGGAACACCUGCAUCACAGCCUUUGGCCCUAG
>hsa-mir-7113 MI0022964
CUCCAGGGAGACAGUGUGUGAGGCCUCUUGCCAUGGCCUCCCUGCCCGCCUCUCUGCAG
>hsa-mir-7114 MI0022965
UCCGCUCUGUGGAGUGGGGUGCCUGUCCCCUGCCACUGGGUGACCCACCCCUCUCCACCAG
>hsa-mir-7150 MI0023610
CACGGUGUCCCCUGGUGGAACCUGGCAGGGGGAGAGGUAAGGUCUUUCAGCCUCUCCAAAGCCCA
UGGUCAGGUACUCAGGUGGGGGAGCCCUG
>hsa-mir-7151 MI0023611

GAUCCAUCUCUGCCUGUAUUGGCUUGGAUUCUGCAAAGCCUACAGGCUGGAAUGGGCUCA
>hsa-mir-7152 MI0023612
UUUCCUGUCCUCCAACCAGACCAUGCCACAUCCGUCUGGUCCUGGACAGGAGGC
>hsa-mir-7153 MI0023613
UGAGAACUGACAAAUGUGGUAGGUGGUCGAAGUCAUCCACACCAUGGACGGUUUACC
>hsa-mir-7154 MI0023614
UUCAUGAACUGGGUCUAGCUUGGAGCCUUGGUAGGGAAGCAAGCUGAGGAGAUAGGAGGACAAGU
UGUGGGAU
>hsa-mir-7155 MI0023615
UCUGGGGUCUUGGGCCAUCUGGUUGUGACAUCACUGAUGGCCCAAGACCUCAGACC
>hsa-mir-7156 MI0023616
UUGUUCUCAAACUGGCUGUCAGAGUGUGCAUGGCAGGCUGCAGCCACUUGGGGAACUGGU
>hsa-mir-7157 MI0023617
UCAGCAUUCAUUGGCACCAGAGAUGAAAUUGGGACUCCUCUGUGCUACUGGAUGAAGAGU
>hsa-mir-7158 MI0023618
GGCUCAAUCUCUGGUCCUGCAGCCUUCUGCCUUUGGCUUUCUGAAGCGAGCUGAACUAGAGAUUG
GGCCCA
>hsa-mir-7159 MI0023620
UUCAACAAGGGUGUAGGAUGGCCAGGGAAGUAUGCCGGGCCUUGGCAUUUCUAUGUUAGUUGGAA
G
>hsa-mir-7160 MI0023621
UGCUGAGGUCCGGGCUGUGCCCCGUACCGGACAGGGCCCUGGCUUUAGCAGA
>hsa-mir-7161 MI0023619
UAAAGACUGUAGAGGCAACUGGUGUUCUCACGCAAAGUGGCCAGGGUGUGGGAGACUAGAUCUUU
GACUCUGGCAGUCUCCAGG
>hsa-mir-7162 MI0023623
UGCUUCCUUUCUCAGCUGAACCCCUGCCCCUGGAGCCCCCAGCAGGGCCCUCUGAGGUGGAACAG
CAGC
>hsa-mir-7515 MI0024354
CUUUUAUCAUUUCAUUUAUGCCUGUUAGCAUGAAAAAAGAGGCUGAGAAGGGAAGAUGGUGACAA
GG
>hsa-mir-7641-1 MI0024975
UCUCGUUUGAUCUCGGAAGCUAAGCAGGGUUGGGCCUGGUUAGUACUUGGAUGGGAAACUU
>hsa-mir-7702 MI0025238
CUUAGACUGCCAGACUCCCUGAGGACAUGCAGUUUCAGGGAGUCUGGUAGUCUAAGUGU
>hsa-mir-7703 MI0025239
UUAGGGGAGGUGGAUGAGUGGGGGCAGGCAGGGGAUUGGCUAAGAACCUGACUCUUGCACUCUGG
CCUUCUCCCAGG
>hsa-mir-7704 MI0025240
CGGGGUCGGCGGCGACGUGCUCAGCUUGGCACCCAAGUUCUGCCGCUCCGACGCCCGGC
>hsa-mir-7705 MI0025241
AAUAGCUCAGAAUGUCAGUUCUGUUUUAAGUAACAGAAUUGAUAACUGAGCAAGGAA
>hsa-mir-7706 MI0025242
UGGAGCUGUGUGCAGGGCCAGCGCGGAGCCCGAGCAGCCGCGGUGAAGCGCCUGUGCUCUGCCGA

GA
>hsa-mir-7843 MI0025510
GUAGACAGGAUGAGGGCAGAGCCAGCUUCCUGAUCUGAUGGAAGUCAUGAAGCCUUCUCUGCCUU
ACGCUUGGCUCUGC
>hsa-mir-7844 MI0025514
UGAUUGUGCGGACUCGUGUGUAAGAAAAACUAGGACUGUGUGGUGUAAAUAACUACAAUUCUCUU
AACUCCGUAGCAGUUGCCAACUCAGUCCUUGUACUUCGUUAACACGAAUCUGUUUCA
>hsa-mir-7845 MI0025515
GCAAGGGACAGGGAGGGUCGUGGCGACACUCGCGCCAGCUCCCGGGACGGCUGGGCUCGGGCUGG
UCGCCGACCUCCGACCCUCCACUAGAUGCCUGGC
>hsa-mir-7846 MI0025516
GCCCCGCCGCCUGGCCUCUGGCCCGCUGGGGCGCGGGCUUUCGCUUUCAGUCGAGGGCUAGCGAG
CGCAGCGGAGCCUGGAGAGAAGGCGCUGGGC
>hsa-mir-7847 MI0025517
GUGUCGGCUGUGGCGUGACUGUCCCUCUGUGUCCCCCACUAGGCCCACUGCUCAGUGGAGCGUGG
AGGACGAGGAGGAGGCCGUCCACGAGCAAUGCCAGCAU
>hsa-mir-7848 MI0025518
GCUGGGGCUGGGUGGGUGUGGCAGGCCCACCUUGGGUAUGCAAAGCUCUGACAGUGUUUCACUUG
CUACCCUCGGUCUGCUUACCACACUCCCAGUUCUGC
>hsa-mir-7849 MI0025519
GACUUUAUAUUGUUGAACAUCAGACUCAAGGCCAACAACUGACUACUUCGAAAAUGGGAAGUAGA
CAAUUGUUGAUCUUGGGCCUGAUGUUCAACAAUAUAAAGUU
>hsa-mir-7850 MI0025520
GGUCAGAAGUUUGGACAUAGUGUGGCUGGGUCCUCUGUGCAGGGUCUUACCCAGCCACACUACAU
CCAAACUUCUGACC
>hsa-mir-7851 MI0025521
UGGCUCACUGCAGCCUCCCCGCCCCCUCAGGUGAUCCUCCCACCUCAUCCUCCCAAGUAGCUGGG
AAUACAGGUGUGUGCCACCAUGCCUCUACAAGCUACCUGGGAGACUGAGGUUGGAAGAUUGCUUG
AGCCUAGGAGGUCGAGGCGACAGUGAGCCA
>hsa-mir-7852 MI0025522
UAAAUGCCUUUGACUACUACAUACAUAUGCCUACUACUACUUUCAUAUGUAGGCAUAUGUAUGUA
GUAGUCAAAGGCAUUUA
>hsa-mir-7853 MI0025523
UCUCACACUUCAAUAGGCAUUGUCUGGGGUCUUAUUCAAAUGCAGAUCCUGACUUCACUGGUGCA
GUAAGAAGUCAGAGAAUCUGCAUCUGUAACAAGCUCCCAGGUGAUGAUGGUGCUGCUGGUGUGCA
GA
>hsa-mir-7854 MI0025524
UUCCUUCCAUCUCCAUCACCUUGAGCAUCUUCUGGGCAGCUGAGGUGACCGCAGAUGGGAAGGAA
>hsa-mir-7855 MI0025525
GCUUGGUGAGGACCCCAAGCUCGGCACUAUUGUGACUGUGCUUUUGGCUUCCUUUCCAGGC
>hsa-mir-7856 MI0025526
UGUUUUAAGGACACUGAGGGAUCCUUUGUUCUGGAUCCCUCAGUGUCCUUAAAACA
>hsa-mir-7973-1 MI0025748
UGUGACCCUAGAAUAAUUACCCAACGUCUCUGAGUCUCGGUUUUGAGAGGGUGAGUAAUUAUUCU

AGGGUCACACA
>hsa-mir-7973-2 MI0025749
UGUGACCCUAGAAUAAUUACUCACCCUCUCAAAACCGAGACUCAGAGACGUUGGGUAAUUAUUCU
AGGGUCACACA
>hsa-mir-7974 MI0025750
GCUCGGCCCCCACAGCGAAACGGCCGCCUAAACCACCCAGGCCUUAUGGCUUCAUAGGCUGUGAU
GCUCUCCUGAGCCC
>hsa-mir-7975 MI0025751
GUGCAAAGAGCAGGAGGACAGGGGAUUUAUCUCCCAAGGGAGGUCCCCUGAUCCUAGUCACGGCA
CCA
>hsa-mir-7976 MI0025752
UGCCCUGAGACUUUUGCUCUAAUAAUUUAUUCUAAUAAUAAUUUAGAUCAAAAGCCUCAGGGCAG
A
>hsa-mir-7977 MI0025753
UUCCCAGCCAACGCACCAAAAAUGAUAUGGGUCUGUUGUCUGGAGAAAC
>hsa-mir-7978 MI0025754
UCUGGUGUAUAGCGUUGCUCAAGUCCUCUAUUUCUUUGAACAACAGUAUACACCAAAUA
>hsa-mir-8052 MI0025888
UGGAGGGCUGCGGGACUGUAGAGGGCAUGAGCUCAGGAGCUCAGGCCAGCUCAUGGUGCAAGGCC
UCUG
>hsa-mir-8053 MI0025889
GCUUUUCCACUGGCGAUUUUGGAACUCAAUGGCAGAAAUGUCAAGAAGAGUUUUAUCCUUUGCCG
AAAGAGAAAA
>hsa-mir-8054 MI0025890
CAGCAAUGCAGAAAGUACAGAUCGGAUGGGUUCAAUAGAUUGCUUUAGCUGAAGCAAGCCUUCCC
AUUGUACAUAUCUCAUUGUCU
>hsa-mir-8055 MI0025891
UUACGGUCACACUGUGCUCACUUUGUCAAAUAAAAGAUGGAGUCGUUUGACGUCUUUUAACACCC
UUUGAGCACAUGAGCAGACGGAGUGAUCAUGA
>hsa-mir-8056 MI0025892
CUUCUCUGAGCAUCCAGCCUCUAGGAGCUGUGCCUCCUGGCCUAUUCCUGUUCGUGGAUUGUCUG
GAUGCAUCCUUGAGACU
>hsa-mir-8057 MI0025893
AAUUUGGGAUGUGGCUCUGUAGUAAGAUGGACUUGCGGUUCAAUCCCAGCUCAGGGCCUUUUUAA
AUUA
>hsa-mir-8058 MI0025894
UGGUCACUGCCAGUGCUGAGACAUUAAAGAUCCAGGCCCCACGGAGAACCAGGGCACCUGGACUU
UGAUCUUGCCAUAAUGGUAUGCCU
>hsa-mir-8059 MI0025895
UACAGGUGCAGGGGAACUGUAGAUGAAAAGGCUUGGCACUUGAGGGAAAGCCUCAGUUCAUUCUC
AUUUUGCUCACCUGUU
>hsa-mir-8060 MI0025896
UUUAAAUCCUGUUUUUCCCACUUACUAUUCUGGUCAGAUAUCCCAUGAAGCAGUGGGUAGGAGGA
CAGGAAAAAGC

>hsa-mir-8061 MI0025897
GAGAGGAUGCCUUAGAUUAGAGGAUAUUGUUCAGUGGGAUUUGGAGAGGACGCCUUAGAUUAGAG
GUAUUGUUCA
>hsa-mir-8062 MI0025898
CUUAUGCAUGCAGUGAUUUGAGGAUUAUUGCUCACGGUAAGAAAAACAUGAGUAAUAUAUUGUCU
UCAAAGAACCUAUGCUCUAA
>hsa-mir-8063 MI0025899
UAGAGGCAGUUUCAACAGAUGUGUAGACUUUUGAUAUGAGAAAUUGGUUUCAAAAUCAGGAGUCG
GGGCUUUACUGCUUUU
>hsa-mir-8064 MI0025900
AGAUGGAGUGAGCACACUGAGCGAGCGGACAUCCUUCCAUAGUAAAUAAGCGGUGCCGCGCGCUC
CGCUGGCUCCCAAACACUCCAGUCC
>hsa-mir-8065 MI0025901
CAGUACAUGAGAGCCAUCAUUUUUGUUGUUGUUGUAUUUUUCAGCAAGGCAAGGGCUUUGAUGUC
UUGUAGGAACAGUUGAAUUUUGGCUUCAUGUAAUU
>hsa-mir-8066 MI0025902
CAUCUUGCUUACAUUCCUGUGGAACAUAUUGUUGCCUCUGCAUGUUACAAUGUGAUCUUUUGGAU
GUAGGGCAAAUCU
>hsa-mir-8067 MI0025903
UUCUGUGUUUCCUAGAAACUGUAAACUUAGUCAUAAAGAUGAUCCUGAGUGCACAGUCUUUCUUG
AGACUUUCAGCC
>hsa-mir-8068 MI0025904
GGCUAACUCAUGUUUGUUGUAAGGAUCGUUGUGGCAAUACAGUUAUCCUUACAACAAAUACUUUG
CCA
>hsa-mir-8069 MI0025905
CGCCUGAGCGUGCAGCAGGACAUCUUCCUGACCUGGUAAUAAUUAGGUGAGAAGGAUGGUUGGGG
GCGGUCGGCGUAACUCAGGGA
>hsa-mir-8070 MI0025906
ACUUGAUCCCAUGUGAUUGACGGCUGACUCCAUGUGAGUGAAGCCUGAUCAUCAUGUGAGUGAAG
CCUGAUCAUCAUGUGAGUGAAGC
>hsa-mir-8071-1 MI0025907
CGGCCACAUGGCCCAGGCUCUUCUCCGAGUGAUCUCGGUGGACUGGAGUGGGUGGUAGGUGGCAG
>hsa-mir-8072 MI0025908
GCGUCAAGAUGGCGGCGGGGAGGUAGGCAGAGCAGGACGCCGCUGCUGCCGCCGCCACCGCCGCC
UCCGCUCCAGUCGCC
>hsa-mir-8073 MI0025909
GAUUUCAGUGACCUGGCAGCAGGGAGCGUCGUCAGUGUUUGACUGUUUAUGGUAUGUCAGGGAGC
UGGUUCC
>hsa-mir-8074 MI0025910
CCAGUUCCUGAGUUUAUGCAAGAUGCCCAUGGGAGCCCAGAGACGUCCUAUGGCGAGACUGGCAU
GUACUCACACAACUGA
>hsa-mir-8075 MI0025911
CCUUGCUGAUGGCAGAUGUCGGAUCUGCCUCGCUUAUACGUGCCCUUGCUGAUGGCAGAUGUCGG
GUCUGCCUCGCUUAU

>hsa-mir-8076 MI0025912
CCUAUCCACACGUCUGGUCUAGAGUUCUCCUGUGAGAGCUACUAAGUCUUAUAUGGACUUUUCUG
AUACAAUGUAGUGGAAAG
>hsa-mir-8077 MI0025913
GUGGGGCAGGGGGUGGGAUGGGCACUGGGAGUCCAAAGGAAAGGGCUGAGUGGGGUUCUGACUCC
UCUGCCCAAC
>hsa-mir-8078 MI0025914
UCCGCCCCGCCCCCUCCACCGGGCUGACCGGCCUGGGAUUCCUGCCUUCUAGGUCUAGGCCCGGU
GAGAGACUCCACACAGCGG
>hsa-mir-8079 MI0025915
UGCCUGGGUUCAGUGAUCGUCUCUGCUGGCUGUAGGGCAGCAGUCAGAGGGGACAUCACAGGCCU
CCAGAGG
>hsa-mir-8080 MI0025916
AUGGCUCUAAGAAGGACACUGGUGUCAACGGCUGAGCCCUUUGAUAAGGGGGAGGUUUGGAAGAC
ACUUAUGUCCUGGAUUGGGAAAUG
>hsa-mir-8081 MI0025917
UUAUUGUCUUUAUUUUCUGAGUCUUGCCUCUAUGGCCCCCCUCCUCUCUGAAAGCAAGGCCACUU
GAGUCGUGCCUUUCUGAAUGAAGUCAAUGU
>hsa-mir-8082 MI0025918
GCCUGUGUGAUGAUGGAGCUGGGAAUACUCUGGGGAGAGAGUCCUCUUUUCAGCUGUAUUUUGCU
UCCUUCCCACACAGAC
>hsa-mir-8083 MI0025919
CGAGGGGGUGCAGGACUUGACGGCUGCAACUGUGCGGCCGGCCACACCGUCACACCGGGUGCAGC
GUCUGUCAUUCUGUACCCUCAUCU
>hsa-mir-8084 MI0025920
CAGAUAAAUAGAAUACUAAGUAAAAAAUCAGUAUGGAUUAAAUUUAUAUUUUCCUCGAUACUGAG
UAUUUUUUCUUAGGUUUAGUUUGG
>hsa-mir-8085 MI0025921
CUAGGAGGGAUGGGAGAGAGGACUGUGAGGCAUGGGUGGCUCUAUGGUCACGCCCAUCUUCCUAC
>hsa-mir-8086 MI0025922
UCCACUAGUCUGCUAGUCUGGACUGAUAUGGUUAGCUUUUUUUUUUUUUUGAGAUGGAGUCUGGC
UCUGUUGCCCAGGCUGGAGUACAGUGGC
>hsa-mir-8087 MI0025923
UCUAAGAAGUGAAGACUUCUUGGAUUACAGGGGCCCUACUUUAAGGGCCCUUUCAGUUGGAAGUU
UUCCUUUCUGCCU
>hsa-mir-8088 MI0025924
GGGAGGAGGGCCUCGGUACUGGAAAGGGGUAUGGAUUCUGUUGUUUUGGCAACAUUCUUCUUAUC
GGCACUGGGUCACUCAGCUCA
>hsa-mir-8089 MI0025925
AAGGAGCACUCACUCCAAUUUCCCUGGACUGGGGGCAGGCUGCCACCUCCUGGGGACAGGGGAUU
GGGGCAGGAUGUUCCAG

(2). 1612 Xue pseudo miRNAs

>random_seq_from_cds__NO_6 RANDOM_LENGTH 97 SEQ_LENGTH 84 GC_CONTENT 0.702380952380952 BASEPAIR 31 FREE_ENERGY

-44.90 LEN_BP_RATIO 2.70967741935484
GAGGGCAGGGGGCACAGUCCAACUCCAGGCUUGUAGCUGUCCAGGGGCUGGGUGCCCGCCCGGCA
GCGGCAGACUGUGUCCUGUGUGGCCGUGCACA
>random_seq_from_cds__NO_16 RANDOM_LENGTH 81 SEQ_LENGTH 69
GC_CONTENT 0.565217391304348 BASEPAIR 23 FREE_ENERGY
-23.00 LEN_BP_RATIO 3
AAGCACCAGAGACGAACAGUCUGGUGUCAGGAGCAAGAGAAAGGCUCAUGACAUCUCCAGUGUGU
CCGGUAAACGUGGUCG
>random_seq_from_cds__NO_21 RANDOM_LENGTH 110 SEQ_LENGTH 99
GC_CONTENT 0.565656565656566 BASEPAIR 29 FREE_ENERGY
-28.75 LEN_BP_RATIO 3.41379310344828
AUGGCGUAGAUCUUGGCCAGGUGCCCCCGCAGUGUCCUCCUCGUGCGCAUUUGGAUUCUUCCCAC
UGGGUCGAUGUUGUUUGUGAUCCUGAGAGAGAGUUGCAUCUGCAC
>random_seq_from_cds__NO_41 RANDOM_LENGTH 96 SEQ_LENGTH 83
GC_CONTENT 0.746987951807229 BASEPAIR 30 FREE_ENERGY
-39.40 LEN_BP_RATIO 2.76666666666667
CCACCAGAUCCGCGCCACGCUCCCGCACCAGUGCGGCCAGCCCCUUAUCCAGGCGGCCUGAGGUC
GAGGCACGGGGAGCUGGAGCCCGUCUGCACC
>random_seq_from_cds__NO_44 RANDOM_LENGTH 86 SEQ_LENGTH 75
GC_CONTENT 0.453333333333333 BASEPAIR 26 FREE_ENERGY
-24.44 LEN_BP_RATIO 2.88461538461538
CAAAGGGGAAGACUCCCAAAAUGAUGUCUAUUCCACUGUUAUCUGCGAAAAUUAAGGCACAUUUA
UGAGGGGGCCCCCUUUAAUCU
>random_seq_from_cds__NO_49 RANDOM_LENGTH 110 SEQ_LENGTH 104
GC_CONTENT 0.625 BASEPAIR 34 FREE_ENERGY -40.10
LEN_BP_RATIO 3.05882352941176
GGGUCCCAUAGGCGCAAGGGCUGCUGCCUCAGGGUCUGAAGCUUGUUCCAGUACUUCUGCCGGAA
CUUCUCCGCCCUCUCGGCUGCAUCCACAGAGUCUGGCUGGCUCGC
>random_seq_from_cds__NO_65 RANDOM_LENGTH 75 SEQ_LENGTH 58
GC_CONTENT 0.568965517241379 BASEPAIR 21 FREE_ENERGY
-21.60 LEN_BP_RATIO 2.76190476190476
UUGGCGACUUUGUGCUGUACCGUUGAAUAGUAGGCCAGCUUGGUUAACGACCCGCCCUAUGUCGA
UGGCGAAGCG
>random_seq_from_cds__NO_71 RANDOM_LENGTH 89 SEQ_LENGTH 73
GC_CONTENT 0.616438356164384 BASEPAIR 29 FREE_ENERGY
-35.10 LEN_BP_RATIO 2.51724137931034
CACCUUGGUCUGGUGCUGACAUUCCUCCAGGGUCCCAUUGGGAGAGAAGGUCCCCGGGGGGCAGU
UCUGACACAGGGUGUCCUGACUCU
>random_seq_from_cds__NO_76 RANDOM_LENGTH 78 SEQ_LENGTH 64
GC_CONTENT 0.671875 BASEPAIR 22 FREE_ENERGY -26.30
LEN_BP_RATIO 2.90909090909091
AGGACGGCAGAGCUGGGGCGUAGCAGGGGGCUCCCAGGAAGGUGAGAUACAGCACCCAGCCUCAA
GACGUCGGUUUUG
>random_seq_from_cds__NO_80 RANDOM_LENGTH 70 SEQ_LENGTH 61

GC_CONTENT 0.573770491803279 BASEPAIR 23 FREE_ENERGY
-19.90 LEN_BP_RATIO 2.65217391304348
GCAUAGUUGAUGAAGAAGAGCUGCUCAUGGGUGAGAUCCAGGCCGGGCAGCUGCUGGUCCUUGCC
ACCCU
>random_seq_from_cds__NO_86 RANDOM_LENGTH 84 SEQ_LENGTH 75
GC_CONTENT 0.546666666666667 BASEPAIR 25 FREE_ENERGY
-23.10 LEN_BP_RATIO 3
GCGCCCACCUUGAGGUUCUGCAGACUGUUCUCAAAGUACAGGUCCUCUGAGAAGUUCAGCAUUGG
AGUACUCCUCGUCCAGGCG
>random_seq_from_cds__NO_93 RANDOM_LENGTH 77 SEQ_LENGTH 57
GC_CONTENT 0.56140350877193 BASEPAIR 20 FREE_ENERGY
-15.80 LEN_BP_RATIO 2.85
AGAGUCCAGUUAAAUCCCCUUCAGGCCAAACUGGCUUUGCAGCUCCUCCAGUCCCAUCCGGUGGU
ACAAGGCGAUGA
>random_seq_from_cds__NO_112 RANDOM_LENGTH 110 SEQ_LENGTH 94
GC_CONTENT 0.542553191489362 BASEPAIR 34 FREE_ENERGY
-32.91 LEN_BP_RATIO 2.76470588235294
UUCAGCAAGGUCAAGGUCCCUCCCACUGAUCUCUGCCUCCCGGUGGCUGCUGAAGCGCGGAGAGC
UGUUCUUAGUGGAAGAAACCGGACUUUUUCGAAAAAUUGCCAGCC
>random_seq_from_cds__NO_117 RANDOM_LENGTH 88 SEQ_LENGTH 79
GC_CONTENT 0.683544303797468 BASEPAIR 27 FREE_ENERGY
-27.20 LEN_BP_RATIO 2.92592592592593
GCAGGCGGACGUGGUCCUGGUUCUGCAGCAGGAGGAUGGGGUGGCUCUAUGGCGAGAGGCUCCGG
GACGGAGAGACGGGAUGGUUCCC
>random_seq_from_cds__NO_129 RANDOM_LENGTH 94 SEQ_LENGTH 61
GC_CONTENT 0.655737704918033 BASEPAIR 22 FREE_ENERGY
-23.60 LEN_BP_RATIO 2.77272727272727
AUGCUCACGUAAUCUUUGCAGUCGCGCCGUUCUGCCAGCGCCAUGUAGCGGCCGUCCCUGGUGAA
GGUGAUUCCCCUGCAGACAAGCUUUCGGG
>random_seq_from_cds__NO_130 RANDOM_LENGTH 85 SEQ_LENGTH 75
GC_CONTENT 0.453333333333333 BASEPAIR 26 FREE_ENERGY
-22.10 LEN_BP_RATIO 2.88461538461538
UAUUUGAUGUAAGACACGGAUUUUGUGCACAAGGACCAGACGGUUAUCCGCAGCAUGGAAUUCCG
UGGUGUUGAGAAUGUGGCGC
>random_seq_from_cds__NO_132 RANDOM_LENGTH 110 SEQ_LENGTH 106
GC_CONTENT 0.575471698113208 BASEPAIR 37 FREE_ENERGY
-32.90 LEN_BP_RATIO 2.86486486486486
CCUGCACCAGCCCUCGCUUGUACAUGGCGCACAGGAUGAAGAGCGAGUCUGCCGACCACUCGAUG
UGCUGGAUCUGGUCUAGGCACGUGUACAGCUGAAGGAUCUGAAGG
>random_seq_from_cds__NO_135 RANDOM_LENGTH 72 SEQ_LENGTH 67
GC_CONTENT 0.701492537313433 BASEPAIR 23 FREE_ENERGY
-26.40 LEN_BP_RATIO 2.91304347826087
UUCGGCGUGGCUGGCCGGAGCUGCCAGGAGGUGCUGCGCAAGGGCUGUCUCCGCUUCCAGGCUCC
CUGAGCG

>random_seq_from_cds__NO_140 RANDOM_LENGTH 89 SEQ_LENGTH 80 GC_CONTENT 0.5875 BASEPAIR 29 FREE_ENERGY -31.00 LEN_BP_RATIO 2.75862068965517
UGGAGUCCCGAUUUCAGAGCAAGUCUGGCUAUCUGAGAUACAGCUGUGAGAGCCGGAUCCGGAGUUACCUGAGGGAGGGUGAGCUCCUA
>random_seq_from_cds__NO_149 RANDOM_LENGTH 71 SEQ_LENGTH 49 GC_CONTENT 0.693877551020408 BASEPAIR 20 FREE_ENERGY -28.50 LEN_BP_RATIO 2.45
CGGAGAGUGUCCCUGGCUCUCUCGCAGGGGCAGGCGGGGUCACAGAGGAUGCUCCCGGAGAGAGGUCGGCU
>random_seq_from_cds__NO_153 RANDOM_LENGTH 70 SEQ_LENGTH 53 GC_CONTENT 0.566037735849057 BASEPAIR 19 FREE_ENERGY -17.70 LEN_BP_RATIO 2.78947368421053
UGCAUAUGUCACAGGUCCUUUGUCCACCUGCGCUGGAGAAACUAUUUGGAGGACAGGGACUGCAAAUCUG
>random_seq_from_cds__NO_159 RANDOM_LENGTH 90 SEQ_LENGTH 71 GC_CONTENT 0.577464788732394 BASEPAIR 24 FREE_ENERGY -30.30 LEN_BP_RATIO 2.95833333333333
UCACGGCCUUGGCGAUCCUCUUUGGGUUGGUCACUGUGAGAUCAUCCCCCACUACCUGGAUUCCUGCACUGGCUGUGAACUUCUGCCAAG
>random_seq_from_cds__NO_187 RANDOM_LENGTH 77 SEQ_LENGTH 58 GC_CONTENT 0.551724137931034 BASEPAIR 22 FREE_ENERGY -20.90 LEN_BP_RATIO 2.63636363636364
ACGGCCAUGACACUGUCCUUGUGCUCUCGCAGCACCUCCAUCACUGUGUGGCAUGUGAUUCUGUAGUUGCCAUCCAG
>random_seq_from_cds__NO_196 RANDOM_LENGTH 97 SEQ_LENGTH 76 GC_CONTENT 0.565789473684211 BASEPAIR 28 FREE_ENERGY -35.40 LEN_BP_RATIO 2.71428571428571
UGCAAAGACGGUGCUAUGGACUGAAUGCGAAUGAUUGGCUGGUUGGGGUCAUAUGUUCCUGGCACAGCCAAUUCAAGGUCCCGGCACAUCAGAAGUU
>random_seq_from_cds__NO_197 RANDOM_LENGTH 70 SEQ_LENGTH 56 GC_CONTENT 0.517857142857143 BASEPAIR 21 FREE_ENERGY -18.80 LEN_BP_RATIO 2.66666666666667
UUGGGGAAACAUAUUGCAGCUCUAAGGAUGUGAGCCUGAGGCAGCUGCUUUGAGAUUCGUCGGAACACAU
>random_seq_from_cds__NO_198 RANDOM_LENGTH 84 SEQ_LENGTH 78 GC_CONTENT 0.487179487179487 BASEPAIR 31 FREE_ENERGY -28.00 LEN_BP_RATIO 2.51612903225806
GAUAAUAGAGGUCCCAGGCUUGGGUGAGGUCCUUGACAUUCCCUGAUUUCAUGUACUUCCUGCACCACUCUUGGGCCUCCAUUA
>random_seq_from_cds__NO_204 RANDOM_LENGTH 68 SEQ_LENGTH 46 GC_CONTENT 0.5 BASEPAIR 20 FREE_ENERGY -20.12 LEN_BP_RATIO 2.3
UAGCCAGGUAUCAAUCUGGAUGGCUUUCACCCCCUCCACUAAGGCCUCAUUGACAUCUGGCCAGU

GAC
>random_seq_from_cds__NO_207 RANDOM_LENGTH 80 SEQ_LENGTH 74 GC_CONTENT 0.716216216216216 BASEPAIR 24 FREE_ENERGY -31.00 LEN_BP_RATIO 3.08333333333333
CCCUCGGUGCUGGCAGUGGUGGUGGCAGUGGCGGCCGUGGUGGCGGCAGUGGUGGCGUUGGUGAUGUUGGCCCCGCUGGC
>random_seq_from_cds__NO_219 RANDOM_LENGTH 82 SEQ_LENGTH 74 GC_CONTENT 0.581081081081081 BASEPAIR 22 FREE_ENERGY -26.45 LEN_BP_RATIO 3.36363636363636
UUGGCUUGGGUCUCAUCAUUAACCAGGGUCCACUUUUCACAGCACUGCUGGUGGAGUUGACCCCCAUUCCCCCAAGGCCUCG
>random_seq_from_cds__NO_222 RANDOM_LENGTH 78 SEQ_LENGTH 64 GC_CONTENT 0.578125 BASEPAIR 22 FREE_ENERGY -28.20 LEN_BP_RATIO 2.90909090909091
CCGGCCGCUGCCUCCGGCUGCUGUAGCUUAUUAUUAAUGCCUGAUGAGAGAUUCUAGAAUGGCAGGGGUGGGGCCUUU
>random_seq_from_cds__NO_239 RANDOM_LENGTH 90 SEQ_LENGTH 72 GC_CONTENT 0.458333333333333 BASEPAIR 29 FREE_ENERGY -26.90 LEN_BP_RATIO 2.48275862068966
GGUAGAACUCAUCCAGAGGCAAGUUUCCCAUGUUGACCAGCAUUUCACUAGUGCUAUAGUCAGCAGGAAUCCUGACUUGACUUGGAUUCU
>random_seq_from_cds__NO_242 RANDOM_LENGTH 87 SEQ_LENGTH 75 GC_CONTENT 0.586666666666667 BASEPAIR 27 FREE_ENERGY -29.50 LEN_BP_RATIO 2.77777777777778
AAGUAGGGUACUUCCUGUAGGGCUCUACUACAUAGCCAGUGCUGGCCACCAACUGUCCCAGGGUCCACAGAGCCACCCUGCCUUUUG
>random_seq_from_cds__NO_256 RANDOM_LENGTH 82 SEQ_LENGTH 62 GC_CONTENT 0.564516129032258 BASEPAIR 23 FREE_ENERGY -21.14 LEN_BP_RATIO 2.69565217391304
UUGCUCGAGCCAGCAUGCUGAUGCAAGUGAAGACUGUGGCAUCCACCUGCAUUGCCUUCUGCCUCCUUAUGGGCGAAGUCCU
>random_seq_from_cds__NO_261 RANDOM_LENGTH 84 SEQ_LENGTH 68 GC_CONTENT 0.617647058823529 BASEPAIR 28 FREE_ENERGY -36.80 LEN_BP_RATIO 2.42857142857143
CCGGCUCUCCACCAGGGUGGACUUAGCUGGACUGGGGGAGGUCCCAAAUCCCAUGAGGCCUUGGUGAGAGCUGUACCCCAGCAG
>random_seq_from_cds__NO_275 RANDOM_LENGTH 110 SEQ_LENGTH 99 GC_CONTENT 0.686868686868687 BASEPAIR 35 FREE_ENERGY -45.30 LEN_BP_RATIO 2.82857142857143
GCUCGAAGCGGACGAAGCGGACGCCCGGCCCGUAGUCGGUGAAGGUGUGGGAGAUCCUCCAUCCAGCCCCCGCCGUCACUGUCUUGGGGCACUGCCACCUGCCCGCUGCU
>random_seq_from_cds__NO_280 RANDOM_LENGTH 73 SEQ_LENGTH 60 GC_CONTENT 0.6 BASEPAIR 21 FREE_ENERGY -19.10 LEN_BP_RATIO 2.85714285714286

GCCUUCCAAGUCCUCCUUCCCCACACGGGUUACGCAGAAGGUUGCGGCGCCGCUUGCUCAGGAAG
UAGAACUG
>random_seq_from_cds__NO_289 RANDOM_LENGTH 97 SEQ_LENGTH 83
GC_CONTENT 0.590361445783133 BASEPAIR 28 FREE_ENERGY
-34.00 LEN_BP_RATIO 2.96428571428571
UUUUGUCACAUCCUACGAGAAUGUGCCUCAAGUCCCAGCUGGUGGACCUUGUAGCCGAGGGCUAC
UGGGAGGAGCUACUAGACACAUUCCGGCCGGA
>random_seq_from_cds__NO_292 RANDOM_LENGTH 78 SEQ_LENGTH 56
GC_CONTENT 0.660714285714286 BASEPAIR 19 FREE_ENERGY
-23.30 LEN_BP_RATIO 2.94736842105263
CCUCUUCCAGCAUGGGGGCAGGGACACCCAGUACUGGGCAGGCUGGUAUGGGCCCCGAGUCACCA
ACAGCAGCAUUGU
>random_seq_from_cds__NO_310 RANDOM_LENGTH 89 SEQ_LENGTH 84
GC_CONTENT 0.630952380952381 BASEPAIR 27 FREE_ENERGY
-35.40 LEN_BP_RATIO 3.11111111111111
UCCCAAUGGGCCCCAGCCCCCCAGCUGAAGGGAGCACUGGCGACUUCGCUCUUCCAGUUGGGACU
GAUUGUGGGUGUGACAGCCUUGGG
>random_seq_from_cds__NO_318 RANDOM_LENGTH 77 SEQ_LENGTH 60
GC_CONTENT 0.516666666666667 BASEPAIR 21 FREE_ENERGY
-20.70 LEN_BP_RATIO 2.85714285714286
UGGCCGCGUGGAAAGUGUCAAAAUUCUUCCCAAGAGGGGAUCUGAAGGAGGAGUGGCUGCCUUUG
UGGAUUUUGUGG
>random_seq_from_cds__NO_336 RANDOM_LENGTH 97 SEQ_LENGTH 82
GC_CONTENT 0.475609756097561 BASEPAIR 25 FREE_ENERGY
-20.92 LEN_BP_RATIO 3.28
UUUGACCGCUUAAAAGGCAUGGCCCUGGUUCUCUACAAUGAAAUUGAAUAUGCACAAGCAGCUGU
AAAAGAGACCAAAGGGAGGAAAAUCGGUGGGA
>random_seq_from_cds__NO_349 RANDOM_LENGTH 71 SEQ_LENGTH 57
GC_CONTENT 0.491228070175439 BASEPAIR 19 FREE_ENERGY
-16.30 LEN_BP_RATIO 3
CUGCCCUGGACCAGAAACUUCAGGUCUCUCAGACGGAGCCUGCAAAAUCUGACUUGUCUAAACUG
GAAUCA
>random_seq_from_cds__NO_351 RANDOM_LENGTH 99 SEQ_LENGTH 78
GC_CONTENT 0.576923076923077 BASEPAIR 28 FREE_ENERGY
-27.70 LEN_BP_RATIO 2.78571428571429
AGGAAGCACCUCAAGCCUGAGCAGCCUGCAGAUGGGGUAAGUGCUGUGGAUCUGGAGAAGCUGGA
AGCCAGGAAAAGGCGCUUUGCAGAUUCCAAUUUA
>random_seq_from_cds__NO_398 RANDOM_LENGTH 110 SEQ_LENGTH 79
GC_CONTENT 0.443037974683544 BASEPAIR 26 FREE_ENERGY
-17.40 LEN_BP_RATIO 3.03846153846154
CCAUUCCACUCCUCCUCAGUCAUGUACUUCUGACCUAAGCAAGAUUCCCUCCACAGAGAAUUCGU
CCCAAGAAAUCAGUGUUGAGGAAAGGACUCCAACCAAAGCAUCUG
>random_seq_from_cds__NO_410 RANDOM_LENGTH 82 SEQ_LENGTH 71
GC_CONTENT 0.633802816901408 BASEPAIR 28 FREE_ENERGY

-38.70 LEN_BP_RATIO 2.53571428571429
CGGGUGCGGUUACUGCUGCAUCUGGUGGUGUAACGGCCACAACAGGCACGGUGACAAUGGCAGGG GCAGUGAUUGCGCCGUC
>random_seq_from_cds__NO_424 RANDOM_LENGTH 80 SEQ_LENGTH 66 GC_CONTENT 0.590909090909091 BASEPAIR 23 FREE_ENERGY -26.90 LEN_BP_RATIO 2.8695652173913
UCGCUCGAGCCACAGCCCCUGUGCAGUCAGAGGUACUAGUCAUGCAGUCUGAGUACCGACUGCAC CCCUAUACUGUGCCA
>random_seq_from_cds__NO_440 RANDOM_LENGTH 110 SEQ_LENGTH 87 GC_CONTENT 0.586206896551724 BASEPAIR 21 FREE_ENERGY -17.38 LEN_BP_RATIO 4.14285714285714
CAGAUCUUCCCGCCCUGUGAGUUCUCUGAGAGUCACCUGUCCCGCCUGGCCCCUGACCUCCUUGC CAGCAUCUCCAACAUCUCUCCCCACCUCAUGAUUGUCAUUGCCUC
>random_seq_from_cds__NO_441 RANDOM_LENGTH 87 SEQ_LENGTH 74 GC_CONTENT 0.662162162162162 BASEPAIR 27 FREE_ENERGY -34.90 LEN_BP_RATIO 2.74074074074074
CGUGUGAGAUGGAGGAGUUUGUGGGGCUGCGUGAAGGCUCCUCAGGGAACCCUGUGACUCUGCAG GAGCUGUGGGGCCCCUGUCCCC
>random_seq_from_cds__NO_450 RANDOM_LENGTH 119 SEQ_LENGTH 93 GC_CONTENT 0.602150537634409 BASEPAIR 34 FREE_ENERGY -37.60 LEN_BP_RATIO 2.73529411764706
AGACCAGUUUCCGGGUGGACGUUCCCUUCGACCUGCCUGAGAUCUUCUUUUUGUGGCGCUGGGG GGGUCUCUGUGGCAUCCUGGGCAGCGCUUACCUCUUCUGUCAGCGAAUCUUCUU
>random_seq_from_cds__NO_468 RANDOM_LENGTH 110 SEQ_LENGTH 88 GC_CONTENT 0.443181818181818 BASEPAIR 33 FREE_ENERGY -29.60 LEN_BP_RATIO 2.66666666666667
AGUUUCUCACGCUCUUCUAUGGACUGCAGAUACUGCUGCUUGCCUUCCUGAGAUUCAUCCUUCUU CUUCAAAUAAGGCUCAAUGGAUUUGUACUGUGCAUAGAAGUUGCU
>random_seq_from_cds__NO_481 RANDOM_LENGTH 75 SEQ_LENGTH 62 GC_CONTENT 0.596774193548387 BASEPAIR 21 FREE_ENERGY -20.50 LEN_BP_RATIO 2.95238095238095
GGUCCCAAGUGGCCCUCUCACUACCUGAUGGUCCCCGGUGGAAAGCACAACAUGGACUUCUACGU GGAGGCCCUC
>random_seq_from_cds__NO_487 RANDOM_LENGTH 86 SEQ_LENGTH 58 GC_CONTENT 0.672413793103448 BASEPAIR 21 FREE_ENERGY -27.10 LEN_BP_RATIO 2.76190476190476
UGGGAACCUGGAAGUGAGCCCCCCAGUCACAGUCAGGGGCAAGGAAUACCCGCUGGGCAGGAUUC UCUUCGGGGACAGCUGUUAUC
>random_seq_from_cds__NO_489 RANDOM_LENGTH 77 SEQ_LENGTH 54 GC_CONTENT 0.537037037037037 BASEPAIR 19 FREE_ENERGY -18.90 LEN_BP_RATIO 2.84210526315789
CCCUGUGAAGCUCUAUUCUGACUGGCUGUCCGUGGGCCACGUGGACGAGUUCCUGAGCUUUGUGC CAGCACCCGACA
>random_seq_from_cds__NO_501 RANDOM_LENGTH 81 SEQ_LENGTH 55

GC_CONTENT 0.672727272727273 BASEPAIR 23 FREE_ENERGY -33.70 LEN_BP_RATIO 2.39130434782609
UCAGGCAGCUGGGCCUGCCCACCGCCCUCAAGAGAGGGUGUGGUGACUCUGCUGUCUGACUACGA
GGUGUGCAAGGAGGGC
>random_seq_from_cds__NO_502 RANDOM_LENGTH 87 SEQ_LENGTH 71 GC_CONTENT 0.549295774647887 BASEPAIR 22 FREE_ENERGY -20.22 LEN_BP_RATIO 3.22727272727273
GAUGUGCUGACCCCAGAGCAGGCUCGCGUCCUGGAAGCUUUUUGGGUAUGAGAUGGCUGAAUUCA
AGGUGACCAUCAAAUACAUGUG
>random_seq_from_cds__NO_509 RANDOM_LENGTH 89 SEQ_LENGTH 76 GC_CONTENT 0.460526315789474 BASEPAIR 27 FREE_ENERGY -18.20 LEN_BP_RATIO 2.81481481481481
UCCAGCAAAGCCAUGAUCCAGAUCCCAGAGGUAGACAGAUGAGACGCCACCUUCAAAAUACCAGG
UCUCGAUACUGGUCAAAGGCAUUG
>random_seq_from_cds__NO_512 RANDOM_LENGTH 84 SEQ_LENGTH 70 GC_CONTENT 0.514285714285714 BASEPAIR 27 FREE_ENERGY -23.40 LEN_BP_RATIO 2.59259259259259
CUGGUCAACAGAAGACAGGAGAUCCUCACAUAGACUGGGGACCCAGGUCGAUCAGGUCGCUGAGG
UUUUUCUCGAUUUGCUGGG
>random_seq_from_cds__NO_516 RANDOM_LENGTH 75 SEQ_LENGTH 49 GC_CONTENT 0.673469387755102 BASEPAIR 19 FREE_ENERGY -24.80 LEN_BP_RATIO 2.57894736842105
CCUGGGGAGGAGACCAGGCUGGAGAUGGACAAGUUCCCCUUCGUGGCCCUCUCCAAGGACGUACA
ACACCAAUGC
>random_seq_from_cds__NO_521 RANDOM_LENGTH 110 SEQ_LENGTH 80 GC_CONTENT 0.5625 BASEPAIR 24 FREE_ENERGY -27.80 LEN_BP_RATIO 3.33333333333333
AAGAAUAAAACUGAUGUGGAGUAUGAGAGUGACGAGAAAGCCAGGGGCACGAGGCUGGACGGCCU
GGACCUCGUUGACACCUGGAAGAGCUUCAAACCGAGAUACAAGGC
>random_seq_from_cds__NO_522 RANDOM_LENGTH 110 SEQ_LENGTH 95 GC_CONTENT 0.568421052631579 BASEPAIR 34 FREE_ENERGY -30.50 LEN_BP_RATIO 2.79411764705882
ACUCCCACUUCAUCUGGAACCGCACGGAACUCCUGACCCUUGACCCCCACAAUGUGGACUACCUA
UUGGGGUCUCUUCGAGCCAGGGGACAUGCAGUACGAGCUGAACAG
>random_seq_from_cds__NO_529 RANDOM_LENGTH 84 SEQ_LENGTH 78 GC_CONTENT 0.705128205128205 BASEPAIR 28 FREE_ENERGY -33.90 LEN_BP_RATIO 2.78571428571429
GGCCCCAUGGCGCACCUGCUGCACGGCGUCCACGAGCAGAACUACGUCCCCCACGUGAUGGCGUA
UGCAGCCUGCAUCGGGGCC
>random_seq_from_cds__NO_551 RANDOM_LENGTH 86 SEQ_LENGTH 47 GC_CONTENT 0.74468085106383 BASEPAIR 19 FREE_ENERGY -31.60 LEN_BP_RATIO 2.47368421052632
UGGAGGUGGUGCUCCCCUCCCCCUUGGUGGUGGUGGAGCACCUCGCCCUCCCCUUCCUCCUCCUC
UUCCUCUUACUGCAUAGCCAU

>random_seq_from_cds__NO_553 RANDOM_LENGTH 86 SEQ_LENGTH 59 GC_CONTENT 0.711864406779661 BASEPAIR 21 FREE_ENERGY -27.30 LEN_BP_RATIO 2.80952380952381
UAUCCACCUCUCCCCCCACCACGACCCCGACCUCUAAUUGGAGGUGGCAUGCGAGGAGGAGGGUG
GUAGUAAUAAUCUUCAUACCG
>random_seq_from_cds__NO_567 RANDOM_LENGTH 84 SEQ_LENGTH 67 GC_CONTENT 0.477611940298507 BASEPAIR 19 FREE_ENERGY -17.60 LEN_BP_RATIO 3.52631578947368
GUCUGAAAUAUUUCAUCAAGUCUUUCUGCCACCUUCUGUGGGAGGCCUGCCUCUAUCAGUGUCUU
GUAGUGUUCUGUGUGAGUU
>random_seq_from_cds__NO_578 RANDOM_LENGTH 71 SEQ_LENGTH 65 GC_CONTENT 0.523076923076923 BASEPAIR 23 FREE_ENERGY -18.30 LEN_BP_RATIO 2.82608695652174
CUGGGGUUGGGAAAACAGUAAAUAGCUUGCGAAAACACGAGCAUGUUGGAAGCUUUGCCAGGGAC
CUAGUG
>random_seq_from_cds__NO_595 RANDOM_LENGTH 90 SEQ_LENGTH 63 GC_CONTENT 0.492063492063492 BASEPAIR 22 FREE_ENERGY -18.10 LEN_BP_RATIO 2.86363636363636
CCCUAAAAUGAUGACCUUGCACCAGCAAUGCAUCCGAGUACUUAAAAACAACAUCGAUUGCAAUC
UUUGAAGUGGGAGGAGUCCCAUACU
>random_seq_from_cds__NO_605 RANDOM_LENGTH 88 SEQ_LENGTH 75 GC_CONTENT 0.613333333333333 BASEPAIR 26 FREE_ENERGY -27.10 LEN_BP_RATIO 2.88461538461538
UCUGUCCCCAUGCGGCCUAGGAUCCCUGUGACCCUCAACAUGAAGAUGGUGAUGCCCUCCUGGGU
UUGACCUGAUGGGGCUGAGUCCA
>random_seq_from_cds__NO_611 RANDOM_LENGTH 73 SEQ_LENGTH 64 GC_CONTENT 0.578125 BASEPAIR 19 FREE_ENERGY -27.74 LEN_BP_RATIO 3.36842105263158
GCUCCUGUCCUCAGGGAGAUGGCAGCUGUGAAGGAAUUUCUUGAGAAGCUGCUGCCUCCUGUCUA
ACUCAGGC
>random_seq_from_cds__NO_620 RANDOM_LENGTH 78 SEQ_LENGTH 65 GC_CONTENT 0.584615384615385 BASEPAIR 21 FREE_ENERGY -20.20 LEN_BP_RATIO 3.0952380952381
ACACCAGGUUCUUCACCCCGUGGGCCUUCAUGAUCCUCCAGAAGCUGGAUGGUCCCGGUCAGGUU
AACUCUGUAAUAA
>random_seq_from_cds__NO_626 RANDOM_LENGTH 80 SEQ_LENGTH 68 GC_CONTENT 0.470588235294118 BASEPAIR 23 FREE_ENERGY -19.20 LEN_BP_RATIO 2.95652173913043
AGGCUCUUCAUGAUUCUUUGGCUCAAAGGAGUUGUAUUUAGUGUGACGACUGUUGACCUGAAAAG
GGAAGCCAGCAGACC
>random_seq_from_cds__NO_639 RANDOM_LENGTH 71 SEQ_LENGTH 49 GC_CONTENT 0.63265306122449 BASEPAIR 19 FREE_ENERGY -20.80 LEN_BP_RATIO 2.57894736842105
AGCGGCACAGAGCAGGAUGACCUCUUCAGCUUCUGACCUACAGGCUGGACAGAUGGUGGGGGGCC

CCUGGG
>random_seq_from_cds__NO_659 RANDOM_LENGTH 75 SEQ_LENGTH 59 GC_CONTENT 0.610169491525424 BASEPAIR 19 FREE_ENERGY -23.50 LEN_BP_RATIO 3.10526315789474
UCCUGAGGAUCGUGCAGCCACGUGGCUGGGAUCUGCCACAACAUCCUGGUCUGCUGCCCCAAGGA
GCUGCUGGAA
>random_seq_from_cds__NO_661 RANDOM_LENGTH 87 SEQ_LENGTH 80 GC_CONTENT 0.625 BASEPAIR 28 FREE_ENERGY -35.10 LEN_BP_RATIO 2.85714285714286
ACCCAGGGUUCCCACUGACUCCCGACUGCAGAUCCAGCCUGGAGACGAGGUUGUCCAGAUCAACG
AGCAGGUGGUGGGUGGGAUGGC
>random_seq_from_cds__NO_669 RANDOM_LENGTH 70 SEQ_LENGTH 58 GC_CONTENT 0.517241379310345 BASEPAIR 21 FREE_ENERGY -16.60 LEN_BP_RATIO 2.76190476190476
CCAUGAUGUGUACAAACCCUUCAUCUUCGCUGCUGAUACCCUGACAGAUCUGAGCAUGGUGGGUG
CGUCA
>random_seq_from_cds__NO_673 RANDOM_LENGTH 110 SEQ_LENGTH 99 GC_CONTENT 0.626262626262626 BASEPAIR 35 FREE_ENERGY -45.00 LEN_BP_RATIO 2.82857142857143
CAGUGAAGAGGCACUGGAAGGAAUGGUACGGGGGCUGAGGCAGGGUGGCGUGUCCCUCCUAGGCC
AGCCACAGCCCCUGACCCAGGAACAGUGGCGGAGCUCUUUCAUGC
>random_seq_from_cds__NO_675 RANDOM_LENGTH 110 SEQ_LENGTH 97 GC_CONTENT 0.618556701030928 BASEPAIR 32 FREE_ENERGY -39.00 LEN_BP_RATIO 3.03125
CUGCAGGUCCUAGAAGAAGUGCUGGGUGACCCUGAGCUGACAGGAGAGAAGUUCCGCCAGUGGAA
GGAGCAGAACCGGGAGCUGUACUCAGAGGGCCUGGGGGCCUGGGG
>random_seq_from_cds__NO_682 RANDOM_LENGTH 84 SEQ_LENGTH 72 GC_CONTENT 0.569444444444444 BASEPAIR 26 FREE_ENERGY -21.71 LEN_BP_RATIO 2.76923076923077
CAUGGAGCCUUGUGGAGGGGUGCUGGCUGGUUCCGGUUUUAUGGAUGAGAAAUUGGAGAGGUUGA
UUAGGGCUGAGGGGCCAAA
>random_seq_from_cds__NO_698 RANDOM_LENGTH 72 SEQ_LENGTH 64 GC_CONTENT 0.765625 BASEPAIR 24 FREE_ENERGY -32.00 LEN_BP_RATIO 2.66666666666667
CCAGGUCUGUGGGCCGCGUCCGGCAGAGAUGGCCCUCUGGACUGGGGGGCGAGGCCAGGGCUGCA
CAGGGCC
>random_seq_from_cds__NO_710 RANDOM_LENGTH 73 SEQ_LENGTH 67 GC_CONTENT 0.656716417910448 BASEPAIR 20 FREE_ENERGY -20.90 LEN_BP_RATIO 3.35
GACCGCUGGUAUGCCAUCUGCCACCCACUAUUGUUCAAGAGCACAGCCCGGCGGGCCCGUGGCUC
CAUCCUGG
>random_seq_from_cds__NO_713 RANDOM_LENGTH 80 SEQ_LENGTH 62 GC_CONTENT 0.645161290322581 BASEPAIR 21 FREE_ENERGY -27.10 LEN_BP_RATIO 2.95238095238095

ACUGGGCCUCAUGGCCAUGGCCUAUUUCCAGAUAUUCCGCAAGCUCUGGGGCCGCCAGGAUCCCC
GGCACCACCUCAGCA
>random_seq_from_cds__NO_714 RANDOM_LENGTH 84 SEQ_LENGTH 75
GC_CONTENT 0.733333333333333 BASEPAIR 27 FREE_ENERGY
-38.60 LEN_BP_RATIO 2.77777777777778
CUGGUGCGGAACUGGAAGCGCCCCUCAGACCAGCUGGGGGACCUGGAGCAGGGCCUGAGUGGAGA
GCCCCAGCCCCGGGCCCGC
>random_seq_from_cds__NO_716 RANDOM_LENGTH 85 SEQ_LENGTH 78
GC_CONTENT 0.628205128205128 BASEPAIR 25 FREE_ENERGY
-24.92 LEN_BP_RATIO 3.12
UUCGCCCUCUGCUACCUGCCCAUCAGCGUCCUCAAUGUCCUUAAGAGGGGUGUUCGGGAUGUUCC
GCCAAGCCAGUGACCGCGAA
>random_seq_from_cds__NO_717 RANDOM_LENGTH 110 SEQ_LENGTH 87
GC_CONTENT 0.586206896551724 BASEPAIR 22 FREE_ENERGY
-25.64 LEN_BP_RATIO 3.95454545454545
GCUGUCUACGCCUGCUUCACCUUCUCCCACUGGCUGGUGUACGCCAACAGCGCUGCCAACCCCAU
CAUCUACAACUUCCUCAGUGGGCAAAUUCCGGGAGCAGUUUAAGG
>random_seq_from_cds__NO_720 RANDOM_LENGTH 64 SEQ_LENGTH 52
GC_CONTENT 0.596153846153846 BASEPAIR 20 FREE_ENERGY
-19.00 LEN_BP_RATIO 2.6
AUGGGUGGGUACUGCUGCUCCAUCGGCAUGGCCCCAAAGCAGUCAGAGGGAUUACAGUGGCCAG
>random_seq_from_cds__NO_723 RANDOM_LENGTH 110 SEQ_LENGTH 89
GC_CONTENT 0.685393258426966 BASEPAIR 30 FREE_ENERGY
-42.70 LEN_BP_RATIO 2.96666666666667
AAGCCCUGCCGUCCUUCUCUGCCAAUCUGACCAGGGAGCCCAGGUGACCCUGGAGCACCUGGCCU
GCCCGGAGCACCAUCCUUCCCUGGAGGCCCUGGUCGUCCCGGAGC
>random_seq_from_cds__NO_725 RANDOM_LENGTH 90 SEQ_LENGTH 65
GC_CONTENT 0.723076923076923 BASEPAIR 21 FREE_ENERGY
-28.90 LEN_BP_RATIO 3.0952380952381
GAUUUCAUCAUAAUUCACCAUGUCUCCCCAUGGAGCCAGGCACACCAGGCAAGCCAGGGCUCCCC
GAAGGCCCCGGAGCUCCAGGGUGGC
>random_seq_from_cds__NO_728 RANDOM_LENGTH 84 SEQ_LENGTH 77
GC_CONTENT 0.714285714285714 BASEPAIR 30 FREE_ENERGY
-43.70 LEN_BP_RATIO 2.56666666666667
CCCCUCUUUGCCAGCUGCACCAUCCGUACCAGGUUCGCCCUGGGGGGCCAGGGUGUCCAGGGGGG
CCGGGCUGGCCUGGGAGGC
>random_seq_from_cds__NO_739 RANDOM_LENGTH 78 SEQ_LENGTH 63
GC_CONTENT 0.698412698412698 BASEPAIR 23 FREE_ENERGY
-30.80 LEN_BP_RATIO 2.73913043478261
CCCGGAGGACCAGGUAGGCCUGGGCUCCCAACACAGCCAGGAUCUCCCUCACUGUUGUCACCCCC
GGGCCUCCUCGGC
>random_seq_from_cds__NO_763 RANDOM_LENGTH 68 SEQ_LENGTH 51
GC_CONTENT 0.705882352941177 BASEPAIR 19 FREE_ENERGY
-22.40 LEN_BP_RATIO 2.68421052631579

UGGACCAGGCAGGCCCCGCUCACCUUUCCCUCCCCUUGGGGCCAGAGGGAGCAAGUGUGACCAUU
GCU
>random_seq_from_cds__NO_766 RANDOM_LENGTH 73 SEQ_LENGTH 59
GC_CONTENT 0.610169491525424 BASEPAIR 23 FREE_ENERGY
-22.70 LEN_BP_RATIO 2.56521739130435
UAGACCUUGCCUUCAGACUGUGGAUUGAUCUCAAUGAGCUCAUUGCUCUGGGUGUCCCGGCGGGC
CUUGGAGG
>random_seq_from_cds__NO_790 RANDOM_LENGTH 99 SEQ_LENGTH 81
GC_CONTENT 0.703703703703704 BASEPAIR 26 FREE_ENERGY
-38.00 LEN_BP_RATIO 3.11538461538462
GGACGGCAGACAUCCAGGUGAGCGCCAGCAGGGGCAGCACCACGCAGGAGCUCCAGAGUGAGGCC
CAUGGCGUUCCUGGCCGAGGCUGAGCUGAGCAGG
>random_seq_from_cds__NO_792 RANDOM_LENGTH 80 SEQ_LENGTH 62
GC_CONTENT 0.451612903225806 BASEPAIR 22 FREE_ENERGY
-19.70 LEN_BP_RATIO 2.81818181818182
CGGCCCUCUGCUUCUUGGAUUUGUCGGAGAUGCCAUCACGUGCCAUGAGCUUGUUGAAGACGAUG
AUUCCGAUGAGCAUG
>random_seq_from_cds__NO_821 RANDOM_LENGTH 70 SEQ_LENGTH 59
GC_CONTENT 0.661016949152542 BASEPAIR 22 FREE_ENERGY
-26.20 LEN_BP_RATIO 2.68181818181818
CAGCCGCCGGGUCGCCCUGUCUGCGCCAUGUAUAGCCCAGGCUCAUCUGCAGACCUCGGCCACUG
GGUUU
>random_seq_from_cds__NO_830 RANDOM_LENGTH 85 SEQ_LENGTH 60
GC_CONTENT 0.783333333333333 BASEPAIR 21 FREE_ENERGY
-32.80 LEN_BP_RATIO 2.85714285714286
CGAGAAGGCCCCGUAGAGCACACCCGAGGCCAGGGCAGAGCAGGCACUGGGGGCGGGGUCGAAGG
CGGUGGCCAGGCGCAGGGAC
>random_seq_from_cds__NO_834 RANDOM_LENGTH 102 SEQ_LENGTH 89
GC_CONTENT 0.471910112359551 BASEPAIR 32 FREE_ENERGY
-26.60 LEN_BP_RATIO 2.78125
UCUUAUGUGAGCUCUACCACUGGGGGAGAGUGUGAUCACAAGAGAGAUGGUGGAGAUGCUCUUUU
CUGAUGAUUCUGACCUGCAGUUAGCAACCACACAGAA
>random_seq_from_cds__NO_848 RANDOM_LENGTH 100 SEQ_LENGTH 87
GC_CONTENT 0.551724137931034 BASEPAIR 26 FREE_ENERGY
-20.60 LEN_BP_RATIO 3.34615384615385
CGAGAACCAGGAGAUCUACCAGAAGGCCUUCGACCUCAUUGAGCACUACUUUGGUGUAGAAGACG
AUGAUAGCAGCCUGGCUCCCCAAGUCGAUGAAACG
>random_seq_from_cds__NO_854 RANDOM_LENGTH 83 SEQ_LENGTH 57
GC_CONTENT 0.596491228070175 BASEPAIR 19 FREE_ENERGY
-20.90 LEN_BP_RATIO 3
GUGAAACUUAAUAAGCAGCAGACGGACAUCGCUGUGAAUUGGGCUGGGGGCCUGCACCAUGCAAA
GAAGUCCGAGGCAUCUGG
>random_seq_from_cds__NO_855 RANDOM_LENGTH 88 SEQ_LENGTH 62
GC_CONTENT 0.5 BASEPAIR 22 FREE_ENERGY -19.80 LEN_BP_RATIO

2.81818181818182
CUUCUGUUACGUCAAUGAUAUCGUCUUGGCCAUCCUGGAACUGCUAAAGGUAUCACCAGAGGGUG
CUGUACAUUGACAUUGAUAUUCA
>random_seq_from_cds__NO_870 RANDOM_LENGTH 89 SEQ_LENGTH 81
GC_CONTENT 0.62962962962963 BASEPAIR 29 FREE_ENERGY
-32.50 LEN_BP_RATIO 2.79310344827586
CCAAGGCCCUCUUCCGUCAGAUGGUUGAGGCCAUCCGCUACUGCCAUGGCUGUGGUGUGGCCCAC
CGGGACCUCAAAUGUGAGAACGCC
>random_seq_from_cds__NO_872 RANDOM_LENGTH 88 SEQ_LENGTH 72
GC_CONTENT 0.611111111111111 BASEPAIR 25 FREE_ENERGY
-27.86 LEN_BP_RATIO 2.88
GUACAGCCUAUGCUGCCCCCGAGGUGCUGCAGGGCAUUCCCCACGAUAGCAAAAAAGGUGAUGUC
UGGAGCAUGGGUGUGGUCCUGUA
>random_seq_from_cds__NO_881 RANDOM_LENGTH 81 SEQ_LENGTH 70
GC_CONTENT 0.5 BASEPAIR 24 FREE_ENERGY -22.74 LEN_BP_RATIO
2.91666666666667
ACAUCCUUCUAAACCAGGAUCCUUCUGGAGAGUGCAACCCAGACUUGCGUCUCCGUGGACAUCAG
AAGGAAGGCUAUGGGC
>random_seq_from_cds__NO_884 RANDOM_LENGTH 69 SEQ_LENGTH 62
GC_CONTENT 0.451612903225806 BASEPAIR 21 FREE_ENERGY
-19.60 LEN_BP_RATIO 2.95238095238095
CUGGCAUCUACUCCAUGAGUCUCUGUUUGGGUCAGUUGCUGAUGAUCAGAAACUUAUGAUGUUGG
GAUA
>random_seq_from_cds__NO_885 RANDOM_LENGTH 119 SEQ_LENGTH 67
GC_CONTENT 0.477611940298507 BASEPAIR 23 FREE_ENERGY
-22.09 LEN_BP_RATIO 2.91304347826087
CUCGUUCAAACAAUACUUCCAAACCAAGCCACUCAGUUGAUGCUCACACUGCUGAAGUGAACUGC
CUUUCUUUCAAUCCUUAUAGUGAGUUCAUUCUUGCCACAGGAUCAGCUGACAAG
>random_seq_from_cds__NO_894 RANDOM_LENGTH 109 SEQ_LENGTH 93
GC_CONTENT 0.602150537634409 BASEPAIR 34 FREE_ENERGY
-34.70 LEN_BP_RATIO 2.73529411764706
UUGACACCAACAGCGAUGGCACCAUAGACUUUCGGGAGUUCAUCAUUGCGCUGAGCGUGACCUCG
CGCGGCCGCCUGGAGCAGAAGCUCAUGUGGGCCUUCAGCAUGUA
>random_seq_from_cds__NO_905 RANDOM_LENGTH 82 SEQ_LENGTH 72
GC_CONTENT 0.430555555555556 BASEPAIR 29 FREE_ENERGY
-21.70 LEN_BP_RATIO 2.48275862068966
UAUCAUCUGUUUUGAGGUGCUUAGGUGAAGGAGAAGUCAUGAAAGAUGGACCCCUUUUCUUUACC
CUGAGACUUAGACGAUU
>random_seq_from_cds__NO_906 RANDOM_LENGTH 72 SEQ_LENGTH 65
GC_CONTENT 0.492307692307692 BASEPAIR 19 FREE_ENERGY
-17.14 LEN_BP_RATIO 3.42105263157895
CCUUUGCCGCCUCAGCCUUGACAGGAGAAAGUGUAUGAAAGACAAAGUUUCUUGAAUUUCGAGGA
GCACUGG
>random_seq_from_cds__NO_907 RANDOM_LENGTH 79 SEQ_LENGTH 65

GC_CONTENT 0.553846153846154 BASEPAIR 22 FREE_ENERGY -21.50 LEN_BP_RATIO 2.95454545454545
GGUUAUGGUCAGAGAGAGCAGCCAGUUUCUGAAGCACAGCUUUGGGCUGGUUUAGCAGUGAGCCUGUCUUCACCCUGUU
>random_seq_from_cds__NO_939 RANDOM_LENGTH 110 SEQ_LENGTH 94 GC_CONTENT 0.372340425531915 BASEPAIR 30 FREE_ENERGY -18.50 LEN_BP_RATIO 3.13333333333333
UUGUCUGCUCAGAACCUUUACUAUGGUGAUCACUGUUCAUUUCAGUAGUAUUUGCAGUGUCUAUGAUUUCUUUGUGAUGGCUUGACUGAUAUUUAGAUGACCUUCAAUAG
>random_seq_from_cds__NO_946 RANDOM_LENGTH 85 SEQ_LENGTH 67 GC_CONTENT 0.432835820895522 BASEPAIR 25 FREE_ENERGY -15.90 LEN_BP_RATIO 2.68
CUUUAUCAUGUAUGUGCUUUUUGGAACUCUUUCUGUCAGUGGUAAAGUCUGUAGAGUUUCCAGACUGAAGACUCAGCUCUAAGCA
>random_seq_from_cds__NO_953 RANDOM_LENGTH 81 SEQ_LENGTH 63 GC_CONTENT 0.555555555555556 BASEPAIR 23 FREE_ENERGY -21.80 LEN_BP_RATIO 2.73913043478261
CUGGCCAAUUACUUUGAGGUGGACAUCCCUAAGAUCGACGUGUACCACUACGAGGUGGACAUCAAGCCGGAUAAGUGUCCC
>random_seq_from_cds__NO_954 RANDOM_LENGTH 67 SEQ_LENGTH 52 GC_CONTENT 0.5 BASEPAIR 19 FREE_ENERGY -16.20 LEN_BP_RATIO 2.73684210526316
CGUAGAGUCAACCGGGGAAGUGGUGGAAUACAUGGUCCAGCAUUUCAAGCCUCAGAUCUUUGGUGAU
>random_seq_from_cds__NO_980 RANDOM_LENGTH 110 SEQ_LENGTH 98 GC_CONTENT 0.612244897959184 BASEPAIR 34 FREE_ENERGY -39.60 LEN_BP_RATIO 2.88235294117647
CACACGCUCUGUCUCUAUCCCAGCACCUGCCUACUAUGCCCGCCUGGUGGCUUUCCGGGCACGAUACCACCUGGUGGACAAGGAGCAUGACAGGUGGAGAGGGGAGCCAC
>random_seq_from_cds__NO_1004 RANDOM_LENGTH 80 SEQ_LENGTH 70 GC_CONTENT 0.628571428571429 BASEPAIR 27 FREE_ENERGY -31.40 LEN_BP_RATIO 2.59259259259259
AUGGUGGUGGACUCCAUGAGCAGCGCGUAGUCGGCCGUCAGGGCCCUCUGGAUGCCCUCCUCGUUGUUCUUCACCAGCGC
>random_seq_from_cds__NO_1020 RANDOM_LENGTH 81 SEQ_LENGTH 73 GC_CONTENT 0.534246575342466 BASEPAIR 25 FREE_ENERGY -19.41 LEN_BP_RATIO 2.92
GUUGUCCACAUUGAGAAUCCGGAAUCCUGUCAGGUUCACGCCUGAGUAGCGGUAGGGCUCCAGGUCUAAAGCGUAGAGAUC
>random_seq_from_cds__NO_1023 RANDOM_LENGTH 71 SEQ_LENGTH 53 GC_CONTENT 0.566037735849057 BASEPAIR 19 FREE_ENERGY -17.40 LEN_BP_RATIO 2.78947368421053
GGAGCUGACGGAUCUUCAGGCGGAUGUUGUAUCUUGAUGGGGCCAUGAUGAGCUCCUGCAGUCGGAUGAGC

>random_seq_from_cds__NO_1025 RANDOM_LENGTH 90 SEQ_LENGTH 64 GC_CONTENT 0.640625 BASEPAIR 25 FREE_ENERGY -31.90 LEN_BP_RATIO 2.56
UAGAGGUUCACGUAGAAGGUGUCCUUGUUGUCCAGCGGGUGGUGCUUCCAACGCAGCUGGAUGUG
GGGCACCUCCAGGGCAUUGCAGAUG
>random_seq_from_cds__NO_1031 RANDOM_LENGTH 72 SEQ_LENGTH 57 GC_CONTENT 0.385964912280702 BASEPAIR 21 FREE_ENERGY -16.80 LEN_BP_RATIO 2.71428571428571
AAGCUAAAUAUGCAGCAAUUGCUAUCCAAAACCUUGGAUCUUUCAGCAGUGCUUUAUCAAAGCAG
CUAAACA
>random_seq_from_cds__NO_1057 RANDOM_LENGTH 96 SEQ_LENGTH 89 GC_CONTENT 0.539325842696629 BASEPAIR 33 FREE_ENERGY -42.80 LEN_BP_RATIO 2.6969696969697
CGAGACUUGAAUCUGCCCACAAAUCACUUUGCGGUCAUUGAGGAGCAUGCCAUUCAUCUUCUCGA
UGGCCUUGUCGGCAGCCUCUUGGGUCUCGAA
>random_seq_from_cds__NO_1062 RANDOM_LENGTH 86 SEQ_LENGTH 73 GC_CONTENT 0.63013698630137 BASEPAIR 24 FREE_ENERGY -35.84 LEN_BP_RATIO 3.04166666666667
CAGGUCGCCCACGUACAGGGAGGCCAUGGGGUAGCUGCUGGCCGCAGCGUUCAUGAUGGCCACCA
CCAAGCGCGUCUUGUACGUGG
>random_seq_from_cds__NO_1064 RANDOM_LENGTH 92 SEQ_LENGTH 69 GC_CONTENT 0.420289855072464 BASEPAIR 26 FREE_ENERGY -24.10 LEN_BP_RATIO 2.65384615384615
CAUCACAGAUAUUCAGAUUCCUCUGGAUUAUGAAACAGGAAAAGCACCGAGGAUUUGCUUUUGUU
GAAUUUGAGUUGGCAGAGGGAUGCUGC
>random_seq_from_cds__NO_1106 RANDOM_LENGTH 88 SEQ_LENGTH 63 GC_CONTENT 0.555555555555556 BASEPAIR 22 FREE_ENERGY -19.91 LEN_BP_RATIO 2.86363636363636
GCUUGGUGGAACCGUGGGGGACAUAGAAAGCAUGCCCUUUAUUGAGGCCUUCCGUCAGUUCCAAU
UCAAGGUCAAAAGAGAGAACUUU
>random_seq_from_cds__NO_1112 RANDOM_LENGTH 89 SEQ_LENGTH 71 GC_CONTENT 0.52112676056338 BASEPAIR 19 FREE_ENERGY -18.90 LEN_BP_RATIO 3.73684210526316
UGUCAUUAAGGCUCUGGAGCAUUCUGCACUGGCCAUCAACCACAAAUUGGAAAUCAAGGUACAUA
GAUUCUGCGGACUUGGAGCCCAUC
>random_seq_from_cds__NO_1114 RANDOM_LENGTH 78 SEQ_LENGTH 71 GC_CONTENT 0.52112676056338 BASEPAIR 27 FREE_ENERGY -23.10 LEN_BP_RATIO 2.62962962962963
UCCAAGCAAUUGCCUGGGCUCGGAAUCAGAAAAAGCCUUUUUUGGGGCGUGUGCUUAGGGAUGCA
GUUGGCAGUGGUU
>random_seq_from_cds__NO_1134 RANDOM_LENGTH 77 SEQ_LENGTH 55 GC_CONTENT 0.6 BASEPAIR 21 FREE_ENERGY -26.30 LEN_BP_RATIO 2.61904761904762
ACAGGAGGGGGCACCAGUGAUCGACACAUUCGCAUCUGGAAUGUGUGCUCUGGGGCCUGUCUGAG

UGCCGUGGAUGC
>random_seq_from_cds__NO_1143 RANDOM_LENGTH 85 SEQ_LENGTH 78 GC_CONTENT 0.602564102564103 BASEPAIR 27 FREE_ENERGY -32.20 LEN_BP_RATIO 2.88888888888889
GUCUGCGUGGGCAUCGUGGGCAGUGGGGCUGCCCUGGCCGAUGCUCAGAACCCCAGCCUCUUUGUAAAGAUUCUCAUCGUGGAGA
>random_seq_from_cds__NO_1172 RANDOM_LENGTH 84 SEQ_LENGTH 75 GC_CONTENT 0.44 BASEPAIR 24 FREE_ENERGY -19.10 LEN_BP_RATIO 3.125
GGAUCAUCGAUGUUGUCUACAAUGCAUCUAAUAACGAGCUGGUUCGUACCAAGACCCUGGUGAAGAAUUGCAUCGUGCUCAUCG
>random_seq_from_cds__NO_1181 RANDOM_LENGTH 92 SEQ_LENGTH 83 GC_CONTENT 0.686746987951807 BASEPAIR 28 FREE_ENERGY -38.90 LEN_BP_RATIO 2.96428571428571
CGGGGGCAAAUGUGUGCUCAAGGGCAUGGGCGGCCCGCAGGUUCCGGCUGCCCUGGGUGGUCAGGAAGCCCCAGAGCCACGUGGACUGUGAA
>random_seq_from_cds__NO_1196 RANDOM_LENGTH 94 SEQ_LENGTH 76 GC_CONTENT 0.644736842105263 BASEPAIR 25 FREE_ENERGY -36.90 LEN_BP_RATIO 3.04
UCAGAGCCCAGUGGGUCAGAAGGUGGGGGCACCAGGUGGGCUUGGGGAGGCAGGAUGGUGACCACAUGCUGGCUGCUGGCUUCACAGUGGGUAA
>random_seq_from_cds__NO_1208 RANDOM_LENGTH 77 SEQ_LENGTH 70 GC_CONTENT 0.685714285714286 BASEPAIR 23 FREE_ENERGY -28.70 LEN_BP_RATIO 3.04347826086957
GGAGCCCUGCCUGCUGUGAUGGUUGGGGGCACUAGGUGGGCAGUGGAGGUCAUCAGGGUGCAGACAGGGCCGCCCCA
>random_seq_from_cds__NO_1212 RANDOM_LENGTH 81 SEQ_LENGTH 74 GC_CONTENT 0.554054054054054 BASEPAIR 31 FREE_ENERGY -30.50 LEN_BP_RATIO 2.38709677419355
UCCCAUAGAGUGAUACUUGGACUUUACUGGCAGUGAGGGCUGCCUGGAGGUGGAGGCCAAGUGCUUCGGGUGUGAGGAUGU
>random_seq_from_cds__NO_1217 RANDOM_LENGTH 85 SEQ_LENGTH 75 GC_CONTENT 0.56 BASEPAIR 25 FREE_ENERGY -23.80 LEN_BP_RATIO 3
GCGGCGAUCGAGUCAUCAUGGAAGCGAAUGGGUUGGGGACCUCAGGGUUUUCCGGAGCUGAAGAAUGACACAUUCCUGCGAGCAG
>random_seq_from_cds__NO_1223 RANDOM_LENGTH 84 SEQ_LENGTH 67 GC_CONTENT 0.597014925373134 BASEPAIR 24 FREE_ENERGY -28.60 LEN_BP_RATIO 2.79166666666667
GUUGAGGGUGGUGGCUCAAGCACCAUGGCUCAGGCCAAGCGCUGGCUCUAUCAGAGACCUCAGGCUAGUCACCAGCUGCUUCGC
>random_seq_from_cds__NO_1243 RANDOM_LENGTH 67 SEQ_LENGTH 57 GC_CONTENT 0.43859649122807 BASEPAIR 20 FREE_ENERGY -15.10 LEN_BP_RATIO 2.85

CCGAUGAAAAAUGCCUUUGAAGUGAAGGUAGCUGAGGCCCACUGCUAUGUCAUAGGCCAGUUUUA
CC
>random_seq_from_cds__NO_1248 RANDOM_LENGTH 102 SEQ_LENGTH 90
GC_CONTENT 0.577777777777778 BASEPAIR 28 FREE_ENERGY
-32.30 LEN_BP_RATIO 3.21428571428571
UGGAGGAGGAGCUUGGCUCCCAGCCAGCUGGCCAAGAGAAAACCUGAAGGCAGGUCCUGUGAUGA
UGAAGACUGGCAACCUGGCCUAGUGGACUCCUAGGAA
>random_seq_from_cds__NO_1251 RANDOM_LENGTH 85 SEQ_LENGTH 62
GC_CONTENT 0.725806451612903 BASEPAIR 19 FREE_ENERGY
-33.00 LEN_BP_RATIO 3.26315789473684
AGGGUCCUCUGGGCUCUCGAGCAUUGGGCCUGAAAAGGGCUGGGGUCCGCCGGGCCCUCCAUGAC
CCCCUGGAAAAAGAUGCCUU
>random_seq_from_cds__NO_1257 RANDOM_LENGTH 81 SEQ_LENGTH 66
GC_CONTENT 0.515151515151515 BASEPAIR 23 FREE_ENERGY
-20.90 LEN_BP_RATIO 2.8695652173913
GUCUUCUCCCAUCCUCAUCAUUUCCUAUGAGACCUUCCGCCUUCAUGUUGGAGUCCUCCAGAAAG
GAAGUGUUGGUCUGGU
>random_seq_from_cds__NO_1265 RANDOM_LENGTH 84 SEQ_LENGTH 70
GC_CONTENT 0.585714285714286 BASEPAIR 26 FREE_ENERGY
-25.30 LEN_BP_RATIO 2.69230769230769
UCUAAGGCCCUGGAGCCCCAGCUGUCAGGGUAAGAUGCUGGUCCUGGAUUAUAUUCUGGCGGUGA
CCCGAAGCCGUAGCAGUGA
>random_seq_from_cds__NO_1272 RANDOM_LENGTH 72 SEQ_LENGTH 60
GC_CONTENT 0.616666666666667 BASEPAIR 21 FREE_ENERGY
-22.30 LEN_BP_RATIO 2.85714285714286
CCGUCAGAUCCGGCCACCCCCUGAUGGUUCUGACUGCACUUCAGACCUGGCAGGGUGGAACCACU
GCACUGA
>random_seq_from_cds__NO_1273 RANDOM_LENGTH 77 SEQ_LENGTH 57
GC_CONTENT 0.666666666666667 BASEPAIR 19 FREE_ENERGY
-26.80 LEN_BP_RATIO 3
UAAGUGGGGGCUCCGGGAUGAGGUACUCCAGGCUGCCUGGGAUGCUGCCUCCACUGCCAUCACCU
UCGUCUUCCACC
>random_seq_from_cds__NO_1275 RANDOM_LENGTH 98 SEQ_LENGTH 86
GC_CONTENT 0.813953488372093 BASEPAIR 37 FREE_ENERGY
-63.10 LEN_BP_RATIO 2.32432432432432
CCUGGCCUGCUGCUUCGUGGCGGCGGCCGUGGCCCUGCGCUGGUCCGGGCGCCGGACGGCGCGGG
GCGCGGUGGUCCGGGCGCGACAGAGGCAGCGAG
>random_seq_from_cds__NO_1276 RANDOM_LENGTH 86 SEQ_LENGTH 62
GC_CONTENT 0.741935483870968 BASEPAIR 25 FREE_ENERGY
-46.00 LEN_BP_RATIO 2.48
CGGGCCUGGAGAACAUGGACAGGGCGGCGCAGCGCUUCCGGCUCCAGGAACCCAGACCUGGACUC
AGAGGCGCUGCUAGCCCUGCC
>random_seq_from_cds__NO_1280 RANDOM_LENGTH 82 SEQ_LENGTH 71
GC_CONTENT 0.704225352112676 BASEPAIR 24 FREE_ENERGY

-30.90 LEN_BP_RATIO 2.95833333333333
UGAAGGGGUGCCGGCGGAGUGCGACAGCGUAGUGGUGCAUGUGCUGAAGCUGCAGGGUGCCGUGCCCUUCGUGCACACCAAU
>random_seq_from_cds__NO_1284 RANDOM_LENGTH 71 SEQ_LENGTH 56 GC_CONTENT 0.642857142857143 BASEPAIR 20 FREE_ENERGY -21.20 LEN_BP_RATIO 2.8
AGAGCCUGGCACUGUGCCUGCGAGCCCUGCUGUGUGAGGACAUGUUCCGCUUGGACCCCACUGUGCCUCCC
>random_seq_from_cds__NO_1286 RANDOM_LENGTH 87 SEQ_LENGTH 74 GC_CONTENT 0.702702702702703 BASEPAIR 26 FREE_ENERGY -33.70 LEN_BP_RATIO 2.84615384615385
CUAUACCAUGCCCUCCCCGGCCAUGAGGCGGGCCGUGCUGGAGACCAAACAGAGCCUUGAGGCUGCGGGGCACACGGCUGGUUCCCU
>random_seq_from_cds__NO_1306 RANDOM_LENGTH 86 SEQ_LENGTH 66 GC_CONTENT 0.560606060606061 BASEPAIR 25 FREE_ENERGY -27.30 LEN_BP_RATIO 2.64
CAGUGGUAUCUCCUGGUUUCUCUACUGCAUGGCCCUGUACCCUGAGCACCAGCAUCGUUGUAGAGAGGAGGUCCGCGAGAUCCUAG
>random_seq_from_cds__NO_1310 RANDOM_LENGTH 83 SEQ_LENGTH 72 GC_CONTENT 0.555555555555556 BASEPAIR 24 FREE_ENERGY -24.40 LEN_BP_RATIO 3
ACUGAGAAUGCAUCCAAACGCCAUCCCUUUGCCUUUAUGCCCUUCUCUGCUGGGCCCAGGGAACUGCAUUGGGCAGCAGUUUG
>random_seq_from_cds__NO_1319 RANDOM_LENGTH 82 SEQ_LENGTH 49 GC_CONTENT 0.510204081632653 BASEPAIR 19 FREE_ENERGY -20.90 LEN_BP_RATIO 2.57894736842105
CACCUCAGCACGGAGGUCCUUGUCUGACAAGAUGCUCCCAUUCUCCAUCUUUGGCCAAGAGGAGGAUAUCCAGAAAAUCCAA
>random_seq_from_cds__NO_1322 RANDOM_LENGTH 68 SEQ_LENGTH 59 GC_CONTENT 0.542372881355932 BASEPAIR 19 FREE_ENERGY -17.20 LEN_BP_RATIO 3.10526315789474
GGUCACUAAUGGCCUGUAUGUAGGACUGAGAAUUCCCUGUCCACCUGGAUGCUGCCCUGAUGGCUGAA
>random_seq_from_cds__NO_1324 RANDOM_LENGTH 106 SEQ_LENGTH 94 GC_CONTENT 0.574468085106383 BASEPAIR 32 FREE_ENERGY -35.60 LEN_BP_RATIO 2.9375
AGGAGCUCUUCCCAUUUGUCCCAGCAUCACUCGUACAGAGUCUGCCAUGAGCCCCACAUAGGGCUUCAGGAUGUCAUAGUGGAAGGCUGGGGUCAGCAUCCGUCGA
>random_seq_from_cds__NO_1335 RANDOM_LENGTH 96 SEQ_LENGTH 69 GC_CONTENT 0.405797101449275 BASEPAIR 27 FREE_ENERGY -19.40 LEN_BP_RATIO 2.55555555555556
AUUCCAUUUUAACAUCCUGAAAGCAUACAUUGAGGUGAUGGCUCAUUCUGUGAAAAUGAUGCUGGGAUAAGUGGGAGAAGAUUUGCAGCACUCAGG
>random_seq_from_cds__NO_1336 RANDOM_LENGTH 67 SEQ_LENGTH 57

GC_CONTENT 0.43859649122807 BASEPAIR 21 FREE_ENERGY
-18.20 LEN_BP_RATIO 2.71428571428571
ACACAAGCGUGGAGGUCUAUGAGCACAUCAACUCGAUGUCUCUGGAUAUAAUCAUGAAAUGCGCU
UU
>random_seq_from_cds__NO_1345 RANDOM_LENGTH 83 SEQ_LENGTH 73
GC_CONTENT 0.465753424657534 BASEPAIR 27 FREE_ENERGY
-15.70 LEN_BP_RATIO 2.7037037037037
UUGAGGUUCUCUCAGGAGAAUUCUGAUCAGAGACACCCCUAUGCCUACUUACCAUUCUCAGCUGG
AUCAAGGGAACUGCAUUG
>random_seq_from_cds__NO_1346 RANDOM_LENGTH 82 SEQ_LENGTH 70
GC_CONTENT 0.485714285714286 BASEPAIR 22 FREE_ENERGY
-21.10 LEN_BP_RATIO 3.18181818181818
GGCAGGAGUUUGCCAUGAUUGAGUUAAAGGUAACCAUUGCCUUGAUUCUGCUCCACUUCAGAGUG
ACUCCAGACCCCACCAG
>random_seq_from_cds__NO_1349 RANDOM_LENGTH 110 SEQ_LENGTH 99
GC_CONTENT 0.494949494949495 BASEPAIR 31 FREE_ENERGY
-23.70 LEN_BP_RATIO 3.19354838709677
CCUACCAGAUGUACCUUGGAAGCAGGAGCCAUGACUUUUCAGCUACAUUAUUGAGCAGGGGGUGU
GUGAUUUGGUUUUAUGUGAAGCUGCCUUCCCUAAGACGUUGGCUU
>random_seq_from_cds__NO_1352 RANDOM_LENGTH 82 SEQ_LENGTH 64
GC_CONTENT 0.46875 BASEPAIR 20 FREE_ENERGY -15.50
LEN_BP_RATIO 3.2
AGAGGAUCAUGGUGGCCAAUAUCGAAGAAGUGUUACAACGAGGAGAAGCACUCUCAGGCAUUGGA
UUCAAAGGCUAACAAUU
>random_seq_from_cds__NO_1354 RANDOM_LENGTH 88 SEQ_LENGTH 66
GC_CONTENT 0.772727272727273 BASEPAIR 24 FREE_ENERGY
-30.10 LEN_BP_RATIO 2.75
UGUUAAUAGUGUAUGUCCGAUUCUGGUGGCUGUGAGAUGGUGCCCCGCCUGCUGCUGCGCGCCUG
GCCCCGGGGCCCCGCGGUUGGUC
>random_seq_from_cds__NO_1372 RANDOM_LENGTH 68 SEQ_LENGTH 49
GC_CONTENT 0.591836734693878 BASEPAIR 19 FREE_ENERGY
-23.40 LEN_BP_RATIO 2.57894736842105
GCCCUCCAGGCACAGUGCUGGUGAGCUUCAGCAGAUGAUGGUUGAGUGCUCCAAGUACCAUGGCC
AGC
>random_seq_from_cds__NO_1373 RANDOM_LENGTH 99 SEQ_LENGTH 72
GC_CONTENT 0.569444444444444 BASEPAIR 24 FREE_ENERGY
-27.90 LEN_BP_RATIO 3
UGACCAAAGAAGCAGCAAUGGGGCCAGGGCUUUGACCGACACUUGUUUGCUCUGCGGCAUCUGGC
AGCAGCCAAAGGGAUCAUCUUGCCUGAGCUCUAC
>random_seq_from_cds__NO_1378 RANDOM_LENGTH 110 SEQ_LENGTH 96
GC_CONTENT 0.333333333333333 BASEPAIR 27 FREE_ENERGY
-20.81 LEN_BP_RATIO 3.55555555555556
CUGGACAAGAUAAUAAAAUACUCGUAAGCCUUCUGGAUCCCUUGGAUUGAUUGACAUCAAUAAGG
GAACCAAUUUUUGAUGUUGUAAAAGAAAUGUGUUCAUCUCCAAUG

>random_seq_from_cds__NO_1406 RANDOM_LENGTH 75 SEQ_LENGTH 58 GC_CONTENT 0.431034482758621 BASEPAIR 20 FREE_ENERGY -17.10 LEN_BP_RATIO 2.9
CAAGUUCAUCAAUGACUAUGGCACCCAUUACAUCACAUCUGGAUCCAUGGGUGGCAUUUAUGAAU
AUAUCCUGGU
>random_seq_from_cds__NO_1417 RANDOM_LENGTH 75 SEQ_LENGTH 66 GC_CONTENT 0.560606060606061 BASEPAIR 21 FREE_ENERGY -23.40 LEN_BP_RATIO 3.14285714285714
CAAUGGGGGUAUCUCUUCCGAUAGGAGACCUCACAGGCUAGGCCUUGGGAUCCAACAGGACAGAU
GCAGUCACAG
>random_seq_from_cds__NO_1420 RANDOM_LENGTH 102 SEQ_LENGTH 88 GC_CONTENT 0.636363636363636 BASEPAIR 30 FREE_ENERGY -35.60 LEN_BP_RATIO 2.93333333333333
GAUGAUGGCUGGGUUGUACUGCACAGCGUCUCCCCACUCCUGCAUCAGGUCCGCCGUCGGCAGCU
CCUGGUAUGCCAGGGUGGUGAUGUGCUCACUUGCCCC
>random_seq_from_cds__NO_1423 RANDOM_LENGTH 81 SEQ_LENGTH 64 GC_CONTENT 0.484375 BASEPAIR 21 FREE_ENERGY -17.60 LEN_BP_RATIO 3.04761904761905
CAUGGCCUCUUUGUUCAUAACGAGGGUGUAUUCAUAAAUGCCCCCAAGCACAGCCUCUGUGAUGU
AGUGGGUCCCAAAAUC
>random_seq_from_cds__NO_1470 RANDOM_LENGTH 110 SEQ_LENGTH 105 GC_CONTENT 0.419047619047619 BASEPAIR 33 FREE_ENERGY -31.40 LEN_BP_RATIO 3.18181818181818
GAGAUGUCAAUGCUGAAUGCAUAUAAACCCAAGGAGCAAGGCUAAAGAGCUACCUCUUUCUGCUG
UACGCUUUAUGGAAGAAUUGGGUGAGUGUGCCUUUGGAAAAAUCU
>random_seq_from_cds__NO_1473 RANDOM_LENGTH 85 SEQ_LENGTH 74 GC_CONTENT 0.445945945945946 BASEPAIR 26 FREE_ENERGY -29.82 LEN_BP_RATIO 2.84615384615385
GUGCAUGCUUUUUGAGUAUAUUAAUCAGGGGGAUCUCCAUGAGUUCCUCAUCAUGAGAUCCCCAC
ACUCUGAUGUUGGCUGCAGC
>random_seq_from_cds__NO_1495 RANDOM_LENGTH 110 SEQ_LENGTH 96 GC_CONTENT 0.572916666666667 BASEPAIR 28 FREE_ENERGY -27.79 LEN_BP_RATIO 3.42857142857143
ACUCUCAGGCUUCAGAGAUUUAACAGCCACCUGCUCCCCUGUAUUGUCCCCUUCGGGGUCAUACC
UGCAGAGCUCAACCUUCCCAAAGUGGCCCCUCUCCCAAGUCACGG
>random_seq_from_cds__NO_1507 RANDOM_LENGTH 86 SEQ_LENGTH 71 GC_CONTENT 0.577464788732394 BASEPAIR 27 FREE_ENERGY -25.30 LEN_BP_RATIO 2.62962962962963
GGUUGGAGAUUUCCUCGGGGCUUGGGCUGGCAGCAGCGUUUUAGCAUGAAGCUGAUGUUAUCCGU
GCGCAGGAUCUGCUUCUUGAG
>random_seq_from_cds__NO_1522 RANDOM_LENGTH 86 SEQ_LENGTH 66 GC_CONTENT 0.454545454545455 BASEPAIR 24 FREE_ENERGY -18.20 LEN_BP_RATIO 2.75
AUUUUUCUGCUUCUUUGGAGAAUGACGCCACACUGACUGCUCAUUGUCGUUGGUUCCAUGCCAAU

UGGUGAAAUAGAACCCUCAUC
>random_seq_from_cds__NO_1530 RANDOM_LENGTH 90 SEQ_LENGTH 73
GC_CONTENT 0.397260273972603 BASEPAIR 25 FREE_ENERGY
-15.90 LEN_BP_RATIO 2.92
UUCUGGAACCACAUGCUUCUAUGUACUGCACUGCUGAAUGUCCCAAACAUUUUCAAGAGACACUG
AUAUGUGGAAAAGACAUUUCUUCUG
>random_seq_from_cds__NO_1558 RANDOM_LENGTH 89 SEQ_LENGTH 76
GC_CONTENT 0.368421052631579 BASEPAIR 26 FREE_ENERGY
-15.70 LEN_BP_RATIO 2.92307692307692
GGCCAAAGGAAGACAUGAUUUGCUGGAUCUGAAACCAUUUACAGAAUAUGAAUUUCAGAUUUCCU
CUAAGCUACAUCUUUAUAAGGGAA
>random_seq_from_cds__NO_1563 RANDOM_LENGTH 74 SEQ_LENGTH 56
GC_CONTENT 0.607142857142857 BASEPAIR 20 FREE_ENERGY
-25.20 LEN_BP_RATIO 2.8
CGUAUUAACAUAAUGAACCUGUGUGAGGCAGGGGUUGCUGGCUCCUCGCCAGGUCUCUGCAAACU
CAGAGGGCA
>random_seq_from_cds__NO_1571 RANDOM_LENGTH 100 SEQ_LENGTH 81
GC_CONTENT 0.481481481481481 BASEPAIR 26 FREE_ENERGY
-22.83 LEN_BP_RATIO 3.11538461538462
CAUGGUGGGCAUUUUCUCAACGCAUUACUUCCAGCAAAAGGGUGUUUGUUCUCCUAGCAGCCCUC
AGACCUCAGUGGUGUAGCAGAGAAAUUCCAGAUCC
>random_seq_from_cds__NO_1579 RANDOM_LENGTH 82 SEQ_LENGTH 67
GC_CONTENT 0.626865671641791 BASEPAIR 23 FREE_ENERGY
-21.60 LEN_BP_RATIO 2.91304347826087
GAUAAGGUGGGGGAUGCCCUGGAGGAAGUGCUCAGCAAAGCCCUGAGUCAGCGCACGAUCACUGU
CGGGGUGUACGAAGCGG
>random_seq_from_cds__NO_1582 RANDOM_LENGTH 71 SEQ_LENGTH 63
GC_CONTENT 0.80952380952381 BASEPAIR 23 FREE_ENERGY
-34.90 LEN_BP_RATIO 2.73913043478261
UGCGCGUCAGCAACCCGGGCCGGCUGGCGGAGCUCCUGCUCUUGGAGACCGACGCUGGCCCCGCG
GCGAGC
>random_seq_from_cds__NO_1585 RANDOM_LENGTH 110 SEQ_LENGTH 100
GC_CONTENT 0.76 BASEPAIR 37 FREE_ENERGY -57.80
LEN_BP_RATIO 2.7027027027027
CCUGAACGGUGACAUGAGCAACACUACCGUCGUCCCCAGCACUGCAGGUCCGGGCCCCAGCGGCG
GGCCCGGUGGCGGAGGUGGUGGUGGCGGCGGAGGCGGCGGCACCG
>random_seq_from_cds__NO_1587 RANDOM_LENGTH 73 SEQ_LENGTH 62
GC_CONTENT 0.516129032258065 BASEPAIR 21 FREE_ENERGY
-19.60 LEN_BP_RATIO 2.95238095238095
UUCGCCUUUGCCAGUCUCAUCUCGUGUCUGCUUUGUUAAGUUCCAUGAUCCAGACUCAGCAGUUG
UGGCACAG
>random_seq_from_cds__NO_1633 RANDOM_LENGTH 81 SEQ_LENGTH 68
GC_CONTENT 0.426470588235294 BASEPAIR 24 FREE_ENERGY
-16.90 LEN_BP_RATIO 2.83333333333333

UUUUCUACUAUAAGUACCAGAGCGAAUGUAUGUCUCCACGGGGUUGACACCACAUGCAUGGACCU
UGAUUAGAACCCUGAU
>random_seq_from_cds__NO_1636 RANDOM_LENGTH 87 SEQ_LENGTH 73 GC_CONTENT 0.506849315068493 BASEPAIR 25 FREE_ENERGY -18.10 LEN_BP_RATIO 2.92
UGCCCAUAAUCCCAGAAUAAAGCCCCCAGGAGGAUGCCAGUCUUUCAGCUUCGGUUUCCUGGUGU
AUUAUCUGAGCUACAGGAAGUU
>random_seq_from_cds__NO_1646 RANDOM_LENGTH 110 SEQ_LENGTH 90 GC_CONTENT 0.433333333333333 BASEPAIR 33 FREE_ENERGY -26.30 LEN_BP_RATIO 2.72727272727273
AACACUGUAGCUGGUUUGGGAUUUCUAGGAUUACAGGCCAUAUCAUCUGCAAGCAUUAGGACAAU
GUGACCUGUCAGGAAUACCUAGCCUCUUGACACUUCUAUAAACAG
>random_seq_from_cds__NO_1655 RANDOM_LENGTH 87 SEQ_LENGTH 77 GC_CONTENT 0.649350649350649 BASEPAIR 32 FREE_ENERGY -45.80 LEN_BP_RATIO 2.40625
GAGGCCCAGGAGGUCCAUGGGGGCCUGGGACACCAUGGGGCCCAUGGGGUACAGGUGGCCCUAAA
GGAUUUACUGGGCCCACCAAUC
>random_seq_from_cds__NO_1675 RANDOM_LENGTH 89 SEQ_LENGTH 82 GC_CONTENT 0.609756097560976 BASEPAIR 28 FREE_ENERGY -25.00 LEN_BP_RATIO 2.92857142857143
CACCAGGCCCGGGGUCCAGCAUACCACAAACGCCCCCUAAGACAGUCAUCACCGUCUUCAUUAGC
UUCAUGGGUGUCCUCCGGCGGCUG
>random_seq_from_cds__NO_1680 RANDOM_LENGTH 68 SEQ_LENGTH 53 GC_CONTENT 0.415094339622642 BASEPAIR 19 FREE_ENERGY -16.70 LEN_BP_RATIO 2.78947368421053
UUUUUGAAACUGGGCCUGUGUUAAACAUCAGGAAUACAUAGGCAAUUCCAGCGAAGAAAUCGGCA
GCA
>random_seq_from_cds__NO_1683 RANDOM_LENGTH 109 SEQ_LENGTH 91 GC_CONTENT 0.373626373626374 BASEPAIR 31 FREE_ENERGY -24.50 LEN_BP_RATIO 2.93548387096774
CAGUGUUGCUCCUAUUAUAAAAAAAGUCCAUGUGCUUGUCAUAGUGACACUCAUUCAUGUCAUUG
UCGUGAAACAGUACGUGAUCUUAAGGGAAGAAACAUCUCACUAG
>random_seq_from_cds__NO_1709 RANDOM_LENGTH 70 SEQ_LENGTH 50 GC_CONTENT 0.44 BASEPAIR 20 FREE_ENERGY -19.00 LEN_BP_RATIO 2.5
CAUCAAUUGAAAAACACAGUGACUGUGGAUAAUACUGUGGGCAACGACACUAUGUUUCUAGUUAC
GUGGC
>random_seq_from_cds__NO_1727 RANDOM_LENGTH 82 SEQ_LENGTH 63 GC_CONTENT 0.492063492063492 BASEPAIR 20 FREE_ENERGY -17.20 LEN_BP_RATIO 3.15
GAAGACAAAGGCUGACUAUGUGAGACCAAAACUUGAGACCUACAAAAAUGGCUGAUGUUCUGGUU
GCUGAGUCUACUCCUCC
>random_seq_from_cds__NO_1738 RANDOM_LENGTH 82 SEQ_LENGTH 62 GC_CONTENT 0.483870967741936 BASEPAIR 22 FREE_ENERGY

-18.31 LEN_BP_RATIO 2.81818181818182
CGGAUGGGGAAGACAACACUAUAAGUGGGUGCUUUAACGAGGUCAAACAAAGUGGUGCCAUCAUC
CACACAGUCGCUUUGGG
>random_seq_from_cds__NO_1774 RANDOM_LENGTH 94 SEQ_LENGTH 73
GC_CONTENT 0.465753424657534 BASEPAIR 24 FREE_ENERGY
-20.20 LEN_BP_RATIO 3.04166666666667
GGAAAAUUUCACAGUGGAUGCAACUUCCAAAAUGGCCUAUCUCAGUAUUCCAGGAACUGCAAAGG
GUGGGCACUUGGGCAUACAAUCUUCAAGC
>random_seq_from_cds__NO_1787 RANDOM_LENGTH 87 SEQ_LENGTH 57
GC_CONTENT 0.385964912280702 BASEPAIR 20 FREE_ENERGY
-15.50 LEN_BP_RATIO 2.85
AUUGAAAAGCUCACUGGUAACUUUGAAUAACAAUGGAUAUGAUGGCAUUGUCAUUGCAAUUAAUC
CCAGUGUACCAGAAGAUGAAAA
>random_seq_from_cds__NO_1833 RANDOM_LENGTH 71 SEQ_LENGTH 62
GC_CONTENT 0.564516129032258 BASEPAIR 23 FREE_ENERGY
-22.40 LEN_BP_RATIO 2.69565217391304
UCCUGGAGGGUUUCUGUGGGCAGCUGCACCUUCUGGCCCAUCUGCUGUUCAUAGUGGGCAAUAGC
CUUUUG
>random_seq_from_cds__NO_1844 RANDOM_LENGTH 79 SEQ_LENGTH 68
GC_CONTENT 0.529411764705882 BASEPAIR 25 FREE_ENERGY
-25.20 LEN_BP_RATIO 2.72
UCUGGAUUCGCCAUCAGUCGCCCAUUAGUGUUCUCAAUGAGGCACAUUGGGCCUGUCAUGUGGAU
CUCUGAUGCCAUGA
>random_seq_from_cds__NO_1846 RANDOM_LENGTH 102 SEQ_LENGTH 60
GC_CONTENT 0.4 BASEPAIR 25 FREE_ENERGY -15.30 LEN_BP_RATIO
2.4
UGAUACAAGAUAAAAAUAAAUACAACACACCCAAAUACAGGAUGAUAGUUCGUGUGACAAACAGA
GAUAUCAUUUGUCAGGAUUGCUUAUGCCCGUAUAGAG
>random_seq_from_cds__NO_1849 RANDOM_LENGTH 62 SEQ_LENGTH 51
GC_CONTENT 0.490196078431373 BASEPAIR 21 FREE_ENERGY
-21.80 LEN_BP_RATIO 2.42857142857143
CAAGUGGAGGUGACUGGUGAUGAAUACAAUGUGGAAAGCAUUGAUGGUCAGCCAGGUGCCUU
>random_seq_from_cds__NO_1855 RANDOM_LENGTH 98 SEQ_LENGTH 76
GC_CONTENT 0.5 BASEPAIR 26 FREE_ENERGY -29.50 LEN_BP_RATIO
2.92307692307692
GAAGAAGGAUCGGGUAGCUCAAAAGAAGGCAAGCUUCCUCAGAGCUCAGGAGCGGGCUGCUGAGA
GCUAAAUCAAAGCUCUGCCUGCUUUACAGGAGG
>random_seq_from_cds__NO_1864 RANDOM_LENGTH 68 SEQ_LENGTH 52
GC_CONTENT 0.557692307692308 BASEPAIR 20 FREE_ENERGY
-18.00 LEN_BP_RATIO 2.6
CCACUGCAAUGCCGAUGGCCAUUGUGUUGUGUCCUUUCAAUUAUGUCCAGGCGCAGCUGGUGUCC
GUG
>random_seq_from_cds__NO_1869 RANDOM_LENGTH 81 SEQ_LENGTH 59
GC_CONTENT 0.627118644067797 BASEPAIR 22 FREE_ENERGY

-27.70 LEN_BP_RATIO 2.68181818181818
GUAGUUGACGCCAGAGUUCCGGGGACCUGGCGCCUCAUUCUGCUUGGCUGUGAGCAGUUCUGUGU
GGCUUCCCUGGGCACA
>random_seq_from_cds__NO_1877 RANDOM_LENGTH 92 SEQ_LENGTH 75
GC_CONTENT 0.666666666666667 BASEPAIR 30 FREE_ENERGY
-37.30 LEN_BP_RATIO 2.5
GGUUGCCCACGUAGCAGCGCACCAGGCCGGGGAUGGAGUCGAAGCUCUCCAUCUCGAACUGGUAC
UGCACGCGGCUGUAGGCCUCGCUGAGU
>random_seq_from_cds__NO_1887 RANDOM_LENGTH 110 SEQ_LENGTH 97
GC_CONTENT 0.463917525773196 BASEPAIR 33 FREE_ENERGY
-27.00 LEN_BP_RATIO 2.93939393939394
AGUCAAAUCUGGUGGCAUCACACAGCAGGAGGCAAGAUUAACUUGGUUACUAUUUGGUUUUACCC
UGUGCCCACUGAUACAGCUGUUCCAACUGUGUUUUGUCUAGAUCA
>random_seq_from_cds__NO_1897 RANDOM_LENGTH 89 SEQ_LENGTH 78
GC_CONTENT 0.487179487179487 BASEPAIR 27 FREE_ENERGY
-24.50 LEN_BP_RATIO 2.88888888888889
ACAGGGUUCAGGUCAGGAAGCAGGUCGUCCUUCGGGGAUUUGAUCUUCAUUGUGACGAUAUAGCC
AUCUCCAAAUCUUGGACUUGAGAU
>random_seq_from_cds__NO_1911 RANDOM_LENGTH 72 SEQ_LENGTH 52
GC_CONTENT 0.538461538461538 BASEPAIR 19 FREE_ENERGY
-16.40 LEN_BP_RATIO 2.73684210526316
AUCCCAUACAGCAGGAGCAGUGCCACAAGGGCAGGAAGGUUUUCUGGAGAAGUGUAGGCUUUCUU
CUGAAAC
>random_seq_from_cds__NO_1914 RANDOM_LENGTH 80 SEQ_LENGTH 57
GC_CONTENT 0.491228070175439 BASEPAIR 20 FREE_ENERGY
-20.30 LEN_BP_RATIO 2.85
GAAGGACAUGGAGAAAAUCACGCAGAUGGCAACCACAGCAUCCACUGAAGUGGUCAGCCACUGUA
AUCUCUGAGAGCUGC
>random_seq_from_cds__NO_1930 RANDOM_LENGTH 110 SEQ_LENGTH 101
GC_CONTENT 0.475247524752475 BASEPAIR 29 FREE_ENERGY
-25.17 LEN_BP_RATIO 3.48275862068966
CUUAUUUGGAAGAAGGAAGAUAAGUUCUUGACCAAUGCACUCCACCAGCUUUGCCUCUGGAACAU
GGUGGAGAACUACAUCCAUCAGCUCAUUUACAUCCCCCAUCCAGG
>random_seq_from_cds__NO_1940 RANDOM_LENGTH 98 SEQ_LENGTH 83
GC_CONTENT 0.542168674698795 BASEPAIR 24 FREE_ENERGY
-28.90 LEN_BP_RATIO 3.45833333333333
AGUCCGUGUAUUCCUUCUGGGUGCUCUGGAUCCUCCGUUUCCUCUGUUAGGGGCUCGGUCUUUUC
CAGGGCUCUUUCUUCUCUGGUUGAACACCCCUU
>random_seq_from_cds__NO_1946 RANDOM_LENGTH 92 SEQ_LENGTH 65
GC_CONTENT 0.476923076923077 BASEPAIR 26 FREE_ENERGY
-21.70 LEN_BP_RATIO 2.5
ACAGGAAGAGGAUGAAUGGGUCGCUGUAAUGUAGGAUUCUUCCAUGCCAUGAUGAAUAUCGUCAG
GAGGAAGAUGCUCAUCGACAUGAUGGA
>random_seq_from_cds__NO_1953 RANDOM_LENGTH 84 SEQ_LENGTH 77

GC_CONTENT 0.519480519480519 BASEPAIR 28 FREE_ENERGY -22.40 LEN_BP_RATIO 2.75

CAUGUCAGGGAAUACCACUCCGGCCCAGAACAUGUUUUCCUCCAGUAGAGAGAGGGCACGUUGGGUGAGCUGAGUUUCAUCAUU

>random_seq_from_cds__NO_1959 RANDOM_LENGTH 81 SEQ_LENGTH 70 GC_CONTENT 0.4 BASEPAIR 28 FREE_ENERGY -19.70 LEN_BP_RATIO 2.5

CUGGAUCAAUGCAUUACAAAAGGAUGCUUGUUCUUCUGUCAUAAGAAUAGAUAGGAUCCUUCCUUGUGGAGUCAAUCCCCA

>random_seq_from_cds__NO_1970 RANDOM_LENGTH 72 SEQ_LENGTH 49 GC_CONTENT 0.469387755102041 BASEPAIR 20 FREE_ENERGY -18.90 LEN_BP_RATIO 2.45

UGUUGGGGAAAUGGCCAUUCAUGAUGGCUGUAGAGUGGGUUGGCAUUCCUUAACCAGAUCAAGACCAGAAAU

>random_seq_from_cds__NO_1998 RANDOM_LENGTH 70 SEQ_LENGTH 58 GC_CONTENT 0.431034482758621 BASEPAIR 20 FREE_ENERGY -16.60 LEN_BP_RATIO 2.9

UCUUCCGGUUAACUGUUCGGGAGGGAAUCACUGCUUGAACACUGAAACAGUAGUUUUCUCCUUUAUCCAC

>random_seq_from_cds__NO_2005 RANDOM_LENGTH 63 SEQ_LENGTH 54 GC_CONTENT 0.796296296296296 BASEPAIR 19 FREE_ENERGY -27.10 LEN_BP_RATIO 2.84210526315789

ACCUGGGCGAAGACCCAGCCGAGCAGGAGCGUCCGAGCGACGGCGGUCUCGGGGCGCGGGACC

>random_seq_from_cds__NO_2007 RANDOM_LENGTH 84 SEQ_LENGTH 61 GC_CONTENT 0.459016393442623 BASEPAIR 22 FREE_ENERGY -17.50 LEN_BP_RATIO 2.77272727272727

CUGACGAACUACUCUCAGGCAGUGUUCUCAGUAGUCCGAACUCUAAUAUGAGCAGCAUGGUAGUUACAGGCCAAUGGUAAUGAU

>random_seq_from_cds__NO_2020 RANDOM_LENGTH 90 SEQ_LENGTH 69 GC_CONTENT 0.405797101449275 BASEPAIR 24 FREE_ENERGY -16.50 LEN_BP_RATIO 2.875

AGACAGCGCUCUAAUACAGAUGGCUGAUGGAAACCAAUCACAACUUGGCCAUGAAUCAUCUUAAUGGACAGAAAAUGUAUGGAAAAAUUA

>random_seq_from_cds__NO_2031 RANDOM_LENGTH 75 SEQ_LENGTH 54 GC_CONTENT 0.518518518518518 BASEPAIR 21 FREE_ENERGY -24.50 LEN_BP_RATIO 2.57142857142857

UCCAUGAGUUCAGCUAUACGUGGAACUGGUUUCCCUUUCUGGUGACUCACAGUAGCUGGACUCUGUCCAUCCCAG

>random_seq_from_cds__NO_2038 RANDOM_LENGTH 81 SEQ_LENGTH 75 GC_CONTENT 0.546666666666667 BASEPAIR 27 FREE_ENERGY -27.30 LEN_BP_RATIO 2.77777777777778

UUGUCCAGGGCCAUACAUGGGGCCAGAGGUGGUUCCCCGGAUGAUUCUGGGGGAAACAUUUGUCACAAUGUCCCUUAUCAA

>random_seq_from_cds__NO_2053 RANDOM_LENGTH 88 SEQ_LENGTH 77

GC_CONTENT 0.493506493506494 BASEPAIR 30 FREE_ENERGY -27.80 LEN_BP_RATIO 2.56666666666667
UGGGGGAGGCAGCGAAGGAUUUCUGAUCUGUGGGAUACUCAUUGCUUUGAAUACCCUCAGUAGCAAAUUGCUGCAAUCCACCAAUAUU
>random_seq_from_cds__NO_2061 RANDOM_LENGTH 85 SEQ_LENGTH 62 GC_CONTENT 0.483870967741936 BASEPAIR 22 FREE_ENERGY -15.40 LEN_BP_RATIO 2.81818181818182
AUGAGUCUGUCCCAGAGAAUGUGCUGAGUUUCGAUGACCUUACUGCAGACGCUUUAGCUGAACCUGAAAGUCUCACAAAUCAAAA
>random_seq_from_cds__NO_2065 RANDOM_LENGTH 80 SEQ_LENGTH 71 GC_CONTENT 0.535211267605634 BASEPAIR 23 FREE_ENERGY -20.90 LEN_BP_RATIO 3.08695652173913
UGGUGUUCCUGGAACAGGCUCUCAUCCAGUAUGCCCUUCGCACCUUGGGAAGUCGGGGCUACAUUCCCAUUUAUACCCCC
>random_seq_from_cds__NO_2071 RANDOM_LENGTH 73 SEQ_LENGTH 57 GC_CONTENT 0.56140350877193 BASEPAIR 23 FREE_ENERGY -20.00 LEN_BP_RATIO 2.47826086956522
AAUAUUGUCUCAGGGUUCUUUGAAUCAUGCUGCCAGUAAGAAGCUUGACCUGGAGGCCUGGUUUCCGGGCUCA
>random_seq_from_cds__NO_2072 RANDOM_LENGTH 63 SEQ_LENGTH 54 GC_CONTENT 0.62962962962963 BASEPAIR 22 FREE_ENERGY -25.10 LEN_BP_RATIO 2.45454545454545
GGAGCCUUCCGUGAGUUGGUCUCCUGUUCUAAUUGCACGGAUUACCAGGCUCGCCGGCUUCGA
>random_seq_from_cds__NO_2082 RANDOM_LENGTH 85 SEQ_LENGTH 76 GC_CONTENT 0.684210526315789 BASEPAIR 24 FREE_ENERGY -28.60 LEN_BP_RATIO 3.16666666666667
CCUGCAAGCUGGCACAGGCCCCCGGGCUCAGGGCAGGGGAAAGGUCACCAGAAGAGUCCCUGGGUGGGCGUCGGAAAAGGAAUGU
>random_seq_from_cds__NO_2096 RANDOM_LENGTH 94 SEQ_LENGTH 81 GC_CONTENT 0.592592592592593 BASEPAIR 29 FREE_ENERGY -29.30 LEN_BP_RATIO 2.79310344827586
CAGGCUGGAUCUCUGUGGCUGCUGAACUGGACCGGGAGGAAGUUGAUUUCUACAGCUUUGGGGUAGAAGCUCGAGACCAUGGCACUCCAGCACU
>random_seq_from_cds__NO_2098 RANDOM_LENGTH 88 SEQ_LENGTH 80 GC_CONTENT 0.5875 BASEPAIR 27 FREE_ENERGY -29.10 LEN_BP_RATIO 2.96296296296296
GCGGCUCAAUGAGGAUGCAGCUGUGGGCACCAGCGUGGUGACGGUGUCAGCUGUGGACCGUGAUGCUCAUAGUGUCAUCACCUACCAG
>random_seq_from_cds__NO_2103 RANDOM_LENGTH 110 SEQ_LENGTH 84 GC_CONTENT 0.595238095238095 BASEPAIR 27 FREE_ENERGY -32.60 LEN_BP_RATIO 3.11111111111111
AUCGAUGCAGACACGGGGGCUGUCACCACCCAGGCUGAGCUGGACUAUGAAGACCAAGUGUCUUACACCCUGGCCAUUACUGCUCGGGACAAUGGCAUUCCCCAGAAGUC
>random_seq_from_cds__NO_2110 RANDOM_LENGTH 64 SEQ_LENGTH 47

GC_CONTENT 0.531914893617021 BASEPAIR 19 FREE_ENERGY -17.30 LEN_BP_RATIO 2.47368421052632
AACAUCCCUGAGGUCUUUCAGCUGGACAUCUUCUCCGGGGAGCUGACAGCCCUGGUAGACUUAG
>random_seq_from_cds__NO_2119 RANDOM_LENGTH 86 SEQ_LENGTH 75 GC_CONTENT 0.666666666666667 BASEPAIR 25 FREE_ENERGY -24.00 LEN_BP_RATIO 3
CUGCCUGCGGGAGCCCUGCGAGAACUACAUGCGCUGCGUGUCGGUGCUGCGCUUCGACUCCUCCG
CGCCCUUCAUCGCCUCCUCCU
>random_seq_from_cds__NO_2120 RANDOM_LENGTH 87 SEQ_LENGTH 83 GC_CONTENT 0.734939759036145 BASEPAIR 28 FREE_ENERGY -33.70 LEN_BP_RATIO 2.96428571428571
CCGUGCUCUUCCGGCCCAUCCACCCCGUCGGAGGGCUGCGCUGCCGCUGCCCGCCCGGCUUCACG
GGUGACUACUGCGAGACCGAGG
>random_seq_from_cds__NO_2127 RANDOM_LENGTH 85 SEQ_LENGTH 70 GC_CONTENT 0.642857142857143 BASEPAIR 26 FREE_ENERGY -31.10 LEN_BP_RATIO 2.69230769230769
ACUGUUGGGUCAGACAGGGCUCCCACAGGGCCCAUCAGAGCAGAAGGUGGCUGUGGUGACCGUGG
AUGGCUGUGACACAGGAGUG
>random_seq_from_cds__NO_2128 RANDOM_LENGTH 71 SEQ_LENGTH 63 GC_CONTENT 0.650793650793651 BASEPAIR 24 FREE_ENERGY -32.30 LEN_BP_RATIO 2.625
GCCUUGCGCUUCGGAUCUGUCCUGGGCAACUACUCCUGUGCUGCCCAGGGCACCCAGGGUGGCAG
CAAGAA
>random_seq_from_cds__NO_2160 RANDOM_LENGTH 90 SEQ_LENGTH 61 GC_CONTENT 0.655737704918033 BASEPAIR 20 FREE_ENERGY -27.00 LEN_BP_RATIO 3.05
UGCCCUGGGCCUGGCUCAGCUGGUCUUCCUCCUGGGAAUCAACCAGGCUGACCUCCCUGUUUGCC
UGCACAGUCAUUGCCAUCCUGCUGC
>random_seq_from_cds__NO_2166 RANDOM_LENGTH 73 SEQ_LENGTH 51 GC_CONTENT 0.627450980392157 BASEPAIR 19 FREE_ENERGY -18.90 LEN_BP_RATIO 2.68421052631579
UCCUAUGUGGUGCUUAGCAAGGAGGUCCGGAAAGCACUCAAGCUUGCCUGCAGCCGCAAGCCCAG
CCCUGACC
>random_seq_from_cds__NO_2173 RANDOM_LENGTH 73 SEQ_LENGTH 63 GC_CONTENT 0.666666666666667 BASEPAIR 22 FREE_ENERGY -26.10 LEN_BP_RATIO 2.86363636363636
CCCCUGAGGAGCGGCUGCGGGAGAAUGGAGAUGCCCUGUCUCGAGAGGGGUCCCUAGGCCCCCUU
CCAGGCUC
>random_seq_from_cds__NO_2186 RANDOM_LENGTH 68 SEQ_LENGTH 48 GC_CONTENT 0.520833333333333 BASEPAIR 19 FREE_ENERGY -24.60 LEN_BP_RATIO 2.52631578947368
GCUAGGCCAGUGAAAUAGAUGGGGUCCCUGGUGAACGUGUAGGUUUGCCAGCAUUGACCUUCGUC
UGU
>random_seq_from_cds__NO_2198 RANDOM_LENGTH 77 SEQ_LENGTH 62

GC_CONTENT 0.387096774193548 BASEPAIR 22 FREE_ENERGY -15.10 LEN_BP_RATIO 2.81818181818182
GAGGGCUAUACAUCAUCUGAGUGAGAGGAUGAAAAGGGAGAUCUGUUUGCACAAAAUUCUUCGCAAAAUCUGAUGAU
>random_seq_from_cds__NO_2200 RANDOM_LENGTH 102 SEQ_LENGTH 81 GC_CONTENT 0.382716049382716 BASEPAIR 27 FREE_ENERGY -19.60 LEN_BP_RATIO 3
GCCAAAUUCAGUCCGAAUAAAGGUGUUAUUGAUGAGAUCUGUAAUAUCCUUAAAGUUCUUCCCAUAAUCCUCACCUUCGAUAUAGCUUGGACUGUCCAAAAG
>random_seq_from_cds__NO_2211 RANDOM_LENGTH 79 SEQ_LENGTH 52 GC_CONTENT 0.557692307692308 BASEPAIR 19 FREE_ENERGY -17.75 LEN_BP_RATIO 2.73684210526316
AAUUAGCCCACUCAUGGCACAACCCUAUGUGAGCAUCAAUCUCUACAAUUUUCUCAAUGCUGCUGGGCUCCAUCAGUGG
>random_seq_from_cds__NO_2218 RANDOM_LENGTH 97 SEQ_LENGTH 75 GC_CONTENT 0.52 BASEPAIR 26 FREE_ENERGY -20.30 LEN_BP_RATIO 2.88461538461538
GACUCAUGCAUACGAUUCCACUUCGUCAUCUUCCACCAGCACCAUAUCAUAGGCACUGAGGGCUGCACAGAAAAUGAUGCAGGUGACUCCCUCGAAG
>random_seq_from_cds__NO_2221 RANDOM_LENGTH 102 SEQ_LENGTH 68 GC_CONTENT 0.544117647058823 BASEPAIR 23 FREE_ENERGY -21.80 LEN_BP_RATIO 2.95652173913043
UAAUUCGUUCUAAUUGGUUCAGGUAGCUAAGAUGCGGAGUCAUUAAGCUGGUAUUCUGCAGCUCUCUCGAAGCAGGCUUGCACCCCACCAUCCUUCCACAAC
>random_seq_from_cds__NO_2234 RANDOM_LENGTH 85 SEQ_LENGTH 74 GC_CONTENT 0.621621621621622 BASEPAIR 26 FREE_ENERGY -29.30 LEN_BP_RATIO 2.84615384615385
UCACAAAGAUGGCUGUCUGGGGCAACAAGUAGCAUGCCCAUGACUCUGGGGUACUGGGACAUCCGUGGGGCUGGCCCACGCCAUC
>random_seq_from_cds__NO_2239 RANDOM_LENGTH 68 SEQ_LENGTH 53 GC_CONTENT 0.509433962264151 BASEPAIR 20 FREE_ENERGY -17.50 LEN_BP_RATIO 2.65
UGGGGAAGCGGCCAUGGUUUGCAGGAGACAAGGAUCACCUUUGUGGAUUUCCUUGCCUAUGAUGUCCU
>random_seq_from_cds__NO_2247 RANDOM_LENGTH 88 SEQ_LENGTH 75 GC_CONTENT 0.52 BASEPAIR 25 FREE_ENERGY -20.50 LEN_BP_RATIO 3
UCAUUUGCUGCAGUGUUGUGGUAUAAAUGGCACGAGUGAUUGGACCAGUGGCCCACCAGCAUCUUGCCCCUCAGAUCGAAAAGUGGAG
>random_seq_from_cds__NO_2263 RANDOM_LENGTH 110 SEQ_LENGTH 84 GC_CONTENT 0.476190476190476 BASEPAIR 28 FREE_ENERGY -19.53 LEN_BP_RATIO 3
UGAAGAACGGCCAAGGUUUUGCACUAGUAUAUUCUAUUACAGCUCAGUCCACGUUUAACGACUUACAGGACCUGAGGGAACAGAUUUUACGGGUUAAGGACACGGAAGAU

>random_seq_from_cds__NO_2276 RANDOM_LENGTH 110 SEQ_LENGTH 87 GC_CONTENT 0.540229885057471 BASEPAIR 28 FREE_ENERGY -29.80 LEN_BP_RATIO 3.10714285714286
UGGAACGGAACACAUCACUAGAUCCUGGGAACCUGGAUCAUCCAUGUCAUGUUGGGGCAGUGGAU
GCGAUUGCAGACGAACCAGGAGGAGAGUUUGCUCCACUCAUCAGG
>random_seq_from_cds__NO_2277 RANDOM_LENGTH 90 SEQ_LENGTH 76 GC_CONTENT 0.605263157894737 BASEPAIR 29 FREE_ENERGY -28.50 LEN_BP_RATIO 2.62068965517241
ACUGCGGCCAUAGAUGGACAGGCGGGGCUCAGCAUGCUGGUACUUGGCCUCCACCAGGUCCGCAC
CUACCUCCCUUGAUGAUAGUGGCAA
>random_seq_from_cds__NO_2285 RANDOM_LENGTH 77 SEQ_LENGTH 67 GC_CONTENT 0.522388059701492 BASEPAIR 25 FREE_ENERGY -23.50 LEN_BP_RATIO 2.68
AGGAAGGUUGUCUGUUCGGAAGGGGUCCUCUCCCUUCUUCACAGGAGGAGUAAAGACCUGGAUAG
AAGCUCUCAUUU
>random_seq_from_cds__NO_2288 RANDOM_LENGTH 85 SEQ_LENGTH 78 GC_CONTENT 0.371794871794872 BASEPAIR 27 FREE_ENERGY -17.10 LEN_BP_RATIO 2.88888888888889
UGGAGAUGAGGAAAUGUAUUCAUCAAUGUGGGACAGUUUGGUGGAAGAUGUUUCACUUAGUGGAA
UGGACAAAUUAACAGUCUUC
>random_seq_from_cds__NO_2294 RANDOM_LENGTH 95 SEQ_LENGTH 75 GC_CONTENT 0.466666666666667 BASEPAIR 26 FREE_ENERGY -18.50 LEN_BP_RATIO 2.88461538461538
UAACUCUUGGCCAGUUCGUGGGCUUGUUUUGUAUCAACUGUCCUUGUUGGCAAAUCACACUUGUU
UCCCACUAGCACCAUAGGUACAUCAUCCGA
>random_seq_from_cds__NO_2334 RANDOM_LENGTH 75 SEQ_LENGTH 58 GC_CONTENT 0.5 BASEPAIR 19 FREE_ENERGY -15.40 LEN_BP_RATIO 3.05263157894737
UUGCACAUGAUGACAGGUGUGAUGUACCUACAGUUUGUACCCAAGUCUCACCUCUUCUUCACUGC
CGGAAAAGAU
>random_seq_from_cds__NO_2339 RANDOM_LENGTH 85 SEQ_LENGTH 66 GC_CONTENT 0.5 BASEPAIR 21 FREE_ENERGY -19.77 LEN_BP_RATIO 3.14285714285714
AAAAUGAAGGAACACAAAGCCAUUUGUAAAGCUGCAGGGAAAGAGGGUUCCACUUCCCAGCAACC
CCAUCCUAAUGGCUUAUGGC
>random_seq_from_cds__NO_2360 RANDOM_LENGTH 87 SEQ_LENGTH 66 GC_CONTENT 0.621212121212121 BASEPAIR 25 FREE_ENERGY -31.80 LEN_BP_RATIO 2.64
ACUUGCCCACAGAGGCUGGGAAAGGAUGAUAGGCUGGGAGAAUGGUCUGAGCUACCUGCCCGUCC
UGCUGGGGCAUCAUGGCAGUGG
>random_seq_from_cds__NO_2366 RANDOM_LENGTH 87 SEQ_LENGTH 75 GC_CONTENT 0.52 BASEPAIR 23 FREE_ENERGY -18.40 LEN_BP_RATIO 3.26086956521739
ACAUAGGGUUGGGUGAGGCCUGUAAGAUCCCAGGGGAUGUAAUCAUUGGAGAGGAUGUGGUGUCG

GAAACAUACGUGUGAGGAGAUU
>random_seq_from_cds__NO_2385 RANDOM_LENGTH 110 SEQ_LENGTH 90 GC_CONTENT 0.511111111111111 BASEPAIR 30 FREE_ENERGY -29.12 LEN_BP_RATIO 3
UGUUCUUGUUCACCAGGAAGGGAUCUGCGUGUCAUCCUCUGUUUCUUCAUAGCAGCUGACUUCUCACCAUAAUAGGGGUACACCAUGAGUUCCCCCUGGGAGUCCCGCUU
>random_seq_from_cds__NO_2392 RANDOM_LENGTH 81 SEQ_LENGTH 72 GC_CONTENT 0.708333333333333 BASEPAIR 27 FREE_ENERGY -30.40 LEN_BP_RATIO 2.66666666666667
CUGGCAGGGGCUACUGGCACAGCCUGACUCGCAGUCCCGGGGACUGGGGCAGAAGCAGCGGGGUCCAGAGGCGGUGUGCAC
>random_seq_from_cds__NO_2396 RANDOM_LENGTH 98 SEQ_LENGTH 83 GC_CONTENT 0.566265060240964 BASEPAIR 29 FREE_ENERGY -28.60 LEN_BP_RATIO 2.86206896551724
AGGGGUUGGAGAGGCACUCGUUGAUGUCUCCCUCACAACGCUCCCCAGCAAAGCCAGGCAAGCAGCGACAACUGUAGCCUCCAAUCCUAUCCAUGCAC
>random_seq_from_cds__NO_2403 RANDOM_LENGTH 84 SEQ_LENGTH 60 GC_CONTENT 0.633333333333333 BASEPAIR 21 FREE_ENERGY -23.20 LEN_BP_RATIO 2.85714285714286
UUCACCAGGGUCCUGACAGUUUUUCCCAGUGUAGCCCAGGGGGCAGCUGCAGCGGUAGGUACCCAGGCCAUCAACACACGUUCC
>random_seq_from_cds__NO_2422 RANDOM_LENGTH 94 SEQ_LENGTH 80 GC_CONTENT 0.5125 BASEPAIR 28 FREE_ENERGY -25.30 LEN_BP_RATIO 2.85714285714286
AUGCACACCUUUGAAACCCUGGCAUGCACAGACAUGUGAAGCCUCCAAUCUUAUCCAGACAGGUAGCAUCAUUCUGGCAGGGGUCUGAAUGGCA
>random_seq_from_cds__NO_2423 RANDOM_LENGTH 81 SEQ_LENGTH 61 GC_CONTENT 0.557377049180328 BASEPAIR 22 FREE_ENERGY -15.40 LEN_BP_RATIO 2.77272727272727
CUCAUUGAUGUCCAUCUCACAACGAGGUCCUGCAUAACCCUUCAGACACUCACAGUGGAAGGCGCCAUCCGUGUUCACACA
>random_seq_from_cds__NO_2424 RANDOM_LENGTH 97 SEQ_LENGTH 74 GC_CONTENT 0.567567567567568 BASEPAIR 26 FREE_ENERGY -34.26 LEN_BP_RATIO 2.84615384615385
UUUUCCUGCAUGCUCACAAGGAUUGCUAUUGGCCCAUGGCACAUUCAUCCACAUCUUCUGUGCAGUCAGCCCCUUUGUAGCCUUGUGGGCAGGUGCA
>random_seq_from_cds__NO_2443 RANDOM_LENGTH 101 SEQ_LENGTH 83 GC_CONTENT 0.55421686746988 BASEPAIR 25 FREE_ENERGY -31.64 LEN_BP_RATIO 3.32
AGGCUGCUAUGGAGGGACUCAAUGGCCAGGAUUUGAUGGGACAGCCCAUCAGCGUUGACUGGUGUUUUGUUCGGGGUCCACCAAAAGGCAAGAGGAGGAGG
>random_seq_from_cds__NO_2453 RANDOM_LENGTH 87 SEQ_LENGTH 64 GC_CONTENT 0.421875 BASEPAIR 20 FREE_ENERGY -15.09 LEN_BP_RATIO 3.2

CCUUCACAUUUCCUUUCACUUUCACCAAGCCAGAAAUGAUACGAUUUGGCAGGUCAUCAUUUAAG
GUUGGAUGCUGACUCAGAGCUC
>random_seq_from_cds__NO_2465 RANDOM_LENGTH 96 SEQ_LENGTH 83
GC_CONTENT 0.662650602409639 BASEPAIR 31 FREE_ENERGY
-38.40 LEN_BP_RATIO 2.67741935483871
CCAGCCAAUCCCAGCCAUCCCCCUGUGACUCCAAGAGUGGGGGCCAUACCCCUAAAGCACUCCCU
GGCCCAGGUGGGAGCAUGGGGCUGAAGAAUG
>random_seq_from_cds__NO_2489 RANDOM_LENGTH 97 SEQ_LENGTH 80
GC_CONTENT 0.6375 BASEPAIR 30 FREE_ENERGY -42.10
LEN_BP_RATIO 2.66666666666667
UACGCCCAGGUGGCUCAGACAUGCUGCCUGCUCAGCAGAAGAUGGUGCCACUGCCAUUUGGUGAG
CACCCCCAGCAGGAGUAUGGCAUGGGCCCCAG
>random_seq_from_cds__NO_2493 RANDOM_LENGTH 86 SEQ_LENGTH 55
GC_CONTENT 0.6 BASEPAIR 22 FREE_ENERGY -23.10 LEN_BP_RATIO
2.5
AACCUCAAGUCCCCCCAGACUCCAUCGCAGCUGGCAGGCAUGCUGGCGGGCCCAGCUGCUGCUGC
UUCCAUUAAGUCCCCCCCUGU
>random_seq_from_cds__NO_2518 RANDOM_LENGTH 84 SEQ_LENGTH 72
GC_CONTENT 0.638888888888889 BASEPAIR 23 FREE_ENERGY
-32.62 LEN_BP_RATIO 3.1304347826087
GGAGGUGGCACUGGGGGUGGGAAGGAGCCAGGAGGAGGCAUGCCCUGGUGGAGGAAGCCCAGACC
CCAAUGAUGAUACCACAGG
>random_seq_from_cds__NO_2547 RANDOM_LENGTH 70 SEQ_LENGTH 64
GC_CONTENT 0.5625 BASEPAIR 24 FREE_ENERGY -26.20
LEN_BP_RATIO 2.66666666666667
GGUCAACACUGGACGUAUGAGGGGCCCACAUGGUCAGGACCAUUGGCCAGCCUCUUACCCUGAGU
GUGGA
>random_seq_from_cds__NO_2554 RANDOM_LENGTH 85 SEQ_LENGTH 67
GC_CONTENT 0.462686567164179 BASEPAIR 27 FREE_ENERGY
-24.20 LEN_BP_RATIO 2.48148148148148
AGUGUGCUCUGGACAGUUUUUUAUAGAAGGUCCCAGAUUUCAAUGGAACAGGCUGGAAAAGCUUC
AGGGGACAUUGUUCUCCACA
>random_seq_from_cds__NO_2556 RANDOM_LENGTH 67 SEQ_LENGTH 61
GC_CONTENT 0.540983606557377 BASEPAIR 20 FREE_ENERGY
-15.60 LEN_BP_RATIO 3.05
GGCAGGAUCCUCGUAUACCACAGGGUGAAAUGCUGAGUCUAGGUGUAGGAAUCUUGGUUGGCUGU
CU
>random_seq_from_cds__NO_2562 RANDOM_LENGTH 71 SEQ_LENGTH 58
GC_CONTENT 0.482758620689655 BASEPAIR 21 FREE_ENERGY
-20.50 LEN_BP_RATIO 2.76190476190476
UCCCAUGGCUUUGUAGGGAUAGGAAGCGUCUGAGUCGAUGCCCUUGUUAUCAAUGAUGUACUGGA
AAGCCG
>random_seq_from_cds__NO_2592 RANDOM_LENGTH 98 SEQ_LENGTH 78
GC_CONTENT 0.461538461538462 BASEPAIR 26 FREE_ENERGY

-23.50 LEN_BP_RATIO 3
GUGUUGCUUUUGGGUCUUUAUGCUCCUGGGAUAAGGAACUUUGAAGCACAUGCUCCUUUAAGUCU
UUAUUACCCUGUGGUGCAGUGUCUACCUCUCCC
>random_seq_from_cds__NO_2625 RANDOM_LENGTH 106 SEQ_LENGTH 97
GC_CONTENT 0.628865979381443 BASEPAIR 33 FREE_ENERGY
-37.00 LEN_BP_RATIO 2.93939393939394
GGAUGUCCUUGGGCUCAAUGACCCUUGUGCAGCUUUGGGGCCCGGGGCUCAGAGCCCACGUCCAC
CACAUAGAUGCGAGAGGAGAUGAGACUGGGCAGCACCAGCU
>random_seq_from_cds__NO_2629 RANDOM_LENGTH 78 SEQ_LENGTH 68
GC_CONTENT 0.647058823529412 BASEPAIR 24 FREE_ENERGY
-26.40 LEN_BP_RATIO 2.83333333333333
AAGCGUUUUUGGGGUCGCGGUCCGGACUUUGGGCGGGGGGUCCGGCCCCAGGACAGUUUUACCGC
AUUCCGUCCACUC
>random_seq_from_cds__NO_2630 RANDOM_LENGTH 78 SEQ_LENGTH 74
GC_CONTENT 0.608108108108108 BASEPAIR 27 FREE_ENERGY
-24.40 LEN_BP_RATIO 2.74074074074074
CCGAUUCCUUCAUGGAUCCGGCGUCUGCACUUUACAGAGGUCCAAUCACGCGGACCCAGGAACCC
CAUGGUGACCGGG
>random_seq_from_cds__NO_2633 RANDOM_LENGTH 88 SEQ_LENGTH 73
GC_CONTENT 0.493150684931507 BASEPAIR 27 FREE_ENERGY
-29.42 LEN_BP_RATIO 2.7037037037037
UUCUCGGCCAGAUGGUGGAUUGAUGAGGAGCUUCUGGGAGAUGGACACAGCUAUAGUCCUAGAGC
UAUUCAUUCAUGGCUGACCAGGG
>random_seq_from_cds__NO_2636 RANDOM_LENGTH 97 SEQ_LENGTH 68
GC_CONTENT 0.573529411764706 BASEPAIR 24 FREE_ENERGY
-25.50 LEN_BP_RATIO 2.83333333333333
CAGUGCUAAGCCAGACCGAGGCCCGCGACUUAGUAGAACGCUGCAUGCGAGUGCUGUACUACCGA
GAUGCCCGUUCUUACAACCGGGUUUCAAAUCG
>random_seq_from_cds__NO_2655 RANDOM_LENGTH 100 SEQ_LENGTH 85
GC_CONTENT 0.517647058823529 BASEPAIR 31 FREE_ENERGY
-27.91 LEN_BP_RATIO 2.74193548387097
UGAGGACACUCGGUCUCUAGCAAUUUCUUCAGGUCAUCCCUGUAGACGGCAUGGAAAUUCCCCUU
UAUCAGGGAGUACUUGUGGUAGACGUCGAUGAUAG
>random_seq_from_cds__NO_2674 RANDOM_LENGTH 84 SEQ_LENGTH 78
GC_CONTENT 0.474358974358974 BASEPAIR 25 FREE_ENERGY
-19.29 LEN_BP_RATIO 3.12
AGUUUGAGUAUCGGACAUGUCAUGCUGCCCAAGGACAUAGCCAAGCUGGUCCCUAAAACCCAUCU
GAUGUCUGAAUCUGAAUGG
>random_seq_from_cds__NO_2675 RANDOM_LENGTH 95 SEQ_LENGTH 85
GC_CONTENT 0.576470588235294 BASEPAIR 27 FREE_ENERGY
-26.90 LEN_BP_RATIO 3.14814814814815
AGGAAUCUUGGCGUUCAGCAGAGUCAGGGAUGGGUCCAUUAUAUGAUCCAUGAACCAGGAACCUC
ACAUCUUGCUGUUCCGGCGCCCACUACCCA
>random_seq_from_cds__NO_2676 RANDOM_LENGTH 85 SEQ_LENGTH 69

GC_CONTENT 0.579710144927536 BASEPAIR 25 FREE_ENERGY -24.00 LEN_BP_RATIO 2.76
AGAAACCAAAGAAAUGACUUAGCUCUCGGAGGCUACAGCCUCCCCGUCGCGGCUCUCCGUCUCCUCCGGGAUGUCCUGCAUUCUG
>random_seq_from_cds__NO_2686 RANDOM_LENGTH 86 SEQ_LENGTH 79 GC_CONTENT 0.518987341772152 BASEPAIR 26 FREE_ENERGY -23.41 LEN_BP_RATIO 3.03846153846154
CCUCCCUCUUGCGGUGAUCUCAGCUGAUUUCCAUUUCGAUCCCCUCUGGCUAGAGUCUGAGUCAGCUCUGCAGAGUUCAAUGGAGC
>random_seq_from_cds__NO_2689 RANDOM_LENGTH 68 SEQ_LENGTH 63 GC_CONTENT 0.682539682539683 BASEPAIR 20 FREE_ENERGY -29.30 LEN_BP_RATIO 3.15
UCCUGCAGCAGCCGCUCGCCACGAGGGGACUCAAGGGACUCAGAGCGGAAAAGGCCCCUGGGCAGGGU
>random_seq_from_cds__NO_2698 RANDOM_LENGTH 82 SEQ_LENGTH 68 GC_CONTENT 0.661764705882353 BASEPAIR 24 FREE_ENERGY -34.00 LEN_BP_RATIO 2.83333333333333
ACCAGCAGGAUGCACUCCUGUACCCCGUGCCGCUUGAGCACGGCGGGGCGGGUCACUUUCCCGGAUGAAUUGCUGGAAGCGU
>random_seq_from_cds__NO_2703 RANDOM_LENGTH 87 SEQ_LENGTH 77 GC_CONTENT 0.480519480519481 BASEPAIR 22 FREE_ENERGY -18.19 LEN_BP_RATIO 3.5
AUCUUCAGUGUCCUCACAUGGUGCAGCUCUGUCUGGAUUAGCUCCAUAGAUGACAUCUUGCUGCUUCAUCACCUCCUUUUUAUGCUG
>random_seq_from_cds__NO_2706 RANDOM_LENGTH 70 SEQ_LENGTH 54 GC_CONTENT 0.462962962962963 BASEPAIR 19 FREE_ENERGY -16.60 LEN_BP_RATIO 2.84210526315789
GCGCAGCCCCAGGGGAGACUCAUCAUUGAAAUGUCCCAGCAAUGUUGGUGGUAGAAACACUCUUGGCUAA
>random_seq_from_cds__NO_2721 RANDOM_LENGTH 110 SEQ_LENGTH 79 GC_CONTENT 0.379746835443038 BASEPAIR 25 FREE_ENERGY -19.60 LEN_BP_RATIO 3.16
UGAUUUUCCAGUUGCCAGAGAAGUUGGGCAUAAUGGUGAAUGAAUACAAGAAAAUUCUUUUGCUGAAAGGAUUUGAGCUCAUGGAUGAUUAUCAUUUUACAUCAAUUAAG
>random_seq_from_cds__NO_2726 RANDOM_LENGTH 89 SEQ_LENGTH 75 GC_CONTENT 0.586666666666667 BASEPAIR 24 FREE_ENERGY -20.10 LEN_BP_RATIO 3.125
CAAAAGGAAUAAGGUGUCCCAAGAGCAGAGUAAGCCCCCAGGUCCCUCAGGAGCCAGCACAUCUGCAGCUGUGGAUCAUCCCCCACUAC
>random_seq_from_cds__NO_2742 RANDOM_LENGTH 72 SEQ_LENGTH 61 GC_CONTENT 0.508196721311475 BASEPAIR 23 FREE_ENERGY -21.90 LEN_BP_RATIO 2.65217391304348
UUCUUGGGUCUCAAACGUGAAGGGCUUCUUUGCUUUCAGUACCAUAACUGGCAAACAGCGCUUCUGAAACCC

>random_seq_from_cds__NO_2743 RANDOM_LENGTH 89 SEQ_LENGTH 75 GC_CONTENT 0.506666666666667 BASEPAIR 25 FREE_ENERGY -25.10 LEN_BP_RATIO 3
UUCUCUGAUAGAUUCCUGCUGGGCCACCAUCUGUUUCUGUUCAGGCCUUAACAUGAGGAGAGACU
UUUGGUGCAGCACGUUGCUUUGCG
>random_seq_from_cds__NO_2756 RANDOM_LENGTH 90 SEQ_LENGTH 75 GC_CONTENT 0.64 BASEPAIR 26 FREE_ENERGY -30.50 LEN_BP_RATIO 2.88461538461538
ACCCUUGGUGUGGGUCUGAAUCAGAGGAUCUGGCGUGGCAUCCCGUAGCCAGUCAUGCCUGCCUG
AGACGCCCCGCGGUUGGUGCCCAUC
>random_seq_from_cds__NO_2757 RANDOM_LENGTH 110 SEQ_LENGTH 87 GC_CONTENT 0.540229885057471 BASEPAIR 34 FREE_ENERGY -36.10 LEN_BP_RATIO 2.55882352941176
UGUAACCCGAUCACGUUCUUGCCCUCUUGCAGCUGGUUAUCCGAGAAGUUCCGAGGAUUCUCCUU
GGAUUUCCUUAGGGAACCAGUUGGGAUCCCCAGAGAAGAGCCCAU
>random_seq_from_cds__NO_2766 RANDOM_LENGTH 75 SEQ_LENGTH 63 GC_CONTENT 0.53968253968254 BASEPAIR 24 FREE_ENERGY -25.90 LEN_BP_RATIO 2.625
AGCUGAUCCUGGAGGUUAGCAGCCCAAGUCCUAGAAGACAAGGGUGUUGGCUUCGGGCUGGUAGA
CUCUGAGAAG
>random_seq_from_cds__NO_2795 RANDOM_LENGTH 109 SEQ_LENGTH 64 GC_CONTENT 0.46875 BASEPAIR 24 FREE_ENERGY -24.40 LEN_BP_RATIO 2.66666666666667
CUUCUUCCUCCUCUUCCUCUUCCUCUGAGCGGUCAUGGACUCGAUUUUCAUCAUUAAUGGAGUAA
UGACCAGUGUCCUCCAUGCUGUCAGAGUCCUUAUCUUCACCUGA
>random_seq_from_cds__NO_2796 RANDOM_LENGTH 86 SEQ_LENGTH 60 GC_CONTENT 0.6 BASEPAIR 20 FREE_ENERGY -22.90 LEN_BP_RATIO 3
GCUCUCUGUGUCUGUGCCUCGACUUUCUGUGCUGGUGCGGUUGGGGCCACCAUCAUCCCCAGUCA
AGCUCAAACUCAGGUCUGAGG
>random_seq_from_cds__NO_2801 RANDOM_LENGTH 87 SEQ_LENGTH 74 GC_CONTENT 0.608108108108108 BASEPAIR 28 FREE_ENERGY -34.90 LEN_BP_RATIO 2.64285714285714
GAUUUUUCGGGUCCUGUUGGGCCACCUCAGGCUUGGGCCCGAGUUCUAGUAGGCGCCGAGCAAAG
GUGGCAGCUGUCUUGAAGUUCU
>random_seq_from_cds__NO_2802 RANDOM_LENGTH 72 SEQ_LENGTH 56 GC_CONTENT 0.535714285714286 BASEPAIR 21 FREE_ENERGY -22.00 LEN_BP_RATIO 2.66666666666667
UGAGCUUGAAGAACAGAUUGAGGGCUGUACGCAGCACCAGGAUCAUGUGCACAGGCUGCAGGUUU
GAGUGGG
>random_seq_from_cds__NO_2809 RANDOM_LENGTH 87 SEQ_LENGTH 67 GC_CONTENT 0.582089552238806 BASEPAIR 26 FREE_ENERGY -29.00 LEN_BP_RATIO 2.57692307692308
CAGAUCCUGAGUUGGACUUGUUCCCUUGGUUGGGGGCACAAAGAAACCAUCUUCAGCCCCACCAG

CUGCCCCAGGGGAUAUAUCCCA
>random_seq_from_cds__NO_2813 RANDOM_LENGTH 77 SEQ_LENGTH 70 GC_CONTENT 0.5 BASEPAIR 24 FREE_ENERGY -16.50 LEN_BP_RATIO 2.91666666666667
AGCAGCUUGGCAUUAGGGUCAAUGUCUGGGAUCUGUCUCCUUCUCUGGGUCAAAUGUCUCCUUUAGGCUCUCAGCUU
>random_seq_from_cds__NO_2814 RANDOM_LENGTH 89 SEQ_LENGTH 68 GC_CONTENT 0.529411764705882 BASEPAIR 25 FREE_ENERGY -19.60 LEN_BP_RATIO 2.72
CUUCAUCUAAGCCAUGGGUAGCAGCUGUGAGAUAGGCCAGGGACUCUCUGUCCACAGUUCUUCAGGAUCCGCACACGCUCUGACACAUC
>random_seq_from_cds__NO_2822 RANDOM_LENGTH 81 SEQ_LENGTH 69 GC_CONTENT 0.463768115942029 BASEPAIR 20 FREE_ENERGY -15.27 LEN_BP_RATIO 3.45
GACAGCAUAUUUGAUGUGGUUGCUUGUGGUAUAGAUAAAUACCCCACUCUCAUCCCAGGCCCCACUCUUGACACGAAUGUU
>random_seq_from_cds__NO_2823 RANDOM_LENGTH 87 SEQ_LENGTH 76 GC_CONTENT 0.447368421052632 BASEPAIR 29 FREE_ENERGY -24.00 LEN_BP_RATIO 2.62068965517241
CUCAUGAAUGUUACAUAAAGCAUCCAGUUUGCGGUUACAGAUCACAAUGGCCGUGUUUGGCUAGUAGUGCUACAUGUGACAUGUCUG
>random_seq_from_cds__NO_2830 RANDOM_LENGTH 86 SEQ_LENGTH 77 GC_CONTENT 0.506493506493506 BASEPAIR 22 FREE_ENERGY -21.95 LEN_BP_RATIO 3.5
UAGCAUAUUGCCAUGAACAGCAUAGGCUGGCCGUUCCCGUUCCAGCUUAAACACAAUCAUACCACCAUCAUGGCCCUGCUGCAAAG
>random_seq_from_cds__NO_2834 RANDOM_LENGTH 70 SEQ_LENGTH 49 GC_CONTENT 0.591836734693878 BASEPAIR 20 FREE_ENERGY -24.00 LEN_BP_RATIO 2.45
AGGGGCAUAGUGGGGUGGAAGGCAGCCCAGUUUACUCCACGAUCGUGACCCCUCUAGUACAUGCUUCACC
>random_seq_from_cds__NO_2842 RANDOM_LENGTH 80 SEQ_LENGTH 72 GC_CONTENT 0.458333333333333 BASEPAIR 23 FREE_ENERGY -15.50 LEN_BP_RATIO 3.1304347826087
CUUCUGAGUUUUCCUUGGCUGCUGGCUUCUCAUCUUCUCCCAUGAGACGAAUGUUGUGCUUUUCCCGGAAUUCAUUUAGU
>random_seq_from_cds__NO_2845 RANDOM_LENGTH 97 SEQ_LENGTH 86 GC_CONTENT 0.546511627906977 BASEPAIR 33 FREE_ENERGY -26.00 LEN_BP_RATIO 2.60606060606061
UCCUUCAGUGUAUCGAUCACUAGGCCUGUGCUCAUUCAACUCCAUCUCCAACUCAGCUGCUUUGGAUGCCAGGCCUCGCUGUUCCUGCCGAAGGCGG
>random_seq_from_cds__NO_2854 RANDOM_LENGTH 81 SEQ_LENGTH 63 GC_CONTENT 0.603174603174603 BASEPAIR 20 FREE_ENERGY -19.70 LEN_BP_RATIO 3.15

CAUGGCGGGGUCCCCGGGGGUCACACACAUACAGAUUGUGGUCAUUUUCUGGCAAGAGGUCCACA
UCGUGCAAGAACAGGC
>random_seq_from_cds__NO_2856 RANDOM_LENGTH 110 SEQ_LENGTH 91
GC_CONTENT 0.659340659340659 BASEPAIR 28 FREE_ENERGY
-38.00 LEN_BP_RATIO 3.25
UGUUCCAUUUCCAGCCCUGGUGGAUGACAUAGAUGCCAUAAGCAAGCUGCUGGCGCUGCAAGAAG
GGGUGCAGGUGGUAGAGCAGCAGGCGCAGGUGGUGCUCCCGGGCA
>random_seq_from_cds__NO_2862 RANDOM_LENGTH 82 SEQ_LENGTH 59
GC_CONTENT 0.576271186440678 BASEPAIR 20 FREE_ENERGY
-22.01 LEN_BP_RATIO 2.95
UUGGUUGAACAAGCAGGCGGCCCUGGGAGAGCCUCAGCUCUGCUAUAUCCUGGAUGCCAUCCUGU
UUCUGUAUGGAAUUGUC
>random_seq_from_cds__NO_2864 RANDOM_LENGTH 81 SEQ_LENGTH 60
GC_CONTENT 0.533333333333333 BASEPAIR 22 FREE_ENERGY
-21.20 LEN_BP_RATIO 2.72727272727273
GACUUACGAGACUCUGAAGCAUGAGAAACCACCACAGUAGUUCACUGGGUGGCAGGCUGUGUUCC
AAGUUCCACGAAAUAG
>random_seq_from_cds__NO_2873 RANDOM_LENGTH 68 SEQ_LENGTH 63
GC_CONTENT 0.634920634920635 BASEPAIR 24 FREE_ENERGY
-28.50 LEN_BP_RATIO 2.625
GUGUUGUCCUCUAUGGGGCUGGCAGCCAUGUGACAUGUUGGAGGCUCCGGGCCCGAGUGAUGGCU
GCG
>random_seq_from_cds__NO_2887 RANDOM_LENGTH 84 SEQ_LENGTH 62
GC_CONTENT 0.580645161290323 BASEPAIR 20 FREE_ENERGY
-22.24 LEN_BP_RATIO 3.1
CAGCUGCCAAAUAUGGAAGGGCUGGACUCAGAAGAAGGGGAUGGAGCCUGGUGCCCUGAGAUUCC
AGUGGAACCUGAUGACCUG
>random_seq_from_cds__NO_2902 RANDOM_LENGTH 80 SEQ_LENGTH 66
GC_CONTENT 0.575757575757576 BASEPAIR 24 FREE_ENERGY
-22.80 LEN_BP_RATIO 2.75
GCUGACAUAGUGAACCUCCAAGGAGUGACAGGAGGCAACACAUACUCAGUGCCUGCCGUCACCAU
GGACCUGCUCUCAGG
>random_seq_from_cds__NO_2903 RANDOM_LENGTH 82 SEQ_LENGTH 51
GC_CONTENT 0.509803921568627 BASEPAIR 19 FREE_ENERGY
-20.80 LEN_BP_RATIO 2.68421052631579
AAAAGAUGUGGCUGUGGAGGAGUUCCCCAGGAAACUCCUAACUUUCAAAGAGAAGCUGGGAGAAG
GACAGUUUGGGGAGGGU
>random_seq_from_cds__NO_2914 RANDOM_LENGTH 98 SEQ_LENGTH 79
GC_CONTENT 0.468354430379747 BASEPAIR 28 FREE_ENERGY
-29.70 LEN_BP_RATIO 2.82142857142857
AUAUGGUUUUGUGCUUCUAACUGGUGCUGCCAGCUUUAUAAUGGUGGCCCACCUAGCCAUCAAUG
UUUCCAAGGCCCGCAAGAAGUACAAAGUGGAGG
>random_seq_from_cds__NO_2921 RANDOM_LENGTH 89 SEQ_LENGTH 66
GC_CONTENT 0.515151515151515 BASEPAIR 24 FREE_ENERGY

-20.10 LEN_BP_RATIO 2.75
CCCGUUGGAUGUCCCCUGGUAAAGACGCCAGCUGCUAGUCCAAAAGUGGUAUCAUUGGCUCUUUC
UAGAACCUCAGCUUCAGUGUCAAA
>random_seq_from_cds__NO_2938 RANDOM_LENGTH 82 SEQ_LENGTH 56
GC_CONTENT 0.607142857142857 BASEPAIR 20 FREE_ENERGY
-18.90 LEN_BP_RATIO 2.8
UACCGUGUCCUUACCUCGGAGCAGAAGGCCAAAGCCCUGAAGGGCCAGUUCAACUUUGACCACCC
GGGAUGCCUUUGACAAU
>random_seq_from_cds__NO_2974 RANDOM_LENGTH 83 SEQ_LENGTH 73
GC_CONTENT 0.493150684931507 BASEPAIR 26 FREE_ENERGY
-22.90 LEN_BP_RATIO 2.80769230769231
ACUGUGAAGUGAUGAUAGGGAAUGAAGUCCCUGCCUCGGCAGUGGUCACAUCUGUGGGCAUCUUC
UUCUAAAAAGCGGGCACA
>random_seq_from_cds__NO_2975 RANDOM_LENGTH 81 SEQ_LENGTH 54
GC_CONTENT 0.481481481481481 BASEPAIR 20 FREE_ENERGY
-17.60 LEN_BP_RATIO 2.7
UUUCAAGUGCAUGGCUUUUCUCUGGUCCUUGAGCCACAGCUCGUAGGCUGUUUUCUGCAUCAUAG
GGUUACAGAAUCGAAU
>random_seq_from_cds__NO_2980 RANDOM_LENGTH 86 SEQ_LENGTH 69
GC_CONTENT 0.463768115942029 BASEPAIR 26 FREE_ENERGY
-23.30 LEN_BP_RATIO 2.65384615384615
UUUCCUUUUAAUGACGUUGGAGGUGACAGGUUUUUCAGUCUGACACCACUUGUGAGGUGACAGAC
UUCUUCACUUUCCUUAUCACU
>random_seq_from_cds__NO_2981 RANDOM_LENGTH 84 SEQ_LENGTH 64
GC_CONTENT 0.390625 BASEPAIR 24 FREE_ENERGY -17.80
LEN_BP_RATIO 2.66666666666667
AUGGAGAGUAACCAUGUUUAACUUCUCUGUUAGCUUAAUGGAAUACUCUGAACAGGUUAUUCCAG
GUCCUAUUUGUCUUUUCCU
>random_seq_from_cds__NO_2997 RANDOM_LENGTH 72 SEQ_LENGTH 48
GC_CONTENT 0.5 BASEPAIR 19 FREE_ENERGY -21.50 LEN_BP_RATIO
2.52631578947368
GACAGAAGUGAUGUGCAUAUAGGCAUCCUGGAUGGCUGGGCCUAUCUCUUCUGCUUUGUCUUGGU
CUUCAAA
>random_seq_from_cds__NO_3009 RANDOM_LENGTH 66 SEQ_LENGTH 54
GC_CONTENT 0.555555555555556 BASEPAIR 19 FREE_ENERGY
-19.10 LEN_BP_RATIO 2.84210526315789
AAGGCGACCCUACGGAGAAGCAACUUCAGAUCAUCCUGGAGGAUGCACCUCUCUGGCAGAGAUUC
A
>random_seq_from_cds__NO_3011 RANDOM_LENGTH 71 SEQ_LENGTH 52
GC_CONTENT 0.557692307692308 BASEPAIR 20 FREE_ENERGY
-20.80 LEN_BP_RATIO 2.6
CCCCAAUGCCAUGUACUCCCUCCUGCUGGACUUUGUCCCUACGGACAGUCACCGCUGGAAGUACG
UCAACG
>random_seq_from_cds__NO_3017 RANDOM_LENGTH 72 SEQ_LENGTH 62

GC_CONTENT 0.661290322580645 BASEPAIR 20 FREE_ENERGY -19.00 LEN_BP_RATIO 3.1
UGCCGCUCCUCUGCCUCUGCCUGCUCCCCACACCCACCAUGGCUGUGAGCACUAUUCGGGUCUCC
GAGGACA
>random_seq_from_cds__NO_3023 RANDOM_LENGTH 81 SEQ_LENGTH 66 GC_CONTENT 0.696969696969697 BASEPAIR 26 FREE_ENERGY -29.60 LEN_BP_RATIO 2.53846153846154
CACCUGGACAGCAGUGGCCUCGCAUCCCUUCGCGGGCUGGGGUGGCCCAGGAGCGGGUGGGCACC
AUUCUCCUUCCUCACU
>random_seq_from_cds__NO_3031 RANDOM_LENGTH 70 SEQ_LENGTH 57 GC_CONTENT 0.421052631578947 BASEPAIR 19 FREE_ENERGY -16.90 LEN_BP_RATIO 3
AUUCCUUGUGCUGUUCCCUUUGAAGAUCUGCCAGUUGAUCUGGUUGGAACUGUAAGCUACAUAGA
ACUCU
>random_seq_from_cds__NO_3040 RANDOM_LENGTH 73 SEQ_LENGTH 61 GC_CONTENT 0.491803278688525 BASEPAIR 19 FREE_ENERGY -20.00 LEN_BP_RATIO 3.21052631578947
CAUAGGCAUGUUGCUGUCCUUAUGUAGUAUUCCUUUUUGGCAGAUUAGGAGGGGACCUAUCAAGC
CUGAGUGA
>random_seq_from_cds__NO_3048 RANDOM_LENGTH 102 SEQ_LENGTH 57 GC_CONTENT 0.456140350877193 BASEPAIR 21 FREE_ENERGY -15.91 LEN_BP_RATIO 2.71428571428571
UAACAUCAGUUUUGUAGGGGUCAUCAUAGGGCACAUAAUCAAUUUCAGCAUAGUCAUCUUCACUG
CUCUGGACCUCUUCCUUUGGAAUGAUCUCAAUGUAAU
>random_seq_from_cds__NO_3058 RANDOM_LENGTH 81 SEQ_LENGTH 72 GC_CONTENT 0.513888888888889 BASEPAIR 20 FREE_ENERGY -21.90 LEN_BP_RATIO 3.6
GUCUGGCUGAGGUCUAAAGAAAGGGUUGUAUGGCUGAGGUCUGGAGAAAGGGUUGUAUGGCUGAG
GUCUGGAGAAAUGGGC
>random_seq_from_cds__NO_3059 RANDOM_LENGTH 86 SEQ_LENGTH 67 GC_CONTENT 0.552238805970149 BASEPAIR 25 FREE_ENERGY -20.90 LEN_BP_RATIO 2.68
AUCUGACCGAGGGCUGGGGAAAGGUUUCUCUGAAUGAGUUCUGGAGAGAGAGUCGUGUGGCUGAG
GUCUGGAGAGAGGUUUGUCUG
>random_seq_from_cds__NO_3063 RANDOM_LENGTH 83 SEQ_LENGTH 59 GC_CONTENT 0.525423728813559 BASEPAIR 23 FREE_ENERGY -26.50 LEN_BP_RATIO 2.56521739130435
UCUGAUAAAGACCUGGAGGACAGCUUGCCUGACCAGUGUCAUUUGAGGAAUUCUGAUUAUGGUCA
GGAAGUGAGGCUAUCCAG
>random_seq_from_cds__NO_3077 RANDOM_LENGTH 84 SEQ_LENGTH 49 GC_CONTENT 0.448979591836735 BASEPAIR 20 FREE_ENERGY -16.50 LEN_BP_RATIO 2.45
CAUAGUCAGCAUCACUCUCUUCAUCUUCAGGUUCUAAACGAUCAUGCAUUUUCCGUGUAGCCAUG
ACUGUAGAUUCUGGAGGUU

>random_seq_from_cds__NO_3105 RANDOM_LENGTH 87 SEQ_LENGTH 58 GC_CONTENT 0.482758620689655 BASEPAIR 24 FREE_ENERGY -25.40 LEN_BP_RATIO 2.41666666666667
CCCCAGAGGUUGGAGCAGUUCAUCGCUAUUGGCUUAUUAACAUGUAGUUCUGAGCAUUUCACAGC
CUCUGCAUGCUGGAGUUACUGC
>random_seq_from_cds__NO_3107 RANDOM_LENGTH 87 SEQ_LENGTH 79 GC_CONTENT 0.569620253164557 BASEPAIR 27 FREE_ENERGY -27.70 LEN_BP_RATIO 2.92592592592593
GCCUACAGUACAUGGUUCCCUGCCCAGGAGUGGUGAGGGCUGGACAUUGCACCCCUGGAGUAGGA
AGUGAUGCUAUGCCCUUUGCAG
>random_seq_from_cds__NO_3117 RANDOM_LENGTH 95 SEQ_LENGTH 73 GC_CONTENT 0.616438356164384 BASEPAIR 19 FREE_ENERGY -26.25 LEN_BP_RATIO 3.84210526315789
CAGGUUGGCAAGGGUGCAGACCAGUGUCCAGAGUCAAUGCAGCGGAGCAUGUCCAAGCCCCUCAC
UCUGUAGCCGGGCUGGCACUCAAAUUUACA
>random_seq_from_cds__NO_3122 RANDOM_LENGTH 66 SEQ_LENGTH 49 GC_CONTENT 0.591836734693878 BASEPAIR 20 FREE_ENERGY -19.60 LEN_BP_RATIO 2.45
CAGAAGCCAAGCAUUCCAGCUUGCUGGGCCCAUUUACUUGGUACCCGUCAGUGCAGUGGAAGCUG
C
>random_seq_from_cds__NO_3127 RANDOM_LENGTH 97 SEQ_LENGTH 82 GC_CONTENT 0.51219512195122 BASEPAIR 25 FREE_ENERGY -21.40 LEN_BP_RATIO 3.28
UUGUUGUUAGGUUCAUUAUCAGCCCAGUUCUCAGCCUCGUUGGUGAGAGCCUUUUUGGUUCCCAC
CCAUGUCCAUGUCUUAUUGUUCUUUCGGAUCC
>random_seq_from_cds__NO_3128 RANDOM_LENGTH 81 SEQ_LENGTH 63 GC_CONTENT 0.365079365079365 BASEPAIR 24 FREE_ENERGY -19.20 LEN_BP_RATIO 2.625
CAAUCCAGUAGUAGGAGCUGUAGUAGGGUAGGACCUUAUUGAGGUAAUCAAUUUCAUUUUUAUUC
UGGAUGGCCACUAAGU
>random_seq_from_cds__NO_3129 RANDOM_LENGTH 77 SEQ_LENGTH 50 GC_CONTENT 0.44 BASEPAIR 19 FREE_ENERGY -15.90 LEN_BP_RATIO 2.63157894736842
CUGUGUAGCGAUUCUGGCAGUAUUUACGGGAAAUAUUCCAUGAGUAUGCUUUUGUGCUGUAAUGA
UAAGUCCAUGCU
>random_seq_from_cds__NO_3145 RANDOM_LENGTH 81 SEQ_LENGTH 74 GC_CONTENT 0.445945945945946 BASEPAIR 23 FREE_ENERGY -22.41 LEN_BP_RATIO 3.21739130434783
UUAAAGGAUGUAAGAAGGCUUUUGGUAGCUUCCAUCUGAUUCAAGGCUUUGGCAGCUGCCUGGCA
GGAACAAAUUUCUUUG
>random_seq_from_cds__NO_3147 RANDOM_LENGTH 79 SEQ_LENGTH 61 GC_CONTENT 0.60655737704918 BASEPAIR 22 FREE_ENERGY -27.60 LEN_BP_RATIO 2.77272727272727
CAAGGGAAUGUUGGACUCAGUGGGAGCCUUCACAGGUAGGUAGCAGGCCAGACCAGUGUCCUGUG

GCUCCACAUGUCCG
>random_seq_from_cds__NO_3153 RANDOM_LENGTH 70 SEQ_LENGTH 51 GC_CONTENT 0.568627450980392 BASEPAIR 20 FREE_ENERGY -18.60 LEN_BP_RATIO 2.55
GGAUUUGCUGUGUCCACUGCCCUUGAGUGGUGCAUUCAACCUGGGCUGGUCCCUGCAACAUGAAGCCUUC
>random_seq_from_cds__NO_3164 RANDOM_LENGTH 97 SEQ_LENGTH 72 GC_CONTENT 0.527777777777778 BASEPAIR 22 FREE_ENERGY -19.80 LEN_BP_RATIO 3.27272727272727
CAGGUGUGUGUACCUUUGCUGACAAUAAGCACUGGCCUCAUCAUAAGUCAUAGCUUCCGUGGAGGUGUUGUAAGACCAGGCUCCACUCUCUUUAAUG
>random_seq_from_cds__NO_3166 RANDOM_LENGTH 70 SEQ_LENGTH 52 GC_CONTENT 0.557692307692308 BASEPAIR 19 FREE_ENERGY -16.70 LEN_BP_RATIO 2.73684210526316
UGAUUGGAGCUGGGGUCAGUGGCCUAAUUUCUCUGAAGUGCUGUGUGGAUGAGGGACUUGAGCCCACUUG
>random_seq_from_cds__NO_3175 RANDOM_LENGTH 90 SEQ_LENGTH 56 GC_CONTENT 0.410714285714286 BASEPAIR 21 FREE_ENERGY -15.70 LEN_BP_RATIO 2.66666666666667
GAACAACAGAUGAAUCGGUGGUUCAACCAUGAAAAUUAUGGCCUUGAGCCUCAAAACAAGAUACAUUAUGAAGGAACCUGUACUAAAUGA
>random_seq_from_cds__NO_3186 RANDOM_LENGTH 74 SEQ_LENGTH 63 GC_CONTENT 0.507936507936508 BASEPAIR 24 FREE_ENERGY -21.30 LEN_BP_RATIO 2.625
AAAAUGCUCAGAUUCUGCUGUCUCUGGCCAAUGGGAGGUGGUCACUAUGCAUGAAGAGAAGCAAGAGUCAGCCA
>random_seq_from_cds__NO_3233 RANDOM_LENGTH 97 SEQ_LENGTH 69 GC_CONTENT 0.739130434782609 BASEPAIR 26 FREE_ENERGY -37.57 LEN_BP_RATIO 2.65384615384615
GUUUCCAGCUGGUCCCGCUCCCGCCUCAGGGUGCCCAGCUCCCUCUGCAGCCCCUCCUGGGUCUCCUGGGGCCUGGCAGCCUGGUCCAAGGUCAAUU
>random_seq_from_cds__NO_3236 RANDOM_LENGTH 80 SEQ_LENGTH 62 GC_CONTENT 0.693548387096774 BASEPAIR 23 FREE_ENERGY -28.62 LEN_BP_RATIO 2.69565217391304
GCCUUCCUGAGCUGAGCUGUCCUGGCCCCCACAUCCCACACCAGGCAGGCCAGAAGCAGCAGCUGGACAGCUGGCAUCUC
>random_seq_from_cds__NO_3249 RANDOM_LENGTH 86 SEQ_LENGTH 69 GC_CONTENT 0.565217391304348 BASEPAIR 23 FREE_ENERGY -20.80 LEN_BP_RATIO 3
UCUACAUAGUGACUUCGAACUUGGCCUGAAGCUGAUGAAGGAACCAUCCUGCUUGAACGUCCGCAGCAGCGAGCCCAGCAUGAAGG
>random_seq_from_cds__NO_3254 RANDOM_LENGTH 87 SEQ_LENGTH 63 GC_CONTENT 0.587301587301587 BASEPAIR 23 FREE_ENERGY -23.71 LEN_BP_RATIO 2.73913043478261

AGAGACUUCAACAUCCCUGGCUUCCCGACUGUGAGGGUUCUUCAAGGCCUUUACCAAGAACGGCU
CGGGAGCAGUAUUUCCAGGUGG
>random_seq_from_cds__NO_3258 RANDOM_LENGTH 81 SEQ_LENGTH 74
GC_CONTENT 0.567567567567568 BASEPAIR 22 FREE_ENERGY
-21.70 LEN_BP_RATIO 3.36363636363636
CCUGCUGUUCCGGAAUGGCUCUGUCUCCCGAGUCCCCGUGGCUCAUGGAAUCCAGGUCCUUCUAU
ACCGCUUACCUGCAGA
>random_seq_from_cds__NO_3274 RANDOM_LENGTH 80 SEQ_LENGTH 60
GC_CONTENT 0.583333333333333 BASEPAIR 23 FREE_ENERGY
-33.00 LEN_BP_RATIO 2.60869565217391
GGCUCUAUUCCCUGUCCUUCAUGGGCCUGCUGGCCAUGUACACCUACUUCCAGGCCAAGAUAAGG
GCCCUGAAGGGCCAU
>random_seq_from_cds__NO_3278 RANDOM_LENGTH 90 SEQ_LENGTH 61
GC_CONTENT 0.688524590163934 BASEPAIR 23 FREE_ENERGY
-28.90 LEN_BP_RATIO 2.65217391304348
GGUCACCAAGUCCUGUCACCUGUGCGACGCCGGGCAGCCCCACCUGCAGCACGGGGCAGCCUUCC
UGACCGACUACAACAACCAGGCCGA
>random_seq_from_cds__NO_3302 RANDOM_LENGTH 92 SEQ_LENGTH 81
GC_CONTENT 0.530864197530864 BASEPAIR 29 FREE_ENERGY
-21.50 LEN_BP_RATIO 2.79310344827586
ACUAUGGAGAUUCAACUGCAGGCACCUCCUCCGAUUGCCAACCCUGUCCGUGUCCUGGAGGUUCA
AGUUGUGCUGUUGUUCCCAAGACAAAG
>random_seq_from_cds__NO_3308 RANDOM_LENGTH 70 SEQ_LENGTH 58
GC_CONTENT 0.637931034482759 BASEPAIR 21 FREE_ENERGY
-21.30 LEN_BP_RATIO 2.76190476190476
UCUGCAGAGUGGGCAAGGCUGUGAGAGGGUGUGACUGCCAUGCCUUGGGCUCCACCAAUGGGCAG
UGUGA
>random_seq_from_cds__NO_3312 RANDOM_LENGTH 88 SEQ_LENGTH 71
GC_CONTENT 0.47887323943662 BASEPAIR 22 FREE_ENERGY
-20.30 LEN_BP_RATIO 3.22727272727273
CAGGAAUUAGAGAGUCUCAUAGCAAACCUUGGAACUGGGGAUGAGAUGGUGACAGAUCAAGCCUU
CGAGGAUAGACUAAAGGAAGCAG
>random_seq_from_cds__NO_3328 RANDOM_LENGTH 110 SEQ_LENGTH 85
GC_CONTENT 0.505882352941176 BASEPAIR 27 FREE_ENERGY
-24.20 LEN_BP_RATIO 3.14814814814815
CAAGAAGCCGAGAUCAAUGCCAGAAAAGCCAAAAACUCUGUUACUAGCCUCCUCAGCAUUAUUAA
UGACCUCUUGGAGCAGCUGGGGGCAGCUGGAUACAGUGGACCUGA
>random_seq_from_cds__NO_3331 RANDOM_LENGTH 89 SEQ_LENGTH 78
GC_CONTENT 0.538461538461538 BASEPAIR 24 FREE_ENERGY
-17.50 LEN_BP_RATIO 3.25
CCAUCUGGCUGCUUCAACACCCCGUCCAUUGAAAAGCCCUAGCCUAGACUUCUCUCCGAGUGCUU
UCCAAAUCUGUAGUUGCGCAGUCU
>random_seq_from_cds__NO_3334 RANDOM_LENGTH 88 SEQ_LENGTH 79
GC_CONTENT 0.455696202531646 BASEPAIR 27 FREE_ENERGY

-18.30 LEN_BP_RATIO 2.92592592592593
UGCCUCCACUUGGCUGCCUUUCUUAAGCUGAGGUUCUGUUGUCUGGUUAUUAGCAUCAGCUUUUU
CACUUUCCUUGGGUUCAUCUGGA
>random_seq_from_cds__NO_3360 RANDOM_LENGTH 83 SEQ_LENGTH 60
GC_CONTENT 0.5 BASEPAIR 20 FREE_ENERGY -22.50 LEN_BP_RATIO
3
UACUUCUAUGCCUUUGUUGCAUCGGGCUGCAGCUCCUUGCCACUGCAGCUACAACAAUUCUGGCU
GCUAGUUCCUUGUAAUAU
>random_seq_from_cds__NO_3377 RANDOM_LENGTH 78 SEQ_LENGTH 70
GC_CONTENT 0.442857142857143 BASEPAIR 20 FREE_ENERGY
-18.00 LEN_BP_RATIO 3.5
AGGAUUUAUCAAGCUGUGCAGGGAGAUGUGGGGAAGGGUAUUCUAGAGAUGCCACCUGCAACUGU
GAUUAUAACUGUC
>random_seq_from_cds__NO_3417 RANDOM_LENGTH 80 SEQ_LENGTH 57
GC_CONTENT 0.473684210526316 BASEPAIR 22 FREE_ENERGY
-16.63 LEN_BP_RATIO 2.59090909090909
GCAUUCCGAGGGUCAUUAUUUCUGGAUGCUAAGUCCAUUCAGUCCACCAUCUCCAGCUCGCAGAA
UUACUGAAGUUUGGG
>random_seq_from_cds__NO_3419 RANDOM_LENGTH 95 SEQ_LENGTH 81
GC_CONTENT 0.469135802469136 BASEPAIR 29 FREE_ENERGY
-18.90 LEN_BP_RATIO 2.79310344827586
CUGGCGUUUUACCAAUGAUAUAAAAGAUGCAGGGUACCCCAAACCAAUUUUCAAAGGAUUUGGAG
GACUAACUGGACAAAUAGUGGCAGCGCUUU
>random_seq_from_cds__NO_3436 RANDOM_LENGTH 68 SEQ_LENGTH 49
GC_CONTENT 0.510204081632653 BASEPAIR 19 FREE_ENERGY
-21.30 LEN_BP_RATIO 2.57894736842105
CCGUAGAUGCUCAGGGACUUGAGGAGGGUAGAUCAUCUCUGCCUGAGUAUCUUUGACUGUGGGAG
GAU
>random_seq_from_cds__NO_3441 RANDOM_LENGTH 82 SEQ_LENGTH 62
GC_CONTENT 0.403225806451613 BASEPAIR 27 FREE_ENERGY
-19.10 LEN_BP_RATIO 2.2962962962963
UUUGUAGCCAUAGUCAGCAUUGUAAGUUGGUGGACUGUCAAUCAAAUGUGAUCUGGCAUGUCAAC
ACAUAACUCAUAAUUGC
>random_seq_from_cds__NO_3445 RANDOM_LENGTH 84 SEQ_LENGTH 60
GC_CONTENT 0.5 BASEPAIR 24 FREE_ENERGY -20.00 LEN_BP_RATIO
2.5
AGCAGCAGGGCGCGGGCGAGCAUUCUAUGUUCUUAAGGGUUCUUGAUAAGACAGAGUGCCUUUGU
CUUGGAAUACAUCUUGGAU
>random_seq_from_cds__NO_3452 RANDOM_LENGTH 90 SEQ_LENGTH 78
GC_CONTENT 0.371794871794872 BASEPAIR 24 FREE_ENERGY
-20.47 LEN_BP_RATIO 3.25
CCCUUCAUAUUUCCACUUCAUUUCUAUGUUAUUUCUGUUCAUGUAAUCCACAUUAACAGUACAUG
GUUCCCAAGCAAACAGGUGGGGAUG
>random_seq_from_cds__NO_3461 RANDOM_LENGTH 97 SEQ_LENGTH 73

GC_CONTENT 0.479452054794521 BASEPAIR 25 FREE_ENERGY -19.81 LEN_BP_RATIO 2.92
GCACUUUAAUUCUGGUACAUUUUGGUGUGAGAGACCAUCCGUAUGUGAGACAUUCUACCUCCUCUGUCUUCUUUCCUCCAGCUGUGUAGUAGCCAGU
>random_seq_from_cds__NO_3466 RANDOM_LENGTH 85 SEQ_LENGTH 67 GC_CONTENT 0.328358208955224 BASEPAIR 24 FREE_ENERGY -15.50 LEN_BP_RATIO 2.79166666666667
UUUUGUCUAUGCUCAUUGGAAAGUAAAAGCUUUUAAAAGUAUAGUAAUAUUGGGCAAUUCUUCCAUUUUCCACAUGAGGAAAACC
>random_seq_from_cds__NO_3468 RANDOM_LENGTH 77 SEQ_LENGTH 69 GC_CONTENT 0.666666666666667 BASEPAIR 27 FREE_ENERGY -38.20 LEN_BP_RATIO 2.55555555555556
GCCUUGGAGGAAUAAGGGUCUCCUGGGAGCCCUGGUGUUGGUCGAGGCUGCCCAGGGAGGACCCGAGGUUCAGGCAU
>random_seq_from_cds__NO_3489 RANDOM_LENGTH 81 SEQ_LENGTH 67 GC_CONTENT 0.567164179104478 BASEPAIR 23 FREE_ENERGY -18.40 LEN_BP_RATIO 2.91304347826087
UCCCAAACAUCCUGCAUGCCGAUGACAGCGUAGAUGAAGAAGAGCAUGACGAUGAGCAGAGCCACGUAGGGUAGGGCCCUG
>random_seq_from_cds__NO_3498 RANDOM_LENGTH 95 SEQ_LENGTH 77 GC_CONTENT 0.584415584415584 BASEPAIR 25 FREE_ENERGY -29.70 LEN_BP_RATIO 3.08
CAUUGGAGUCUAUGGCCUUGUACAGCAGCCUGAGGCCAUCCCUCGAAGGUGGAGACCGUGAAGAGGGACAUCAUGGCUGAGAGCACAUUGUCGAA
>random_seq_from_cds__NO_3511 RANDOM_LENGTH 73 SEQ_LENGTH 64 GC_CONTENT 0.609375 BASEPAIR 23 FREE_ENERGY -26.60 LEN_BP_RATIO 2.78260869565217
GGCAGAAGUCAGGCUCUCCGCCUCGGCCAGGUUGUCCACGGCAAUGGCCAGGAAGACAUUGAGCAGGAUGUCA
>random_seq_from_cds__NO_3514 RANDOM_LENGTH 84 SEQ_LENGTH 69 GC_CONTENT 0.536231884057971 BASEPAIR 23 FREE_ENERGY -16.70 LEN_BP_RATIO 3
UGAGGGCUUGGGGAAAGUUGUCAAAGUUGCUGCGCCGUACUUCUGUGUCUUCAAAGUCAUACCUCCCCCCAAAGAGCUGCAUGC
>random_seq_from_cds__NO_3534 RANDOM_LENGTH 88 SEQ_LENGTH 67 GC_CONTENT 0.582089552238806 BASEPAIR 27 FREE_ENERGY -34.30 LEN_BP_RATIO 2.48148148148148
UCAGGCAGAACAAGGCCCGGGGUGGCCUUGGCAGAAUCUCAGGAACUGGCUUCUUGGGCUGUUUCUUCCUCAGGCCUUCAUCCUGGGG
>random_seq_from_cds__NO_3540 RANDOM_LENGTH 90 SEQ_LENGTH 74 GC_CONTENT 0.567567567567568 BASEPAIR 26 FREE_ENERGY -23.30 LEN_BP_RATIO 2.84615384615385
GCCAUAGCCUUUGGGCCCAUACUUCUUGCCGUAGCAGGACUUGCAGUAAAUCUCCUCACCAUGCACGGCCACAGUGGUACUGUCCAGAUU

>random_seq_from_cds__NO_3544 RANDOM_LENGTH 69 SEQ_LENGTH 55 GC_CONTENT 0.763636363636364 BASEPAIR 21 FREE_ENERGY -29.50 LEN_BP_RATIO 2.61904761904762
CCAAGGCGCCCGGCGGCGGCGGCGGCAUGGCCAAGGCCAGCGCGGCUGAGCUGAAGGUCUUCAAG UCCG
>random_seq_from_cds__NO_3546 RANDOM_LENGTH 110 SEQ_LENGTH 86 GC_CONTENT 0.604651162790698 BASEPAIR 31 FREE_ENERGY -40.70 LEN_BP_RATIO 2.7741935483871
AAAAAGCUGCAGCUUUAUGAGCCCGAAUGGAGCGACGAUAUGGCCAAGGCGCCCAAAGGCUUAGG CAAGGUGGGGUCCAAGGGCCGUGAAGCUCCGCUGAUGUCCAAGAC
>random_seq_from_cds__NO_3549 RANDOM_LENGTH 84 SEQ_LENGTH 72 GC_CONTENT 0.666666666666667 BASEPAIR 25 FREE_ENERGY -31.70 LEN_BP_RATIO 2.88
CUCUCCAGCAAGGCCAAGGCGCAAAAGAGCUCUGGGCCUGUCCCCUCUGCCAAGGGCCAGGAGGA GCGCGCCUUCCUCAAGGUG
>random_seq_from_cds__NO_3551 RANDOM_LENGTH 78 SEQ_LENGTH 65 GC_CONTENT 0.615384615384615 BASEPAIR 23 FREE_ENERGY -27.10 LEN_BP_RATIO 2.82608695652174
AAAGAGGACAGUGCAGAAUGUCCUGGAUCUCCGGCAGAACCUGGAAGAGACCAUGUCCAGCCUGC GAGGGUCCCAGGU
>random_seq_from_cds__NO_3552 RANDOM_LENGTH 92 SEQ_LENGTH 71 GC_CONTENT 0.619718309859155 BASEPAIR 23 FREE_ENERGY -19.20 LEN_BP_RATIO 3.08695652173913
GACUCACAGGCUCCCUGGAGAUGACCUGCUACGACAGCGAUGAUGCCAACCCACGCAGCGUGUCC AGCCUCUCCAACCGCUCGUCCCCUCUG
>random_seq_from_cds__NO_3571 RANDOM_LENGTH 93 SEQ_LENGTH 77 GC_CONTENT 0.584415584415584 BASEPAIR 23 FREE_ENERGY -20.70 LEN_BP_RATIO 3.34782608695652
CAUCCAGUGAUACCACCCAUGCUUCAAAGGUCCCAGAUCUGCAUGCUACAAGCUCAGCAUCUGGG GGCCCUCUCCCUUCCUGCUUCACCCCCA
>random_seq_from_cds__NO_3572 RANDOM_LENGTH 89 SEQ_LENGTH 68 GC_CONTENT 0.529411764705882 BASEPAIR 20 FREE_ENERGY -20.20 LEN_BP_RATIO 3.4
GUCCGGCACCCAUCCUCAAUAUUAACUCAGCCAGCUUCUCCCAGGGCCUGGAGCUAAUGAGUGGU UUCAGUGUGCCAAAAGAGACCCGC
>random_seq_from_cds__NO_3574 RANDOM_LENGTH 86 SEQ_LENGTH 64 GC_CONTENT 0.640625 BASEPAIR 19 FREE_ENERGY -19.20 LEN_BP_RATIO 3.36842105263158
AGCAGUACUCCCGUCCCCACCCCACCUGCUCCCCCUGCUGCUCCCACAGAAGAAGAGACGGAAGA GCUGACUUGGAGUGGAAGCCC
>random_seq_from_cds__NO_3575 RANDOM_LENGTH 82 SEQ_LENGTH 69 GC_CONTENT 0.579710144927536 BASEPAIR 19 FREE_ENERGY -21.70 LEN_BP_RATIO 3.63157894736842
CAGAGCUGGGCAACUGGACAGGUAAUCAGCGGGAUCGGAACACUCUUCCCAAGAAAGGGCUCAGG

GUACCAGCUUCAGUCCC
>random_seq_from_cds__NO_3584 RANDOM_LENGTH 78 SEQ_LENGTH 63 GC_CONTENT 0.507936507936508 BASEPAIR 21 FREE_ENERGY -16.50 LEN_BP_RATIO 3
AUCUCAAGCCUCAACAGCAUCACUAGCCAUUCCAGCAUCGGCAGCAGCAAGGAUGCUGAUGCGAAAAAGAAGAAAAAA
>random_seq_from_cds__NO_3594 RANDOM_LENGTH 83 SEQ_LENGTH 68 GC_CONTENT 0.470588235294118 BASEPAIR 20 FREE_ENERGY -16.80 LEN_BP_RATIO 3.4
UUCCAAGUGUUCAAGGGACUAUAUUUCUAAAAUGGACCCAGCCUCUACCCUGGGACUAAGCACUGAGUCCAUCCAUGGCUACA
>random_seq_from_cds__NO_3614 RANDOM_LENGTH 82 SEQ_LENGTH 77 GC_CONTENT 0.597402597402597 BASEPAIR 26 FREE_ENERGY -27.30 LEN_BP_RATIO 2.96153846153846
CAGCAGCUUGGCGAUCUCCAUCUCCGUCUUGCCUCCACAGAUGUGUCGCUGGUUGUGGAAGCGGAGCUCGGUCAGCGUGUUG
>random_seq_from_cds__NO_3617 RANDOM_LENGTH 110 SEQ_LENGTH 99 GC_CONTENT 0.505050505050505 BASEPAIR 33 FREE_ENERGY -24.10 LEN_BP_RATIO 3
AACUCCAGAGCCUCAGUAAACCGGACCAAGAUCUCAUUUGUGAUGCAGUCUGAGUUGUUGACGUUCACCUCAGUCAUCUCGGGGUCAUUGUUCUUCACUCUCUCCAGAGG
>random_seq_from_cds__NO_3618 RANDOM_LENGTH 87 SEQ_LENGTH 59 GC_CONTENT 0.677966101694915 BASEPAIR 22 FREE_ENERGY -26.90 LEN_BP_RATIO 2.68181818181818
CUCAUCAAAUAUGCUGGGAGCUGCCUCCUCCUCCACCUUGGCCGGUCCUUCAGAGGGCUUGGUGGGGCCACUGGGCGUCUGUUUCUC
>random_seq_from_cds__NO_3627 RANDOM_LENGTH 75 SEQ_LENGTH 52 GC_CONTENT 0.596153846153846 BASEPAIR 20 FREE_ENERGY -15.80 LEN_BP_RATIO 2.6
CACAGAAGUUGAGCAUGGCCUCCCGGUUGUACACACCCGUGGACUGUUUCUCCGUCUGGUUUCUCUGCCGCAGCC
>random_seq_from_cds__NO_3638 RANDOM_LENGTH 68 SEQ_LENGTH 58 GC_CONTENT 0.655172413793103 BASEPAIR 24 FREE_ENERGY -26.90 LEN_BP_RATIO 2.41666666666667
CGGGAUGCCACCUUCUGCUUCAUCGUGUCGCUGGCGGUGGCUGAUGUGGCCGUGGGUGCCCUGGUCAU
>random_seq_from_cds__NO_3641 RANDOM_LENGTH 77 SEQ_LENGTH 52 GC_CONTENT 0.769230769230769 BASEPAIR 20 FREE_ENERGY -31.40 LEN_BP_RATIO 2.6
GUGACCCCCCGGAGGGCGGCGGUGGCCAUAGCCGGCUGCUGGAUCCUCUCCUUCGUGGUGGGACUGACCCCUAUGUU
>random_seq_from_cds__NO_3649 RANDOM_LENGTH 68 SEQ_LENGTH 56 GC_CONTENT 0.607142857142857 BASEPAIR 20 FREE_ENERGY -24.61 LEN_BP_RATIO 2.8

GUCGGGCAGCUUUGCUGGAACAGCCGCCCCGCUGCACAGCUGUAGAAGCUGGACCGUUCCCGAGG
AUU
>random_seq_from_cds__NO_3652 RANDOM_LENGTH 97 SEQ_LENGTH 81
GC_CONTENT 0.654320987654321 BASEPAIR 26 FREE_ENERGY
-30.60 LEN_BP_RATIO 3.11538461538462
UGGAUGAGGGGGUAUCGGCCCUGGUUGCAGGAGAAGCCGGCAAAGUCAUCUAAGUCCAGUGCCCA
GACCAUGGCCCCGCCCAGUCCCUUCUGCUUCA
>random_seq_from_cds__NO_3654 RANDOM_LENGTH 72 SEQ_LENGTH 57
GC_CONTENT 0.56140350877193 BASEPAIR 20 FREE_ENERGY
-17.90 LEN_BP_RATIO 2.85
UGAUCCUGGAUUCUCUGUUUGGUGGCCCCCUUCCAGGAGCAGACCUUCAUAGUAGGCCAGCAUCC
CUCCUUC
>random_seq_from_cds__NO_3658 RANDOM_LENGTH 71 SEQ_LENGTH 43
GC_CONTENT 0.558139534883721 BASEPAIR 19 FREE_ENERGY
-20.15 LEN_BP_RATIO 2.26315789473684
AAUCCAGGUUCCUGGGCGAUUUUGUCCACCUCGUAUCCAGCAUCCACAUAGGUCUGCCCAGCUGG
AACCGC
>random_seq_from_cds__NO_3661 RANDOM_LENGTH 82 SEQ_LENGTH 75
GC_CONTENT 0.533333333333333 BASEPAIR 26 FREE_ENERGY
-20.00 LEN_BP_RATIO 2.88461538461538
GGCCGAGUUGACAAAGGUCUGACGGUUGUUGGCCGUGGCUACCAUAUCUGUGAACCUUCUGAGUG
CCGAAAUUCCAGCCUCC
>random_seq_from_cds__NO_3670 RANDOM_LENGTH 68 SEQ_LENGTH 54
GC_CONTENT 0.555555555555556 BASEPAIR 21 FREE_ENERGY
-23.10 LEN_BP_RATIO 2.57142857142857
UCAGGUCCAGCAAGAUCAGUGACCGGAGGCCCCUCAUGGAACUGCCCACUUCCUGGAUCUCAUUG
UGU
>random_seq_from_cds__NO_3687 RANDOM_LENGTH 85 SEQ_LENGTH 77
GC_CONTENT 0.532467532467532 BASEPAIR 27 FREE_ENERGY
-24.50 LEN_BP_RATIO 2.85185185185185
UGUCCUGGCUGAGAAAGCCACUGACUGUCCCUGUUGAAUAGCGGAGGGUGAGUUCUGUUCCAUUG
UGCUUGUAGCUGGAGGAAUC
>random_seq_from_cds__NO_3699 RANDOM_LENGTH 110 SEQ_LENGTH 99
GC_CONTENT 0.484848484848485 BASEPAIR 36 FREE_ENERGY
-25.00 LEN_BP_RATIO 2.75
CCAACCAUCUCAUUGUAGGUAGGAUUGCAGGUUUUCCGGGCCACUUUGGUUUUCCUCUUAGUGGU
UUUCUGAGGGUCAGGAAGGAGGUAAAUUUUCACAUAGGGGUCAGG
>random_seq_from_cds__NO_3704 RANDOM_LENGTH 89 SEQ_LENGTH 76
GC_CONTENT 0.513157894736842 BASEPAIR 26 FREE_ENERGY
-26.30 LEN_BP_RATIO 2.92307692307692
UGGAACUCCUCAAAGGUCCGCUGGAUGUAGGUGGCCUCGUGAGUGUUCUCUCGCAUCACCUUUAC
CACAUAUAUCAUAGCCUUUGUUGG
>random_seq_from_cds__NO_3708 RANDOM_LENGTH 100 SEQ_LENGTH 88
GC_CONTENT 0.556818181818182 BASEPAIR 30 FREE_ENERGY

-29.80 LEN_BP_RATIO 2.93333333333333
GGCAUCGUACACAUACUUGAGGUCCUCCAGGUCUGAGAGUUCAGGGAUCCCACAGGACAACAUCC
AGGCCCAGAAGGUUGAGGAAGAGGUGGGUGUGCUU
>random_seq_from_cds__NO_3720 RANDOM_LENGTH 71 SEQ_LENGTH 62
GC_CONTENT 0.596774193548387 BASEPAIR 22 FREE_ENERGY
-24.61 LEN_BP_RATIO 2.81818181818182
GCCACAGCAGCACAGUAAGGCUGCCAGCAGAUACUGGUAGCGGAUGCUGAACUGAGAGUCCUUGA
GGCCGU
>random_seq_from_cds__NO_3726 RANDOM_LENGTH 90 SEQ_LENGTH 80
GC_CONTENT 0.5875 BASEPAIR 27 FREE_ENERGY -29.30
LEN_BP_RATIO 2.96296296296296
UGUCUUUAAGCUUGCGCUGGUCUUCUUCCCGGAGGCUGCCAAACUCAUAGCGGGGGCUGAACUUG
UCUCCAGGGGGGCUGGUGAACUUGA
>random_seq_from_cds__NO_3736 RANDOM_LENGTH 69 SEQ_LENGTH 57
GC_CONTENT 0.684210526315789 BASEPAIR 20 FREE_ENERGY
-20.30 LEN_BP_RATIO 2.85
CACGGCGGCCAGGGCGUUGCAGAUGGCCUUGACGGACUGGACCACCCUGUCAGCCUUCAGUGGGA
AGUC
>random_seq_from_cds__NO_3739 RANDOM_LENGTH 81 SEQ_LENGTH 66
GC_CONTENT 0.515151515151515 BASEPAIR 24 FREE_ENERGY
-22.00 LEN_BP_RATIO 2.75
GUAGCCGAAUGUCAAUGUCAAACUUGCGGCAGUAUUGGAUGUACUCAUGACUGCCCAAGGCAUGC
UUGCUUCUGCAGGAAC
>random_seq_from_cds__NO_3745 RANDOM_LENGTH 70 SEQ_LENGTH 58
GC_CONTENT 0.655172413793103 BASEPAIR 20 FREE_ENERGY
-22.00 LEN_BP_RATIO 2.9
CUCCUGGCCACGGGUUUUCCAGAGGUGUCUUUGCUGAAGUCCAGCGGCCCUCGGCCCUCCUUCCA
GCCCC
>random_seq_from_cds__NO_3763 RANDOM_LENGTH 78 SEQ_LENGTH 61
GC_CONTENT 0.39344262295082 BASEPAIR 24 FREE_ENERGY
-18.90 LEN_BP_RATIO 2.54166666666667
UUUGAAUGAGUCAGUAUCAGAGCAGUUAGGUGUUGGAAUAAAAGUUGAAGCUGCUGAUACUGAAC
AAACAAGUGAAGA
>random_seq_from_cds__NO_3771 RANDOM_LENGTH 80 SEQ_LENGTH 69
GC_CONTENT 0.565217391304348 BASEPAIR 21 FREE_ENERGY
-23.74 LEN_BP_RATIO 3.28571428571429
GCCCUUGUCUCCUCUUCAGGCUUGGAGUUCAGCCCUGGGAUCCCAAACCACCUUCGGGCCUGUCU
UUGAAGACCAGCCCC
>random_seq_from_cds__NO_3774 RANDOM_LENGTH 81 SEQ_LENGTH 67
GC_CONTENT 0.641791044776119 BASEPAIR 24 FREE_ENERGY
-28.80 LEN_BP_RATIO 2.79166666666667
UGGGGGGCAACCUGGUCAUCAUGAACCCCACCAAGGCACAGGAUGCCGGGGUCUACCAGUGCCUG
GCCUCCAACCCAGUGG
>random_seq_from_cds__NO_3791 RANDOM_LENGTH 110 SEQ_LENGTH 95

GC_CONTENT 0.621052631578947 BASEPAIR 34 FREE_ENERGY -42.70 LEN_BP_RATIO 2.79411764705882
CAGGUCCCCCAGGAGGUGUGGUGGUGAGGGACAUUGGCGACACCACCAUCCAGCUCAGCUGGAGCCGUGGCUUCGACAACCACAGCCCCAUCGCUAAGUACACCCUGCAA
>random_seq_from_cds__NO_3800 RANDOM_LENGTH 84 SEQ_LENGTH 77 GC_CONTENT 0.701298701298701 BASEPAIR 24 FREE_ENERGY -32.76 LEN_BP_RATIO 3.20833333333333
GUGACCGUGAGGGCCUACAACCGGGCUGGCACUGGGCCUGCCAGCCCUUCUGCCAACGCCACGACCAUGAAGCCCCGCUCCGCG
>random_seq_from_cds__NO_3804 RANDOM_LENGTH 72 SEQ_LENGTH 62 GC_CONTENT 0.629032258064516 BASEPAIR 20 FREE_ENERGY -18.00 LEN_BP_RATIO 3.1
AUCGUGAGGAAUGGAGGGCACAAGCAUGAUGGUGGAGAACAUGGCAGUCCGCCCAGCACCACACCCUGGCAC
>random_seq_from_cds__NO_3810 RANDOM_LENGTH 82 SEQ_LENGTH 76 GC_CONTENT 0.565789473684211 BASEPAIR 24 FREE_ENERGY -26.50 LEN_BP_RATIO 3.16666666666667
UCAUCCAUCAAGGAGGUUUGGACUGCAUCCGGUGGGGGGCCGUAAGGAUUUCUUCUGGGUCUUCUACCUCAGGGGCAAUGG
>random_seq_from_cds__NO_3821 RANDOM_LENGTH 84 SEQ_LENGTH 70 GC_CONTENT 0.471428571428571 BASEPAIR 24 FREE_ENERGY -18.40 LEN_BP_RATIO 2.91666666666667
AUCUCGUGGAUGAUAUUGGACUUUUAAGAUGGGUGAAGGGAAUCGAAACCUCUGGUCACAGUCGCCUGAAAGAACAUCCCACUG
>random_seq_from_cds__NO_3831 RANDOM_LENGTH 100 SEQ_LENGTH 84 GC_CONTENT 0.511904761904762 BASEPAIR 25 FREE_ENERGY -27.10 LEN_BP_RATIO 3.36
CUAUAUUCAUGUACCUCGAGACCAUCUAGCUUAUCGAUAUGAGGUGCUGAAAAUUAUUGGCAAGGGGAGUUUUGGGCAGGUGGCCAGGGUCUAUGAUCAC
>random_seq_from_cds__NO_3837 RANDOM_LENGTH 84 SEQ_LENGTH 66 GC_CONTENT 0.454545454545455 BASEPAIR 19 FREE_ENERGY -17.70 LEN_BP_RATIO 3.47368421052632
CCAGUCUCGGUUCUACAGAGCUCCAGAAAUCAUCUUAGGAAGCCGCUACAGCACACCAAUUGACAUAUGGAGUUUUGGCUGCAU
>random_seq_from_cds__NO_3865 RANDOM_LENGTH 81 SEQ_LENGTH 75 GC_CONTENT 0.586666666666667 BASEPAIR 22 FREE_ENERGY -26.70 LEN_BP_RATIO 3.40909090909091
GGGGUGCAAGCCAUCUGCCUGGAGUUGCCACAGAGCCUUGUUGAAGGUGGGUGUCAGGUCAGGCAACGAAGACAUCUCCAG
>random_seq_from_cds__NO_3870 RANDOM_LENGTH 86 SEQ_LENGTH 70 GC_CONTENT 0.671428571428571 BASEPAIR 26 FREE_ENERGY -30.40 LEN_BP_RATIO 2.69230769230769
CCCUGGAGAGUUCGCCCUGAGGGAGAGGAUGGCACUGGCCACCUGAGCCACCUCCUGCUCUGUGACUGCGGGGCUGCUGAGAACUG

>random_seq_from_cds__NO_3880 RANDOM_LENGTH 68 SEQ_LENGTH 55 GC_CONTENT 0.581818181818182 BASEPAIR 20 FREE_ENERGY -21.10 LEN_BP_RATIO 2.75
CAGAUGCAGGUCUCCUGAAUGGAAGCUCCCGGGCGCCGGUUCCGGAAAUAGUGCAGCUGACACCGCUC
>random_seq_from_cds__NO_3888 RANDOM_LENGTH 70 SEQ_LENGTH 65 GC_CONTENT 0.661538461538462 BASEPAIR 24 FREE_ENERGY -27.30 LEN_BP_RATIO 2.70833333333333
ACUGGCACCGAACAUCCUGCCAGCUCUGAGGCCGACCCUGGCGGACCCGAGGGAAGGUGGAGGUGCAGUC
>random_seq_from_cds__NO_3890 RANDOM_LENGTH 85 SEQ_LENGTH 69 GC_CONTENT 0.623188405797101 BASEPAIR 25 FREE_ENERGY -25.30 LEN_BP_RATIO 2.76
UGGAAUCUCCUGUCCAGGUCCAGCUGCAGAGAGACAGGGUUCACAUCCAUUCUGUGACUGCCACCAGCGCAUGGGGCCGGAGGAU
>random_seq_from_cds__NO_3903 RANDOM_LENGTH 110 SEQ_LENGTH 94 GC_CONTENT 0.595744680851064 BASEPAIR 31 FREE_ENERGY -41.20 LEN_BP_RATIO 3.03225806451613
ACUUCCAUCUCCAGGGGUUCAGUUUUGGGCCACACUGCCUCCGGGCUGCAGUUGCCCACACUGCAAUUGCCCACACUGGCUGGCGCCAUGGGAGAACCCAUUGAUGUUCA
>random_seq_from_cds__NO_3904 RANDOM_LENGTH 84 SEQ_LENGTH 70 GC_CONTENT 0.542857142857143 BASEPAIR 28 FREE_ENERGY -29.60 LEN_BP_RATIO 2.5
GGAAGGGGAAGGUGUCCUGGAUGGGAACAUGGUGCUGCGACUGAUCCAGCUCAUCUUCCUCAUCUUCUUCAUCCACAUCAUUAU
>random_seq_from_cds__NO_3908 RANDOM_LENGTH 72 SEQ_LENGTH 55 GC_CONTENT 0.581818181818182 BASEPAIR 22 FREE_ENERGY -30.00 LEN_BP_RATIO 2.5
GUUGAGGGCUAUGCCGGGUGGCAUGUUUCCAGGGAAUCUGGAAGCGUUUAGAGUCCCUGUGUAGCCAGAUGA
>random_seq_from_cds__NO_3918 RANDOM_LENGTH 99 SEQ_LENGTH 84 GC_CONTENT 0.416666666666667 BASEPAIR 26 FREE_ENERGY -20.42 LEN_BP_RATIO 3.23076923076923
GACUCCAAGUCGAUCAACUUUGCAAAUAGGGAAAAGAGAUGCUAAUAGCAGUUUCUUUGACAAUUCUAGCAGUCCUCAUCUUUUGGAUCAAUUAAAAGC
>random_seq_from_cds__NO_3933 RANDOM_LENGTH 82 SEQ_LENGTH 58 GC_CONTENT 0.396551724137931 BASEPAIR 23 FREE_ENERGY -19.87 LEN_BP_RATIO 2.52173913043478
ACAGCAGAAAGCUGAGUUCUCAGAUCAGAAACAUCAGAAGGAAAUAGAAAAUAUGUGUUUGAAGACUUCUCAGCUUACUGGG
>random_seq_from_cds__NO_3939 RANDOM_LENGTH 81 SEQ_LENGTH 70 GC_CONTENT 0.428571428571429 BASEPAIR 28 FREE_ENERGY -17.60 LEN_BP_RATIO 2.5
AAGUCAGCGCAUUAGUAAGUUACAGGAAGACACUUCUGCUCACCAGAAUGUUGUUGCUGAAACCU

UAAGUGCCCUUGAGAA
>random_seq_from_cds__NO_3956 RANDOM_LENGTH 62 SEQ_LENGTH 54 GC_CONTENT 0.444444444444444 BASEPAIR 20 FREE_ENERGY -21.90 LEN_BP_RATIO 2.7
GAAGACAUACCAGGAGGUGAAUUUGGUGAACAACCAAAUGAACAGCACCCUGUGUCUUUGGC
>random_seq_from_cds__NO_3959 RANDOM_LENGTH 67 SEQ_LENGTH 55 GC_CONTENT 0.454545454545455 BASEPAIR 21 FREE_ENERGY -18.00 LEN_BP_RATIO 2.61904761904762
CUGCAGACCUAUGUUGACUCAUUAAAGGCCGAAAAUUUGGUCUUGUCAACGAAUCUGAGAAACUUUC
>random_seq_from_cds__NO_3978 RANDOM_LENGTH 85 SEQ_LENGTH 60 GC_CONTENT 0.516666666666667 BASEPAIR 20 FREE_ENERGY -16.40 LEN_BP_RATIO 3
GGAGAAAACACAAGAGCUUGAGUCUCAUCAAAGUGAGUGUCUCCAUUGCAUUCAGGUGGCAGAGGCAGAGGUGAAGGAAAAGACG
>random_seq_from_cds__NO_3979 RANDOM_LENGTH 94 SEQ_LENGTH 88 GC_CONTENT 0.454545454545455 BASEPAIR 32 FREE_ENERGY -25.71 LEN_BP_RATIO 2.75
GAACUCCUUCAGACUUUGUCCUCUGAUGUGAGUGAGCUGUUAAAAGACAAAACUCAUCUCCAGGAAAAGCUGCAGAGUUUGGAAAAGGACUCAC
>random_seq_from_cds__NO_3996 RANDOM_LENGTH 78 SEQ_LENGTH 69 GC_CONTENT 0.420289855072464 BASEPAIR 24 FREE_ENERGY -18.70 LEN_BP_RATIO 2.875
UAUUGUUUUGCAAUCUUCAGUGAAUGGCCUCAUUCAAGAAGUAGAAGAUGGCAAGCAGAAACUGGAGAAGAAGGAUGA
>random_seq_from_cds__NO_4018 RANDOM_LENGTH 84 SEQ_LENGTH 55 GC_CONTENT 0.545454545454545 BASEPAIR 19 FREE_ENERGY -18.91 LEN_BP_RATIO 2.89473684210526
CCCACAGUGCUUGCUUCCCGGGUGGAGAGUGACACGACCAUUAAUGUUAUGAAAUGGAAGACGGUCUCCACGAUAUUCCUGGUG
>random_seq_from_cds__NO_4032 RANDOM_LENGTH 83 SEQ_LENGTH 70 GC_CONTENT 0.457142857142857 BASEPAIR 24 FREE_ENERGY -23.50 LEN_BP_RATIO 2.91666666666667
AAUAAUCAAUGCCUUGGUCGCUAUAUUGGUAAUCUCUGGGGUAGUCAUCCUGGUACUCGCCAUGAUACUCAUCAGGGUAUUCU
>random_seq_from_cds__NO_4035 RANDOM_LENGTH 110 SEQ_LENGTH 82 GC_CONTENT 0.524390243902439 BASEPAIR 30 FREE_ENERGY -27.80 LEN_BP_RATIO 2.73333333333333
AAAUUGUCGAGUUGUCCACCGGCUGUAAUGUUAGCUUCUGAUCAUAGAUGUCUCUUCUGGUACCUGGUGCUAACAUCCCUGCCCUGGCUGGCUCCUUUAUUAGUGCCCAU
>random_seq_from_cds__NO_4058 RANDOM_LENGTH 82 SEQ_LENGTH 73 GC_CONTENT 0.438356164383562 BASEPAIR 26 FREE_ENERGY -22.30 LEN_BP_RATIO 2.80769230769231
AUAUGCAGGAUACAUUACCUGUUUCACUAGUAGGACGAAUGGCAAUUGGUUCUGCCAGCUCGGUU

UUGCCAGAUCUUGUAAC
>random_seq_from_cds__NO_4059 RANDOM_LENGTH 82 SEQ_LENGTH 67 GC_CONTENT 0.492537313432836 BASEPAIR 21 FREE_ENERGY -16.50 LEN_BP_RATIO 3.19047619047619
CCAAGCAACCCUCUGGGGCAAAGUCAGCAACAUGAGUCUUCUCUUUCUCUAAUGCACUUUGAGACACAAACAUGGGGAAGUA
>random_seq_from_cds__NO_4071 RANDOM_LENGTH 90 SEQ_LENGTH 82 GC_CONTENT 0.402439024390244 BASEPAIR 26 FREE_ENERGY -19.96 LEN_BP_RATIO 3.15384615384615
AGCCACUCUAUUGUAAAGGACCAAGGAAUCCUCAGAUGUAGUACAAUUAUUAUUCAGAGAAGGUGUUGGUCUUUCCUUAAAAGGAGCAGA
>random_seq_from_cds__NO_4084 RANDOM_LENGTH 84 SEQ_LENGTH 71 GC_CONTENT 0.464788732394366 BASEPAIR 22 FREE_ENERGY -17.23 LEN_BP_RATIO 3.22727272727273
AAACUGCUCAUCUCUGUCAUGGUAUUCUGUUGUUCUCAGGGCAUGUGUAACACCUUCGAUGCUGUCAACUAUGGGGCAGGCAAA
>random_seq_from_cds__NO_4085 RANDOM_LENGTH 95 SEQ_LENGTH 76 GC_CONTENT 0.407894736842105 BASEPAIR 24 FREE_ENERGY -19.60 LEN_BP_RATIO 3.16666666666667
AUCAUAUGUUGGAUAAACACUUGUAUUUAUUUCCAGUUCUUGGAUGUGGUUGAAUUUUGCAGCGAUAAAGGGUUGGAUCUCUCAUGCAUCCAUUG
>random_seq_from_cds__NO_4102 RANDOM_LENGTH 89 SEQ_LENGTH 75 GC_CONTENT 0.56 BASEPAIR 29 FREE_ENERGY -28.80 LEN_BP_RATIO 2.58620689655172
CUGAUAGCCGACUGGCCGGUGGUGGUCUUGGGCAUGUGCACCAUGUUCAUCGUAGUCUGUGCCUUGGUUGGAGUAUUAGUGCCAGAGCU
>random_seq_from_cds__NO_4111 RANDOM_LENGTH 81 SEQ_LENGTH 68 GC_CONTENT 0.455882352941176 BASEPAIR 21 FREE_ENERGY -22.51 LEN_BP_RATIO 3.23809523809524
CACGCCAGCUUUAAAAUACAGCAUGCUCUUCUCUCCCACAGAGAAAGGGGAGAGCAUGAUGAACAUUUACUUGGACAACUU
>random_seq_from_cds__NO_4140 RANDOM_LENGTH 94 SEQ_LENGTH 85 GC_CONTENT 0.541176470588235 BASEPAIR 27 FREE_ENERGY -18.30 LEN_BP_RATIO 3.14814814814815
CUUGAACUAUGGCCCACACUCUUGCCAGCAGAUGGGGGACUGCUUGUGCCACCAGUGCUCUCCUACCACUAGCAGCUUUGUCCAGAUCCAAAAC
>random_seq_from_cds__NO_4142 RANDOM_LENGTH 72 SEQ_LENGTH 66 GC_CONTENT 0.53030303030303 BASEPAIR 21 FREE_ENERGY -20.10 LEN_BP_RATIO 3.14285714285714
UGCAGGGCAGAGUAAAGCCAGCCGGAAUGCAGAAUUCUCUGCCUAGGAAUUUUUUCCUCCACCCAGUGCAGC
>random_seq_from_cds__NO_4155 RANDOM_LENGTH 77 SEQ_LENGTH 58 GC_CONTENT 0.603448275862069 BASEPAIR 22 FREE_ENERGY -24.40 LEN_BP_RATIO 2.63636363636364

UAUGAAGCGCUAUCAGGGGGUGCCACCACUGAGGGCUUCGAAGACUUCACCGGAGGCAUUGCUGA
GUGGUAUGAGUU
>random_seq_from_cds__NO_4167 RANDOM_LENGTH 67 SEQ_LENGTH 58
GC_CONTENT 0.517241379310345 BASEPAIR 22 FREE_ENERGY
-16.60 LEN_BP_RATIO 2.63636363636364
AGAUCUCUGCCUUUGAGCUGCAGACCAUCCUGAGAAGGGUUCUAGCAAAGCGGCCAAGAUAUCAA
GU
>random_seq_from_cds__NO_4168 RANDOM_LENGTH 86 SEQ_LENGTH 79
GC_CONTENT 0.506329113924051 BASEPAIR 30 FREE_ENERGY
-25.80 LEN_BP_RATIO 2.63333333333333
CAGAUGGCUUCAGCAUCGAGACAUGCAAAAUUAUGGUUGACAUGCUAGAUGUCGGACGGGAGUGG
CAAGCUGGGGCUGAAGGAGUU
>random_seq_from_cds__NO_4172 RANDOM_LENGTH 110 SEQ_LENGTH 93
GC_CONTENT 0.56989247311828 BASEPAIR 33 FREE_ENERGY
-40.50 LEN_BP_RATIO 2.81818181818182
AUCCUCCUCACUUCAGUGCUGGGCUUUGCCAUCUACUGGUUCAUCUCCCGGGACAAAGAGGAAAC
UUUGCCACUUGAAGAUGGGUGGUGGGGGCCAGGCACGAGGUCCGC
>random_seq_from_cds__NO_4187 RANDOM_LENGTH 84 SEQ_LENGTH 71
GC_CONTENT 0.633802816901408 BASEPAIR 27 FREE_ENERGY
-31.80 LEN_BP_RATIO 2.62962962962963
UGUCGGUGCUGGAGCGGCAAUGACCUAUGGCUGGAGCCUCCUUGGCACGAGCGCACCAUCCGAGG
CACAGCUGCACUUCUGCAC
>random_seq_from_cds__NO_4191 RANDOM_LENGTH 80 SEQ_LENGTH 68
GC_CONTENT 0.647058823529412 BASEPAIR 21 FREE_ENERGY
-27.70 LEN_BP_RATIO 3.23809523809524
GCAGCGGGUGCCCUCGGUCAUUGGUGCUUCAGGGUCACAGUCGCCCUGAGCUCCCAUAGUCCCCA
AGGUCCAGGGUGUGC
>random_seq_from_cds__NO_4207 RANDOM_LENGTH 94 SEQ_LENGTH 87
GC_CONTENT 0.793103448275862 BASEPAIR 33 FREE_ENERGY
-47.00 LEN_BP_RATIO 2.63636363636364
GGGAGGUGCGGUUGGAGCCGUCGUCGCGGACGCGCAGCCACUCGACGGUCACCCGGGCCUGGGCG
CUGCGCGGGGACAGCCGCCCGUGCCUGUG
>random_seq_from_cds__NO_4209 RANDOM_LENGTH 84 SEQ_LENGTH 70
GC_CONTENT 0.671428571428571 BASEPAIR 26 FREE_ENERGY
-26.50 LEN_BP_RATIO 2.69230769230769
CAUGCCGAACACCAGCAGGUGUGUGCUGGCCUCCGACGCCAGGAACCUGCCGGCCACCUCCUCGG
AAGCUCUGGCUGAACCUCU
>random_seq_from_cds__NO_4216 RANDOM_LENGTH 86 SEQ_LENGTH 76
GC_CONTENT 0.513157894736842 BASEPAIR 28 FREE_ENERGY
-24.60 LEN_BP_RATIO 2.71428571428571
UUUGCAGGGAGGCAAGUGAGGCCUAUCUGGUUGGCCUUUUUGAAGACACCAACCUGUGUGCUAUC
CAUGCCAAACGUGUAACAAUU
>random_seq_from_cds__NO_4233 RANDOM_LENGTH 89 SEQ_LENGTH 74
GC_CONTENT 0.851351351351351 BASEPAIR 28 FREE_ENERGY

-49.00 LEN_BP_RATIO 2.64285714285714
CUCCGCCGCGCCCCCAGCCGCCGCCUCCCCGGGCUCGGGCUGCUCCCCUGAGGCGCCCGGGCCGC
GACCGGAUCCAGGUGGCGAGGGCG
>random_seq_from_cds__NO_4243 RANDOM_LENGTH 82 SEQ_LENGTH 68
GC_CONTENT 0.544117647058823 BASEPAIR 25 FREE_ENERGY
-25.90 LEN_BP_RATIO 2.72
CUCAUAGUUGACAUCGAUGGGAUCCUUGCUGCUAUCAUCAGACCCUCCCCUGAGCAGACUGUAGG
CCACCUCGAUGUCCAGC
>random_seq_from_cds__NO_4251 RANDOM_LENGTH 110 SEQ_LENGTH 90
GC_CONTENT 0.555555555555556 BASEPAIR 32 FREE_ENERGY
-32.70 LEN_BP_RATIO 2.8125
UUGUAGUAGGAGUUGGUUCCUUUAACGAUGUCCACCAGGCCAAGGGUGGCACUGAAGACCUUCCC
ACCUUUCUCCAGGACAUGCGCAGAGUGUUCCAGUCCCAGAAUCAG
>random_seq_from_cds__NO_4267 RANDOM_LENGTH 110 SEQ_LENGTH 75
GC_CONTENT 0.586666666666667 BASEPAIR 20 FREE_ENERGY
-18.24 LEN_BP_RATIO 3.75
GCCUUGCUACCAAUUCCAUCCUGGCCUUUGCCCUGUCACUCCUCCAGCUUCCGCUGUCUUCUUGA
CUUUCUGCUGGUCAUCCCACCGAAGCUCAGAGAACCCAUCCACCU
>random_seq_from_cds__NO_4274 RANDOM_LENGTH 82 SEQ_LENGTH 66
GC_CONTENT 0.545454545454545 BASEPAIR 22 FREE_ENERGY
-20.00 LEN_BP_RATIO 3
CUUCAUUGGUGGUGCUGCCCACCUUGAGUGCCGUCUCAUACUUGUGCUUCAGAUGCUUGCCCACC
UCAUGGAAAGGCGCCAA
>random_seq_from_cds__NO_4275 RANDOM_LENGTH 73 SEQ_LENGTH 52
GC_CONTENT 0.692307692307692 BASEPAIR 22 FREE_ENERGY
-29.70 LEN_BP_RATIO 2.36363636363636
CUGCCGCCAGCAGGUCCGCACCGUGCAUGAGCCUGACACGCCGUGGCACUUGCAGGUGGUCUCCA
CCCCAGCC
>random_seq_from_cds__NO_4284 RANDOM_LENGTH 88 SEQ_LENGTH 73
GC_CONTENT 0.698630136986301 BASEPAIR 28 FREE_ENERGY
-34.60 LEN_BP_RATIO 2.60714285714286
ACCCGAUCUGGUGGGUCGCUGGCUGUUGGGCCACAGUAUUCCUCCCUGGGCUCGCAGCCCAUCCU
GUGUGCCAGCAUCCCGGGCCUGG
>random_seq_from_cds__NO_4287 RANDOM_LENGTH 85 SEQ_LENGTH 68
GC_CONTENT 0.676470588235294 BASEPAIR 25 FREE_ENERGY
-35.00 LEN_BP_RATIO 2.72
UUGCCUCAGCCGGUGUGGCCUUUGCAGUGACACGCUCAUGUGCAGAAGGCACGGCCGCCAUCUGU
GGCUGCAGCAGCCGCCACCA
>random_seq_from_cds__NO_4297 RANDOM_LENGTH 75 SEQ_LENGTH 48
GC_CONTENT 0.625 BASEPAIR 20 FREE_ENERGY -29.05
LEN_BP_RATIO 2.4
GCCAGGACAAGAUCCGGCCCCUGUGGCGCCACUACUUCCAGAACACACAAGGGCCUGAUCUUCGU
GGUGGACAGC
>random_seq_from_cds__NO_4304 RANDOM_LENGTH 72 SEQ_LENGTH 58

GC_CONTENT 0.706896551724138 BASEPAIR 19 FREE_ENERGY -25.38 LEN_BP_RATIO 3.05263157894737
CGGGGCACUUGGGGACCCUGUCUUUCCGGCUCACGUUGUCCCUGCACACACCCAGGGCCCACAAC
GCGUCCC
>random_seq_from_cds__NO_4306 RANDOM_LENGTH 97 SEQ_LENGTH 85 GC_CONTENT 0.670588235294118 BASEPAIR 31 FREE_ENERGY -40.80 LEN_BP_RATIO 2.74193548387097
AUCGGUCCUUGCUGCAGAACCCACUGCCCUCCGGCGAAGAGCCGAGGUAGCGCCUCUGGCGGCUC
UCAUACAGGAGGAGGUAGGGUACGCGGAGGU
>random_seq_from_cds__NO_4309 RANDOM_LENGTH 110 SEQ_LENGTH 70 GC_CONTENT 0.7 BASEPAIR 29 FREE_ENERGY -47.30 LEN_BP_RATIO 2.41379310344828
UCCGCUCCUCCAGCUGCAGCAGCAGCAGCUCCAGAGAGUGACCCUGCCGGUCCAGGCAGGCCACG
CUCUCCCGGAGCCUGCUGGCAGUCUCCUCUUCUUCCGUCUCCAGA
>random_seq_from_cds__NO_4313 RANDOM_LENGTH 95 SEQ_LENGTH 82 GC_CONTENT 0.658536585365854 BASEPAIR 30 FREE_ENERGY -38.10 LEN_BP_RATIO 2.73333333333333
AAAAGCUUGAGGGGCUCGUGGUGCUCCUGGCACAGGUCUUGCUUCUGCAGACCAGGAUGCUGCUG
CGCCAUCUCGGCCACCUUGGUCAGCAGCCG
>random_seq_from_cds__NO_4316 RANDOM_LENGTH 71 SEQ_LENGTH 61 GC_CONTENT 0.524590163934426 BASEPAIR 23 FREE_ENERGY -16.70 LEN_BP_RATIO 2.65217391304348
GCUUCCUCCUGCAGUUUUCUGGCGAGUUCCACAGCCUCCAUUCUAGAAGCAUUUGCGGUGGACGA
UGGAAG
>random_seq_from_cds__NO_4322 RANDOM_LENGTH 77 SEQ_LENGTH 66 GC_CONTENT 0.621212121212121 BASEPAIR 21 FREE_ENERGY -16.60 LEN_BP_RATIO 3.14285714285714
CGCCAUCUCGUUCUCGAAGUCCAGGGCCACGUAGCACAGCUUCUCCUUGAUGUCGCGCACGAUCU
CGCGCUCAGCCU
>random_seq_from_cds__NO_4333 RANDOM_LENGTH 78 SEQ_LENGTH 55 GC_CONTENT 0.4 BASEPAIR 21 FREE_ENERGY -17.11 LEN_BP_RATIO 2.61904761904762
AUCAUCUUUGCCAUCAGAACUGGACCAGCUUAUCUCUCUGAAGAGCUUUGCAAAGGAUUUCCAGU
UUUAGAUCAUUUA
>random_seq_from_cds__NO_4337 RANDOM_LENGTH 95 SEQ_LENGTH 76 GC_CONTENT 0.421052631578947 BASEPAIR 27 FREE_ENERGY -22.29 LEN_BP_RATIO 2.81481481481481
UCAAGCCAAGAAGGGUUUUCUUCUUUGCAAAGUAACGAGUUUCCAUAUUUGCCAAACCCAGAAGU
GUUGCAUGAGCCCUUUUCUAAUUCUUGGCU
>random_seq_from_cds__NO_4348 RANDOM_LENGTH 73 SEQ_LENGTH 67 GC_CONTENT 0.447761194029851 BASEPAIR 24 FREE_ENERGY -20.90 LEN_BP_RATIO 2.79166666666667
UUGUUCAAAGCAAUCAAUAUGGCUGUGUUGACAAGGUCAGAAAGCCGGGAGUGGUGGUUCUUGAG
AACAAUGG

>random_seq_from_cds__NO_4359 RANDOM_LENGTH 87 SEQ_LENGTH 76 GC_CONTENT 0.5 BASEPAIR 24 FREE_ENERGY -18.40 LEN_BP_RATIO 3.16666666666667
GAUGAGACAUGGAUUGUCUGCUGAGUGCCAUGCUGCUGCCAAAAUCACCAGCCCAUCACCAGUUU
UGCUUCAAGUCCAAAUAUCGAA
>random_seq_from_cds__NO_4367 RANDOM_LENGTH 97 SEQ_LENGTH 85 GC_CONTENT 0.482352941176471 BASEPAIR 26 FREE_ENERGY -16.40 LEN_BP_RATIO 3.26923076923077
CCAAAUAAUGAGCUUCUCUUUGCACACCAGACAAGCCCAUCCACCUUCAUCUAUGUUAAUGGUCA
GCUGGUCAUCGACCUUCAGCCAAUGUUAGGGC
>random_seq_from_cds__NO_4391 RANDOM_LENGTH 90 SEQ_LENGTH 83 GC_CONTENT 0.843373493975904 BASEPAIR 29 FREE_ENERGY -45.20 LEN_BP_RATIO 2.86206896551724
CAGCCCCAGGAGCUUCCGGGCCUCCGGGAGUCCGGCCGCUGCGGGGCGCAGCCGCCCCUUGUCCC
CGGGAGGCGCCGCCGGCCCGCGCCG
>random_seq_from_cds__NO_4400 RANDOM_LENGTH 90 SEQ_LENGTH 84 GC_CONTENT 0.666666666666667 BASEPAIR 28 FREE_ENERGY -32.90 LEN_BP_RATIO 3
CCGUUGGGAGAAAAGGCGAGAGAAAGCACGGGGCCACGGUGGCCUGUGAAAAGCCUCACCGAGUU
CCCCUGCUGAGCGCUCCACAGCCGG
>random_seq_from_cds__NO_4402 RANDOM_LENGTH 75 SEQ_LENGTH 70 GC_CONTENT 0.6 BASEPAIR 27 FREE_ENERGY -29.00 LEN_BP_RATIO 2.59259259259259
UAUAUCCUCAGCGGGUACGUCCGAUCAAAUGACCACAGCCUGGCGGUGCGGUCGUGGGACCCGCU
GGCGAAGUAC
>random_seq_from_cds__NO_4410 RANDOM_LENGTH 86 SEQ_LENGTH 76 GC_CONTENT 0.473684210526316 BASEPAIR 25 FREE_ENERGY -23.50 LEN_BP_RATIO 3.04
CACAGGGCAGUAUUGUUGUCACUUUGGAGGUAGCGGAUAAGGUAGUUGUAGCUGUCUUCUUGGAG
ACGGACCACGUACUUGUUAUC
>random_seq_from_cds__NO_4411 RANDOM_LENGTH 69 SEQ_LENGTH 59 GC_CONTENT 0.491525423728814 BASEPAIR 20 FREE_ENERGY -15.10 LEN_BP_RATIO 2.95
UAGGAAUGCUCGAAGCUUGAAGUUAGAUAGGAUGUCCUGGAUGGUUUGAGUGGUCUGUAGCUGCU
CAAU
>random_seq_from_cds__NO_4412 RANDOM_LENGTH 110 SEQ_LENGTH 87 GC_CONTENT 0.494252873563218 BASEPAIR 33 FREE_ENERGY -25.30 LEN_BP_RATIO 2.63636363636364
GACAUCCUUCUGGCUAGCAUUCUGCAGAAACAUUCCAUGGAAGCGGCUGUAAAAACUUUCCACUG
UGCUCUUCGGACUGUUUUGGACCAGGUUGAGAUGGAGGUAGACAA
>random_seq_from_cds__NO_4414 RANDOM_LENGTH 81 SEQ_LENGTH 66 GC_CONTENT 0.53030303030303 BASEPAIR 22 FREE_ENERGY -20.90 LEN_BP_RATIO 3
CAGUCGUCCAAACUGUACUUCAUAUUGCUGGGGUUCUGCCUGGCAAGGGGCUGCAGACACUAUGU

UGGCACAACCAGAUUC
>random_seq_from_cds__NO_4416 RANDOM_LENGTH 87 SEQ_LENGTH 63 GC_CONTENT 0.587301587301587 BASEPAIR 25 FREE_ENERGY -26.60 LEN_BP_RATIO 2.52
CCAUCUGAGUCCACGUACUGCCGGCGUUUGAGGUAGCAGGACACUGCCAUCUGAAUCUGCUCGGUACGCACUCGUUUCAUAAUGCGG
>random_seq_from_cds__NO_4423 RANDOM_LENGTH 94 SEQ_LENGTH 79 GC_CONTENT 0.531645569620253 BASEPAIR 29 FREE_ENERGY -28.20 LEN_BP_RATIO 2.72413793103448
UGAUCGCUGAGGACGGGGCUCUCUUGGGGAAAAUUGAGAAAGUGCGAGUUCUUAGAAAUGAUCGACGAGAAGGGCCUCAUGCGCUCACGGGUUC
>random_seq_from_cds__NO_4432 RANDOM_LENGTH 95 SEQ_LENGTH 81 GC_CONTENT 0.518518518518518 BASEPAIR 21 FREE_ENERGY -19.54 LEN_BP_RATIO 3.85714285714286
UUGCUGAUGGUGUGGUUGGAGUUUAUGAAUGUCACAAUGCUGGGGGAAACCAGGGAAUGGGCCUUGACGAAGGAGAAGUCGGUGAAGCACAUGGA
>random_seq_from_cds__NO_4434 RANDOM_LENGTH 89 SEQ_LENGTH 78 GC_CONTENT 0.666666666666667 BASEPAIR 22 FREE_ENERGY -26.94 LEN_BP_RATIO 3.54545454545455
AAAUGGGAACAGAUCGAGGGCAACUCCAAGCUGAGGCACGUGGGCAGCAACCUGUGCCUGGACAGUCGCACGGCCAAGAGCGGGGGCCU
>random_seq_from_cds__NO_4457 RANDOM_LENGTH 97 SEQ_LENGTH 84 GC_CONTENT 0.607142857142857 BASEPAIR 28 FREE_ENERGY -36.40 LEN_BP_RATIO 3
GCUGAUUGUGCUCAGGUAUGCGGAUGAGGAGCUCCAGCUGGACUUCGAUGACUUCCUCAACUGCCUGGUCCGGCUGGAGAAUGCGAGCCGGGGUGUU
>random_seq_from_cds__NO_4462 RANDOM_LENGTH 87 SEQ_LENGTH 76 GC_CONTENT 0.421052631578947 BASEPAIR 26 FREE_ENERGY -18.80 LEN_BP_RATIO 2.92307692307692
AGGUGCUCCUGAUAUGGAAGAUAUAUUGACUGAAUCAGAAAUUAAAUUGGAUGGUGUCAGACAAAAGAUAUUCCAGGUAGCCCAAGA
>random_seq_from_cds__NO_4470 RANDOM_LENGTH 84 SEQ_LENGTH 78 GC_CONTENT 0.615384615384615 BASEPAIR 26 FREE_ENERGY -31.50 LEN_BP_RATIO 3
GCUUCCUGGUGCUGGGCUACUUGCUCUACCUGGUCUUCGGCGCAGUGGUCUUCUCCUCGGUGGAGCUGCCCUAUGAGGACCUGC
>random_seq_from_cds__NO_4472 RANDOM_LENGTH 72 SEQ_LENGTH 55 GC_CONTENT 0.672727272727273 BASEPAIR 19 FREE_ENERGY -21.80 LEN_BP_RATIO 2.89473684210526
GGCGUGUCGGUGCUCAGCAACGCCUCGGGCAACUGGAACUGGGACUUCACCUCCGCGCUCUUCUUCGCCAGC
>random_seq_from_cds__NO_4474 RANDOM_LENGTH 81 SEQ_LENGTH 55 GC_CONTENT 0.654545454545455 BASEPAIR 20 FREE_ENERGY -22.00 LEN_BP_RATIO 2.75

UCACCCUCCUGUUCCUGACGGCUGUGGUCCAGCGCAUCACCGUGCACGUCACCCGCAGGCCGGUC
CUCUACUUCCACAUCC
>random_seq_from_cds__NO_4503 RANDOM_LENGTH 81 SEQ_LENGTH 55
GC_CONTENT 0.454545454545455 BASEPAIR 20 FREE_ENERGY
-18.10 LEN_BP_RATIO 2.75
UGAUCAGAAGAAAGAACUGUGAGGGCUUCUAGUGCAGACUGCAAAGUCCUUAUUGGCAUCUGAAC
GGCAGGUAGAAACGGU
>random_seq_from_cds__NO_4504 RANDOM_LENGTH 88 SEQ_LENGTH 57
GC_CONTENT 0.421052631578947 BASEPAIR 21 FREE_ENERGY
-23.40 LEN_BP_RATIO 2.71428571428571
UCCAGAAUUUCACUCCCUGUGGUUGGUAUAAGGGUGUGAUAUUUUUCAACAGGAAAGAGAGGCA
CACCCAUUGAUCAUGAAUAUAUU
>random_seq_from_cds__NO_4527 RANDOM_LENGTH 71 SEQ_LENGTH 63
GC_CONTENT 0.73015873015873 BASEPAIR 21 FREE_ENERGY
-24.79 LEN_BP_RATIO 3
CCGCCGACACCUCCACCGCCCUCUGCAGCAGGUAGCGCGCUCGCUUGCGCGUCAGGGCGUCCGCC
UGGCCC
>random_seq_from_cds__NO_4532 RANDOM_LENGTH 89 SEQ_LENGTH 70
GC_CONTENT 0.8 BASEPAIR 29 FREE_ENERGY -44.90 LEN_BP_RATIO
2.41379310344828
GCCCGUCCUCGCGGGGCCGCAAACAUGGCCCGACGGCUGCUAGCACUUCCACGGCAGCCUCGGCG
CCAGGCGCGCGCCACCCGGCGAGC
>random_seq_from_cds__NO_4537 RANDOM_LENGTH 81 SEQ_LENGTH 58
GC_CONTENT 0.448275862068966 BASEPAIR 21 FREE_ENERGY
-20.40 LEN_BP_RATIO 2.76190476190476
ACCCACUCCAUAAUGAGUGACACUUUGACAGCGGAUGUCAUUGGUCGAAGAGUUGAAGUUAAUGG
AGAACAUGCAACAGUA
>random_seq_from_cds__NO_4553 RANDOM_LENGTH 110 SEQ_LENGTH 104
GC_CONTENT 0.509615384615385 BASEPAIR 33 FREE_ENERGY
-29.00 LEN_BP_RATIO 3.15151515151515
UGCCGGGGCUCCAUGACAAUUCAAAAGGUGAAGGGAUUGCUGUCACGUCUUCUCAAAGUUCCUGU
GUCAGACCUUCUGUUGUCCUAUGAAAGUCCCAAAGAAGCCGGGCA
>random_seq_from_cds__NO_4557 RANDOM_LENGTH 86 SEQ_LENGTH 79
GC_CONTENT 0.518987341772152 BASEPAIR 27 FREE_ENERGY
-17.90 LEN_BP_RATIO 2.92592592592593
CUCCCACCUAAGGAAAGCCGGCACCCAGAUUGAGAACAUCGAGGAAGACUUCAGGAAUGGCCUUA
AGCUCAUGCUGCUUUUGGAAG
>random_seq_from_cds__NO_4563 RANDOM_LENGTH 78 SEQ_LENGTH 72
GC_CONTENT 0.555555555555556 BASEPAIR 24 FREE_ENERGY
-17.00 LEN_BP_RATIO 3
UCGUGAACACCCCUAAACCCGAUGAAAGAGCCAUCAUGACGUACGUCUCUUGCUUCUACCACGCU
UUUGCGGGCGCGG
>random_seq_from_cds__NO_4567 RANDOM_LENGTH 72 SEQ_LENGTH 61
GC_CONTENT 0.60655737704918 BASEPAIR 25 FREE_ENERGY

-32.90 LEN_BP_RATIO 2.44
CUGCGGAUCAGCAACCGUCCUGCCUUCAUGCCCUCCGAGGGCAAGAUGGUGUCGGGAUAUUGCUG
GUGCCUG
>random_seq_from_cds__NO_4581 RANDOM_LENGTH 75 SEQ_LENGTH 55
GC_CONTENT 0.490909090909091 BASEPAIR 19 FREE_ENERGY
-19.50 LEN_BP_RATIO 2.89473684210526
GAUGAAUGAGUUCAGAGCCUCCUUCAACCACUUUGACAGGGAGGAAGAAUGGCCUGAUGGAUCAU
GAGGAUUUCA
>random_seq_from_cds__NO_4582 RANDOM_LENGTH 87 SEQ_LENGTH 79
GC_CONTENT 0.556962025316456 BASEPAIR 32 FREE_ENERGY
-38.70 LEN_BP_RATIO 2.46875
GAGCCUGCCUGAUUUCCAUGGGUUAUGACCUGGGGUGAAGCCGAAUUUGCCCGCAUUAUGACCCU
GGUAGAUCCCAACGGGCAAGGC
>random_seq_from_cds__NO_4588 RANDOM_LENGTH 98 SEQ_LENGTH 74
GC_CONTENT 0.418918918918919 BASEPAIR 26 FREE_ENERGY
-18.90 LEN_BP_RATIO 2.84615384615385
CUGAAAGGCAACAAUGACAUUUUAAGUAUAACUCAGCCUGAUGUCAUUUACCAAAUCCAUAAGGG
AAUACUUGCUGGCUGGGGCAGAUAUCAUUGAAA
>random_seq_from_cds__NO_4593 RANDOM_LENGTH 73 SEQ_LENGTH 55
GC_CONTENT 0.527272727272727 BASEPAIR 21 FREE_ENERGY
-17.60 LEN_BP_RATIO 2.61904761904762
UGCCAAUGCCAAGGGCAGCCUUGUUUGCACUCCAAAAUCUUUUUGAGGAGAAAUAUGCUCCCCGG
CCUAUCUU
>random_seq_from_cds__NO_4594 RANDOM_LENGTH 84 SEQ_LENGTH 64
GC_CONTENT 0.53125 BASEPAIR 27 FREE_ENERGY -28.91
LEN_BP_RATIO 2.37037037037037
UGAUUUCAGGGACGAUCGUUGAUAAAAGUGGGCGGACUCUUUCCGGACAGACAGGAGAGGGAUUU
GUCAUCAGCGUGUCUCAUG
>random_seq_from_cds__NO_4597 RANDOM_LENGTH 78 SEQ_LENGTH 71
GC_CONTENT 0.464788732394366 BASEPAIR 22 FREE_ENERGY
-21.29 LEN_BP_RATIO 3.22727272727273
UGAUGGCCAAGCACCUAAAGGGAUUUUGCUAUGGAUGGCUUGGUCAAUAUAGUUGGAGGAUGCUG
UGGGUCAACACCA
>random_seq_from_cds__NO_4602 RANDOM_LENGTH 86 SEQ_LENGTH 81
GC_CONTENT 0.45679012345679 BASEPAIR 26 FREE_ENERGY
-19.40 LEN_BP_RATIO 3.11538461538462
CCAAUUUUGCUGUGAUUGAAGCUGGGUUAAAGUGCUGCCAAGGGAAGUGCAUUGUCAAUAGCAUU
AGUCUGAAGGAAGGAGAGGAC
>random_seq_from_cds__NO_4607 RANDOM_LENGTH 89 SEQ_LENGTH 78
GC_CONTENT 0.461538461538462 BASEPAIR 28 FREE_ENERGY
-26.60 LEN_BP_RATIO 2.78571428571429
AGAAGCAAUGCAUGGGGUUUUCCUUUACCAUGCAAUCAAGGUCUGGCAUGGACAUGGGGAUAGUG
AAUGCUGGAAACCUCCCUGUGUAU
>random_seq_from_cds__NO_4612 RANDOM_LENGTH 70 SEQ_LENGTH 54

GC_CONTENT 0.5 BASEPAIR 20 FREE_ENERGY -15.20 LEN_BP_RATIO 2.7
GCUGUUGGCCACCUUAUCCCUUUCAUGGAAAAAGAAAGAGAAGAAACCAGAGUGCUUAACGGCACAGUAG
>random_seq_from_cds__NO_4633 RANDOM_LENGTH 87 SEQ_LENGTH 79 GC_CONTENT 0.594936708860759 BASEPAIR 29 FREE_ENERGY -32.10 LEN_BP_RATIO 2.72413793103448
CUGGCAGACUGACACGCUGCAGUGCAGAUGCACCCGGUCCCCUGAGGGCCUGUUUCUCCACUGUAGGGUUCACAAAGCUGAAGGUGA
>random_seq_from_cds__NO_4634 RANDOM_LENGTH 69 SEQ_LENGTH 56 GC_CONTENT 0.589285714285714 BASEPAIR 19 FREE_ENERGY -21.30 LEN_BP_RATIO 2.94736842105263
AGAUGCUGAAGCGCUGGUGGUGAGAGGGAAAUGGAAGAUCCAAGGCUUUCUGGACAGGGAUCAGCUGGG
>random_seq_from_cds__NO_4637 RANDOM_LENGTH 109 SEQ_LENGTH 100 GC_CONTENT 0.52 BASEPAIR 36 FREE_ENERGY -32.80 LEN_BP_RATIO 2.77777777777778
GGUAGUCACCAACACCGUAGUAAGAGCCAUAGUUUUUAUCCUUUGGCAAUCUGAAGUUCCAGAGUGAGGGGUCCAGGCUGGGUCUCAGGAAAGGGUGGUGGGAGAGUGA
>random_seq_from_cds__NO_4664 RANDOM_LENGTH 68 SEQ_LENGTH 48 GC_CONTENT 0.416666666666667 BASEPAIR 19 FREE_ENERGY -16.00 LEN_BP_RATIO 2.52631578947368
AGCUUUAAUAACUGGGGUCUGGCAUGCGUUCUGUCACACCUCCAAUCUUAAAGUUCAUCGUAGAUCUC
>random_seq_from_cds__NO_4672 RANDOM_LENGTH 110 SEQ_LENGTH 96 GC_CONTENT 0.625 BASEPAIR 32 FREE_ENERGY -43.20 LEN_BP_RATIO 3
AGCCCCGGGGCAAAGAGCCUGAUGAGACUGUGCACAGUUUUGCCCAGCGCCGCCUUGGACCUGAGGGUGGCGUCUCUAGCCAUGGACAGUCUCUGCCGUGGAGUGUUUGC
>random_seq_from_cds__NO_4678 RANDOM_LENGTH 78 SEQ_LENGTH 66 GC_CONTENT 0.515151515151515 BASEPAIR 24 FREE_ENERGY -21.80 LEN_BP_RATIO 2.75
CCAUCUGCCUGUCCAGGGGAUUUGGACAUUUGGUGCCAUCUUCAGAAGAUCCAGGAGUCCUGGGAAUCGUGUAUGACU
>random_seq_from_cds__NO_4694 RANDOM_LENGTH 75 SEQ_LENGTH 70 GC_CONTENT 0.471428571428571 BASEPAIR 25 FREE_ENERGY -17.60 LEN_BP_RATIO 2.8
CAAGCCAGACAGGAGCUUGGAUCUAGGAGGCAGUAUGGUAAUAAAAUUUGUGGAUGCGCUUCAGGAGCGUGGUUU
>random_seq_from_cds__NO_4699 RANDOM_LENGTH 84 SEQ_LENGTH 66 GC_CONTENT 0.515151515151515 BASEPAIR 21 FREE_ENERGY -16.60 LEN_BP_RATIO 3.14285714285714
UGGGUGGCGUUGGUAGGAUGGAUCAGAAUAUUGCCAAGUACAAGGUGAAGAUCCGAGGCAUGAAGUGGUACUCAAGCUUUAUUG

>random_seq_from_cds__NO_4707 RANDOM_LENGTH 84 SEQ_LENGTH 66 GC_CONTENT 0.575757575757576 BASEPAIR 19 FREE_ENERGY -19.63 LEN_BP_RATIO 3.47368421052632
CUUUCUCCUGCGUGUCCCUGCUUCUCCUCGCCGGUCUCCUCCGCAGGGUUCUCUUCUUCUUGUUU
CUGGGACAUGUUAGCCGUC
>random_seq_from_cds__NO_4708 RANDOM_LENGTH 102 SEQ_LENGTH 78 GC_CONTENT 0.474358974358974 BASEPAIR 24 FREE_ENERGY -19.30 LEN_BP_RATIO 3.25
UUCUUUUGGGGGUGUGUACCAGCCCGUUUUUUUCGUGGAUCUGCACCAAGGACUUGUAGGACUGC
UGUGCUCUUGUCAGACUGUAUUGAGACUUUGUUGGCU
>random_seq_from_cds__NO_4717 RANDOM_LENGTH 84 SEQ_LENGTH 74 GC_CONTENT 0.608108108108108 BASEPAIR 26 FREE_ENERGY -26.10 LEN_BP_RATIO 2.84615384615385
GGGUCAUCAGACUCCACCGACCAGAUGGGGGGCACAGCAGGGUAUGACUCCCGUGAUGUUGCAGU
GGAUGCGGACAGGAUCCCC
>random_seq_from_cds__NO_4719 RANDOM_LENGTH 81 SEQ_LENGTH 61 GC_CONTENT 0.704918032786885 BASEPAIR 20 FREE_ENERGY -28.10 LEN_BP_RATIO 3.05
GAACUCGCAGCUCAGCUCGUCCAGGCAGGCGCUGGCAAUGCGGAAGCGCUCGUGGCCGCGGUGGA
AGAUGGACUCGAGCAG
>random_seq_from_cds__NO_4728 RANDOM_LENGTH 89 SEQ_LENGTH 77 GC_CONTENT 0.519480519480519 BASEPAIR 22 FREE_ENERGY -24.43 LEN_BP_RATIO 3.5
CAAGCCAGGGUCACUGUUCAUGGUCUGAACGAUUUCCAUGCCCCUGGUUGAGCACAACCCAGUUU
GGGUCAAUCUGAGCAUCACCCUCA
>random_seq_from_cds__NO_4757 RANDOM_LENGTH 86 SEQ_LENGTH 72 GC_CONTENT 0.458333333333333 BASEPAIR 25 FREE_ENERGY -15.60 LEN_BP_RATIO 2.88
GAUGAAAAUUUUAAACUUAGACACUCAGCUCCAGGCCUGCUUUCCAUGGGCGAACAGUGGUCCAA
GUACAAAUGGCUGUCAGUUCU
>random_seq_from_cds__NO_4763 RANDOM_LENGTH 87 SEQ_LENGTH 75 GC_CONTENT 0.466666666666667 BASEPAIR 26 FREE_ENERGY -19.11 LEN_BP_RATIO 2.88461538461538
GUGCUGGUGCCUGCUGCUAAAGUUUGUCCUGCUCCCGAAGCACUGGUCACACAACCAACUGAAUU
GCAGAGAGUAAUAGUAAACUCA
>random_seq_from_cds__NO_4768 RANDOM_LENGTH 78 SEQ_LENGTH 69 GC_CONTENT 0.420289855072464 BASEPAIR 28 FREE_ENERGY -18.30 LEN_BP_RATIO 2.46428571428571
AGUGAUGGUUGUCAUUGUUUGAGGAGGUUUUAAUGCAUUUUCAUUGGCCGAUUCUACAAAUGAAU
GAGGACUGAGCCA
>random_seq_from_cds__NO_4770 RANDOM_LENGTH 89 SEQ_LENGTH 53 GC_CONTENT 0.471698113207547 BASEPAIR 19 FREE_ENERGY -15.50 LEN_BP_RATIO 2.78947368421053
UUCAUAUCUUAUAAUUAUUCCCAUUUAGUUCCGCUGGUGGAGACCAUUCUACAUGAAGUUCUGUA

GAACUGAUUUUCUGCAUCUUAGGU
>random_seq_from_cds__NO_4772 RANDOM_LENGTH 75 SEQ_LENGTH 58 GC_CONTENT 0.482758620689655 BASEPAIR 19 FREE_ENERGY -17.80 LEN_BP_RATIO 3.05263157894737
CAGAAAAAUCGUACUUGGCAAAUGGAACCAGAUUCCAGAUGGUAGCUGAGGUUUCAUGACCUUCGUAGGAAACAC
>random_seq_from_cds__NO_4779 RANDOM_LENGTH 94 SEQ_LENGTH 84 GC_CONTENT 0.464285714285714 BASEPAIR 26 FREE_ENERGY -21.97 LEN_BP_RATIO 3.23076923076923
UUACAAGGCUGACAUCCUUCCAGUCUGAGGAUCAAAUCCAAAGUAAUGGUCUUUGCAUUGGUCACAACGUUGCCCAGCAAUGGAAGCAUCUUGG
>random_seq_from_cds__NO_4780 RANDOM_LENGTH 78 SEQ_LENGTH 55 GC_CONTENT 0.509090909090909 BASEPAIR 21 FREE_ENERGY -19.50 LEN_BP_RATIO 2.61904761904762
CAAACACACUGACCAGUCAGGCUAUUACAGAUGUGAUUAACUGCACCAGUUGUAUGGCAUGAGCAUGGCAGGCAGCCA
>random_seq_from_cds__NO_4787 RANDOM_LENGTH 88 SEQ_LENGTH 67 GC_CONTENT 0.432835820895522 BASEPAIR 23 FREE_ENERGY -22.72 LEN_BP_RATIO 2.91304347826087
AAGUCCUUUGGCUUCUUUUUUGCACUCACACUGCCCAGAGUGAGGAUUGCAGAAUUUGUUCACUGAGCCAUGGAGGUUACACUGGCAG
>random_seq_from_cds__NO_4794 RANDOM_LENGTH 81 SEQ_LENGTH 64 GC_CONTENT 0.453125 BASEPAIR 22 FREE_ENERGY -17.70 LEN_BP_RATIO 2.90909090909091
UGGAAUGGCUGUUGCAUUGACAAGGUUUACAAUUGAAAGCGUAAACUUGAUCACCUUGGCGGAAAGGCUUGUCAUUAUAAA
>random_seq_from_cds__NO_4803 RANDOM_LENGTH 90 SEQ_LENGTH 78 GC_CONTENT 0.564102564102564 BASEPAIR 27 FREE_ENERGY -28.70 LEN_BP_RATIO 2.88888888888889
UUCAACCGUGACACUCUAUUAUCAGCUGUGUCUCCUGCAUCAUUAGGAAUGCAGUACCGCUGUGCCAAAGGGUGGACCCGCGGGUGGCUG
>random_seq_from_cds__NO_4838 RANDOM_LENGTH 84 SEQ_LENGTH 72 GC_CONTENT 0.597222222222222 BASEPAIR 25 FREE_ENERGY -26.80 LEN_BP_RATIO 2.88
ACUAAAGGCCUGUGAGGCUGGAAGAGUGACACGCUAUGGGAUUGCAGGUCCUUGGUCACUACCUCCGGCGGCAGCGAGAUCCCA
>random_seq_from_cds__NO_4841 RANDOM_LENGTH 97 SEQ_LENGTH 80 GC_CONTENT 0.6 BASEPAIR 26 FREE_ENERGY -33.00 LEN_BP_RATIO 3.07692307692308
GAUGGGAUUCUUUUUGGGAUGGUGGGGCCUAUGACUGGGGAGGCUCUGUGCUAUGGCUUGAAGGAGGCCACCGCCUUUUCCCCCCACGAAUGGCAC
>random_seq_from_cds__NO_4849 RANDOM_LENGTH 72 SEQ_LENGTH 48 GC_CONTENT 0.5625 BASEPAIR 19 FREE_ENERGY -23.00 LEN_BP_RATIO 2.52631578947368

UCCAUCUGACCCCAUCACUGGAGGUGACCCCACAGGCCAUCAGUGUGGUUCAGAGGGACUGUAGG
CGGCGAG
>random_seq_from_cds__NO_4852 RANDOM_LENGTH 86 SEQ_LENGTH 62
GC_CONTENT 0.548387096774194 BASEPAIR 22 FREE_ENERGY
-18.80 LEN_BP_RATIO 2.81818181818182
CUACACUUCCAUGUGCUGGGAUACAUCAGAUUACCUCCGGCCAGUGGCCUUGACUGUGACCUUUG
CCUUGGACAAUACUACAAAGC
>random_seq_from_cds__NO_4853 RANDOM_LENGTH 88 SEQ_LENGTH 67
GC_CONTENT 0.611940298507463 BASEPAIR 23 FREE_ENERGY
-31.00 LEN_BP_RATIO 2.91304347826087
CAGGGCCUGUGCUGAAUGAGGGCUCACCCACCUCUAUACAAAAGCUGGGUCCCCUUCUCAAAGGA
UUGUGGCCCUGACAAUGAAUGUG
>random_seq_from_cds__NO_4855 RANDOM_LENGTH 110 SEQ_LENGTH 99
GC_CONTENT 0.484848484848485 BASEPAIR 28 FREE_ENERGY
-26.70 LEN_BP_RATIO 3.53571428571429
CGGAAAGUGCUGGUAUCUACAACUCUGGAGAACAGAAAGGAAAAUGCUUACAAUACGAGCCUGAG
UCUCAUCUUCUCUAGAAACCUCCACCUGGCCAGUCUCACUCCUCA
>random_seq_from_cds__NO_4864 RANDOM_LENGTH 98 SEQ_LENGTH 80
GC_CONTENT 0.5875 BASEPAIR 25 FREE_ENERGY -29.70
LEN_BP_RATIO 3.2
CCGAAGAGGGCAGUGUCCUACAGCUGACUGAAGCCUCCCGUUGGAGUGAGGAGCCUCUUGGAGGU
GGUUCAGACCCGGCCUAUCCUCAUCUCCCUGUG
>random_seq_from_cds__NO_4865 RANDOM_LENGTH 68 SEQ_LENGTH 61
GC_CONTENT 0.59016393442623 BASEPAIR 22 FREE_ENERGY
-31.50 LEN_BP_RATIO 2.77272727272727
GAUCCUCAUAGGCAGUGUCCUGGGAGGGUUGCUCCUGCUUGCUCUCCUUGUCUUCUGCCUGUGGA
AGG
>random_seq_from_cds__NO_4881 RANDOM_LENGTH 85 SEQ_LENGTH 60
GC_CONTENT 0.65 BASEPAIR 22 FREE_ENERGY -30.60
LEN_BP_RATIO 2.72727272727273
UCAGCACCCUGGCUCUGGCCAUGGCGGGAGGAAGGCUGGCUUGGUCCUGCCUCAGAGCCCCAUUG
CUCACACUCUUCCUUGGACC
>random_seq_from_cds__NO_4885 RANDOM_LENGTH 70 SEQ_LENGTH 58
GC_CONTENT 0.689655172413793 BASEPAIR 22 FREE_ENERGY
-27.80 LEN_BP_RATIO 2.63636363636364
GGAGCGUGUUGCGGCGAGCCAUGGGGAGGUGGAGCGACUUGAGCGUCAUGGCCUCCAUGCCCCGC
ACCAU
>random_seq_from_cds__NO_4886 RANDOM_LENGTH 65 SEQ_LENGTH 55
GC_CONTENT 0.636363636363636 BASEPAIR 21 FREE_ENERGY
-25.90 LEN_BP_RATIO 2.61904761904762
GCUGUUCAUCACCUCCAGGCUGUGGCGCUUGUUCACAGUGCCGUGGUGCUUGGUGGCCGUGAGGG
>random_seq_from_cds__NO_4888 RANDOM_LENGTH 81 SEQ_LENGTH 67
GC_CONTENT 0.73134328358209 BASEPAIR 26 FREE_ENERGY
-38.90 LEN_BP_RATIO 2.57692307692308

GGGUCACCAGGUCCGGGCUGCUGCCGCUGACGUACUUGUGGCGGUGGCUGGCCAGGUCUGGGGUG
CUGGUGGCAGGUCGUG
>random_seq_from_cds__NO_4891 RANDOM_LENGTH 83 SEQ_LENGTH 65
GC_CONTENT 0.523076923076923 BASEPAIR 21 FREE_ENERGY
-21.60 LEN_BP_RATIO 3.0952380952381
AUUAUUCUUUGGGUUCCUCUGGUCAGAUGGACUGACAAGUUUGUUGCUGUAGACCCCCUGUGGCC
CAUAAGGGACAGUGUAGG
>random_seq_from_cds__NO_4892 RANDOM_LENGTH 81 SEQ_LENGTH 74
GC_CONTENT 0.554054054054054 BASEPAIR 25 FREE_ENERGY
-20.52 LEN_BP_RATIO 2.96
GGUGCCUCUCCCGCAUCUCCGGUUGGCUGUACACCAGAUCCUCUGGCUGGUUGUAGGCAUGGGUG
UUGAUAAUGUUGAGGU
>random_seq_from_cds__NO_4906 RANDOM_LENGTH 70 SEQ_LENGTH 59
GC_CONTENT 0.593220338983051 BASEPAIR 22 FREE_ENERGY
-17.70 LEN_BP_RATIO 2.68181818181818
GGCAUUCUUGCCCUGUGCUUUCCACCGACAGCGUGCACUCGAUAACAUUGCUGUCCAGCAGGCGA
AUCCG
>random_seq_from_cds__NO_4911 RANDOM_LENGTH 80 SEQ_LENGTH 69
GC_CONTENT 0.478260869565217 BASEPAIR 26 FREE_ENERGY
-22.70 LEN_BP_RATIO 2.65384615384615
CAUCAUCAUCAUCGGUGUGGGUUGUCUCUUCAUGAGUUAGUUGCUGUCAUUCUUGAUGACGACCU
CUAAUCCGUGGUGCC
>random_seq_from_cds__NO_4928 RANDOM_LENGTH 80 SEQ_LENGTH 66
GC_CONTENT 0.666666666666667 BASEPAIR 22 FREE_ENERGY
-24.80 LEN_BP_RATIO 3
AACCCAUCGGACUCCCCAUCUCGAUCCCAGGCUGCAGGAUCCAGUAGCAGCUGGGGCACCUGGCC
CAGAGGUUCUGCCAC
>random_seq_from_cds__NO_4931 RANDOM_LENGTH 87 SEQ_LENGTH 75
GC_CONTENT 0.693333333333333 BASEPAIR 28 FREE_ENERGY
-38.00 LEN_BP_RATIO 2.67857142857143
CAGCAGGAUCCCAGCCCUCCUUUCCAGUCUCCCCUUGAGGGGGUGGCCUCUGCUCUCCAGUGGGG
UCCCACCUGGGGGAGGUGGGGG
>random_seq_from_cds__NO_4932 RANDOM_LENGTH 81 SEQ_LENGTH 72
GC_CONTENT 0.597222222222222 BASEPAIR 28 FREE_ENERGY
-28.90 LEN_BP_RATIO 2.57142857142857
ACCUGAGGUAGAAGCCCAGGGGAGUGGAGUCUGGAAGGGUCUCCCCGAGCUCAUCCUCCUCGCUC
UCUUCUCUGCUCUCCU
>random_seq_from_cds__NO_4939 RANDOM_LENGTH 95 SEQ_LENGTH 86
GC_CONTENT 0.686046511627907 BASEPAIR 29 FREE_ENERGY
-29.30 LEN_BP_RATIO 2.96551724137931
GGCCUGGCUCAGCUCCCGCAGCAGACUCACCCAUGGCAGGCUCUGACCCCAACAUGACCUCUGGG
GAGGCUUGGUCACCCCCUGGGGCCACAUCA
>random_seq_from_cds__NO_4950 RANDOM_LENGTH 71 SEQ_LENGTH 53
GC_CONTENT 0.490566037735849 BASEPAIR 19 FREE_ENERGY

-18.60 LEN_BP_RATIO 2.78947368421053
CCCUAGAGACCUCCGUCGCUGUUGAGUCUCUUUUUCAAGAGUUCUCAAUGUCUCUUGGUCCUGUUCUUCUA
>random_seq_from_cds__NO_4957 RANDOM_LENGTH 82 SEQ_LENGTH 72 GC_CONTENT 0.513888888888889 BASEPAIR 23 FREE_ENERGY -27.80 LEN_BP_RATIO 3.1304347826087
UCUCCCUUUCUAGUGUCUCAUGGCUCUGGUUUUCCAGAGUCUUCAGUGACUCUUGAAUCUCCUCUCCCAGAGACUUCAGGGU
>random_seq_from_cds__NO_4961 RANDOM_LENGTH 83 SEQ_LENGTH 77 GC_CONTENT 0.701298701298701 BASEPAIR 26 FREE_ENERGY -36.60 LEN_BP_RATIO 2.96153846153846
GGGACUGGCCUGUACUGGCCUCUUGCCGCUGGCCCCCAGGCUCCUCUGGUCCAGGCAGGACGCUGGCAGGAAUGGCCACCCUG
>random_seq_from_cds__NO_4963 RANDOM_LENGTH 71 SEQ_LENGTH 53 GC_CONTENT 0.622641509433962 BASEPAIR 19 FREE_ENERGY -17.20 LEN_BP_RATIO 2.78947368421053
CUGAGGUGUGGGGGGGAUGGGGGUGCUGGCCAAGGUAGGGGUACGGGCCUGGAGGAAUUCUUGGUUCUUAA
>random_seq_from_cds__NO_4970 RANDOM_LENGTH 89 SEQ_LENGTH 80 GC_CONTENT 0.725 BASEPAIR 24 FREE_ENERGY -32.69 LEN_BP_RATIO 3.33333333333333
CUGGCCCAGCGACGUCUCCAUGUGUGCCACGCGCUCCUGGUAGCCGCGCACUGCCCCGCGCCACGCCUCGCCCAGUCGCCUUGCCAGCU
>random_seq_from_cds__NO_4974 RANDOM_LENGTH 75 SEQ_LENGTH 64 GC_CONTENT 0.78125 BASEPAIR 23 FREE_ENERGY -27.00 LEN_BP_RATIO 2.78260869565217
CCAGGUUGUCGCGCGCCACCUCGGCCGCGUGCUUCUCCCGCCAGCGUUGGUCAACGAGGGCCCGCAGGGCCGCCA
>random_seq_from_cds__NO_4997 RANDOM_LENGTH 81 SEQ_LENGTH 69 GC_CONTENT 0.492753623188406 BASEPAIR 23 FREE_ENERGY -18.20 LEN_BP_RATIO 3
UGUGUUUCCCAAGGCCUCCCAUCUGAAUGAGACAUUGUCAUCUGCAUCCUCCACAGAGCAAGUCAGAUGGAGCUCACAGGU
>random_seq_from_cds__NO_4999 RANDOM_LENGTH 82 SEQ_LENGTH 65 GC_CONTENT 0.523076923076923 BASEPAIR 24 FREE_ENERGY -24.10 LEN_BP_RATIO 2.70833333333333
GUGUAACUGGACAGCUUUGCAGAGGUCUUUGUGGAUAUCUGGGCUCUGUAAGAGCCUGUGUCUUCCAUCUUCAGGUUGCUGA
>random_seq_from_cds__NO_5002 RANDOM_LENGTH 86 SEQ_LENGTH 74 GC_CONTENT 0.5 BASEPAIR 27 FREE_ENERGY -26.40 LEN_BP_RATIO 2.74074074074074
AACUCCAGGGGAAGAGUUACUGACUCCCCCAGAAUCCCGUUCACCAUCAAUGGGGUUAAGCUGCUUUGUGAAACUACAUUCCCCUG
>random_seq_from_cds__NO_5019 RANDOM_LENGTH 89 SEQ_LENGTH 76

GC_CONTENT 0.473684210526316 BASEPAIR 26 FREE_ENERGY -23.90 LEN_BP_RATIO 2.92307692307692
UCAUUGGGAGAAGCUGUCUUUGCUCGGUGCUUAUCAUCUCUGAAGGAUGAGAGAAUUCAAGCUAG
CAAAAAGCUGAAGGGUCCCCAGAA
>random_seq_from_cds__NO_5031 RANDOM_LENGTH 110 SEQ_LENGTH 103 GC_CONTENT 0.621359223300971 BASEPAIR 36 FREE_ENERGY -41.20 LEN_BP_RATIO 2.86111111111111
CCCUUCAUCUCAGGGUCCAGAGGUGACUGGUGUGGAUCCUGCUCGCUGUGCCAAGAUGGCCCUCC
AGAUGCGGCAUACCAUCCCUCCCCCUGAUGCGGGUGUAGAUGAGG
>random_seq_from_cds__NO_5034 RANDOM_LENGTH 69 SEQ_LENGTH 52 GC_CONTENT 0.615384615384615 BASEPAIR 19 FREE_ENERGY -15.50 LEN_BP_RATIO 2.73684210526316
GCACCAGGGCCUGGUACUCCCGUUCCUUCCCCGCCAGGAUUUCGCGCAGCCCGAUCAGUCUCUGC
CCUC
>random_seq_from_cds__NO_5039 RANDOM_LENGTH 69 SEQ_LENGTH 58 GC_CONTENT 0.706896551724138 BASEPAIR 21 FREE_ENERGY -28.90 LEN_BP_RATIO 2.76190476190476
CUGGGCGAGCUGCCGGCGGUUGGGAGUGUGGAUGUGUGCCCCGAGGCAGCGCAGCAGCUCUUCCA
CAUG
>random_seq_from_cds__NO_5046 RANDOM_LENGTH 110 SEQ_LENGTH 100 GC_CONTENT 0.53 BASEPAIR 32 FREE_ENERGY -30.50 LEN_BP_RATIO 3.125
CCAUCUCAAUGACAGUGCAGCCCAGUGACCAGAUGUCAGCUGCUUUCCCAUACCCGCGUGGGCCC
UGGUCAAUGAUUUCUGGGGCCAUAUACUGCAGAGUUCCCUGUGAA
>random_seq_from_cds__NO_5078 RANDOM_LENGTH 72 SEQ_LENGTH 66 GC_CONTENT 0.636363636363636 BASEPAIR 21 FREE_ENERGY -27.59 LEN_BP_RATIO 3.14285714285714
ACUUCUCCGGCGGUGGUGUCUGUGGAUGGGCGGCCCGUGAGACUCCAACUCUGUGACACUGCCGG
ACAGGGA
>random_seq_from_cds__NO_5101 RANDOM_LENGTH 71 SEQ_LENGTH 63 GC_CONTENT 0.571428571428571 BASEPAIR 21 FREE_ENERGY -18.30 LEN_BP_RATIO 3
CCCUUCCUCGUCGUGGUACCCAACCUGUGGGUUCUCUAGAACUAAAGUCUGCCUGCCAGGUGACU
GCUGGA
>random_seq_from_cds__NO_5103 RANDOM_LENGTH 92 SEQ_LENGTH 78 GC_CONTENT 0.551282051282051 BASEPAIR 28 FREE_ENERGY -30.20 LEN_BP_RATIO 2.78571428571429
CUUGAGUCAUGUCCUGGUGAGACGGUUGUGGCAGAAGAUCCCGCCGGGCUGAAGGGUCCCUUUGC
UACUACACCAGAAGCAGGCAUUGGCUU
>random_seq_from_cds__NO_5111 RANDOM_LENGTH 95 SEQ_LENGTH 91 GC_CONTENT 0.450549450549451 BASEPAIR 27 FREE_ENERGY -21.73 LEN_BP_RATIO 3.37037037037037
AGUCAGGUUGACAAUGGCUCAAAGAAAGGAGGAGAACGGUUAAGUAUUUUAACCAAGAGCCUUUU
GCUGAGGAGAACAAAAGACCAGCUGGACUC

>random_seq_from_cds__NO_5117 RANDOM_LENGTH 87 SEQ_LENGTH 79 GC_CONTENT 0.468354430379747 BASEPAIR 27 FREE_ENERGY -17.90 LEN_BP_RATIO 2.92592592592593
UUUCCCUUAACGGCACCUUCUUCAAGAUGGAGCUUUUUGAAGGCAUGCGAGAGAGCACCAAGGAUUUCAUCUCUGUUGGCAGAAUUG
>random_seq_from_cds__NO_5128 RANDOM_LENGTH 75 SEQ_LENGTH 52 GC_CONTENT 0.480769230769231 BASEPAIR 19 FREE_ENERGY -18.80 LEN_BP_RATIO 2.73684210526316
GAUGAAAAUGAGGCACAAGCUGGCUGGAUUGAGGGGGCAGCCAUCCUUUUCUCAGUGAUCAUCGUGGUGUUAGUG
>random_seq_from_cds__NO_5129 RANDOM_LENGTH 67 SEQ_LENGTH 58 GC_CONTENT 0.517241379310345 BASEPAIR 21 FREE_ENERGY -15.80 LEN_BP_RATIO 2.76190476190476
ACUGCCUUUAAUGAUUGGAGCAAAGAGAAGCAAUUCCGGGGGCUGCAGUGCCGCAUUGAACAGGAGC
>random_seq_from_cds__NO_5137 RANDOM_LENGTH 75 SEQ_LENGTH 57 GC_CONTENT 0.526315789473684 BASEPAIR 20 FREE_ENERGY -22.76 LEN_BP_RATIO 2.85
GUACUCCCAUCUACAUCCAGUACUUUGUCAAGUUCUUCAUCAUCGGCAUCACUGUACUGGUGGUGGCUGUGCCAG
>random_seq_from_cds__NO_5149 RANDOM_LENGTH 110 SEQ_LENGTH 85 GC_CONTENT 0.576470588235294 BASEPAIR 26 FREE_ENERGY -30.30 LEN_BP_RATIO 3.26923076923077
AACGAGAAAGGCGAGGGUAGAGCAAGAAAAGCUGGACAAGAUCUGGCCUAAGCUUCGGGUCCUGGCGCGAUCUUCUCCCACUGACAAGCACACCCUGGUGAAAGGGCAUA
>random_seq_from_cds__NO_5151 RANDOM_LENGTH 82 SEQ_LENGTH 57 GC_CONTENT 0.508771929824561 BASEPAIR 19 FREE_ENERGY -18.30 LEN_BP_RATIO 3
GGAUGUUGGUUUUGCCAUGGGGCAUCGCAGGCACAGAUGUAGCAAAGGAGGCUUCAGACAUCAUCCUAACAGAUGACAACUU
>random_seq_from_cds__NO_5152 RANDOM_LENGTH 73 SEQ_LENGTH 60 GC_CONTENT 0.516666666666667 BASEPAIR 22 FREE_ENERGY -22.10 LEN_BP_RATIO 2.72727272727273
CACCAGCAUUGUGAAGGCAGUGAUGUGGGGACGAAAUGUCUAUGACAGCAUCUCCAAGUUCCUGCAGUUCCAG
>random_seq_from_cds__NO_5156 RANDOM_LENGTH 82 SEQ_LENGTH 71 GC_CONTENT 0.422535211267606 BASEPAIR 24 FREE_ENERGY -20.41 LEN_BP_RATIO 2.95833333333333
CAUUCUAUCAGCUCAUUGUCAUCUUUAUCCUUGUCUUUGCGGGGUGAGAAAUUCUUUGAUAUUGAUAGUGGGAGGAAGGCAC
>random_seq_from_cds__NO_5164 RANDOM_LENGTH 89 SEQ_LENGTH 77 GC_CONTENT 0.506493506493506 BASEPAIR 23 FREE_ENERGY -17.59 LEN_BP_RATIO 3.34782608695652
UAAGUUUGGGACUAGGGUGCUCCUGUUGGAUGGUGAGGUCACUCCAUAUGCCAAUACAAACAACA

AUGCGGUGGAUUGCAACCAAGUGC
>random_seq_from_cds__NO_5182 RANDOM_LENGTH 82 SEQ_LENGTH 71 GC_CONTENT 0.71830985915493 BASEPAIR 23 FREE_ENERGY -32.10 LEN_BP_RATIO 3.08695652173913
GGGACGGAGCGGGAGGCCGAGGCUGGGGGCAGGUCCCGGCGCACGCCCAUAAGCAGAAUCCCCAGGGCCAGACUGACUCCCA
>random_seq_from_cds__NO_5209 RANDOM_LENGTH 110 SEQ_LENGTH 89 GC_CONTENT 0.426966292134831 BASEPAIR 30 FREE_ENERGY -28.30 LEN_BP_RATIO 2.96666666666667
AACAUCUGAUGAUUCUGGGCUAGAGCUUGAUAAUAACAAUGCAGCAAUGGCAAUUGAUCCAGUAAUGGAUGGUGCUAGUGAAAUUGAAUUAGUAUUCAGGCCUCAUCCCA
>random_seq_from_cds__NO_5210 RANDOM_LENGTH 89 SEQ_LENGTH 70 GC_CONTENT 0.428571428571429 BASEPAIR 23 FREE_ENERGY -17.60 LEN_BP_RATIO 3.04347826086957
CACUUAUGGAAAAAGAUGACAGUGCACAGACGAGGAUACAUAAAGACUUCUGGUAACGCCACUGUUGAUCACUUAUCCAAGUAUCUGGC
>random_seq_from_cds__NO_5225 RANDOM_LENGTH 78 SEQ_LENGTH 65 GC_CONTENT 0.430769230769231 BASEPAIR 23 FREE_ENERGY -19.80 LEN_BP_RATIO 2.82608695652174
AAAGAGUCUUGACAUUGGAAUGGCUGAGGAUGUAGGAACAAAAUUCUGCAACUUGUUCUAUGAAAUCAGGGUUCAGCU
>random_seq_from_cds__NO_5232 RANDOM_LENGTH 71 SEQ_LENGTH 49 GC_CONTENT 0.551020408163265 BASEPAIR 20 FREE_ENERGY -20.24 LEN_BP_RATIO 2.45
GUGUUCUGGCUUCUUGGGAUGAGGCACACACCACAUCCAGAUUCCCUUGGUGUGAGACUUCACUGUGGAGC
>random_seq_from_cds__NO_5244 RANDOM_LENGTH 65 SEQ_LENGTH 51 GC_CONTENT 0.529411764705882 BASEPAIR 19 FREE_ENERGY -16.80 LEN_BP_RATIO 2.68421052631579
UCUGAAUCUCGUUACCUCCCAGCAGACCAGGAGUAUACUGUUGGCUGUUAGGAAUAUGUUGUGGC
>random_seq_from_cds__NO_5252 RANDOM_LENGTH 89 SEQ_LENGTH 66 GC_CONTENT 0.454545454545455 BASEPAIR 21 FREE_ENERGY -17.40 LEN_BP_RATIO 3.14285714285714
AACAACCUACAGAAUAUCAUCUAUAACCCGGGUAAUCCCGUUUGUUGGCACCAUUCCUGAUCAGCUGGAUCCUGGAACUUUGAUUGUGA
>random_seq_from_cds__NO_5270 RANDOM_LENGTH 99 SEQ_LENGTH 89 GC_CONTENT 0.50561797752809 BASEPAIR 26 FREE_ENERGY -27.03 LEN_BP_RATIO 3.42307692307692
CCGCAUGCCCUAUAUCUCAGACAAGCACCCUCGACAAACCUUGGAAGUGAUUAACCUUCUGAGAAAGCACCGGGAGCUAUGUGAUGUGGUGCUAGUUGU
>random_seq_from_cds__NO_5276 RANDOM_LENGTH 80 SEQ_LENGTH 73 GC_CONTENT 0.493150684931507 BASEPAIR 24 FREE_ENERGY -22.70 LEN_BP_RATIO 3.04166666666667
GUGUUCAAUGCAGUGAUGGCCUGGGUCAAAUACAGUAUUCAGGAAAGACGUCCUCAAUUACCCCA

GGGUGCUGCAGCAUG
>random_seq_from_cds__NO_5280 RANDOM_LENGTH 82 SEQ_LENGTH 70 GC_CONTENT 0.485714285714286 BASEPAIR 27 FREE_ENERGY -25.80 LEN_BP_RATIO 2.59259259259259
CAGACCAAUGAAUGGAGAAUGGUGGCUUCAAUGAGCAAAAGGAGAUGCGGAGUUGGGGUCAGUGU
UCUUGAUGAUCUGUUAU
>random_seq_from_cds__NO_5281 RANDOM_LENGTH 110 SEQ_LENGTH 94 GC_CONTENT 0.5 BASEPAIR 30 FREE_ENERGY -31.10 LEN_BP_RATIO 3.13333333333333
AUGCAGUAGGAGGCCAUGAUGGAUCCUCUUAUCUCAAUAGUGUUGAAAGGGUAUGACCCCAAAAC
AAACCAGUGGAGCAGUGAUGUGGCCCCUACAAGCACCUGCAGGAC
>random_seq_from_cds__NO_5294 RANDOM_LENGTH 80 SEQ_LENGTH 65 GC_CONTENT 0.538461538461538 BASEPAIR 24 FREE_ENERGY -24.80 LEN_BP_RATIO 2.70833333333333
GAUGGCCAUGGCAGUCAGUGGCAGCAGAAAGAGGCAGCAGAAGGUGAAGAGGUUAUAGGUGGUCU
CUUGCCAUUGAGCCU
>random_seq_from_cds__NO_5296 RANDOM_LENGTH 72 SEQ_LENGTH 60 GC_CONTENT 0.6 BASEPAIR 20 FREE_ENERGY -20.40 LEN_BP_RATIO 3
UGCCCCCAGAAGUUUCCUUACACCUGAACGGGAUCCAAGCGGGUUGAGUACUGCUGCCUGGCGGU
CCAAUCC
>random_seq_from_cds__NO_5301 RANDOM_LENGTH 84 SEQ_LENGTH 65 GC_CONTENT 0.523076923076923 BASEPAIR 25 FREE_ENERGY -27.50 LEN_BP_RATIO 2.6
UGCAGCUGUUCUACUUUCCUUUUCUCUUGGAGCCCUGGCCGAUCGGGCAUUGAUGAGAACAAGGG
CUGUGUAGAGGGCAAUCUC
>random_seq_from_cds__NO_5307 RANDOM_LENGTH 64 SEQ_LENGTH 58 GC_CONTENT 0.689655172413793 BASEPAIR 23 FREE_ENERGY -27.60 LEN_BP_RATIO 2.52173913043478
GGUGUGCUGCGGAAACUGGGGCUGCCGUAGCUGUCUGGGCCCUGUCCCAGUUCCCCAAGCCCAG
>random_seq_from_cds__NO_5309 RANDOM_LENGTH 85 SEQ_LENGTH 66 GC_CONTENT 0.636363636363636 BASEPAIR 26 FREE_ENERGY -26.60 LEN_BP_RATIO 2.53846153846154
CUCUCUCUGCCCUCAGCCUUGCCCCGCUCAGGGCUGUAUUCAAGGUGGCAUGAGGCCCCAUUGAG
CCCUGCCUUGGCCAAGUUGU
>random_seq_from_cds__NO_5311 RANDOM_LENGTH 82 SEQ_LENGTH 74 GC_CONTENT 0.662162162162162 BASEPAIR 28 FREE_ENERGY -40.20 LEN_BP_RATIO 2.64285714285714
GGCAGCUGCCCGUCUGGGAGCCCCAAGGUGUAGGUGAGGGUAUCUGCUCCUUGGGCCCCUGCUGG
AGGGGUCUUGACCACUG
>random_seq_from_cds__NO_5316 RANDOM_LENGTH 90 SEQ_LENGTH 81 GC_CONTENT 0.62962962962963 BASEPAIR 25 FREE_ENERGY -30.42 LEN_BP_RATIO 3.24
UGCAGGUGUGGGUCUUCUUUGCAGCCAGCAGCUCCCCGUGAGGCUCGGUGCUGUCUCUGUGGGGC

CCUGUCCAUUCUAUUUCCAGCGCCC
>random_seq_from_cds__NO_5322 RANDOM_LENGTH 94 SEQ_LENGTH 77 GC_CONTENT 0.519480519480519 BASEPAIR 25 FREE_ENERGY -16.31 LEN_BP_RATIO 3.08
CUCUGCCCGACGUCUCUCCGAUCCUGUCUUUGAGAGAAACGAGCUCCUCCUCUUUCUUCCUG
UUCUCAAAGUGAGCCUCGAUCAGCGCCUG
>random_seq_from_cds__NO_5332 RANDOM_LENGTH 80 SEQ_LENGTH 65 GC_CONTENT 0.615384615384615 BASEPAIR 21 FREE_ENERGY -23.90 LEN_BP_RATIO 3.0952380952381
ACCUCGAUCAUCUUCUGGUACAUGUCCUUCCGCAGGCUGGGCUUCUUCCGGGCCUUCGUGAGCUC
CUCCUCCAGGUAGGU
>random_seq_from_cds__NO_5334 RANDOM_LENGTH 71 SEQ_LENGTH 58 GC_CONTENT 0.603448275862069 BASEPAIR 24 FREE_ENERGY -21.20 LEN_BP_RATIO 2.41666666666667
UCUGGUUGUAGCGCUCCCCGUCCUUCACCACAUCCCCAUGGUAGGCAGGUACGAAGGGCCUCAGC
ACAUCC
>random_seq_from_cds__NO_5335 RANDOM_LENGTH 64 SEQ_LENGTH 53 GC_CONTENT 0.622641509433962 BASEPAIR 20 FREE_ENERGY -20.80 LEN_BP_RATIO 2.65
ACCAUCAGCCGGUCCAGGCAGCGCUGCUCUGACUCACAGUGCUUCUUCAGGAUCCUGCCAUUGG
>random_seq_from_cds__NO_5339 RANDOM_LENGTH 81 SEQ_LENGTH 73 GC_CONTENT 0.684931506849315 BASEPAIR 29 FREE_ENERGY -45.20 LEN_BP_RATIO 2.51724137931034
CCCGUGGAGGAGGCCGAGGAAGAGGACAGUUUCCUCAGGGGCAGGUUGCCCCGAGGGCUUCCCUG
CGUCUCCUCCAAGGCC
>random_seq_from_cds__NO_5346 RANDOM_LENGTH 71 SEQ_LENGTH 60 GC_CONTENT 0.633333333333333 BASEPAIR 20 FREE_ENERGY -23.20 LEN_BP_RATIO 3
CACAGUCGUCUCCUCUGGCCUUUUGCCCACUUCAGGCUCCCCAGAGCCCGGCAUGCCACAGGGCA
GAUAUC
>random_seq_from_cds__NO_5348 RANDOM_LENGTH 86 SEQ_LENGTH 65 GC_CONTENT 0.707692307692308 BASEPAIR 27 FREE_ENERGY -36.80 LEN_BP_RATIO 2.40740740740741
CUGCCUCUCCACCAGGGCCUCUGGGGGCUGCAGGUCCUCAAGCUCACGGGCUCUCCCAGACGGCU
CAGUGAGGGCAAGAUCCUGUG
>random_seq_from_cds__NO_5360 RANDOM_LENGTH 82 SEQ_LENGTH 64 GC_CONTENT 0.734375 BASEPAIR 25 FREE_ENERGY -31.40 LEN_BP_RATIO 2.56
GCAGUACACAGCCAUCAUGCAGCGCGCUGGCUCCAGCGGUGGCCGCGGGGAAUGUGACAUCAGCG
GCGCCGGGCGCUUGGGG
>random_seq_from_cds__NO_5361 RANDOM_LENGTH 110 SEQ_LENGTH 78 GC_CONTENT 0.756410256410256 BASEPAIR 31 FREE_ENERGY -48.10 LEN_BP_RATIO 2.51612903225806
CUGGAGGAGGCAGCUCGCCUCAGCUGCGCUGUGCACACCUCGCCCGGGGGAGGACGCAGACCCGG

GCAGGCGGCAGGGAUGUCGGCGAAGGAGAGGCCAAAGGGCAAAGU
>random_seq_from_cds__NO_5381 RANDOM_LENGTH 84 SEQ_LENGTH 65 GC_CONTENT 0.538461538461538 BASEPAIR 20 FREE_ENERGY -19.40 LEN_BP_RATIO 3.25
CUCCUGCACUGGCUCCAUCCGCUAUAAAACCUUGACAGACCAUGAGCCCAGUGGGAUAGUGAGGGUUGAGGCUCACCCAGAGAA
>random_seq_from_cds__NO_5390 RANDOM_LENGTH 92 SEQ_LENGTH 83 GC_CONTENT 0.481927710843373 BASEPAIR 27 FREE_ENERGY -18.20 LEN_BP_RATIO 3.07407407407407
CUGUGUCCACAGCGUUCAAGGCAAACAGUGAGUUUUUCCAGAUUCUCAGUCAGAAUGGAGCACCUCAGGCAGAUGUGAGCAUGUACAGUCUU
>random_seq_from_cds__NO_5402 RANDOM_LENGTH 110 SEQ_LENGTH 97 GC_CONTENT 0.65979381443299 BASEPAIR 33 FREE_ENERGY -42.10 LEN_BP_RATIO 2.93939393939394
UGGGGAGCAGCGGGUCGCAGGAGCAGCUUGUCAGCAUCGCCUCCUCCAGUGAGGCCAGUGGGCACCGUGUGGAGGAGACGAAGGCGGAGCAGGAUGACCUUGCAGCAGGU
>random_seq_from_cds__NO_5409 RANDOM_LENGTH 73 SEQ_LENGTH 61 GC_CONTENT 0.639344262295082 BASEPAIR 19 FREE_ENERGY -17.94 LEN_BP_RATIO 3.21052631578947
CCAUGUCCCACCCCCAGAGACAGGCCAGGGAUGCUACCCUCUUCUGUGAGCCCUGGACCCUGAACAUGCAGCC
>random_seq_from_cds__NO_5415 RANDOM_LENGTH 81 SEQ_LENGTH 63 GC_CONTENT 0.634920634920635 BASEPAIR 23 FREE_ENERGY -29.20 LEN_BP_RATIO 2.73913043478261
CAGCCCCCGGAACUGCACCGGAAGGCCUGCAUGGGCUGCCCUUGUCCGAGGGCUUGCAGCCUUACCCAGCUUUCCCUUUUC
>random_seq_from_cds__NO_5430 RANDOM_LENGTH 71 SEQ_LENGTH 63 GC_CONTENT 0.714285714285714 BASEPAIR 21 FREE_ENERGY -32.80 LEN_BP_RATIO 3
CACCCAGUGAGCCUCCUGCUGCAGGUGCCCAAUGUGGGAGGCGGCUUGGAGGCCUGGCACAUCCGGGGUUG
>random_seq_from_cds__NO_5437 RANDOM_LENGTH 79 SEQ_LENGTH 69 GC_CONTENT 0.579710144927536 BASEPAIR 23 FREE_ENERGY -19.12 LEN_BP_RATIO 3
GCCCAGAACUUUGGUCUCCAGCAUCUCUCCAGCGGCCACUUCUUGCGGGAGAACAUCAAGGCCAGCACCGGAAGUUGGU
>random_seq_from_cds__NO_5467 RANDOM_LENGTH 82 SEQ_LENGTH 53 GC_CONTENT 0.547169811320755 BASEPAIR 19 FREE_ENERGY -16.30 LEN_BP_RATIO 2.78947368421053
CCGUCGCUAUAUCCGCAGACUCCGGGCUAUCCGGGAGCUCAAUAGGAACUGUAGAAGAUUUGAAAAAUAAUGAAAGCCAAUG
>random_seq_from_cds__NO_5469 RANDOM_LENGTH 102 SEQ_LENGTH 82 GC_CONTENT 0.573170731707317 BASEPAIR 27 FREE_ENERGY -24.20 LEN_BP_RATIO 3.03703703703704

CUGUGCUGAUGCUGGCCUACUUGACGAGAGCUUCCUGAGAAGAUGUCUGAAUUUUAUGGCCUUC
UCAUUCAGCUGCUGCUCCGCAUCCUGGACCCCGCAUA
>random_seq_from_cds__NO_5479 RANDOM_LENGTH 90 SEQ_LENGTH 71
GC_CONTENT 0.492957746478873 BASEPAIR 24 FREE_ENERGY
-17.60 LEN_BP_RATIO 2.95833333333333
AGCAACUUUGUGGCCCCAAGUGCCGUGACCUGAAAGUUGAAAACCCUGAGAAAUACGGCUUUGAA
CCAAAGAAGCUGUUGGACCAACUGA
>random_seq_from_cds__NO_5480 RANDOM_LENGTH 83 SEQ_LENGTH 77
GC_CONTENT 0.480519480519481 BASEPAIR 24 FREE_ENERGY
-20.10 LEN_BP_RATIO 3.20833333333333
CGGAUAUUUACUUACAGCUGGACUGUGCUCGGUUCGCGAAAGCCAUUGCUGACGACCAGGAGAUC
CUACAGUAAGGAAUUGUU
>random_seq_from_cds__NO_5488 RANDOM_LENGTH 72 SEQ_LENGTH 65
GC_CONTENT 0.676923076923077 BASEPAIR 27 FREE_ENERGY
-36.00 LEN_BP_RATIO 2.40740740740741
AGGCCUGGGGAUGGCCGAGGGAAUGGGCAGUUGACUGGAAGAUUUAGCAGCUGCUCCCUGCGGCC
GGGGCAG
>random_seq_from_cds__NO_5491 RANDOM_LENGTH 110 SEQ_LENGTH 81
GC_CONTENT 0.666666666666667 BASEPAIR 31 FREE_ENERGY
-43.27 LEN_BP_RATIO 2.61290322580645
GAGGGCCUCUUUCCAGAUGUAGCCCGGAGAGCCCUGACUGACCCCUUCCUAUCAUUACUCCGGAG
UCGAGGCGUCAGGCUGCUUGGGGAGGGGGUGCUCCGCGUCAAAGA
>random_seq_from_cds__NO_5498 RANDOM_LENGTH 84 SEQ_LENGTH 73
GC_CONTENT 0.438356164383562 BASEPAIR 23 FREE_ENERGY
-16.30 LEN_BP_RATIO 3.17391304347826
GUGCAUUUGCCCAGUAUAACAUGGAUCAGUUCACGCCUGUGAAGAUAGAAGGAUAUGAAGAUCAG
GGUCUUAAUUACAGAGCAC
>random_seq_from_cds__NO_5518 RANDOM_LENGTH 69 SEQ_LENGTH 61
GC_CONTENT 0.557377049180328 BASEPAIR 20 FREE_ENERGY
-18.51 LEN_BP_RATIO 3.05
GCUGAGCAUUUCCCUGGGACCAGGAGACAGAGAUGUGGCCUAUUCCUGCAUUGUCUCCAACCCUG
UCAG
>random_seq_from_cds__NO_5519 RANDOM_LENGTH 83 SEQ_LENGTH 62
GC_CONTENT 0.645161290322581 BASEPAIR 20 FREE_ENERGY
-26.40 LEN_BP_RATIO 3.1
CUGGGACUUGGCCACAGUCACGCCCUGGGAUAGCUGUCAUCAUGAGGCAGGCACCAGGGAAGGCC
UCCUACAAAGAUGUGCUG
>random_seq_from_cds__NO_5530 RANDOM_LENGTH 110 SEQ_LENGTH 79
GC_CONTENT 0.417721518987342 BASEPAIR 26 FREE_ENERGY
-19.00 LEN_BP_RATIO 3.03846153846154
UAUAUUCUUUAAGAACCAAUUCCCAGUCUAAGUCUGGAAUGGUCAAGUUUGCAAGAGUUCCCAAA
CAUUCAAUCACAAACUCCUCUUCUUCAUCAUUAGAGAUCUGGGCU
>random_seq_from_cds__NO_5535 RANDOM_LENGTH 85 SEQ_LENGTH 63
GC_CONTENT 0.444444444444444 BASEPAIR 22 FREE_ENERGY

-17.40 LEN_BP_RATIO 2.86363636363636
GAAGCAGUCCAACUUGUACCAUCUUAUUCCUCAGUCCUGUGUCAAAGGAUAGGUUUAGUAAAAGU
CGGAGGGUGAUAUUCAGCAG
>random_seq_from_cds__NO_5549 RANDOM_LENGTH 87 SEQ_LENGTH 60
GC_CONTENT 0.65 BASEPAIR 23 FREE_ENERGY -28.00
LEN_BP_RATIO 2.60869565217391
UUGCAUCUCAUGCUGUGCUCAGCGGGUUGGCCACGCGGCCCAGGAAGUGCAGGGCAGUGGCGCUU
UGAUCAUACACAGCAAACAGGA
>random_seq_from_cds__NO_5553 RANDOM_LENGTH 109 SEQ_LENGTH 96
GC_CONTENT 0.59375 BASEPAIR 32 FREE_ENERGY -32.70
LEN_BP_RATIO 3
AGGUCAGAGGCAUAGUGAGGCUGGAUCAGCAGCAGGCAGGCGCUCUCAGUGAAGGGCACUUGAGU
CACCGAGAAGUUGUCCUGGAUGUCACUCCAGUGCUGGAAGGUGC
>random_seq_from_cds__NO_5556 RANDOM_LENGTH 93 SEQ_LENGTH 78
GC_CONTENT 0.551282051282051 BASEPAIR 28 FREE_ENERGY
-26.80 LEN_BP_RATIO 2.78571428571429
CCAGUCUUCCAUCCUGUCACAGCCUGCAUGAACCUGUCAAUCUUCUCAGCAGCAACAUCCAGUUC
UGUGAAGUCCAGAGAGCGUGGGAGGACC
>random_seq_from_cds__NO_5557 RANDOM_LENGTH 71 SEQ_LENGTH 65
GC_CONTENT 0.646153846153846 BASEPAIR 22 FREE_ENERGY
-27.10 LEN_BP_RATIO 2.95454545454545
ACAGGGGUAUAGAGAGCCAGGCCCUGCACAAACGGCUGCUUCAGGUGCAGGCCUGGGGCUGUGAA
CACGCC
>random_seq_from_cds__NO_5560 RANDOM_LENGTH 78 SEQ_LENGTH 66
GC_CONTENT 0.651515151515151 BASEPAIR 21 FREE_ENERGY
-24.10 LEN_BP_RATIO 3.14285714285714
GCCUGUCAGCUGUGUGGUCCAAGGCUCCCAGAUAGAGAGAGGCCAGGGUGCCAAAGACAGCCGUU
GGGGAGAGGACGG
>random_seq_from_cds__NO_5578 RANDOM_LENGTH 89 SEQ_LENGTH 79
GC_CONTENT 0.594936708860759 BASEPAIR 27 FREE_ENERGY
-26.20 LEN_BP_RATIO 2.92592592592593
GCUCCAGCUUCUGGUCAUGAUUCACCAGCUGUCCACCCUGCGGGACCAGCUCCUGACAGCCCACU
CGGAGCAGAAGAACAUGGCUGCCA
>random_seq_from_cds__NO_5588 RANDOM_LENGTH 77 SEQ_LENGTH 63
GC_CONTENT 0.619047619047619 BASEPAIR 22 FREE_ENERGY
-25.60 LEN_BP_RATIO 2.86363636363636
UUCCCCGAGUCCCGAAACAGCAGCCACAUCAAGAGGCCCAUGAACGCCUUCAUGGUGUGGGCCAA
GGAUGAGCGGAG
>random_seq_from_cds__NO_5603 RANDOM_LENGTH 68 SEQ_LENGTH 58
GC_CONTENT 0.620689655172414 BASEPAIR 19 FREE_ENERGY
-19.60 LEN_BP_RATIO 3.05263157894737
CACUGGCCACCUUCAGAAAUAUGUCCCGUGGUUCCCCGGGGCCCCACAUUCUGGGUCUCACAGAU
GAC
>random_seq_from_cds__NO_5607 RANDOM_LENGTH 87 SEQ_LENGTH 78

GC_CONTENT 0.641025641025641 BASEPAIR 29 FREE_ENERGY -29.31 LEN_BP_RATIO 2.68965517241379
CACUGCGCUGGACUUCCAAGGUGACACGGCGUCCAUGCCCUUCUCGUUGCUCAACCCAGGAGAGG CCCUGCACCAUGGCCAGCUGCC
>random_seq_from_cds__NO_5612 RANDOM_LENGTH 99 SEQ_LENGTH 79 GC_CONTENT 0.721518987341772 BASEPAIR 32 FREE_ENERGY -36.40 LEN_BP_RATIO 2.46875
CCUUCGUGCGUUCCCGGUAGGCGGCGAUGACCUGUAGGUCCCGCAAGUGCUGUGUGCGGACGCUC UGCUGCGCCCAACACGCUCGUCCCGCGCCGGCCC
>random_seq_from_cds__NO_5624 RANDOM_LENGTH 68 SEQ_LENGTH 56 GC_CONTENT 0.535714285714286 BASEPAIR 21 FREE_ENERGY -22.90 LEN_BP_RATIO 2.66666666666667
CGUCACAGGUUCUGUAGCGCUGACAGCCUCGGCUGGCUGUUUAUUGGCAUCCAGAAUCUCGGGAA AGC
>random_seq_from_cds__NO_5625 RANDOM_LENGTH 87 SEQ_LENGTH 65 GC_CONTENT 0.676923076923077 BASEPAIR 27 FREE_ENERGY -37.00 LEN_BP_RATIO 2.40740740740741
CGGAGGACUGCAGGAGCACCAUGGAGGCUCUCGAGGGCUGCCCUGCAGAGCCUCCUGGUCUGGGU UCCCACAACAAUAGGGGCAUGC
>random_seq_from_cds__NO_5627 RANDOM_LENGTH 82 SEQ_LENGTH 76 GC_CONTENT 0.684210526315789 BASEPAIR 28 FREE_ENERGY -31.40 LEN_BP_RATIO 2.71428571428571
CUCCUGCUGCCUGGGCCGGGGAGGCCUGAGAGCCAUGGGGUAGCUGUGAAGUAGGCCUGGCCAAG CAGUGCUGAGUCGGGGA
>random_seq_from_cds__NO_5629 RANDOM_LENGTH 80 SEQ_LENGTH 69 GC_CONTENT 0.710144927536232 BASEPAIR 28 FREE_ENERGY -39.10 LEN_BP_RATIO 2.46428571428571
GCGCUGGCGGGCCUCGAAGGGGACGUGGGUGGUUUCCUGGAAGGCCGCCGCUCCACUUUGGGGCC ACUGACAGGCUCCGU
>random_seq_from_cds__NO_5634 RANDOM_LENGTH 90 SEQ_LENGTH 76 GC_CONTENT 0.605263157894737 BASEPAIR 26 FREE_ENERGY -28.50 LEN_BP_RATIO 2.92307692307692
CGGAGCCUGGUCUUGGAAGAGGAGACCACUUUCCUGCUGAAGCUAGCUGAGCGCGUCAAGGCCAC AUCCAUCUCAGGCACCAGUACAACG
>random_seq_from_cds__NO_5641 RANDOM_LENGTH 85 SEQ_LENGTH 78 GC_CONTENT 0.551282051282051 BASEPAIR 24 FREE_ENERGY -21.50 LEN_BP_RATIO 3.25
CCUGGGAAGCAGAGAUAGGAGAGUCCGGCUGGUUGCUGAAGAUCCGAAGAAUUCCAAACCCACAG GACAAUGUCUGGAGGCUCCC
>random_seq_from_cds__NO_5648 RANDOM_LENGTH 64 SEQ_LENGTH 51 GC_CONTENT 0.666666666666667 BASEPAIR 20 FREE_ENERGY -20.70 LEN_BP_RATIO 2.55
UCCACGGGGACUGGGGCUGGCUCACGUAUCCGGCUCAUGGGGUGGGACUCCAUCAACGAGGUGG
>random_seq_from_cds__NO_5652 RANDOM_LENGTH 84 SEQ_LENGTH 77

GC_CONTENT 0.649350649350649 BASEPAIR 27 FREE_ENERGY -35.00 LEN_BP_RATIO 2.85185185185185
CACCAUUGCGGCCGACGAGAGCUUCACAGGUGCCGACCUUGGUGUGCGGCGUCUCAAGCUCAACACGGAGGUGCGCAGUGUGGG
>random_seq_from_cds__NO_5655 RANDOM_LENGTH 87 SEQ_LENGTH 79 GC_CONTENT 0.708860759493671 BASEPAIR 27 FREE_ENERGY -27.90 LEN_BP_RATIO 2.92592592592593
GGAGGUGAGGGGCCAGUGCGUGCGGCACUCAGAGGAGCGGGACACACCCAAGAUGUACUGCAGCGCGGAGGGCGAGUGGCUCGUGCC
>random_seq_from_cds__NO_5681 RANDOM_LENGTH 97 SEQ_LENGTH 64 GC_CONTENT 0.546875 BASEPAIR 22 FREE_ENERGY -18.90 LEN_BP_RATIO 2.90909090909091
CCCGCCGGCACCUCUGAUCUACUUCUGCAUGUCACACUGCAGCAGCAACACAAUGGAUGGGUCACUCUUGGCUGCCUCCUUGAACUCCUCCAAUGUA
>random_seq_from_cds__NO_5688 RANDOM_LENGTH 94 SEQ_LENGTH 76 GC_CONTENT 0.565789473684211 BASEPAIR 23 FREE_ENERGY -26.10 LEN_BP_RATIO 3.30434782608696
ACCGUGGGUAUGCAGACAGUCCUUCAAAAGGCAGGAGCAGCUCCAUAUGUGCAGGCAUUUGACUCGCUGCUUGCUGGUCCUGUGGCAGAGUACU
>random_seq_from_cds__NO_5694 RANDOM_LENGTH 110 SEQ_LENGTH 100 GC_CONTENT 0.65 BASEPAIR 30 FREE_ENERGY -36.40 LEN_BP_RATIO 3.33333333333333
CCUGGAGCAAAACGGGGGCCUGUGGCAAAAGAACUGAGCGGACUGCCAUCUGGACCCUCUGCCGGAUCAUGUCCUCCUCCCCCUCCACCAUGCCCCCCUCCUCCCCCAGU
>random_seq_from_cds__NO_5700 RANDOM_LENGTH 75 SEQ_LENGTH 68 GC_CONTENT 0.470588235294118 BASEPAIR 23 FREE_ENERGY -16.00 LEN_BP_RATIO 2.95652173913043
UUGGCCUGGUAUUCGAUGACGUGGUGGGCAUUGUGGAGAUAAUCAACAGUAAGGAUGUCAAAGUUCAGGGUAAUG
>random_seq_from_cds__NO_5727 RANDOM_LENGTH 97 SEQ_LENGTH 75 GC_CONTENT 0.56 BASEPAIR 29 FREE_ENERGY -28.20 LEN_BP_RATIO 2.58620689655172
UCUGCCCCUGCCAAAACAGACUGUGUGCAUCUCCUUUCAGAAGUUCAACGGUAUCCCCGGCCUGGAGCUGUAAAGGGGGUCCUUCAUGCAGAGCUGG
>random_seq_from_cds__NO_5744 RANDOM_LENGTH 100 SEQ_LENGTH 91 GC_CONTENT 0.450549450549451 BASEPAIR 34 FREE_ENERGY -26.20 LEN_BP_RATIO 2.67647058823529
UUCCACAAGGGUUUCACUCUAUUAAAUCAGGAAGGCCUUUGUAGAUGUCUUCAUCAUUAAUGCUUUCUUCUGUUGGGAAGGGCCCUGAUUCCUGUGGCCA
>random_seq_from_cds__NO_5746 RANDOM_LENGTH 97 SEQ_LENGTH 53 GC_CONTENT 0.509433962264151 BASEPAIR 21 FREE_ENERGY -26.50 LEN_BP_RATIO 2.52380952380952
AGUUCACUUUUCCUCAUUCCAAACGUCUCACAACAGGCCGUGAGAAAUGUCCUUAUGUUCUUCAAACAGAGAAACCUGGGACAUCUGCGGCCUCAGG

>random_seq_from_cds__NO_5778 RANDOM_LENGTH 71 SEQ_LENGTH 60 GC_CONTENT 0.65 BASEPAIR 22 FREE_ENERGY -26.50 LEN_BP_RATIO 2.72727272727273
UGGUCGGGGGUUCCAGAUAGAUUCGACCCCGCAUGGCCAGCUCUAUCAGGAUGCCCCCUCGCAGGCCUGAU
>random_seq_from_cds__NO_5780 RANDOM_LENGTH 84 SEQ_LENGTH 63 GC_CONTENT 0.46031746031746 BASEPAIR 20 FREE_ENERGY -20.29 LEN_BP_RATIO 3.15
CCAUAAGAGUAAGGCGGAUAUCCUUAGAGUCUCCAGAAUCUUCAUUGUCUGGACUUUUCUCCCAAUUACUGUCUUCCUCACUUU
>random_seq_from_cds__NO_5793 RANDOM_LENGTH 79 SEQ_LENGTH 69 GC_CONTENT 0.550724637681159 BASEPAIR 23 FREE_ENERGY -29.70 LEN_BP_RATIO 3
AAUUGGGAGCCAGGUCCUGAUUCCUCUUCAGCAAGGCCUCACUGAAGGAAGUUUCAUCAGGUGCUGGCUUGACCCGGGG
>random_seq_from_cds__NO_5795 RANDOM_LENGTH 109 SEQ_LENGTH 93 GC_CONTENT 0.548387096774194 BASEPAIR 32 FREE_ENERGY -36.30 LEN_BP_RATIO 2.90625
UCUGUCACCCCCUCAUAAUGAGCCUCUUUGAUCUCUUCCGGGGCUUUUUCGGCUUUCCUGGACCUCGGAGGCCACAGAGAUCCCUUUUUUGGAGGGAUGACUCGAGAUG
>random_seq_from_cds__NO_5817 RANDOM_LENGTH 95 SEQ_LENGTH 74 GC_CONTENT 0.540540540540541 BASEPAIR 26 FREE_ENERGY -31.40 LEN_BP_RATIO 2.84615384615385
AUUAUGAAACUGUCCUGUCCCUGGGAAUUCCCAUUCCCUAAGCCAGACAUGAUCACCCGUUUGGAAGGGGAGGAGGAGUCUCAGAAUUCUGACGA
>random_seq_from_cds__NO_5824 RANDOM_LENGTH 81 SEQ_LENGTH 61 GC_CONTENT 0.540983606557377 BASEPAIR 21 FREE_ENERGY -19.30 LEN_BP_RATIO 2.9047619047619
AAGGGUUCAAGGCAAGCUUGGGUUGGUGUCGAAGAAUGAUGAGAAGGUAUGACCUGUCUCUGAGGCAUAAAGUGCCCGUGC
>random_seq_from_cds__NO_5826 RANDOM_LENGTH 63 SEQ_LENGTH 55 GC_CONTENT 0.490909090909091 BASEPAIR 19 FREE_ENERGY -15.40 LEN_BP_RATIO 2.89473684210526
CGCAUGACUAUGAGGUAGCUCAGAUGGGGAAUGCAGAUGAGACGCCCAUUUGUUUAGAGGUGC
>random_seq_from_cds__NO_5830 RANDOM_LENGTH 77 SEQ_LENGTH 61 GC_CONTENT 0.60655737704918 BASEPAIR 20 FREE_ENERGY -17.60 LEN_BP_RATIO 3.05
GACUGAAGACUUGAUGCAGGACUGGUUGGAAGUGGUGUGGAGACGGAGGACAGGAGCAGUGCCCAAGCAGCGAGGGA
>random_seq_from_cds__NO_5847 RANDOM_LENGTH 71 SEQ_LENGTH 59 GC_CONTENT 0.694915254237288 BASEPAIR 19 FREE_ENERGY -22.80 LEN_BP_RATIO 3.10526315789474
CCGGAGGUCUGCUUCUCGGGGACGUGGCUCCCAACUUUGAGGCCAAUACCACCGUCGGCCGCAUCCGUUUC

>random_seq_from_cds__NO_5848 RANDOM_LENGTH 67 SEQ_LENGTH 50 GC_CONTENT 0.6 BASEPAIR 19 FREE_ENERGY -18.29 LEN_BP_RATIO 2.63157894736842
CACGACUUUCUGGGAGACUCGAUGGGGCAUUCUCUUCUCCCACCCUCGGGACUUUACCCCAGUGUGC
>random_seq_from_cds__NO_5851 RANDOM_LENGTH 72 SEQ_LENGTH 49 GC_CONTENT 0.489795918367347 BASEPAIR 19 FREE_ENERGY -17.40 LEN_BP_RATIO 2.57894736842105
AGCAGAGAAGGAUGAAAAGGGCAUGCCUGUGACAGCUCGUGUGGGUGUUUGUUUUUGGUCCUGAUAAGAAGC
>random_seq_from_cds__NO_5865 RANDOM_LENGTH 88 SEQ_LENGTH 67 GC_CONTENT 0.597014925373134 BASEPAIR 22 FREE_ENERGY -24.60 LEN_BP_RATIO 3.04545454545455
GACAGUUAAUGAAUGGGAGCUUCUCCAUGGACGGGACAGGACAAUCCUAUCAGGACUUGAGGGAUGGGAGCCCCUAUGGAAUCCCCCA
>random_seq_from_cds__NO_5866 RANDOM_LENGTH 87 SEQ_LENGTH 65 GC_CONTENT 0.553846153846154 BASEPAIR 23 FREE_ENERGY -19.90 LEN_BP_RATIO 2.82608695652174
GUCUCCAUCCUCCAUAUCGUCCCUGCCAUCCCACGCUCCUUUGCUCAAUGGGCUGGAUUACACGGUGGACAGUAAUUUGGGCAUCAU
>random_seq_from_cds__NO_5871 RANDOM_LENGTH 87 SEQ_LENGTH 79 GC_CONTENT 0.455696202531646 BASEPAIR 22 FREE_ENERGY -17.04 LEN_BP_RATIO 3.59090909090909
UAUAUCAUUAGCUUUAAAAGAGAUGAGCACCUUCAAGACAAUGCUGCCUGCCCGGUCCCUUCACUGCCUGACUCUUGGUGUUGAUAG
>random_seq_from_cds__NO_5873 RANDOM_LENGTH 88 SEQ_LENGTH 69 GC_CONTENT 0.550724637681159 BASEPAIR 21 FREE_ENERGY -17.20 LEN_BP_RATIO 3.28571428571429
AUCUUCUUCGUCCACGAACUUGUUCUCGUCAUAUUCAUCCACGUCCACCUUCCGGAAGCGGGCCGACGACACUGUGUUCUUCGACAUA
>random_seq_from_cds__NO_5874 RANDOM_LENGTH 75 SEQ_LENGTH 66 GC_CONTENT 0.636363636363636 BASEPAIR 23 FREE_ENERGY -21.70 LEN_BP_RATIO 2.8695652173913
UCAGUGGGUCGGAGAGGCAUCCUCCAAGCCCAGCAGCUCUCUGACGAGUCUCUCUCCAAACUGCGCCCGGCUGUA
>random_seq_from_cds__NO_5879 RANDOM_LENGTH 81 SEQ_LENGTH 67 GC_CONTENT 0.626865671641791 BASEPAIR 23 FREE_ENERGY -22.60 LEN_BP_RATIO 2.91304347826087
CAUGGACAGUGGGAGGCCGGUGCAGUCACUCUUCCGCGCGCGGAGCAAGCCGCAGCAGUAAAUCCCCUUGCUUCUCAAACU
>random_seq_from_cds__NO_5889 RANDOM_LENGTH 68 SEQ_LENGTH 51 GC_CONTENT 0.745098039215686 BASEPAIR 19 FREE_ENERGY -25.20 LEN_BP_RATIO 2.68421052631579
AUGGAUUAUAGGGCGGCCGAGGCGUCGACUCCAUCCACAGCUGUCGCUGCCUCUGGCCCAAGGAG

CUC
>random_seq_from_cds__NO_5926 RANDOM_LENGTH 82 SEQ_LENGTH 68 GC_CONTENT 0.441176470588235 BASEPAIR 23 FREE_ENERGY -15.30 LEN_BP_RATIO 2.95652173913043
AGAUAUCACAGAAACCACUGGUAGAGAGAACACUAAUGAAGCCUCCUCUGAAGGGAACUCUUCUGAUGAUUCUGAAGAUGAG
>random_seq_from_cds__NO_5940 RANDOM_LENGTH 77 SEQ_LENGTH 60 GC_CONTENT 0.433333333333333 BASEPAIR 20 FREE_ENERGY -15.60 LEN_BP_RATIO 3
CUGUGAUGCAUCCAUCUGGUGAUAGUGACACAACGAUGUUAGAAUCUGAAUGUCAAGCUCCUGUACAGAAGGGAUAU
>random_seq_from_cds__NO_5948 RANDOM_LENGTH 67 SEQ_LENGTH 57 GC_CONTENT 0.87719298245614 BASEPAIR 25 FREE_ENERGY -42.00 LEN_BP_RATIO 2.28
GCCGCUGCCGCCGCCGUCCGGGUCUGCGCCCGCCACGCCGAGGCCCAGGCGGCCGCGCGGCAGGGAA
>random_seq_from_cds__NO_5949 RANDOM_LENGTH 100 SEQ_LENGTH 91 GC_CONTENT 0.538461538461538 BASEPAIR 26 FREE_ENERGY -26.60 LEN_BP_RATIO 3.5
CUGGCGCUGAAGACCCUGGGGACAGAUGGCCUUUUUCUCUUUUCCUCCUUGGACACUGACGGGGAUAUGUACAUCAGCCCUGAGGAGUUCAAACCCAUUG
>random_seq_from_cds__NO_5950 RANDOM_LENGTH 84 SEQ_LENGTH 71 GC_CONTENT 0.549295774647887 BASEPAIR 27 FREE_ENERGY -25.20 LEN_BP_RATIO 2.62962962962963
CUGAGAAGCUAACAGGGGUCUUGUUCUGUCACCCAGACUGGAGUGCAGUGGUGCAGUCACAGCUCACUGCAGCCUCAACUUCCC
>random_seq_from_cds__NO_5978 RANDOM_LENGTH 84 SEQ_LENGTH 62 GC_CONTENT 0.451612903225806 BASEPAIR 21 FREE_ENERGY -16.30 LEN_BP_RATIO 2.95238095238095
CACUCUUUGUAACUUGGAAACUCAGGAGCCUUUGGAAAGUACUGCUGCAGUUUGCUCCAAGCCUCUUUAGAAACAAGCCUUCUU
>random_seq_from_cds__NO_5992 RANDOM_LENGTH 73 SEQ_LENGTH 68 GC_CONTENT 0.441176470588235 BASEPAIR 26 FREE_ENERGY -24.60 LEN_BP_RATIO 2.61538461538462
AGCUGCAAGUUCAGAGUGCAAGGAAGGCUAAGAAGUCGAAUCUUUCUUGUUGCAUUCUGUUUGCUUUGACAGU
>random_seq_from_cds__NO_6016 RANDOM_LENGTH 106 SEQ_LENGTH 70 GC_CONTENT 0.557142857142857 BASEPAIR 22 FREE_ENERGY -26.14 LEN_BP_RATIO 3.18181818181818
AUUUUGUAUUUUUCAUCAAUAAAGAACUUGAGUUUGCCAAUCAUAGGGCGGUAUAUUCUGUGGAGAUCCUUCAGGGCUGCCCCCCAGUGUGAGCUCUGAGGCUUCC
>random_seq_from_cds__NO_6028 RANDOM_LENGTH 81 SEQ_LENGTH 50 GC_CONTENT 0.62 BASEPAIR 19 FREE_ENERGY -21.40 LEN_BP_RATIO 2.63157894736842

UCCACUGCAGCCCUUCUACCGCUGGAGGACGUGGGUCCCUCCUGGGGGUUGUUAUGAUCCCUGCU
CUCCAUGAUGUCGUCG
>random_seq_from_cds__NO_6031 RANDOM_LENGTH 93 SEQ_LENGTH 86
GC_CONTENT 0.383720930232558 BASEPAIR 33 FREE_ENERGY
-23.70 LEN_BP_RATIO 2.60606060606061
CUUCAAAUGAACAACAAGAGCUUUUCUGUCAGAAGUUGCAGCAGUGUUGUAUACUGUUUGAUUUC
AUGGACUCUGUUUCAGACUUGAAGAGCA
>random_seq_from_cds__NO_6042 RANDOM_LENGTH 96 SEQ_LENGTH 78
GC_CONTENT 0.320512820512821 BASEPAIR 25 FREE_ENERGY
-18.36 LEN_BP_RATIO 3.12
UUGUACUUCUGGAAUAACGAAUAUAUUCUUAGUUUGAUUGAGGAGAACAUUGAUAAAAUUCUGCC
AAUUAUGUUUGCCAGUUUGUACAAAAUUUCC
>random_seq_from_cds__NO_6050 RANDOM_LENGTH 110 SEQ_LENGTH 92
GC_CONTENT 0.717391304347826 BASEPAIR 33 FREE_ENERGY
-51.90 LEN_BP_RATIO 2.78787878787879
UGCCCAUGGGGGCCCUGCUUGCCUGGGUACCCAGGGGGCCCAGGAGGUCCUGGAGGACCCAUCAU
GCCCACCGCACCCAGGGCUUCCCGCUUGGCACUCACGGCGACCUC
>random_seq_from_cds__NO_6059 RANDOM_LENGTH 79 SEQ_LENGTH 54
GC_CONTENT 0.722222222222222 BASEPAIR 19 FREE_ENERGY
-25.60 LEN_BP_RATIO 2.84210526315789
UCCCAUCCUUGCCGUUGAUGCCUGGGGGGCCCCGUUGCUCCUUUCGGGCCUGUGAUCCCCUGGGG
UCCACGAAUACCUG
>random_seq_from_cds__NO_6062 RANDOM_LENGTH 70 SEQ_LENGTH 51
GC_CONTENT 0.686274509803922 BASEPAIR 20 FREE_ENERGY
-29.90 LEN_BP_RATIO 2.55
CCCCGGUGGUCCAGGGAUGCCUUGCUCUCCAGAGGCACCCACAUCUCCCUUGGGACCCCGGCUUC
CCCUG
>random_seq_from_cds__NO_6065 RANDOM_LENGTH 78 SEQ_LENGTH 72
GC_CONTENT 0.666666666666667 BASEPAIR 24 FREE_ENERGY
-36.90 LEN_BP_RATIO 3
UGGGGGCCCCGAUGGUCCAUCUGGUCCAGGGUCCCCCCUUGGGGCCUCGGAUUCCAAUCUCACCA
GGGAGGCCAACAG
>random_seq_from_cds__NO_6070 RANDOM_LENGTH 82 SEQ_LENGTH 63
GC_CONTENT 0.666666666666667 BASEPAIR 21 FREE_ENERGY
-28.50 LEN_BP_RATIO 3
UCCCGGUGGACCUCUCAAUCUGCGCCAGAGCGAGCACUACCACCUGGAGGAGAACAAGGAGGCUG
CGGGGGGAGGCCGUAGC
>random_seq_from_cds__NO_6076 RANDOM_LENGTH 84 SEQ_LENGTH 64
GC_CONTENT 0.4375 BASEPAIR 20 FREE_ENERGY -18.40
LEN_BP_RATIO 3.2
AGAUUGUGAAACCUGGCUUCAACAUCAGCAUUCUGAAAAUAUUCAUCACCAUGAUGUCUGAGAGU
GUUCGGAUGAUGCUGGAAC
>random_seq_from_cds__NO_6078 RANDOM_LENGTH 88 SEQ_LENGTH 58
GC_CONTENT 0.586206896551724 BASEPAIR 19 FREE_ENERGY

-20.60 LEN_BP_RATIO 3.05263157894737
AAGUGUGCCUUCAGCCACCAGGGCAGCAUCCAGUUGGACAGGUACCCUGGACUCAUACCUGAAAG
CAGUGUUCAACCUUAGCAAAAUC
>random_seq_from_cds__NO_6099 RANDOM_LENGTH 75 SEQ_LENGTH 59
GC_CONTENT 0.559322033898305 BASEPAIR 23 FREE_ENERGY
-32.10 LEN_BP_RATIO 2.56521739130435
GCCAGAGGCUGGAAUGUGGACAGUGAAGGACCUCAAGCAGUGGAAGGCACUCUGUUCGCAUUACU
GGCCUCAGUA
>random_seq_from_cds__NO_6108 RANDOM_LENGTH 70 SEQ_LENGTH 61
GC_CONTENT 0.475409836065574 BASEPAIR 22 FREE_ENERGY
-15.10 LEN_BP_RATIO 2.77272727272727
AAUGUUACAGUCACUCCUGGAGAGAGAGCAGUUUUAACAUGUCUCAUCAUCAGUGCGGUGGAUUA
CAAUC
>random_seq_from_cds__NO_6149 RANDOM_LENGTH 84 SEQ_LENGTH 67
GC_CONTENT 0.432835820895522 BASEPAIR 22 FREE_ENERGY
-16.90 LEN_BP_RATIO 3.04545454545455
GAGAAAAGGAAAUCAAAUAUGAAGUUGAUGUCUUGGGUGCCACCAGCUAUAGAAGGAGGAGAUGA
AACAUCUUACUUCAUUGUG
>random_seq_from_cds__NO_6155 RANDOM_LENGTH 86 SEQ_LENGTH 62
GC_CONTENT 0.516129032258065 BASEPAIR 22 FREE_ENERGY
-18.00 LEN_BP_RATIO 2.81818181818182
AUGGAACCACCUAGUCUGGAAGAUGCUGGAAAAAUGCUGAAUGAGACUGUGUUGGUGAGCAACCC
UGUACAGCUGGAGUGUAAGGC
>random_seq_from_cds__NO_6170 RANDOM_LENGTH 87 SEQ_LENGTH 78
GC_CONTENT 0.423076923076923 BASEPAIR 26 FREE_ENERGY
-18.40 LEN_BP_RATIO 3
UCAUGUAUCUGACACAGGCCGUUAUGUGUGUGUUGCUGUGAAUGUAGCAGGAAUGACUGACAAAA
AAUAUGACUUAAGUGUCCAUGG
>random_seq_from_cds__NO_6173 RANDOM_LENGTH 84 SEQ_LENGTH 56
GC_CONTENT 0.482142857142857 BASEPAIR 22 FREE_ENERGY
-18.90 LEN_BP_RATIO 2.54545454545455
CUACGGCUGAUGCAGACCACAAUGGAAGAUGCUGGCCAAUAUACUUGCGUUGUAAGGAAUGCAGC
UGGUGAAGAAAGAAAAAUC
>random_seq_from_cds__NO_6177 RANDOM_LENGTH 96 SEQ_LENGTH 77
GC_CONTENT 0.532467532467532 BASEPAIR 27 FREE_ENERGY
-22.40 LEN_BP_RATIO 2.85185185185185
UGGCUUCCAGUCCAGCUGGCCACAAGAGCAGGAGCUUCAGUCUUAAUGUAUUUGGUAUCUCCUAC
AAUUGCUGGUGUAGGUAGUGAUGGCAACCCU
>random_seq_from_cds__NO_6192 RANDOM_LENGTH 110 SEQ_LENGTH 82
GC_CONTENT 0.48780487804878 BASEPAIR 29 FREE_ENERGY
-26.20 LEN_BP_RATIO 2.82758620689655
UGGUGAACAAUUUCAUCUCUUUGACCUGUGAGGUCUCUGGUUUUCCACCUCCUGACCUCAGCUGG
CUCAAGAAUGAACAGCCCAUCAAACUGAACACAAAUACUCUCAUU
>random_seq_from_cds__NO_6209 RANDOM_LENGTH 80 SEQ_LENGTH 60

GC_CONTENT 0.65 BASEPAIR 22 FREE_ENERGY -32.00 LEN_BP_RATIO 2.72727272727273
UACUGAUGGAACCCCAGCUCCCAGUAUGGCCUGGCUUAGAGAUGGCCAGCCUCUGGGGCUUGAUGCCCAUCUGACAGUCA
>random_seq_from_cds__NO_6215 RANDOM_LENGTH 66 SEQ_LENGTH 55 GC_CONTENT 0.490909090909091 BASEPAIR 20 FREE_ENERGY -17.60 LEN_BP_RATIO 2.75
AACUGAUGAGCCCCGGGAUAUCACUGUGUUACGGAACAGACAAGUGACAUUGGAAUGCAAGUCAGA
>random_seq_from_cds__NO_6218 RANDOM_LENGTH 82 SEQ_LENGTH 64 GC_CONTENT 0.453125 BASEPAIR 19 FREE_ENERGY -17.99 LEN_BP_RATIO 3.36842105263158
CUACAAGAGAAUUUAUUCUCACUGUAAAUGGUUCCUCCAAACAUAAAGGGGGGCCCCCAGAGCCUUGUAAUUCUUUUAAAUA
>random_seq_from_cds__NO_6230 RANDOM_LENGTH 67 SEQ_LENGTH 48 GC_CONTENT 0.583333333333333 BASEPAIR 19 FREE_ENERGY -21.80 LEN_BP_RATIO 2.52631578947368
CAUGCAGUUCUUCCUAGUGGCGGCUUACAGAUCUCCAGAGCUGUCCGAGAGGAUGCUGGCACUUACA
>random_seq_from_cds__NO_6231 RANDOM_LENGTH 90 SEQ_LENGTH 70 GC_CONTENT 0.585714285714286 BASEPAIR 24 FREE_ENERGY -28.44 LEN_BP_RATIO 2.91666666666667
UGUGUGUGGCCCAGAACCCGGCUGGUACAGCCUUGGGCAAAAUCAAGUUAAAUGUCCAAGGUUCCUCCAGUCAUUAGCCCUCAUCUAAAG
>random_seq_from_cds__NO_6232 RANDOM_LENGTH 84 SEQ_LENGTH 75 GC_CONTENT 0.493333333333333 BASEPAIR 28 FREE_ENERGY -18.10 LEN_BP_RATIO 2.67857142857143
GAAUAUGUUAUUGCUGUGGACAAGCCCAUCACGUUAUCCUGUGAAGCAGAUGGCCUCCCUCCGCCUGACAUUACAUGGCAUAAA
>random_seq_from_cds__NO_6237 RANDOM_LENGTH 87 SEQ_LENGTH 74 GC_CONTENT 0.527027027027027 BASEPAIR 25 FREE_ENERGY -26.30 LEN_BP_RATIO 2.96
AUGUUGUGGCUGGAGGAUUCUGGCUUCUAUACCUGUGUUGCUAACAAUGCUGCAGGUGAAGAUACACACACUGUCAGCCUGACUGUG
>random_seq_from_cds__NO_6249 RANDOM_LENGTH 85 SEQ_LENGTH 62 GC_CONTENT 0.580645161290323 BASEPAIR 24 FREE_ENERGY -25.10 LEN_BP_RATIO 2.58333333333333
AGCCUUGUCCAGGUGGAUGGUAGCUGGUCGGAAUGGAGUCUUUGGGAAGAAUGCACAAGGAGCUGUGGACGCGGCAACCAAACCA
>random_seq_from_cds__NO_6262 RANDOM_LENGTH 90 SEQ_LENGTH 85 GC_CONTENT 0.376470588235294 BASEPAIR 28 FREE_ENERGY -16.42 LEN_BP_RATIO 3.03571428571429
GGUUCAGCAAUGAGAAAGAUAGUUUCUAUUCUAAAUCCCAUUUAUUGGACAACAGCAAAGGAAAUAGGAGAAGCAGUCAAUGGCUUUACC

>random_seq_from_cds__NO_6277 RANDOM_LENGTH 84 SEQ_LENGTH 65 GC_CONTENT 0.507692307692308 BASEPAIR 24 FREE_ENERGY -20.40 LEN_BP_RATIO 2.70833333333333
CUUCAAGUGUAUCUGUCCACCAGGACAACAUUUAUUAGGGGACGGGAAAUCUUGCGCUGGAUUGG
AGAGGCUGCCAAAUUAUGG
>random_seq_from_cds__NO_6283 RANDOM_LENGTH 67 SEQ_LENGTH 53 GC_CONTENT 0.528301886792453 BASEPAIR 19 FREE_ENERGY -15.30 LEN_BP_RATIO 2.78947368421053
GGGGUUCUGCCUCAAGAACUGUCCACCCAAUGAUUUGGAAUGUGCCUUGAGCCCAUAUGCCUUGG
AA
>random_seq_from_cds__NO_6284 RANDOM_LENGTH 84 SEQ_LENGTH 72 GC_CONTENT 0.5 BASEPAIR 22 FREE_ENERGY -17.80 LEN_BP_RATIO 3.27272727272727
UACAAACUCGUCUCCCUCCCAUUUGGAAUAGCCACCAAUCAAGAUUUAAUCCGGCUGGUUGCAUA
CACACAGGAUGGAGUGAUG
>random_seq_from_cds__NO_6285 RANDOM_LENGTH 88 SEQ_LENGTH 76 GC_CONTENT 0.473684210526316 BASEPAIR 28 FREE_ENERGY -31.60 LEN_BP_RATIO 2.71428571428571
CAUCCCAGGACAACUUUCCUCAUGGUAGAUGAGGAACAGACUGUUCCUUUUGCCUUGAGGGAUGA
AAACCUGAAAGGAGUGGUGUAUA
>random_seq_from_cds__NO_6295 RANDOM_LENGTH 79 SEQ_LENGTH 67 GC_CONTENT 0.626865671641791 BASEPAIR 24 FREE_ENERGY -28.70 LEN_BP_RATIO 2.79166666666667
CUGUGGGCUGCUCCAGGUCUGAGGUCUCAGGGGCUGGGCUCUUGGCUUCAUCCUUGGGACACACA
AGGGACUCCAGCAG
>random_seq_from_cds__NO_6301 RANDOM_LENGTH 74 SEQ_LENGTH 66 GC_CONTENT 0.621212121212121 BASEPAIR 22 FREE_ENERGY -28.20 LEN_BP_RATIO 3
CCCCAUAGGAGGGAGGCCAGCUGUCCGGAGUGGCUUGAGGGGCAUAGGAGGAAGGCUGGACCUUA
GAGAUGGCC
>random_seq_from_cds__NO_6311 RANDOM_LENGTH 87 SEQ_LENGTH 75 GC_CONTENT 0.64 BASEPAIR 28 FREE_ENERGY -37.40 LEN_BP_RATIO 2.67857142857143
GGGUAGCUGUGCUGCAGAGAGCUGAACCUGUCAGUCAUCUUGGUGGCUGACCGGCCUCCCGCACU
GACAGCGGUGACCCUGGCAUAG
>random_seq_from_cds__NO_6315 RANDOM_LENGTH 67 SEQ_LENGTH 59 GC_CONTENT 0.711864406779661 BASEPAIR 22 FREE_ENERGY -25.90 LEN_BP_RATIO 2.68181818181818
CCGUCAGCUCCAGGCGGUCCUGGUGGCCGCUGCCACUGCUGCUGCUGCUGCUGCUGCUCCUGGGU
CC
>random_seq_from_cds__NO_6320 RANDOM_LENGTH 86 SEQ_LENGTH 81 GC_CONTENT 0.679012345679012 BASEPAIR 27 FREE_ENERGY -32.42 LEN_BP_RATIO 3
CUGUCUUUGCCCAGAGCAUCCCGUGGAACCUGGAGCGGAUUACCCCUCCACGGUACCGGGCGGAU

GAAUACCAGCCCCCCGGACGG
>random_seq_from_cds__NO_6322 RANDOM_LENGTH 83 SEQ_LENGTH 78 GC_CONTENT 0.602564102564103 BASEPAIR 28 FREE_ENERGY -26.50 LEN_BP_RATIO 2.78571428571429
CAUGGUCACCGACUUCGAGAAUGUGCCCGAGGAGGACGGGACCCGCUUCCACAGACAGGGCCAGCAAGUGUGACAGUCAUGGC
>random_seq_from_cds__NO_6356 RANDOM_LENGTH 84 SEQ_LENGTH 66 GC_CONTENT 0.651515151515151 BASEPAIR 22 FREE_ENERGY -24.20 LEN_BP_RATIO 3
GCCCAUGCUGACAGCCUCACCCAGGAUGCUGCUCCCUUCUUCUUCCUCGAAGCCUCCAGGCCUGGGCACAGAGACACCGCUGUC
>random_seq_from_cds__NO_6372 RANDOM_LENGTH 70 SEQ_LENGTH 62 GC_CONTENT 0.645161290322581 BASEPAIR 21 FREE_ENERGY -25.50 LEN_BP_RATIO 2.95238095238095
AAGCCCUAGGAGGGGAAGGCUGGGAGAAGCUAUCAUUGGUGCCCUCCUCUGCCCGCCUGCAUUAUGGCAC
>random_seq_from_cds__NO_6394 RANDOM_LENGTH 86 SEQ_LENGTH 67 GC_CONTENT 0.388059701492537 BASEPAIR 26 FREE_ENERGY -16.90 LEN_BP_RATIO 2.57692307692308
UUCCCGGUUGAUCGUGGAUUAUUUGAGGGAUAAAGUAGCCAAUAUUUGGUGCAGCUUCAAUGUCUUUCUGAAGAUUAAGGAUAUUU
>random_seq_from_cds__NO_6405 RANDOM_LENGTH 73 SEQ_LENGTH 60 GC_CONTENT 0.616666666666667 BASEPAIR 19 FREE_ENERGY -24.20 LEN_BP_RATIO 3.15789473684211
GUGGGAAGGAGCACUCCCCCUCACAGUAAUAGGCCGAGUAGCCUUGGGGAGCGAUGACCCAGUCCCAGCCAGC
>random_seq_from_cds__NO_6410 RANDOM_LENGTH 110 SEQ_LENGTH 92 GC_CONTENT 0.608695652173913 BASEPAIR 27 FREE_ENERGY -30.34 LEN_BP_RATIO 3.40740740740741
GUGACAUCCAGCACCAGCCAGCCCUCGUCUCCAGCUCGGAGCGUCUGAAGAUCCAAAAAGAACAAGUCAGACUCCCCUGUUGGACUGCUCCUGGACCACCUGGAACAUGC
>random_seq_from_cds__NO_6415 RANDOM_LENGTH 84 SEQ_LENGTH 76 GC_CONTENT 0.789473684210526 BASEPAIR 27 FREE_ENERGY -40.70 LEN_BP_RATIO 2.81481481481481
CGAGCACCGCCAGGAUCUCGCGCUGCACGUCCCGGCGCUCGCGCGCGCCCAGACGUCGCUGGGGACAGCCGGGCGGGGGUCGCA
>random_seq_from_cds__NO_6437 RANDOM_LENGTH 97 SEQ_LENGTH 76 GC_CONTENT 0.565789473684211 BASEPAIR 28 FREE_ENERGY -29.00 LEN_BP_RATIO 2.71428571428571
AUGUUGACUAUAUAUCCUGUUGAAACGUGGCUUCCUCCAGUCCUGAUCGACGGAACUCUGCGAGGAUGGCUCUCAGGAAGCUCUGUUCCAGAACAGA
>random_seq_from_cds__NO_6444 RANDOM_LENGTH 77 SEQ_LENGTH 60 GC_CONTENT 0.533333333333333 BASEPAIR 21 FREE_ENERGY -16.41 LEN_BP_RATIO 2.85714285714286

UUUGGCCUGUUAGCUUCCUGCAAGAUUUGCACAUAGACUUGGUGGGGCUCCGUCAGCUUCAUGCC
AUUGACCUCAAU
>random_seq_from_cds__NO_6451 RANDOM_LENGTH 110 SEQ_LENGTH 91
GC_CONTENT 0.516483516483517 BASEPAIR 29 FREE_ENERGY
-29.60 LEN_BP_RATIO 3.13793103448276
CUUUUACUAUUACCUAGAAACCCGAAGCUGCUGCCUAAUCCGAUUCAUAGUCAAGACAGAACUCU
UUCUGCGGAUACGAUGGGGAGUAGAGGUCGCUUCAUUCUGGGCUU
>random_seq_from_cds__NO_6454 RANDOM_LENGTH 72 SEQ_LENGTH 62
GC_CONTENT 0.483870967741936 BASEPAIR 23 FREE_ENERGY
-20.00 LEN_BP_RATIO 2.69565217391304
UGAAGCCUUCUUGUCAUCCUCAGUAUAAGAGAGUCCAGUCUCUCUGGUUUUCUCUGGGGCUUUCA
GAGCUGG
>random_seq_from_cds__NO_6457 RANDOM_LENGTH 81 SEQ_LENGTH 70
GC_CONTENT 0.571428571428571 BASEPAIR 24 FREE_ENERGY
-28.90 LEN_BP_RATIO 2.91666666666667
GGAGGCGGAGUGCCUUGAUUCAAUCCUUUUGGCCACAUGUUCUGCUGGGGUCCAAGAAGGGCUCU
CUGCACUCUUACUCUU
>random_seq_from_cds__NO_6458 RANDOM_LENGTH 87 SEQ_LENGTH 63
GC_CONTENT 0.555555555555556 BASEPAIR 20 FREE_ENERGY
-20.00 LEN_BP_RATIO 3.15
GGCUCUCACGGGUUUCUGGCACUUGGCUGCACUCUCUUGUGGUUUAUUCAACUCCGCAAAUAGUU
CUGAGGAAAGUGGCCUGAAUUU
>random_seq_from_cds__NO_6468 RANDOM_LENGTH 85 SEQ_LENGTH 71
GC_CONTENT 0.647887323943662 BASEPAIR 23 FREE_ENERGY
-18.90 LEN_BP_RATIO 3.08695652173913
GGCCCGUUCCGAGGAAUUGUAAUAGCUCCCCAGGAAGACCAGGAUCCAGAAGCCGUUGCCCGCGG
ACAGCUCGCCCUUCUCCUUC
>random_seq_from_cds__NO_6469 RANDOM_LENGTH 85 SEQ_LENGTH 53
GC_CONTENT 0.716981132075472 BASEPAIR 21 FREE_ENERGY
-26.70 LEN_BP_RATIO 2.52380952380952
CUGUUCACGUUCUCCCUGCACACCCCCAGGGCCCAGCUGGUGCGGUCCCCAACCUCCACCUCCCA
GUAGUGGCGGCCUGAGGUGA
>random_seq_from_cds__NO_6477 RANDOM_LENGTH 77 SEQ_LENGTH 72
GC_CONTENT 0.75 BASEPAIR 23 FREE_ENERGY -32.40
LEN_BP_RATIO 3.1304347826087
GCUCCCCAGAGCGCUCGCAGGCCGCACACAGGAGGCGCAGCUCGUCGCCACAGAAGGCGGCCAGU
GGCUCGCGGUGC
>random_seq_from_cds__NO_6495 RANDOM_LENGTH 83 SEQ_LENGTH 72
GC_CONTENT 0.625 BASEPAIR 22 FREE_ENERGY -25.90
LEN_BP_RATIO 3.27272727272727
CUGAGGAGCUCAUCUACCCAGACCGCAGGCUGGAGACCAAGUAUCCGGUGGUGUCCGAUGGCAAG
AGGUUCCUGGGCUCUGGA
>random_seq_from_cds__NO_6500 RANDOM_LENGTH 86 SEQ_LENGTH 78
GC_CONTENT 0.615384615384615 BASEPAIR 28 FREE_ENERGY

-24.70 LEN_BP_RATIO 2.78571428571429
GGCAUUGGGGGAUGAAGCUCUGCCCACGGUCCUGGUCGGCGUGUUCAUCGAACAGCCCACGCCGU
UUGUGUCCCUGUUCUUCCAGC
>random_seq_from_cds__NO_6507 RANDOM_LENGTH 72 SEQ_LENGTH 58
GC_CONTENT 0.655172413793103 BASEPAIR 20 FREE_ENERGY
-20.40 LEN_BP_RATIO 2.9
CCCUGCGGGGUGAGCUGCAGUCCUCAGAUCUCUUCCACCACAGCAAGCUGGACCCCGACAUGGCC
UUCUGUG
>random_seq_from_cds__NO_6518 RANDOM_LENGTH 83 SEQ_LENGTH 59
GC_CONTENT 0.457627118644068 BASEPAIR 21 FREE_ENERGY
-23.12 LEN_BP_RATIO 2.80952380952381
AGUGACCUGGAUAUUCUGUUGUUAGCCCAGCAGGAAUAUGGGCACCUCUUGCUGUAGCGACAACA
GUAAAACUGCCACUCCCG
>random_seq_from_cds__NO_6519 RANDOM_LENGTH 110 SEQ_LENGTH 81
GC_CONTENT 0.592592592592593 BASEPAIR 27 FREE_ENERGY
-25.10 LEN_BP_RATIO 3
AUCCAGCACUGACUCGAAGUAGCGGCUCUGGAAUCCUGCCACCAGCCCAUUGUUGGAGCACGUCU
GGUACCCAUUCCAUGCCAGCCCUGUUGAUCUCCUCCCACCAGCAC
>random_seq_from_cds__NO_6520 RANDOM_LENGTH 81 SEQ_LENGTH 75
GC_CONTENT 0.573333333333333 BASEPAIR 24 FREE_ENERGY
-22.80 LEN_BP_RATIO 3.125
UCCGUGGGUUCCCCGAGGCUCUGUGGCGUGGGCAUGCAGGCGUAGUUCCAUUGUCUGUCAGAACC
UUCCUUCUUGCUGAAG
>random_seq_from_cds__NO_6531 RANDOM_LENGTH 68 SEQ_LENGTH 48
GC_CONTENT 0.458333333333333 BASEPAIR 19 FREE_ENERGY
-17.40 LEN_BP_RATIO 2.52631578947368
UUUUAAUUGGUGACGGAGCUGGUUUCAAAGUUGCAAUGGGAGCUUUUGAUAAAACCAGACCUGUA
GGU
>random_seq_from_cds__NO_6540 RANDOM_LENGTH 81 SEQ_LENGTH 63
GC_CONTENT 0.666666666666667 BASEPAIR 24 FREE_ENERGY
-28.80 LEN_BP_RATIO 2.625
CUGCUGCACACGGAUGGUCCCUUGGCCAGCUCCUGGCGCCACUACAUUGCCAUCAUGGGCUGCCG
CCCGCCAUCAGUGUUC
>random_seq_from_cds__NO_6541 RANDOM_LENGTH 87 SEQ_LENGTH 77
GC_CONTENT 0.688311688311688 BASEPAIR 20 FREE_ENERGY
-31.70 LEN_BP_RATIO 3.85
UUACCUGGUAGGCUCCCACAUGGCCGAGUUUCUGCAGACUGGUGGUGACCCUGAGUGGCUGCUGG
GCCUCCACCGGGCCCCCGAGAA
>random_seq_from_cds__NO_6543 RANDOM_LENGTH 75 SEQ_LENGTH 62
GC_CONTENT 0.661290322580645 BASEPAIR 24 FREE_ENERGY
-26.90 LEN_BP_RATIO 2.58333333333333
CAGGGCCUUGCUGAAGACCGGCGAGCACACUUGGUCCCUGGCCGAGCUCAUUCAGGCUCUGGUCC
UGCUCACCCA
>random_seq_from_cds__NO_6548 RANDOM_LENGTH 67 SEQ_LENGTH 53

GC_CONTENT 0.584905660377358 BASEPAIR 20 FREE_ENERGY -17.80 LEN_BP_RATIO 2.65
UCGGAUAUGAGGACUUCACUCGGAGAGGGGCUCAGGCACCCCCUACCUUCCGGGCCCAGGGAUUAUA
>random_seq_from_cds__NO_6549 RANDOM_LENGTH 89 SEQ_LENGTH 80 GC_CONTENT 0.6 BASEPAIR 32 FREE_ENERGY -39.30 LEN_BP_RATIO 2.5
CCUGGGAAGACCAUGGCUACUCGCUGAUCCAGCGGCUUUACCCUGAGGGUGGGCAGCUGCUGGAUGAGAAGUUCCAGGCAGCCUAUAGC
>random_seq_from_cds__NO_6557 RANDOM_LENGTH 87 SEQ_LENGTH 51 GC_CONTENT 0.529411764705882 BASEPAIR 19 FREE_ENERGY -17.20 LEN_BP_RATIO 2.68421052631579
CUUGACAGAUUCUGAUCUCCCGUAUGGCCGUGUGGUAUCUCUCCAGCUUCUCUCCCAACUUCACUGUGUUGUCUGGGAGCUUCAUGC
>random_seq_from_cds__NO_6558 RANDOM_LENGTH 110 SEQ_LENGTH 103 GC_CONTENT 0.553398058252427 BASEPAIR 38 FREE_ENERGY -40.80 LEN_BP_RATIO 2.71052631578947
UGGCACCUGCGAGUUAGGGUUGUUUCCGAGUUUUCUUUCUUGGGUUUCAUCCCGAAAGGAGAUGGUCCUGGGGCUGGAGCGUUUGAGGGAGCUGCUGUAGGUUCGCUGUG
>random_seq_from_cds__NO_6559 RANDOM_LENGTH 109 SEQ_LENGTH 92 GC_CONTENT 0.66304347826087 BASEPAIR 28 FREE_ENERGY -39.65 LEN_BP_RATIO 3.28571428571429
UGGGUGAGGACAUAUCUGCCUCUUCCUCCUUGCUGGGGAUUUUCACCCUGGAGUGUGACGGGUGGGAGGCGGGAGGCCACAGUCGGAGGGUCUGAAGCCCCCUGCUUUG
>random_seq_from_cds__NO_6568 RANDOM_LENGTH 90 SEQ_LENGTH 78 GC_CONTENT 0.628205128205128 BASEPAIR 28 FREE_ENERGY -38.10 LEN_BP_RATIO 2.78571428571429
CUGCCUCCGCUCCUGCCGUGUUCUGAGGAUGCUCUGGAUGUCCUCGUCCUCAUCUUUGGAGGCUGGGGGCAGUGGCUUGGGCACCUCUGC
>random_seq_from_cds__NO_6569 RANDOM_LENGTH 90 SEQ_LENGTH 85 GC_CONTENT 0.611764705882353 BASEPAIR 31 FREE_ENERGY -35.10 LEN_BP_RATIO 2.74193548387097
UUCUUCCACGCUCGGUAGUCCUCUCAGAAGCAGAGGCCUGCCGGUCUCCAUUCUGGCUGAGCCUGGGAGCCUCAUCGUCCGUGGUGGAGC
>random_seq_from_cds__NO_6571 RANDOM_LENGTH 88 SEQ_LENGTH 75 GC_CONTENT 0.613333333333333 BASEPAIR 28 FREE_ENERGY -33.00 LEN_BP_RATIO 2.67857142857143
CCUGCUGACAGCCAUCAUGGCGGCGGCAUUUGAAGCCUCGGGAGCCUUAGCAGCAGUGGCGACUGCUAUGCCGGCUGAGCAUGUGGCC
>random_seq_from_cds__NO_6576 RANDOM_LENGTH 86 SEQ_LENGTH 74 GC_CONTENT 0.378378378378378 BASEPAIR 22 FREE_ENERGY -18.55 LEN_BP_RATIO 3.36363636363636
GAUCUUGGCUCCUACAAGAGAAAUUGCUGUACAGAUACAUUCUGUUAUUACAGCCAUUGGAAUAAAAAUGGAAGGCUUAGAGUGUC

>random_seq_from_cds__NO_6595 RANDOM_LENGTH 81 SEQ_LENGTH 70 GC_CONTENT 0.428571428571429 BASEPAIR 25 FREE_ENERGY -18.80 LEN_BP_RATIO 2.8
UGGAUGAUCGUAUUUCUUUGGAACAACCACCAAAUGGAAGUGACACCCCCAAUCCAGAGAAAUAUCAAGAAUCACCUGGAA
>random_seq_from_cds__NO_6614 RANDOM_LENGTH 67 SEQ_LENGTH 58 GC_CONTENT 0.620689655172414 BASEPAIR 20 FREE_ENERGY -24.60 LEN_BP_RATIO 2.9
GCCAGAGCUGCGCGGUGAGGAAGACCAUCUCAGAUGGGGAUCCCCACAGUGUUCACCUGGUCCUUGA
>random_seq_from_cds__NO_6615 RANDOM_LENGTH 90 SEQ_LENGTH 70 GC_CONTENT 0.628571428571429 BASEPAIR 25 FREE_ENERGY -26.00 LEN_BP_RATIO 2.8
GUCCCAGCAGGCUGUAGGCGAUGCGCUUCUGGUGGCCGGGCAGCCGCACCCCAAUCCUCUUGAUGUCGCUCGUUGGUCAUCUGCACCACC
>random_seq_from_cds__NO_6619 RANDOM_LENGTH 72 SEQ_LENGTH 52 GC_CONTENT 0.711538461538462 BASEPAIR 21 FREE_ENERGY -28.70 LEN_BP_RATIO 2.47619047619048
GGCGGGCACGCUCCUGCUGCCAGCACUGCAUCAUGAGCUGGUAGAUGGCGGAGGGGCAGUCCAUGGGUGUGG
>random_seq_from_cds__NO_6629 RANDOM_LENGTH 66 SEQ_LENGTH 58 GC_CONTENT 0.672413793103448 BASEPAIR 21 FREE_ENERGY -16.60 LEN_BP_RATIO 2.76190476190476
AAUCACCGCCAAGUUGCCAGAUCCCUCCGGGGCACAGCGUCUGGAAUUCGUGCACCUUGCUGCCGG
>random_seq_from_cds__NO_6632 RANDOM_LENGTH 67 SEQ_LENGTH 50 GC_CONTENT 0.64 BASEPAIR 19 FREE_ENERGY -23.10 LEN_BP_RATIO 2.63157894736842
GAGACGCUAAGCGAGGUGGUGCUGCGGCCCUCCAGCCUCACCUUGGGGGGCUCCUGUCUGGUUGAUG
>random_seq_from_cds__NO_6642 RANDOM_LENGTH 84 SEQ_LENGTH 73 GC_CONTENT 0.671232876712329 BASEPAIR 26 FREE_ENERGY -35.40 LEN_BP_RATIO 2.80769230769231
CCUGGAAGGCCAGGUAGAAGCCUUUGCGGGUGAGCGGCCCCACGGAGCGCUCCUCCACGUUCAGCUUCACGUGGCGUGCCUCGA
>random_seq_from_cds__NO_6657 RANDOM_LENGTH 75 SEQ_LENGTH 60 GC_CONTENT 0.566666666666667 BASEPAIR 20 FREE_ENERGY -23.90 LEN_BP_RATIO 3
AACUCCACGUUUUCUGCUACCUGGAACCGAGGAGUCUCUUCUCCUUCAAGUCCGUGGUUCUGGUGGAGCCCGACU
>random_seq_from_cds__NO_6663 RANDOM_LENGTH 86 SEQ_LENGTH 56 GC_CONTENT 0.5 BASEPAIR 23 FREE_ENERGY -20.50 LEN_BP_RATIO 2.43478260869565
UGUCAAGAAUUUGGCCUGGGUGGACUCAGACCAGCUCCUCUAUCAGCAUUUUUGGUGUCUCCCAG

UGAUCAAAAAGAGAGUGGUUG
>random_seq_from_cds__NO_6666 RANDOM_LENGTH 72 SEQ_LENGTH 64 GC_CONTENT 0.703125 BASEPAIR 20 FREE_ENERGY -20.70 LEN_BP_RATIO 3.2
UGCAGCGUUCAGCCAGCUCCACCUGCGCCGUGUGGAAGCCGCGGCCACAGCACAGCAGCUCACAGCCGUCGA
>random_seq_from_cds__NO_6667 RANDOM_LENGTH 82 SEQ_LENGTH 78 GC_CONTENT 0.666666666666667 BASEPAIR 29 FREE_ENERGY -36.20 LEN_BP_RATIO 2.68965517241379
UGGCCUUGGACGUCUUGUUGCAUGUGCGGCCCCUCGUGCCCAGCACGCCGCUGCGCAUGUCCUGCUCACAGAAGUCGGGGCU
>random_seq_from_cds__NO_6683 RANDOM_LENGTH 86 SEQ_LENGTH 67 GC_CONTENT 0.552238805970149 BASEPAIR 24 FREE_ENERGY -25.00 LEN_BP_RATIO 2.79166666666667
CUUUACCUGGUGACUGUUCUCCCUUGGUCACUCGACGGGAAUCCUCCAGCUGAGUGAUGAUGGUCUGCACACGGUCAUUCCCCGCC
>random_seq_from_cds__NO_6687 RANDOM_LENGTH 86 SEQ_LENGTH 80 GC_CONTENT 0.6625 BASEPAIR 30 FREE_ENERGY -40.90 LEN_BP_RATIO 2.66666666666667
AUGAUCACCUCGUGGCGGCAGGUGGGGCAGCGGAAACGGCCUCCAGACAUGGACACUGAGCUGCCCCGGCUGGUCCAGUAGGGAUU
>random_seq_from_cds__NO_6721 RANDOM_LENGTH 110 SEQ_LENGTH 75 GC_CONTENT 0.546666666666667 BASEPAIR 29 FREE_ENERGY -33.30 LEN_BP_RATIO 2.58620689655172
AGCACACGAUGUAUUUUGCCUGAUUUAGAUCUGGGGGCUGGUGGGGGCCAUAGCCUUGGCAGCUUGCUGGGCUAACCAGUACUUAUAUCUUUUGGUCUUGGGCUUCUCAA
>random_seq_from_cds__NO_6731 RANDOM_LENGTH 72 SEQ_LENGTH 54 GC_CONTENT 0.703703703703704 BASEPAIR 21 FREE_ENERGY -34.01 LEN_BP_RATIO 2.57142857142857
CCUCUCCAGCCUCCUGAGGACCCGGGGCUGCGUGAGCGAGCAGAGGCUUAAGGUCUUCAGCGGGGCGCUCCA
>random_seq_from_cds__NO_6734 RANDOM_LENGTH 78 SEQ_LENGTH 61 GC_CONTENT 0.688524590163934 BASEPAIR 21 FREE_ENERGY -30.10 LEN_BP_RATIO 2.9047619047619
UCCUACCGCAUUGGGGAGGACGGCUCCAUCUGCGUCUUGUACGAGGAGGCCCCACUGGCCGCCUCCUGUGGGCUCCUC
>random_seq_from_cds__NO_6751 RANDOM_LENGTH 68 SEQ_LENGTH 53 GC_CONTENT 0.452830188679245 BASEPAIR 19 FREE_ENERGY -15.50 LEN_BP_RATIO 2.78947368421053
AAGAAGUUCCAGCACAAGACUUGAGAAAACUCUGUGAGAGACUCAGGGGUAUGGAUUCUAGCACUCCC
>random_seq_from_cds__NO_6754 RANDOM_LENGTH 83 SEQ_LENGTH 71 GC_CONTENT 0.366197183098592 BASEPAIR 24 FREE_ENERGY -16.30 LEN_BP_RATIO 2.95833333333333

UGAUGAAGCUUAUGACCUGCUUGAUAAACUUCUAGAUCUAAAUCCAGCUUCAAGAAUAACAGCAG
AAGAAGCUUUGUUGCAUC
>random_seq_from_cds__NO_6758 RANDOM_LENGTH 87 SEQ_LENGTH 80
GC_CONTENT 0.5375 BASEPAIR 26 FREE_ENERGY -20.62
LEN_BP_RATIO 3.07692307692308
AUUUGGAUGGAGAGCUCUGUCUAGGGGCUGGGGUUGGAGCGUGAGUUGGUCGAUGGUGUGGUAGA
AGUCACAGGGGUCAAAUUCAAC
>random_seq_from_cds__NO_6763 RANDOM_LENGTH 72 SEQ_LENGTH 54
GC_CONTENT 0.722222222222222 BASEPAIR 19 FREE_ENERGY
-23.70 LEN_BP_RATIO 2.84210526315789
UGCUGAGUGGCAGGAAAGAGACCCGGCUGGCCCCAUGCGAUGGGCUGAGCCCCCGGCAUCACCUG
AGCGACU
>random_seq_from_cds__NO_6768 RANDOM_LENGTH 110 SEQ_LENGTH 86
GC_CONTENT 0.476744186046512 BASEPAIR 31 FREE_ENERGY
-26.60 LEN_BP_RATIO 2.7741935483871
UCACGGAUUGGCUCGUGUCCAGCCUCAAACACAAUGUACCUGGUACACAGGAUCUUCAACAUCCU
CUUCCAAUAUUGUCCUGGUACACAGCUUGUUCACACUGCUUAUCC
>random_seq_from_cds__NO_6770 RANDOM_LENGTH 87 SEQ_LENGTH 78
GC_CONTENT 0.538461538461538 BASEPAIR 20 FREE_ENERGY
-19.55 LEN_BP_RATIO 3.9
CAGCCCGCCUGGGCUGUUUUUAUGGCCACAAAUCUGUGAUUCCCUUCCUUCCCACAAACAUAUCC
AAAGGCCCGGUGAUCUGUAAUG
>random_seq_from_cds__NO_6774 RANDOM_LENGTH 72 SEQ_LENGTH 61
GC_CONTENT 0.524590163934426 BASEPAIR 20 FREE_ENERGY
-17.21 LEN_BP_RATIO 3.05
AAAGUGGCUUCACUGCGAUCCUGACCCUUUCUUUCUGGAGUCUUUCUUGGCGCUGGUUUUCACAG
CUACUUG
>random_seq_from_cds__NO_6789 RANDOM_LENGTH 72 SEQ_LENGTH 59
GC_CONTENT 0.576271186440678 BASEPAIR 20 FREE_ENERGY
-20.34 LEN_BP_RATIO 2.95
UACCGGGUGGACCAUUACUUAGGCAAGCAGGGCUGUGGCGCAGAUCCUGCCUUUCCGAGACCAGA
ACCGCAA
>random_seq_from_cds__NO_6790 RANDOM_LENGTH 68 SEQ_LENGTH 60
GC_CONTENT 0.616666666666667 BASEPAIR 20 FREE_ENERGY
-20.80 LEN_BP_RATIO 3
GGCUUUGGACGGCCUCUGGAACCGGCACCAUGUGGAGCGGGUGGAGAUCAUCAUGAAAGAGACCG
UGG
>random_seq_from_cds__NO_6793 RANDOM_LENGTH 85 SEQ_LENGTH 77
GC_CONTENT 0.61038961038961 BASEPAIR 29 FREE_ENERGY
-28.00 LEN_BP_RATIO 2.6551724137931
AGUCUUACAGUGAGCAGGUGCGCAGAGAGCUGCAGAAGCCAGACAGCUUCCACAGCCUGACGCCG
ACCUUCGCAGGUGGUCCUAG
>random_seq_from_cds__NO_6806 RANDOM_LENGTH 87 SEQ_LENGTH 62
GC_CONTENT 0.725806451612903 BASEPAIR 19 FREE_ENERGY

-23.81 LEN_BP_RATIO 3.26315789473684
CCCACAGUCACCCACUGGCCUGGAUGGCGAGCAGCUGGUCGUGCUGACCACGAGCCCCUCCCAGC
CACACCGCCGCAUGAGCCUUAG
>random_seq_from_cds__NO_6819 RANDOM_LENGTH 62 SEQ_LENGTH 55
GC_CONTENT 0.654545454545455 BASEPAIR 19 FREE_ENERGY
-17.80 LEN_BP_RATIO 2.89473684210526
ACGGGGAAUGACAUGCCAAGCUCGGAGCUCCUACCUGGUAGACGAGGUGCUGUGGGGCCACC
>random_seq_from_cds__NO_6822 RANDOM_LENGTH 92 SEQ_LENGTH 55
GC_CONTENT 0.763636363636364 BASEPAIR 20 FREE_ENERGY
-30.40 LEN_BP_RATIO 2.75
GGUGGAGGAGGAGGGGGCGGGGGAGGGGGCGGGUGGGGAAGCUGGGGCUGACAAGGAGCAGAAUG
GCUGCCUGCCACCCCCAGAGAGUGAGU
>random_seq_from_cds__NO_6825 RANDOM_LENGTH 88 SEQ_LENGTH 56
GC_CONTENT 0.517857142857143 BASEPAIR 21 FREE_ENERGY
-19.10 LEN_BP_RATIO 2.66666666666667
CCACCACUGCCUCCACUACCGCUGCCACCCCUGAAGAAGAGAGGGAACCACAGCACAGGCCUGUG
UCUCCUUGUGAUGUUUUUCAUGG
>random_seq_from_cds__NO_6830 RANDOM_LENGTH 81 SEQ_LENGTH 67
GC_CONTENT 0.567164179104478 BASEPAIR 23 FREE_ENERGY
-21.00 LEN_BP_RATIO 2.91304347826087
AACAACCUGCCCCUGAGCCACAAGGUCUACAUGAGGAACUCUAAGUAUCCCCAGGAUCUGGUGAU
GAUGGAGGGGAAGAUG
>random_seq_from_cds__NO_6831 RANDOM_LENGTH 65 SEQ_LENGTH 56
GC_CONTENT 0.589285714285714 BASEPAIR 21 FREE_ENERGY
-25.60 LEN_BP_RATIO 2.66666666666667
AUGAGCUACUGCACUACUGGGCAGAUGUGGGCCCGCAGCAGCUACCUGGGGGCAGUGUUCAAUCU
>random_seq_from_cds__NO_6832 RANDOM_LENGTH 98 SEQ_LENGTH 77
GC_CONTENT 0.402597402597403 BASEPAIR 26 FREE_ENERGY
-19.60 LEN_BP_RATIO 2.96153846153846
UACCAGUGCUGAUCAUUUAUAUGUCAACGUAUCUGAGCUCUCUCUGGUCAAUUUUGAGGAAUCUC
AGACGUUUUUCGGCUUAUAUAAGCUCUAACUCA
>random_seq_from_cds__NO_6835 RANDOM_LENGTH 79 SEQ_LENGTH 60
GC_CONTENT 0.55 BASEPAIR 19 FREE_ENERGY -24.90
LEN_BP_RATIO 3.15789473684211
AAGAGGGGCUCCUCAUCCUUCUGGUAAUGAAGGCUAAUGUUGACUUCCUGGGAGAAGUAGCCCUU
CAGGGAGAUGAGAU
>random_seq_from_cds__NO_6861 RANDOM_LENGTH 79 SEQ_LENGTH 54
GC_CONTENT 0.481481481481481 BASEPAIR 20 FREE_ENERGY
-16.90 LEN_BP_RATIO 2.7
UAGAUGAGGAGGAAGGAUGGGUGGGGAUUUGAAUCUUUCCUCAGAUCGAAACGCAUACAUUUCUU
GACCAUAAGGCCCU
>random_seq_from_cds__NO_6874 RANDOM_LENGTH 82 SEQ_LENGTH 73
GC_CONTENT 0.657534246575342 BASEPAIR 25 FREE_ENERGY
-28.40 LEN_BP_RATIO 2.92

CAGGCUGCAGCUGGACAGAGUGGCAGAGGCGGCUGAGGCUUGGGCAGCCAGGUCCCGGUAAGCAG
GCGACUUCAGGAAGCGU
>random_seq_from_cds__NO_6880 RANDOM_LENGTH 84 SEQ_LENGTH 54
GC_CONTENT 0.62962962962963 BASEPAIR 19 FREE_ENERGY
-22.70 LEN_BP_RATIO 2.84210526315789
AUUUGUGAAGAAAGAUCCCCAGACGUGUCUUGAACUCUUUGGCUCCUCUCCAGGCAGGUGGUGGG
GAAGGCGGCCAGGGUGCGG
>random_seq_from_cds__NO_6884 RANDOM_LENGTH 84 SEQ_LENGTH 65
GC_CONTENT 0.661538461538462 BASEPAIR 23 FREE_ENERGY
-27.30 LEN_BP_RATIO 2.82608695652174
AGGAUGCUGUGGGCUCCAUGAUGCUAGGGUCGGUGCUGCUGCUGGGCUGGGCAGAGUCAGGGCUG
GGGCAGAGGGUGGAGGUAG
>random_seq_from_cds__NO_6886 RANDOM_LENGTH 62 SEQ_LENGTH 50
GC_CONTENT 0.66 BASEPAIR 19 FREE_ENERGY -19.60
LEN_BP_RATIO 2.63157894736842
GUUGUCCCUCAGUCCUGUCGGGCACUUCCAGUCUCGUCUGCGGAGGGGCCUUGACGGCAAUC
>random_seq_from_cds__NO_6887 RANDOM_LENGTH 110 SEQ_LENGTH 98
GC_CONTENT 0.530612244897959 BASEPAIR 35 FREE_ENERGY
-35.40 LEN_BP_RATIO 2.8
ACUGUCUGCUCCUUAAAGUUGCCAACAGCACGGAUAUCCUGGUAAGUCACAUAGGCCAGCUCUCU
UGUUGGCCUUGUCCUCAGUCAGGUGCUUGAAGCUCAGAGAGCAGC
>random_seq_from_cds__NO_6893 RANDOM_LENGTH 81 SEQ_LENGTH 72
GC_CONTENT 0.694444444444444 BASEPAIR 25 FREE_ENERGY
-35.80 LEN_BP_RATIO 2.88
AUCCAGCUUCCUUUUGGCCCGGCAGCCGGCCUGCCGGCAGGCAUCGCACAACUUGGCCCUCGGGU
CCGUGGGGAGUGGCGU
>random_seq_from_cds__NO_6894 RANDOM_LENGTH 81 SEQ_LENGTH 64
GC_CONTENT 0.6875 BASEPAIR 23 FREE_ENERGY -34.70
LEN_BP_RATIO 2.78260869565217
CGAGGCAGGUGCCUGGCGCCGCUGCGGGAGGCGCCGUCUGCGGGUACAGCGGUGUGUAGUAGGUA
GCAGUAGCUGGGCAGA
>random_seq_from_cds__NO_6898 RANDOM_LENGTH 110 SEQ_LENGTH 83
GC_CONTENT 0.373493975903614 BASEPAIR 30 FREE_ENERGY
-23.20 LEN_BP_RATIO 2.76666666666667
CCUUGCAUUUGAUAUGUCUGUGUUCCAUCCUGGGAGAGUCUUAUAUUGAACUUCAACUUUAUUUA
AGACUUCUUGGUUUGCCUGGGAUAUGAGGUAUGAUUUCACCAUCU
>random_seq_from_cds__NO_6919 RANDOM_LENGTH 63 SEQ_LENGTH 50
GC_CONTENT 0.62 BASEPAIR 19 FREE_ENERGY -18.50
LEN_BP_RATIO 2.63157894736842
GCAUGACCAUCGCUGCCUACCAGACCCUGUACGAGAGCAGCGUGGCGUUUGGCAUGAGGAAGG
>random_seq_from_cds__NO_6922 RANDOM_LENGTH 73 SEQ_LENGTH 55
GC_CONTENT 0.545454545454545 BASEPAIR 19 FREE_ENERGY
-16.90 LEN_BP_RATIO 2.89473684210526
GAAGCACAAUGAGGAGAUUCAGUUCCUGAAGCGAACAAAUCAGCAGCUGAAGGGCCCAACUGGAA

GGCAUUAU
>random_seq_from_cds__NO_6923 RANDOM_LENGTH 99 SEQ_LENGTH 81 GC_CONTENT 0.444444444444444 BASEPAIR 26 FREE_ENERGY -18.00 LEN_BP_RATIO 3.11538461538462
UGCACCAAAGAAGUGAAAUGUCCCUGCUCUUCUCUCGAUGCAACUCUAUCGUCACAGUCAAGAAA
AAUAAGAGACACAUGGCUGAGGUGAAUGCAUCCC
>random_seq_from_cds__NO_6932 RANDOM_LENGTH 97 SEQ_LENGTH 85 GC_CONTENT 0.647058823529412 BASEPAIR 32 FREE_ENERGY -38.30 LEN_BP_RATIO 2.65625
UGCAUCUGCCUCAGAGCCCGAGUACAUGGAGGAGGGUGCGGCGGCAGCACAUGGAGCGUUGUACC
AGCUUCCUGGUGGAUGAGCUGGGCGUGGUGGA
>random_seq_from_cds__NO_6937 RANDOM_LENGTH 72 SEQ_LENGTH 59 GC_CONTENT 0.542372881355932 BASEPAIR 20 FREE_ENERGY -18.30 LEN_BP_RATIO 2.95
CUGGAGCUCUUGGCUCAAGACUAUAAGCUGCGAAUUAAGCAGAUUACGGAGGAAGUGGAGAGGCA
GGGUGUC
>random_seq_from_cds__NO_6940 RANDOM_LENGTH 68 SEQ_LENGTH 51 GC_CONTENT 0.568627450980392 BASEPAIR 19 FREE_ENERGY -19.90 LEN_BP_RATIO 2.68421052631579
UCCACGGCCAUCACCAACUCCCUGCAGACCAUGCAGCAGGACAUGAUAGGAUGGCUUGAAACCCC
UCC
>random_seq_from_cds__NO_6955 RANDOM_LENGTH 78 SEQ_LENGTH 60 GC_CONTENT 0.466666666666667 BASEPAIR 24 FREE_ENERGY -20.30 LEN_BP_RATIO 2.5
GUGAAAAUGAUGUUAAGUGGUGUGUGCUUGUUUGCUGUUUUCAUAGCUUUGCUGCCCAUCCUUGG
ACAUCGAGACUAU
>random_seq_from_cds__NO_6958 RANDOM_LENGTH 110 SEQ_LENGTH 81 GC_CONTENT 0.481481481481481 BASEPAIR 29 FREE_ENERGY -23.60 LEN_BP_RATIO 2.79310344827586
AAGAGUUAAAUUUAAAAGUCAGCAGCACAGACAAGGCAGAUCUCAUCAUUUGGAAAUGGUAAUCC
AGCUCCUGGCGAUAAUGUGUGUCUCCUGUAUUUGUUGGAGCCCAU
>random_seq_from_cds__NO_6969 RANDOM_LENGTH 78 SEQ_LENGTH 55 GC_CONTENT 0.618181818181818 BASEPAIR 21 FREE_ENERGY -26.40 LEN_BP_RATIO 2.61904761904762
CCCGCACGUGUACCGUUUCUCCUCAUAAGAGGUAUCCGUGAACUCCAGGAGCAGGCGGAUGGCGU
GCGCCAGCCCCAC
>random_seq_from_cds__NO_6979 RANDOM_LENGTH 110 SEQ_LENGTH 72 GC_CONTENT 0.347222222222222 BASEPAIR 24 FREE_ENERGY -16.50 LEN_BP_RATIO 3
AAAUUAAUGACAUAUUUCAAAAACUCAACAUAUUUGAUCAGUCUUUUUAUGAUCUAUCGCUGCAA
ACCAGUGAAAUCAAAGAAGAAGAAAAGGAACUGAGAAGAACUACA
>random_seq_from_cds__NO_6985 RANDOM_LENGTH 89 SEQ_LENGTH 58 GC_CONTENT 0.46551724137931 BASEPAIR 22 FREE_ENERGY -19.30 LEN_BP_RATIO 2.63636363636364

GGCAUUCCUGCUGAAUGUACCACCAUUUAUAACAGAGGUGAACAUACAAGUGGCAUGUAUGCCAU
CAGACCCAGCAACUCUCAAGUUUU
>random_seq_from_cds__NO_6989 RANDOM_LENGTH 86 SEQ_LENGTH 60 GC_CONTENT 0.433333333333333 BASEPAIR 21 FREE_ENERGY -18.40 LEN_BP_RATIO 2.85714285714286
UACUGGCAAUGUCCCCAAUGCAAUCCCGGAAAACAAAGAUUUGGUGUUUUCUACUUGGGAUCACA
AAGCAAAAGGACACUUCAACU
>random_seq_from_cds__NO_7016 RANDOM_LENGTH 62 SEQ_LENGTH 57 GC_CONTENT 0.614035087719298 BASEPAIR 22 FREE_ENERGY -26.10 LEN_BP_RATIO 2.59090909090909
CAGCAGAGUUAGGGGUGCCUCCCUCCAGGGGCUGGAGGUGGAGUUCAGCCCCCGAUAUUGU
>random_seq_from_cds__NO_7025 RANDOM_LENGTH 73 SEQ_LENGTH 60 GC_CONTENT 0.616666666666667 BASEPAIR 21 FREE_ENERGY -22.70 LEN_BP_RATIO 2.85714285714286
CACCACCCACUACUACCUGGUCAUGCAGCUGUGUUUCUGGUGGGGAGCUCUUUGACCGGAUCCUG
GAGCGGGG
>random_seq_from_cds__NO_7026 RANDOM_LENGTH 94 SEQ_LENGTH 86 GC_CONTENT 0.5 BASEPAIR 27 FREE_ENERGY -23.90 LEN_BP_RATIO 3.18518518518519
UGUCUACACAGAGAAGGAUGCCAGUCUGGUGAUCCAGCAGGUCUUGUCGGCAGUGAAAUACCUAC
AUGAGAAUGGCAUCGUCCACAGAGACUUA
>random_seq_from_cds__NO_7035 RANDOM_LENGTH 110 SEQ_LENGTH 80 GC_CONTENT 0.7 BASEPAIR 25 FREE_ENERGY -31.60 LEN_BP_RATIO 3.2
CUGACCCAAUUACCCUGCCAGCAUGGCCGCCGGCCCACUGCCCCUGGUGGCAGGUCCCUCAACUG
CCUGGUCAAUGGCUCCCUCCACAUCAGCAGCAGCCUGGUGCCCAU
>random_seq_from_cds__NO_7039 RANDOM_LENGTH 79 SEQ_LENGTH 67 GC_CONTENT 0.597014925373134 BASEPAIR 25 FREE_ENERGY -27.70 LEN_BP_RATIO 2.68
GAUCCUGUCUGGCUCGCUUAGGAUUCUGCAGGUCACCAGGUGGUGAGGCCUUGCUUGCUGAGAUG
CUCCGGAGAGAAUG
>random_seq_from_cds__NO_7049 RANDOM_LENGTH 102 SEQ_LENGTH 96 GC_CONTENT 0.635416666666667 BASEPAIR 35 FREE_ENERGY -37.20 LEN_BP_RATIO 2.74285714285714
GUCAGGGACUUAUCAAUGGCCAGAAUGUCACAGGCCCUUGCUCCUCAGGUCUUCGAGGCAGGAGG
CGGCCACGCCGUGCUGUUCGCGGCUGUAGUUGCGGCG
>random_seq_from_cds__NO_7051 RANDOM_LENGTH 96 SEQ_LENGTH 91 GC_CONTENT 0.538461538461538 BASEPAIR 31 FREE_ENERGY -26.60 LEN_BP_RATIO 2.93548387096774
GCUGUGGCUGAAGAGGCUCUGGGUGCUCUUGGAGGUGGCUGUGCAUGUGGUCGUGGGUAAAGUGC
UUCUGAUAUUGUUUCCAGACAGAGUCAAGCG
>random_seq_from_cds__NO_7053 RANDOM_LENGTH 79 SEQ_LENGTH 65 GC_CONTENT 0.553846153846154 BASEPAIR 23 FREE_ENERGY -26.90 LEN_BP_RATIO 2.82608695652174

CCCAGUAUUUCUGGUUCGUCUUGAAGGUCCGUUGGCAGCGACUAGAGGACACGACUGAGCUAGGG
GGUCUGGCCCCAAA
>random_seq_from_cds__NO_7066 RANDOM_LENGTH 72 SEQ_LENGTH 61
GC_CONTENT 0.639344262295082 BASEPAIR 19 FREE_ENERGY
-21.70 LEN_BP_RATIO 3.21052631578947
UCCCGCUGCUCCUCAACCCUGCGCAGCUGAAAACCCUUGACGGAUGGCUGAAAGCAGGUCGCUAC
GGGCAUC
>random_seq_from_cds__NO_7072 RANDOM_LENGTH 73 SEQ_LENGTH 56
GC_CONTENT 0.517857142857143 BASEPAIR 19 FREE_ENERGY
-19.60 LEN_BP_RATIO 2.94736842105263
AUCUUUUGGGUCCAGCCAAACCAGAUCCUCUUUGGUUGUCCACUGGGUAACCUGAAUUCUGCUGG
UGGAGGAG
>random_seq_from_cds__NO_7076 RANDOM_LENGTH 110 SEQ_LENGTH 68
GC_CONTENT 0.397058823529412 BASEPAIR 24 FREE_ENERGY
-19.95 LEN_BP_RATIO 2.83333333333333
CUAUGCUUUCUCUUCUCUUUCAUGAUAUCCUUGGUGUCCUGCAGCAUCUUCUCCUUCCAAAGAUC
AAAGAAGUAUGAAGGGUCUGUGUAGAAUUUGAGUGCCUCUUUUCC
>random_seq_from_cds__NO_7079 RANDOM_LENGTH 87 SEQ_LENGTH 76
GC_CONTENT 0.552631578947368 BASEPAIR 25 FREE_ENERGY
-24.90 LEN_BP_RATIO 3.04
CCUUCUUCUUCCUUGGGAUCCAGCUGAGUGACUUUAACCUGUAGUCGGUCGACCCUCUCAGCAAG
GGAGCUUACCCGAGAGGCAAAG
>random_seq_from_cds__NO_7092 RANDOM_LENGTH 90 SEQ_LENGTH 72
GC_CONTENT 0.680555555555556 BASEPAIR 27 FREE_ENERGY
-33.20 LEN_BP_RATIO 2.66666666666667
GUGGAAGGUAGUGACCAGCACAGCCAGCGCCUGCUCCAGAGAACUGCACAUCAUGCGGCUGGCGC
UGCUCUGGGCCCUGGGGCUCCUGGG
>random_seq_from_cds__NO_7093 RANDOM_LENGTH 73 SEQ_LENGTH 57
GC_CONTENT 0.701754385964912 BASEPAIR 21 FREE_ENERGY
-28.70 LEN_BP_RATIO 2.71428571428571
CGCGGGCAGCCCUCUGCCUUCCUGGCCGCUCCCAAAUAUAGGGUGGCACUGAGGAGCAGCAGGCA
GAGUCAGA
>random_seq_from_cds__NO_7098 RANDOM_LENGTH 100 SEQ_LENGTH 91
GC_CONTENT 0.571428571428571 BASEPAIR 24 FREE_ENERGY
-26.75 LEN_BP_RATIO 3.79166666666667
GGUCCUCCCAUUAUUUCGCGAAUCCAAGAUCUCCACCUGCCAGGCCACACCUGUGCCCUGAGCUG
GCGGGAAUCUGUACACACUCAGAAGCCACCAGAGC
>random_seq_from_cds__NO_7101 RANDOM_LENGTH 94 SEQ_LENGTH 74
GC_CONTENT 0.662162162162162 BASEPAIR 26 FREE_ENERGY
-26.20 LEN_BP_RATIO 2.84615384615385
AAUGUACGAGUGGCACUAGUGGGCCUGGAGGCCUGGACCCAGCGUGACCUGGUGGAGAUCAGCCC
AAACCCAGCUGUCACCCUCGAAAACUUCC
>random_seq_from_cds__NO_7114 RANDOM_LENGTH 84 SEQ_LENGTH 73
GC_CONTENT 0.534246575342466 BASEPAIR 28 FREE_ENERGY

-29.30 LEN_BP_RATIO 2.60714285714286
AUAGACCAUCGAUGCCAGCGUGUGGAUCUCCUGGGGGCACAGGAAUGUCGAAGCAAAUGCCAUGG
ACAUGGGGGUCUGUGACAG
>random_seq_from_cds__NO_7120 RANDOM_LENGTH 98 SEQ_LENGTH 78
GC_CONTENT 0.641025641025641 BASEPAIR 27 FREE_ENERGY
-35.80 LEN_BP_RATIO 2.88888888888889
AGUGUCCUCGCUCUACCUCUGAGAUGGCGGACGGGGAGCUGAACGUGGACAGCCUCAUCACCCGG
CUGCUGGAGGGUACGAGGAUGUCGUCCAGGAAA
>random_seq_from_cds__NO_7150 RANDOM_LENGTH 89 SEQ_LENGTH 75
GC_CONTENT 0.466666666666667 BASEPAIR 19 FREE_ENERGY
-18.30 LEN_BP_RATIO 3.94736842105263
GCAGUCUCUUGGUACUUUUGAUGCACAGCGUUUAUGGCAGUUGAAUUUGCAAUCCUUUACACUGC
AUUCCUUGGCGAAAGAGGCCUUUC
>random_seq_from_cds__NO_7153 RANDOM_LENGTH 79 SEQ_LENGTH 65
GC_CONTENT 0.492307692307692 BASEPAIR 19 FREE_ENERGY
-16.40 LEN_BP_RATIO 3.42105263157895
GUCUUGGAACUGAGAGGCCGGGUCCUGGUAAAGAUACAUUUGACAGACGUCUCUUUCUUACUCCA
CUACAGUUAUUUGG
>random_seq_from_cds__NO_7179 RANDOM_LENGTH 68 SEQ_LENGTH 61
GC_CONTENT 0.540983606557377 BASEPAIR 19 FREE_ENERGY
-16.10 LEN_BP_RATIO 3.21052631578947
GUGGAGAAGUGGGAGCACGCAGUCAUGGUAAACCAGGAGAAACGUGGACUUCAGGAACUCUUUGU
CUC
>random_seq_from_cds__NO_7203 RANDOM_LENGTH 73 SEQ_LENGTH 62
GC_CONTENT 0.483870967741936 BASEPAIR 23 FREE_ENERGY
-20.00 LEN_BP_RATIO 2.69565217391304
AGCAUGUUAGCCUAGGUUUGUUUGGGGCAAGCUGUCGACUUGGCCGUCUAAUAGACUGGUUUUGC
UGUAUCUU
>random_seq_from_cds__NO_7254 RANDOM_LENGTH 82 SEQ_LENGTH 71
GC_CONTENT 0.591549295774648 BASEPAIR 25 FREE_ENERGY
-23.80 LEN_BP_RATIO 2.84
GAACCGCCGUUCUUCUGACACUGGCUCCAAGGCACCCCCCACCUUCAAGGGUCUCACUUAUGGAC
CAGUCAGUAAGAGAAGG
>random_seq_from_cds__NO_7256 RANDOM_LENGTH 71 SEQ_LENGTH 64
GC_CONTENT 0.75 BASEPAIR 26 FREE_ENERGY -39.40
LEN_BP_RATIO 2.46153846153846
CCAGCGGCGCUUUGCGGAGGAGGCUGAGGGUGGGCUGUGCCGGCUGCGGAUCCUGGCUGCAGAGC
GUGGCG
>random_seq_from_cds__NO_7257 RANDOM_LENGTH 64 SEQ_LENGTH 50
GC_CONTENT 0.64 BASEPAIR 19 FREE_ENERGY -19.30
LEN_BP_RATIO 2.63157894736842
AUGCUGGUUUCUACACUUGCAAAGCGGUCAAUGAGUAUGGUGCUCGGCAGUGCGAGGCCCGCUU
>random_seq_from_cds__NO_7270 RANDOM_LENGTH 69 SEQ_LENGTH 63
GC_CONTENT 0.476190476190476 BASEPAIR 24 FREE_ENERGY

-18.60 LEN_BP_RATIO 2.625
CAGCUCAUCUGCAGUCACAUUUAGUGAAGAAACUAUAUGGUCUAUACUGCUGGCUCAUCAGGUGA
CUGG
>random_seq_from_cds__NO_7280 RANDOM_LENGTH 89 SEQ_LENGTH 76
GC_CONTENT 0.421052631578947 BASEPAIR 21 FREE_ENERGY
-17.50 LEN_BP_RATIO 3.61904761904762
UCGUUCUUGGUUACCAUGUAUAUUUUUUGCUGGAGGCAUAACAAUGGGAAACAUUGGUCGACAAC
UGGCAAUGGUACGAAUGUAAAGUU
>random_seq_from_cds__NO_7292 RANDOM_LENGTH 89 SEQ_LENGTH 80
GC_CONTENT 0.4875 BASEPAIR 29 FREE_ENERGY -19.20
LEN_BP_RATIO 2.75862068965517
CAUGGGCAGCUAUCGAUGUACCUGCAAAAUAGGAUUUGGGCCGGAUCCUACCUUUUCAAGUUGUG
UUCGCUGAUCCCCCUGUGAUCUCG
>random_seq_from_cds__NO_7293 RANDOM_LENGTH 78 SEQ_LENGTH 57
GC_CONTENT 0.526315789473684 BASEPAIR 20 FREE_ENERGY
-16.00 LEN_BP_RATIO 2.85
GAAGAGAAAGGGCCCUGUUACCGACUUGUCAGUUCUGGAAGACAGUGUAUGCACCCUCUGUCUGU
UCACCUCACCAAG
>random_seq_from_cds__NO_7295 RANDOM_LENGTH 79 SEQ_LENGTH 70
GC_CONTENT 0.485714285714286 BASEPAIR 23 FREE_ENERGY
-22.20 LEN_BP_RATIO 3.04347826086957
CGGUUUCUGGCGUUCAUAGACGCAGGCCAAUCCAUCACCAUGUAGGUAAAGGACCUGUAUUUGUC
AAGCCAAAGAACAC
>random_seq_from_cds__NO_7301 RANDOM_LENGTH 82 SEQ_LENGTH 72
GC_CONTENT 0.541666666666667 BASEPAIR 23 FREE_ENERGY
-24.00 LEN_BP_RATIO 3.1304347826087
GUUCAGUGAACAACAGAGGAAAUGUGUGGGAUAUUGAUGAGUGUACUCAGGUCCAACACCUCUGC
UCCCAGGGCCGCUGUGA
>random_seq_from_cds__NO_7309 RANDOM_LENGTH 70 SEQ_LENGTH 61
GC_CONTENT 0.524590163934426 BASEPAIR 22 FREE_ENERGY
-17.90 LEN_BP_RATIO 2.77272727272727
AUCUCUGUGCUCAUGGGCAGUGCAGGAACACUGAGGGCUCUUUUCAAUGUGUGUGUGACCAGGGU
UACAG
>random_seq_from_cds__NO_7314 RANDOM_LENGTH 88 SEQ_LENGTH 64
GC_CONTENT 0.59375 BASEPAIR 25 FREE_ENERGY -24.90
LEN_BP_RATIO 2.56
GCUGGCUCCUUCCGCUGCCUCUGUUAUCAGGGCUUUCAAGCCCCACAGGAUGGGCAAGGGUGUGU
GGGAUGUGAAUGAAUGUGAACUG
>random_seq_from_cds__NO_7321 RANDOM_LENGTH 87 SEQ_LENGTH 74
GC_CONTENT 0.459459459459459 BASEPAIR 27 FREE_ENERGY
-20.40 LEN_BP_RATIO 2.74074074074074
GAUGAAUGUCAAGACCCCAGUAGUUGUAUUGAUGGCCAGUGUGUUAAUACAGAGGGCUCUUACAA
CUGCUUCUGUACUCACCCCAUG
>random_seq_from_cds__NO_7326 RANDOM_LENGTH 74 SEQ_LENGTH 61

GC_CONTENT 0.409836065573771 BASEPAIR 20 FREE_ENERGY -18.40 LEN_BP_RATIO 3.05
GGUUUUCUAAAUAGCUUUGAGGAGUUACAGGCUGAGGAAUGCGGCAUCCUCAAUGGAUGUGAAAAUGGUCGCUG
>random_seq_from_cds__NO_7336 RANDOM_LENGTH 73 SEQ_LENGTH 65 GC_CONTENT 0.492307692307692 BASEPAIR 24 FREE_ENERGY -20.20 LEN_BP_RATIO 2.70833333333333
UUGGUAACCACUUCCUCUGCCAUCAAAGCUAGCUACUAUAAUGUUUUCUGUGCUUGCAAGGUAAGUGGCCCAG
>random_seq_from_cds__NO_7354 RANDOM_LENGTH 109 SEQ_LENGTH 82 GC_CONTENT 0.304878048780488 BASEPAIR 33 FREE_ENERGY -16.26 LEN_BP_RATIO 2.48484848484848
UUAUUUUCUUGUUUGUAGAGAUAUUCAUGAUCCUGAAAUCCAUCUUAAGGAGUAUAACUUCAGUCUAUAAGUAUUUUUUAAGUAAUCAGUUAGAGUGUAAGUUUUGCGA
>random_seq_from_cds__NO_7364 RANDOM_LENGTH 85 SEQ_LENGTH 76 GC_CONTENT 0.552631578947368 BASEPAIR 23 FREE_ENERGY -21.96 LEN_BP_RATIO 3.30434782608696
GGACCUGUAGUAGCGAUGCAGCACCAGGAUGAAGUCAGUGAUGGUCAGCAUCCCCCACAAAGCUCUGCUUCUUGCUGUCCCAUAG
>random_seq_from_cds__NO_7382 RANDOM_LENGTH 90 SEQ_LENGTH 73 GC_CONTENT 0.493150684931507 BASEPAIR 24 FREE_ENERGY -21.04 LEN_BP_RATIO 3.04166666666667
UGAUCCAAAAGAAGAUCACAAUUACAGUGCAAGUAGCAUGGCAGCACAGCGUUGUGCAUCCAGGUCUAGCGUGUCUUCCCUGUCUUCUGU
>random_seq_from_cds__NO_7407 RANDOM_LENGTH 85 SEQ_LENGTH 65 GC_CONTENT 0.553846153846154 BASEPAIR 24 FREE_ENERGY -24.00 LEN_BP_RATIO 2.70833333333333
GGCAUCUGUUUCUGCAGUUUGAGGUGUUAAGGCACGUGCCCUGGAACUCCAGCCCGUAGAUCACUGGUGCAUCGUCCUCCAUGAU
>random_seq_from_cds__NO_7412 RANDOM_LENGTH 72 SEQ_LENGTH 64 GC_CONTENT 0.40625 BASEPAIR 23 FREE_ENERGY -16.30 LEN_BP_RATIO 2.78260869565217
GACAGCACUUUUCAAAAACCUGGAUUCAGAAUAUGAACAACAUAAUCUCCUUCCCUUUGGACAGUUUGCUGA
>random_seq_from_cds__NO_7439 RANDOM_LENGTH 72 SEQ_LENGTH 64 GC_CONTENT 0.609375 BASEPAIR 22 FREE_ENERGY -21.30 LEN_BP_RATIO 2.90909090909091
GAAGCUCUGGGUCCUCUGUCCAAUGCUAUGGUCCUGCAGCCCCCUGCACCCAUGCCUAGGAAGUCGCAGGCA
>random_seq_from_cds__NO_7442 RANDOM_LENGTH 96 SEQ_LENGTH 88 GC_CONTENT 0.545454545454545 BASEPAIR 30 FREE_ENERGY -31.80 LEN_BP_RATIO 2.93333333333333
GCUGACUGAGAUGGACCUCGAGGAGGCGGGAAGAGUUUAAAGAACGCUGUACUCAGUGUGCUGCUGUCUCAUGGGGUCUUACUGAUGAAGGCAAAU

>random_seq_from_cds__NO_7453 RANDOM_LENGTH 82 SEQ_LENGTH 67 GC_CONTENT 0.402985074626866 BASEPAIR 22 FREE_ENERGY -17.30 LEN_BP_RATIO 3.04545454545455
UAGCUUAACUUGCCACGUGGUAAAAAUGACUGGAAUGGGAGAAGUGGAUUUUCUGACAUUUGAUC
CUAUAGCUAAAAUGGCA
>random_seq_from_cds__NO_7461 RANDOM_LENGTH 71 SEQ_LENGTH 61 GC_CONTENT 0.426229508196721 BASEPAIR 19 FREE_ENERGY -17.30 LEN_BP_RATIO 3.21052631578947
UCUCUUCUCCUUCCUGCUCAGAAUAAAGACUUCCCUUCUCCAUGAAGAAGUGAGCUUAGUUGAGA
AGAAAC
>random_seq_from_cds__NO_7465 RANDOM_LENGTH 87 SEQ_LENGTH 79 GC_CONTENT 0.493670886075949 BASEPAIR 29 FREE_ENERGY -20.10 LEN_BP_RATIO 2.72413793103448
GAGAGAAAACCCCCGCCGCUUUGUCAUCUUCCCCAUCGAGUACCAUGAUAUCUGGCAGAUGUAUA
AGAAGGCAGAGGCUUCCUUUUG
>random_seq_from_cds__NO_7476 RANDOM_LENGTH 72 SEQ_LENGTH 62 GC_CONTENT 0.451612903225806 BASEPAIR 21 FREE_ENERGY -16.40 LEN_BP_RATIO 2.95238095238095
AGAGGAUGGGAGUGAUGUCAAGUCCAACAGAGAAUUCUUUUACCUUGGAUGCUGACUUCUAAUCU
ACACAUU
>random_seq_from_cds__NO_7490 RANDOM_LENGTH 79 SEQ_LENGTH 62 GC_CONTENT 0.5 BASEPAIR 22 FREE_ENERGY -19.00 LEN_BP_RATIO 2.81818181818182
AAUCCUGUCCCGGUAGCAGCAAGGGUCUUCACGAUGGCUUUGCUAUCAUUACAUUUGACUGCAUA
AAAGGGGGUGACAC
>random_seq_from_cds__NO_7503 RANDOM_LENGTH 85 SEQ_LENGTH 79 GC_CONTENT 0.759493670886076 BASEPAIR 29 FREE_ENERGY -38.40 LEN_BP_RATIO 2.72413793103448
AGGCUGCGGGCACGCAGGGGCUGGCACACGGCUAGGCAGCGCUCGGCGCUCAGGCCUGCCACGCU
CAGCACCGUGGCGUAGGCGC
>random_seq_from_cds__NO_7511 RANDOM_LENGTH 77 SEQ_LENGTH 72 GC_CONTENT 0.638888888888889 BASEPAIR 22 FREE_ENERGY -21.20 LEN_BP_RATIO 3.27272727272727
UGCCAGCUGAAAAGGGGCUCUGUGGACAGGAUGAGAGGCCUGGACCCCAGCACGCCAGCCCAAGU
GAUCGCUCCCAG
>random_seq_from_cds__NO_7520 RANDOM_LENGTH 73 SEQ_LENGTH 55 GC_CONTENT 0.490909090909091 BASEPAIR 21 FREE_ENERGY -18.40 LEN_BP_RATIO 2.61904761904762
CAGCAAACAAGACGGAUUCUCCUUCCAGGAAAAGAGGAUAAACGAAGCCGACAUCUUGGUGCUGA
CGGCGUCU
>random_seq_from_cds__NO_7534 RANDOM_LENGTH 99 SEQ_LENGTH 86 GC_CONTENT 0.383720930232558 BASEPAIR 29 FREE_ENERGY -15.10 LEN_BP_RATIO 2.96551724137931
UUGAAAAGAAGCCAGAAAAGUUUAAAGUCCAGUGUUUGACAGACAUCAAAAACCUGUUUUUCCCC

AACACAGAACCCUUUUAUGCUGCUUUUGGAAACC
>random_seq_from_cds__NO_7547 RANDOM_LENGTH 81 SEQ_LENGTH 72 GC_CONTENT 0.597222222222222 BASEPAIR 25 FREE_ENERGY -21.20 LEN_BP_RATIO 2.88
GGCCAUCAUCUUGAGGGACGCCAUGUCUGCCCGGUCCACGCCCACAGCAUAGAGCUCAAUACCAGAUGCUUGGGCCCGAGC
>random_seq_from_cds__NO_7548 RANDOM_LENGTH 81 SEQ_LENGTH 66 GC_CONTENT 0.606060606060606 BASEPAIR 22 FREE_ENERGY -24.80 LEN_BP_RATIO 3
CGCCACCUCAUUCACCUGGUCCUGGGGCCUCCCAUCUGUAACAAUGAUGGCCACCUUAGGGAUGUUAGAAGAGGGCUCUCG
>random_seq_from_cds__NO_7549 RANDOM_LENGTH 82 SEQ_LENGTH 70 GC_CONTENT 0.6 BASEPAIR 27 FREE_ENERGY -28.10 LEN_BP_RATIO 2.59259259259259
AGCCCCUGCCUCCACUGUGAAGGCUUCGUCCAUUGCUGUCUGGAUGGCUAGGCCUGACAUGGUGCCUGUUGACAAGGGUGUG
>random_seq_from_cds__NO_7553 RANDOM_LENGTH 99 SEQ_LENGTH 87 GC_CONTENT 0.839080459770115 BASEPAIR 35 FREE_ENERGY -57.30 LEN_BP_RATIO 2.48571428571429
GGCGGCGUCCAGGGCUGCCCCCGGGACCUCGGGUCUCCAGCCUCCGGAAGCCCGGGCGGGCCACGGGGUCGGGGGCGGCGGAGGGCAGCAGCAGCAGCG
>random_seq_from_cds__NO_7554 RANDOM_LENGTH 89 SEQ_LENGTH 77 GC_CONTENT 0.662337662337662 BASEPAIR 24 FREE_ENERGY -34.70 LEN_BP_RATIO 3.20833333333333
GCCAGAGCAGCAGGAGGAGUCCCGGGAGGCGGCGCGCGGGGGCCGGGCGCGGCAUAUUAGAGGAUGAUAGUAAGUUCUCCUGGAGCAAG
>random_seq_from_cds__NO_7560 RANDOM_LENGTH 110 SEQ_LENGTH 96 GC_CONTENT 0.385416666666667 BASEPAIR 29 FREE_ENERGY -19.43 LEN_BP_RATIO 3.31034482758621
GGCACUGACAAUAUAUUCAGAAUGGAAGUCCUUAAGAGCAACUAACAGGUUCUUGAUCAGACUGACUAUCUUUUCUUCAAGUUCAUAAUAUUUCACUGUCCAGCCAACUA
>random_seq_from_cds__NO_7568 RANDOM_LENGTH 89 SEQ_LENGTH 75 GC_CONTENT 0.453333333333333 BASEPAIR 23 FREE_ENERGY -17.39 LEN_BP_RATIO 3.26086956521739
AAUUCUUGAGUAACUCGUACCAAGCCAUCAAACACGUUAUCCUUGAGUCCCUGGAAACUGGCUUGGCCUUCCUGAGUCAACAGUUCCUG
>random_seq_from_cds__NO_7570 RANDOM_LENGTH 78 SEQ_LENGTH 60 GC_CONTENT 0.5 BASEPAIR 20 FREE_ENERGY -18.50 LEN_BP_RATIO 3
AAACCCACUCAGCAUUGUUCUGCAGAUUUCUUCUCAGCUUUGAAGACACUUCUCUCAGGGUGAGCCCUGUGUGUUCCC
>random_seq_from_cds__NO_7571 RANDOM_LENGTH 79 SEQ_LENGTH 71 GC_CONTENT 0.47887323943662 BASEPAIR 25 FREE_ENERGY -24.30 LEN_BP_RATIO 2.84

AGUGGUACUUGUUGACAUAAUCAUAAAGGACCCCUGUGGCCUUGGGCACGUUGUCUUUCAGAGAG
GUUAGCAAGCCAGA
>random_seq_from_cds__NO_7574 RANDOM_LENGTH 80 SEQ_LENGTH 63
GC_CONTENT 0.619047619047619 BASEPAIR 25 FREE_ENERGY
-23.90 LEN_BP_RATIO 2.52
CGUCAUCUUCAUCCAUAUCCAUGCCCACGGUGCCUACGGCUGGGGAGGCUGCUGAGGUGGAGAUG
CCUUUCUUGUCUUUC
>random_seq_from_cds__NO_7576 RANDOM_LENGTH 92 SEQ_LENGTH 65
GC_CONTENT 0.430769230769231 BASEPAIR 26 FREE_ENERGY
-15.60 LEN_BP_RATIO 2.5
UUGCCAUCUUCUUCAUAUUCUGCACUGAAGUCACGGUGUGCAAAUGUUCCUUUAGUCUUAGAGGC
UAACGUACCAUCUUCGAUUUUGUGUGU
>random_seq_from_cds__NO_7586 RANDOM_LENGTH 102 SEQ_LENGTH 91
GC_CONTENT 0.428571428571429 BASEPAIR 33 FREE_ENERGY
-28.00 LEN_BP_RATIO 2.75757575757576
AAGCUCUUGUCAUAGACUGGUAGGAUGAUAUUUUUGAGGAACCUUAGGUGUCCUUCUAAGGAUCC
UGCAAUGUCAAGGUGUGCCUUUUCUUGGUCAUUGGAA
>random_seq_from_cds__NO_7592 RANDOM_LENGTH 94 SEQ_LENGTH 66
GC_CONTENT 0.454545454545455 BASEPAIR 23 FREE_ENERGY
-18.40 LEN_BP_RATIO 2.8695652173913
CUGAAUAUUCCCGAGAAAGAACCGAACCCUUGACAUCUCCUUUGGUAGAUGACUCAAUGGAAAAG
UAAGAGGUGAGGCUUUCCAAGCUAAGCUU
>random_seq_from_cds__NO_7595 RANDOM_LENGTH 110 SEQ_LENGTH 62
GC_CONTENT 0.467741935483871 BASEPAIR 25 FREE_ENERGY
-21.72 LEN_BP_RATIO 2.48
UUUUUCGUGGUUAAGCUCACAGUACUGUUAUGACUACCCUCCACAAAUUUGUUGCUCAGAGACAG
AGCUGUGGCUAACUUCAAUCCCCUUUUUCUUGUCAAUCUUGUGGU
>random_seq_from_cds__NO_7612 RANDOM_LENGTH 87 SEQ_LENGTH 76
GC_CONTENT 0.5 BASEPAIR 28 FREE_ENERGY -23.00 LEN_BP_RATIO
2.71428571428571
CUGAGAAUCUGGGGCAGGCCCAUUUCCAUGACCCUUUUCCAGAAGAAGUCCAUGCUAUGUGGCCA
GCUUUCAACAGUGUCUUGAUCU
>random_seq_from_cds__NO_7613 RANDOM_LENGTH 84 SEQ_LENGTH 78
GC_CONTENT 0.448717948717949 BASEPAIR 25 FREE_ENERGY
-19.50 LEN_BP_RATIO 3.12
CGUUGCGCAGGUCAGCCUGACUAGAGAAGUCCAGUUUGGGGAUGUUCAAUUUGUGGAAGUAUUUA
GUGUUGCUAUCCAGGGUAA
>random_seq_from_cds__NO_7627 RANDOM_LENGTH 84 SEQ_LENGTH 63
GC_CONTENT 0.412698412698413 BASEPAIR 22 FREE_ENERGY
-16.40 LEN_BP_RATIO 2.86363636363636
GCCAAAGAUGCUGAACUUAAAGCCUCCUGUAACCAAUUGAUGAUUAAGGUUAUUUUGGUGUCCUG
UAGGCUUUCCAGAUACACU
>random_seq_from_cds__NO_7638 RANDOM_LENGTH 92 SEQ_LENGTH 80
GC_CONTENT 0.3 BASEPAIR 25 FREE_ENERGY -15.40 LEN_BP_RATIO

3.2
CAUUAAAGUUGAUUUUGGCAUCAUCUAAUGCAAUUUGUAUAUCAUUUUCUGUAAUUCUAUACUUU
UUUGUGAGAGCAGUCAGUUUCUCCUUG
>random_seq_from_cds__NO_7641 RANDOM_LENGTH 100 SEQ_LENGTH 67
GC_CONTENT 0.373134328358209 BASEPAIR 23 FREE_ENERGY
-19.30 LEN_BP_RATIO 2.91304347826087
UUGCAAGGUCUCAAAAAAUGGGAGGUUAAUGGAGUGAACAUCUUGGUUUUUAUCAUACUUUACAA
AAGCAACAAUUGUAAAUUCUUGGGGCUUCUCAACG
>random_seq_from_cds__NO_7644 RANDOM_LENGTH 75 SEQ_LENGTH 59
GC_CONTENT 0.542372881355932 BASEPAIR 20 FREE_ENERGY
-21.20 LEN_BP_RATIO 2.95
GUUGUUAAAUUGGGUCUUGAGUUUCCAGGUGCCUGUCUGCUCAGCUGGAGUAAGCAGGGCACUGA
CUUUGUGUUC
>random_seq_from_cds__NO_7673 RANDOM_LENGTH 82 SEQ_LENGTH 77
GC_CONTENT 0.558441558441558 BASEPAIR 29 FREE_ENERGY
-24.10 LEN_BP_RATIO 2.6551724137931
UCCAUUUGGAGAAGCAGUUUGGCAGGCGACCAGUGGGCGAGGAUCUCACUUCUGGCUUCUGCUUG
CAAACGGGGUAUGGAAA
>random_seq_from_cds__NO_7677 RANDOM_LENGTH 90 SEQ_LENGTH 78
GC_CONTENT 0.525641025641026 BASEPAIR 22 FREE_ENERGY
-22.87 LEN_BP_RATIO 3.54545454545455
UGCUUCGCACCCUUCUGCUUGAGUUACAAACUUCAGGGUAUCCACCAAGGCUCUGUCCUCUCUCU
GGAGCUCAUAGGUUGCGCUGACAGA
>random_seq_from_cds__NO_7683 RANDOM_LENGTH 75 SEQ_LENGTH 63
GC_CONTENT 0.53968253968254 BASEPAIR 20 FREE_ENERGY
-17.30 LEN_BP_RATIO 3.15
CUUGGCUCCGGGAGCAAUGACUCCAGAUGAAGAUAUUUGCAACUGUAAUCCAGCUCCAGUGGGGA
GUUCAAAGGC
>random_seq_from_cds__NO_7690 RANDOM_LENGTH 83 SEQ_LENGTH 68
GC_CONTENT 0.352941176470588 BASEPAIR 27 FREE_ENERGY
-16.70 LEN_BP_RATIO 2.51851851851852
UAUUUGGAUCAAAUAUAAGAUUCCCUUCUAUUUUGGCUGAGGCUGGGUCAAGUGAUGGAAGAGAA
ACAGAUUUGUAGAGUUGA
>random_seq_from_cds__NO_7694 RANDOM_LENGTH 81 SEQ_LENGTH 68
GC_CONTENT 0.529411764705882 BASEPAIR 25 FREE_ENERGY
-22.10 LEN_BP_RATIO 2.72
UCUGAAGAAGAACCUCCUGGUCCCUUGUCUUUAGGCUCCAUUUUCCGCAGAGCCUGGAUGGCAGC
UUUCUGGAUCAUCAGU
>random_seq_from_cds__NO_7695 RANDOM_LENGTH 85 SEQ_LENGTH 66
GC_CONTENT 0.454545454545455 BASEPAIR 25 FREE_ENERGY
-21.70 LEN_BP_RATIO 2.64
GAUGGCUUUGUACUUUGGACACAUUUCAGGAUUGAAGACUUGAGUUCUGGAGUUAACUGCUCCAU
GGUUUGGCCCAUAUUUCCAA
>random_seq_from_cds__NO_7709 RANDOM_LENGTH 70 SEQ_LENGTH 55

GC_CONTENT 0.581818181818182 BASEPAIR 23 FREE_ENERGY -31.70 LEN_BP_RATIO 2.39130434782609
CUGGCUGGUCUUCAGGAUGAAGCUGCAGAGCUGGGGAACCUCCAGCUCAACCCUUGCAGUUGAUCCUGGU
>random_seq_from_cds__NO_7711 RANDOM_LENGTH 80 SEQ_LENGTH 61 GC_CONTENT 0.655737704918033 BASEPAIR 19 FREE_ENERGY -21.80 LEN_BP_RATIO 3.21052631578947
GUGCUUGAAUCGGGUCGCAUCUUCUUGGACAGACCAGGCUGACAUUUUCCAGCAUUUCCUCUUCCGGCCCUGGCGCCCGC
>random_seq_from_cds__NO_7713 RANDOM_LENGTH 67 SEQ_LENGTH 55 GC_CONTENT 0.563636363636364 BASEPAIR 19 FREE_ENERGY -21.70 LEN_BP_RATIO 2.89473684210526
UGACAGAGGGGCCAUUGGGGAAGGUGGCUAGCUUAUCCCGCCCCUUCAAGAAGAAGGUCAGCAGCUC
>random_seq_from_cds__NO_7719 RANDOM_LENGTH 67 SEQ_LENGTH 60 GC_CONTENT 0.416666666666667 BASEPAIR 22 FREE_ENERGY -17.00 LEN_BP_RATIO 2.72727272727273
GAUUUCAUUGAGGAAACGCAGACACUCAAUACCACCAUUGUUGAUGCUCUCCUCUGUGUAGAAGUCA
>random_seq_from_cds__NO_7730 RANDOM_LENGTH 70 SEQ_LENGTH 58 GC_CONTENT 0.637931034482759 BASEPAIR 22 FREE_ENERGY -21.60 LEN_BP_RATIO 2.63636363636364
CCAGGGCCGUGCAGAGCAGGACGACGCAGGAGCAGCUGAAGGCAGCCCCACUCUGCUUCUCCUUCUCCAC
>random_seq_from_cds__NO_7733 RANDOM_LENGTH 82 SEQ_LENGTH 68 GC_CONTENT 0.676470588235294 BASEPAIR 24 FREE_ENERGY -33.10 LEN_BP_RATIO 2.83333333333333
GCGCAGCCUCCGGCGUGGGUUGGGGAAUGAGGGGUUGUCGGCCCUGGGCAUCCUGCUCCUCGGGCUUCUCCGACGUGGACCC
>random_seq_from_cds__NO_7734 RANDOM_LENGTH 71 SEQ_LENGTH 55 GC_CONTENT 0.636363636363636 BASEPAIR 20 FREE_ENERGY -26.90 LEN_BP_RATIO 2.75
ACUGCUGUGGGCACUCCCGUUGGGCUCCUUGGUCUCAAUGAGGGCAGGGGAGCUGGACUUUGAGGAAGCUG
>random_seq_from_cds__NO_7742 RANDOM_LENGTH 78 SEQ_LENGTH 66 GC_CONTENT 0.575757575757576 BASEPAIR 19 FREE_ENERGY -16.20 LEN_BP_RATIO 3.47368421052632
GAACUGCUGCUGGUCCUUCUGGCUCUCGUCUUUCUUCAUGUCUUUCAGCAUCUCGUCAGCCACGUGCUUGGGCAGGAU
>random_seq_from_cds__NO_7744 RANDOM_LENGTH 89 SEQ_LENGTH 71 GC_CONTENT 0.619718309859155 BASEPAIR 21 FREE_ENERGY -18.90 LEN_BP_RATIO 3.38095238095238
GUAGUAGGACAUGAUGCCCACAGCGAUGGCGCACAGGUAGAGGAAGACGUUGGCCAGGAUCCUCCCGCAGCAGCUGCAUCCCCUUGAGC

>random_seq_from_cds__NO_7750 RANDOM_LENGTH 72 SEQ_LENGTH 57 GC_CONTENT 0.614035087719298 BASEPAIR 20 FREE_ENERGY -20.80 LEN_BP_RATIO 2.85
CAGCACCAGCAGGGUCUCGUGGCGCUGCCUUUUGAAGUAGGUCUGGUAGAGGUUCUCCAAGGACU
CCGGCAC
>random_seq_from_cds__NO_7764 RANDOM_LENGTH 71 SEQ_LENGTH 52 GC_CONTENT 0.596153846153846 BASEPAIR 20 FREE_ENERGY -17.80 LEN_BP_RATIO 2.6
UGCACUUCCAGUCCAUGUGCUCGUGCCUGUGGUACAAGAGCCGCGAGAAGCUGCAGUACUCCAUC
CCCAUG
>random_seq_from_cds__NO_7767 RANDOM_LENGTH 75 SEQ_LENGTH 58 GC_CONTENT 0.827586206896552 BASEPAIR 21 FREE_ENERGY -30.60 LEN_BP_RATIO 2.76190476190476
CGUGUGACAUGGGCCCGCGCCGCCGGAGCCGAAAGCCCGAGGCCCCGAGGAGGCGCAGCCCGAGC
CCGACCCCGA
>random_seq_from_cds__NO_7839 RANDOM_LENGTH 84 SEQ_LENGTH 72 GC_CONTENT 0.666666666666667 BASEPAIR 25 FREE_ENERGY -27.70 LEN_BP_RATIO 2.88
UCGGAGGAAGGACUUGAGGUCUCCCCCCGCCAUGAGCUCCAGCAGGAUGAACCGGGGCAGGGAUU
GCAGGCUCACCCCAAUGCA
>random_seq_from_cds__NO_7843 RANDOM_LENGTH 72 SEQ_LENGTH 56 GC_CONTENT 0.607142857142857 BASEPAIR 22 FREE_ENERGY -23.60 LEN_BP_RATIO 2.54545454545455
CAGCUUGUACUCAGGGCUCUGCAGCUCCAUCUGCAUGGCUUGCAGCUCCUGGUGCUUCCGGCGGU
ACACCAA
>random_seq_from_cds__NO_7845 RANDOM_LENGTH 84 SEQ_LENGTH 60 GC_CONTENT 0.666666666666667 BASEPAIR 19 FREE_ENERGY -28.30 LEN_BP_RATIO 3.15789473684211
GUGGCUCCGGGGUGGGUGACACCAAUGCAGGAGACGCCAUCCUCAGCCAGCACCGUCCCGUGGUC
ACAGAAGCAGAUGACCUUG
>random_seq_from_cds__NO_7864 RANDOM_LENGTH 72 SEQ_LENGTH 56 GC_CONTENT 0.571428571428571 BASEPAIR 19 FREE_ENERGY -18.40 LEN_BP_RATIO 2.94736842105263
AGUCCUGGUGGAAGUCACAGGCCUGCCCAAGCUGGAGGACUGUCCCAUUCCAACAAGUGAAGGAG
CUCUGCA
>random_seq_from_cds__NO_7866 RANDOM_LENGTH 85 SEQ_LENGTH 66 GC_CONTENT 0.545454545454545 BASEPAIR 25 FREE_ENERGY -27.70 LEN_BP_RATIO 2.64
UCCACUGGAGAUGUAUUCCAGGGCCACUCGAAAUGGGUUGUCUGGACGCCCGAUUCUUCCCUGGA
GCACUGUCCAACCCAUGCUU
>random_seq_from_cds__NO_7869 RANDOM_LENGTH 81 SEQ_LENGTH 72 GC_CONTENT 0.597222222222222 BASEPAIR 27 FREE_ENERGY -31.00 LEN_BP_RATIO 2.66666666666667
UGCUUGGAGUCAGCUGAGGUGUUGAGAAGGAGAAAGGAGCCCUCUGGGCAUCUCCUUAGAACGCU

CUGCCCCAGGCCCAUC
>random_seq_from_cds__NO_7873 RANDOM_LENGTH 86 SEQ_LENGTH 76 GC_CONTENT 0.671052631578947 BASEPAIR 27 FREE_ENERGY -36.00 LEN_BP_RATIO 2.81481481481481
CCCGAAGAUCUGGAAGAGAAGGCGGGGCUGGGAGGCGCGAAUUGCCGCGGACAGCCUUCCCUCUC
UGCCCACUUCCGACGCCUUCU
>random_seq_from_cds__NO_7881 RANDOM_LENGTH 84 SEQ_LENGTH 70 GC_CONTENT 0.528571428571429 BASEPAIR 29 FREE_ENERGY -26.20 LEN_BP_RATIO 2.41379310344828
AGGGUUUGCAGUUUUCUGGGACACCAGUGACACACCAGGGUGGUGAACUUGUCCACGCAGGCAUU
GCGGAUCUUCUCCGGGGUG
>random_seq_from_cds__NO_7890 RANDOM_LENGTH 78 SEQ_LENGTH 69 GC_CONTENT 0.623188405797101 BASEPAIR 23 FREE_ENERGY -23.10 LEN_BP_RATIO 3
GAGCUCUACUGGCCACCCUGGACCAUUUUGGUAUGAAGGCCUUGGCCCAUCUCAGUCCCCCCGUG
GGUCAGCAGCACA
>random_seq_from_cds__NO_7893 RANDOM_LENGTH 71 SEQ_LENGTH 60 GC_CONTENT 0.533333333333333 BASEPAIR 24 FREE_ENERGY -21.60 LEN_BP_RATIO 2.5
GAUACUGAGAGCUUGCUAGGCAUUCUUCCCAGCAUCUGGGCAAGGUGAAACCCUCAAGCUUCUGG
UUGAAG
>random_seq_from_cds__NO_7901 RANDOM_LENGTH 96 SEQ_LENGTH 89 GC_CONTENT 0.49438202247191 BASEPAIR 32 FREE_ENERGY -21.80 LEN_BP_RATIO 2.78125
UUGGAACAGCAAUGGUGCAGUGAGUCUCCAGGUAGAAGUGCUCUUGGCCACCGAUGUAUAUCUCC
CCCUGACACAACAUUAUCUGCUUCGGAAAAC
>random_seq_from_cds__NO_7929 RANDOM_LENGTH 66 SEQ_LENGTH 49 GC_CONTENT 0.387755102040816 BASEPAIR 19 FREE_ENERGY -15.50 LEN_BP_RATIO 2.57894736842105
UUUUUUUCUCAUAAGGAAUGACUGUAGUCAAAUACUGUGCCGGGCAUAUGACUUCUCUCAUUCUU
C
>random_seq_from_cds__NO_7932 RANDOM_LENGTH 89 SEQ_LENGTH 59 GC_CONTENT 0.457627118644068 BASEPAIR 20 FREE_ENERGY -15.66 LEN_BP_RATIO 2.95
GCACCAGCUCCUCAGAGUACUCCUUAAUGGCAGUCUCAAUUUCUUCCAGGAUUUCAUUUAACUCA
GACACUGAGAGCCUUUUCACUCCU
>random_seq_from_cds__NO_7933 RANDOM_LENGTH 87 SEQ_LENGTH 74 GC_CONTENT 0.445945945945946 BASEPAIR 25 FREE_ENERGY -15.10 LEN_BP_RATIO 2.96
CUCUUCAUAACUGCCGGUACUAGACCUCUUGAGAGUUUGAAUUUCCUGGGAAAGCAUUGAAAGCC
GAUCUGACUGUGUAGGGGUUUC
>random_seq_from_cds__NO_7934 RANDOM_LENGTH 109 SEQ_LENGTH 93 GC_CONTENT 0.451612903225806 BASEPAIR 29 FREE_ENERGY -24.42 LEN_BP_RATIO 3.20689655172414

AUCAUCUUCUGGGUCCGGUGAUUCCUGCAUCAUUUCUUCAAUUUCUUCAAUAACCCUGGUCUGCC
GUGAAGAGGGGUUCAUCAUUAACACAGGAGACGAUGAUUGAGUG
>random_seq_from_cds__NO_7948 RANDOM_LENGTH 94 SEQ_LENGTH 85
GC_CONTENT 0.588235294117647 BASEPAIR 32 FREE_ENERGY
-27.60 LEN_BP_RATIO 2.65625
GGGGGGCGGCCCCGGGCCGAAGGCUUUCGCAGUGCCUCAAGAACUUGUCCAGGAUGAAGUUGCUG
AAGUUGCGGUUGAGCUGCUCGAAUUCGCG
>random_seq_from_cds__NO_7951 RANDOM_LENGTH 95 SEQ_LENGTH 89
GC_CONTENT 0.786516853932584 BASEPAIR 34 FREE_ENERGY
-47.50 LEN_BP_RATIO 2.61764705882353
GGCGCCGUCCGCGCUGCCGCGCACCAGCAGCGCCACCAGCUCGCGCGCCUCGCUCAGCACGUGGC
CCUCGAGGACUUGGCGGCUGCGCGGCUGGC
>random_seq_from_cds__NO_7955 RANDOM_LENGTH 80 SEQ_LENGTH 65
GC_CONTENT 0.815384615384615 BASEPAIR 22 FREE_ENERGY
-36.50 LEN_BP_RATIO 2.95454545454545
CCGCCUGGCCCACCGCCGCCGCGUUUCCGAUCAGUGGCCACGCAAACGGGCCCGGGGGCGCGGAC
CGGAGCUGCCGCCUC
>random_seq_from_cds__NO_7958 RANDOM_LENGTH 88 SEQ_LENGTH 79
GC_CONTENT 0.721518987341772 BASEPAIR 28 FREE_ENERGY
-37.60 LEN_BP_RATIO 2.82142857142857
GCAGGCGGCGGCCAGGGUCGAGGCUGGGAGACUCAGAGCCGCUGAGGCUGCCGGAGCUCAGGGAG
CCGCUUAGGUAGCUGUCGCGGUC
>random_seq_from_cds__NO_7963 RANDOM_LENGTH 80 SEQ_LENGTH 68
GC_CONTENT 0.838235294117647 BASEPAIR 24 FREE_ENERGY
-47.60 LEN_BP_RATIO 2.83333333333333
GUCCUCGGCGCCCCCGGUGCCGUACAGCAGAGCGGCCGCAGCCGCGGCCGCCGCGGAGGCGCAGC
AUGUCGGGGCGCCCG
>random_seq_from_cds__NO_7969 RANDOM_LENGTH 71 SEQ_LENGTH 57
GC_CONTENT 0.684210526315789 BASEPAIR 19 FREE_ENERGY
-23.30 LEN_BP_RATIO 3
GAAGGGCCGGCACAGCUCGGUCUUGUAGCGCGUGGAGUUGAUCUGGGAGCCGCCGCCCCCCUUCU
GCUGCU
>random_seq_from_cds__NO_7976 RANDOM_LENGTH 85 SEQ_LENGTH 79
GC_CONTENT 0.417721518987342 BASEPAIR 26 FREE_ENERGY
-22.40 LEN_BP_RATIO 3.03846153846154
UGGGCAGGUUUUCUCAAUGAAUUGAAUUCCUUGAGUGAAGGCACACAUUGGAUUAGUUGUCACAG
AAACAUUUGAGCUGCCCACA
>random_seq_from_cds__NO_7986 RANDOM_LENGTH 98 SEQ_LENGTH 89
GC_CONTENT 0.393258426966292 BASEPAIR 31 FREE_ENERGY
-17.80 LEN_BP_RATIO 2.87096774193548
UUCAAAGUUUUAUGACAAAUUGCUGAUUUCUUGUAGGCAGAUUCUAUCAUCUGGACUCUCUUGGA
GGUUUCUAUUUCCCGUUCCUUGCUUUGGGUAUC
>random_seq_from_cds__NO_7997 RANDOM_LENGTH 89 SEQ_LENGTH 76
GC_CONTENT 0.605263157894737 BASEPAIR 26 FREE_ENERGY

-31.20 LEN_BP_RATIO 2.92307692307692
GGAGCUCUGGGAGCCUCUGUUUACUUGGAGACCCAUGGACCCUCCGGGGGUCAAAGAUGAGAGGU
CACCCAUCAUGGCGCCCCCGCAGG
>random_seq_from_cds__NO_7998 RANDOM_LENGTH 78 SEQ_LENGTH 70
GC_CONTENT 0.614285714285714 BASEPAIR 25 FREE_ENERGY
-27.10 LEN_BP_RATIO 2.8
UCCUCGCGUUCGGGCUUCUGCUUGCCGCGGCGACGGCGACUUUUGCCGCAGCUCAGGAAGGAAUG
UGUCUGUGAAAAC
>random_seq_from_cds__NO_8009 RANDOM_LENGTH 71 SEQ_LENGTH 52
GC_CONTENT 0.673076923076923 BASEPAIR 21 FREE_ENERGY
-24.60 LEN_BP_RATIO 2.47619047619048
AGGAGACGCUGCAGUUGGAGAGCGCGGCCGAGGUCGGCUUCGUGCGCUUCUUUCAGGGCAUGCCG
GAGAAG
>random_seq_from_cds__NO_8034 RANDOM_LENGTH 81 SEQ_LENGTH 66
GC_CONTENT 0.53030303030303 BASEPAIR 21 FREE_ENERGY
-23.04 LEN_BP_RATIO 3.14285714285714
GUCAGCAGAAGUGUCCAUUGUGGACUGCAUCUUAGCCCGAGUAGGGGCUGGUGACAGUCAAUUGA
AAGGAGUCUCCACGUU
>random_seq_from_cds__NO_8038 RANDOM_LENGTH 110 SEQ_LENGTH 92
GC_CONTENT 0.402173913043478 BASEPAIR 30 FREE_ENERGY
-24.42 LEN_BP_RATIO 3.06666666666667
UAACUAUGCUUUAUCAGGUGAAGAAAGGGUGUCUGUGAUCAAAGUUUUGGGAUUCAUGUUGCAGA
GCUUGCUAAUUUCCCUAAGCAUGUAAUAGAGUGUGCUAAACAGAA
>random_seq_from_cds__NO_8049 RANDOM_LENGTH 94 SEQ_LENGTH 81
GC_CONTENT 0.802469135802469 BASEPAIR 32 FREE_ENERGY
-48.10 LEN_BP_RATIO 2.53125
GGUACACCGAGGGCUUCCAGCCCGCCAGGCUGUCGGCCUCCGAGAGCGCGGAGCCGCGGCGGAAG
GUGGCGGGCAGCAGGCCGCUGCGGCGCAG
>random_seq_from_cds__NO_8051 RANDOM_LENGTH 69 SEQ_LENGTH 57
GC_CONTENT 0.666666666666667 BASEPAIR 22 FREE_ENERGY
-29.50 LEN_BP_RATIO 2.59090909090909
AGCCCGUAGGCGAUGAGGAAGGCCUUCCCGCCCACCGUCGCGGGGGUGGUCAUGCCGAAACCCUA
UGGU
>random_seq_from_cds__NO_8076 RANDOM_LENGTH 86 SEQ_LENGTH 77
GC_CONTENT 0.467532467532468 BASEPAIR 24 FREE_ENERGY
-20.50 LEN_BP_RATIO 3.20833333333333
UGCCGGUUUGAGCUGAUGCGUUUCAAGACUUUGUAUAAUGGGGAUAAUCUUCCCUUUUCCUUGAA
GUCUGUAGUGGUUGUCCAGGG
>random_seq_from_cds__NO_8079 RANDOM_LENGTH 63 SEQ_LENGTH 46
GC_CONTENT 0.434782608695652 BASEPAIR 19 FREE_ENERGY
-19.20 LEN_BP_RATIO 2.42105263157895
UGUCUGGGGAGUUUACAGGAACUUGAAUCUGAACCUGUCAUUCAAGUCACUGUGGGGUCAGCA
>random_seq_from_cds__NO_8096 RANDOM_LENGTH 110 SEQ_LENGTH 98
GC_CONTENT 0.418367346938776 BASEPAIR 31 FREE_ENERGY

-21.50 LEN_BP_RATIO 3.16129032258065
GAAGGUACCUGAAGAAGAAGCUGACAGUAUUUCAAAUGAGGAUUCAGCCACAAACAGUAGUGAUA
AUGAAGACCCUCAAGUAAACAUUGUAGAAGAGGGACCCUUUAAAU
>random_seq_from_cds__NO_8115 RANDOM_LENGTH 110 SEQ_LENGTH 84
GC_CONTENT 0.357142857142857 BASEPAIR 27 FREE_ENERGY
-19.02 LEN_BP_RATIO 3.11111111111111
AAAUUGUGGACAACUUUUGGGGUAAAGUCACAAAAGAAGGAAAAUUUGUUGAAAGUGCUGUGAUA
ACUCAAAUUUAUUGCCUCUGCUUUGUGAAUGGGAACCUGGAAUGC
>random_seq_from_cds__NO_8134 RANDOM_LENGTH 82 SEQ_LENGTH 68
GC_CONTENT 0.426470588235294 BASEPAIR 24 FREE_ENERGY
-19.00 LEN_BP_RATIO 2.83333333333333
GCAUAAUCAGAAUGGCAUGUCUUAAUCGCAGCUUUUGGUCCAGGUGAAUAGCAUAGGUGAUGGUG
UGCCAUCUUUCUAGAGU
>random_seq_from_cds__NO_8143 RANDOM_LENGTH 70 SEQ_LENGTH 54
GC_CONTENT 0.592592592592593 BASEPAIR 19 FREE_ENERGY
-23.70 LEN_BP_RATIO 2.84210526315789
CCAUAGCUCGGCAGGGCCUGCAAUUUGGUGGAAGAAAUAUCCCAAGGUUUUCGGCCCUGUGGCCC
CACGG
>random_seq_from_cds__NO_8150 RANDOM_LENGTH 67 SEQ_LENGTH 51
GC_CONTENT 0.823529411764706 BASEPAIR 19 FREE_ENERGY
-29.40 LEN_BP_RATIO 2.68421052631579
CAGCGCAGGGCGCCGUCGGGCACGCAGUUGCAGGGCUCAGGGCAGAGCGCCUCGCGCAGCGCUCG
UG
>random_seq_from_cds__NO_8151 RANDOM_LENGTH 70 SEQ_LENGTH 55
GC_CONTENT 0.727272727272727 BASEPAIR 22 FREE_ENERGY
-33.20 LEN_BP_RATIO 2.5
GCAGCGGCGGCUGCAGCAGCAGCAGCAGCUUCAGCAGCUGCAGCGCCGAGAACCGCUGCUUCAUC
AUGUC
>random_seq_from_cds__NO_8197 RANDOM_LENGTH 86 SEQ_LENGTH 50
GC_CONTENT 0.56 BASEPAIR 19 FREE_ENERGY -18.38
LEN_BP_RATIO 2.63157894736842
GAGAACUGCAUCCAACAGAGGGGGAACAAAAUUUCAGCGACCCAUCUGUGGAUCAUUGGAUCGG
CUCACCCAACCAGAUAUUAAC
>random_seq_from_cds__NO_8201 RANDOM_LENGTH 77 SEQ_LENGTH 52
GC_CONTENT 0.346153846153846 BASEPAIR 19 FREE_ENERGY
-16.50 LEN_BP_RATIO 2.73684210526316
AUUAUACUAUCCCACACUUGAUUAGGGAGUAACAUGUACUUUUCUAUCAAGUGUUCUUGUACUGU
UUGAUCUGUUUG
>random_seq_from_cds__NO_8237 RANDOM_LENGTH 87 SEQ_LENGTH 70
GC_CONTENT 0.485714285714286 BASEPAIR 22 FREE_ENERGY
-17.50 LEN_BP_RATIO 3.18181818181818
UUCCCCUCCUGAAAGGAUGUCAUCGCAACAGAUAAAGAAGACGUUGCCUUCAAAGACCUGGAUGU
GGCCAUUCUUGUGGGCUCCAUG
>random_seq_from_cds__NO_8247 RANDOM_LENGTH 82 SEQ_LENGTH 64

GC_CONTENT 0.515625 BASEPAIR 23 FREE_ENERGY -22.40
LEN_BP_RATIO 2.78260869565217
AGCCAGCAACUGUAAAGGGAUCACGCUGAGAAUGCCCUGCAAGCAGUCCACUGAGUGGGGCACCU
UGAUCGUUCUUUUUGUG
>random_seq_from_cds__NO_8258 RANDOM_LENGTH 97 SEQ_LENGTH 78
GC_CONTENT 0.512820512820513 BASEPAIR 28 FREE_ENERGY
-26.61 LEN_BP_RATIO 2.78571428571429
GCCCCUUCAUGAUCUGCUGGAGUUCCAUCUGGAGUGUUUGCACAGCUCGUCCGGGGUGAUCUCCU
GCAGUUCGUUUAAUUCGAUGGAUAGAAAGACG
>random_seq_from_cds__NO_8264 RANDOM_LENGTH 90 SEQ_LENGTH 74
GC_CONTENT 0.364864864864865 BASEPAIR 26 FREE_ENERGY
-15.60 LEN_BP_RATIO 2.84615384615385
GUUUCAGAUUCGAAGUCAUAGCCUUUGCUUUCCCAAAAACUUUUUCAAGUCUUUGUAGUUGGUGA
UGAUUCCAUUGUGAAUAACGAUAAA
>random_seq_from_cds__NO_8265 RANDOM_LENGTH 78 SEQ_LENGTH 60
GC_CONTENT 0.566666666666667 BASEPAIR 20 FREE_ENERGY
-18.60 LEN_BP_RATIO 3
UUCCAUUAUUUUUAUCAGAGCGCUGGGGGUGGCUAUUGACAGGACUGGGUUCUCCAUGUGUUGCC
CAACGGGUAUGAG
>random_seq_from_cds__NO_8293 RANDOM_LENGTH 83 SEQ_LENGTH 72
GC_CONTENT 0.652777777777778 BASEPAIR 23 FREE_ENERGY
-29.60 LEN_BP_RATIO 3.1304347826087
CUCCGAGGCCUUUGCCACCUCCAACAUUCUGCGGCUGCUGGAGCAGGGCCGGCUGCUCUCUGUGC
CCAGGGCCCCUAGCCUCC
>random_seq_from_cds__NO_8298 RANDOM_LENGTH 85 SEQ_LENGTH 71
GC_CONTENT 0.577464788732394 BASEPAIR 22 FREE_ENERGY
-21.96 LEN_BP_RATIO 3.22727272727273
CCGAGAACACAUGCAGGCGGUCACCCGAAACUACAUCACCCACCCCCGUGUCAGCCUACAGGACU
GUGUGCAGCGUGAACGGGCC
>random_seq_from_cds__NO_8300 RANDOM_LENGTH 87 SEQ_LENGTH 74
GC_CONTENT 0.554054054054054 BASEPAIR 29 FREE_ENERGY
-26.10 LEN_BP_RATIO 2.55172413793103
UGCUUGAGGUGGCUGGCACCAAGGCGAUUGUUCAGGGUGUUUGAAGGGACAUCAGGGAUCGAUGC
CAGGAAGACCACUUGCGAAUUU
>random_seq_from_cds__NO_8375 RANDOM_LENGTH 89 SEQ_LENGTH 68
GC_CONTENT 0.338235294117647 BASEPAIR 26 FREE_ENERGY
-16.60 LEN_BP_RATIO 2.61538461538462
GCCCGAAGACCCUUCUACUUUAGUUACUGUAGAUGAAAUACAAGAUGACAGCAGUGAUUUGCAUU
UAGUGACUUUGGAUGAAGUAACUG
>random_seq_from_cds__NO_8376 RANDOM_LENGTH 110 SEQ_LENGTH 59
GC_CONTENT 0.355932203389831 BASEPAIR 20 FREE_ENERGY
-16.80 LEN_BP_RATIO 2.95
AAGAGGAUGAAGACUCUCUGGCGGAUUUUAACAACCUUAAAGAAGAGCUUAAUUUUGUUACUGUU
GAUGAAGUUGGAGAGGAGGAAGAUGGAGAUAAUGAUUUAAAAGUU

>random_seq_from_cds__NO_8403 RANDOM_LENGTH 99 SEQ_LENGTH 81 GC_CONTENT 0.604938271604938 BASEPAIR 26 FREE_ENERGY -28.00 LEN_BP_RATIO 3.11538461538462
AGUCCCAGAGAGUUCACAGGCUUCCCAGACCCCUACACAGAGCUCAACACAGGCAAGGGGGGAAG
GUGUGGCUUAUCGUGGCCGGCUUCUGCUCUCCCU
>random_seq_from_cds__NO_8409 RANDOM_LENGTH 73 SEQ_LENGTH 66 GC_CONTENT 0.636363636363636 BASEPAIR 23 FREE_ENERGY -15.40 LEN_BP_RATIO 2.8695652173913
ACCAGUACCUGUACCAGCUGCGCACCCAUCACCUGAGCCAAAUCACUGAGGCUGCCCUGGCCCUG
AAGCUCGG
>random_seq_from_cds__NO_8414 RANDOM_LENGTH 85 SEQ_LENGTH 69 GC_CONTENT 0.492753623188406 BASEPAIR 26 FREE_ENERGY -21.50 LEN_BP_RATIO 2.65384615384615
AGUUUGCUGAGGGGAAGCUGUCUGUCUUUGCUGAAACCGUAUGAGAACGAGACUAAGUUGGCCCU
UGUUGGGAACUGGGGCACAA
>random_seq_from_cds__NO_8425 RANDOM_LENGTH 78 SEQ_LENGTH 58 GC_CONTENT 0.603448275862069 BASEPAIR 19 FREE_ENERGY -16.30 LEN_BP_RATIO 3.05263157894737
GAACACCCUUAACCCCACCUGGGACCAGACGCUCAUCUUCUACGAGAUCGAGAUCUUUGGCGAGC
CGGCCACAGUUGC
>random_seq_from_cds__NO_8435 RANDOM_LENGTH 88 SEQ_LENGTH 69 GC_CONTENT 0.565217391304348 BASEPAIR 25 FREE_ENERGY -32.60 LEN_BP_RATIO 2.76
AGCCCCUCAUCCCCAUCCAGGGAGGAAGAGUUCAUCGAUUGGUGGAGCAAAUUCUUUGCCUCCAU
AGGGGAGAGGGAAAAGUGCGGCU
>random_seq_from_cds__NO_8436 RANDOM_LENGTH 97 SEQ_LENGTH 81 GC_CONTENT 0.493827160493827 BASEPAIR 28 FREE_ENERGY -28.90 LEN_BP_RATIO 2.89285714285714
CCUACCUGGAGAAGGAUUUUGACACCCUGAAGGGUCUAUGACACACAGCUGGAGAAUGUGGAGGC
CUUUGAGGGCCUGUCUGACUUUUGUAACACCU
>random_seq_from_cds__NO_8438 RANDOM_LENGTH 85 SEQ_LENGTH 67 GC_CONTENT 0.671641791044776 BASEPAIR 19 FREE_ENERGY -21.20 LEN_BP_RATIO 3.52631578947368
UCCCAGAAGACCCAGCCAUCCCCAUGCCCCCAAGACAGUUCCACCAGCUGGCCGCCCAGGGACCC
CAGGAGUGCUUGGUCCGUAU
>random_seq_from_cds__NO_8445 RANDOM_LENGTH 110 SEQ_LENGTH 94 GC_CONTENT 0.680851063829787 BASEPAIR 35 FREE_ENERGY -51.32 LEN_BP_RATIO 2.68571428571429
CUGGUCCCGGAGCACGUGGAGUCACGGCCCCUCUACAGCCCCCUGCAGCCAGACAUCGAGCAGGG
GGAAGCUGCAGAUGUGGGUCGACCUAUUUCCGAAGGCCCUGGGGC
>random_seq_from_cds__NO_8473 RANDOM_LENGTH 86 SEQ_LENGTH 72 GC_CONTENT 0.472222222222222 BASEPAIR 22 FREE_ENERGY -20.40 LEN_BP_RATIO 3.27272727272727
AAUGUUGAGGAUGUCUCCUGACACUGUGCCAAAGGCUCCUAAACAUUUAAAAGCAGGGAGACACU

UCUAAAGGAGGCAUAGCUAAA
>random_seq_from_cds__NO_8500 RANDOM_LENGTH 88 SEQ_LENGTH 71 GC_CONTENT 0.492957746478873 BASEPAIR 24 FREE_ENERGY -20.60 LEN_BP_RATIO 2.95833333333333
ACCUGAAGAGGCACAGAAAGUUUCACCUGUUCUUGGACCAGCUGACCAGAAGACUGGGACACCAACUCCAACCUCUGCUUCUUACUCA
>random_seq_from_cds__NO_8509 RANDOM_LENGTH 73 SEQ_LENGTH 48 GC_CONTENT 0.5 BASEPAIR 20 FREE_ENERGY -19.30 LEN_BP_RATIO 2.4
CUGUAACCUCCCCUUCCAGCUCAUUUGGAGAGAAGCCCAUUGUUAUCUACAAACAGGCCUUUCCAGAGGGUCA
>random_seq_from_cds__NO_8565 RANDOM_LENGTH 110 SEQ_LENGTH 89 GC_CONTENT 0.438202247191011 BASEPAIR 28 FREE_ENERGY -19.40 LEN_BP_RATIO 3.17857142857143
AAAUACAAGUGAACAUUUCAGAUUUCGAAGGACAUUCCAAUCCAGAGGGGACCCCAGUAUUUGCAGAUCGGAUUACCAGAGAAGAUGAAGACCCCACUUUCUGCUUUCUC
>random_seq_from_cds__NO_8582 RANDOM_LENGTH 110 SEQ_LENGTH 75 GC_CONTENT 0.466666666666667 BASEPAIR 27 FREE_ENERGY -23.20 LEN_BP_RATIO 2.77777777777778
UUCUUCAGAAUCCAAUCACACAUUCUCUCCAGGUCUCAGAAAGUACACAUGAUGAUAGCAGAGGGGAACGAAGUGUGAAGGAAUGGAGUGGUAGACAACAGCAGAGAAAU
>random_seq_from_cds__NO_8593 RANDOM_LENGTH 80 SEQ_LENGTH 57 GC_CONTENT 0.385964912280702 BASEPAIR 19 FREE_ENERGY -16.10 LEN_BP_RATIO 3
UGAGGACAGAAAGUUAAAAAAGAACAAGAAGAAUUCCCAUGAAGGGAGUUUCCUGGUUUGUUCCUGUGGAAAAUGUGGAG
>random_seq_from_cds__NO_8597 RANDOM_LENGTH 110 SEQ_LENGTH 86 GC_CONTENT 0.558139534883721 BASEPAIR 27 FREE_ENERGY -22.20 LEN_BP_RATIO 3.18518518518519
GAUAAAGCGCCUGAAGUUAAUAGUCCAGGAGAGGAAGCUGCAGAGCAUGUUACAGACCGAGCGGGAUGCACUAUUCAACAUUGACAGGGAACGGCAGGGCCACCAGAAUC
>random_seq_from_cds__NO_8609 RANDOM_LENGTH 67 SEQ_LENGTH 54 GC_CONTENT 0.574074074074074 BASEPAIR 21 FREE_ENERGY -24.80 LEN_BP_RATIO 2.57142857142857
AAUGAGAUGGCCACAGCAGCUUCCUCUUCCUCCCUGGAGAAGAGCUAUGAGCUGCCAGAUGGGCAGG
>random_seq_from_cds__NO_8651 RANDOM_LENGTH 72 SEQ_LENGTH 62 GC_CONTENT 0.887096774193548 BASEPAIR 21 FREE_ENERGY -34.90 LEN_BP_RATIO 2.95238095238095
UCCAGCCGUGCUACGCGGCCCGCCGCCCCGGCCGUCCCGUCGCCCGGGGCCUCCCCGAGCCGCCCUCUCGGC
>random_seq_from_cds__NO_8653 RANDOM_LENGTH 87 SEQ_LENGTH 79 GC_CONTENT 0.683544303797468 BASEPAIR 27 FREE_ENERGY -33.20 LEN_BP_RATIO 2.92592592592593

UGCAGAGUCAGCGCUUUGGGACCAAGGAGGUGGAGGAAGACCUGGGCCGUGCUCUACCCGGCCAG
UCCCCACGGCGUAGCGCGGCUC
>random_seq_from_cds__NO_8663 RANDOM_LENGTH 81 SEQ_LENGTH 69
GC_CONTENT 0.579710144927536 BASEPAIR 23 FREE_ENERGY
-26.60 LEN_BP_RATIO 3
CAUGCCCUUCCCAGGACUCCCUAUACUCAGACCCCUUGGACAGCACGUCUGCUCAGGCAGGAGAG
GGAGUACAACGGAAGA
>random_seq_from_cds__NO_8666 RANDOM_LENGTH 99 SEQ_LENGTH 79
GC_CONTENT 0.645569620253165 BASEPAIR 20 FREE_ENERGY
-22.02 LEN_BP_RATIO 3.95
CAACCCUGCCACUGAUGACUACGCUGUGCCACCCCCUCGGAGCACAAAGCCCCUCCUUGCUCCCA
AGCCCCAGGGCCCAGCCUUCCCUGAACCUGGUAC
>random_seq_from_cds__NO_8668 RANDOM_LENGTH 69 SEQ_LENGTH 59
GC_CONTENT 0.491525423728814 BASEPAIR 21 FREE_ENERGY
-18.50 LEN_BP_RATIO 2.80952380952381
GUAGGGACUGACAAGACUGGGGUCAAGUCAGAGGGCUCUACCUGAUCUAGUUUUUGAACUUGCAA
ACAA
>random_seq_from_cds__NO_8674 RANDOM_LENGTH 81 SEQ_LENGTH 54
GC_CONTENT 0.518518518518518 BASEPAIR 24 FREE_ENERGY
-43.20 LEN_BP_RATIO 2.25
CAGGAACAUCAGGGAGGAGAUCAGCAUGAAGAACGAGUUGGUCUGAGCCAUCAUGGCUCAGACCA
GCUCAUACUUCAUGCU
>random_seq_from_cds__NO_8679 RANDOM_LENGTH 73 SEQ_LENGTH 65
GC_CONTENT 0.6 BASEPAIR 21 FREE_ENERGY -25.80 LEN_BP_RATIO
3.0952380952381
AGCAGUGGGUCCCUGGUCUCCUACAAGUCCUGGGGCAUUGGAGCCCCAAGCAGUGUUAAUCCUGG
CUACUGUG
>random_seq_from_cds__NO_8702 RANDOM_LENGTH 110 SEQ_LENGTH 93
GC_CONTENT 0.666666666666667 BASEPAIR 34 FREE_ENERGY
-39.20 LEN_BP_RATIO 2.73529411764706
AGGCGUCGGACGGCAGCCUCCUGGGGGACCUCGGGCACACACCACUUAGCAAGAAGGAGGGUAUC
AAGUGGCAGAGGCCGAGGCUCAGCCGCCAGGCUUUGAUGAGAUGC
>random_seq_from_cds__NO_8706 RANDOM_LENGTH 97 SEQ_LENGTH 73
GC_CONTENT 0.63013698630137 BASEPAIR 28 FREE_ENERGY
-37.60 LEN_BP_RATIO 2.60714285714286
AACGCCUUUGAUGCGGACGGGAACGGGGCCAUCCACUUUGAGGGACUUUGUGGUUGGCCUCUCCA
UCCUGCUGCGGGGCACAGUCCACGAGAAGCUC
>random_seq_from_cds__NO_8718 RANDOM_LENGTH 81 SEQ_LENGTH 74
GC_CONTENT 0.756756756756757 BASEPAIR 27 FREE_ENERGY
-34.00 LEN_BP_RATIO 2.74074074074074
AGCACAUGAGCCGGGCUGUCGGGCCGGAGCUCCGCCACCGAGGCACUGCCCUCGUCCAGCACCAC
GGCCCGCGCCAGCUCC
>random_seq_from_cds__NO_8741 RANDOM_LENGTH 87 SEQ_LENGTH 67
GC_CONTENT 0.597014925373134 BASEPAIR 22 FREE_ENERGY

-25.50 LEN_BP_RATIO 3.04545454545455
CUCUGGUCCAGCUUCACCUCAAACCAGGCACCAGGGUUACUUAAGAUGGCCCAGGGCCAUAGGGU
AGAGACUGAGCAUUAUGAAGAA
>random_seq_from_cds__NO_8743 RANDOM_LENGTH 81 SEQ_LENGTH 73
GC_CONTENT 0.465753424657534 BASEPAIR 24 FREE_ENERGY
-16.00 LEN_BP_RATIO 3.04166666666667
GUGAUGAUGAAGAUUUGGAUGACUUAUUUGUGGGACCUGUUGGGAACUCUGACCUCUCACCUUAU
CCUUGCCAUCCACCUA
>random_seq_from_cds__NO_8746 RANDOM_LENGTH 82 SEQ_LENGTH 69
GC_CONTENT 0.608695652173913 BASEPAIR 24 FREE_ENERGY
-27.70 LEN_BP_RATIO 2.875
UGAAGCAGAGCAUGUGGAGUCUGCUGACAGCGCUCUCCGGAAAGGAGGCAGAUGCAGAGGGCAAA
CCACAGGGAAGCUGGAA
>random_seq_from_cds__NO_8750 RANDOM_LENGTH 95 SEQ_LENGTH 84
GC_CONTENT 0.75 BASEPAIR 33 FREE_ENERGY -49.40
LEN_BP_RATIO 2.54545454545455
ACUUCGCGGAGCUGGAACGCUGGCCGCGCAGGCCCUGAGGGCUCGCGGCCCCAGUGGCGCGGCCG
CGAUGCGCUCCAUGGCAUCUGGAGGGUGGU
>random_seq_from_cds__NO_8764 RANDOM_LENGTH 82 SEQ_LENGTH 65
GC_CONTENT 0.584615384615385 BASEPAIR 25 FREE_ENERGY
-23.70 LEN_BP_RATIO 2.6
AGGAGCACAGGAUGCCUCCUCCGAGAAGGUCUGCAGCUGAUCCUUGGAGUAGACGUACUGCCAGA
UGCGUUCCAUGUCGUUC
>random_seq_from_cds__NO_8767 RANDOM_LENGTH 96 SEQ_LENGTH 78
GC_CONTENT 0.525641025641026 BASEPAIR 23 FREE_ENERGY
-25.33 LEN_BP_RATIO 3.39130434782609
UCUCCUGCAAAGCCAGCUUUAAUCACCCCCGAACCCGUUGUCGAUGACCACAGGCUGGUUGGCGA
UGAUGUCGUAGGACUCCAUAUCAGUUGGUGG
>random_seq_from_cds__NO_8770 RANDOM_LENGTH 99 SEQ_LENGTH 75
GC_CONTENT 0.386666666666667 BASEPAIR 26 FREE_ENERGY
-19.42 LEN_BP_RATIO 2.88461538461538
CUUCACUCCUUAAAAGGCAAAACUUCCACAGUUGUGUUUAGCAGAAUAUCUCCAGGAUGUUCUUG
GUUGCCGCUAUGGAACAAAUAACCUUUCUACAUU
>random_seq_from_cds__NO_8775 RANDOM_LENGTH 72 SEQ_LENGTH 57
GC_CONTENT 0.456140350877193 BASEPAIR 21 FREE_ENERGY
-15.30 LEN_BP_RATIO 2.71428571428571
UCAGAGUAAGAUCCGGCGCUUGAAACAUUUUACCCAAUGAAGCCCAGCUGGGAAAACUCUAGAAU
CAUCCAU
>random_seq_from_cds__NO_8788 RANDOM_LENGTH 84 SEQ_LENGTH 73
GC_CONTENT 0.452054794520548 BASEPAIR 23 FREE_ENERGY
-17.70 LEN_BP_RATIO 3.17391304347826
AGAAUUGUCUUUGGAAGCUCAAGGCAUCAAAGCUGACAGAGAAACUGUUGCAGUGAAGGCCAACA
GAAAACAAUGAAGAGGAAU
>random_seq_from_cds__NO_8816 RANDOM_LENGTH 81 SEQ_LENGTH 67

GC_CONTENT 0.447761194029851 BASEPAIR 25 FREE_ENERGY -22.00 LEN_BP_RATIO 2.68
GAUGUCAUCUUGAUCAAUGGGCGUUUGAAGGAAGGAGAUACAAUCAUUGUUCCUGGAGUAGAAGG
GCCCAUUGUAACUCAG
>random_seq_from_cds__NO_8818 RANDOM_LENGTH 110 SEQ_LENGTH 86 GC_CONTENT 0.441860465116279 BASEPAIR 26 FREE_ENERGY -22.30 LEN_BP_RATIO 3.30769230769231
GCUCAGGGGGUAAAGAUUCUUGGAAAAGACCUGGAGAAAACAUUGGCUGGUUUACCCCUCCUUGU
GGCUUAUAAAGAAGAUGAAAUCCCUGUUCUUAAAGGAUGAAUUGA
>random_seq_from_cds__NO_8828 RANDOM_LENGTH 70 SEQ_LENGTH 56 GC_CONTENT 0.678571428571429 BASEPAIR 19 FREE_ENERGY -27.12 LEN_BP_RATIO 2.94736842105263
GGUGGGCGCUGUUCCGCAGCCAGUGCAGGCCCUGUUGGGAGUACUGGACCAGGUGCUCCAUGCUG
CUGUG
>random_seq_from_cds__NO_8830 RANDOM_LENGTH 90 SEQ_LENGTH 78 GC_CONTENT 0.58974358974359 BASEPAIR 26 FREE_ENERGY -27.00 LEN_BP_RATIO 3
GUAGUCGUAGCUGUGCAGGAUGCUGUUGGUGAUGCUGACGUGGUUGGCCGCCAUCUGGUGGAUGC
GCUGUGGGAUGCUGACGAUGGUCGA
>random_seq_from_cds__NO_8833 RANDOM_LENGTH 119 SEQ_LENGTH 107 GC_CONTENT 0.476635514018692 BASEPAIR 36 FREE_ENERGY -26.30 LEN_BP_RATIO 2.97222222222222
AAUAGUCGAUUAGUGCUUUUGAAUACUUUACAGCGUGGUCCCUUUUGAGUCGAAACAUCCGCCAG
UACAGGAGGGCCAGGCAUCGGUAACCAUAAUGCAGCCAGUUGUUUGUCUUCUGG
>random_seq_from_cds__NO_8837 RANDOM_LENGTH 86 SEQ_LENGTH 74 GC_CONTENT 0.662162162162162 BASEPAIR 29 FREE_ENERGY -35.50 LEN_BP_RATIO 2.55172413793103
UCUGCCUCUUGCAGUCCCUGUGGCCGUUGGAGCCUGGAGACCACGGCUUCGUCUGCGGCCGUGAC
UUGUGGAGGGGAAUGUUUUCA
>random_seq_from_cds__NO_8840 RANDOM_LENGTH 90 SEQ_LENGTH 72 GC_CONTENT 0.458333333333333 BASEPAIR 25 FREE_ENERGY -23.00 LEN_BP_RATIO 2.88
UUUAUUGGUAUUGCCACACCUGUUGAUGUUCAUGUUGCAGUGGUUUGCAGACAAAGUAUUACUGG
UGGAGGUGGCCAGUCUUGAAGAGCU
>random_seq_from_cds__NO_8842 RANDOM_LENGTH 75 SEQ_LENGTH 62 GC_CONTENT 0.612903225806452 BASEPAIR 20 FREE_ENERGY -22.70 LEN_BP_RATIO 3.1
UUGGAUUUUGGCAAAGCCUUUUCUGCAGGUGUGUCCGAGGUGUGGCUGGGCGGUGCGCUCUCAGA
GUCCUUGGUG
>random_seq_from_cds__NO_8844 RANDOM_LENGTH 78 SEQ_LENGTH 58 GC_CONTENT 0.551724137931034 BASEPAIR 23 FREE_ENERGY -19.20 LEN_BP_RATIO 2.52173913043478
CCUGAUCUCAUCACUGUCCUUUAGAGGGGAGAGAAGUUCGUUCCGGCCAAAGGGGACCAGUGUGU
AGAACUGCUCCUC

>random_seq_from_cds__NO_8848 RANDOM_LENGTH 71 SEQ_LENGTH 58 GC_CONTENT 0.689655172413793 BASEPAIR 19 FREE_ENERGY -28.20 LEN_BP_RATIO 3.05263157894737
ACGGAGGAGCGCAGCUCCUUGCGGUGGCUCGCUCUGUUGUUGCCACAGGGCCUGGUUUUGGUGGG
CUCCGG
>random_seq_from_cds__NO_8850 RANDOM_LENGTH 87 SEQ_LENGTH 75 GC_CONTENT 0.76 BASEPAIR 30 FREE_ENERGY -46.00 LEN_BP_RATIO 2.5
GUCCUCUCGGUGCGCCUGGUGGGCUUCUUGCCCGCGGACCUCCGGGCAGGCGCGGGCGCGUUCUC
CGCGGGCGCACAGGGCACUGCG
>random_seq_from_cds__NO_8872 RANDOM_LENGTH 110 SEQ_LENGTH 99 GC_CONTENT 0.464646464646465 BASEPAIR 33 FREE_ENERGY -22.90 LEN_BP_RATIO 3
UGGACAAAACCACCGUGUUGGAAAAGGUCAUCGGAUUUUUGCAGAAACACAAUGGAAGUCUCAGC
GCAAACGGAAAUCUGUGACAUUCAGCAAGACUGGAAGCCUUCAUU
>random_seq_from_cds__NO_8874 RANDOM_LENGTH 75 SEQ_LENGTH 55 GC_CONTENT 0.490909090909091 BASEPAIR 21 FREE_ENERGY -21.80 LEN_BP_RATIO 2.61904761904762
UCAUCUAUGUCUCUGACAGUAUCACGCCUCUCCUUGGGCAUUUACCGGUCGGAUGUCAUGGAUCA
GAAUUUGUUA
>random_seq_from_cds__NO_8883 RANDOM_LENGTH 72 SEQ_LENGTH 60 GC_CONTENT 0.633333333333333 BASEPAIR 21 FREE_ENERGY -27.20 LEN_BP_RATIO 2.85714285714286
UCCGGGUGGAAAGGAGGCAGGAGCUGGCUCUGGAAGACCCGCCAUCCGAGGCCCUCCACUCCUCA
GCACUAA
>random_seq_from_cds__NO_8901 RANDOM_LENGTH 88 SEQ_LENGTH 63 GC_CONTENT 0.46031746031746 BASEPAIR 23 FREE_ENERGY -16.90 LEN_BP_RATIO 2.73913043478261
CUACAGAACAAGCCUCCAGGAUUCAUCAACACAAAGAGAAACUUUGGUUUGUUCCUGCUAAGGUG
GAGGAUUCAGGACAUUACUAUUG
>random_seq_from_cds__NO_8929 RANDOM_LENGTH 92 SEQ_LENGTH 67 GC_CONTENT 0.537313432835821 BASEPAIR 25 FREE_ENERGY -23.90 LEN_BP_RATIO 2.68
GCCUUCCUUUCAUGUGCCACGCUGGAGUGUCCACAGCAUACAUUAUAUUACAGCUCCCAGGCUCC
GGAUUUUCGAGCUUACUUGAUAGGAGG
>random_seq_from_cds__NO_8934 RANDOM_LENGTH 87 SEQ_LENGTH 77 GC_CONTENT 0.584415584415584 BASEPAIR 24 FREE_ENERGY -26.10 LEN_BP_RATIO 3.20833333333333
UCCCCGAAUCGCUGGGCUUUGGCCUGUUGAAGAACCUGUCAGAAGAACAAAUCGCGGUCUACAGU
GCCCUGAUCCAGGACGGGAUGA
>random_seq_from_cds__NO_8946 RANDOM_LENGTH 70 SEQ_LENGTH 51 GC_CONTENT 0.470588235294118 BASEPAIR 20 FREE_ENERGY -15.80 LEN_BP_RATIO 2.55
AAUAGUGGAAGGAUCGCAGUAAUAUAGUUCCGGUUCUUCUUGGACCAAAGCUUAACCAUGUUGCA

GUGGA
>random_seq_from_cds__NO_8951 RANDOM_LENGTH 83 SEQ_LENGTH 56 GC_CONTENT 0.410714285714286 BASEPAIR 23 FREE_ENERGY -15.20 LEN_BP_RATIO 2.43478260869565
UGGUGGCAGUAGUGUGCCUAGUGACUGUGUGUGUCAUUUAUAGAGUUGACUUGGUUCUAUUUUAUAGACAUUUAACGAGAAGA
>random_seq_from_cds__NO_8972 RANDOM_LENGTH 84 SEQ_LENGTH 76 GC_CONTENT 0.407894736842105 BASEPAIR 24 FREE_ENERGY -17.40 LEN_BP_RATIO 3.16666666666667
CUUUGUAAAAAUGAUGUUACUGAUGGAAGAGCAAAAUACUGGCUUGAAAGAGCAGCCAAACUUUUCCCAGGAAGUCCUGCAAUU
>random_seq_from_cds__NO_8976 RANDOM_LENGTH 95 SEQ_LENGTH 69 GC_CONTENT 0.449275362318841 BASEPAIR 24 FREE_ENERGY -22.89 LEN_BP_RATIO 2.875
UUUACAGUGUUUGGAGUCUGAUAAAAGUGACUGGCGAGCAACCAAUACAGACUUACUGCUGGCCUAUGCUAAUCUUAUGCUUCUUACGCUUUCCA
>random_seq_from_cds__NO_8982 RANDOM_LENGTH 86 SEQ_LENGTH 78 GC_CONTENT 0.41025641025641 BASEPAIR 26 FREE_ENERGY -16.57 LEN_BP_RATIO 3
CUUGGUAGCGAUGAUAUUGGAAACAUUGAUGUACGAGAACCAGAGCUUGAAGAUUUGACUAGAUACGAUGUUGGGUGCUAUUCGAG
>random_seq_from_cds__NO_8997 RANDOM_LENGTH 89 SEQ_LENGTH 74 GC_CONTENT 0.527027027027027 BASEPAIR 25 FREE_ENERGY -23.90 LEN_BP_RATIO 2.96
CCUCAUCGUUGGCCCACAGAGAAUUAUGGACCAGACUCAGUGCCUGAUGGAUAUCAGGGGUCACAGACAUUUCAUGGGGCUCCACUAAC
>random_seq_from_cds__NO_9026 RANDOM_LENGTH 89 SEQ_LENGTH 70 GC_CONTENT 0.457142857142857 BASEPAIR 23 FREE_ENERGY -23.60 LEN_BP_RATIO 3.04347826086957
AAGGAAGGACAGUGGGAUUGCAGUGUGUGCUUAGUAAGAAAUGAAGCCAGUGCUACCAAAUGUAUUGCUUGUCAGUGUCCAAGUAAACA
>random_seq_from_cds__NO_9046 RANDOM_LENGTH 88 SEQ_LENGTH 70 GC_CONTENT 0.371428571428571 BASEPAIR 25 FREE_ENERGY -17.00 LEN_BP_RATIO 2.8
AGAUGGACAGUACUUUGAACCUGUUGUUCCUUUACCUGAUCUAGUUGAAGUAUCCAGUGGUGAGGAAAAUGAACAAGUUGUUUUUAGU
>random_seq_from_cds__NO_9063 RANDOM_LENGTH 110 SEQ_LENGTH 81 GC_CONTENT 0.37037037037037 BASEPAIR 30 FREE_ENERGY -19.90 LEN_BP_RATIO 2.7
UAAAUCUGAAGAACCUGAUUCUAUUACCAAAUCCAUUAGUUCACCAUCUGUUUCCUCUGAAACUAUGGACAAACCUGUAGAUUUGUCAACUAGAAAGGAAAUUGAUACAG
>random_seq_from_cds__NO_9090 RANDOM_LENGTH 110 SEQ_LENGTH 90 GC_CONTENT 0.455555555555556 BASEPAIR 30 FREE_ENERGY -22.40 LEN_BP_RATIO 3

CUUUUCCAUCUUCUUCUUUGGGUAAUUUUUGGGAUCUACACUCCUCCAGCUGUAGAGUGGGCUUA
UCAUCUUUCAACACGCAGGACAGGUACAGAUUCUUUUCCUUGAGG
>random_seq_from_cds__NO_9111 RANDOM_LENGTH 96 SEQ_LENGTH 84
GC_CONTENT 0.726190476190476 BASEPAIR 29 FREE_ENERGY
-36.80 LEN_BP_RATIO 2.89655172413793
GCGCCCGGGUGCCCAACGGGUGGACAGGGUCUCUACCUGCGGCAGUCUGGCCUCUGUCACGCUCG
ACCCGGCCUCCUCCUCCUGCGCCUCCUCUGG
>random_seq_from_cds__NO_9113 RANDOM_LENGTH 75 SEQ_LENGTH 58
GC_CONTENT 0.724137931034483 BASEPAIR 21 FREE_ENERGY
-32.30 LEN_BP_RATIO 2.76190476190476
UGAAAAAGUUGGUGGUGCGGUGCAGCUGGGCCGCUGGCUGCGGCUGGUGAGCAGGCGCCGCGAGA
UGCUGAGGCG
>random_seq_from_cds__NO_9115 RANDOM_LENGTH 85 SEQ_LENGTH 76
GC_CONTENT 0.776315789473684 BASEPAIR 25 FREE_ENERGY
-31.20 LEN_BP_RATIO 3.04
GACACCGGCACGCUGUCUCCAUCGCUGCCGCUGCCGCUGCUGCCGCUGGCGCCCGGACUGAGGCU
CAGGCUGAGGCCGCCCGGAG
>random_seq_from_cds__NO_9117 RANDOM_LENGTH 110 SEQ_LENGTH 99
GC_CONTENT 0.666666666666667 BASEPAIR 35 FREE_ENERGY
-45.70 LEN_BP_RATIO 2.82857142857143
GAUGAUGCUGGUCCUGCAGGUGCCCUGGCUCUGCUCCAAGUGGCUGGCCUUGGUGCUGUUGCUGA
AGGAGGCCACGGGGUGCAGUGGGAACUCACGGAGGUGCCAUUGCU
>random_seq_from_cds__NO_9124 RANDOM_LENGTH 67 SEQ_LENGTH 49
GC_CONTENT 0.571428571428571 BASEPAIR 19 FREE_ENERGY
-18.70 LEN_BP_RATIO 2.57894736842105
CCAGCAGCCAGGAGUAGUUGGCCAUGAUGCAGUACUGGAACAGCACCAUGACCAGCUUGCAGCCC
GC
>random_seq_from_cds__NO_9126 RANDOM_LENGTH 92 SEQ_LENGTH 74
GC_CONTENT 0.621621621621622 BASEPAIR 24 FREE_ENERGY
-22.60 LEN_BP_RATIO 3.08333333333333
GGACACGAACAGGUGCAUGUGGAUGUAGUUGCGAGUGCAGUGGAGCCUCCCGGAAAGCACAGAGG
AUGCCAAGGGCGACCAGGAGCAUGACC
>random_seq_from_cds__NO_9160 RANDOM_LENGTH 89 SEQ_LENGTH 66
GC_CONTENT 0.46969696969697 BASEPAIR 25 FREE_ENERGY
-21.00 LEN_BP_RATIO 2.64
GUACGAAACAAGAGGGCUGGCAAGGAAGAACCCGUUGUUGUCCAUUGCAGGUGCUGGAAUCGGAA
GAACUGGGGUUCUUAUUACUAUGG
>random_seq_from_cds__NO_9161 RANDOM_LENGTH 77 SEQ_LENGTH 64
GC_CONTENT 0.40625 BASEPAIR 22 FREE_ENERGY -17.10
LEN_BP_RATIO 2.90909090909091
AAACAGCCAUGUGUCUCAUUGAAUGCAAUCAGCCAGUUUAUCCACUAGAUAUUGUAAGAACAAUG
AGAGAUCAGCGA
>random_seq_from_cds__NO_9170 RANDOM_LENGTH 77 SEQ_LENGTH 60
GC_CONTENT 0.85 BASEPAIR 23 FREE_ENERGY -41.70

LEN_BP_RATIO 2.60869565217391
AUGGACGGGCUGCCCGGUCGGGCGCUGGGGGCCGCCUGCCUUCUGCUGCUGGCGGCCGGCUGGCUGGGGCCUGAGGC
>random_seq_from_cds__NO_9200 RANDOM_LENGTH 86 SEQ_LENGTH 62 GC_CONTENT 0.419354838709677 BASEPAIR 21 FREE_ENERGY -15.02 LEN_BP_RATIO 2.95238095238095
UGUCAUUCCUCCUUUCAUGAAACUUGAUCAGAAACUGGCAAGCUCUAAAUUUGUUGGGGUUCAUGGUGUACAGCAAGAUUCGUUUC
>random_seq_from_cds__NO_9211 RANDOM_LENGTH 78 SEQ_LENGTH 66 GC_CONTENT 0.5 BASEPAIR 25 FREE_ENERGY -26.70 LEN_BP_RATIO 2.64
CAUUUGCUCAUAGAAGUCAAACAGGUCCAUGGGGAUGUCAGAUUUACCCUGUGGAUCUGUCACUCGGGAAGUGGAGGG
>random_seq_from_cds__NO_9212 RANDOM_LENGTH 81 SEQ_LENGTH 62 GC_CONTENT 0.5 BASEPAIR 21 FREE_ENERGY -19.20 LEN_BP_RATIO 2.95238095238095
CCCACCACUGCUUUCAGCAGUCUUAGAAAUCGGCAGAUUUGCUUGUGAAAGUCUCUGUGAUGAGCUCAGUGGCCUCCCCUU
>random_seq_from_cds__NO_9215 RANDOM_LENGTH 81 SEQ_LENGTH 72 GC_CONTENT 0.527777777777778 BASEPAIR 24 FREE_ENERGY -24.30 LEN_BP_RATIO 3
CUGAGCUUCCUGAGGUACUCGGUGAUGUCACUGGUUUGCAGCCCAACGCUGACAGCUGCAUACAAGGAGUAGGCAGUUAGU
>random_seq_from_cds__NO_9234 RANDOM_LENGTH 85 SEQ_LENGTH 75 GC_CONTENT 0.613333333333333 BASEPAIR 28 FREE_ENERGY -26.60 LEN_BP_RATIO 2.67857142857143
CCCCUCUGCACAUGCCGCCACUCGCUCAGGUAGCGGGACACGGGGUCUCGUAGCAGGGUGAUGUAGUAGAACUUCCCUGGGCGUG
>random_seq_from_cds__NO_9235 RANDOM_LENGTH 99 SEQ_LENGTH 92 GC_CONTENT 0.760869565217391 BASEPAIR 35 FREE_ENERGY -46.70 LEN_BP_RATIO 2.62857142857143
CGCAGCGCGGCGGAGUCGCGGCGGUCCAGCACGCCGGGCACGCAGUUGGUGAGCUCGGUCCAGUCGGCGUGCAGCCCGCAGCUCCAGCCGGUGGAGAAG
>random_seq_from_cds__NO_9237 RANDOM_LENGTH 86 SEQ_LENGTH 76 GC_CONTENT 0.644736842105263 BASEPAIR 31 FREE_ENERGY -35.20 LEN_BP_RATIO 2.45161290322581
GCCGAAGGUGGUGCCGCCCGUCUUCUGGAUGUGCAGGAAGACGAUCACGUCGUCGCCCUUCAUGUCGAAGCGCAGCGAGCGCUCCA
>random_seq_from_cds__NO_9248 RANDOM_LENGTH 87 SEQ_LENGTH 56 GC_CONTENT 0.607142857142857 BASEPAIR 20 FREE_ENERGY -23.80 LEN_BP_RATIO 2.8
GAAGAAUUCCGGUGGAUGAGACUACGGAUCCGGCGAAUGGCUGACGCAUGGAUCCAAGCAAUCAAGUCCCUGGCAGAAAAGCAGAAC
>random_seq_from_cds__NO_9263 RANDOM_LENGTH 78 SEQ_LENGTH 53

GC_CONTENT 0.60377358490566 BASEPAIR 21 FREE_ENERGY -21.40 LEN_BP_RATIO 2.52380952380952
AACAAGGACAAGGACAUGCUGAAGGUACAAGGUGACCUGCCAAAGCUCAGAGCUGGCCAAGGACAUCCUGGUGCCCUC
>random_seq_from_cds__NO_9270 RANDOM_LENGTH 80 SEQ_LENGTH 65 GC_CONTENT 0.415384615384615 BASEPAIR 23 FREE_ENERGY -19.00 LEN_BP_RATIO 2.82608695652174
UUACAACAUCCUGCUCUAUUCCUUGGUGCCACUUAUGUUAAUUGCGGGGAUUGUCAUUUGUGCAUUUUGGGUGUACAGGC
>random_seq_from_cds__NO_9272 RANDOM_LENGTH 86 SEQ_LENGTH 66 GC_CONTENT 0.439393939393939 BASEPAIR 19 FREE_ENERGY -17.30 LEN_BP_RATIO 3.47368421052632
GUUAUUAGAAGUGAAAGCAAGGGGAAGAUUUGGUUGUGUCUGGAAAGCCCAGUUGCUUAACGAAUAUGUGGCUGUCAAAAUAUUUC
>random_seq_from_cds__NO_9285 RANDOM_LENGTH 88 SEQ_LENGTH 73 GC_CONTENT 0.383561643835616 BASEPAIR 21 FREE_ENERGY -15.33 LEN_BP_RATIO 3.47619047619048
CAUCCAUUUGAAUGCCUUCCAUUCCUGUCAGAAGCACUGUCCUCUUAGAUGUUCCUGAAAAUAUCCUGAUACUUUUUCAAGUGUAUUU
>random_seq_from_cds__NO_9291 RANDOM_LENGTH 77 SEQ_LENGTH 55 GC_CONTENT 0.381818181818182 BASEPAIR 20 FREE_ENERGY -19.00 LEN_BP_RATIO 2.75
CGAGCUCACUUGAAACGAACAGGAGAUAUUUGACAACUGGCUGUCAUUCUCAGGAGUUUCAACUGAUAAGAAUUUCA
>random_seq_from_cds__NO_9296 RANDOM_LENGTH 110 SEQ_LENGTH 96 GC_CONTENT 0.510416666666667 BASEPAIR 32 FREE_ENERGY -30.60 LEN_BP_RATIO 3
CGCAGAAGCUAAGGCGGUGUGUGAAUUUGAAGGCGGCCAUCUCGCAACUUACAAGCAGCUAGAGGCAGCCAGAAAAAUUGGGAUUUCAUGUCUGUGCUGCUGGAUGGAUG
>random_seq_from_cds__NO_9311 RANDOM_LENGTH 82 SEQ_LENGTH 72 GC_CONTENT 0.458333333333333 BASEPAIR 23 FREE_ENERGY -18.10 LEN_BP_RATIO 3.1304347826087
AUACCUCGGUAGUUAAUGUCACUGAAGUCCUGCAUGUUCUUCUUUACUCGUUCAGUGAUAGGAUCUGUCACCACUGGUGUGA
>random_seq_from_cds__NO_9316 RANDOM_LENGTH 86 SEQ_LENGTH 58 GC_CONTENT 0.53448275862069 BASEPAIR 24 FREE_ENERGY -21.90 LEN_BP_RATIO 2.41666666666667
UGUAUAACACCCGAGCUAAUGUUUUCUUGAUUGCGUUUGACUCUCUGCAUCUCAGGAGUGACAGGGGUUGCGGUGGCUUUCCCCAC
>random_seq_from_cds__NO_9323 RANDOM_LENGTH 72 SEQ_LENGTH 48 GC_CONTENT 0.416666666666667 BASEPAIR 19 FREE_ENERGY -15.70 LEN_BP_RATIO 2.52631578947368
ACACGCAGUAUUUCUGGCGUGUCUUGAACAACUGUAAUUUUUCCUUUACUGUUUUUAAGGUCACACUGGUAU

>random_seq_from_cds__NO_9332 RANDOM_LENGTH 89 SEQ_LENGTH 73 GC_CONTENT 0.438356164383562 BASEPAIR 30 FREE_ENERGY -27.30 LEN_BP_RATIO 2.43333333333333
UCUUUGUAUUUCACCUAUACUCUGUAAUUCUGUGGCCUCUUUUAGGUGAAGCUGCUCCAGAUUAUCGGGUAUGGUGUGGUAGUGGCCUU
>random_seq_from_cds__NO_9333 RANDOM_LENGTH 81 SEQ_LENGTH 73 GC_CONTENT 0.397260273972603 BASEPAIR 26 FREE_ENERGY -17.90 LEN_BP_RATIO 2.80769230769231
UACUCUUCUCAUACUUCUUCUUGUACUGGUACAGAACUGGCAAGUUGGCUUGUAUUCAGGACAUGAUUCAUGAUCAGAGAC
>random_seq_from_cds__NO_9337 RANDOM_LENGTH 83 SEQ_LENGTH 64 GC_CONTENT 0.546875 BASEPAIR 22 FREE_ENERGY -25.10 LEN_BP_RATIO 2.90909090909091
GGCCUGUUUGGCUGCCUGUGUGGCCUUCUUGAUGUCUGGUCGAUCAGCCACUGUGGUGUAAUGCAGGUUCUCUUUGGCAUCUU
>random_seq_from_cds__NO_9341 RANDOM_LENGTH 82 SEQ_LENGTH 67 GC_CONTENT 0.477611940298507 BASEPAIR 22 FREE_ENERGY -17.50 LEN_BP_RATIO 3.04545454545455
CAGGAUCUGAGGCGUGUCAGGUACGGCAUGGCAGGUUCCUCUUUCCUUGACAUGUUUCUCUUUGUAUUUCAGCAUCACUUUC
>random_seq_from_cds__NO_9342 RANDOM_LENGTH 97 SEQ_LENGTH 58 GC_CONTENT 0.5 BASEPAIR 24 FREE_ENERGY -23.90 LEN_BP_RATIO 2.41666666666667
UAUUAAAGUAUUCCUGAGGGCGAGCACCGUGUUUUUGUCAUCAGUGACAGAAAGCUUGCAACCCUUGAGGAACUCCCGGUCCAGCUUAUAUUCAAAC
>random_seq_from_cds__NO_9343 RANDOM_LENGTH 80 SEQ_LENGTH 64 GC_CONTENT 0.625 BASEPAIR 24 FREE_ENERGY -27.30 LEN_BP_RATIO 2.66666666666667
AUCGCUUUGCUGCAGGGAUGACUUGGCAGCCUGGAGGAAGUCCGGUCGGUCAGGAGUCCACUUCCAGUGGGCUUUGUUGG
>random_seq_from_cds__NO_9356 RANDOM_LENGTH 79 SEQ_LENGTH 71 GC_CONTENT 0.52112676056338 BASEPAIR 21 FREE_ENERGY -15.60 LEN_BP_RATIO 3.38095238095238
CCAUGUCCUUCACGUCUUUAGCAUGCUUCAAGGCUGUGGUCUGGUUUCCAGCGUGAUGAUGGGGUCUCUCUUUGGUGGC
>random_seq_from_cds__NO_9357 RANDOM_LENGTH 84 SEQ_LENGTH 72 GC_CONTENT 0.430555555555556 BASEPAIR 23 FREE_ENERGY -18.90 LEN_BP_RATIO 3.1304347826087
AAGUUCAACAUACAGAUACCUGACUCUGAAGCUUCUGCCCUCGCUUGGCUCUUAAAAGAUCAGGAGUAUCAGGAACUGAAGUAA
>random_seq_from_cds__NO_9361 RANDOM_LENGTH 84 SEQ_LENGTH 67 GC_CONTENT 0.477611940298507 BASEPAIR 24 FREE_ENERGY -18.20 LEN_BP_RATIO 2.79166666666667
ACAUAGCGGCAAUGGAUCACUUGGGGUGUGUAUGGCAGAGAGAUCAUGUGGCCCUGCAUCUUGAA

GAAGUCUGAUUUGUAGAUC
>random_seq_from_cds__NO_9362 RANDOM_LENGTH 69 SEQ_LENGTH 50 GC_CONTENT 0.58 BASEPAIR 20 FREE_ENERGY -17.60 LEN_BP_RATIO 2.5
AACCUCACUGACAGCCUCUUGUGUCUUCUUGACUUGGCGGAUCUCAGGGGUAUCGGGAGUUGUAUGGAU
>random_seq_from_cds__NO_9368 RANDOM_LENGTH 110 SEQ_LENGTH 99 GC_CONTENT 0.404040404040404 BASEPAIR 34 FREE_ENERGY -29.99 LEN_BP_RATIO 2.91176470588235
GACAAGCUUGUAGUCAUUCCUUGUUUUCAACAUGUGAGCUUUAUACUUGAUCAUCAUCACGUAGAUCAUAAGCAUGCUUGGCAUGGAGGAUUUCAGGAGUGUCCCAGACG
>random_seq_from_cds__NO_9370 RANDOM_LENGTH 81 SEQ_LENGTH 64 GC_CONTENT 0.53125 BASEPAIR 24 FREE_ENERGY -19.90 LEN_BP_RATIO 2.66666666666667
UUCCUGACGUGAUCAUUGACUUGCAAGUCGGGGUGGCAAAUCCAUUCGUGGAGGCGCAGGCGGUAAUCAAUCUCACUGACU
>random_seq_from_cds__NO_9374 RANDOM_LENGTH 87 SEQ_LENGTH 62 GC_CONTENT 0.548387096774194 BASEPAIR 23 FREE_ENERGY -22.50 LEN_BP_RATIO 2.69565217391304
GACAAGGUGCUGGAAGGCAGGAUGUAGCUGGUGGCUUUCACUCUAUCCCAGGCCUCCUUGUACAGGUUCACUGCUGUAAAGAUUCUU
>random_seq_from_cds__NO_9378 RANDOM_LENGTH 110 SEQ_LENGTH 79 GC_CONTENT 0.531645569620253 BASEPAIR 27 FREE_ENERGY -23.80 LEN_BP_RATIO 2.92592592592593
AGGUCAUCUUUAUACACAUUCAUCACUCAAGAUCUCCUGGGCGUUUCGGACGCGUAUAACAUUGGGUUCUUCCAGAAGAGACGUCCACUGGUGGAAAUAGUGUCGAUACU
>random_seq_from_cds__NO_9386 RANDOM_LENGTH 81 SEQ_LENGTH 74 GC_CONTENT 0.540540540540541 BASEPAIR 22 FREE_ENERGY -19.93 LEN_BP_RATIO 3.36363636363636
AGUGGGAUCCAGCCAAUGCCUCGGAGCCACUCCAGGUCAGCUCUGUAGACAUUCAUCACUCUGUAGGUCAUAGGCCUUUCU
>random_seq_from_cds__NO_9390 RANDOM_LENGTH 72 SEQ_LENGTH 57 GC_CONTENT 0.473684210526316 BASEPAIR 21 FREE_ENERGY -15.80 LEN_BP_RATIO 2.71428571428571
UCAGCUUUGUAUUUCAGCCUCACUCUGAACGUCAUUGCAGUGAUGGGCAUGAACAAAUGGCACAGCAUCUGG
>random_seq_from_cds__NO_9392 RANDOM_LENGTH 93 SEQ_LENGTH 74 GC_CONTENT 0.5 BASEPAIR 27 FREE_ENERGY -21.50 LEN_BP_RATIO 2.74074074074074
UGUGCAGCAGGGGGGUUUCUGUGAGGGUGUACUUUGAUUUGGUGGCAUUCCAGUCUUUCCGGUAUUUAAUCAUCAUCGAGGAUCUCGCCACUU
>random_seq_from_cds__NO_9395 RANDOM_LENGTH 85 SEQ_LENGTH 66 GC_CONTENT 0.590909090909091 BASEPAIR 25 FREE_ENERGY -27.40 LEN_BP_RATIO 2.64

AACAUUCAUCGCUCUGCAGUUCGUAGGCCUUCUUGGCCUGAAUCACAUCGUUCUGGUCGGGCAUG
CAGGUCCACUGGUGCAGGUA
>random_seq_from_cds__NO_9410 RANDOM_LENGTH 82 SEQ_LENGTH 73
GC_CONTENT 0.520547945205479 BASEPAIR 24 FREE_ENERGY
-26.10 LEN_BP_RATIO 3.04166666666667
UGAAUUUGAGCUGGUCUGCAGGCUGGCGAUACUUCCUGUCACUCAGGAUUUCUCCAGCUCUCUUC
ACUUUCUCGACCUCUAC
>random_seq_from_cds__NO_9420 RANDOM_LENGTH 85 SEQ_LENGTH 69
GC_CONTENT 0.478260869565217 BASEPAIR 28 FREE_ENERGY
-25.10 LEN_BP_RATIO 2.46428571428571
GUCUGACUUAUACAAAUUCGUCACUCUGGAGGUCAUAGGCUUUCCGUGCAUGAAUGAUGUCAUUC
UGGUCGGGCAGGCAGAUCCA
>random_seq_from_cds__NO_9422 RANDOM_LENGTH 101 SEQ_LENGTH 61
GC_CONTENT 0.508196721311475 BASEPAIR 22 FREE_ENERGY
-20.20 LEN_BP_RATIO 2.77272727272727
UGUACCUUGUCUUGUAUUUCUCAAAAUCUUUCUUGUACUCACGGUCACUCUGCACUUUGGCAAUG
UGGAGGGACCACAUUAUCUUGGGGUCAUCGUGUACU
>random_seq_from_cds__NO_9442 RANDOM_LENGTH 78 SEQ_LENGTH 46
GC_CONTENT 0.478260869565217 BASEPAIR 19 FREE_ENERGY
-21.60 LEN_BP_RATIO 2.42105263157895
AAAAUCUGGGUGGCUCGUUUAUUUUCUCAUCCUCGAGAGAACCACUAGUCAUCCAGCCAAUGCC
UUUUAGCCACUGA
>random_seq_from_cds__NO_9448 RANDOM_LENGTH 86 SEQ_LENGTH 64
GC_CONTENT 0.453125 BASEPAIR 24 FREE_ENERGY -15.40
LEN_BP_RATIO 2.66666666666667
GCUUUAGCCAGAACAAUGUCAGGUGUAUCAGGCAUGAUGUGGAUCUGAGUCUUGUCUUUGUCCCA
AGCUUCUCUAUAAAGUCUCAU
>random_seq_from_cds__NO_9454 RANDOM_LENGTH 89 SEQ_LENGTH 75
GC_CONTENT 0.413333333333333 BASEPAIR 22 FREE_ENERGY
-17.40 LEN_BP_RATIO 3.40909090909091
CUUGUAUUCUCUUUCACUCUGCAUUUUGGCUACAUUCAUGGACAACACAAGUUUUGGGUCAUCUU
GCAGACUCCGGAAUCCAACAUGGU
>random_seq_from_cds__NO_9457 RANDOM_LENGTH 86 SEQ_LENGTH 79
GC_CONTENT 0.354430379746835 BASEPAIR 28 FREE_ENERGY
-15.90 LEN_BP_RATIO 2.82142857142857
AAAUAUCUGGUGUAUCAGGCAUCACAUGAAUCUUGGUCUUAUCUUUAUUCCAAUCAAUGGUGUAU
AAAUGCCUUGUUCAUGGUAUG
>random_seq_from_cds__NO_9460 RANDOM_LENGTH 73 SEQ_LENGTH 63
GC_CONTENT 0.444444444444444 BASEPAIR 23 FREE_ENERGY
-18.70 LEN_BP_RATIO 2.73913043478261
UGGUCAGCUCAAUGUUCAUUGCAUCUGGAAGGAGGAUGUACUUGUGAAUCAGGUGCUUGUAGUUA
GUGUUGGU
>random_seq_from_cds__NO_9465 RANDOM_LENGTH 82 SEQ_LENGTH 62
GC_CONTENT 0.483870967741936 BASEPAIR 22 FREE_ENERGY

-17.10 LEN_BP_RATIO 2.81818181818182
AAUCUGGGGGAUAUCAGGCAUGAUGUGGACUUUGGUUUUGUCAGCCUCCCAUGCUUGUUUGUAGA
GAUGCUUCGUUCAUAAU
>random_seq_from_cds__NO_9470 RANDOM_LENGTH 68 SEQ_LENGTH 59
GC_CONTENT 0.559322033898305 BASEPAIR 23 FREE_ENERGY
-25.30 LEN_BP_RATIO 2.56521739130435
GUCCACCGGGGUGUGGAAGGAGGUCUUGGAUUUCUCAUAUCCCUUCUUGUAUUCCCGGUCUGACU
GCA
>random_seq_from_cds__NO_9475 RANDOM_LENGTH 81 SEQ_LENGTH 70
GC_CONTENT 0.485714285714286 BASEPAIR 24 FREE_ENERGY
-17.40 LEN_BP_RATIO 2.91666666666667
CAGUUUGUUACUCUUGUUAAGUGCCUGUUCCAUUGUGUCCAUGGCGUAAGUGAACUUCAGCUUCU
CGGGGUGCUGGCGAUA
>random_seq_from_cds__NO_9486 RANDOM_LENGTH 87 SEQ_LENGTH 68
GC_CONTENT 0.397058823529412 BASEPAIR 26 FREE_ENERGY
-16.60 LEN_BP_RATIO 2.61538461538462
ACUCGUCUUUAUACACAUUCAUCACUCUGAAUUUGAUUGACAUUCCUCGUAUGCUCAAGACUCAU
GGCGUCAGGUAGGUAUGUGUAA
>random_seq_from_cds__NO_9487 RANDOM_LENGTH 81 SEQ_LENGTH 67
GC_CONTENT 0.477611940298507 BASEPAIR 22 FREE_ENERGY
-18.30 LEN_BP_RATIO 3.04545454545455
UGAUGCAAUGGCUGUUUGUAGUUGACAUUGGUAGCGACAUCCUGGGCCAUCUUUGCAGCUGUGAU
GCUAACCAUGUCCCCA
>random_seq_from_cds__NO_9490 RANDOM_LENGTH 78 SEQ_LENGTH 66
GC_CONTENT 0.5 BASEPAIR 23 FREE_ENERGY -24.70 LEN_BP_RATIO
2.8695652173913
AUGUUUCUCGAUGCCUUGGCUGCAGUGAUGGGAAUAGCAUCGCCCAGCACAUUGUUGCCCUUGGC
UAUUAAGUCAUAC
>random_seq_from_cds__NO_9500 RANDOM_LENGTH 100 SEQ_LENGTH 77
GC_CONTENT 0.506493506493506 BASEPAIR 25 FREE_ENERGY
-25.20 LEN_BP_RATIO 3.08
GAUGAACUGGGGCAGGUCUGGUGGCAGGUUGUAUUUGUGAAUAAUUUCCUCUCCUUUGGCUUUGU
AAGCGAUCAUCACUCCUCUGGGCCUGGUUAAUUUU
>random_seq_from_cds__NO_9503 RANDOM_LENGTH 89 SEQ_LENGTH 64
GC_CONTENT 0.5625 BASEPAIR 23 FREE_ENERGY -22.40
LEN_BP_RATIO 2.78260869565217
UUUGUAUUCAACCAUCGCUCUGCAGCGCAUAUGCCUUCUUGGCAAGGUCCACAUUGAUGCUAUCA
GGGGGGUAGCUGUAACUGUGUAAG
>random_seq_from_cds__NO_9519 RANDOM_LENGTH 84 SEQ_LENGTH 57
GC_CONTENT 0.473684210526316 BASEPAIR 21 FREE_ENERGY
-17.20 LEN_BP_RATIO 2.71428571428571
ACUUGGGCUUGUAGCAGAACAGGAGAGUCUGUAACUUGGGUGAAUUUUGUCUUAUCUGGAUGGAC
UUUGUAGGUGUGCGUCUUU
>random_seq_from_cds__NO_9521 RANDOM_LENGTH 99 SEQ_LENGTH 76

GC_CONTENT 0.434210526315789 BASEPAIR 29 FREE_ENERGY -23.40 LEN_BP_RATIO 2.62068965517241
GUAUUCUGCUUUGUAGUUUUUCAUCACUGUUUUGAGCUGUGACUUUCAUGCAGUGUGAAUGGUAUGGAUCCUCGAAGCUGCCUACAUAAUGUCCCAAAA
>random_seq_from_cds__NO_9523 RANDOM_LENGTH 95 SEQ_LENGTH 76 GC_CONTENT 0.460526315789474 BASEPAIR 28 FREE_ENERGY -19.80 LEN_BP_RATIO 2.71428571428571
CUUUGCUUUUGUCUUUUCAUAGUUUUCCUUGUAUAGUUUCGUCACUUAGGGCAUCUCCUGCUGCCUUCAGCUGCCUAAGCUGUGGGUUCUCUGAA
>random_seq_from_cds__NO_9526 RANDOM_LENGTH 88 SEQ_LENGTH 78 GC_CONTENT 0.371794871794872 BASEPAIR 24 FREE_ENERGY -15.67 LEN_BP_RATIO 3.25
UGUGCUAAUGUUAUCAGCAUUCAUUCUGGCAUUUGCAACUUCAAAGCAAGGUGUCUCACUCCAUUUGCCUUUGAUUUUAUUUUCAUAG
>random_seq_from_cds__NO_9528 RANDOM_LENGTH 102 SEQ_LENGTH 97 GC_CONTENT 0.556701030927835 BASEPAIR 32 FREE_ENERGY -29.90 LEN_BP_RATIO 3.03125
AGCCAGAGGCGUGAAUUGAGCUUGCUGUUCAGCGAGACCUUUUUUGUAGGCAACCAUCACUGAGAGCUUUCUGGGCCUGGGCAACUCUCCUCAGUUCCGGGC
>random_seq_from_cds__NO_9530 RANDOM_LENGTH 85 SEQ_LENGTH 73 GC_CONTENT 0.506849315068493 BASEPAIR 24 FREE_ENERGY -21.10 LEN_BP_RATIO 3.04166666666667
CGGCCUGGACAAGUUCAGGGGCAUCAGGAGGAAGCAGGUACUUAUCCUUGGUGUCUUCCCAGUUCUGCUUGUAUAAAACCCUUAC
>random_seq_from_cds__NO_9552 RANDOM_LENGTH 81 SEQ_LENGTH 64 GC_CONTENT 0.625 BASEPAIR 23 FREE_ENERGY -31.30 LEN_BP_RATIO 2.78260869565217
UGUUCCUGUUACACCAGUCCCCUUGGCAGCACUCCACGGCUUGGCCAGGGGACGGCGGGGUCUUACAGGUCAUCUUUCCCU
>random_seq_from_cds__NO_9559 RANDOM_LENGTH 110 SEQ_LENGTH 72 GC_CONTENT 0.513888888888889 BASEPAIR 24 FREE_ENERGY -23.80 LEN_BP_RATIO 3
ACUGGUGUAAGCAAACCUAGAGUUUCUGACGCUGUCCAGCCCAACAACUAUCUCAUCAGGACAGAGCCAGAACAAGGAACCCUCUAUUCACCAGAACAGACAUCUCUCCA
>random_seq_from_cds__NO_9583 RANDOM_LENGTH 90 SEQ_LENGTH 64 GC_CONTENT 0.578125 BASEPAIR 22 FREE_ENERGY -23.13 LEN_BP_RATIO 2.90909090909091
UAAAACCAUAUCUGACUCUUCUAGCAGAAAGUUCCAACCCAGCCACCUUGGAAGGCUCUGCAGGGUCUCUCCAGAACCUCUCUGCUGGCA
>random_seq_from_cds__NO_9585 RANDOM_LENGTH 68 SEQ_LENGTH 59 GC_CONTENT 0.474576271186441 BASEPAIR 20 FREE_ENERGY -16.80 LEN_BP_RATIO 2.95
GAUAACGAUAGAGUUGUUUCUUCCGUGGCAACAGCCUUGAGGAAUAUGGCACUAGAUGUUCGCAACAA

>random_seq_from_cds__NO_9589 RANDOM_LENGTH 99 SEQ_LENGTH 84 GC_CONTENT 0.464285714285714 BASEPAIR 27 FREE_ENERGY -19.77 LEN_BP_RATIO 3.11111111111111
GCAAUAUCGGGACCUCCGGAGCAUUUAUAAAAAGGGAUGGGUGGAAUCAGAACCAUUUUAUUACA
CCUGUGUCGACAUUGGAGCGAGACCGAUUCAAAU
>random_seq_from_cds__NO_9606 RANDOM_LENGTH 87 SEQ_LENGTH 63 GC_CONTENT 0.46031746031746 BASEPAIR 22 FREE_ENERGY -16.55 LEN_BP_RATIO 2.86363636363636
CACUGGAUGCGCCAAGAAAUCUCUUUUGGAAAAUUAAAACUUACGAACAACAAAGGAGCUUCAAA
UAACAAUGGGCAGGAUGGUGGU
>random_seq_from_cds__NO_9644 RANDOM_LENGTH 88 SEQ_LENGTH 71 GC_CONTENT 0.549295774647887 BASEPAIR 25 FREE_ENERGY -21.90 LEN_BP_RATIO 2.84
CUUUGGCUUGCUUCGUGGCCCCUCCAACACCAGGUGAAGCUGUUAGUCCCAGUAUCUGAGGAAGG
GGAAUCACUGGUUUGUUUUCUUU
>random_seq_from_cds__NO_9647 RANDOM_LENGTH 94 SEQ_LENGTH 68 GC_CONTENT 0.338235294117647 BASEPAIR 23 FREE_ENERGY -16.30 LEN_BP_RATIO 2.95652173913043
CAGGACUUGACAACUUCUGGAAAUGAUAUUUUCAGUUGGGUAUCACCACUUAAUCCAAUAACACG
AUACCAUUUCUUCAAAAAUGGUUGGAACU
>random_seq_from_cds__NO_9658 RANDOM_LENGTH 65 SEQ_LENGTH 54 GC_CONTENT 0.62962962962963 BASEPAIR 20 FREE_ENERGY -22.00 LEN_BP_RATIO 2.7
CAGACUCCCUUCUCCAAGGUGCUCAGCAGCAGUUCAACUGCCUGCAUGUUCCCGGAGGUGGCGAC
>random_seq_from_cds__NO_9664 RANDOM_LENGTH 110 SEQ_LENGTH 90 GC_CONTENT 0.4 BASEPAIR 29 FREE_ENERGY -18.00 LEN_BP_RATIO 3.10344827586207
AUGGAACUUACUCUCGCCUUCAGUUUGGAAACCUGCAGAAUUAUGAUUGCCAUGUUGGAUGAGAG
AUCACACAGGAAAAAUGGGAUUUAAUGCAUUCAAAGAGCUAUGGG
>random_seq_from_cds__NO_9673 RANDOM_LENGTH 81 SEQ_LENGTH 55 GC_CONTENT 0.345454545454545 BASEPAIR 20 FREE_ENERGY -16.60 LEN_BP_RATIO 2.75
ACACUGAAGGCGGUGUUUUAUUUCAAAUCUUUUUGAAAGAUCACCAAAUGCUUUCUUGUUUAACA
AUUUUUGCUGCAUCUG
>random_seq_from_cds__NO_9709 RANDOM_LENGTH 85 SEQ_LENGTH 66 GC_CONTENT 0.454545454545455 BASEPAIR 22 FREE_ENERGY -18.66 LEN_BP_RATIO 3
AUAUUGGCUUUCUGAAUGGCUGUCUUGAAGGGCUUCUCCAGCUCAGUCUCAAGUGCUUCAAUGAA
CCGCCUCUCCUUUCCAAUUC
>random_seq_from_cds__NO_9721 RANDOM_LENGTH 84 SEQ_LENGTH 66 GC_CONTENT 0.484848484848485 BASEPAIR 20 FREE_ENERGY -19.26 LEN_BP_RATIO 3.3
GAUGAGCAACUGAAAUGAUUGUGUGCCCAUGCUGAAUCCUUACUCAGCACUUCUUGCACCAUGGC
UUCACUCUCAUUGUCCAGA

>random_seq_from_cds__NO_9723 RANDOM_LENGTH 69 SEQ_LENGTH 53 GC_CONTENT 0.584905660377358 BASEPAIR 19 FREE_ENERGY -16.50 LEN_BP_RATIO 2.78947368421053
UUCUGGCCACCACUCAUCUGGCCUCCUCCUUCUCCAACAAGGGUGUCAAAUUGCCUGUGGCAGGUCCAU
>random_seq_from_cds__NO_9727 RANDOM_LENGTH 84 SEQ_LENGTH 63 GC_CONTENT 0.444444444444444 BASEPAIR 23 FREE_ENERGY -21.70 LEN_BP_RATIO 2.73913043478261
UUUAAUGACCAUGUUGAGGUCAUUUAGAAUCCUUCACCUCUGGUCUGGAAGGAUAAUGGAAGGUCACAUUAUGGAAUUCAAUUU
>random_seq_from_cds__NO_9744 RANDOM_LENGTH 110 SEQ_LENGTH 74 GC_CONTENT 0.662162162162162 BASEPAIR 28 FREE_ENERGY -31.20 LEN_BP_RATIO 2.64285714285714
AGAUCCCGGGCGGGGGCACCGAGGGCUACCACGUUCUGCGGGGUACAAGAAAAUUCCCCAGGACACAGAGCUGGUUUGGAGCCUUUCUUUGAUUUUAUUGUUUCUAUUAA
>random_seq_from_cds__NO_9758 RANDOM_LENGTH 82 SEQ_LENGTH 70 GC_CONTENT 0.642857142857143 BASEPAIR 25 FREE_ENERGY -20.30 LEN_BP_RATIO 2.8
ACUCACUGUGGAUGUGACGCCCCCCACUGCCAAGGCCCCCACCACCGUUGAGGACAGAGUCGGCGACUCCACCCCAGUCAGC
>random_seq_from_cds__NO_9761 RANDOM_LENGTH 85 SEQ_LENGTH 71 GC_CONTENT 0.408450704225352 BASEPAIR 25 FREE_ENERGY -17.90 LEN_BP_RATIO 2.84
UGACAUUAGAACUUUCUUUCCUGAGUAUACCCAUCAACUCUUUGGGGAUGAGUGAAACUGCUUUUGGUUACAAGGGUCUAAAGAU
>random_seq_from_cds__NO_9774 RANDOM_LENGTH 81 SEQ_LENGTH 72 GC_CONTENT 0.736111111111111 BASEPAIR 26 FREE_ENERGY -30.60 LEN_BP_RATIO 2.76923076923077
CCGCCGUGAUGGUGGUGGUGGUGAUGCGGCUGCGGGGUCUGAGUGGGGUGCAGCAGCGGCGCCGUGGCCUGCAGGUGUGAG
>random_seq_from_cds__NO_9779 RANDOM_LENGTH 82 SEQ_LENGTH 73 GC_CONTENT 0.520547945205479 BASEPAIR 28 FREE_ENERGY -27.70 LEN_BP_RATIO 2.60714285714286
CUGGAAACUGGAGUAGAUGGUGCGGGGUUUCCGGACUUUCUUUGGCUUCCCGUUCACUAUCCGAAUUUCAGGCUCAAGGUCC
>random_seq_from_cds__NO_9780 RANDOM_LENGTH 68 SEQ_LENGTH 57 GC_CONTENT 0.596491228070175 BASEPAIR 21 FREE_ENERGY -22.20 LEN_BP_RATIO 2.71428571428571
UCCUUCUCCAGGCUCGUUGUUGGCUGGGGACGAACUGGUUCCAUAGGGAGCGUAGGAGGUGUAGGCGG
>random_seq_from_cds__NO_9781 RANDOM_LENGTH 81 SEQ_LENGTH 65 GC_CONTENT 0.6 BASEPAIR 25 FREE_ENERGY -26.20 LEN_BP_RATIO 2.6
CGGUGUAGCCCAGGUCAUAGCUGCUCUUGGCGGAGUAAGGGACGUUGUUGAGGCCGCUGGCUUGG

UACUGGUAGGAACCCA
>random_seq_from_cds__NO_9783 RANDOM_LENGTH 68 SEQ_LENGTH 59
GC_CONTENT 0.711864406779661 BASEPAIR 19 FREE_ENERGY
-27.10 LEN_BP_RATIO 3.10526315789474
ACCGGAAGGGUGGGCGACUCCUGGGGCUUGUGGAGGCUGCUGCUGCUGCUGUUGCCACCCGG
GCC
>random_seq_from_cds__NO_9793 RANDOM_LENGTH 97 SEQ_LENGTH 86
GC_CONTENT 0.441860465116279 BASEPAIR 31 FREE_ENERGY
-29.80 LEN_BP_RATIO 2.7741935483871
GAAGAUGGGCCUUAUGAAGUUGGUGGAGAGACUGAGCAUGAUGAAAGUCUCGUUCCUGUUCCUGC
UAACAGUUACUUAGGGUUUUUCUUUGGACUCA
>random_seq_from_cds__NO_9811 RANDOM_LENGTH 84 SEQ_LENGTH 75
GC_CONTENT 0.386666666666667 BASEPAIR 29 FREE_ENERGY
-19.10 LEN_BP_RATIO 2.58620689655172
UUGCUAAACCUUCCCAGGUGUAUUUUGGAGGUACAGUUGUUGGCGAGCAAGCUAUGAAAUCUGAA
GAUGAAGUGGGAAGUUUAA
>random_seq_from_cds__NO_9812 RANDOM_LENGTH 85 SEQ_LENGTH 65
GC_CONTENT 0.461538461538462 BASEPAIR 20 FREE_ENERGY
-15.01 LEN_BP_RATIO 3.25
UAGAGUAUGAAUUCAGGGGUAAUAAACUUAGGUAAACCUCUUACAAACCUCGGCACAGCAACCUU
GAACAUUCAGUGGCCAAAAG
>random_seq_from_cds__NO_9817 RANDOM_LENGTH 89 SEQ_LENGTH 81
GC_CONTENT 0.506172839506173 BASEPAIR 29 FREE_ENERGY
-28.12 LEN_BP_RATIO 2.79310344827586
CUGCCGAAAAUAUCAGGCUGCCAAAUGCAGGCACUCAGGGUUCGAGUGACUGUGUUUCCCUCAAA
GACUGUAGCUCAGUAUUCGGGAGU
>random_seq_from_cds__NO_9824 RANDOM_LENGTH 84 SEQ_LENGTH 72
GC_CONTENT 0.555555555555556 BASEPAIR 26 FREE_ENERGY
-28.10 LEN_BP_RATIO 2.76923076923077
UGGGAUAGUGUCGAUAAAAACCCUUCCGCACCCAGUUGGGCAUGACAUGGGUGCUGGGUGAGCGG
UGGUGUGUGUUGAUGACGA
>random_seq_from_cds__NO_9825 RANDOM_LENGTH 84 SEQ_LENGTH 70
GC_CONTENT 0.471428571428571 BASEPAIR 22 FREE_ENERGY
-18.00 LEN_BP_RATIO 3.18181818181818
UGACAGUGAUGAUGAUGGAGGCAAUGACGAACACCAUGGUGAACAGCAUGUAUUUUCCAAUCAAG
GGCACAGCACUGGACGUGG
>random_seq_from_cds__NO_9831 RANDOM_LENGTH 68 SEQ_LENGTH 57
GC_CONTENT 0.508771929824561 BASEPAIR 19 FREE_ENERGY
-17.70 LEN_BP_RATIO 3
CUCACAGUAGCUUUUAAAGAUGGCUGGAGGUGUCCACGUGAUGUGGCCAGUGUACUGCAGGAGCA
CUU
>random_seq_from_cds__NO_9838 RANDOM_LENGTH 85 SEQ_LENGTH 68
GC_CONTENT 0.867647058823529 BASEPAIR 26 FREE_ENERGY
-47.00 LEN_BP_RATIO 2.61538461538462

CGGGGCGGGCGGCGGCGGCGGCAGCGGCGGCUGCGGCGGCGGCGGCGGCAGCCUCCGGCUUUGCG
UACCCCGGGACCUCUGAGCG
>random_seq_from_cds__NO_9841 RANDOM_LENGTH 69 SEQ_LENGTH 58
GC_CONTENT 0.672413793103448 BASEPAIR 20 FREE_ENERGY
-20.50 LEN_BP_RATIO 2.9
CCGUAUGUCGCACGGCGUGGGCUUACAGCAGAAUGCGCUCAAGUCAUCGCCGCACGCCUCGCUGG
GAGG
>random_seq_from_cds__NO_9853 RANDOM_LENGTH 81 SEQ_LENGTH 66
GC_CONTENT 0.757575757575758 BASEPAIR 23 FREE_ENERGY
-32.50 LEN_BP_RATIO 2.8695652173913
ACGCCGGGCUCCACGACCCUGCUCCAGGGGGCUCCCUGCGCCCCUGGCUUCAAGGACGACACCAA
GGGCCCGCUCAACUUG
>random_seq_from_cds__NO_9858 RANDOM_LENGTH 84 SEQ_LENGTH 74
GC_CONTENT 0.662162162162162 BASEPAIR 25 FREE_ENERGY
-32.60 LEN_BP_RATIO 2.96
AACGACUUUGACGAGUGCGGCCAGAGCGCAGCCAGCAUGUACCUGCCGGGCUGCGCCUACUAUGU
GGCCCCGUCUGACUUCGCU
>random_seq_from_cds__NO_9860 RANDOM_LENGTH 87 SEQ_LENGTH 79
GC_CONTENT 0.873417721518987 BASEPAIR 28 FREE_ENERGY
-48.70 LEN_BP_RATIO 2.82142857142857
CGGCCUGGAGCGCGCCAAGUGGCCGUACCGCGGCGGCGGCGGCGGCGGCAGCGCGGGGGGCGGCA
GCAGCGGGGGCGGCCCCGGCGG
>random_seq_from_cds__NO_9885 RANDOM_LENGTH 81 SEQ_LENGTH 76
GC_CONTENT 0.815789473684211 BASEPAIR 28 FREE_ENERGY
-45.00 LEN_BP_RATIO 2.71428571428571
GCCUCUGCCUCCGAGCCCGGCCGCUACGUGCGCUCCUGGAUGGAGCCGCUGCCCGGCUUCCCGGG
CGGUGCGGGCGGUGGC
>random_seq_from_cds__NO_9903 RANDOM_LENGTH 84 SEQ_LENGTH 73
GC_CONTENT 0.520547945205479 BASEPAIR 21 FREE_ENERGY
-19.80 LEN_BP_RATIO 3.47619047619048
CCAAGAGCUGGAGGAAGACAGAGCCGAAGGCCUGACAAAUUAAAAUGGUCAUGAGUUCGUAUAUG
GUGAACUCCAAGUAUGUGG
>random_seq_from_cds__NO_9908 RANDOM_LENGTH 75 SEQ_LENGTH 69
GC_CONTENT 0.666666666666667 BASEPAIR 21 FREE_ENERGY
-21.40 LEN_BP_RATIO 3.28571428571429
GCCCUCCGGGACGGCACUCAAGCAGCCGGCCGUGGUCUACCCCUGGAUGAAGAAGGUGCACGUGA
AUUCGGGUGA
>random_seq_from_cds__NO_9910 RANDOM_LENGTH 86 SEQ_LENGTH 54
GC_CONTENT 0.611111111111111 BASEPAIR 20 FREE_ENERGY
-21.90 LEN_BP_RATIO 2.7
AAAAAGAAUUUCAUUUUAACAGGUAUCUGACAAGGCGCCGUCGGAUUGAAAUCGCUCACACCCUG
UGUCUGUCGGAGCGCCAGAUC
>random_seq_from_cds__NO_9931 RANDOM_LENGTH 68 SEQ_LENGTH 64
GC_CONTENT 0.75 BASEPAIR 25 FREE_ENERGY -33.50

LEN_BP_RATIO 2.56
GGGCCUACGAACCUGGUGCCGCACCUGCCGCGGCAGCUGGGGGCGCGGACUACGGCUUCCUGGGG
UCC
>random_seq_from_cds__NO_9947 RANDOM_LENGTH 89 SEQ_LENGTH 77
GC_CONTENT 0.363636363636364 BASEPAIR 26 FREE_ENERGY
-16.50 LEN_BP_RATIO 2.96153846153846
UGUAGUUAAGAUUGUGAAUCUAGCUUGCAAAUAUAAUCUUUGUAUCAUACCAAUUGGUGGGAGGA
ACAAGUGUUUCAUAUGGCCUGAUG
>random_seq_from_cds__NO_9973 RANDOM_LENGTH 75 SEQ_LENGTH 63
GC_CONTENT 0.507936507936508 BASEPAIR 22 FREE_ENERGY
-20.70 LEN_BP_RATIO 2.86363636363636
AAGGCUUGGAUCGUACUUUGGAGCUUCUGUCUGUGCUGUGGACCUCAAUGCAGAUGGCUUCUCAG
AUCUGCUCGU
>random_seq_from_cds__NO_9980 RANDOM_LENGTH 110 SEQ_LENGTH 91
GC_CONTENT 0.395604395604396 BASEPAIR 32 FREE_ENERGY
-19.20 LEN_BP_RATIO 2.84375
GUUGAAAAUGGAUGGCCUUCUGUGUGCAUAGAUCUAACACUUUGUUUCUCAUAUAAGGGCAAGGA
AGUUCCAGGUUACAUUGGUUUUGUUUUAUAACAUGAGUUUGGAUG
>random_seq_from_cds__NO_9992 RANDOM_LENGTH 88 SEQ_LENGTH 59
GC_CONTENT 0.372881355932203 BASEPAIR 20 FREE_ENERGY
-17.40 LEN_BP_RATIO 2.95
UUUUAGCCCCCAAACUGAUAAGCUGUUCAACAUUUUGGAUGUCCAGGACUACUACUGGAGAAUGC
CACUUUGAAAAUUAUCAAAGAGU
>random_seq_from_cds__NO_10003 RANDOM_LENGTH 89 SEQ_LENGTH 68
GC_CONTENT 0.617647058823529 BASEPAIR 23 FREE_ENERGY
-37.80 LEN_BP_RATIO 2.95652173913043
CCCUCCUGGUAUUCCUGGGAGAAAUGGUGACCCUGGUAUUCCAGGACAACCAGGGUCCCCUGGUU
CUCCUGGCCCCCCUGGAAUCUGUG
>random_seq_from_cds__NO_10004 RANDOM_LENGTH 94 SEQ_LENGTH 80
GC_CONTENT 0.5375 BASEPAIR 29 FREE_ENERGY -26.00
LEN_BP_RATIO 2.75862068965517
AAUCAUGCCCUACUGGUCCUCAGGAACUAUUCUCCCCAGUAUGAUUCAUAUGAUGUCAAGUCUGG
AGUAGCAGUAGGAGGACUCGCAGGCUAUC
>random_seq_from_cds__NO_10019 RANDOM_LENGTH 110 SEQ_LENGTH
104 GC_CONTENT 0.625 BASEPAIR 36 FREE_ENERGY -41.90
LEN_BP_RATIO 2.88888888888889
CCAUCUGGUCCCCGAGGUCAGCCUGGUGUCAUGGGCUUCCCCGGUCCUAAAGGAAAUGAUGGGUG
CUCCUGGUAAGAAUGGAGAACGAGGUGGCCCUGGAGGACCUGGCC
>random_seq_from_cds__NO_10029 RANDOM_LENGTH 80 SEQ_LENGTH 70
GC_CONTENT 0.657142857142857 BASEPAIR 25 FREE_ENERGY
-30.60 LEN_BP_RATIO 2.8
UCCCUGGUGCUCGUGGUCUUCCUGGUCCUCCUGGUAGUAAUGGGUAACCCAGGACCCCCAGGUCC
CAGCGGUUCUCCAGG
>random_seq_from_cds__NO_10038 RANDOM_LENGTH 70 SEQ_LENGTH 56

GC_CONTENT 0.607142857142857 BASEPAIR 20 FREE_ENERGY -26.60 LEN_BP_RATIO 2.8
GGCCCCAGAGGGACCUGUUGGACCCAGUGGACCUCCUGGCAAAGAUGGAACCAGUGGACAUCCAG
GUCCC
>random_seq_from_cds__NO_10055 RANDOM_LENGTH 81 SEQ_LENGTH 66 GC_CONTENT 0.409090909090909 BASEPAIR 21 FREE_ENERGY -16.90 LEN_BP_RATIO 3.14285714285714
UUUACGUGGUACACUGGAUGGGUUUGCUGAAAUACAUGUUUCUCCUGUUUCCAUGUUGCAGUAAA
CUUUGAUUGCAUCUUC
>random_seq_from_cds__NO_10059 RANDOM_LENGTH 85 SEQ_LENGTH 65 GC_CONTENT 0.753846153846154 BASEPAIR 23 FREE_ENERGY -38.30 LEN_BP_RATIO 2.82608695652174
UGGCCAGGGGGACCCGGAGGGCCAGGUGGGCCAGGCUCACCAGGAGGGCCCCUCAGGUCCUGCUU
CUCCUACACUGCCUCGUACA
>random_seq_from_cds__NO_10060 RANDOM_LENGTH 81 SEQ_LENGTH 68 GC_CONTENT 0.647058823529412 BASEPAIR 25 FREE_ENERGY -37.22 LEN_BP_RATIO 2.72
CCUGGAGGUCCAAUUGGCCCAAGUGGCCCAGGGUUUCCUUCUUUACCUGAAGGACCAACUGGGCC
UGGAGGACCCUCUUGG
>random_seq_from_cds__NO_10063 RANDOM_LENGTH 71 SEQ_LENGTH 57 GC_CONTENT 0.649122807017544 BASEPAIR 21 FREE_ENERGY -27.71 LEN_BP_RATIO 2.71428571428571
GAACCCCGGAUCUCCUCUUUGUCCUGCAUCUCCUGGAGCACCCACAGGGCCAGGAGUUCCAGGGG
CACCCU
>random_seq_from_cds__NO_10065 RANDOM_LENGTH 84 SEQ_LENGTH 66 GC_CONTENT 0.606060606060606 BASEPAIR 24 FREE_ENERGY -33.80 LEN_BP_RATIO 2.75
GUUCCCCUACAGGACCAUUGGAGCCUGGGGGCCCCACAGGUCCAGGUGGACCUUUAUCUCCUGUU
GCACCAGUUGGUCCUACUU
>random_seq_from_cds__NO_10066 RANDOM_LENGTH 99 SEQ_LENGTH 77 GC_CONTENT 0.649350649350649 BASEPAIR 26 FREE_ENERGY -34.23 LEN_BP_RATIO 2.96153846153846
UUCCUGGUGUUCCCCGCUGGGCCUGGUAGGCCGGGCAUGCCUCUCUCUCCACGUUGCCCAGGCAU
GCCAACAAUUCCUCUCUGCCCGGUCGUUCCAGCU
>random_seq_from_cds__NO_10073 RANDOM_LENGTH 81 SEQ_LENGTH 63 GC_CONTENT 0.603174603174603 BASEPAIR 21 FREE_ENERGY -21.60 LEN_BP_RATIO 3
UUUUCUCCAGUAGGACCUGCCGGACCUGGAGGGCCCAAAGGACCUGGAAGACCCUCUUGCACCAU
CAUUUCCAGCUGUGCC
>random_seq_from_cds__NO_10085 RANDOM_LENGTH 79 SEQ_LENGTH 69 GC_CONTENT 0.666666666666667 BASEPAIR 25 FREE_ENERGY -32.30 LEN_BP_RATIO 2.76
ACCCUGAGGUCCUGGAGAUCCAGGAGGCCCUGCUGAGCCAGGAGGACCAGAGGUACCUGGAGAGC
CCCGUUGGGCCUUU

>random_seq_from_cds__NO_10133 RANDOM_LENGTH 95 SEQ_LENGTH 78 GC_CONTENT 0.41025641025641 BASEPAIR 28 FREE_ENERGY -17.20 LEN_BP_RATIO 2.78571428571429
GGGUUUGCUUGGUGUACCAGAUGAGUAUGAGGAUAUUUUUGUAAAAAUACAAAUUCACACUCUCC
AGAGCAGUAAUUGGCCUUAUAUCUUUUAGG
>random_seq_from_cds__NO_10137 RANDOM_LENGTH 86 SEQ_LENGTH 72 GC_CONTENT 0.458333333333333 BASEPAIR 24 FREE_ENERGY -18.90 LEN_BP_RATIO 3
GGAUUCAGGUUGUUUGAGCCAAUUUUGCAACACUGUCUUCACAUCAAUGCUCUGCCAAAUACCAG
UGCCUGGGUUCAUGUCAAGUU
>random_seq_from_cds__NO_10140 RANDOM_LENGTH 95 SEQ_LENGTH 57 GC_CONTENT 0.43859649122807 BASEPAIR 21 FREE_ENERGY -16.80 LEN_BP_RATIO 2.71428571428571
UCCAUCCACUUGCAUUAGAAAAUCAGCACUCUGUAGGCAUGGUAAUGAUUGUUUCCGUUGUAGCG
UGAUAAUCGUCAUCUUCCAAAGAGCCAUCG
>random_seq_from_cds__NO_10144 RANDOM_LENGTH 80 SEQ_LENGTH 54 GC_CONTENT 0.666666666666667 BASEPAIR 19 FREE_ENERGY -21.90 LEN_BP_RATIO 2.84210526315789
GUCAGAUAUCCUCCGGGAGCUGCUCUGUGUCUCUGAGAAGGCUGCUAACAUUGCCCGGGCGUGCA
GACAGCAGGAAGCCC
>random_seq_from_cds__NO_10164 RANDOM_LENGTH 93 SEQ_LENGTH 65 GC_CONTENT 0.430769230769231 BASEPAIR 21 FREE_ENERGY -20.33 LEN_BP_RATIO 3.0952380952381
UUCAGAAUGGUCCACCCAGGUGAAAGUUAUUCCUCCGAGAUGGCUUUCACUGAAUCUUAAUAAAA
AGGUGCCAGGCAUUUUAUCCUUUAGCAA
>random_seq_from_cds__NO_10182 RANDOM_LENGTH 87 SEQ_LENGTH 68 GC_CONTENT 0.382352941176471 BASEPAIR 19 FREE_ENERGY -15.57 LEN_BP_RATIO 3.57894736842105
GAGUAGGUUUUUCUCUUUGGAAACACGACCUAACUGUUCAUCCAGUUGUAUUAACAAGUUUUGAA
GAAGAAUCGUUGCCAUGGUUUC
>random_seq_from_cds__NO_10201 RANDOM_LENGTH 102 SEQ_LENGTH 89 GC_CONTENT 0.550561797752809 BASEPAIR 31 FREE_ENERGY -35.40 LEN_BP_RATIO 2.87096774193548
UGGAGGCGUUGGUCCAGCCACCAUCUGUGCCAAGAAUUCCUGCCAGAAACUCAUUGGAAGGGCAC
UGGAACGAACAGAUGCUGGAUAUAGCCUGCAGGCUUA
>random_seq_from_cds__NO_10202 RANDOM_LENGTH 80 SEQ_LENGTH 66 GC_CONTENT 0.53030303030303 BASEPAIR 27 FREE_ENERGY -28.00 LEN_BP_RATIO 2.44444444444444
UUCUGAAUGAAGUCUCCCUUUUGGGCUCGGCGCCAGGUGGGAAAGUGGAGUUCAAGAGGACUCUC
AUCAUCAGCUUCCUC
>random_seq_from_cds__NO_10214 RANDOM_LENGTH 102 SEQ_LENGTH 79 GC_CONTENT 0.518987341772152 BASEPAIR 27 FREE_ENERGY -22.70 LEN_BP_RATIO 2.92592592592593
AGGCUGGAUUCAUGAACGAUGGCAGAAUCUUGGCCCUGGACAUGGAGCAUUACAGCAAUGCAGGC

GCCUCCUUGGAUGAAUCAUUAUUCGGUGAUAGAAAUG

>random_seq_from_cds__NO_10223 RANDOM_LENGTH 110 SEQ_LENGTH 103 GC_CONTENT 0.475728155339806 BASEPAIR 34 FREE_ENERGY -33.30 LEN_BP_RATIO 3.02941176470588

UUCAGCUGUUGGAUACUUCAGGAGGUUAUGAGUCAGACAUGAACUGGGAGAAAGGCGAAGGCCAG
CCCUUCGAAUACUUUGUUUAUGGAGCUGCCUGUUCCGAGGUUGAA

>random_seq_from_cds__NO_10243 RANDOM_LENGTH 80 SEQ_LENGTH 67 GC_CONTENT 0.492537313432836 BASEPAIR 24 FREE_ENERGY -23.00 LEN_BP_RATIO 2.79166666666667

UCAUCUCGGCGGGUGACAUUUCACUAUAAGAGAAGCAGUCUGAGUUACUUUGGAGGUGUCUGUUC
UCGCACAAGAGGAGU

>random_seq_from_cds__NO_10251 RANDOM_LENGTH 110 SEQ_LENGTH 94 GC_CONTENT 0.48936170212766 BASEPAIR 35 FREE_ENERGY -24.40 LEN_BP_RATIO 2.68571428571429

AACUGCGGGAAGGAUGGAGACCGGUGGAUUCAGUGCAGCAAACAGUGAUGUGUUCUGUGGAUUCU
UACUCUGUACCAAUCUUACUCGAGCUCCACGUAUUGGUCAACUUC

>random_seq_from_cds__NO_10254 RANDOM_LENGTH 84 SEQ_LENGTH 77 GC_CONTENT 0.571428571428571 BASEPAIR 23 FREE_ENERGY -22.26 LEN_BP_RATIO 3.34782608695652

GGCCAUGGGGGUGUGUAGUAAUGAAGCCACCUGCAUUUGUGAUUUCACCUGGGCAGGGACAGAUU
GCAGUAUCCGGGAUCCAGU

>random_seq_from_cds__NO_10266 RANDOM_LENGTH 97 SEQ_LENGTH 81 GC_CONTENT 0.62962962962963 BASEPAIR 30 FREE_ENERGY -35.20 LEN_BP_RATIO 2.7

ACUGUCUUGGGGGAUGAGACAACAGGAGCGGAUGGAGUCGCUGAGGCCCAUCCAUUGCUGGUAGU
CGGGGUACUCCCCUCGCCGCAGCAAGUAUUGU

>random_seq_from_cds__NO_10270 RANDOM_LENGTH 64 SEQ_LENGTH 52 GC_CONTENT 0.596153846153846 BASEPAIR 21 FREE_ENERGY -21.20 LEN_BP_RATIO 2.47619047619048

GGAUCCAGCUGCCCUCCAGCACAUUGAGGGAGUGAAUUUCAGUGAGGUGGAAGCGGUCCUGAAC

>random_seq_from_cds__NO_10296 RANDOM_LENGTH 64 SEQ_LENGTH 57 GC_CONTENT 0.491228070175439 BASEPAIR 22 FREE_ENERGY -21.70 LEN_BP_RATIO 2.59090909090909

CCAUUCAGCAAACCUUACAGGCCCUAUACAGGUACUUCAUGUCCUGUGUGAGGUUGUAGAGUGU

>random_seq_from_cds__NO_10311 RANDOM_LENGTH 99 SEQ_LENGTH 84 GC_CONTENT 0.5 BASEPAIR 31 FREE_ENERGY -33.40 LEN_BP_RATIO 2.70967741935484

GAAGGUUCCCCACAAAUAGAAGUAGCAUCCAUCAAACAGCUUUGGCAACCAGCUGUUCUCUGUUG
AGCCUGCUUCUGCGUGGACCUUCAGGAAUUUCAU

>random_seq_from_cds__NO_10317 RANDOM_LENGTH 78 SEQ_LENGTH 61 GC_CONTENT 0.524590163934426 BASEPAIR 24 FREE_ENERGY -20.20 LEN_BP_RATIO 2.54166666666667

UCUUGGCUGCAUCGUGAAGUGGUGAGUCAUUUUGAUACCCGGUGGUGUUCACCAAUGCCUUAUGC
UGGAGCAAUAAUU

>random_seq_from_cds__NO_10334 RANDOM_LENGTH 85 SEQ_LENGTH 60 GC_CONTENT 0.666666666666667 BASEPAIR 24 FREE_ENERGY -36.70 LEN_BP_RATIO 2.5
GAUCCUCGGCUGCCGGUUCCUCGGCUGCCGAUUAUCCGGCAUCAUGGUGCGGUCGGGGAAUAAGG
GCAGCUGUUGUGCUGUGUAU
>random_seq_from_cds__NO_10342 RANDOM_LENGTH 96 SEQ_LENGTH 72 GC_CONTENT 0.5 BASEPAIR 24 FREE_ENERGY -20.60 LEN_BP_RATIO 3
GCAUUCCAUUCACUGGCCCUGCCGACUGACCAUUGGCUCCAAUUUGUCUAUAAGGAUUGCAGCCU
AUAAAUCGGAUUCUACAGGAGAGAGUUAAAA
>random_seq_from_cds__NO_10385 RANDOM_LENGTH 88 SEQ_LENGTH 81 GC_CONTENT 0.604938271604938 BASEPAIR 27 FREE_ENERGY -32.10 LEN_BP_RATIO 3
CGGCUGACCUGGUGGUGUUUGCUGAGCUGUUUUGGAACCCAGGGGGUGCUGAUCCAGGCUGAGGA
CCGCGUGCACCGCAUUGGACAGA
>random_seq_from_cds__NO_10391 RANDOM_LENGTH 70 SEQ_LENGTH 53 GC_CONTENT 0.641509433962264 BASEPAIR 19 FREE_ENERGY -18.70 LEN_BP_RATIO 2.78947368421053
GUAACAUGGGCCAAACGUACCAAGAAAGUCGGGAUCGUCGGUAAAUACGGGACCCGCUAUGGGGC
CUCCC
>random_seq_from_cds__NO_10393 RANDOM_LENGTH 84 SEQ_LENGTH 59 GC_CONTENT 0.627118644067797 BASEPAIR 23 FREE_ENERGY -25.00 LEN_BP_RATIO 2.56521739130435
GAAGAGACGAGCUGUGGGGAUCUGGCACUGUGGUUCCUGCAUGAAGACAGUGGCUGGCGGUGCCU
GGACGUACAAGUACCACUU
>random_seq_from_cds__NO_10400 RANDOM_LENGTH 70 SEQ_LENGTH 58 GC_CONTENT 0.620689655172414 BASEPAIR 21 FREE_ENERGY -27.70 LEN_BP_RATIO 2.76190476190476
GGCAGGGCCAAUGGGAUGAUGGAGUGCUGGUAGACCAGGGCAGACAGCGAUCCCGAAGUUUGGCU
CAUUG
>random_seq_from_cds__NO_10402 RANDOM_LENGTH 87 SEQ_LENGTH 69 GC_CONTENT 0.565217391304348 BASEPAIR 24 FREE_ENERGY -24.50 LEN_BP_RATIO 2.875
CCCUUUUUUAUUCUGCUGCAUGAUGGUUGGAGGUGGCGAAGACACCUUCAUGGCCAGCCCGUACG
CGCCUCGGAAGGAGUGACUGUC
>random_seq_from_cds__NO_10406 RANDOM_LENGTH 110 SEQ_LENGTH 101 GC_CONTENT 0.673267326732673 BASEPAIR 36 FREE_ENERGY -48.00 LEN_BP_RATIO 2.80555555555556
GUCUCCCACUGACAUCCUUCGCUUCUCUGGCAAGGCUGGUGUUGGGCUCCCUUGCCGGAAGGCUG
CGGAGUCUGGUGACGGGGAGGUGGCAGGGCUGCUCAGGCCAGGGU
>random_seq_from_cds__NO_10409 RANDOM_LENGTH 89 SEQ_LENGTH 79 GC_CONTENT 0.658227848101266 BASEPAIR 31 FREE_ENERGY -37.90 LEN_BP_RATIO 2.54838709677419
CCCAGGCUGGGGCUUCCAGGGCUGGCUAUUGCAUUGCUAUGAAGACCGGAGGCCAGGUUGCCUUG

GUGAGCCCCAGGGUGCCGGCCCAG
>random_seq_from_cds__NO_10422 RANDOM_LENGTH 71 SEQ_LENGTH 63 GC_CONTENT 0.587301587301587 BASEPAIR 26 FREE_ENERGY -30.90 LEN_BP_RATIO 2.42307692307692
CUGGACAGGGGAGCGAACCCCAGGGCUACGAGGGGAUGUGGCUUCUGGAGACUGGUUCUCAUACUGUCCAU
>random_seq_from_cds__NO_10424 RANDOM_LENGTH 92 SEQ_LENGTH 85 GC_CONTENT 0.588235294117647 BASEPAIR 28 FREE_ENERGY -30.90 LEN_BP_RATIO 3.03571428571429
CUGCUACCCUGUGGGCCACCAGCCCUUCUAAAUUCAAUGGCUCCUCCUCUGGAGUCCGGGAGGGGUCUGAAGUCGCUUCCUUUGGUGAGAGC
>random_seq_from_cds__NO_10429 RANDOM_LENGTH 110 SEQ_LENGTH 98 GC_CONTENT 0.612244897959184 BASEPAIR 32 FREE_ENERGY -35.10 LEN_BP_RATIO 3.0625
UGUGUAGUGGGGCAGCAGCGGAGGCUGGCUCCAGGUCCAGCAUCAGCAUAUUGAGUGUUUCGAUGGACUGUUCAAUCUCCUGCUGGGAGGCUGCUCGCGGGAACUCAGGG
>random_seq_from_cds__NO_10430 RANDOM_LENGTH 71 SEQ_LENGTH 61 GC_CONTENT 0.59016393442623 BASEPAIR 21 FREE_ENERGY -24.30 LEN_BP_RATIO 2.9047619047619
AGACUGGGGAUGGGGGUCUCUGCCAAUGGCUGAGGGCUGGGCCUGCUGGCUACAAGACUCUCCAAGUGGGC
>random_seq_from_cds__NO_10433 RANDOM_LENGTH 72 SEQ_LENGTH 63 GC_CONTENT 0.666666666666667 BASEPAIR 23 FREE_ENERGY -34.50 LEN_BP_RATIO 2.73913043478261
GGAGGUGGUCACUGGCUGUGGCCAGGCUGGGUGGGGCCCCUCCCUCUGGUAGCCAGCUAAACCCUCCUGUGC
>random_seq_from_cds__NO_10435 RANDOM_LENGTH 67 SEQ_LENGTH 54 GC_CONTENT 0.666666666666667 BASEPAIR 20 FREE_ENERGY -23.20 LEN_BP_RATIO 2.7
ACUUGUCCAGACCGUUGGUCAGUGGGGACAGGGCCUCUGGGUAGCCCCCCUCACUGGUGUUGGUGAC
>random_seq_from_cds__NO_10442 RANDOM_LENGTH 71 SEQ_LENGTH 66 GC_CONTENT 0.651515151515151 BASEPAIR 23 FREE_ENERGY -21.60 LEN_BP_RATIO 2.8695652173913
CCUCCCUCCAUGCCGUCAUCUCGAUGCCCACUGAAGUUGUCGUAGGAGUCCCAGCGGAUGAGGGGUCGGA
>random_seq_from_cds__NO_10446 RANDOM_LENGTH 72 SEQ_LENGTH 55 GC_CONTENT 0.581818181818182 BASEPAIR 20 FREE_ENERGY -22.80 LEN_BP_RATIO 2.75
CAAGAGCAGUCCUGGCUCGAUGGUGAUGCAGACGCUAGUCUGGCUGUCUCCUGGGAUGUUGCUAGAUGCCAG
>random_seq_from_cds__NO_10452 RANDOM_LENGTH 87 SEQ_LENGTH 62 GC_CONTENT 0.548387096774194 BASEPAIR 21 FREE_ENERGY -20.30 LEN_BP_RATIO 2.95238095238095

GCCAAAUUCCAGUACCCUUGGCAUGGAGCUUCGUGAUGUCAGGUCUCCGCUCAGAGAGGUUGAAC
AGCCAGGUAGUUGCCUCCAUGU
>random_seq_from_cds__NO_10465 RANDOM_LENGTH 71 SEQ_LENGTH 58 GC_CONTENT 0.551724137931034 BASEPAIR 19 FREE_ENERGY -17.71 LEN_BP_RATIO 3.05263157894737
CAGACCUCCUCCUUAGUGUCCACCUGCCACACUUUCAAGAGGCCACUCAUGUCCCCUGUGGCCAC
UAGAGU
>random_seq_from_cds__NO_10469 RANDOM_LENGTH 102 SEQ_LENGTH 67 GC_CONTENT 0.611940298507463 BASEPAIR 21 FREE_ENERGY -21.46 LEN_BP_RATIO 3.19047619047619
CGUUGCCCUCUUCCUCCUCUUCUUCCUCAAAGUCCACAUCUUCCAUCUCCUGGGCCAGGUCAUCC
UGGGUCCGGCGGACCGGGAUCAAGUUCUACCACCUCG
>random_seq_from_cds__NO_10484 RANDOM_LENGTH 87 SEQ_LENGTH 68 GC_CONTENT 0.602941176470588 BASEPAIR 23 FREE_ENERGY -25.60 LEN_BP_RATIO 2.95652173913043
AAAACCCACACUGUGGGCUCCGUGGGCCAAAGUGGAACAGGUGAAGUUCGAUGCCACAUCCAUGC
AUGUCAAGCCUCAGGUGGCUGC
>random_seq_from_cds__NO_10499 RANDOM_LENGTH 99 SEQ_LENGTH 62 GC_CONTENT 0.532258064516129 BASEPAIR 20 FREE_ENERGY -19.36 LEN_BP_RATIO 3.1
CCUGUCCAUUGAAGAUUUCACUCAGGCCUUUGGGAUGACUCCAGCUGCCUUCUCUGCUCUGCCUC
GAUGGAAGCAACAAAACCUCAAGAAAGAAAAAGG
>random_seq_from_cds__NO_10500 RANDOM_LENGTH 90 SEQ_LENGTH 71 GC_CONTENT 0.802816901408451 BASEPAIR 28 FREE_ENERGY -47.40 LEN_BP_RATIO 2.53571428571429
ACUAUUUUGACAUGGCUGCGCUGGGCUGCGCGAGGCUGAGGUGGGCGCUGCGAGGGGCCGGCCGU
GGCCUCUGCCCCCACGGGGCCAGAG
>random_seq_from_cds__NO_10506 RANDOM_LENGTH 70 SEQ_LENGTH 59 GC_CONTENT 0.559322033898305 BASEPAIR 22 FREE_ENERGY -16.60 LEN_BP_RATIO 2.68181818181818
GGUGAUUGAUGACUUUAUGACUCGACUGGACCAGCUGCGGGCAGAGAGUGCUUCGGGGAACCAGG
UGUCG
>random_seq_from_cds__NO_10509 RANDOM_LENGTH 84 SEQ_LENGTH 57 GC_CONTENT 0.526315789473684 BASEPAIR 22 FREE_ENERGY -25.00 LEN_BP_RATIO 2.59090909090909
CCCGUGCUGCCUUUCUGGAAGCGAUACCUGGAUGGUUGGAAUGCCAUCUUUUCCUUUGGGGAAGA
AGCUGAUUGAUGAGAAGCU
>random_seq_from_cds__NO_10526 RANDOM_LENGTH 82 SEQ_LENGTH 76 GC_CONTENT 0.684210526315789 BASEPAIR 25 FREE_ENERGY -28.30 LEN_BP_RATIO 3.04
UGCGGAGCCACACGCGCACCGAGUGCAAAUGCCACGGGCUGUCGGGAUCAUGCGCGCUGCGCACC
UGCUGGCAGAAGCUGCC
>random_seq_from_cds__NO_10529 RANDOM_LENGTH 97 SEQ_LENGTH 84 GC_CONTENT 0.773809523809524 BASEPAIR 31 FREE_ENERGY

-45.80 LEN_BP_RATIO 2.70967741935484
ACGCGCGGUCGCGCCUGCAAUAGCAGCGCCCCGGACCUCAGCGGCUGCGACCUGCUGUGCUGCGG
CCGCGGGCACCGCCAGGAGAGCGUGCAGCUCG
>random_seq_from_cds__NO_10543 RANDOM_LENGTH 84 SEQ_LENGTH 78 GC_CONTENT 0.705128205128205 BASEPAIR 27 FREE_ENERGY -39.40 LEN_BP_RATIO 2.88888888888889
GGCAGCAUGUGCUGCGGCCGCGGCCACAACAUCCUGCGCCAGACGCGCAGCGAGCGCUGCCACUG
CCGCUUCCACUGGUGCUGU
>random_seq_from_cds__NO_10545 RANDOM_LENGTH 90 SEQ_LENGTH 62 GC_CONTENT 0.580645161290323 BASEPAIR 21 FREE_ENERGY -23.51 LEN_BP_RATIO 2.95238095238095
CAGAGGUUUCUUCCUGUCCCUGCUGCAGCUGCCACAUGUCAGCAUACACCCCACCUCGGGACAAC
AGAGCCUCGUGUCCGUCCCCUCUCC
>random_seq_from_cds__NO_10550 RANDOM_LENGTH 73 SEQ_LENGTH 65 GC_CONTENT 0.523076923076923 BASEPAIR 19 FREE_ENERGY -21.14 LEN_BP_RATIO 3.42105263157895
UCAUUCCCAGCUGUGACACGGCCGUAACGGAUAUUGUCGGCGAUGGUGUCAUUAAAGAGGACAGU
GUCUUGGG
>random_seq_from_cds__NO_10553 RANDOM_LENGTH 110 SEQ_LENGTH 92 GC_CONTENT 0.630434782608696 BASEPAIR 31 FREE_ENERGY -35.92 LEN_BP_RATIO 2.96774193548387
AAGACACGUCCUGCAGAGUCUCCCGCCCCAUCGGCAUAGCUGAAGUGCACGUUCUCAAACUCAAU
ACGGCCCUUCUGAAAGCGAAGGGGCCCUGCUCCAGGAAGGUCCUU
>random_seq_from_cds__NO_10554 RANDOM_LENGTH 85 SEQ_LENGTH 76 GC_CONTENT 0.460526315789474 BASEPAIR 25 FREE_ENERGY -17.00 LEN_BP_RATIO 3.04
CACCUUCUGUCUCCUCUUUCAGCAAGUCAAACAUGUUCUCCAUGUCAAUGAAGUUGGUCUGGAUC
AUCCCUGUAGUAGGUGCCAA
>random_seq_from_cds__NO_10580 RANDOM_LENGTH 85 SEQ_LENGTH 72 GC_CONTENT 0.680555555555556 BASEPAIR 29 FREE_ENERGY -35.40 LEN_BP_RATIO 2.48275862068966
UGACGAUGACGGUGCAGCCGCUCUCCCACACCAUCCUGCCAGAAGUCUGCGAUGGUAUGGGACAG
CGGGCCCUGCGUGGCUAUGU
>random_seq_from_cds__NO_10585 RANDOM_LENGTH 110 SEQ_LENGTH 100 GC_CONTENT 0.61 BASEPAIR 36 FREE_ENERGY -37.90 LEN_BP_RATIO 2.77777777777778
GGACACACUGCUCACCCGUGAAGGCUCCGGUGGACCCUCUGCCCGGUUGAACAAGGACUUCGUGG
CCAUGUGCUGGCGGCACAGGUCCCUGGUACUCAAAGGUAGUGUCA
>random_seq_from_cds__NO_10590 RANDOM_LENGTH 95 SEQ_LENGTH 84 GC_CONTENT 0.630952380952381 BASEPAIR 28 FREE_ENERGY -33.70 LEN_BP_RATIO 3
CUCCAGCAGCUUCACUCCUGCAGCCAGGCUCAGGGGCCUUCUGAUCAGUGACGAUGUAGCCAUAU
UCCUCUGCUGCUGGGCGGGCUGAGGGCUGU
>random_seq_from_cds__NO_10594 RANDOM_LENGTH 78 SEQ_LENGTH 71

GC_CONTENT 0.647887323943662 BASEPAIR 26 FREE_ENERGY -30.20 LEN_BP_RATIO 2.73076923076923
CAACCCCUCCCCGGAUUUCUUCCUGCACCCUUGGGCAGUAGCUGCAGCAGGGUCAGGAGUGUGGAGAGCUGCUCAGGG
>random_seq_from_cds__NO_10599 RANDOM_LENGTH 82 SEQ_LENGTH 62 GC_CONTENT 0.693548387096774 BASEPAIR 21 FREE_ENERGY -31.90 LEN_BP_RATIO 2.95238095238095
GGCAGGGGCUUCAGCCUUGGGCAGGGGGCCGACACUGACCAUCCCUGGGGAGCCCUCUGAGACCCUGGAGCCAUCACGGGAG
>random_seq_from_cds__NO_10602 RANDOM_LENGTH 78 SEQ_LENGTH 69 GC_CONTENT 0.681159420289855 BASEPAIR 25 FREE_ENERGY -26.90 LEN_BP_RATIO 2.76
GGGCAGCAGGGGCGGAGCCAGUGGGGAUGUCCUGUAAAAGCAGCUCUCCAGCAGGACCAGGUCUCUUGGGUGCCAAGC
>random_seq_from_cds__NO_10621 RANDOM_LENGTH 81 SEQ_LENGTH 59 GC_CONTENT 0.440677966101695 BASEPAIR 20 FREE_ENERGY -17.10 LEN_BP_RATIO 2.95
CAAAAGCUAUGAUGGUGGAGGAGUUCCUGGUCAUGAAGUACCUUGCUCUCGGGCUUAAUAUUCCAUUUCUCAGUCUCCUUG
>random_seq_from_cds__NO_10633 RANDOM_LENGTH 95 SEQ_LENGTH 80 GC_CONTENT 0.55 BASEPAIR 27 FREE_ENERGY -32.20 LEN_BP_RATIO 2.96296296296296
GGACAUCCGGGCUCAGUAUGAGACCAUCGCGGCUAAGAACAUUUCUGAAGCUGAGGAGUGGUACAAGUCGAAGGGUGUCAGACCUGACCCAGGCA
>random_seq_from_cds__NO_10644 RANDOM_LENGTH 81 SEQ_LENGTH 72 GC_CONTENT 0.680555555555556 BASEPAIR 27 FREE_ENERGY -32.90 LEN_BP_RATIO 2.66666666666667
CAAUAGCUCUGAGCCCCUGCUAGGCCUGCUGGCACUGUCACCGGGAGGACCCGUGGCUGUGCCCAUGUCUUUGGGCCAUGC
>random_seq_from_cds__NO_10664 RANDOM_LENGTH 86 SEQ_LENGTH 65 GC_CONTENT 0.692307692307692 BASEPAIR 22 FREE_ENERGY -31.10 LEN_BP_RATIO 2.95454545454545
ACAGCGGCUGACAUAGGGGCGGAUCGCCUCUUCAGAGAGUGGCAUCAUGGGGAGGGGCGCAGCGCUGGCCAGCCAGUAGGAUCUGU
>random_seq_from_cds__NO_10677 RANDOM_LENGTH 73 SEQ_LENGTH 68 GC_CONTENT 0.632352941176471 BASEPAIR 21 FREE_ENERGY -26.64 LEN_BP_RATIO 3.23809523809524
GCCAGGUGAUCCGGAGGGACCCUUUUAUUCCCGGAGGACCUGGUAUCCCUGGAUCCCCCUGGAGGCCUCUUGG
>random_seq_from_cds__NO_10683 RANDOM_LENGTH 98 SEQ_LENGTH 86 GC_CONTENT 0.523255813953488 BASEPAIR 24 FREE_ENERGY -30.42 LEN_BP_RATIO 3.58333333333333
CUUGCAUCCCGGGAGUUCCUUUAUCACCCUCUUUCUCCUGGGAAUCCAUCAUCUCCAGGAGGUCCAGGUUCCCCAGGUGUUCCCUUUUGUGAAAUGAU

>random_seq_from_cds__NO_10686 RANDOM_LENGTH 84 SEQ_LENGTH 74 GC_CONTENT 0.662162162162162 BASEPAIR 27 FREE_ENERGY -40.10 LEN_BP_RATIO 2.74074074074074
ACCAGGCAGCCCCCGGGGUCCCCUUUGGACCUGGAGGACCAGGUAGCCCAUCAUCUCCAAAGGGA
CCUGGGAUUCCUGGGAGGC
>random_seq_from_cds__NO_10715 RANDOM_LENGTH 110 SEQ_LENGTH 89 GC_CONTENT 0.651685393258427 BASEPAIR 31 FREE_ENERGY -49.50 LEN_BP_RATIO 2.87096774193548
UGAAAGCCCAAUGGGUCCUGGGGCUCCCAGGGGUCCAAUUGGACCCUGUGGCCCUGGUGGUCCUG
GUGGACCCCCGAGACCCCUUUUCAGGAACACAGUGGCAAACAGAG
>random_seq_from_cds__NO_10728 RANDOM_LENGTH 74 SEQ_LENGTH 57 GC_CONTENT 0.526315789473684 BASEPAIR 19 FREE_ENERGY -17.60 LEN_BP_RATIO 3
CCCCUAUUGCUUCUGUGCCUGGAUCCACUAAUACGGGUACUGUUCCGGGAUCAGAGAAAGACAGG
UGACUCGAU
>random_seq_from_cds__NO_10734 RANDOM_LENGTH 86 SEQ_LENGTH 65 GC_CONTENT 0.415384615384615 BASEPAIR 23 FREE_ENERGY -16.70 LEN_BP_RATIO 2.82608695652174
AUCAGGAAGGUGGAGGUCUCUAUGCACUAGGUCUUAUUCAUGCCAAUCAUGGUGGUGAUAUAAUU
GACUAUCUGCUUAAUCAGCUU
>random_seq_from_cds__NO_10756 RANDOM_LENGTH 110 SEQ_LENGTH 69 GC_CONTENT 0.550724637681159 BASEPAIR 20 FREE_ENERGY -18.30 LEN_BP_RATIO 3.45
UUAUUUCCAUCAAUUUCACCGUCUUCCAUGGCCUCCUUUGGCAUCCUCCUCACUGUUGAAGUCUA
CAAAACCAAACCCCUUUGGAGGACCCAGUUUCCCGGUCAGUAACU
>random_seq_from_cds__NO_10773 RANDOM_LENGTH 84 SEQ_LENGTH 67 GC_CONTENT 0.567164179104478 BASEPAIR 21 FREE_ENERGY -24.30 LEN_BP_RATIO 3.19047619047619
UGUGGCUCCCUUCUUGCCAGGUGUGGUAACUGCUUUGGCUGGUGUAACUGUCUUCUUGGCAGGUG
UUGCUGCUGCCUUUUUGCC
>random_seq_from_cds__NO_10778 RANDOM_LENGTH 84 SEQ_LENGTH 75 GC_CONTENT 0.666666666666667 BASEPAIR 25 FREE_ENERGY -33.30 LEN_BP_RATIO 3
AUCCCAGGUUGAGGAGGAGAACCCGGACUUCUGGAACCGCGAGGCAGCCGAGGCCCUGGGUGCCG
CCAAGAAGCUGCAGCCUGC
>random_seq_from_cds__NO_10788 RANDOM_LENGTH 86 SEQ_LENGTH 73 GC_CONTENT 0.575342465753425 BASEPAIR 28 FREE_ENERGY -29.50 LEN_BP_RATIO 2.60714285714286
UGGAGGGGUGGUCGCAUCGACCAUGGUCAUCAUGAAAGCAGGGCUUACCGGGCACUGACUGAGAC
GAUCAUGUUCGACGACGCCAU
>random_seq_from_cds__NO_10794 RANDOM_LENGTH 72 SEQ_LENGTH 57 GC_CONTENT 0.719298245614035 BASEPAIR 21 FREE_ENERGY -28.00 LEN_BP_RATIO 2.71428571428571
GCGUUGCUUCCUCUGCUGGCCGGGACCCUGCUGCUGCUGGAGACGGCCACUGCUCCCUGACAUGC

AGGGGCC
>random_seq_from_cds__NO_10823 RANDOM_LENGTH 82 SEQ_LENGTH 73 GC_CONTENT 0.657534246575342 BASEPAIR 24 FREE_ENERGY -31.92 LEN_BP_RATIO 3.04166666666667
CCCUGAUGGAGAUGACAGAGGCUGCCCUGCGCCUGCUGAGCAGGAACCCCCGCGGCUUCUACCUCUUUGUGGAGGGGCGGCC
>random_seq_from_cds__NO_10825 RANDOM_LENGTH 86 SEQ_LENGTH 68 GC_CONTENT 0.602941176470588 BASEPAIR 23 FREE_ENERGY -25.71 LEN_BP_RATIO 2.95652173913043
CCAGCUCACCAGCGAGGAGGACACGCUGACCCUCGUCACCGCUGACCACUCCCAUGUCUUCUCCUUUGGUGGCUACACCUUGCGAG
>random_seq_from_cds__NO_10833 RANDOM_LENGTH 77 SEQ_LENGTH 72 GC_CONTENT 0.652777777777778 BASEPAIR 26 FREE_ENERGY -36.90 LEN_BP_RATIO 2.76923076923077
UGUGUGUACUUGAGCCGGGGAAGUGGGUGCUCUGGGCCGUGCUCCCGCACCCACUUCUGAUAGGCCGUGGUAGGCCA
>random_seq_from_cds__NO_10835 RANDOM_LENGTH 90 SEQ_LENGTH 72 GC_CONTENT 0.597222222222222 BASEPAIR 27 FREE_ENERGY -27.80 LEN_BP_RATIO 2.66666666666667
GAAGUUGUCAUAGAGACGGACGAUGCACUCAGCCUUUCGCAGGAAGCGGCUGUAGGAGGCCUCCGUCCACCAGUGCAGCAGGUUCCCUGA
>random_seq_from_cds__NO_10842 RANDOM_LENGTH 106 SEQ_LENGTH 79 GC_CONTENT 0.708860759493671 BASEPAIR 29 FREE_ENERGY -44.30 LEN_BP_RATIO 2.72413793103448
AGAGGGCGCCAAGCGCCAUGCCAAAGUGGCGAUUGGCCUGGCCCAAGCAGACCCGGGCCAGCUCCUGUGGCUUGUCGCUGCCCUCCAUCUCCUGUGCCAGCUCGUG
>random_seq_from_cds__NO_10844 RANDOM_LENGTH 89 SEQ_LENGTH 79 GC_CONTENT 0.582278481012658 BASEPAIR 25 FREE_ENERGY -21.00 LEN_BP_RATIO 3.16
AUGUAGUCUGUCGCCAGCAGCACCACCUCCUCUUCCUCUGAGAAGUCCUCCUGGAAGAUCUGGUCUAGCAGCCACUUCCACCGCAACGU
>random_seq_from_cds__NO_10863 RANDOM_LENGTH 85 SEQ_LENGTH 77 GC_CONTENT 0.584415584415584 BASEPAIR 26 FREE_ENERGY -29.20 LEN_BP_RATIO 2.96153846153846
AGGAGAACCGCACCUACCCCGUGGAGUGGAUCAUCAUUGAUCCUGAAGGCUUCACAGGAGAACGGGGAGUGGGAGAUAGUCCACC
>random_seq_from_cds__NO_10866 RANDOM_LENGTH 71 SEQ_LENGTH 58 GC_CONTENT 0.586206896551724 BASEPAIR 20 FREE_ENERGY -23.10 LEN_BP_RATIO 2.9
AGGUGGUGAGAAGACAUCAGUGGCCAUCUCGGUGCUCCUGGCUCAGUCUGUCUUCCUGCUGCUCAUCUCCA
>random_seq_from_cds__NO_10869 RANDOM_LENGTH 81 SEQ_LENGTH 69 GC_CONTENT 0.710144927536232 BASEPAIR 24 FREE_ENERGY -30.50 LEN_BP_RATIO 2.875

CUGCCGGAGCUCCUGCACAUGUCCCGCCCAGCAGAGGAUGGACCCAGCCCUGGGGCCCUGGUGCG
GAGGAGCAGCUCCCUG
>random_seq_from_cds__NO_10888 RANDOM_LENGTH 77 SEQ_LENGTH 49
GC_CONTENT 0.673469387755102 BASEPAIR 19 FREE_ENERGY
-24.60 LEN_BP_RATIO 2.57894736842105
CUGAAGCAAGCCUUCCCAGCUGAGUACCUGCCUCAGUGACAUGGCAGCCAGCGGGAAGACCAGCA
AGUCCGAACCGA
>random_seq_from_cds__NO_10891 RANDOM_LENGTH 79 SEQ_LENGTH 69
GC_CONTENT 0.594202898550725 BASEPAIR 19 FREE_ENERGY
-17.14 LEN_BP_RATIO 3.63157894736842
UCACUCUGACCUGCGCCUUCCGCUAUGGCCAAGAGGACAUUGACGUGAUCGGCUUGACCUUCCGC
AGGGACCUGUACUU
>random_seq_from_cds__NO_10898 RANDOM_LENGTH 94 SEQ_LENGTH 69
GC_CONTENT 0.434782608695652 BASEPAIR 24 FREE_ENERGY
-17.00 LEN_BP_RATIO 2.875
UAACACAGAGAAGACCGUGAAGAAGAUUAAAGCAUUCGGUGGAACAGGUGGCCAAUGUGGUUCUC
UACUCGAGUGAUUAUUACGUCAAGCCCGU
>random_seq_from_cds__NO_10899 RANDOM_LENGTH 86 SEQ_LENGTH 71
GC_CONTENT 0.577464788732394 BASEPAIR 23 FREE_ENERGY
-23.04 LEN_BP_RATIO 3.08695652173913
GGCUAUGGAGGAAGCGCAGAGAAAAAGUGCCACCAAACAGCACUUUGACCAAGACGCUGACGCUG
CUGCCCUUGCUGGCUAACAAU
>random_seq_from_cds__NO_10904 RANDOM_LENGTH 70 SEQ_LENGTH 62
GC_CONTENT 0.741935483870968 BASEPAIR 24 FREE_ENERGY
-31.40 LEN_BP_RATIO 2.58333333333333
GACGUUGAUGAGUGACAUGGCCCGGGCCCUGUGCCGCCUCCCGCGGCGCGGCCUCUGGCUGCUCC
UGGGC
>random_seq_from_cds__NO_10906 RANDOM_LENGTH 82 SEQ_LENGTH 78
GC_CONTENT 0.641025641025641 BASEPAIR 27 FREE_ENERGY
-30.90 LEN_BP_RATIO 2.88888888888889
CAGUUCCAGGUAGACAUGGAGGCCGUCGGGGAGACGCUGUGGUGUGACUGGGGCAGGACCAUCAG
GGAGCUACAGGGAGCUG
>random_seq_from_cds__NO_10907 RANDOM_LENGTH 68 SEQ_LENGTH 61
GC_CONTENT 0.639344262295082 BASEPAIR 21 FREE_ENERGY
-26.80 LEN_BP_RATIO 2.9047619047619
GCCGACUGCACCUGGCACAUGGCGGAGAAGCUGGGCUGCUUCUGGCCCAAUGCAGAGGUGGACAG
GUU
>random_seq_from_cds__NO_10910 RANDOM_LENGTH 94 SEQ_LENGTH 81
GC_CONTENT 0.679012345679012 BASEPAIR 24 FREE_ENERGY
-35.20 LEN_BP_RATIO 3.375
GCACUGAGGGCAUUGUGUAGCAUGGCGGAGGGCGGCAGCCCAGACGGGCGGGCAGGGCCGGGCUC
CGCAGGGUCGUAAUCUGAAGGAGUGGCUG
>random_seq_from_cds__NO_10924 RANDOM_LENGTH 78 SEQ_LENGTH 66
GC_CONTENT 0.46969696969697 BASEPAIR 23 FREE_ENERGY

-21.30 LEN_BP_RATIO 2.8695652173913
AUCUCCAUGACUUUCUCCAUGGAACGGACAGCGUUUGCAUUGGGUCUUUGCUGUAAAACCUUUUC
UGGGAGAGGAUCA
>random_seq_from_cds__NO_10928 RANDOM_LENGTH 77 SEQ_LENGTH 65
GC_CONTENT 0.723076923076923 BASEPAIR 22 FREE_ENERGY
-30.80 LEN_BP_RATIO 2.95454545454545
GGGGGGGUCCAGGCCGCCGGUGAAAGCCAUGUUGACGUUGAAACCCACGCCGGGCCCUGUGCCCA
CCCUCAUCAGGA
>random_seq_from_cds__NO_10934 RANDOM_LENGTH 68 SEQ_LENGTH 55
GC_CONTENT 0.636363636363636 BASEPAIR 21 FREE_ENERGY
-23.00 LEN_BP_RATIO 2.61904761904762
CGGUUGAGGGGGUUCGUGCCAUACAGGAGGGUGUGGGCUUCCGAGUGCACCGUCUGUAGCUCCUC
CAG
>random_seq_from_cds__NO_10945 RANDOM_LENGTH 69 SEQ_LENGTH 51
GC_CONTENT 0.666666666666667 BASEPAIR 19 FREE_ENERGY
-19.90 LEN_BP_RATIO 2.68421052631579
GUGCUUGUGCCGGCGGCUGCUCCAGUAAGACCAUGUGCUGCAGAAGAGGGCUGUGCGCUGCCCCU
CCGU
>random_seq_from_cds__NO_10953 RANDOM_LENGTH 71 SEQ_LENGTH 58
GC_CONTENT 0.586206896551724 BASEPAIR 22 FREE_ENERGY
-25.50 LEN_BP_RATIO 2.63636363636364
CAGUAGCGAGGGUCGCUGGAAAUGCAGUGGUUCAGAUUCCGGUGGGCCAGCGCCUUCUUUUUAUU
GAGGAC
>random_seq_from_cds__NO_10976 RANDOM_LENGTH 72 SEQ_LENGTH 51
GC_CONTENT 0.705882352941177 BASEPAIR 20 FREE_ENERGY
-29.20 LEN_BP_RATIO 2.55
ACGGGAUGGCCAGAGGCCGGGUACCUCCCCGAGGUCAUGGGUGACGGCCUGGCCAACCAGAUCAA
CAACCCC
>random_seq_from_cds__NO_10982 RANDOM_LENGTH 88 SEQ_LENGTH 76
GC_CONTENT 0.671052631578947 BASEPAIR 28 FREE_ENERGY
-39.40 LEN_BP_RATIO 2.71428571428571
UCCUGCUGGGGCCUGGUCCUUCCAACCUGCCUCCUCGCAUCAUGGCAGCCGGGGGGCUGCAGAUG
AUCGGGUCCAUGAGCAAGGAUAU
>random_seq_from_cds__NO_10984 RANDOM_LENGTH 92 SEQ_LENGTH 82
GC_CONTENT 0.670731707317073 BASEPAIR 30 FREE_ENERGY
-34.50 LEN_BP_RATIO 2.73333333333333
CCUGGAGGCCGCCCUGGUCAAUGUGCUGGAGCCUGGGGACUCCUUCCUGGUUGGGGCCAAUGGCA
UUUGGGGGCAGCGAGCCGUGGACAUCG
>random_seq_from_cds__NO_10986 RANDOM_LENGTH 75 SEQ_LENGTH 58
GC_CONTENT 0.672413793103448 BASEPAIR 22 FREE_ENERGY
-24.40 LEN_BP_RATIO 2.63636363636364
CCUGGCCCAGCACAAGCCAGUGCUGCUGUUCUUAACCCACGGGGAGUCGUCCACCGGCGUGCUGC
AGCCCCUUGA
>random_seq_from_cds__NO_10987 RANDOM_LENGTH 88 SEQ_LENGTH 67

GC_CONTENT 0.686567164179104 BASEPAIR 23 FREE_ENERGY -34.60 LEN_BP_RATIO 2.91304347826087
UGGCUUCGGGGAACUCUGCCACAGGGUACAAGUGCCUGCUCCUGGUGGAUUCGGUGGCAUCCCUGGGCGGGACCCCCCUUUACAUGGA
>random_seq_from_cds__NO_10991 RANDOM_LENGTH 89 SEQ_LENGTH 75 GC_CONTENT 0.746666666666667 BASEPAIR 31 FREE_ENERGY -37.70 LEN_BP_RATIO 2.41935483870968
GCACCGCGAGGCCGCGGCGUAUCUGCAUGGGCGCCUGCAGGCACUGGGGCUGCAGCUCUUCGUGAAGGACCCGGGCGCUCCGGCUUCCC
>random_seq_from_cds__NO_10999 RANDOM_LENGTH 68 SEQ_LENGTH 59 GC_CONTENT 0.745762711864407 BASEPAIR 22 FREE_ENERGY -34.80 LEN_BP_RATIO 2.68181818181818
UGGGCCACACGCCUGAGGCCGUGCAGAUCGCCACGGGAAGGAACGCCGCGCGCCUCUGCUGUGUGGCC
>random_seq_from_cds__NO_11000 RANDOM_LENGTH 84 SEQ_LENGTH 74 GC_CONTENT 0.756756756756757 BASEPAIR 29 FREE_ENERGY -39.80 LEN_BP_RATIO 2.55172413793103
AGCCGUGACGCCGGCCUCUCGUGGGGCAGCGCCCGGGACCUCACCGAGGAGGCCAUCGGUGGUGCCGUGCAGGGACUGGGCCAC
>random_seq_from_cds__NO_11006 RANDOM_LENGTH 84 SEQ_LENGTH 60 GC_CONTENT 0.7 BASEPAIR 23 FREE_ENERGY -30.60 LEN_BP_RATIO 2.60869565217391
GUCCACGAACCCCCAGAGGAGGCUGCUGUAGACCCCCGUGGAGGCCAGGUGCCUGGUGGGCCCUUCAGCCGUCUGCAGCCUCGG
>random_seq_from_cds__NO_11009 RANDOM_LENGTH 80 SEQ_LENGTH 74 GC_CONTENT 0.72972972972973 BASEPAIR 28 FREE_ENERGY -33.10 LEN_BP_RATIO 2.64285714285714
UGGACCCGCGCAGCUGGACAGAGCCCUGGGUGAUCUACGAGGGCCCCAGCGGCUACUCCGACCUGGCGUCCAUCGGGCCG
>random_seq_from_cds__NO_11010 RANDOM_LENGTH 71 SEQ_LENGTH 60 GC_CONTENT 0.633333333333333 BASEPAIR 22 FREE_ENERGY -30.50 LEN_BP_RATIO 2.72727272727273
GCUCCUGAGGGGGGCCUGGUUUUUGCCUGCCUGUACGAGAGCGGGGCCAGGACCUCCUAUGAUGAGAUUUC
>random_seq_from_cds__NO_11019 RANDOM_LENGTH 82 SEQ_LENGTH 71 GC_CONTENT 0.535211267605634 BASEPAIR 23 FREE_ENERGY -21.54 LEN_BP_RATIO 3.08695652173913
UCUCAGACAUGGUGGUCCCUCGAUCAACCAAGACUCAUUACUGAGGACAAGGAAGGGGGCCUUUUCACUGUGACUCUGUUUC
>random_seq_from_cds__NO_11030 RANDOM_LENGTH 87 SEQ_LENGTH 64 GC_CONTENT 0.5 BASEPAIR 22 FREE_ENERGY -17.20 LEN_BP_RATIO 2.90909090909091
UGUUUGUAGGAGCCACUGAUUCAGCCGUGCCAUGUGCAAUGAUGUUGGAACUUGCUCGUGCCUUAGACAAGAAACUCCUUUCCUUAA

>random_seq_from_cds__NO_11045 RANDOM_LENGTH 75 SEQ_LENGTH 60 GC_CONTENT 0.45 BASEPAIR 22 FREE_ENERGY -23.40 LEN_BP_RATIO 2.72727272727273
GGGAUUUUUGCUGAUACAGAAAAUUUCCCUUGGCAGUAUUCUGUCAGGGAGUUUGUGUGCUGGAAUAGCAACAGG
>random_seq_from_cds__NO_11059 RANDOM_LENGTH 110 SEQ_LENGTH 106 GC_CONTENT 0.367924528301887 BASEPAIR 32 FREE_ENERGY -23.90 LEN_BP_RATIO 3.3125
ACUUUAUCCAACAGCUCGAUUGCCAAAGACCGUGUCAAAUGUUGUGUUACAAAAGGAUGCCUGUAAUAAUUUUUCAGCAGUAGGUCUUUUUUUCGGAUUUUUGGUAAGUG
>random_seq_from_cds__NO_11065 RANDOM_LENGTH 80 SEQ_LENGTH 73 GC_CONTENT 0.465753424657534 BASEPAIR 24 FREE_ENERGY -15.40 LEN_BP_RATIO 3.04166666666667
GCAAUUUGCAGUUCUGACAGAGGUCCAGUUACCGUGAUAAAUAUCCUGUAAAGAACCACCUCCACAAAACUCCAUGCAAA
>random_seq_from_cds__NO_11068 RANDOM_LENGTH 86 SEQ_LENGTH 68 GC_CONTENT 0.588235294117647 BASEPAIR 24 FREE_ENERGY -25.90 LEN_BP_RATIO 2.83333333333333
CCGAUGCGCUGAAUCAGCUCGAAGUCCUCCUGCGGGUUCCGGCGGGACAAAUCGAAGCCGGGGUUCAUCUCAUUUGUUUUUGGGCU
>random_seq_from_cds__NO_11071 RANDOM_LENGTH 92 SEQ_LENGTH 82 GC_CONTENT 0.51219512195122 BASEPAIR 27 FREE_ENERGY -23.10 LEN_BP_RATIO 3.03703703703704
GAAUCGGAGGUCAGUUUAGUUGGUGUGGCAAAUAUGAUAGGUGGUGCUUCUGUGGAAACCACAGGCUUUAAUCCCCUGCGGGCUAUAGGCCU
>random_seq_from_cds__NO_11074 RANDOM_LENGTH 90 SEQ_LENGTH 68 GC_CONTENT 0.720588235294118 BASEPAIR 22 FREE_ENERGY -26.50 LEN_BP_RATIO 3.09090909090909
ACCAGGAGGCUGUCAUCAGGGGCCAGGCUCAGGCGCCGUCCCCCCAUUGUCACCACCGAGGAGCCCUCCCAGCUGCCACAGCCACACGUC
>random_seq_from_cds__NO_11087 RANDOM_LENGTH 87 SEQ_LENGTH 81 GC_CONTENT 0.567901234567901 BASEPAIR 24 FREE_ENERGY -27.15 LEN_BP_RATIO 3.375
ACAUCCUUGGGCACCACAUCUCCACGGUACAGCAGGCAGCAGGCCAUGUACUUGCCGUGCCGGGGAUCACACUUUACCAUCUGGUUG
>random_seq_from_cds__NO_11089 RANDOM_LENGTH 77 SEQ_LENGTH 66 GC_CONTENT 0.606060606060606 BASEPAIR 25 FREE_ENERGY -22.00 LEN_BP_RATIO 2.64
UGACUGGUGCAUAGGUGGCCAGGGGGAAGUGGAUGCGAGGGUAGGGCACCAGGUUGGUCUGGAACUCUGUCAGGUCC
>random_seq_from_cds__NO_11102 RANDOM_LENGTH 82 SEQ_LENGTH 64 GC_CONTENT 0.46875 BASEPAIR 20 FREE_ENERGY -16.00 LEN_BP_RATIO 3.2
GAAAUUUCUGAACGAAUUGUACAGUGGCCUGCAAUAGACGUACAAACCUCUGUUGGACAAAGCUG

GUUUGGGAUCCAUAACU
>random_seq_from_cds__NO_11108 RANDOM_LENGTH 87 SEQ_LENGTH 57 GC_CONTENT 0.56140350877193 BASEPAIR 20 FREE_ENERGY -20.40 LEN_BP_RATIO 2.85
CUACAACAGUUUUUAAGGAGAUACUGCUUGGCUGUACUGCGGCAACUCCACCUAGUAAGGACCCAAGACAGCAAAGUACUCCCCAGG
>random_seq_from_cds__NO_11116 RANDOM_LENGTH 72 SEQ_LENGTH 59 GC_CONTENT 0.542372881355932 BASEPAIR 19 FREE_ENERGY -22.10 LEN_BP_RATIO 3.10526315789474
AUCGUGGCACAGUUGCCUAAAUGCCGAGAGUGUCGCUUGGACAGUCUCCGCAAGGAUAAGGAGCAACAGAAG
>random_seq_from_cds__NO_11121 RANDOM_LENGTH 85 SEQ_LENGTH 78 GC_CONTENT 0.525641025641026 BASEPAIR 24 FREE_ENERGY -17.86 LEN_BP_RATIO 3.25
ACUGGGUGUGUCCUCGGUGUGGGUUUGGAGUAUGUGUGGACUGCUACCGGAUGAAGAGAAAGAAUUGCCAACAGGGGUGCUGCUU
>random_seq_from_cds__NO_11125 RANDOM_LENGTH 84 SEQ_LENGTH 58 GC_CONTENT 0.620689655172414 BASEPAIR 22 FREE_ENERGY -28.40 LEN_BP_RATIO 2.63636363636364
GAAAAACCGACUCUUGGUGCAGUGCUCCAGCAGAAUCCCUCAGUGUUGGAGCCAGCAGCUGUGGGUGGGGAAGCAGCCUCCAAG
>random_seq_from_cds__NO_11140 RANDOM_LENGTH 77 SEQ_LENGTH 56 GC_CONTENT 0.428571428571429 BASEPAIR 22 FREE_ENERGY -18.60 LEN_BP_RATIO 2.54545454545455
UCAUUAAGAAAACGUCUUCAUCAAGAGUAUGGAGUUCAAGGCUGGGCUAUUGUACAGUUUCUUGGGGAUGUGGUGUU
>random_seq_from_cds__NO_11146 RANDOM_LENGTH 84 SEQ_LENGTH 67 GC_CONTENT 0.462686567164179 BASEPAIR 22 FREE_ENERGY -22.00 LEN_BP_RATIO 3.04545454545455
AAAAGGGGCCUUCUGUGGAUUGGGGAAAAAUCCAGAGACCCCCUGAAGAUUCGGAUUCAACCCUAUGAAAAGAUAAAGGCCAGG
>random_seq_from_cds__NO_11190 RANDOM_LENGTH 84 SEQ_LENGTH 70 GC_CONTENT 0.528571428571429 BASEPAIR 25 FREE_ENERGY -18.90 LEN_BP_RATIO 2.8
CCUGCUGCUGUAAUUGUGGAGAAGCCACUGAGUGUACCACCAGCCCAAGGACUUUCCAUUCCAGUGAUUGGCGCAAGAAGUUGA
>random_seq_from_cds__NO_11205 RANDOM_LENGTH 81 SEQ_LENGTH 56 GC_CONTENT 0.410714285714286 BASEPAIR 21 FREE_ENERGY -18.32 LEN_BP_RATIO 2.66666666666667
UUUCAGCCUCACUUGAUCAAUGUUGAAGGAGUUGGUGUUGCUGAAUUGCUUUUUAACACAAUUCAGGCAGCUGACAUUGAU
>random_seq_from_cds__NO_11208 RANDOM_LENGTH 87 SEQ_LENGTH 70 GC_CONTENT 0.485714285714286 BASEPAIR 26 FREE_ENERGY -19.70 LEN_BP_RATIO 2.69230769230769

GGUAUUCCUGGGUGGUGCAGUUCUAGCGGAUAUCAUGAAAGACAAAGACAACUUUUGGAUGACCC
GACAAGAGUACCAAGAAAAGGG
>random_seq_from_cds__NO_11209 RANDOM_LENGTH 73 SEQ_LENGTH 64 GC_CONTENT 0.46875 BASEPAIR 21 FREE_ENERGY -18.40 LEN_BP_RATIO 3.04761904761905
UGUCCGUGUGCUAGAGAAACUUGGUGUGACUGUUCGAUAAGAUGAAGUUCACAGAUUGUCUUUGC
UGGACUUC
>random_seq_from_cds__NO_11217 RANDOM_LENGTH 88 SEQ_LENGTH 76 GC_CONTENT 0.592105263157895 BASEPAIR 26 FREE_ENERGY -34.10 LEN_BP_RATIO 2.92307692307692
UCCUGGGCUGCCCGACUGGCAUUGGGCUUCACAUUCCAAGAAUACCUCCUGUGUGAAUAGUCCUC
UCCAGGGGGACCAGGAGGAGGGA
>random_seq_from_cds__NO_11226 RANDOM_LENGTH 84 SEQ_LENGTH 65 GC_CONTENT 0.6 BASEPAIR 20 FREE_ENERGY -20.74 LEN_BP_RATIO 3.25
AAACGAGUCCCAGGCAUCCGCUGUCCAACUGGGAGAUAGUCUUUCCACCCUUUCGGGGAUGUGGU
UUCCGCCCUUCUUCUUGGC
>random_seq_from_cds__NO_11258 RANDOM_LENGTH 84 SEQ_LENGTH 62 GC_CONTENT 0.516129032258065 BASEPAIR 21 FREE_ENERGY -17.20 LEN_BP_RATIO 2.95238095238095
GUUAUAGAAGGUGAUGGUGGUCUCUGCUCUUAAUAAGGCCUGGUGCGUGAACUGCUUUGCCUGUU
CUACCUGCAACACUAAAUU
>random_seq_from_cds__NO_11261 RANDOM_LENGTH 84 SEQ_LENGTH 67 GC_CONTENT 0.477611940298507 BASEPAIR 23 FREE_ENERGY -18.90 LEN_BP_RATIO 2.91304347826087
UCAGUUUGGCCUCUACAAAAAUCCGUUUGAGGAAAUCUCGAGUAUAGUCUGAGACAUGAGGUGGC
AGCUUUGGGUUUGUUGGCU
>random_seq_from_cds__NO_11291 RANDOM_LENGTH 88 SEQ_LENGTH 78 GC_CONTENT 0.512820512820513 BASEPAIR 28 FREE_ENERGY -23.70 LEN_BP_RATIO 2.78571428571429
AUUCGCACUGAGUUUGACCAGGAGAUUGACAUGGGCUCACUGAACCCGGGAAAGCAACUGGUUUG
AGAAGAUGAUCAGUGGGAUGUAC
>random_seq_from_cds__NO_11294 RANDOM_LENGTH 80 SEQ_LENGTH 72 GC_CONTENT 0.708333333333333 BASEPAIR 24 FREE_ENERGY -26.60 LEN_BP_RATIO 3
GGAGGACUGCGUGGCCACUCACCGGAUCUGCCAGAUCGUGUCCACACGCUCCGCCAGCCUGUGCG
CAGCCACCCUGGCCG
>random_seq_from_cds__NO_11299 RANDOM_LENGTH 82 SEQ_LENGTH 69 GC_CONTENT 0.652173913043478 BASEPAIR 20 FREE_ENERGY -19.80 LEN_BP_RATIO 3.45
CAAGGAGACUCAUGCCAGUGCCCCCGUCAAGAUGCUGCCCACCUACGUGUGUGCUACCCCGGACG
GCACAGGAGAAAGGGGA
>random_seq_from_cds__NO_11312 RANDOM_LENGTH 84 SEQ_LENGTH 61 GC_CONTENT 0.540983606557377 BASEPAIR 23 FREE_ENERGY

-21.20 LEN_BP_RATIO 2.65217391304348
CAGCUUUGCCAAAGUCAUGCAUGAGACAGUGAAGGACCUGGCUCCGAAAUGUGAUGUGUCUUUCC
UGCAGUCAGAGGAUGGCAG
>random_seq_from_cds__NO_11314 RANDOM_LENGTH 68 SEQ_LENGTH 60
GC_CONTENT 0.766666666666667 BASEPAIR 23 FREE_ENERGY
-33.60 LEN_BP_RATIO 2.60869565217391
AGCGGCUGGAAGCGCGGGGCGGCCGGGAGGGGGCAGUCCUCGCCGGCGAGUUCAGCGGACAUCCA
GGC
>random_seq_from_cds__NO_11318 RANDOM_LENGTH 87 SEQ_LENGTH 58
GC_CONTENT 0.603448275862069 BASEPAIR 22 FREE_ENERGY
-32.70 LEN_BP_RATIO 2.63636363636364
UGGGCGGGAAAAGGUGUGAGCGGUACUGGGCCCAGGAGCAGGAGCCACUGCAGACUGGGCUUUUC
UGCAUCACUCUGGAUAAAGGAG
>random_seq_from_cds__NO_11322 RANDOM_LENGTH 79 SEQ_LENGTH 74
GC_CONTENT 0.567567567567568 BASEPAIR 25 FREE_ENERGY
-25.60 LEN_BP_RATIO 2.96
GGCAGCUGCUCCUGACCCAGGAUGAUCCCACCUGACUUCAGUCUCUUUGAUGUGGUCCUUAAGAU
GAGGAAGCAGCGGC
>random_seq_from_cds__NO_11342 RANDOM_LENGTH 93 SEQ_LENGTH 68
GC_CONTENT 0.426470588235294 BASEPAIR 27 FREE_ENERGY
-19.10 LEN_BP_RATIO 2.51851851851852
UCAAAUCCUCCCAUUUUGUUGGUCUGUUCCUUGCAUGCUAGUUUAGAGGAUCUUUAAGCAAGCGA
CAAUGAAGGCAUUGCGGAAAAAAUCUGU
>random_seq_from_cds__NO_11354 RANDOM_LENGTH 81 SEQ_LENGTH 65
GC_CONTENT 0.461538461538462 BASEPAIR 20 FREE_ENERGY
-17.10 LEN_BP_RATIO 3.25
CUUGGGAGUCUGGUUUUGAAUCUGUUGGAUCUAUACUCUGAAGUACCUGCAGGGCUCUAUCCAUC
UUAUCCCUCAUCUAUA
>random_seq_from_cds__NO_11384 RANDOM_LENGTH 83 SEQ_LENGTH 61
GC_CONTENT 0.409836065573771 BASEPAIR 21 FREE_ENERGY
-17.30 LEN_BP_RATIO 2.9047619047619
AACUGCAUUUGUUAAUAGCUGCUAUAUUAAAAGUGAGGAGGCCAAACGCCAACAACUUGGGAUAG
AGAAAGAAGCUGUUCUUC
>random_seq_from_cds__NO_11387 RANDOM_LENGTH 84 SEQ_LENGTH 71
GC_CONTENT 0.507042253521127 BASEPAIR 28 FREE_ENERGY
-24.10 LEN_BP_RATIO 2.53571428571429
ACGUGGCUAGAAACUUGGCUGUGGAGCAGUUAACACUGAGUGAAGAAUUCCCAGUGCCCCCAGCU
GUGUUACAGCAGACUUUCU
>random_seq_from_cds__NO_11394 RANDOM_LENGTH 80 SEQ_LENGTH 65
GC_CONTENT 0.738461538461539 BASEPAIR 22 FREE_ENERGY
-26.50 LEN_BP_RATIO 2.95454545454545
GUCCGGCCUUUAGAGCGCUCCCAGCGAACACAGUCCCGAGUCCUGCGGGGUGGGGGCCCCUGCCA
GCUGCCAGGCCCCUU
>random_seq_from_cds__NO_11396 RANDOM_LENGTH 80 SEQ_LENGTH 72

GC_CONTENT 0.666666666666667 BASEPAIR 27 FREE_ENERGY -36.60 LEN_BP_RATIO 2.66666666666667
UGGCAGUUCCUGGGUGUCACGGGUACCUUUAGGGGCGUGGCACUCCCCUCCCUUUGGGUGCCUCCGUUCGGGCUGUCGCC
>random_seq_from_cds__NO_11397 RANDOM_LENGTH 84 SEQ_LENGTH 75 GC_CONTENT 0.573333333333333 BASEPAIR 28 FREE_ENERGY -33.00 LEN_BP_RATIO 2.67857142857143
AGGGACCUCGACUGGGCUUGGGGGGAUCUAGCAUAGCUUUCUGGGUUUCGCCCAACCUUUGCUGAUUUGACCUGGUCCCUGGAA
>random_seq_from_cds__NO_11407 RANDOM_LENGTH 100 SEQ_LENGTH 83 GC_CONTENT 0.55421686746988 BASEPAIR 28 FREE_ENERGY -28.10 LEN_BP_RATIO 2.96428571428571
CAUGGGGAUACCUCUGCUGGGACCUGAAGCACCAGAGUGAAGCAGACAUACUGUGAAUCCUGGUCCUCUGCAGUGGCAGGAUGCAAAGUGAUGUAGAUCU
>random_seq_from_cds__NO_11421 RANDOM_LENGTH 89 SEQ_LENGTH 75 GC_CONTENT 0.466666666666667 BASEPAIR 27 FREE_ENERGY -22.80 LEN_BP_RATIO 2.77777777777778
GAUUUUCUGUAGCUGCCACUGACAUUAAUGGAGAUGAGUUAUGCAGAUGUGUUUAUUGGAGCACCUCUCUUCAUGGAUCGUGGCUCUGA
>random_seq_from_cds__NO_11441 RANDOM_LENGTH 82 SEQ_LENGTH 71 GC_CONTENT 0.492957746478873 BASEPAIR 22 FREE_ENERGY -20.12 LEN_BP_RATIO 3.22727272727273
UUGGGGUUGUGGAGUUGCUCAGUGCUUGAAGAUUGUCUGCCAAGUUGGGAGAUUAGACAGAGGAAAGAGUGCAAUCUUGUAC
>random_seq_from_cds__NO_11449 RANDOM_LENGTH 89 SEQ_LENGTH 81 GC_CONTENT 0.604938271604938 BASEPAIR 28 FREE_ENERGY -35.20 LEN_BP_RATIO 2.89285714285714
CUGAAGGGAGCCAGAUGGGGGGCUGGUUGUGCUGAGUGGAGGGGGCACCUCUGGCCGGAUGGCAUUCCUCAUGUCGGGUGUCCUUUAAU
>random_seq_from_cds__NO_11450 RANDOM_LENGTH 85 SEQ_LENGTH 60 GC_CONTENT 0.55 BASEPAIR 22 FREE_ENERGY -22.60 LEN_BP_RATIO 2.72727272727273
CAGCUGAUGAAAGGUCUGGGACAGAAACCUCUUUACACCUACCUCAUUGCAGGUGGUGACAGGGUCUGUGGUGGCCUCUAGGGAG
>random_seq_from_cds__NO_11464 RANDOM_LENGTH 69 SEQ_LENGTH 60 GC_CONTENT 0.6 BASEPAIR 21 FREE_ENERGY -18.50 LEN_BP_RATIO 2.85714285714286
GGACAGUCCAAGGCUCGAUGCAUCGAGAGCCUCCUCCGAGCGAUCCACUUUCCCCAGCCACUGUCAGAU
>random_seq_from_cds__NO_11481 RANDOM_LENGTH 81 SEQ_LENGTH 65 GC_CONTENT 0.492307692307692 BASEPAIR 21 FREE_ENERGY -19.13 LEN_BP_RATIO 3.0952380952381
AAGAAACUCAUCCUUCAUGCUCUGGAGCAGUGUGUUAAAGUCGACACCGUCUUGCUCUGAGAAAACAUCACCAAAGAGGGA

>random_seq_from_cds__NO_11515 RANDOM_LENGTH 78 SEQ_LENGTH 55 GC_CONTENT 0.418181818181818 BASEPAIR 20 FREE_ENERGY -16.50 LEN_BP_RATIO 2.75
UGUUUUCCUUCAUGAAAGGUCGCUAGAGAAGUCCGACUCUUCAUCUUUGAUGGAGUCGUAACAAG
AAUCACAAACCCG
>random_seq_from_cds__NO_11537 RANDOM_LENGTH 89 SEQ_LENGTH 69 GC_CONTENT 0.333333333333333 BASEPAIR 27 FREE_ENERGY -19.80 LEN_BP_RATIO 2.55555555555556
UUUUUUUGAUCCAGUUGAAAGUGAUGAAGACAUAACAAAUGUUCAUGAUGAUGAGCUGGAUUCAA
ACAAAGAAGAUGAUGAAAUUGCUG
>random_seq_from_cds__NO_11546 RANDOM_LENGTH 68 SEQ_LENGTH 59 GC_CONTENT 0.525423728813559 BASEPAIR 22 FREE_ENERGY -15.40 LEN_BP_RATIO 2.68181818181818
AAAGUUGUGUCAAAUCUGCCAGCCAUAACCAUGGAGGAAGUAGCCCCAGUGAGUGUUAGUGAUGC
AGC
>random_seq_from_cds__NO_11560 RANDOM_LENGTH 88 SEQ_LENGTH 67 GC_CONTENT 0.567164179104478 BASEPAIR 22 FREE_ENERGY -23.36 LEN_BP_RATIO 3.04545454545455
AACAUGCCCCACAAGUUCGGUAUCCACAACUACAAGGUCCCUACCUUCUGCGAUCACUGUGGGUC
CCUGCUCUGGGGACUCUUGCGGC
>random_seq_from_cds__NO_11565 RANDOM_LENGTH 110 SEQ_LENGTH 104 GC_CONTENT 0.490384615384615 BASEPAIR 35 FREE_ENERGY -33.80 LEN_BP_RATIO 2.97142857142857
UGGGCCUGGAUGAGUUCAACUUCAUCAAGGUGUUGGGCAAAGGCAGCUUUGGCAAGGGUCAUGUU
GGCAGAACUCAAGGGCAAAGAUGAAGUAUAUGCUGUGAAGGUCUU
>random_seq_from_cds__NO_11577 RANDOM_LENGTH 97 SEQ_LENGTH 81 GC_CONTENT 0.481481481481481 BASEPAIR 28 FREE_ENERGY -25.80 LEN_BP_RATIO 2.89285714285714
CGAUGAAGGCCAGGGAUAAAUUUCAAGAGUUGCUUUCGGACACUUCCCUUUCUGCUCUUCCGGGG
CAGGGUCCCAAACAUGCUUGAUAAGUUGCCCU
>random_seq_from_cds__NO_11585 RANDOM_LENGTH 110 SEQ_LENGTH 91 GC_CONTENT 0.54945054945055 BASEPAIR 29 FREE_ENERGY -36.82 LEN_BP_RATIO 3.13793103448276
GCUUGCAGGCCAGCACAGUGAGCUGGGCUGUCCUCCUGUAUUUUGCAUAUCAAAGUGAACAUUUC
CGAGGAGCAGGCAUUCUGAUUCUGGGGCCUGUAAGACCUGAAUUU
>random_seq_from_cds__NO_11595 RANDOM_LENGTH 78 SEQ_LENGTH 69 GC_CONTENT 0.521739130434783 BASEPAIR 27 FREE_ENERGY -19.80 LEN_BP_RATIO 2.55555555555556
UCUUCAUGGUCAUGAUGUCCCCAUAGCAGCCGUGGUACAGGCGUGGAUGUAGGUGAGGCUUCAUU
GAGUGACUGAAGG
>random_seq_from_cds__NO_11607 RANDOM_LENGTH 90 SEQ_LENGTH 47 GC_CONTENT 0.595744680851064 BASEPAIR 20 FREE_ENERGY -27.50 LEN_BP_RATIO 2.35
UCCCUAUGAUCCAACACCAUAUUGAAGCCUGUGCCCAGGGUCCCUGGAAUGCAGGAGAUUCUGCU

CAUUGGCUUCUACCAACCUGAUGAG
>random_seq_from_cds__NO_11610 RANDOM_LENGTH 88 SEQ_LENGTH 80 GC_CONTENT 0.575 BASEPAIR 23 FREE_ENERGY -22.90 LEN_BP_RATIO 3.47826086956522
UGCUCCGACUUCCCCUUGAGUGCUAUGUUGGAAGCCCACCGACGCCAGCGUCACCCUUUCUUACUCCUUGGCACUACGGGCUAACAGG
>random_seq_from_cds__NO_11613 RANDOM_LENGTH 72 SEQ_LENGTH 61 GC_CONTENT 0.655737704918033 BASEPAIR 19 FREE_ENERGY -22.10 LEN_BP_RATIO 3.21052631578947
AAUCAGCAGGAUGGGCAAUUGGGAGGACUCACCAGGCUUGUGGCCAGGGGCAGGUACCAUCCGCCUAGAGCA
>random_seq_from_cds__NO_11616 RANDOM_LENGTH 79 SEQ_LENGTH 71 GC_CONTENT 0.676056338028169 BASEPAIR 26 FREE_ENERGY -31.30 LEN_BP_RATIO 2.73076923076923
UCCGAGGGGAAUGUGUACAUCCACCCGACCGCCAAGGUGGCCCCCUCGGCUGUGGCUGGGCCCCAACGUCUCCAUCGGG
>random_seq_from_cds__NO_11637 RANDOM_LENGTH 110 SEQ_LENGTH 91 GC_CONTENT 0.373626373626374 BASEPAIR 34 FREE_ENERGY -27.00 LEN_BP_RATIO 2.67647058823529
ACAUACGCAGGGUAAGUGGUAUCGAUAAUAAAUAUCCGAACAACGGGAUUCUUAGCUCCAGCCCUUUGGGUAUGGAAUAUUUAUUGUUCUAGGAUAUUGUUCAUCGCCAU
>random_seq_from_cds__NO_11639 RANDOM_LENGTH 94 SEQ_LENGTH 74 GC_CONTENT 0.310810810810811 BASEPAIR 26 FREE_ENERGY -16.00 LEN_BP_RATIO 2.84615384615385
UUCAUAAACCCAGUCUGGGAUUCCAUUAAAUAUUUAUUUUCUCUUCCAUUAAAUGUUAUUUGAAAAGGUGGAUCUCCUGGUCUUUGUUUCAAA
>random_seq_from_cds__NO_11642 RANDOM_LENGTH 95 SEQ_LENGTH 83 GC_CONTENT 0.349397590361446 BASEPAIR 28 FREE_ENERGY -17.90 LEN_BP_RATIO 2.96428571428571
CUAGAUAUACAAAUUGCCGAUCAGGUGAUAAGCCGUAAUUUGAAGCAUUCACACUUUUCCAUGGUUCUAUUACUCAAAAUGGUAUAUGAUUGUCC
>random_seq_from_cds__NO_11648 RANDOM_LENGTH 82 SEQ_LENGTH 71 GC_CONTENT 0.47887323943662 BASEPAIR 22 FREE_ENERGY -17.60 LEN_BP_RATIO 3.22727272727273
GACGAGGAAUGCCUCCCGACACUGCUGGUAAAAAUCUUGAAAAGAAAGGCCCAAUGUAAGAAGGGUUAUCCUGGUUGUCCAG
>random_seq_from_cds__NO_11659 RANDOM_LENGTH 81 SEQ_LENGTH 60 GC_CONTENT 0.4 BASEPAIR 21 FREE_ENERGY -16.00 LEN_BP_RATIO 2.85714285714286
UUUUAUCUCUUUUCAUUCUCUUAGAAGGUGUUUCUUUGCCAACAGGAGCUGAAACUACUCUAUUCUGAGCUUGAAUCUUUU
>random_seq_from_cds__NO_11667 RANDOM_LENGTH 84 SEQ_LENGTH 63 GC_CONTENT 0.555555555555556 BASEPAIR 23 FREE_ENERGY -26.81 LEN_BP_RATIO 2.73913043478261

GGUUUACUCAUGAUGGCGGCCUUCUCCGGAGAUGGGUGUAAUGCCUGAGAUUGCACAAGCUGUGG
AAGAGAUGGAUUGGCUGCC
>random_seq_from_cds__NO_11679 RANDOM_LENGTH 88 SEQ_LENGTH 62 GC_CONTENT 0.483870967741936 BASEPAIR 22 FREE_ENERGY -18.70 LEN_BP_RATIO 2.81818181818182
UAAGAAAUACAUUGAUAAUCCUAAAUUAAGGGGAGCUUCUGAUAAUUGGAGGUGUUGCAGCCCGG
GAUCAGCUCUCUGUUUUGGAAAA
>random_seq_from_cds__NO_11681 RANDOM_LENGTH 84 SEQ_LENGTH 57 GC_CONTENT 0.403508771929825 BASEPAIR 20 FREE_ENERGY -17.20 LEN_BP_RATIO 2.85
AUCUCAAGUUAGAUUCCUGGUCCUGGAUGAAGCUGGAUGGGCUUCUUUCUCAAGGUUAUUCUGAU
UUUAUAAAUAGGAUGCACA
>random_seq_from_cds__NO_11697 RANDOM_LENGTH 110 SEQ_LENGTH 96 GC_CONTENT 0.604166666666667 BASEPAIR 33 FREE_ENERGY -40.50 LEN_BP_RATIO 2.90909090909091
CAAGCGUGAGGCCCUGUAGCGGGGUCAGGGAAGCCAUUGCAUCGGACCACAGAGGCGUAGAACUU
CGCUGAUGCUUUGGGGAUCCUUGGCAGAGAAGGGUCACUGUAGUU
>random_seq_from_cds__NO_11702 RANDOM_LENGTH 99 SEQ_LENGTH 79 GC_CONTENT 0.481012658227848 BASEPAIR 29 FREE_ENERGY -27.00 LEN_BP_RATIO 2.72413793103448
AUAGUUGAGGUUGUAGGCGAGGACAGUGGUGUAGUGAUUGAACCCAAAAAAGUCAUAGGUGCCGU
UGAUCCUCCUCUUCUCACUCUCUGUAAAUUCUGG
>random_seq_from_cds__NO_11713 RANDOM_LENGTH 90 SEQ_LENGTH 75 GC_CONTENT 0.573333333333333 BASEPAIR 23 FREE_ENERGY -24.57 LEN_BP_RATIO 3.26086956521739
UCAGGAAACCGUCCGUACAGAAACUCAUCCUCCCUGGCCAGUGGCAUGCCGUUGUUGGUAAUGAC
CUCUGUGUAGUACCUGGCGGAGGCU
>random_seq_from_cds__NO_11716 RANDOM_LENGTH 88 SEQ_LENGTH 66 GC_CONTENT 0.46969696969697 BASEPAIR 21 FREE_ENERGY -23.30 LEN_BP_RATIO 3.14285714285714
GGUUUUGUGGUAAAAUAUCCUAUCAGUAUCCUCCGUGUUCGGAUUGGUCAGCCCCACUCCGUUUU
CGGUGAUGUAAAUGGGGAUGUCA
>random_seq_from_cds__NO_11721 RANDOM_LENGTH 84 SEQ_LENGTH 71 GC_CONTENT 0.619718309859155 BASEPAIR 26 FREE_ENERGY -29.00 LEN_BP_RATIO 2.73076923076923
CAUUCGGUCAGCGGCUUCCACAUCUCUGGGGACCCCUGGUGACUUGGGCUCUGCCCAGUGUGUAC
UGAGGCUCAGCGAGAUGAC
>random_seq_from_cds__NO_11731 RANDOM_LENGTH 80 SEQ_LENGTH 60 GC_CONTENT 0.5 BASEPAIR 22 FREE_ENERGY -20.10 LEN_BP_RATIO 2.72727272727273
AAAGUGAAGGCUCUGACUUUGGAGGGGAGGUUUACUGUAUUAGGUGGUAGCAGUCUUUUUGCCCC
CUUGGUGAGGAAACC
>random_seq_from_cds__NO_11732 RANDOM_LENGTH 78 SEQ_LENGTH 58 GC_CONTENT 0.448275862068966 BASEPAIR 22 FREE_ENERGY

-17.50 LEN_BP_RATIO 2.63636363636364
GUUCUUUUCUAUGAUGCUAGUGAAAAAGUAGGCAGAUUUCCUGGGAGUCCUUGACUUGCUGCUGU
CGCUGAAGUUGAC
>random_seq_from_cds__NO_11742 RANDOM_LENGTH 71 SEQ_LENGTH 63
GC_CONTENT 0.650793650793651 BASEPAIR 22 FREE_ENERGY
-25.80 LEN_BP_RATIO 2.86363636363636
GGACCAAGUGAGCCACCCUUAAAAGAGGCCACUCCUGGGUCAGAGAUGCCGGGAGGGUGCUGGCC
GGUGCC
>random_seq_from_cds__NO_11744 RANDOM_LENGTH 74 SEQ_LENGTH 56
GC_CONTENT 0.642857142857143 BASEPAIR 20 FREE_ENERGY
-24.50 LEN_BP_RATIO 2.8
CGCUCUCAUUCUGCCAUCCACCAUGAUCCUGCAGGGCCUGAGGCAGGUCCCAGUGGAACAGCGUG
GCCAUGGGC
>random_seq_from_cds__NO_11757 RANDOM_LENGTH 97 SEQ_LENGTH 84
GC_CONTENT 0.571428571428571 BASEPAIR 34 FREE_ENERGY
-29.10 LEN_BP_RATIO 2.47058823529412
UGGUGCAGGAUGACCAUGGGCUGAAGCCGUGCAGUCUUGAGGGCCUUGAGGAGUCGCCGGUAGCA
CUGCACUGUUUUCUCGUCUGGAUUCUGGGUGC
>random_seq_from_cds__NO_11758 RANDOM_LENGTH 89 SEQ_LENGTH 81
GC_CONTENT 0.555555555555556 BASEPAIR 27 FREE_ENERGY
-31.90 LEN_BP_RATIO 3
UUCCUGCUGGGAGGAGCUGUGCCCAUGACAGAAAUACCUUAUAAUGGGUGAUCUGACUGGCAUGG
AGACUGCUGAAGUAUUCUGGCAGG
>random_seq_from_cds__NO_11767 RANDOM_LENGTH 71 SEQ_LENGTH 54
GC_CONTENT 0.351851851851852 BASEPAIR 21 FREE_ENERGY
-16.90 LEN_BP_RATIO 2.57142857142857
UGUUGUAGUCUGCAUUUUGGGAUGCAUACUUAUUUGUUGGCUUACAAAAAAGGAAGUAUUCAUCC
AGUGUG
>random_seq_from_cds__NO_11773 RANDOM_LENGTH 92 SEQ_LENGTH 80
GC_CONTENT 0.625 BASEPAIR 27 FREE_ENERGY -37.00
LEN_BP_RATIO 2.96296296296296
GCGGGAACAGGGGUCUCUGCUGGGGAAGCUGGUCCAGAAUGGAACAGAACCUUCAUCCCUGCCAU
UCUUGGACCCCAAUGCCCGCCCCCUGG
>random_seq_from_cds__NO_11774 RANDOM_LENGTH 85 SEQ_LENGTH 68
GC_CONTENT 0.588235294117647 BASEPAIR 27 FREE_ENERGY
-33.70 LEN_BP_RATIO 2.51851851851852
UACCAGAGGUCUCCAUUAAGGACUCCACGGGUAUUCAAUACAGGGGGUGCCCCUCGGAUCCUUGC
UUUGGACUGUGGCCUCAAGU
>random_seq_from_cds__NO_11775 RANDOM_LENGTH 82 SEQ_LENGTH 67
GC_CONTENT 0.626865671641791 BASEPAIR 22 FREE_ENERGY
-24.90 LEN_BP_RATIO 3.04545454545455
AUAAUCAGAUCCGAUGCCUCUGCCAGCGUGGGGCUGAGGUCACUGUGGUACCCUGGGACCAUGCA
CUAGACAGCCAAGGAGU
>random_seq_from_cds__NO_11783 RANDOM_LENGTH 71 SEQ_LENGTH 60

GC_CONTENT 0.583333333333333 BASEPAIR 19 FREE_ENERGY -19.80 LEN_BP_RATIO 3.15789473684211
AGACGUUGCUGAUCAACCCCAAUAUUGCCACAGUGCAGACCUCCCAGGGGCUGGCCGACAAGGUCUAUUUU
>random_seq_from_cds__NO_11796 RANDOM_LENGTH 87 SEQ_LENGTH 76 GC_CONTENT 0.578947368421053 BASEPAIR 24 FREE_ENERGY -17.00 LEN_BP_RATIO 3.16666666666667
UGGACCGCCUGUAUGAGCUCACACGCAUCGACCGCUGGUUCCUGCACCGAAUGAAGCGUAUCAUCGCACAUGCCCAGCUGCUAGAAC
>random_seq_from_cds__NO_11806 RANDOM_LENGTH 84 SEQ_LENGTH 68 GC_CONTENT 0.632352941176471 BASEPAIR 21 FREE_ENERGY -27.12 LEN_BP_RATIO 3.23809523809524
CCCUCCUAUGUGCUGAGCGGUGCUGCUAUGAAUGUGGCCUACACGGAUGGAGACCUGGAGCGCUUCCUGAGCAGCGCAGCAGCC
>random_seq_from_cds__NO_11811 RANDOM_LENGTH 85 SEQ_LENGTH 68 GC_CONTENT 0.573529411764706 BASEPAIR 28 FREE_ENERGY -28.70 LEN_BP_RATIO 2.42857142857143
GGGUGUGGACCUAGUAGCCUUGGCCACGCGGGUCAUCAUGGGGGAAGAAGUGGAACCUGUGGGGCUAAUGACUGGUUCUGGAGUC
>random_seq_from_cds__NO_11813 RANDOM_LENGTH 81 SEQ_LENGTH 62 GC_CONTENT 0.564516129032258 BASEPAIR 24 FREE_ENERGY -29.60 LEN_BP_RATIO 2.58333333333333
GAGGUGGCCGGCUUUGGGGAGAGCCGCUGUGAGGCAUACCUCAAGGCCAUGCUAAGCACUGGCUUUAAGAUCCCCAAGAAG
>random_seq_from_cds__NO_11820 RANDOM_LENGTH 90 SEQ_LENGTH 70 GC_CONTENT 0.7 BASEPAIR 25 FREE_ENERGY -30.82 LEN_BP_RATIO 2.8
GCCCUGGCUGGGGGUAUCACCAUGGUGUGUGCCAUGCCUAAUACCCGGCCCCCCAUCAUUGACGCCCCUGCUCUGGCCCUGGCCCAGAAG
>random_seq_from_cds__NO_11829 RANDOM_LENGTH 72 SEQ_LENGTH 64 GC_CONTENT 0.609375 BASEPAIR 21 FREE_ENERGY -22.70 LEN_BP_RATIO 3.04761904761905
CCUAUGUGGAGGGUGGAUCUGGAGCAUGAGUGGACAAUUCCCAGCCACAUGCCCUUCUCCAAGGCCCACUGG
>random_seq_from_cds__NO_11834 RANDOM_LENGTH 84 SEQ_LENGTH 74 GC_CONTENT 0.621621621621622 BASEPAIR 21 FREE_ENERGY -21.33 LEN_BP_RATIO 3.52380952380952
AGGUAGCCGAGCCAGGAGCUGAUGGGAACCCCUGAUGGCACCUGCUACCCUCCACCACCAGUACCGAGACAGGCAUCUCCCCAG
>random_seq_from_cds__NO_11835 RANDOM_LENGTH 84 SEQ_LENGTH 69 GC_CONTENT 0.623188405797101 BASEPAIR 19 FREE_ENERGY -19.22 LEN_BP_RATIO 3.63157894736842
AACCUGGGGACCCCUGGCUUGCUGCACCCCCAGACCUCACCCCUGCUGCACUCAUUAGUGGGCCAACAUAUCCUGUCCGUCCAG

>random_seq_from_cds__NO_11838 RANDOM_LENGTH 72 SEQ_LENGTH 63 GC_CONTENT 0.619047619047619 BASEPAIR 22 FREE_ENERGY -23.30 LEN_BP_RATIO 2.86363636363636
UGUGCUCAGCUUCUCGGAAGCCACAUCGUCCGUCCAGAAGGGCGAAUCCCUGGCUGACUCCGUGC
AGACCAU
>random_seq_from_cds__NO_11839 RANDOM_LENGTH 74 SEQ_LENGTH 60 GC_CONTENT 0.716666666666667 BASEPAIR 21 FREE_ENERGY -28.60 LEN_BP_RATIO 2.85714285714286
GAGCUGCUAUGCCGACGUCGUCGUGCUCCGGCACCCCCAGCCUGGAGCAGUGGAGGCUGGCCGCC
AAGCACUGC
>random_seq_from_cds__NO_11847 RANDOM_LENGTH 84 SEQ_LENGTH 70 GC_CONTENT 0.6 BASEPAIR 22 FREE_ENERGY -31.84 LEN_BP_RATIO 3.18181818181818
GGAAGCAGGAGGAGAAGCACCUGAAGAUGCUGCGGGACAUGACCGGCCUCCCGCACAACCGAAAG
UGCUUCGACUGCGACCAGC
>random_seq_from_cds__NO_11889 RANDOM_LENGTH 78 SEQ_LENGTH 65 GC_CONTENT 0.538461538461538 BASEPAIR 23 FREE_ENERGY -20.40 LEN_BP_RATIO 2.82608695652174
GUCACCGAGGACGAGGUCGAGUGGAUGAGACAAGGCUGGACUACUUACUACUGAUCCACGUGUGA
GGGAACGGAAGAA
>random_seq_from_cds__NO_11890 RANDOM_LENGTH 83 SEQ_LENGTH 66 GC_CONTENT 0.575757575757576 BASEPAIR 24 FREE_ENERGY -29.70 LEN_BP_RATIO 2.75
GCCAGGCCAAGAGGGAGCCCGCAGAAAGUUUACGUGGAAGAAACGCUAAGUUAGACUGUGGCGCU
CAGCAUGGCCUCCGUCUC
>random_seq_from_cds__NO_11897 RANDOM_LENGTH 86 SEQ_LENGTH 77 GC_CONTENT 0.662337662337662 BASEPAIR 29 FREE_ENERGY -33.90 LEN_BP_RATIO 2.6551724137931
GCAGCUUCUCCAGCACAGUGGGGUGCUUCAGCAGCUGCAUGAUGAGUGAGGUGCUGGCGCUGGCC
GUGGUGGCAUAGGCCGCAAAG
>random_seq_from_cds__NO_11902 RANDOM_LENGTH 71 SEQ_LENGTH 55 GC_CONTENT 0.690909090909091 BASEPAIR 23 FREE_ENERGY -28.90 LEN_BP_RATIO 2.39130434782609
GGCCUCGGGGUGGCUGCUCCAGGCGCGCAGUGUGUCCUGGAUCACCAGCUGGAUCUUGGGCAGGU
AACUCU
>random_seq_from_cds__NO_11906 RANDOM_LENGTH 89 SEQ_LENGTH 76 GC_CONTENT 0.684210526315789 BASEPAIR 28 FREE_ENERGY -38.60 LEN_BP_RATIO 2.71428571428571
UGGCCGGUCUCUCCGAUGAGCGGGAAGCCCAUGGAUCCCUUGGGGAUGGGCAGCUUGCAGCUCUU
GUCGCGAGUGGCGGCCCAGCGCAG
>random_seq_from_cds__NO_11911 RANDOM_LENGTH 85 SEQ_LENGTH 66 GC_CONTENT 0.454545454545455 BASEPAIR 23 FREE_ENERGY -20.10 LEN_BP_RATIO 2.8695652173913
AUGAUAGCCAGAUUUCUUCUGCAUGGCGGUUACAGGGCAAUCUUUAUGAGCCAGAAGAAGCUGUU

UCAGCUGUGCCACUUCAUUU
>random_seq_from_cds__NO_11914 RANDOM_LENGTH 72 SEQ_LENGTH 50 GC_CONTENT 0.58 BASEPAIR 19 FREE_ENERGY -19.40 LEN_BP_RATIO 2.63157894736842
GUUAGCUGCUCUUCUCCGACGACCACUUGUACUUUGGGUCUGUGGAGUUGUGUGAGCUGGAGAAG
CCCGGAG
>random_seq_from_cds__NO_11915 RANDOM_LENGTH 97 SEQ_LENGTH 86 GC_CONTENT 0.523255813953488 BASEPAIR 31 FREE_ENERGY -30.00 LEN_BP_RATIO 2.7741935483871
UUUCUGUAGUGGAUGUGGCUGGCUGUUGUAAUGACUGCGGUCGAGAUUCCUCUGACUGAGUCCUA
ACCAAUCCGCUACCAUGACCUUUGACAGUAUC
>random_seq_from_cds__NO_11916 RANDOM_LENGTH 71 SEQ_LENGTH 54 GC_CONTENT 0.407407407407407 BASEPAIR 19 FREE_ENERGY -17.60 LEN_BP_RATIO 2.84210526315789
ACCAUUGGUAACUGGAGGAUGUUGCUGGGUCAAAGCAGCUUUUAAUCUCCAUUUUUGCUUCUGAC
UGUACU
>random_seq_from_cds__NO_11917 RANDOM_LENGTH 68 SEQ_LENGTH 63 GC_CONTENT 0.603174603174603 BASEPAIR 20 FREE_ENERGY -18.20 LEN_BP_RATIO 3.15
GGUUGGGGAGAGGAAGGACCUGGGAUUCCUGGAACACUAGGCACCAUGGUGACUGGUCGAACGAG
CUG
>random_seq_from_cds__NO_11924 RANDOM_LENGTH 82 SEQ_LENGTH 71 GC_CONTENT 0.47887323943662 BASEPAIR 26 FREE_ENERGY -26.50 LEN_BP_RATIO 2.73076923076923
AAUCUUGUUGGUGUUGGGGUCUGAUCCAGCCACAAUGACACUGUCAUUACGUGCUGGACCAAAUU
UCAGUGUCAUCUCAUGU
>random_seq_from_cds__NO_11937 RANDOM_LENGTH 69 SEQ_LENGTH 52 GC_CONTENT 0.538461538461538 BASEPAIR 19 FREE_ENERGY -16.00 LEN_BP_RATIO 2.73684210526316
AAGCACAGAAACUUGGUAAUGGGAUCAACAUCAUUGUGGCCACACCAGGCCGUCUGCUGGACCAU
AUGC
>random_seq_from_cds__NO_11940 RANDOM_LENGTH 110 SEQ_LENGTH 87 GC_CONTENT 0.448275862068966 BASEPAIR 31 FREE_ENERGY -28.30 LEN_BP_RATIO 2.80645161290323
UUGAAGACCUGGCAAGGAUUUCUCUGAAAAAGGAGCCAUUGUAUGUUGGCGUUGAUGAUGAUAAA
GCGAAUGCAACAGUGGAUGGUCUUGAACAGGGGAUAUGUUGUUUG
>random_seq_from_cds__NO_11950 RANDOM_LENGTH 90 SEQ_LENGTH 75 GC_CONTENT 0.48 BASEPAIR 27 FREE_ENERGY -27.50 LEN_BP_RATIO 2.77777777777778
AUCAUUCCAGGCCUGAAGUGAGGGAGCCUUUAUAUUGCCACAGUGUUCAUUGUUCCCAGCAUUGU
UGGGCUCACCUGGAAUCCAGAACCC
>random_seq_from_cds__NO_11956 RANDOM_LENGTH 99 SEQ_LENGTH 69 GC_CONTENT 0.536231884057971 BASEPAIR 23 FREE_ENERGY -22.10 LEN_BP_RATIO 3

CCCAACUUCUUGUUAAGAUCUGGAUCUGUGCGUUGGCCUUCUCCACACUGGUUUUAACUUCAGG
AACUGAGAACGCACAUAACCCAGGCUCUCAUUCA
>random_seq_from_cds__NO_11964 RANDOM_LENGTH 87 SEQ_LENGTH 54
GC_CONTENT 0.407407407407407 BASEPAIR 20 FREE_ENERGY
-16.70 LEN_BP_RATIO 2.7
GAAAAGCUAUCCUCACAUCAUUCAGGACAAAUGUACCCUUCACUUUUUGACCCCAGGGGUGAAUA
AUUCUGGGUCAUAUAUUUGUAG
>random_seq_from_cds__NO_11983 RANDOM_LENGTH 89 SEQ_LENGTH 80
GC_CONTENT 0.5625 BASEPAIR 25 FREE_ENERGY -15.90
LEN_BP_RATIO 3.2
CGGACCUCUUCACCACGCUGGUGGACCUCAAGUGGCGCUGGAACCUCUUCAUCUUCAUUCUCACC
UACACCGUGGCCUGGCUUUUCAUG
>random_seq_from_cds__NO_11997 RANDOM_LENGTH 89 SEQ_LENGTH 74
GC_CONTENT 0.513513513513513 BASEPAIR 25 FREE_ENERGY
-23.90 LEN_BP_RATIO 2.96
CCACCAAGAUGUUAUCUGAUCCCAUGAGCCAGUCUGUGGCUGAUUUGCCACCAAAGCUUCAAAAG
AUGGCUGGAGGAGCAGCUAGGAUG
>random_seq_from_cds__NO_12001 RANDOM_LENGTH 101 SEQ_LENGTH 82
GC_CONTENT 0.463414634146341 BASEPAIR 25 FREE_ENERGY
-23.44 LEN_BP_RATIO 3.28
GUUAACAAGCUCGCUCCUCUUUAAUGCUGACUCGUCCUCUUCCUUCUUCCACCUUUCUGAGGUGA
AGCACAAUAAGGUUAGAGAUUCGGCAGUACUCAGAG
>random_seq_from_cds__NO_12009 RANDOM_LENGTH 95 SEQ_LENGTH 80
GC_CONTENT 0.4 BASEPAIR 29 FREE_ENERGY -21.40 LEN_BP_RATIO
2.75862068965517
UCGGGACAUGAUGGGAGCUGACAAAUUUAUAUUCUGUUUCAAUGAUUUUGAUCUGUCAUAGUGUC
CACUGAUUGGGUUUGCUGCUGCCAAAAUGG
>random_seq_from_cds__NO_12018 RANDOM_LENGTH 88 SEQ_LENGTH 70
GC_CONTENT 0.528571428571429 BASEPAIR 21 FREE_ENERGY
-22.15 LEN_BP_RATIO 3.33333333333333
UCGAAUGCCUUCUGUCUCAUAUCCAUCAACACCACUGACACGGGAAUUAGUUUCUGCACGUGCUC
CCUGGUGUGCUAAGCUUGGAGAC
>random_seq_from_cds__NO_12027 RANDOM_LENGTH 95 SEQ_LENGTH 76
GC_CONTENT 0.763157894736842 BASEPAIR 29 FREE_ENERGY
-40.00 LEN_BP_RATIO 2.62068965517241
UUCUGGCACUUCUCGGCCACCUCGUCGCGGACCUCCAGGUGCUGGCUGCCGGCGCCCGGCUCCGC
UGCCGCCGCGAGGUCCAUUUCAGCAUAUGG
>random_seq_from_cds__NO_12047 RANDOM_LENGTH 84 SEQ_LENGTH 55
GC_CONTENT 0.509090909090909 BASEPAIR 19 FREE_ENERGY
-19.80 LEN_BP_RATIO 2.89473684210526
UAUCAUUUCCAAACAUCAUGGACUGGUCCCUGCAGAUCACAUUCACCUCCCUGGUCACAAAUAGG
ACAGUCCAAUGGGUGAUUU
>random_seq_from_cds__NO_12061 RANDOM_LENGTH 84 SEQ_LENGTH 79
GC_CONTENT 0.556962025316456 BASEPAIR 27 FREE_ENERGY

-26.20 LEN_BP_RATIO 2.92592592592593
GGUGGCGAGGGGCUUCCCAUCUCCUGUGGUACUGUCUGGAUAAUGAAUCCCCGCUUCAGGAAAGU
GCUUGAAGCCCUAGUUUCU
>random_seq_from_cds__NO_12073 RANDOM_LENGTH 70 SEQ_LENGTH 61
GC_CONTENT 0.672131147540984 BASEPAIR 22 FREE_ENERGY
-25.60 LEN_BP_RATIO 2.77272727272727
ACGUGGCGCGUCAGCUGCACAUCUCCCUGCAGUCUGAGCCUGUGGUGACCGAUGCGUUCCUGGCC
GUGGC
>random_seq_from_cds__NO_12075 RANDOM_LENGTH 85 SEQ_LENGTH 73
GC_CONTENT 0.712328767123288 BASEPAIR 28 FREE_ENERGY
-36.60 LEN_BP_RATIO 2.60714285714286
CAGGCCCAGCCUGCCAUGGUCCACGCCCUCGUGGACUGCCUGGGGGAGUUCGUGCGCAAGACCCU
GGCAACCUGGCUGCGGAGAC
>random_seq_from_cds__NO_12096 RANDOM_LENGTH 87 SEQ_LENGTH 71
GC_CONTENT 0.647887323943662 BASEPAIR 25 FREE_ENERGY
-32.60 LEN_BP_RATIO 2.84
CUUCCUGCUGGUGUGGCUGGUGGUCUUCUGUUGCAGGAUUAUGGCCCUGGCCGCCGCGGCCCUGC
UCCCCACCUUCCACAUGGCCUC
>random_seq_from_cds__NO_12112 RANDOM_LENGTH 84 SEQ_LENGTH 67
GC_CONTENT 0.462686567164179 BASEPAIR 26 FREE_ENERGY
-22.90 LEN_BP_RATIO 2.57692307692308
ACGUCCUCUCUACAAGGAAUAUUGUCCUCUCUGGAGGUUCAACCAUGUUCAGGGACUUUGGACGU
CGCUUGCAAAGAGAUUUGA
>random_seq_from_cds__NO_12137 RANDOM_LENGTH 92 SEQ_LENGTH 80
GC_CONTENT 0.35 BASEPAIR 29 FREE_ENERGY -19.00
LEN_BP_RATIO 2.75862068965517
CUUAGUGCAGAGAAGAAAGCUGGGGGCUGGUCAAGGUUUGCAAAUAGGUAUAUUUUAACCAAUA
CUGAUCACUUUUAUUCCAUUCUUCUUU
>random_seq_from_cds__NO_12146 RANDOM_LENGTH 92 SEQ_LENGTH 88
GC_CONTENT 0.840909090909091 BASEPAIR 35 FREE_ENERGY
-52.90 LEN_BP_RATIO 2.51428571428571
CGGAGCCGCCUUGGCCGGCCCGGGCCCGGGGCUGCGCGCCGCCGGCUUCAGCCGCAGCUUCAGCU
CGGACUCGGGCUCCAGCCCGGCGUCCG
>random_seq_from_cds__NO_12181 RANDOM_LENGTH 81 SEQ_LENGTH 74
GC_CONTENT 0.486486486486487 BASEPAIR 30 FREE_ENERGY
-26.80 LEN_BP_RATIO 2.46666666666667
CUGCUUUCCCACUCAUCCUUCACAUGGAGAGUCUUGGAAGCCAUUUCUAAUAGAUGUUGAUGGUG
GAGGUGGGGGAGGUGC
>random_seq_from_cds__NO_12184 RANDOM_LENGTH 82 SEQ_LENGTH 74
GC_CONTENT 0.648648648648649 BASEPAIR 22 FREE_ENERGY
-23.70 LEN_BP_RATIO 3.36363636363636
CGGGGGAGGAAGAGGACCUGAACGUCCUGGCGAAGGUAACGGGGGCGUGGACGAACUGAGGGACA
GAUUCCGCUGUGGGAGU
>random_seq_from_cds__NO_12186 RANDOM_LENGTH 81 SEQ_LENGTH 67

GC_CONTENT 0.641791044776119 BASEPAIR 23 FREE_ENERGY -31.70 LEN_BP_RATIO 2.91304347826087
GGAAGGCACUGGAGGCUUGUUGUUCUGAGGAGGAGGAGGGGGAACCGCUUCCCUGUGGAUGGAGGGCCUGUUGCCCACUGG
>random_seq_from_cds__NO_12189 RANDOM_LENGTH 110 SEQ_LENGTH 102 GC_CONTENT 0.607843137254902 BASEPAIR 36 FREE_ENERGY -42.40 LEN_BP_RATIO 2.83333333333333
GGCUGCCUGGGGCCUCCGGGCACUGGUGGGGACCCCGGUUGUGCGGACUUGAUUGAAUGGGUCUUGGAGUACUAGGUACUGGAGGAGGAAUGCUAUCAGGCUUUGAGCC
>random_seq_from_cds__NO_12191 RANDOM_LENGTH 110 SEQ_LENGTH 103 GC_CONTENT 0.58252427184466 BASEPAIR 35 FREE_ENERGY -38.80 LEN_BP_RATIO 2.94285714285714
CCUGGGCCACUUGGGGGUGAAAAGGGUUUCGCAGAUGUGGAUCUUCCUCCCGGUGGCAACAAUGGUGGUCGGCUUCCUCCAGAAUCCAUUAUCCCUGUUGGCCGUGGAUC
>random_seq_from_cds__NO_12196 RANDOM_LENGTH 87 SEQ_LENGTH 79 GC_CONTENT 0.683544303797468 BASEPAIR 29 FREE_ENERGY -39.30 LEN_BP_RATIO 2.72413793103448
GCUGGGGGCGUCGCUCCACGUCUGGGGUUGGCUAAUGCUCGGCAGCUGCCUCCUGGCCAGAGCCCAGGCUGGAUUCUGAUGGCACCA
>random_seq_from_cds__NO_12200 RANDOM_LENGTH 95 SEQ_LENGTH 83 GC_CONTENT 0.433734939759036 BASEPAIR 27 FREE_ENERGY -18.50 LEN_BP_RATIO 3.07407407407407
ACAUGGGCCAAUUAUUCAGACUGCCUUCGCUUUCUGCAGCCAGAUAUCAGCAUAGGAAAGGCAAGAAUUCUUUGAACGCCUCUAUGUAAUGUAUA
>random_seq_from_cds__NO_12204 RANDOM_LENGTH 110 SEQ_LENGTH 103 GC_CONTENT 0.378640776699029 BASEPAIR 28 FREE_ENERGY -19.51 LEN_BP_RATIO 3.67857142857143
GUUGUGAUGUUUAUUUACUUCCUGGCUACAAAUUAUUAUUGGAUCCUGGUGGAAGGUCUCUACCUGCAUAAUCUCAUCUUUGUGGCUUUCUUUUCGGACACCAAAUACCU
>random_seq_from_cds__NO_12208 RANDOM_LENGTH 73 SEQ_LENGTH 66 GC_CONTENT 0.575757575757576 BASEPAIR 23 FREE_ENERGY -24.90 LEN_BP_RATIO 2.8695652173913
GUCCUGGUCCUAGUCUUUGGAGUGCAUUACAUCGUGUUCGUAUGCCUGCCUCACUCCUUCACUGGGCUCGGGU
>random_seq_from_cds__NO_12227 RANDOM_LENGTH 68 SEQ_LENGTH 54 GC_CONTENT 0.592592592592593 BASEPAIR 20 FREE_ENERGY -19.50 LEN_BP_RATIO 2.7
CAUGGUGGCCCGUGUCUCUGUGUCAUGGGAGUGCAGCAAGGAGGCACUCAAGCGCCCAUUAAUCAGCU
>random_seq_from_cds__NO_12234 RANDOM_LENGTH 70 SEQ_LENGTH 63 GC_CONTENT 0.650793650793651 BASEPAIR 20 FREE_ENERGY -28.60 LEN_BP_RATIO 3.15
CGCUGGGGCUCCUUGUCUGUCUCUGUUGCCCGGGCAGGGGAAGGGAGCUUCUUGGAGUCGGCCUCUGGCA

>random_seq_from_cds__NO_12244 RANDOM_LENGTH 82 SEQ_LENGTH 62 GC_CONTENT 0.516129032258065 BASEPAIR 23 FREE_ENERGY -26.30 LEN_BP_RATIO 2.69565217391304
UCGAUAUAAGCGGAGAGUGUCAUGUAGAUUUCUCUCGAUAAGGGGUGACCCGGUUCAGCAGGAG AGAGUUGUGCAUGGAGC
>random_seq_from_cds__NO_12265 RANDOM_LENGTH 99 SEQ_LENGTH 88 GC_CONTENT 0.659090909090909 BASEPAIR 31 FREE_ENERGY -39.40 LEN_BP_RATIO 2.83870967741936
CCUCCAGCUUGCUCUCAUAGUCCCAGCCGCUGCUGCUCCAGCAGGUAGGUGGCCUCCUCCCGCUC GCGGCGGUACUGGUCCUCCAGUUCCUGGAGCCCU
>random_seq_from_cds__NO_12297 RANDOM_LENGTH 71 SEQ_LENGTH 58 GC_CONTENT 0.637931034482759 BASEPAIR 21 FREE_ENERGY -16.90 LEN_BP_RATIO 2.76190476190476
GUCCCACGCCUGCGGCACAUCAUCACUGUUGAUGGAAAGCCACCGACCUGGUCCGAGUUCCCCAA GGGCAU
>random_seq_from_cds__NO_12344 RANDOM_LENGTH 78 SEQ_LENGTH 73 GC_CONTENT 0.465753424657534 BASEPAIR 22 FREE_ENERGY -15.70 LEN_BP_RATIO 3.31818181818182
GAGGUCUUAGAGUAAAUGGUAUUCUCUUUUCAUGGUCAUCAGGCUCACUCUCAGGGUCAUCUUCG CUCUCUCUCUUCA
>random_seq_from_cds__NO_12347 RANDOM_LENGTH 82 SEQ_LENGTH 66 GC_CONTENT 0.439393939393939 BASEPAIR 25 FREE_ENERGY -22.90 LEN_BP_RATIO 2.64
AAACCCUGAUCAUCCUUACUUUCUGAGGAGUGAUGUAUUUAUCCUCUUCAUCUGUGGACACAUC AAGGGUUCUGGAUUCCU
>random_seq_from_cds__NO_12348 RANDOM_LENGTH 98 SEQ_LENGTH 86 GC_CONTENT 0.802325581395349 BASEPAIR 30 FREE_ENERGY -46.60 LEN_BP_RATIO 2.86666666666667
GAGCCUUCGUCCGCGCGGGGAGCCGCUUUGGUGGCACGCCGGGAGCUCGCCCAGACCCGGCCCCG GCCACGAGGGCCCCGAACCCGGUGGGGCAGUCC
>random_seq_from_cds__NO_12351 RANDOM_LENGTH 77 SEQ_LENGTH 64 GC_CONTENT 0.609375 BASEPAIR 21 FREE_ENERGY -25.80 LEN_BP_RATIO 3.04761904761905
CAAAGGGUACGACGAUCUACCCCAUUACGGGGGCAUGGAUGGAGUAGGCAUCCCCUCCACGAUGU AUGGGGACCCGC
>random_seq_from_cds__NO_12367 RANDOM_LENGTH 83 SEQ_LENGTH 49 GC_CONTENT 0.612244897959184 BASEPAIR 19 FREE_ENERGY -23.09 LEN_BP_RATIO 2.57894736842105
UAAUGGCCUGGAGCCAAUCGCCCACUGAUACCACAGCAGAGAAUUCAGGGGAGCUUGGAUCCAAC AAGGCAGUGUUAGGUCCU
>random_seq_from_cds__NO_12376 RANDOM_LENGTH 110 SEQ_LENGTH 58 GC_CONTENT 0.5 BASEPAIR 19 FREE_ENERGY -21.47 LEN_BP_RATIO 3.05263157894737
CUCUCUCUUGCCAGGCACUUUGAGACGCCCACUGCAUACCUCACCAAAUUCACCCAACUCCUAUA

ACUUUUUCAAUCUUAAUGCAGGAUGCGUCAAUUUCUUUGGCAAAC
>random_seq_from_cds__NO_12392 RANDOM_LENGTH 110 SEQ_LENGTH 101 GC_CONTENT 0.495049504950495 BASEPAIR 34 FREE_ENERGY -24.60 LEN_BP_RATIO 2.97058823529412
GUAAAACCCCUUUUUGCUUAAUGGCCCUACAUCCCGGAUCUCGGUGUUCAGCUUCAUGAUUCUGUCACCAAUGUCCACUUGGGUGAAGCUCUCAUCAGCAGCAAUGGUGU
>random_seq_from_cds__NO_12410 RANDOM_LENGTH 102 SEQ_LENGTH 91 GC_CONTENT 0.428571428571429 BASEPAIR 30 FREE_ENERGY -26.16 LEN_BP_RATIO 3.03333333333333
UACUUCUCCAGCUUUCUCCCAUCUCUGAGUGAUGAGGAAUCACUUCUUCUUGGAAAAACUUCCUUACACUUUUCCGGAAAAUGUCAUGCUCUGGAGAAAAGA
>random_seq_from_cds__NO_12426 RANDOM_LENGTH 84 SEQ_LENGTH 62 GC_CONTENT 0.532258064516129 BASEPAIR 22 FREE_ENERGY -22.70 LEN_BP_RATIO 2.81818181818182
CAAGAUCUCAAAUCAAGGCCGGAGUCUGUGGAAUGUAUCAGGGUAUAAUUUCCGUGGGUUUCGCUGGUUACAGGCUAUGAUAUU
>random_seq_from_cds__NO_12433 RANDOM_LENGTH 82 SEQ_LENGTH 55 GC_CONTENT 0.545454545454545 BASEPAIR 20 FREE_ENERGY -15.50 LEN_BP_RATIO 2.75
CUCUGAUGAGGAAGAGAUCCAGCAUGUGGUAGAGGUGAUUCAAAAUUCCACGGCCAAAGUCAUCGUGGUUUUCUCCAGUGGC
>random_seq_from_cds__NO_12439 RANDOM_LENGTH 97 SEQ_LENGTH 76 GC_CONTENT 0.565789473684211 BASEPAIR 22 FREE_ENERGY -16.32 LEN_BP_RATIO 3.45454545454545
ACUUAGCAGUCUACUCCAUUGCCCACGCCUUGCAAGAUAUAUAUACCUGCUUACCUGGGAGAGGGCUCUUCACCAAUGGCUCCUGUGCAGACAUCAA
>random_seq_from_cds__NO_12470 RANDOM_LENGTH 73 SEQ_LENGTH 66 GC_CONTENT 0.545454545454545 BASEPAIR 21 FREE_ENERGY -22.90 LEN_BP_RATIO 3.14285714285714
GGGGAGUGGGAGCUCUUGGGCAUCAACAAGGCCACCCCAAAGAUGUCCAUGGGCAACAACCUAUAUGACCAGA
>random_seq_from_cds__NO_12473 RANDOM_LENGTH 90 SEQ_LENGTH 70 GC_CONTENT 0.585714285714286 BASEPAIR 23 FREE_ENERGY -20.50 LEN_BP_RATIO 3.04347826086957
CUUCCUGCUCAUGAUGAAUGACUUGCUCCCUGCCAGUGGCACCCCCCUCAUCAGGUGUCUACUUCGCCCUGUGCCUGUCCCUGAUGGUGG
>random_seq_from_cds__NO_12478 RANDOM_LENGTH 85 SEQ_LENGTH 72 GC_CONTENT 0.486111111111111 BASEPAIR 24 FREE_ENERGY -24.50 LEN_BP_RATIO 3
UCCUCUGGAACACCUAGAAUGGCUUCUGUUUGGCAGAGAUUGGGUUUUUAUGCCUCUCUUCUGAAAAGACAGCUAAAUGGUGGGC
>random_seq_from_cds__NO_12482 RANDOM_LENGTH 85 SEQ_LENGTH 73 GC_CONTENT 0.534246575342466 BASEPAIR 27 FREE_ENERGY -27.70 LEN_BP_RATIO 2.7037037037037

AUCCUUCUGGUAAGCUGCACAUGGGCCAUGUGCGUGUCUACACCAUCAGCGACACCAUAGCACGG
UUCCAGAAGAUGAGAGGGAU
>random_seq_from_cds__NO_12486 RANDOM_LENGTH 90 SEQ_LENGTH 82 GC_CONTENT 0.548780487804878 BASEPAIR 25 FREE_ENERGY -26.16 LEN_BP_RATIO 3.28
ACUGGGACCCAGUGGAUCAAACAGUGCUUGCCAAUGAGCAGGUGGAUGAACAUGGCUGUUCAUGG
CGUUCUGGAGCAAAGGUGGAACAGA
>random_seq_from_cds__NO_12494 RANDOM_LENGTH 73 SEQ_LENGTH 66 GC_CONTENT 0.606060606060606 BASEPAIR 23 FREE_ENERGY -18.20 LEN_BP_RATIO 2.8695652173913
CACACCAAUCCCCAUUGUCCACUGCCCAGUCUGUGGCCCCACACCUGUGCCCCUGGAGGACUUGC
CUGUGACC
>random_seq_from_cds__NO_12559 RANDOM_LENGTH 84 SEQ_LENGTH 73 GC_CONTENT 0.479452054794521 BASEPAIR 25 FREE_ENERGY -16.70 LEN_BP_RATIO 2.92
GGGAUGUCCGUCCAUUGAUACAAACCAAAGAUGGAGAAAAUUACGCUACAGUGGUUGGGUACAGU
GCUUUCUUACAUUGCGAGU
>random_seq_from_cds__NO_12562 RANDOM_LENGTH 110 SEQ_LENGTH 92 GC_CONTENT 0.402173913043478 BASEPAIR 25 FREE_ENERGY -16.75 LEN_BP_RATIO 3.68
AUAGGAAAAACUGCAGUCACAGCCAAUUUGGAUAUUAGAAGAUGCUACAAAACUUAGAGUUUCUC
CUAAGAAUCCUCGUAUCCCCAAAUUGCAUAUGCUUGAAUUACAUU
>random_seq_from_cds__NO_12567 RANDOM_LENGTH 88 SEQ_LENGTH 81 GC_CONTENT 0.45679012345679 BASEPAIR 27 FREE_ENERGY -29.33 LEN_BP_RATIO 3
AACAAAGAAGAGCCUGGAAGGUGGGAGGAACUGACCAGAGUCCAAGGAAAGAAAACCACAGUUAU
CUUACCUUUGGCUCCAUUUGUGA
>random_seq_from_cds__NO_12585 RANDOM_LENGTH 82 SEQ_LENGTH 64 GC_CONTENT 0.5 BASEPAIR 20 FREE_ENERGY -20.20 LEN_BP_RATIO 3.2
UAUUGGUGCCUACGCUGGAUCUAAGGAGAAGGGAUCUGUUGAAAGCAAUGGAAGUUCUACAGCAA
CUUUUCCCCUUCGGGCA
>random_seq_from_cds__NO_12591 RANDOM_LENGTH 90 SEQ_LENGTH 68 GC_CONTENT 0.485294117647059 BASEPAIR 24 FREE_ENERGY -27.30 LEN_BP_RATIO 2.83333333333333
AGAAGGUCAAGGUGUGGUGCUUCUCUGUGGCCCACCGCCACAUUUUGGAGGAUUUAUCUUAUGCA
UGGACCUUCAAUGAUAACCCCUUAU
>random_seq_from_cds__NO_12611 RANDOM_LENGTH 85 SEQ_LENGTH 75 GC_CONTENT 0.48 BASEPAIR 24 FREE_ENERGY -17.30 LEN_BP_RATIO 3.125
UGAACUCGUCAUUACGUGGGAGGUCAAUUCCAGAAGAACUGCAGAAUGGGGAGGGAUUUGGAUAU
AUCAUCAUGUUCCGGCCAGU
>random_seq_from_cds__NO_12619 RANDOM_LENGTH 82 SEQ_LENGTH 63 GC_CONTENT 0.428571428571429 BASEPAIR 22 FREE_ENERGY

-20.20 LEN_BP_RATIO 2.86363636363636
CAUCAGCUGAGCUUCUGGUUCCAUUUGAAGAAGACUACUUAAUUGAAAUAAGAACAGUCAGUGAU
GGUGGAGAUGGAAGCAG
>random_seq_from_cds__NO_12620 RANDOM_LENGTH 81 SEQ_LENGTH 74
GC_CONTENT 0.378378378378378 BASEPAIR 26 FREE_ENERGY
-17.70 LEN_BP_RATIO 2.84615384615385
CAGUGAGGAAAUUAGGAUUCCAAAAAUGUCAAGGUUUGAGUUCCAGAGGAAUUCAAUUCUUAGAA
CCUAGCACCCAUUUUC
>random_seq_from_cds__NO_12627 RANDOM_LENGTH 86 SEQ_LENGTH 79
GC_CONTENT 0.518987341772152 BASEPAIR 26 FREE_ENERGY
-19.70 LEN_BP_RATIO 3.03846153846154
CUCGCGCUGCCGUGGACUUUUGGAGAAUGAGUUGAAACUGAUGGAAGAAUUUGUCAAGCAAUAUA
AGAGCGAGGCCCUCGGCGUGG
>random_seq_from_cds__NO_12630 RANDOM_LENGTH 90 SEQ_LENGTH 78
GC_CONTENT 0.615384615384615 BASEPAIR 25 FREE_ENERGY
-29.60 LEN_BP_RATIO 3.12
CCCACCUGCUUUGUGUGUGCCAAGUGCUCCGAGCCGCUGGUGGACCUCAUCUACUUCUGGAAGGA
UGGUGCACCCUGGUGCGGCCGCCAU
>random_seq_from_cds__NO_12649 RANDOM_LENGTH 88 SEQ_LENGTH 74
GC_CONTENT 0.513513513513513 BASEPAIR 26 FREE_ENERGY
-28.90 LEN_BP_RATIO 2.84615384615385
CUGAUGGCUCCCAGUACUAUGUUCUGCUCAUCAUCACUGAUGGGGUCAUCUCUGACAUGACGCAG
ACCAAGGAGGCCAUCGUCAGCGG
>random_seq_from_cds__NO_12650 RANDOM_LENGTH 86 SEQ_LENGTH 76
GC_CONTENT 0.486842105263158 BASEPAIR 26 FREE_ENERGY
-23.30 LEN_BP_RATIO 2.92307692307692
CCUCCUCAUUGCCCAUGUCUAUCAUUAUCGUCGGUGUAGGACCAGCCAUGUUUGAGGGCAAUGGA
AGAGUUGGACGGUGAUGAUGU
>random_seq_from_cds__NO_12665 RANDOM_LENGTH 88 SEQ_LENGTH 75
GC_CONTENT 0.653333333333333 BASEPAIR 25 FREE_ENERGY
-30.10 LEN_BP_RATIO 3
CCGUUGCCUGCAGUCACCCUCUCGUGCUGUGGAUUGUGCCCUGUGCCCCAACAAGGGCGGUGCCU
UCAAGCAGACAGAUGACGGGCGC
>random_seq_from_cds__NO_12671 RANDOM_LENGTH 73 SEQ_LENGTH 60
GC_CONTENT 0.666666666666667 BASEPAIR 20 FREE_ENERGY
-22.60 LEN_BP_RATIO 3
GAUCCUGGCAGAGAAGCGGGCAGCAGCACCUGUGGUGUCAGUGCCCUGCAUCCCACCACACAGGG
CUUAGUAA
>random_seq_from_cds__NO_12678 RANDOM_LENGTH 82 SEQ_LENGTH 67
GC_CONTENT 0.701492537313433 BASEPAIR 26 FREE_ENERGY
-31.70 LEN_BP_RATIO 2.57692307692308
ACACCAUCUUCUACCGGGCAGCAGUGCGGCUUCGUGAGCAGGGUGGUGCUGUGCUCCGCCAGGCC
CGGCGCCAGGCAGAAAA
>random_seq_from_cds__NO_12696 RANDOM_LENGTH 110 SEQ_LENGTH

104 GC_CONTENT 0.644230769230769 BASEPAIR 37 FREE_ENERGY -41.60 LEN_BP_RATIO 2.81081081081081
GGGCUCUGCAGCACCGCAGCAAGGUGCAAGGCGAGCAGAGCAGUGAGACCAGCGAUAGUGAUUGA
GAUGGACUGCCACUCUCCGCCCCUACCUGAGUGCCGUGCGGGCCA
>random_seq_from_cds__NO_12699 RANDOM_LENGTH 88 SEQ_LENGTH 62 GC_CONTENT 0.419354838709677 BASEPAIR 24 FREE_ENERGY -20.60 LEN_BP_RATIO 2.58333333333333
UGUGAAACAGGGCUGAUGAGAUCGAGAAGAUUUUGUGCCACAAGUUCAUGCGCUUCAUGAUGAUG
CGAGCAGAGAACUUCUUUAUCCU
>random_seq_from_cds__NO_12714 RANDOM_LENGTH 87 SEQ_LENGTH 73 GC_CONTENT 0.534246575342466 BASEPAIR 20 FREE_ENERGY -18.12 LEN_BP_RATIO 3.65
AGUACCUGCAUGGUCUGGAGAUCAUCCACAGCAACGUCAAGAGGCUCUAAUGUCUUGCUGGACCA
AAAUCUCACCCCCAAACUUGCU
>random_seq_from_cds__NO_12731 RANDOM_LENGTH 89 SEQ_LENGTH 70 GC_CONTENT 0.557142857142857 BASEPAIR 22 FREE_ENERGY -24.50 LEN_BP_RATIO 3.18181818181818
AGCUGGAACAUCUGAAACUUGGUCUCCUGGACAUGCAGGUGUUUGUAGAGGCUGCUGCCCUCGCA
CCACUGGGUCACAAUUGCCAGGUU
>random_seq_from_cds__NO_12737 RANDOM_LENGTH 88 SEQ_LENGTH 72 GC_CONTENT 0.513888888888889 BASEPAIR 28 FREE_ENERGY -30.80 LEN_BP_RATIO 2.57142857142857
UGUGACUUCGAAUUGCAUCCCUCAAUCAUCCUGCUGUCCACAGGCAGGGUGGUGCUGACCAUGUG
GACAUUAGGUGUGGAUGUCGACC
>random_seq_from_cds__NO_12747 RANDOM_LENGTH 110 SEQ_LENGTH 101 GC_CONTENT 0.544554455445545 BASEPAIR 29 FREE_ENERGY -31.77 LEN_BP_RATIO 3.48275862068966
CCUGUAUGUGCUCCAUCCUACUCAUUUUCUUCACUGCGCAGCCUGGCAUUGGGGUUGGUGACUCU
GAUGGCCAGUUGGGCAGCUCUUUCCACGAUGGCUUUGCGGUUCUU
>random_seq_from_cds__NO_12755 RANDOM_LENGTH 88 SEQ_LENGTH 74 GC_CONTENT 0.648648648648649 BASEPAIR 28 FREE_ENERGY -30.00 LEN_BP_RATIO 2.64285714285714
AGAUCAGCUGCCAGUUCAUGCUGUGCCCGGAGCUGCCGCCCAACUGCAUCGAGGCUGUAGUGGUG
GCUGACAGCUGCCCACAGUGCGG
>random_seq_from_cds__NO_12762 RANDOM_LENGTH 90 SEQ_LENGTH 86 GC_CONTENT 0.651162790697674 BASEPAIR 29 FREE_ENERGY -45.40 LEN_BP_RATIO 2.96551724137931
CUGGAUGGGCUGCCCACUACAGCCCCAGCCUGGACCCAGUCUUCCUAUCCAGGAGGAGAGGGCAG
AAGCUGGGGCAAGGGCAGAAGCUGG
>random_seq_from_cds__NO_12764 RANDOM_LENGTH 84 SEQ_LENGTH 65 GC_CONTENT 0.692307692307692 BASEPAIR 20 FREE_ENERGY -29.00 LEN_BP_RATIO 3.25
UGGGCAAGGCUGCUCUCGUCCCAACUCAGGCCGUGCCUGGCUCUCCCAGGGACCCAGUCAAGCCC
AGCCCCCACAACAUCCUGU

>random_seq_from_cds__NO_12772 RANDOM_LENGTH 78 SEQ_LENGTH 66 GC_CONTENT 0.696969696969697 BASEPAIR 24 FREE_ENERGY -35.30 LEN_BP_RATIO 2.75
CACCACGGAGAGGUUUCAGAGGCAGAGAUGGCGGGCCGAGAGGCCCUGUCACUGGGCACAGAGGC
CGAGCUGCCGAAC
>random_seq_from_cds__NO_12781 RANDOM_LENGTH 84 SEQ_LENGTH 72 GC_CONTENT 0.625 BASEPAIR 25 FREE_ENERGY -23.80 LEN_BP_RATIO 2.88
AACCCGCUGAUCUGCGCGCGCGGCUACCACGCCAGCGAUGAUGGGACCAAGUGUGUGGGACGUGA
AUGAGUGUGAGACAGGUGU
>random_seq_from_cds__NO_12783 RANDOM_LENGTH 98 SEQ_LENGTH 79 GC_CONTENT 0.658227848101266 BASEPAIR 27 FREE_ENERGY -36.50 LEN_BP_RATIO 2.92592592592593
UUUGGCCGGGGCUGCAUCGGACGUGAAUGAGUGCUGGGCCUCGCCAGGCCGCCUGUGCCAGCACA
CGUGUGAGAACACACUCGGCUCCUACCGCUGUU
>random_seq_from_cds__NO_12796 RANDOM_LENGTH 84 SEQ_LENGTH 71 GC_CONTENT 0.450704225352113 BASEPAIR 25 FREE_ENERGY -16.80 LEN_BP_RATIO 2.84
ACCUUGACAUCUAUGAACAGCAAGUGAUGACUGCAGCCCAAAAGGGAUGUACAGAUUAUAGUGUU
UCCAGAAGAUGGCAUUCAU
>random_seq_from_cds__NO_12817 RANDOM_LENGTH 80 SEQ_LENGTH 59 GC_CONTENT 0.423728813559322 BASEPAIR 25 FREE_ENERGY -19.30 LEN_BP_RATIO 2.36
GAUGACCUGAACUGGUGAAUUUGGGUAAGUAGGAUAUGCCCUGAACAUACUGAGUUAUAGGAUUC
AUCGUGGUUGUGGGC
>random_seq_from_cds__NO_12824 RANDOM_LENGTH 64 SEQ_LENGTH 59 GC_CONTENT 0.576271186440678 BASEPAIR 19 FREE_ENERGY -21.11 LEN_BP_RATIO 3.10526315789474
CUUGCCAAUGCCCAGGUGGCUAAGGGUUUCCCCAUAGUCUACUGUUCCGAUGGCUUCUGCGAGC
>random_seq_from_cds__NO_12825 RANDOM_LENGTH 85 SEQ_LENGTH 69 GC_CONTENT 0.449275362318841 BASEPAIR 22 FREE_ENERGY -18.30 LEN_BP_RATIO 3.13636363636364
UUGCUGGAUUUGCCCGAACUGAAGUCAUGCAGAAGAGUUGUAGCUGCAAGUUCUUAUUUGGGGUU
GAAACCAAUGAGCAACUGAU
>random_seq_from_cds__NO_12837 RANDOM_LENGTH 82 SEQ_LENGTH 65 GC_CONTENT 0.492307692307692 BASEPAIR 24 FREE_ENERGY -17.40 LEN_BP_RATIO 2.70833333333333
GGGAAGACAACAGCCUUCUGAAGUGGGAAGUUGGGUUGGCUUCAUGAGUUGGGAAAGAGACUGGA
AUCUCCAUACUAUGGCA
>random_seq_from_cds__NO_12838 RANDOM_LENGTH 81 SEQ_LENGTH 61 GC_CONTENT 0.639344262295082 BASEPAIR 22 FREE_ENERGY -21.56 LEN_BP_RATIO 2.77272727272727
ACAAUACCUUGGGGGGCCCGUCGAUCCGAAGUGCCUAUAUUGCCGCUCUGUACUUCACGCUGAGC
AGCCUCACCAGCGUGG

>random_seq_from_cds__NO_12862 RANDOM_LENGTH 110 SEQ_LENGTH 90 GC_CONTENT 0.477777777777778 BASEPAIR 35 FREE_ENERGY -30.60 LEN_BP_RATIO 2.57142857142857
AAUCAACAUCAUCUUCUUCUUCAUCAGACUCUGCAAAAUAUUUUACUUCUUUCCUAGCCCGACCG
GUUCGUGGCAGAGAAGGUGGCUCAGUAGGGAAGUCUGAGGGGAAG
>random_seq_from_cds__NO_12874 RANDOM_LENGTH 75 SEQ_LENGTH 55 GC_CONTENT 0.454545454545455 BASEPAIR 19 FREE_ENERGY -15.50 LEN_BP_RATIO 2.89473684210526
GCAUCUGCCUUCAUAGCUGUAAUUUCAGGAAUUAUUCUUCUGCCAUAAGGUGAGGGCAUUGUCUC
UUCCAACUGG
>random_seq_from_cds__NO_12890 RANDOM_LENGTH 98 SEQ_LENGTH 76 GC_CONTENT 0.407894736842105 BASEPAIR 25 FREE_ENERGY -16.70 LEN_BP_RATIO 3.04
UGACCAAUAGGCUGAAGCAAGUUAAUGUUGUUACUUCCCACAAAGUUCUGAGCCAAAUUCACAAU
AGUCAUCAUCAAUGCUUGCUUCUCCAUGAUGAU
>random_seq_from_cds__NO_12917 RANDOM_LENGTH 82 SEQ_LENGTH 56 GC_CONTENT 0.785714285714286 BASEPAIR 21 FREE_ENERGY -22.90 LEN_BP_RATIO 2.66666666666667
CAGCAUUGUUCCACCCAGGUCAGUGCCCCGUUGCCGCCGCCCACGCCGGCUCCCGCGCCGCAGCC
ACCCGACUUGGCCAUUU
>random_seq_from_cds__NO_12926 RANDOM_LENGTH 87 SEQ_LENGTH 75 GC_CONTENT 0.4 BASEPAIR 24 FREE_ENERGY -18.00 LEN_BP_RATIO 3.125
GACAAUCUGAUGGGAUCGAAUCAUAGUUGUCUCUGAAGCCUUUUGCAAAGGGGUUAUGAUCAAUC
UUUAGUUGAGUAAUCAUCGGUG
>random_seq_from_cds__NO_12930 RANDOM_LENGTH 88 SEQ_LENGTH 76 GC_CONTENT 0.460526315789474 BASEPAIR 22 FREE_ENERGY -19.60 LEN_BP_RATIO 3.45454545454545
AGUAUUAGGAGACUCUGGGUGAACAUACAUUUUGUUGCCCCUGCAUGUUAUUGUCGGCUUUGCCA
CAGGUCACCCAUUUGCCCCCCUG
>random_seq_from_cds__NO_12935 RANDOM_LENGTH 85 SEQ_LENGTH 72 GC_CONTENT 0.805555555555556 BASEPAIR 26 FREE_ENERGY -39.80 LEN_BP_RATIO 2.76923076923077
CCGCUGCUACCGCCCGCGCCACUGCCCGCACCGGCUCCUGGGCCGAACUGCGCCCUCCCGGGUGG
GCACACAGCCGCGGGGAAGC
>random_seq_from_cds__NO_12937 RANDOM_LENGTH 85 SEQ_LENGTH 71 GC_CONTENT 0.690140845070423 BASEPAIR 26 FREE_ENERGY -35.80 LEN_BP_RATIO 2.73076923076923
ACGGGAAGAGUGAGCAGGGCGCAGCCAGCUCCGACCCCUGAGGACCGGGGGACUGGAGGUAGUAC
CGCUCGGAGCUCAGGCUGUC
>random_seq_from_cds__NO_12940 RANDOM_LENGTH 80 SEQ_LENGTH 63 GC_CONTENT 0.825396825396825 BASEPAIR 23 FREE_ENERGY -39.30 LEN_BP_RATIO 2.73913043478261
GCAUGGCCGCGGGGGCCCCUGCGCUGGCGGCUGCGGGCUCCCCGCUCACCGCCUCGCAGGAGAGA

CUGCCGGAAAACUUC
>random_seq_from_cds__NO_12955 RANDOM_LENGTH 81 SEQ_LENGTH 47 GC_CONTENT 0.553191489361702 BASEPAIR 19 FREE_ENERGY -16.62 LEN_BP_RATIO 2.47368421052632
AUGAAUCACCUUUCGUUGGGCACAACAGGAACGGUAUAUGACUGCUGCUGCUCCUAUGCAAGGGA
CCUACAUUCCUCAGUA
>random_seq_from_cds__NO_12972 RANDOM_LENGTH 85 SEQ_LENGTH 56 GC_CONTENT 0.5 BASEPAIR 22 FREE_ENERGY -23.40 LEN_BP_RATIO 2.54545454545455
CACUUUUUUCAUCUUCAGACAAAACCAAUUCAGAGCCUGAGUUUGGCCUCUGGCUCUGGCUGUGA
GGUUUGUUCGUCUUCAGCAG
>random_seq_from_cds__NO_12977 RANDOM_LENGTH 89 SEQ_LENGTH 78 GC_CONTENT 0.58974358974359 BASEPAIR 28 FREE_ENERGY -33.90 LEN_BP_RATIO 2.78571428571429
UGAUUGUGACAACACCGGGGGCCCCCACACUGAGGUGUCUCUGGCUACAGGGAGACGCAGAAUUG
GGUGUUCCAUGUGGGAGCAAAGUG
>random_seq_from_cds__NO_12980 RANDOM_LENGTH 88 SEQ_LENGTH 77 GC_CONTENT 0.571428571428571 BASEPAIR 26 FREE_ENERGY -27.10 LEN_BP_RATIO 2.96153846153846
AGAGUACACCACCAGCAUGUGGGGAGCUUCAUCGCUCAGGGACACUAUGGCUCCAGAUAAAGACA
GGACUCCUCGAGGCUUCUGGUGU
>random_seq_from_cds__NO_13004 RANDOM_LENGTH 110 SEQ_LENGTH 93 GC_CONTENT 0.505376344086022 BASEPAIR 30 FREE_ENERGY -24.93 LEN_BP_RATIO 3.1
GGAGCAAGUUACUCAGAUGGAAAACUGAAAGCCCCUCCUAAACCAUGUGCUGGCAAUCAAGGGAC
CCAGAUCACGGGUGGAGGACCUUUUUUACAACAUAGCCACGAGGA
>random_seq_from_cds__NO_13013 RANDOM_LENGTH 82 SEQ_LENGTH 73 GC_CONTENT 0.547945205479452 BASEPAIR 29 FREE_ENERGY -25.30 LEN_BP_RATIO 2.51724137931034
CCCUGUCCAGUCAGCCCCAGGCCAUUGUCACAGAGGAUAAGACAGAUAUUUCUAGUGGCAGGGCU
AGGCAGCAAGAUGAGGA
>random_seq_from_cds__NO_13014 RANDOM_LENGTH 80 SEQ_LENGTH 65 GC_CONTENT 0.569230769230769 BASEPAIR 21 FREE_ENERGY -23.32 LEN_BP_RATIO 3.0952380952381
GAUGCUUGAACUCCCAGCCCCUGCUGAAGUGGCUGCCAAAAAUCAGAGCUUGGAGGGGGAUACAA
CAAAGGGGACUUCAG
>random_seq_from_cds__NO_13019 RANDOM_LENGTH 84 SEQ_LENGTH 75 GC_CONTENT 0.6 BASEPAIR 26 FREE_ENERGY -27.10 LEN_BP_RATIO 2.88461538461538
CGGGAGCCAGCACCGCUCUUUGACCUUGCCAUGCUUGCCUUAGAUAGUCCAGAGAGUGGCUGGAC
AGAGGAAGAUGGUCCCAAA
>random_seq_from_cds__NO_13020 RANDOM_LENGTH 84 SEQ_LENGTH 55 GC_CONTENT 0.363636363636364 BASEPAIR 20 FREE_ENERGY -16.20 LEN_BP_RATIO 2.75

GAAGGACUUGCUGAAUACAUUGUUGAGUUUCUGAAGAAGAAGGCUGAGAUGCUUGCAGACUAUUU
CUCUUUGGAAAUUGAUGAG
>random_seq_from_cds__NO_13022 RANDOM_LENGTH 82 SEQ_LENGTH 62 GC_CONTENT 0.451612903225806 BASEPAIR 21 FREE_ENERGY -16.80 LEN_BP_RATIO 2.95238095238095
UCAUUCUUCGACUAGCCACUGAGGGUGAAUUGGGACGAAGAAAAGGAAUGUUUUGAAAGCCUCAG
UAAAGAAUGCGCUAUGU
>random_seq_from_cds__NO_13032 RANDOM_LENGTH 88 SEQ_LENGTH 59 GC_CONTENT 0.576271186440678 BASEPAIR 20 FREE_ENERGY -22.10 LEN_BP_RATIO 2.95
CGGCGGGAGGGGAUAAAUCGAUUCAUGUAAAGCUGGACGUGGGCAAGCUGCACACCCAGCCUAAG
UUAGCGGCCCAGCUCAGGAUGGU
>random_seq_from_cds__NO_13038 RANDOM_LENGTH 97 SEQ_LENGTH 91 GC_CONTENT 0.582417582417582 BASEPAIR 26 FREE_ENERGY -35.90 LEN_BP_RATIO 3.5
UGCCAGCCCGUGCCUCAUCCCUCAACUCCAGUGACAUCUUCUUGCUGGUCACAGCCAGCGUCUGC
UACCUCUGGUUUGGGAAGGGGCUGUAAUGGUG
>random_seq_from_cds__NO_13040 RANDOM_LENGTH 70 SEQ_LENGTH 59 GC_CONTENT 0.728813559322034 BASEPAIR 21 FREE_ENERGY -29.30 LEN_BP_RATIO 2.80952380952381
UUCUGGGAGGCCCUGGGAGGCCGGGCCCCCUACCCCAGCAACAAGAGGGCUCCCUGAGGAGGUCC
CCAGC
>random_seq_from_cds__NO_13045 RANDOM_LENGTH 87 SEQ_LENGTH 71 GC_CONTENT 0.690140845070423 BASEPAIR 24 FREE_ENERGY -37.30 LEN_BP_RATIO 2.95833333333333
GCGGCUAUCCAGAUGGCCGGGCAAUGGCAGGGCAGGUGCCGUGGCCCUGCAGGCCCUCAAGGGCU
CCCAGGACAGCUCAGAGAAUGA
>random_seq_from_cds__NO_13052 RANDOM_LENGTH 92 SEQ_LENGTH 84 GC_CONTENT 0.535714285714286 BASEPAIR 30 FREE_ENERGY -24.10 LEN_BP_RATIO 2.8
CAGCCCUGGCUUGCAAAGGCCUCAUUGAUCUCGAAGAUGUCCACGUCACUCACUGUCAGCCCCUG
CUUUUUGCAAAGCUACUGGGAUGGCAU
>random_seq_from_cds__NO_13072 RANDOM_LENGTH 110 SEQ_LENGTH 84 GC_CONTENT 0.380952380952381 BASEPAIR 29 FREE_ENERGY -20.90 LEN_BP_RATIO 2.89655172413793
CUCCUUAUCAUAAAUAUCCACCAAUGAAGGGUUUUAAUGCUGACACUGCAGAACGAUCCUCCUUC
UUUGGAAACUGGUGUUCAAGAUAAAGAAAUGCUGAAAAAAUAUGG
>random_seq_from_cds__NO_13085 RANDOM_LENGTH 63 SEQ_LENGTH 52 GC_CONTENT 0.634615384615385 BASEPAIR 20 FREE_ENERGY -16.60 LEN_BP_RATIO 2.6
CUGAACAGCUGGUACUGAGCGUGCCCCUGGACACUGCAGGUCACCCAGAGCCCUGCCUCAUGU
>random_seq_from_cds__NO_13087 RANDOM_LENGTH 84 SEQ_LENGTH 65 GC_CONTENT 0.538461538461538 BASEPAIR 21 FREE_ENERGY -17.90 LEN_BP_RATIO 3.0952380952381

UAUCCUGAGAACAGGCUCCCAUCCCUGAAGAAUGAGGUUCAACCUGGUUUGUGAUCGGAAGCACC
UGAAGGACACCACACAGUC
>random_seq_from_cds__NO_13098 RANDOM_LENGTH 82 SEQ_LENGTH 70
GC_CONTENT 0.585714285714286 BASEPAIR 24 FREE_ENERGY
-25.82 LEN_BP_RATIO 2.91666666666667
UGCCCGUGGUGGUCACCAUGCUGGCUGUGGUGGGGAAGAUGGCCACAGCUGCUGCCUUUACCAUC
UCCUAUGUGUACUCUGC
>random_seq_from_cds__NO_13112 RANDOM_LENGTH 85 SEQ_LENGTH 71
GC_CONTENT 0.591549295774648 BASEPAIR 24 FREE_ENERGY
-20.86 LEN_BP_RATIO 2.95833333333333
UAUCCUGGGACACUGCUUUUUCGCUGUUGGGGCCGUGUUGCUGACAGGGAUCGCCUACAGUCUUC
CCCACUGGCAGCUGCUGUUU
>random_seq_from_cds__NO_13115 RANDOM_LENGTH 75 SEQ_LENGTH 58
GC_CONTENT 0.53448275862069 BASEPAIR 20 FREE_ENERGY
-19.00 LEN_BP_RATIO 2.9
UGCCCAGAAAGAAGGUGACUCGGGCCUCUGUCCUGGACUUCUGUAAGAAUAGGCAGCUCUGCAAG
GUGACCUUGG
>random_seq_from_cds__NO_13125 RANDOM_LENGTH 90 SEQ_LENGTH 70
GC_CONTENT 0.5 BASEPAIR 26 FREE_ENERGY -28.20 LEN_BP_RATIO
2.69230769230769
UGCACAAGGAUGGGCUGACGGUCACUUCUCCAGUACUAAUGUGGGUCCAGGGCACUGGAUAUCAU
CUUGGAGAAGAUGAAGGCUUCGGGC
>random_seq_from_cds__NO_13129 RANDOM_LENGTH 85 SEQ_LENGTH 59
GC_CONTENT 0.525423728813559 BASEPAIR 24 FREE_ENERGY
-17.90 LEN_BP_RATIO 2.45833333333333
AAAUUGCAAAAAUUUACCAGCAGAACCCCGAGGCCUACUCACAUACGGAGGAGAAUUUCUUUGGU
CAGUAGCUUUGCUGCUUCCC
>random_seq_from_cds__NO_13131 RANDOM_LENGTH 78 SEQ_LENGTH 58
GC_CONTENT 0.586206896551724 BASEPAIR 21 FREE_ENERGY
-21.11 LEN_BP_RATIO 2.76190476190476
CUGGUCCCAGGCUUGCCUUGGUGCCUGUGCACCUCAUUUAGAGGAGAAGCUUAGCCCACCAGUAC
CAUCAUGCUCAGU
>random_seq_from_cds__NO_13133 RANDOM_LENGTH 88 SEQ_LENGTH 78
GC_CONTENT 0.615384615384615 BASEPAIR 26 FREE_ENERGY
-29.60 LEN_BP_RATIO 3
CUGGCAGGCAUGAGACUGGAGGAAGGUGACAUUGCGGGUCAGCCUGGGCACCAGUGACACCCUGU
UUCUCUGGCUCCAAGAGCCCAUG
>random_seq_from_cds__NO_13134 RANDOM_LENGTH 82 SEQ_LENGTH 71
GC_CONTENT 0.549295774647887 BASEPAIR 19 FREE_ENERGY
-22.14 LEN_BP_RATIO 3.73684210526316
CCUGCCCUGGAAGGCCACAUCUUCUGCAACCCGGUUGACUCCCAGCACUACAUGGCACUCCUGUG
GCUUUAAAAAUGGCUCC
>random_seq_from_cds__NO_13142 RANDOM_LENGTH 84 SEQ_LENGTH 60
GC_CONTENT 0.766666666666667 BASEPAIR 24 FREE_ENERGY

-32.30 LEN_BP_RATIO 2.5
GAAUCUUGUCUCAGACCCGGGGGCCUCCGGAGUGACAUGACGGCGCCCUGGGUGGCCCUCGCCCU
CCUCUGGGGAUCGCUGUGC
>random_seq_from_cds__NO_13149 RANDOM_LENGTH 84 SEQ_LENGTH 67
GC_CONTENT 0.686567164179104 BASEPAIR 23 FREE_ENERGY
-33.10 LEN_BP_RATIO 2.91304347826087
GGUGGGCCUGAAGCCACUGCAGCUGCUGGAGAUCAAGGCUCGGGGGCGCUUUGGCUGUGUCUGGA
AGGCCCAGCUCAUGAAUGA
>random_seq_from_cds__NO_13160 RANDOM_LENGTH 90 SEQ_LENGTH 71
GC_CONTENT 0.549295774647887 BASEPAIR 24 FREE_ENERGY
-27.70 LEN_BP_RATIO 2.95833333333333
CAUCUAAGUUAUUCUGUGGUAGAGAUCUGGGCCUGGCUGUCCAGCCCCUCAGCCUCCGUCCCAGU
AUCUAGGCCCUCUGUGCUUGCUGGU
>random_seq_from_cds__NO_13173 RANDOM_LENGTH 81 SEQ_LENGTH 71
GC_CONTENT 0.535211267605634 BASEPAIR 23 FREE_ENERGY
-18.20 LEN_BP_RATIO 3.08695652173913
UUCUCCACAUUGGCUUUCAGUAGUGCCUUCAGGGUGUCAUUCUCCUUGUUCCAGCCUCGAGUGCC
CAAUGGUGAUGAUGAA
>random_seq_from_cds__NO_13175 RANDOM_LENGTH 77 SEQ_LENGTH 58
GC_CONTENT 0.637931034482759 BASEPAIR 20 FREE_ENERGY
-24.10 LEN_BP_RATIO 2.9
GGCUGCUCAGCGCUGACGCCCAGGCCCAUCUCACACCGGCACAGGCUCCAUUAGAGAUGCUGGGA
CAGUGUCCUCAA
>random_seq_from_cds__NO_13181 RANDOM_LENGTH 87 SEQ_LENGTH 52
GC_CONTENT 0.557692307692308 BASEPAIR 19 FREE_ENERGY
-20.90 LEN_BP_RATIO 2.73684210526316
CACCAGGGUCACUAGUACCAAAGAUGUCAGCUGGAUGGAAGCAGCUGAGGUUGUCCAGGUUCCCC
ACGCUCCCUUCCUCCUCUUCCU
>random_seq_from_cds__NO_13194 RANDOM_LENGTH 87 SEQ_LENGTH 73
GC_CONTENT 0.657534246575342 BASEPAIR 28 FREE_ENERGY
-33.20 LEN_BP_RATIO 2.60714285714286
GACAGGGCGGGUGAAACUUCUGGGGCCGCAGAAGUCACGGUCAGGCAGGGGCAUCUGAUCCCAGG
GGCCUUCCUCCUCUGAGUCCCA
>random_seq_from_cds__NO_13206 RANDOM_LENGTH 90 SEQ_LENGTH 82
GC_CONTENT 0.536585365853659 BASEPAIR 24 FREE_ENERGY
-30.20 LEN_BP_RATIO 3.41666666666667
CACGGAAGACUGGUCUGCAGCUCCCACUGCUCAGGCCACUGAAUGGGUAGGAGCAACCACUGACU
GGUCUUAAGAUGUUCACUGUGCUGA
>random_seq_from_cds__NO_13209 RANDOM_LENGTH 82 SEQ_LENGTH 71
GC_CONTENT 0.464788732394366 BASEPAIR 22 FREE_ENERGY
-20.70 LEN_BP_RATIO 3.22727272727273
UGGUAUUGUGCUGGAUGCCGGGUCUUCAAGAACCACAGUCUACGUGUAUCAAUGGCCAGCAGAAA
AAGAGAAUAAUACCGGA
>random_seq_from_cds__NO_13214 RANDOM_LENGTH 71 SEQ_LENGTH 61

GC_CONTENT 0.639344262295082 BASEPAIR 20 FREE_ENERGY -30.80 LEN_BP_RATIO 3.05
UCCUGGAGGAAGAACCUGUGGCACAUGUGGGUGCACCCGCAUGGAGUGGAAACCACGGGUGCCCUGGACUU
>random_seq_from_cds__NO_13220 RANDOM_LENGTH 92 SEQ_LENGTH 78 GC_CONTENT 0.448717948717949 BASEPAIR 25 FREE_ENERGY -19.00 LEN_BP_RATIO 3.12
CAAAGAUUAAAGGGCCAUUUGUGGGCUUUUGCAGGAUUCUACUACACAGCCAGUGCUUUAAAUCUUUCAGGUAGCUUUUCCCUGGACACCUU
>random_seq_from_cds__NO_13237 RANDOM_LENGTH 85 SEQ_LENGTH 81 GC_CONTENT 0.827160493827161 BASEPAIR 32 FREE_ENERGY -48.10 LEN_BP_RATIO 2.53125
CCGCCCGCUGGCUAUGCGUGCUGGCAGGCGCCCUCGCCUGGGCCCUUGGGCCGGCGGGGCGGCCAGGCGGCCAGGCUGCAGGAGG
>random_seq_from_cds__NO_13244 RANDOM_LENGTH 89 SEQ_LENGTH 69 GC_CONTENT 0.608695652173913 BASEPAIR 25 FREE_ENERGY -25.26 LEN_BP_RATIO 2.76
AGUGCUCCGAGGGCUCGGGUGGGCUGUAAGGCAGCCAUGGUCUUUUUCCAAUAUUGUGUCAUGGCUAACUUCUUCUGGCUGCUGGUGGA
>random_seq_from_cds__NO_13245 RANDOM_LENGTH 86 SEQ_LENGTH 72 GC_CONTENT 0.597222222222222 BASEPAIR 27 FREE_ENERGY -26.80 LEN_BP_RATIO 2.66666666666667
GGGCCUCUACCUGUACACCCUGCUUGCCGUCUCCUUCUUCUCUGAGCGGAAGUACUUCUGGGGGUACAUACUCAUCGGCUGGGGGG
>random_seq_from_cds__NO_13258 RANDOM_LENGTH 94 SEQ_LENGTH 68 GC_CONTENT 0.573529411764706 BASEPAIR 21 FREE_ENERGY -17.10 LEN_BP_RATIO 3.23809523809524
AUUUGCCCGCAUCCUCCUACUGACCUAGUGGCAUUCAACCUCCAGGAUCCUCAGCAUGGAUUCUCCUGCCCCUGAAGCUUCUGCCCUUUCCCAG
>random_seq_from_cds__NO_13260 RANDOM_LENGTH 81 SEQ_LENGTH 61 GC_CONTENT 0.557377049180328 BASEPAIR 21 FREE_ENERGY -25.40 LEN_BP_RATIO 2.9047619047619
GGAGGUGUCAGUAUGCUUCACUUCAGAGGAAUGGGCAUGUCUGGGCCCAAUCCAGAGGGCCUUGUACUGGGAUGUGAUGCU
>random_seq_from_cds__NO_13261 RANDOM_LENGTH 86 SEQ_LENGTH 79 GC_CONTENT 0.468354430379747 BASEPAIR 28 FREE_ENERGY -35.00 LEN_BP_RATIO 2.82142857142857
GGAGAAUUAUGGAAAUGUGACCUCCCUAGGAAUGGGAGACCAUGACCGAGAAUGAGGAGGUGACAUCAAAGCCAAGUAGUUCUCAA
>random_seq_from_cds__NO_13286 RANDOM_LENGTH 86 SEQ_LENGTH 57 GC_CONTENT 0.298245614035088 BASEPAIR 23 FREE_ENERGY -15.80 LEN_BP_RATIO 2.47826086956522
AACCCUAUGGGUGUAAUGAUUGUAGUAAAGUUUUUAGGCAAAGAAAAAACCUUACUGUACAUCAGAAAAUCCACACAGAUGAAAAA

>random_seq_from_cds__NO_13294 RANDOM_LENGTH 110 SEQ_LENGTH 95 GC_CONTENT 0.663157894736842 BASEPAIR 37 FREE_ENERGY -48.00 LEN_BP_RATIO 2.56756756756757
ACCUCCUUAGGGCGCUCAGGCUCAGGGGGAGGUGCGGGAGCUGGAGCUGCCUUGGGGGCUGCCUU
GGCAUCAUCCUUCUUGGGCUCUGGCUUUUGGGGGCCAUGCUAAG
>random_seq_from_cds__NO_13298 RANDOM_LENGTH 72 SEQ_LENGTH 67 GC_CONTENT 0.626865671641791 BASEPAIR 23 FREE_ENERGY -22.20 LEN_BP_RATIO 2.91304347826087
UGGUAUCUGACCCUGGCUGGGGUGGAUGAGGUGGUGGCAUCUGGUGGUGCAUCAGAGGGUAGGGA
GGGGGCU
>random_seq_from_cds__NO_13315 RANDOM_LENGTH 81 SEQ_LENGTH 59 GC_CONTENT 0.440677966101695 BASEPAIR 22 FREE_ENERGY -16.50 LEN_BP_RATIO 2.68181818181818
AUACUCUUGGGGGUUUAGACGAUACGUGUCAAUCAUAAAAUUUCGAUAUGCCAAGUAUCAUUUCU
GGAGUCUUGGAUUUGU
>random_seq_from_cds__NO_13339 RANDOM_LENGTH 72 SEQ_LENGTH 64 GC_CONTENT 0.6875 BASEPAIR 19 FREE_ENERGY -24.25 LEN_BP_RATIO 3.36842105263158
CAGGGACCCCAGGAGCCCCCACCACUCUCUCUCCGGGGGGACCUCGCUCACCCCUUCUGGCCCUG
AAGUCCU
>random_seq_from_cds__NO_13345 RANDOM_LENGTH 84 SEQ_LENGTH 77 GC_CONTENT 0.662337662337662 BASEPAIR 24 FREE_ENERGY -36.52 LEN_BP_RATIO 3.20833333333333
UCGGAUACCAGGCACUCCAUCCUUUCCUGGGGAUCCCCGGGUCACCAGGGAUCCCUGCUGCACCA
GGUUGACCCCUGGGGUCCC
>random_seq_from_cds__NO_13349 RANDOM_LENGTH 70 SEQ_LENGTH 57 GC_CONTENT 0.824561403508772 BASEPAIR 22 FREE_ENERGY -39.00 LEN_BP_RATIO 2.59090909090909
CGCUCUCCCCUGCUGCCAGGGGGCCCCCGUGAGUCCUCGGGGUCCCUCCUGGCCGGGGCGGCCAU
CUUCA
>random_seq_from_cds__NO_13360 RANDOM_LENGTH 90 SEQ_LENGTH 80 GC_CONTENT 0.7125 BASEPAIR 29 FREE_ENERGY -43.30 LEN_BP_RATIO 2.75862068965517
AGACCCCGGGGCACCAGGUGGUCCAGGGUCUCCAUGACCACCCCACUGGGCCAGGGGGGCCUCUU
GGACCCUGCAGACCCCGGCUUCCCU
>random_seq_from_cds__NO_13373 RANDOM_LENGTH 75 SEQ_LENGTH 68 GC_CONTENT 0.705882352941177 BASEPAIR 23 FREE_ENERGY -32.01 LEN_BP_RATIO 2.95652173913043
GGUUUGCCAUCCUCGCCUGGCUUUCCCCGGUAAUCCAGGGGGACCAGAGGGGCCAGGGAGGCCCU
GUUCUCCACG
>random_seq_from_cds__NO_13375 RANDOM_LENGTH 75 SEQ_LENGTH 61 GC_CONTENT 0.704918032786885 BASEPAIR 20 FREE_ENERGY -31.00 LEN_BP_RATIO 3.05
CAGUCCGCUCCGGCCAUCCAGCCCAGGGGGACCCCGGUCACCCCUUUUCUCCUGCUGGGCCUCGG

ACACCUGGGU
>random_seq_from_cds__NO_13384 RANDOM_LENGTH 68 SEQ_LENGTH 48
GC_CONTENT 0.75 BASEPAIR 19 FREE_ENERGY -27.13
LEN_BP_RATIO 2.52631578947368
UGGGCCUGGGGGUCCACGUUCGCCCCUUCUCUCCAGCCUCACCCAGGGGCCCUGGAAAGCCCCGG
UCA
>random_seq_from_cds__NO_13386 RANDOM_LENGTH 73 SEQ_LENGTH 61
GC_CONTENT 0.704918032786885 BASEPAIR 23 FREE_ENERGY
-29.20 LEN_BP_RATIO 2.65217391304348
CCCUUUUUCUCCUUUCUUUCCAGGGGGGCCAACGGGGCCUUGGGGUCCAGGGCUUCCGGGAAGAC
CCCGGCAG
>random_seq_from_cds__NO_13392 RANDOM_LENGTH 84 SEQ_LENGTH 75
GC_CONTENT 0.693333333333333 BASEPAIR 25 FREE_ENERGY
-30.20 LEN_BP_RATIO 3
CCGGGAGGCCAGGGUCGCCAGGAGGCCCAACUUGUCCUCUCAGGCCCCAUCUCUCCAGGUUCCCC
CUUCUGGCCCCUUUGGACA
>random_seq_from_cds__NO_13393 RANDOM_LENGTH 78 SEQ_LENGTH 66
GC_CONTENT 0.666666666666667 BASEPAIR 26 FREE_ENERGY
-35.60 LEN_BP_RATIO 2.53846153846154
AUACACUGGGCAGGGCUCUGGCCGGGGCCUGAGUAGUGAAGGAUGCCUGACACAGGGCUGUGGCC
AGACCACUGACUG
>random_seq_from_cds__NO_13415 RANDOM_LENGTH 71 SEQ_LENGTH 57
GC_CONTENT 0.701754385964912 BASEPAIR 22 FREE_ENERGY
-27.70 LEN_BP_RATIO 2.59090909090909
GAGUUUCCGGCUCCCCGGCGGACAGUGAGGACACUGGCACUGCCCUCACGGGGACCCACUCGAGC
AGACAC
>random_seq_from_cds__NO_13417 RANDOM_LENGTH 77 SEQ_LENGTH 68
GC_CONTENT 0.705882352941177 BASEPAIR 26 FREE_ENERGY
-31.00 LEN_BP_RATIO 2.61538461538462
AGGGUCCGCUCAACCCCCCUGGGUGCUGCGCACAAUGAUGCGGUACUGGGUGGCACCAGGGACUG
GGCUCCAGGACA
>random_seq_from_cds__NO_13428 RANDOM_LENGTH 85 SEQ_LENGTH 72
GC_CONTENT 0.652777777777778 BASEPAIR 21 FREE_ENERGY
-28.47 LEN_BP_RATIO 3.42857142857143
GCUUGGCUCAGACAGCACCAGGUCUCGUGGAGCAGAGGUCGAGUCAUCCGCGAGGUCGGGUCACA
GGCACGCCACCAGCAGUCGU
>random_seq_from_cds__NO_13433 RANDOM_LENGTH 84 SEQ_LENGTH 54
GC_CONTENT 0.611111111111111 BASEPAIR 19 FREE_ENERGY
-22.40 LEN_BP_RATIO 2.84210526315789
AUCACAUCACCCCCAGAGCCAAGUGCAUCCAGGCCGAACUCUGUCCCGUGGGUCAUCGCUGUACU
GCACUGUGGCAAAGCGCAC
>random_seq_from_cds__NO_13439 RANDOM_LENGTH 68 SEQ_LENGTH 56
GC_CONTENT 0.678571428571429 BASEPAIR 19 FREE_ENERGY
-25.50 LEN_BP_RATIO 2.94736842105263

ACUGUUCUGUGGAGGGGCCAUGGCUGGGGCCACUGACCGAGUCCUCCCUAGCUCUGAGCAGCUGC
GGG
>random_seq_from_cds__NO_13455 RANDOM_LENGTH 73 SEQ_LENGTH 65
GC_CONTENT 0.676923076923077 BASEPAIR 23 FREE_ENERGY
-24.50 LEN_BP_RATIO 2.82608695652174
GAGAGCCGUGUUGUUGUAGGCCCCAGGUCCCCAGCCCAGGUGCUGGCCUUGCCUCCUCAGGCGCU
GCCUUCCU
>random_seq_from_cds__NO_13474 RANDOM_LENGTH 84 SEQ_LENGTH 61
GC_CONTENT 0.672131147540984 BASEPAIR 22 FREE_ENERGY
-29.60 LEN_BP_RATIO 2.77272727272727
GAUUCCUCAUUGAAGUGGGCAUCCUGUGUGGCUGUCAGCCCGAAGCCCUGCUGAUGGCUCUCGAA
GGCCAGCAGGUGGGCCAGC
>random_seq_from_cds__NO_13477 RANDOM_LENGTH 95 SEQ_LENGTH 90
GC_CONTENT 0.666666666666667 BASEPAIR 25 FREE_ENERGY
-31.10 LEN_BP_RATIO 3.6
CCAGCCAACCCUGGGCCUCAUCACACAGCCGCACAGCAGCACCCCAGGGCCCCCGGGGACAGGG
CACUGUGGCCAGGACGCCAAACUUUGUCUG
>random_seq_from_cds__NO_13479 RANDOM_LENGTH 75 SEQ_LENGTH 67
GC_CONTENT 0.701492537313433 BASEPAIR 23 FREE_ENERGY
-29.50 LEN_BP_RATIO 2.91304347826087
CCUCUGCGAAGGGACUGUCACAGCUGUUGCACUGGCGGCCAAGGGCUCCUGGGCGACAGGGGCAC
UGCCCGCUGU
>random_seq_from_cds__NO_13500 RANDOM_LENGTH 99 SEQ_LENGTH 88
GC_CONTENT 0.75 BASEPAIR 33 FREE_ENERGY -39.70
LEN_BP_RATIO 2.66666666666667
CGAAAGCCGCCGUUGGGCGCGUCGGUGCAGGUGCCCCGUUGCGGCAGACGCCCGGCACGCAGCG
GCCGGCCUCGGUGUCCAGCUCGCAGUCCUCUCCC
>random_seq_from_cds__NO_13505 RANDOM_LENGTH 94 SEQ_LENGTH 84
GC_CONTENT 0.678571428571429 BASEPAIR 30 FREE_ENERGY
-28.70 LEN_BP_RATIO 2.8
CCCUGCAGCGCCCGCCCCGGCCCCACGUGGAGCUAGCGCCGAGAAACUCACAUUGAGCACGGUGC
CCCCUACGUCUGUGUCGUUCUGGAUGUUG
>random_seq_from_cds__NO_13508 RANDOM_LENGTH 68 SEQ_LENGTH 51
GC_CONTENT 0.666666666666667 BASEPAIR 22 FREE_ENERGY
-28.50 LEN_BP_RATIO 2.31818181818182
UGCGCCGUCACGCUGUGCAGGCCAUCCUGUGACAGUCACCAACAUGGAGGCCACCAGUGGGCGGU
UAU
>random_seq_from_cds__NO_13513 RANDOM_LENGTH 109 SEQ_LENGTH 78
GC_CONTENT 0.538461538461538 BASEPAIR 24 FREE_ENERGY
-29.35 LEN_BP_RATIO 3.25
GUUCCCCUCCACGAUCUGGUACAUUAUAUGGGCAUUGGGGCCUUCGUCAGGGUCCACUGCAGUGA
UCUGGGCCACCACUGAGCCCACAAUGCUAUUCUCUUUCACCCGC
>random_seq_from_cds__NO_13523 RANDOM_LENGTH 72 SEQ_LENGTH 56
GC_CONTENT 0.589285714285714 BASEPAIR 20 FREE_ENERGY

-23.70 LEN_BP_RATIO 2.8
CCCCCCUGGGUGCUGAUGGCAAAGCGAUUCCGGGUGUUGCCGCCUGUGAUCUGGUAGCUGAUGGC
ACUGUUG
>random_seq_from_cds__NO_13533 RANDOM_LENGTH 72 SEQ_LENGTH 54
GC_CONTENT 0.759259259259259 BASEPAIR 20 FREE_ENERGY
-28.00 LEN_BP_RATIO 2.7
CGCACAGUGGCCGAGCGCGGCCCGGGUUCCUGGCCCUGGUCGCUGGCUUCCACCACCAGCUCAUA
GCUUUCC
>random_seq_from_cds__NO_13540 RANDOM_LENGTH 78 SEQ_LENGTH 62
GC_CONTENT 0.596774193548387 BASEPAIR 24 FREE_ENERGY
-29.20 LEN_BP_RATIO 2.58333333333333
CAACCACGCGUAGCACCGCGGUGCCUGCUGCCUCAUUCUCCGGCACCAGCGUCUGGUAGUUGUAC
UGCGGAAACUGCG
>random_seq_from_cds__NO_13545 RANDOM_LENGTH 86 SEQ_LENGTH 70
GC_CONTENT 0.714285714285714 BASEPAIR 25 FREE_ENERGY
-37.90 LEN_BP_RATIO 2.8
GCGGUGCCCACUCUUUUGCGGGAGCCUGUCCCAGCGUUCCGCUGGGAGGACACCGGCUUGGGACC
GUGGUGCCGAAUCAAAAAGUC
>random_seq_from_cds__NO_13550 RANDOM_LENGTH 70 SEQ_LENGTH 58
GC_CONTENT 0.672413793103448 BASEPAIR 21 FREE_ENERGY
-21.00 LEN_BP_RATIO 2.76190476190476
CAAAGAGCUAAGGCUCCGCCACCGAUAUGCGCCCUUGGCCCCGUAGUGGCAGCUAAGCCUGGGUC
CCAGC
>random_seq_from_cds__NO_13551 RANDOM_LENGTH 84 SEQ_LENGTH 74
GC_CONTENT 0.675675675675676 BASEPAIR 25 FREE_ENERGY
-32.10 LEN_BP_RATIO 2.96
CCUGGUGCCCACCGCCCCCCAGCUCCUCCUGGCUGAGGGGGAACAAAGAGAGGAGAAGGAGCAGG
AGUAUGGGGGUCGACCGUC
>random_seq_from_cds__NO_13567 RANDOM_LENGTH 75 SEQ_LENGTH 69
GC_CONTENT 0.652173913043478 BASEPAIR 23 FREE_ENERGY
-25.40 LEN_BP_RATIO 3
GGCAUCGGGCCCUGGCGGGGUAGUAGCUUGCCUAGAGAUCUCAGCCGCACCCUCUGGCAAGGCCA
UCUUUGUCAA
>random_seq_from_cds__NO_13574 RANDOM_LENGTH 71 SEQ_LENGTH 54
GC_CONTENT 0.62962962962963 BASEPAIR 23 FREE_ENERGY
-27.30 LEN_BP_RATIO 2.34782608695652
UGGGGAUGUCAUGCUGUACAGGGCUCUGGGUGGCUGCACCCGGCCACACGUGAUUCUCCGGGAGG
GUCUGC
>random_seq_from_cds__NO_13577 RANDOM_LENGTH 71 SEQ_LENGTH 50
GC_CONTENT 0.6 BASEPAIR 20 FREE_ENERGY -27.90 LEN_BP_RATIO
2.5
CACAGGCUCAGCCCACGCACUCACAGCUGUUUGUAACCAUAUCUCCUCGGUACGUGCUGUGGCUG
UGUGGG
>random_seq_from_cds__NO_13583 RANDOM_LENGTH 110 SEQ_LENGTH 87

GC_CONTENT 0.620689655172414 BASEPAIR 29 FREE_ENERGY -33.00 LEN_BP_RATIO 3
CAGCCUGGCUUUCUGGGAUCUCACCACCAUGCUAGACCAUGACUCCACUGUCCUGGAGCCUCCAGUGGAUCCUGGGCUUCCCUACCGGGCUUGGCACCCCCUCCCUGACU
>random_seq_from_cds__NO_13589 RANDOM_LENGTH 72 SEQ_LENGTH 50 GC_CONTENT 0.6 BASEPAIR 21 FREE_ENERGY -24.10 LEN_BP_RATIO 2.38095238095238
CCUGUGAGCCCUGAGUUUGGCCACCGUUGUGCCCUUGGGGGUCAGGGGCUUGAGGUUUACAACUGGUAUGAC
>random_seq_from_cds__NO_13597 RANDOM_LENGTH 83 SEQ_LENGTH 64 GC_CONTENT 0.5625 BASEPAIR 24 FREE_ENERGY -23.70 LEN_BP_RATIO 2.66666666666667
AGCAGGAAAGUUGGUGAUGAUGACCCGUAGUGACUCCAGCACAGCCAUGGCUCGUGGGGCUGUGUCAUUCAGCACAUCACGCA
>random_seq_from_cds__NO_13608 RANDOM_LENGTH 69 SEQ_LENGTH 62 GC_CONTENT 0.516129032258065 BASEPAIR 22 FREE_ENERGY -23.50 LEN_BP_RATIO 2.81818181818182
AGGUGCUGCUUUAGUAGAUUCAUGGUGUGUGGAGUGACCACAUAGCCUGGGGUCUUGUAGUUCUCACCC
>random_seq_from_cds__NO_13614 RANDOM_LENGTH 98 SEQ_LENGTH 86 GC_CONTENT 0.593023255813954 BASEPAIR 32 FREE_ENERGY -33.40 LEN_BP_RATIO 2.6875
GCCGCUUAGCUGGGGCUCAGUGUGGAUCUUCUUACUGGCUAUGUAGCUUACAAGGAAGGAGAGACGCCGGGUAUCCCUGAGUCGGGAGGCCAAGCCAU
>random_seq_from_cds__NO_13616 RANDOM_LENGTH 80 SEQ_LENGTH 73 GC_CONTENT 0.657534246575342 BASEPAIR 27 FREE_ENERGY -34.00 LEN_BP_RATIO 2.7037037037037
GCUGCGCGCUCAGAGCCGAGUUCUUGAGCGUCUCGCGGGCCUUCUGCUCGCUCAGGCCGAGGCUAGUGAAGAGCGACAGG
>random_seq_from_cds__NO_13620 RANDOM_LENGTH 71 SEQ_LENGTH 59 GC_CONTENT 0.661016949152542 BASEPAIR 19 FREE_ENERGY -21.72 LEN_BP_RATIO 3.10526315789474
CUGGGCACGACCCUGGGCACUGCCUGCCGUUUCUUCUGCUGUAGAGGCUGCCAGACUAUUUCCUGCCCGUU
>random_seq_from_cds__NO_13622 RANDOM_LENGTH 85 SEQ_LENGTH 71 GC_CONTENT 0.661971830985916 BASEPAIR 28 FREE_ENERGY -34.50 LEN_BP_RATIO 2.53571428571429
GUACCCUGGUACAGGGUCUGCUCUGUGUCCCGUGUGUCAGCCACUGCCCCCCGGAUGGCACCCUGGGCAAUACCCUGUGCCCGCU
>random_seq_from_cds__NO_13627 RANDOM_LENGTH 82 SEQ_LENGTH 62 GC_CONTENT 0.67741935483871 BASEPAIR 20 FREE_ENERGY -22.00 LEN_BP_RATIO 3.1
GCUGCCUGGGCCCGCUGCUGUGCCUCGCUUGCCUGCCGACGAGUCUCAGCCACUCUGCUGAGGAUGCUACCACCUUCUGCCA

>random_seq_from_cds__NO_13629 RANDOM_LENGTH 95 SEQ_LENGTH 88 GC_CONTENT 0.636363636363636 BASEPAIR 27 FREE_ENERGY -27.91 LEN_BP_RATIO 3.25925925925926
UCUCGACAGCCGGCACCCCCACAAGGGCUUGUAGCACAGGGUGCAUCCCCUGGUGCCCCACACAC
CCAGCUCAUUUAUGUCUGUCAGGCUCAGGG
>random_seq_from_cds__NO_13634 RANDOM_LENGTH 110 SEQ_LENGTH 103 GC_CONTENT 0.650485436893204 BASEPAIR 35 FREE_ENERGY -53.83 LEN_BP_RATIO 2.94285714285714
UGCUCAGUGGCCUCCCCAAUUUCACGCCCGCAGCUCCUCUGUGGCCUCCACAAGCUGUGCAGUGG
AGGCGGCUGAGGUGUUGCGGGCACCUACGAUGCCCUGCACAAUGC
>random_seq_from_cds__NO_13640 RANDOM_LENGTH 75 SEQ_LENGTH 67 GC_CONTENT 0.656716417910448 BASEPAIR 23 FREE_ENERGY -29.20 LEN_BP_RATIO 2.91304347826087
GCCACUGGUGAGGUUCCAGAAGUUGGGGGCACAGCGGUCACAGCUAGGGCCCUGGACAUUGGGGA
GGCAUGGGCA
>random_seq_from_cds__NO_13642 RANDOM_LENGTH 88 SEQ_LENGTH 77 GC_CONTENT 0.649350649350649 BASEPAIR 26 FREE_ENERGY -36.20 LEN_BP_RATIO 2.96153846153846
UCGGGCAGCCUGCCCAUGGAAGCCAGGCUUGCAGUGGGCACAGUGUGGACCCUCUGUGUGGUGUA
AACAGCGCAGGCAUUGCCCCGUG
>random_seq_from_cds__NO_13649 RANDOM_LENGTH 110 SEQ_LENGTH 97 GC_CONTENT 0.639175257731959 BASEPAIR 33 FREE_ENERGY -38.61 LEN_BP_RATIO 2.93939393939394
CACUGGCAGGCCUUGACAGCCUGUGGGGCCAAAGCCAUAGUAGCCAGGGGCACAGAGGUCACAGC
GGCGCCCAACCACUCCAGGCUUGCACAGGCACUGACCACCAUGAG
>random_seq_from_cds__NO_13655 RANDOM_LENGTH 84 SEQ_LENGTH 64 GC_CONTENT 0.609375 BASEPAIR 21 FREE_ENERGY -23.70 LEN_BP_RATIO 3.04761904761905
UCAGUUCCAACUCUGCCCAUUGCUCAGGGACCCUGGGGCUCUAAGCGCAGCAGCAGGUCAUAGUC
CAUAGCCUUCGGCACAGAG
>random_seq_from_cds__NO_13656 RANDOM_LENGTH 67 SEQ_LENGTH 60 GC_CONTENT 0.616666666666667 BASEPAIR 21 FREE_ENERGY -18.10 LEN_BP_RATIO 2.85714285714286
GCCACCAGGAACUCCAGGGUCUGACCUUCCUGUAGCCGCACGAAGCCUGAGCCAGUCCAGGAUGG
AG
>random_seq_from_cds__NO_13667 RANDOM_LENGTH 80 SEQ_LENGTH 67 GC_CONTENT 0.686567164179104 BASEPAIR 25 FREE_ENERGY -37.70 LEN_BP_RATIO 2.68
GAUGCAAGCUCCGUGCACCCAUGCCCUCAGCAUGGGCUGGUGCCCCUGGGGCGGGUGCACACUCU
GAGGCGUGUCCGUAG
>random_seq_from_cds__NO_13670 RANDOM_LENGTH 84 SEQ_LENGTH 69 GC_CONTENT 0.594202898550725 BASEPAIR 25 FREE_ENERGY -25.40 LEN_BP_RATIO 2.76
GAGCUGUAGGGGUCUGGGAUAGGGAUGGCAGGGUCCAGCACACGAUAGAUGACCCUCGCCUUCAG

UGGAUGGCUCAAUCUCUGA
>random_seq_from_cds__NO_13674 RANDOM_LENGTH 75 SEQ_LENGTH 68 GC_CONTENT 0.588235294117647 BASEPAIR 26 FREE_ENERGY -22.00 LEN_BP_RATIO 2.61538461538462
CCAUUCUCUGACUGCCACCAGGCUGCCCGCCGCUGUGGUGCAAAGCUGGUGACUACAUUCUGGAUGCGAUGGCUG
>random_seq_from_cds__NO_13682 RANDOM_LENGTH 75 SEQ_LENGTH 60 GC_CONTENT 0.666666666666667 BASEPAIR 23 FREE_ENERGY -28.90 LEN_BP_RATIO 2.60869565217391
CAGCGGCAGGGAAGGAGGCUUUGCCAUCUUGGCUGCACUGGGACUCACAGAGCCACACCCUGGAGGGCCUCCCCC
>random_seq_from_cds__NO_13687 RANDOM_LENGTH 110 SEQ_LENGTH 87 GC_CONTENT 0.597701149425287 BASEPAIR 31 FREE_ENERGY -32.70 LEN_BP_RATIO 2.80645161290323
GGAAAUGCAAAAAAGGUGGUGGAGAAUGGGGCCCUUCUCUCCUGGAAGCUGGGCUGCUCCCUGAACCAGAACAGUGUGCCUGACAUUCAUGGUGUAGAGGCCCCUGCCAG
>random_seq_from_cds__NO_13688 RANDOM_LENGTH 70 SEQ_LENGTH 63 GC_CONTENT 0.619047619047619 BASEPAIR 20 FREE_ENERGY -23.80 LEN_BP_RATIO 3.15
GGAGGGCGCAAUGUCUGCUCAGCUUGGCUACCCUGUGGUGGGUUGGCACAUCGCCAAUAAGAAGCCCCCU
>random_seq_from_cds__NO_13692 RANDOM_LENGTH 89 SEQ_LENGTH 63 GC_CONTENT 0.587301587301587 BASEPAIR 20 FREE_ENERGY -23.10 LEN_BP_RATIO 3.15
GAAGCUGGCACCACAGUUCCUGGCCAGAUUCGCCCAACGAUGACCAUUCCUGGCUAUGUGGAGCCUACUGCAGUUGCUACCCCUCCCAC
>random_seq_from_cds__NO_13695 RANDOM_LENGTH 88 SEQ_LENGTH 70 GC_CONTENT 0.614285714285714 BASEPAIR 23 FREE_ENERGY -21.74 LEN_BP_RATIO 3.04347826086957
AUCACCAGAUUGGAAACUGCCUCACCGCCUACUCGUAUUCGCACCACCACCAGUGGAGUGCCCCGUGGCGGAGAACCCAACCAGCGCC
>random_seq_from_cds__NO_13700 RANDOM_LENGTH 85 SEQ_LENGTH 72 GC_CONTENT 0.583333333333333 BASEPAIR 24 FREE_ENERGY -25.80 LEN_BP_RATIO 3
CUGGCCUUCGCCUUUGGAGACCGAAACUGUAGCACCAUCACCCUGCAGAAUAUCACCCGGGGCUCCAUCGUGGUGGAAUGGACCA
>random_seq_from_cds__NO_13702 RANDOM_LENGTH 86 SEQ_LENGTH 76 GC_CONTENT 0.618421052631579 BASEPAIR 25 FREE_ENERGY -27.40 LEN_BP_RATIO 3.04
UCGGCCUGCCUUCUCCAACGCCCUAGAGCCUGACUUUAAGGCCACAAGCAUCACUGUGACGGGCUCUGGCAGUUGUCGGCACCUAC
>random_seq_from_cds__NO_13703 RANDOM_LENGTH 72 SEQ_LENGTH 58 GC_CONTENT 0.672413793103448 BASEPAIR 20 FREE_ENERGY -27.22 LEN_BP_RATIO 2.9

AGUUUAUCCCUGUGGUACCACCCAGGAGAGUGCCCUCAGAGGCGCCGCCCACAGAAGUGCCUGAC
AGGGACC
>random_seq_from_cds__NO_13704 RANDOM_LENGTH 81 SEQ_LENGTH 67
GC_CONTENT 0.582089552238806 BASEPAIR 27 FREE_ENERGY
-28.70 LEN_BP_RATIO 2.48148148148148
CUGAGAAGAGCAGUGAGGAUGAUGUCUACCUGCACACAGUCAUUCCGGCCGUGGUGGUCGCAGCC
AUCCUGCUCAUUGCUG
>random_seq_from_cds__NO_13706 RANDOM_LENGTH 110 SEQ_LENGTH 88
GC_CONTENT 0.636363636363636 BASEPAIR 24 FREE_ENERGY
-24.13 LEN_BP_RATIO 3.66666666666667
AUCUUUGCAGACGAACUGGACGACUCCAAGCCCCCACCCUCCUCCAGCAUGCCACUCAUUCUGCA
GGAGGAGAAGGCUCCCCUACCCCCUCCUGAGUACCCCAACCAGAG
>random_seq_from_cds__NO_13715 RANDOM_LENGTH 89 SEQ_LENGTH 74
GC_CONTENT 0.567567567567568 BASEPAIR 24 FREE_ENERGY
-23.00 LEN_BP_RATIO 3.08333333333333
ACACACUUGUUGUAUGUGGCAGAGAAGAAGCGCCCCAAGGCCGAGUCCUUCUUUCAGACCAAAGC
CUUGGACGUCAGUGCCAGCGAUGA
>random_seq_from_cds__NO_13722 RANDOM_LENGTH 82 SEQ_LENGTH 68
GC_CONTENT 0.544117647058823 BASEPAIR 25 FREE_ENERGY
-25.00 LEN_BP_RATIO 2.72
GAGAACUUCUCUGGGAUCUACUGCAGCCUUCUGCCUUUGGGAUGCUGGUCAGCUGACAGCCAGAG
AGUGGUCUUUGACUCGG
>random_seq_from_cds__NO_13726 RANDOM_LENGTH 85 SEQ_LENGTH 57
GC_CONTENT 0.56140350877193 BASEPAIR 19 FREE_ENERGY
-18.60 LEN_BP_RATIO 3
ACAGCCACCCCCAGAGCAAGAGAAUGUGCAGUAUGGCUGGCCUUGACUUUGAAGCAAUCCUGCUG
CAGCCUGGCAGCCCUCCAGA
>random_seq_from_cds__NO_13730 RANDOM_LENGTH 78 SEQ_LENGTH 64
GC_CONTENT 0.5625 BASEPAIR 24 FREE_ENERGY -29.40
LEN_BP_RATIO 2.66666666666667
CAUGUGGCCCUUAUGGGUGGUUCCCAUGGUGGCUUCAUUUCCUGCCACUUGAUUGGUCAGUACCC
AGAGACCUACAGG
>random_seq_from_cds__NO_13734 RANDOM_LENGTH 84 SEQ_LENGTH 62
GC_CONTENT 0.548387096774194 BASEPAIR 21 FREE_ENERGY
-22.40 LEN_BP_RATIO 2.95238095238095
AGACCCGGAAUGUGCCUGUUCGGGCUCCUGCUCUAUCCCAAAAGCACCCACGCAUUAUCAGAGGU
GGAGGUGGAGUCAGACAGC
>random_seq_from_cds__NO_13747 RANDOM_LENGTH 68 SEQ_LENGTH 51
GC_CONTENT 0.588235294117647 BASEPAIR 20 FREE_ENERGY
-21.90 LEN_BP_RATIO 2.55
CCACGGUUGGCUAUGAUGCCCAGAGCAUUGAGUGGCAGGUGCACACCAAAGCGGGCCUCACAGUG
UGC
>random_seq_from_cds__NO_13750 RANDOM_LENGTH 81 SEQ_LENGTH 61
GC_CONTENT 0.59016393442623 BASEPAIR 22 FREE_ENERGY

-16.70 LEN_BP_RATIO 2.77272727272727
GGUGAUCCAGCUUCACGAUGCCCCUGAGGAAUCAGAUUCUUAUGCACUCGCCUCAGCUUGGCCCG
CUUCUGCCCGUUCUUG
>random_seq_from_cds__NO_13754 RANDOM_LENGTH 99 SEQ_LENGTH 70
GC_CONTENT 0.8 BASEPAIR 20 FREE_ENERGY -23.30 LEN_BP_RATIO
3.5
CGGCCGGGACUUGGAGGCGGUGCGGCGCGGCGGGUGCGGUUCAGUCGGUCGGCGGCGGCAGCGGA
GGAGGAGGAGGAGGAGGAGGAGGAGGAGGAUGAG
>random_seq_from_cds__NO_13760 RANDOM_LENGTH 99 SEQ_LENGTH 87
GC_CONTENT 0.459770114942529 BASEPAIR 25 FREE_ENERGY
-20.74 LEN_BP_RATIO 3.48
AGAGCUGAAGAAGAUUCUGGAUGACUGGGGGGAGACAUGCAAAGGCUGUGCAGAAAAGUCUGACU
ACAUCCGGAAGAUAAAUGAACUGAUGCCUAAAUA
>random_seq_from_cds__NO_13771 RANDOM_LENGTH 94 SEQ_LENGTH 67
GC_CONTENT 0.671641791044776 BASEPAIR 27 FREE_ENERGY
-36.40 LEN_BP_RATIO 2.48148148148148
GGGGGCCCUGGAGAGUGUGGUGGCAGGCAGUGAGGGGGCUGCUGAGGGUGCUAUCACCAUCGAGC
UGGCCUCCUACCCCAUCAGUGACUUUGCC
>random_seq_from_cds__NO_13775 RANDOM_LENGTH 89 SEQ_LENGTH 82
GC_CONTENT 0.597560975609756 BASEPAIR 25 FREE_ENERGY
-22.50 LEN_BP_RATIO 3.28
AUGCGGCCAGUUAACGGGCGCCGCCUCUACAAGGACUUUGUGCUCAACGUCAAGUUUGAUGGCCC
CCUUUCGCCCAGCUGACACCCACA
>random_seq_from_cds__NO_13776 RANDOM_LENGTH 81 SEQ_LENGTH 66
GC_CONTENT 0.545454545454545 BASEPAIR 24 FREE_ENERGY
-27.50 LEN_BP_RATIO 2.75
AUGAGGUCCGCUUUGACCGCUUUGGUGAUGGUAUUGGCCGCUACAACAUCUUCACCUAUCUGCGU
GCAGGCAGUGGGCGCU
>random_seq_from_cds__NO_13784 RANDOM_LENGTH 80 SEQ_LENGTH 73
GC_CONTENT 0.63013698630137 BASEPAIR 24 FREE_ENERGY
-28.40 LEN_BP_RATIO 3.04166666666667
CGGCCACGCUUCAUCAGUCCUGCCUCACAGGUGGCCAUCUGCCUGGCACUUAUCUCGGGCCAGCU
GCUCAUCGUGGUCGC
>random_seq_from_cds__NO_13793 RANDOM_LENGTH 99 SEQ_LENGTH 88
GC_CONTENT 0.568181818181818 BASEPAIR 31 FREE_ENERGY
-34.60 LEN_BP_RATIO 2.83870967741936
GCCUGGGCUGUCAGAAAGUAGAGGGUGGCACCUGGCUAUGUGGUGACCGUGUUGACCUGGCCAGG
CACCAACCCUACACUCUCCUCCAUCUUGCUCAAC
>random_seq_from_cds__NO_13796 RANDOM_LENGTH 68 SEQ_LENGTH 57
GC_CONTENT 0.56140350877193 BASEPAIR 20 FREE_ENERGY
-23.40 LEN_BP_RATIO 2.85
CCAGAACCAUCCACAUGACCUUUGUGCCUGGAUGAGGAGGUUGGGGGUCACCAAGGCAUGGAGCU
GUU
>random_seq_from_cds__NO_13798 RANDOM_LENGTH 81 SEQ_LENGTH 64

GC_CONTENT 0.6875 BASEPAIR 24 FREE_ENERGY -27.20
LEN_BP_RATIO 2.66666666666667
CUGUCUUUUAUAGUGAGCGGAGUCCCUGGUGGGGUGCGGGUUACCAGCACUGGGAGGCCAGGCCA
UGCCUCACGCUUCAUG
>random_seq_from_cds__NO_13801 RANDOM_LENGTH 86 SEQ_LENGTH 77
GC_CONTENT 0.584415584415584 BASEPAIR 26 FREE_ENERGY
-34.00 LEN_BP_RATIO 2.96153846153846
CAGCUUUGACUUCCGUGUGGCACCGGAUGUGGACUUCAAGGGCUUUUGAGGAGCAGCUGCAGAGC
UGGUGCCAGGCAGCUGGCGAG
>random_seq_from_cds__NO_13804 RANDOM_LENGTH 71 SEQ_LENGTH 47
GC_CONTENT 0.617021276595745 BASEPAIR 20 FREE_ENERGY
-18.80 LEN_BP_RATIO 2.35
CUCACCCAUGAACCGCACACCUGUGCUGCUGCACGACCACGAUGAACGGCUGCAUGAGGCUGUGU
UCCUCC
>random_seq_from_cds__NO_13807 RANDOM_LENGTH 87 SEQ_LENGTH 69
GC_CONTENT 0.652173913043478 BASEPAIR 24 FREE_ENERGY
-30.40 LEN_BP_RATIO 2.875
UUUGGCCGGCACAGCCUGAGCCCCUUGGCAAUACGGGCACGAGCACGCUUCCCAAGCUUGGGGUG
GGCAAUGUAGGCAAGUCGAUCG
>random_seq_from_cds__NO_13811 RANDOM_LENGTH 81 SEQ_LENGTH 71
GC_CONTENT 0.647887323943662 BASEPAIR 22 FREE_ENERGY
-27.30 LEN_BP_RATIO 3.22727272727273
AGAAGUUCCGGUUAUAGAAGUGGUGGUUGUCCCUGGUCAGGGCCAUGCCCAGCUGGGCCCAGAAG
CUGCGCUGACCACUGG
>random_seq_from_cds__NO_13814 RANDOM_LENGTH 67 SEQ_LENGTH 53
GC_CONTENT 0.69811320754717 BASEPAIR 19 FREE_ENERGY
-22.70 LEN_BP_RATIO 2.78947368421053
CACUGACCCGGUCCGUGUGGGCCAGCACAAACAGCGUCUUGCGGCUGCCAUAGACCGAGGCCCAC
AG
>random_seq_from_cds__NO_13819 RANDOM_LENGTH 72 SEQ_LENGTH 63
GC_CONTENT 0.666666666666667 BASEPAIR 24 FREE_ENERGY
-24.80 LEN_BP_RATIO 2.625
GGUCCUGUGCAAAGAUGCUGAGGCCCUGGAGCUGGCCCGGACUGCCACACUUCACCCGGCUGGGC
AGACCGG
>random_seq_from_cds__NO_13835 RANDOM_LENGTH 84 SEQ_LENGTH 72
GC_CONTENT 0.486111111111111 BASEPAIR 21 FREE_ENERGY
-19.30 LEN_BP_RATIO 3.42857142857143
AUUGAAGGACAGGUUAAGCUUGCGCAGCUGUGUUAGGCCCUGGAAGGCCUUGGUUUUAGUGAUGC
AUUUGUAGAGGAAGUUCUC
>random_seq_from_cds__NO_13837 RANDOM_LENGTH 78 SEQ_LENGTH 63
GC_CONTENT 0.634920634920635 BASEPAIR 21 FREE_ENERGY
-19.00 LEN_BP_RATIO 3
CGGCUCAGGUGGCUGAAGGUAUCGGGAUGUAGCUGGGGGAAGUGACGAGGGCACUCCAUGCAGGG
GUUGGGAGCGUGG

>random_seq_from_cds__NO_13840 RANDOM_LENGTH 62 SEQ_LENGTH 56 GC_CONTENT 0.714285714285714 BASEPAIR 21 FREE_ENERGY -27.50 LEN_BP_RATIO 2.66666666666667
GAGGUUGCCCAGGCCAAGGAGGGCACCCGGGGCCACCUCCAGUGCCUGCCUGCAGGGGUUCU
>random_seq_from_cds__NO_13842 RANDOM_LENGTH 71 SEQ_LENGTH 58 GC_CONTENT 0.517241379310345 BASEPAIR 21 FREE_ENERGY -20.20 LEN_BP_RATIO 2.76190476190476
AUGGCUGAGGGACAGGGAUAUGAGGGAUUUGGGCAGCGCAGGCACAGUCAUGAUGUUGUUGAGC UCAGGU
>random_seq_from_cds__NO_13844 RANDOM_LENGTH 74 SEQ_LENGTH 66 GC_CONTENT 0.575757575757576 BASEPAIR 23 FREE_ENERGY -21.60 LEN_BP_RATIO 2.8695652173913
GGGCAGUUCCACUUGAGGUUGAGAUGCCGCAGGCUGGGCAGGUGGGCAAAGUCAGAAUCAUGGAG GUGGUGGAU
>random_seq_from_cds__NO_13862 RANDOM_LENGTH 88 SEQ_LENGTH 82 GC_CONTENT 0.634146341463415 BASEPAIR 29 FREE_ENERGY -33.90 LEN_BP_RATIO 2.82758620689655
CCUGUGUGGUUGCCCUCAGAGGCUGCAGGGGCCCUGUUUGCUUCCAGCACCAGCGGGGACUUGUU GCUGGCUGACUUGGACUCCUCAG
>random_seq_from_cds__NO_13865 RANDOM_LENGTH 86 SEQ_LENGTH 68 GC_CONTENT 0.676470588235294 BASEPAIR 22 FREE_ENERGY -27.86 LEN_BP_RATIO 3.09090909090909
GCGAGGCCGAUACGCUCCAUGAUGACCCGCCGGGCCUUGUCUGUCCACUCCUCGUCCUCCCCCA GGGCCCCAUGGUCAAUGGGGU
>random_seq_from_cds__NO_13892 RANDOM_LENGTH 97 SEQ_LENGTH 72 GC_CONTENT 0.388888888888889 BASEPAIR 25 FREE_ENERGY -23.00 LEN_BP_RATIO 2.88
AGGUGUAUUUGAAGAAGAUGCCACAGCUAUUUCCAACUAUAUGAACCAGUUGUAUCAAGCUAUGC AUCGGAUUUAUGAUGCACAGGAAUGAAUUAAG
>random_seq_from_cds__NO_13894 RANDOM_LENGTH 99 SEQ_LENGTH 76 GC_CONTENT 0.407894736842105 BASEPAIR 22 FREE_ENERGY -17.54 LEN_BP_RATIO 3.45454545454545
CUACAUUGCAACAGUUUUCAAAAGUUAUAGAUGAGGCUUAGCUCUUGUCAUGCAGUGCUUUCAAC UCAACUUGCUGAUGCCAUGAUGUUCCCCAUUACC
>random_seq_from_cds__NO_13901 RANDOM_LENGTH 84 SEQ_LENGTH 70 GC_CONTENT 0.528571428571429 BASEPAIR 25 FREE_ENERGY -18.61 LEN_BP_RATIO 2.8
AGUAAAACAGGCUUGGUGUCAUCUACCUGGGACAGACAGUUUUACUUCACGCAGGGUGGAAAUUU AAUGAGUCAGGCCCGUGGG
>random_seq_from_cds__NO_13902 RANDOM_LENGTH 68 SEQ_LENGTH 57 GC_CONTENT 0.543859649122807 BASEPAIR 21 FREE_ENERGY -19.50 LEN_BP_RATIO 2.71428571428571
GAUGUAGCAGGAGGCCUGGCCAUGGACAUAGACAACUGUUCAGUGAUGGCUGUGGACUGUGAAGA CAG

>random_seq_from_cds__NO_13912 RANDOM_LENGTH 110 SEQ_LENGTH 97 GC_CONTENT 0.391752577319588 BASEPAIR 35 FREE_ENERGY -25.10 LEN_BP_RATIO 2.77142857142857
GGGAGAAGUGAAAGUAAUCUGUCAUCAGUCUGCUAUAUAUUUGAGUCAAACAAUGAGGGGGAAAA
GGAUAUGUGAUUCUGUUGGACUGGCAAAACAGAUAGCUUUGCAUG
>random_seq_from_cds__NO_13918 RANDOM_LENGTH 110 SEQ_LENGTH 98 GC_CONTENT 0.418367346938776 BASEPAIR 31 FREE_ENERGY -20.90 LEN_BP_RATIO 3.16129032258065
UUUAGGCGGCACAAAUGUUUUAGGGAGCGAGGGUUUGCUUAAGAUAAAAUGUAUUUCUGACCAGA
UCCCCUGUUUUUGGAGCACAGCUUUCAACUUUGAACAGAUCCGAA
>random_seq_from_cds__NO_13928 RANDOM_LENGTH 119 SEQ_LENGTH 106 GC_CONTENT 0.433962264150943 BASEPAIR 30 FREE_ENERGY -25.73 LEN_BP_RATIO 3.53333333333333
GGCAAAUCCUGUUUGUUUGCAAAAAGUAGCAGCACUGCAUCUCUCAAUUCAUCUACCAGAAGCCA
UUUUCUGCAGCUCAUCUGCUACUUCCUGAAUUCUUUCACGAUCGUUGCUAUCUA
>random_seq_from_cds__NO_13934 RANDOM_LENGTH 84 SEQ_LENGTH 70 GC_CONTENT 0.628571428571429 BASEPAIR 23 FREE_ENERGY -26.70 LEN_BP_RATIO 3.04347826086957
UAACAGGAUGGCAUCCUUCCCGGAUCAGGCGGAGCAGGUCCAGGUAGGCUGUUCUUGCCAUGUCC
ACUUGGGCACCAGACAGGA
>random_seq_from_cds__NO_13937 RANDOM_LENGTH 90 SEQ_LENGTH 83 GC_CONTENT 0.602409638554217 BASEPAIR 27 FREE_ENERGY -33.80 LEN_BP_RATIO 3.07407407407407
UAGCAGUGCACCCUUAGCAGCCUGGAGGUGUAUGACAGUGGUCUGGUUCCAAGCCUCGUGCUGGU
CAGCUCCGGAUUGCGUCAGGGUCUG
>random_seq_from_cds__NO_13943 RANDOM_LENGTH 71 SEQ_LENGTH 58 GC_CONTENT 0.5 BASEPAIR 20 FREE_ENERGY -19.60 LEN_BP_RATIO 2.9
GAAGUCUUGGUUCAGAAUGGCAGUGUAGGAGUGCUGGAAGAACUCCAAGAGGCUGACUGCCAGGA
AAUGGA
>random_seq_from_cds__NO_13957 RANDOM_LENGTH 64 SEQ_LENGTH 57 GC_CONTENT 0.596491228070175 BASEPAIR 20 FREE_ENERGY -18.10 LEN_BP_RATIO 2.85
CCAGGUAUCCCCCAAUGACACUCGGUGCACUGGGCUGCCCAUCAUGCGGAGGUUACUGGAACCG
>random_seq_from_cds__NO_13967 RANDOM_LENGTH 86 SEQ_LENGTH 55 GC_CONTENT 0.527272727272727 BASEPAIR 23 FREE_ENERGY -20.60 LEN_BP_RATIO 2.39130434782609
CUUACCAUCAGGCUUGAGGCUUUUUAUUCCAUCUUCACCACGGAGCAGCAAGACCAUGUCAAGUC
GGUGGAGUAUCUGAGAAAUAA
>random_seq_from_cds__NO_13975 RANDOM_LENGTH 89 SEQ_LENGTH 79 GC_CONTENT 0.746835443037975 BASEPAIR 28 FREE_ENERGY -34.00 LEN_BP_RATIO 2.82142857142857
GCUCGCCGGCCUGGGGUUCGGCGGUGGUGGCAUCUCCUCUUUCCCCAGCACUGUGUGGCCCACGC
GCCUCCCGACGGCCGCCUCAGCCA

>random_seq_from_cds__NO_14006 RANDOM_LENGTH 84 SEQ_LENGTH 59 GC_CONTENT 0.491525423728814 BASEPAIR 19 FREE_ENERGY -18.70 LEN_BP_RATIO 3.10526315789474
GAAAAUGGAAAUGGUCCCAUGACAGUAGACAAAAAUGGUGCUGUUCUUAUUGCAGAUGAAUCAGA
CCCUGCUGAGAGCAUGGAG
>random_seq_from_cds__NO_14008 RANDOM_LENGTH 110 SEQ_LENGTH 104 GC_CONTENT 0.836538461538462 BASEPAIR 37 FREE_ENERGY -60.20 LEN_BP_RATIO 2.81081081081081
GGCGGCGGGCGGAGCAGCGGCCGCGGCCGCCCGGCAGCAGCAGCAGCAGCAGCAGCAGCAGCAGC
CGCCGCCUCCGCAGCCCCAGCGGCAGCAGCACCCGCCACCGCCGC
>random_seq_from_cds__NO_14009 RANDOM_LENGTH 69 SEQ_LENGTH 63 GC_CONTENT 0.825396825396825 BASEPAIR 24 FREE_ENERGY -32.80 LEN_BP_RATIO 2.625
CACGGCGCACACGGCCGGAGGACGGCGGGCCCGGCGCCGCCUCCACCUCGGCCGCCGCAAUGGCG
ACGG
>random_seq_from_cds__NO_14010 RANDOM_LENGTH 77 SEQ_LENGTH 65 GC_CONTENT 0.646153846153846 BASEPAIR 21 FREE_ENERGY -25.74 LEN_BP_RATIO 3.0952380952381
UCGGGGAGCGCAGGCCUCUGCCCAGUCCUGAAGUGAUGCUGGGACAGUCGUGGAAUCUGUGGGUU
GAGGCUUCCAAA
>random_seq_from_cds__NO_14017 RANDOM_LENGTH 90 SEQ_LENGTH 54 GC_CONTENT 0.611111111111111 BASEPAIR 19 FREE_ENERGY -18.40 LEN_BP_RATIO 2.84210526315789
UCUGCGGUGGGGCCAACCUGUCCUGCUACUGUGAGUUCCUUAGUCAAGCCUGGCCUUAACUGCCC
CUCAAUACCAAAGCCAACCUUGCCU
>random_seq_from_cds__NO_14023 RANDOM_LENGTH 69 SEQ_LENGTH 53 GC_CONTENT 0.679245283018868 BASEPAIR 21 FREE_ENERGY -22.60 LEN_BP_RATIO 2.52380952380952
CUCCACACCCUCCCCUGCCUGCCACUGAGCCAGCUUCUCGGUUAUCCAGUGAGGAGGGCGAAGGC
GAUG
>random_seq_from_cds__NO_14044 RANDOM_LENGTH 85 SEQ_LENGTH 72 GC_CONTENT 0.416666666666667 BASEPAIR 27 FREE_ENERGY -17.70 LEN_BP_RATIO 2.66666666666667
GAGGGUAACUGUGUUUCCCGCUUCCUUGAUUAGGUUCACAAUGUCUGAAUGGGAUUUGUUGGUGA
UGGAACAUCCAUUUACUGCC
>random_seq_from_cds__NO_14048 RANDOM_LENGTH 86 SEQ_LENGTH 68 GC_CONTENT 0.632352941176471 BASEPAIR 27 FREE_ENERGY -25.50 LEN_BP_RATIO 2.51851851851852
CACCGUGUUCAGCGAGUUCUGGCUGCCCUGCGGAGUGCGCUUCUCUUCUGUCAGCGAGGCCGGCU
GGUUGCUACUGUGAUGAGAGG
>random_seq_from_cds__NO_14056 RANDOM_LENGTH 81 SEQ_LENGTH 70 GC_CONTENT 0.557142857142857 BASEPAIR 22 FREE_ENERGY -22.72 LEN_BP_RATIO 3.18181818181818
UGAGAACUAUUCUGGCUGUCUUUCCUUUCCAGUGGUUGCCGACUUUGGGCUCUUCUUGGGAACUG

GCAGCCCCUCCUCGUU
>random_seq_from_cds__NO_14066 RANDOM_LENGTH 79 SEQ_LENGTH 56 GC_CONTENT 0.535714285714286 BASEPAIR 19 FREE_ENERGY -18.90 LEN_BP_RATIO 2.94736842105263
GAAGUGGAACUUCUCUGGCUGGCUCUGGAUUGCUUGGAGGGUGGUUUGGAAUAACAGGAGGCACA
AGGGCUGAGUGAUC
>random_seq_from_cds__NO_14067 RANDOM_LENGTH 84 SEQ_LENGTH 59 GC_CONTENT 0.661016949152542 BASEPAIR 20 FREE_ENERGY -23.80 LEN_BP_RATIO 2.95
UUCUGUCCAUUCCUUCUGUCUGCUGCUGCUGCUGCUGCUGCUGCUGCUGUUGCUGCUGCUGUUGC
UGCUGCUGCUGCUGCUCAA
>random_seq_from_cds__NO_14069 RANDOM_LENGTH 84 SEQ_LENGTH 65 GC_CONTENT 0.584615384615385 BASEPAIR 23 FREE_ENERGY -18.37 LEN_BP_RATIO 2.82608695652174
AGCAGGCAGUUCCUAGUUCACUGUCCAGCUCCUCGGUGUGUACCCCUUCCAUCAUCUUCACACUC
UUCCAGUGGCUUCUGCUGC
>random_seq_from_cds__NO_14071 RANDOM_LENGTH 72 SEQ_LENGTH 60 GC_CONTENT 0.4 BASEPAIR 21 FREE_ENERGY -18.50 LEN_BP_RATIO 2.85714285714286
CCCAGUUUUCAGGUAGAGGACCUAAAUUAUCCUCUGCAGAAAGAGGUAGGUAUUGAGGGAACUUC
UGAGAAG
>random_seq_from_cds__NO_14072 RANDOM_LENGTH 94 SEQ_LENGTH 87 GC_CONTENT 0.540229885057471 BASEPAIR 33 FREE_ENERGY -29.00 LEN_BP_RATIO 2.63636363636364
GGUCCGUGAUGGGAGCAGCGAUGAUGCUACUAUUCACAGGUGGUAAUGCUGUUUCUUGGAGAGUG
UGCUCCUCUUGUUCACCAGAAUCGGCCUG
>random_seq_from_cds__NO_14074 RANDOM_LENGTH 70 SEQ_LENGTH 58 GC_CONTENT 0.603448275862069 BASEPAIR 22 FREE_ENERGY -21.80 LEN_BP_RATIO 2.63636363636364
UUGGUUCGCUUCGGGGUCGACUGCUUAGAGCCAGACUGAAGGCUGUGCAAGGCAUCCGUCGUGAU
CACUU
>random_seq_from_cds__NO_14078 RANDOM_LENGTH 99 SEQ_LENGTH 80 GC_CONTENT 0.5 BASEPAIR 26 FREE_ENERGY -23.92 LEN_BP_RATIO 3.07692307692308
AUGUCGCAGGUCCUUGUUCAGCCUUCCUCCUUUUAGCGUUUUCCACCAACUCGGGGAGCUGAAAC
UUUCACAGGCUUCACAAUCUUUUGCUUAGGUGCU
>random_seq_from_cds__NO_14096 RANDOM_LENGTH 72 SEQ_LENGTH 55 GC_CONTENT 0.490909090909091 BASEPAIR 19 FREE_ENERGY -15.60 LEN_BP_RATIO 2.89473684210526
ACGGCUCGCUGAAAGCUCAAAGGAUGAAAGAGAUUCAUGCUUCAAGUCAUCGCCCAAAAGCCUGA
UUUUCUG
>random_seq_from_cds__NO_14102 RANDOM_LENGTH 86 SEQ_LENGTH 60 GC_CONTENT 0.4 BASEPAIR 19 FREE_ENERGY -17.84 LEN_BP_RATIO 3.15789473684211

CAAUGUUAAAUUUCUAUUAUCUGAUGCCGGUGUCCUUACAGUACUGGACCAAGUACUGUCCCAUG
ACUUUUAGUAGAUGAUUAUAC
>random_seq_from_cds__NO_14120 RANDOM_LENGTH 86 SEQ_LENGTH 62 GC_CONTENT 0.548387096774194 BASEPAIR 22 FREE_ENERGY -22.80 LEN_BP_RATIO 2.81818181818182
CCUUCAGCGAGAACCAUGGGGUGGUGAUCCAGCCUGCCUAUAAGGACAAGAUAAACAUUACCCAG
CUGGGACUCCAAAACUCAACC
>random_seq_from_cds__NO_14124 RANDOM_LENGTH 106 SEQ_LENGTH 101 GC_CONTENT 0.514851485148515 BASEPAIR 31 FREE_ENERGY -32.49 LEN_BP_RATIO 3.25806451612903
AUAUCAAAGACCCUAAGAAUCAGGUGGGGAAGGAGGUGAUCUGCCAGGUGCUGCACCUGGGGACU
GUGACCGACUUUAAGCAAACCGUCAACAAAGGGCUAUUGGU
>random_seq_from_cds__NO_14130 RANDOM_LENGTH 81 SEQ_LENGTH 67 GC_CONTENT 0.671641791044776 BASEPAIR 24 FREE_ENERGY -33.60 LEN_BP_RATIO 2.79166666666667
AAACUCCUUCCGAGGAGAAGAAGGGGGAGGGUGAUGCUGCCACAGAGCAGGCAGCCCCCCAGGCU
CCUGCAUCCUCAGAGG
>random_seq_from_cds__NO_14137 RANDOM_LENGTH 98 SEQ_LENGTH 85 GC_CONTENT 0.529411764705882 BASEPAIR 27 FREE_ENERGY -28.06 LEN_BP_RATIO 3.14814814814815
ACAACGAUGACAUCUAUGGGGCUGCCUGGAUCGGCAUAUUUGUGGGCAUCUGCCUCUUCUGCCUG
UCUGUUCUAGGCAUUGUAGGCAUCAUGAAGUCC
>random_seq_from_cds__NO_14142 RANDOM_LENGTH 75 SEQ_LENGTH 61 GC_CONTENT 0.540983606557377 BASEPAIR 22 FREE_ENERGY -23.12 LEN_BP_RATIO 2.77272727272727
ACUAGGCGUGCCUGGUUUUUAUCACAAUCAGGGGCUGCUAUGAACUGAUCUCUGGUCCAAUGAAC
CGACACGCCU
>random_seq_from_cds__NO_14144 RANDOM_LENGTH 87 SEQ_LENGTH 72 GC_CONTENT 0.513888888888889 BASEPAIR 25 FREE_ENERGY -25.40 LEN_BP_RATIO 2.88
UGAAUAUUAACUCAAUUAGGUUCUGCUGGGUUCCUGGAGUUGGGAGUGAAGUGGCUCUUGAGUGG
CUCCCAGCACUUGUGGUAGUUC
>random_seq_from_cds__NO_14153 RANDOM_LENGTH 85 SEQ_LENGTH 69 GC_CONTENT 0.521739130434783 BASEPAIR 22 FREE_ENERGY -18.90 LEN_BP_RATIO 3.13636363636364
UAGCCCCUGGUCUCCUCAAAGACAUCUAUGCUGAACCGCAUUCCUCUCCUGAAUGACGCAGAUCU
CAUUGGGCUGUACAAGCAUC
>random_seq_from_cds__NO_14159 RANDOM_LENGTH 110 SEQ_LENGTH 81 GC_CONTENT 0.432098765432099 BASEPAIR 26 FREE_ENERGY -22.90 LEN_BP_RATIO 3.11538461538462
UUUAACUCAGCCAUAAUGAUACCUGGAGGCUUAUCUGAGGCCAAACCCGCCACUCCAGAAAUCCA
GGAGAUUGUUGAUAAGGGUUAAACCACAGCUUGAAGAAAAAACAA
>random_seq_from_cds__NO_14173 RANDOM_LENGTH 97 SEQ_LENGTH 79 GC_CONTENT 0.455696202531646 BASEPAIR 26 FREE_ENERGY

-22.80 LEN_BP_RATIO 3.03846153846154
AGGUGGACUUUUCCCUCUACAGAGAUUAGACAAAGCCCAUACUGCAUUCCGGGUCAUGGUCAGGC
GGUUUUGCUUUGAAAAUAACCUGCAAAAGAGG
>random_seq_from_cds__NO_14191 RANDOM_LENGTH 98 SEQ_LENGTH 81
GC_CONTENT 0.432098765432099 BASEPAIR 25 FREE_ENERGY
-17.20 LEN_BP_RATIO 3.24
AAACACUGGAUGCUACCUCGCUUUUUGGCAUCUUCCAGGAACAGUGAAAUGUUUCUCCAAUGAUA
GGAUUGUAUGGUUUUUUAGCAAUGGCUCCCUUA
>random_seq_from_cds__NO_14212 RANDOM_LENGTH 83 SEQ_LENGTH 62
GC_CONTENT 0.516129032258065 BASEPAIR 21 FREE_ENERGY
-20.50 LEN_BP_RATIO 2.95238095238095
ACGAGAUUAAAAGGUGACCGAGGAGGUGUCCAUUUCUUAAAGGCUUUACGUCGUGGGGGGCUAAG
AGCCUUCUUUGUUAUAUU
>random_seq_from_cds__NO_14214 RANDOM_LENGTH 102 SEQ_LENGTH 97
GC_CONTENT 0.391752577319588 BASEPAIR 32 FREE_ENERGY
-20.50 LEN_BP_RATIO 3.03125
AGUGAAGUCUUUCCUGGUUGGUGAGCAGUUGUUGUCCAUUUCAGAGCCACGUUUUAAAAUGUCAG
UAUGCAAAUGUUCUUUCCUUUCCACAACUUCUACUUU
>random_seq_from_cds__NO_14222 RANDOM_LENGTH 89 SEQ_LENGTH 80
GC_CONTENT 0.4125 BASEPAIR 29 FREE_ENERGY -18.50
LEN_BP_RATIO 2.75862068965517
AGAUAAUUAGCAAGUGAACUUUUGGAUCUGAACUUCAGUCCUUGUGGGCCUGAUAAAGUACACAU
CAAAUCUUCCUGCUGUCUUCCCAA
>random_seq_from_cds__NO_14230 RANDOM_LENGTH 90 SEQ_LENGTH 73
GC_CONTENT 0.616438356164384 BASEPAIR 26 FREE_ENERGY
-33.80 LEN_BP_RATIO 2.80769230769231
CCACCCUGGGCGGGUGAAAUUGCCCUGUGGUCCUUGGUGGUCCUGGCCAUCGAGCGGUACGUGGU
GGUGUGUAAGCCCAUGAGCAACUUC
>random_seq_from_cds__NO_14231 RANDOM_LENGTH 109 SEQ_LENGTH 99
GC_CONTENT 0.686868686868687 BASEPAIR 31 FREE_ENERGY
-48.60 LEN_BP_RATIO 3.19354838709677
CGCUUCGGGGAGAACCAUGCCAUCAUGGGCGUUGCCUUCACCUGGGUCAUGGCGCUGGCCUGCGC
CGCACCCCCACUCGCCGGCUGGUCCAGGGUACAUCCCCGAGGGC
>random_seq_from_cds__NO_14233 RANDOM_LENGTH 82 SEQ_LENGTH 44
GC_CONTENT 0.681818181818182 BASEPAIR 19 FREE_ENERGY
-26.40 LEN_BP_RATIO 2.31578947368421
CACUUCACCAUCCCCAUGAUUAUCAUCUUUUUCUGCUAUGGGCAGCUCGUCUUCACCGUCAAGGA
GGGCCGCUGCCCAGCAG
>random_seq_from_cds__NO_14272 RANDOM_LENGTH 77 SEQ_LENGTH 69
GC_CONTENT 0.521739130434783 BASEPAIR 25 FREE_ENERGY
-20.90 LEN_BP_RATIO 2.76
UUGGCACUGGUCUGGACGACAUCCUUGAGACGAUCAGAAUUCAGUGGGAGAGAGAUGUUGAAAAG
AACCGGGUGGAG
>random_seq_from_cds__NO_14274 RANDOM_LENGTH 66 SEQ_LENGTH 48

GC_CONTENT 0.625 BASEPAIR 19 FREE_ENERGY -21.50
LEN_BP_RATIO 2.52631578947368
GCCAAGUCCAGAGCCUCCAGGCUGAGACAGAAUCCUUACGUGCCCUGGAAACGAGGCCUGGAGAA
C
>random_seq_from_cds__NO_14278 RANDOM_LENGTH 74 SEQ_LENGTH 51
GC_CONTENT 0.627450980392157 BASEPAIR 19 FREE_ENERGY
-17.70 LEN_BP_RATIO 2.68421052631579
CCGUCCUGGGGGCUGUGUCUGGCUGUCCCUGAUAAAACUGUGAGAUGGUGUGCAGUGUCGGAGCA
UGAGGCCAC
>random_seq_from_cds__NO_14285 RANDOM_LENGTH 89 SEQ_LENGTH 71
GC_CONTENT 0.535211267605634 BASEPAIR 27 FREE_ENERGY
-30.70 LEN_BP_RATIO 2.62962962962963
CUGAAGGAUGGUGCUGGGGAUGUGGCCUUUGUCAAGCACUCGACUAUAUUGGAGAACUUGGCAA
ACAAGGCUGACAGGGACCAGUAUG
>random_seq_from_cds__NO_14288 RANDOM_LENGTH 71 SEQ_LENGTH 55
GC_CONTENT 0.509090909090909 BASEPAIR 21 FREE_ENERGY
-20.50 LEN_BP_RATIO 2.61904761904762
CUCAUGGGAAGGACCUGCUGUUUAAGGACUCUGCCCACGGGUUUUUAAAAGUCCCCCCCAGGAUG
GAUGCC
>random_seq_from_cds__NO_14290 RANDOM_LENGTH 81 SEQ_LENGTH 69
GC_CONTENT 0.565217391304348 BASEPAIR 25 FREE_ENERGY
-24.50 LEN_BP_RATIO 2.76
CCCCAACAGAUGAAUGCAAGCCUGUGAAGUGGUGUGCGCUGAGCCACCACGAGAGGCUCAAGUGU
GAUGAGUGGAGUGUUA

(3). 1442 Zou pseudo miRNAs

>EMBL_CDS:CAA01027 CAA01027.1 Homo sapiens (human) hypothetical protein
CCCAACCCACCUCCUCCUCUUUAGAGUAGGAGGGAACUCAGACAUGCUCACGGACGAAAGACGUC
UAAGACUUAGACAAAACGGAGGAGGAAGAGGUUGGA
>EMBL_CDS:CAA01501 CAA01501.1 Homo sapiens (human) hypothetical protein
CACGUCGUACCGGAAGAAGAACUCGUGCGGCUCGGCGUCCCGGAACCCCAGACCUCGCCGUAUCC
ACUCGGUGUCAGGACUCCGGUGUACGACGUG
>EMBL_CDS:CAA03059 CAA03059.1 Homo sapiens (human) CALPASUAUINE HUMAINE
AAAAUCGGUUAUCAAGGGAUAUAAAACCUCCUUAACACUGAAGAGAAAAUGGGUUAAGGAGAUAC
AUCCACCUUGAGUAACCUAGACUUU
>EMBL_CDS:BAA19117 BAA19117.1 Homo sapiens (human) hypothetical protein
CCACAAAGUAACGAUCUACCUCCACCUUCCCGAAGGCCAGAACAAAGACUCUCACAACCGGGAUU
AUAAAGUGAGGGUCUUUAACAUAGACCGUUCUUUCACGG
>EMBL_CDS:BAA24075 BAA24075.1 Homo sapiens (human) DSIF p160
GUAGAAGUCCCUCUACCACAAGACCGACCCGAGGUGCAUCAGGUGGACUCGUUACAGUAGGAACA
UCUAC

>EMBL_CDS:CAA02705 CAA02705.1 Homo sapiens (human) hypothetical protein
UCUCCAAGUCUAAAAGGGGACCUGCCAUUAACCGGAAACUCCCCCUUUACCACCCCGGUAGA
>EMBL_CDS:CAA02215 CAA02215.1 Homo sapiens (human) hypothetical protein
GACCGUCCUUAGACAAAAGGUGACACCCAGUCGUCGUGUGUCGUAAAACCACCACUGAAGUCUCG
GCAUCAUCACCCCGACGACUGUGAGGGCCACCCUUUAGUCGAAGUGUUGUC
>EMBL_CDS:CAB58589 CAB58589.1 Homo sapiens (human) FSH receptor
CCCUCCAGUCUUCCUUAGAGACAGUGGAACGAGAGAACCGUCUCUUUUUGGGACAAUCUCGUCAC
UGUCUAGGCUACUACUGUAGGA
>EMBL_CDS:CAA01870 CAA01870.1 Homo sapiens (human) hypothetical protein
GUACCGGCGAGGGUAGCUGGCACACCCGGUCUGGUAACUCGCGUACCCGAGACCGCACCUCGCGU
ACCGGGACGGUAGCUCGCGUACCCGGACUCGUACCUCGCGUACCACGGGCGUCCAUACCCUCGAC
CGGAC
>EMBL_CDS:CAA01506 CAA01506.1 Homo sapiens (human) hypothetical protein
CAGCGAGUCGUAGACCAACUAGACACGCCACAAGACGUCGUGUCGCGCGCGGCGGACGCGGUCCA
CCAACUCGCUG
>EMBL_CDS:BAA19935 BAA19935.1 Homo sapiens (human) BMAL1b
AGACACAGGUACGAAAGAACCAUCAGUCACCUCGACGGAAAGGCAAACGCCAACGUUAGGUGUGG
UGACCAUGGUUCUCUCGACCUUUCGUCC
>EMBL_CDS:BAA20771 BAA20771.2 Homo sapiens (human) hypothetical protein
CGACGGGGGUAGUGAGUGGAAGCGACCUCUAAACGACGGGAAUCAUCACCCUCUUUAACUUUGUC
AUCACCAUCGUCAGCACCCCCAUCA
>EMBL_CDS:BAA20774 BAA20774.3 Homo sapiens (human) KIAA0316 protein
UGAAACCUCCGUUCGGGGGUCGCCCCCAGAACCUCCGGCCCUUGAGCCAGACGAGAAGACGUUCC
CCCACCAUCGGAAGCACUCA
>EMBL_CDS:BAA20767 BAA20767.3 Homo sapiens (human) hypothetical protein
UGAAAGUAGAGGAGAUCUUAUUAUGGAAAUCGUAGAUCCUCGGAUCCAUCUCUCCAUUCUCCUCU
UCGUCUUUCA
>EMBL_CDS:BAA22954 BAA22954.2 Homo sapiens (human) hypothetical protein
CCGGACCGGAUCCUCAAGCCACAGAGGUGUCGACGACGAGGAGUCGUGAAACUCCCAGUCGAGGG
CCGAGACCCCCGGGUCUAGGUCACGGUCCAG
>EMBL_CDS:BAA23691 BAA23691.3 Homo sapiens (human) KIAA0395 protein
CGUCUACGAAGACCCCCGUCUAGGACGACCCCAGGAAGUCCGUUCCAGAGUCGCCCGACCCGGAG
GUACGACCGUAGAACG
>EMBL_CDS:BAA20837 BAA20837.2 Homo sapiens (human) hypothetical protein
CACCCUCGCUCCUACCCUUACGUGACCCUAUGACUCCACGGGGAAACGGUAGAUUCGAGGGUA
>EMBL_CDS:BAA20763 BAA20763.3 Homo sapiens (human) KIAA0304 protein

GUGGACCCCCGAGACUCCUUCCCCGCCCCGACACGUGUUACCAGACCAAGAGGAGACGUCGGAGG
UCCAC
>EMBL_CDS:BAA20808 BAA20808.2 Homo sapiens (human) hypothetical protein
GUGAGUUGACUGUGUAGUAUAACAACGGAUCCGACACCGAGAAGGACACAGACCCCUGACCUCCA
CACCCCUCCGACGGUCGUUGUCUACACAGUCCUGUCCAC
>EMBL_CDS:BAA24848 BAA24848.2 Homo sapiens (human) hypothetical protein
CCAAACCUCCCCUACCCCCCGCACCGGAAGAACGAGACCAACUGCCACAAGUCACGAACCUGCGC
GGAGAGCUAGAAGAGGUCCGA
>EMBL_CDS:BAA24847 BAA24847.1 Homo sapiens (human) hypothetical protein
UAGACCACAUCGGCGUCGAAAGCCGUCAUCUAUCUCCGACGGAGUCCGAGGUUCCGUUACUACUC
GACGAGGCUCAAGAGCCCCCUCCGGUCCGGACGGACCAUCCGUCGAACAGAGUACUUGACGAACC
GGCCGACGAAGGUCUA
>EMBL_CDS:BAA20773 BAA20773.2 Homo sapiens (human) KIAA0314 protein
UCCCUGAGCACUCUUCAGUCCGUCCCCGACCACCAGUCAGUCUGUGUAUAGGAGACAGCCGAAGA
AGUAAAGGA
>EMBL_CDS:BAA32288 BAA32288.1 Homo sapiens (human) A-type microphthalmia associated
AGACGGAGAGAAACCGGUCACGAGAACGAAGUCUGAGACACUCGAGGGAAAAAUACAACCCUUCC
AACCGACCUGUCCUCAACGACUACCACUCCGGACCACCCCCGUCU
>EMBL_CDS:BAA34700 BAA34700.1 Homo sapiens (human) hypothetical protein
CCCGUCAACCCCAUCUCCUCCACGGCCCGUAAGUGCACCAUCGACAGCGGGCCGAGGAGGCAGAA
GAAGGACGGA
>EMBL_CDS:BAA20839 BAA20839.2 Homo sapiens (human) hypothetical protein
CCUAAGGUCAUCUAGGAGAGGUUUAAGGUACAGAUGACCAUCCAGAGGUCGGUCCCCGAAGAGAC
CGUCCCAGUUACCCAGUUUACCUGACCCCUUUAGUGACCCCAGG
>EMBL_CDS:BAA20778 BAA20778.2 Homo sapiens (human) hypothetical protein
GGUGAAGACGCUCGACCCACUACCACCGACGACUCCUGACCCGGCGGACCCGGUCGAACAGUCCU
CACC
>EMBL_CDS:BAA19790 BAA19790.1 Homo sapiens (human) proteasome subunit p27
UCCGGGCACCUCCCGGGACCGAGAGUGAGACUGGGUCAAACGCCGAGUACCGGAGAAACACCCGG
A
>EMBL_CDS:BAA20758 BAA20758.2 Homo sapiens (human) KIAA0298 protein
GGAGAGGUUGGACCAUCUACUGUUGAGGUAGAGGUCUCACAACCUUUUCGGGUACUACGAGUCGC
UCCACCUCACGAAAGAGUGGUGUAAACCUCUCC
>EMBL_CDS:BAA20759 BAA20759.1 Homo sapiens (human) hypothetical protein
AAAACCGACUCCACUAUUAAAGAUCGACUUCCGAACCGACUAAAUACUUAUCUUGACGGUCCGAU

UUCAUUCUUAAGGUGUGGAAUUUCAGUUUC
>EMBL_CDS:BAA20756 BAA20756.2 Homo sapiens (human) hypothetical protein
CGAGAGUAGACACACGGGUAACCACCACCCGUAGGGAAAGAGGAGGGUCGCACCGAGGACGGAAA
GUGGGAGUACCCUGACGACCACAAGGUCGUCGAGGGCCCCCUCGGCCUUCUGUGCCCCCGUCACC
CCUGUCACCAUUCCGGCUAUGACCCGUACGUCAGCUCUCA
>EMBL_CDS:BAA20762 BAA20762.1 Homo sapiens (human) hypothetical protein
GUGAGAAGUCAUCCUCAAGAUAUUGAAGACACACCUGUUCAGGUACGUGACCAAGAGGUAACUAC
ACUCAC
>EMBL_CDS:BAA20822 BAA20822.2 Homo sapiens (human) hypothetical protein
CCCAUAGUUCAAGUAGGUGUAACUACAGUUAAAGUAGACCUGACAGGUUCAAUAGUAGUCUCUCU
UAUCUAGGAAGUGAAACCAGGUCUCUUUCUAACUAUAAACCCCGACACUCGAACUAAGAG
>EMBL_CDS:BAA23417 BAA23417.1 Homo sapiens (human) NUAK
CGACGACGACGAGGAGAGCGACGACGACGACGACGACAGCGACAUCGACGACGUGGCUGGGAAGA
GGUCACCGCCGCCGCCGUCGCGACUCGUCGUUUGGACGGCG
>EMBL_CDS:BAA20788 BAA20788.2 Homo sapiens (human) hypothetical protein
GAGAAGAGACACAUCCUAAAGGACCUCGGAAGGUCAUUCGACCACCCGAGUAAUUUCUUCAGGAC
GCAACUCGAUCUC
>EMBL_CDS:BAA20784 BAA20784.1 Homo sapiens (human) hypothetical protein
AAGGCCUGGGACGUCUAGGCCCGGGGUGUCGCGUCAGAGCCCGAGGUAGGGUCUCGAGUCCUGAC
GUCACAAGGUCCCUGGCCGAGACCCCAGACCCU
>EMBL_CDS:BAA20562 BAA20562.1 Homo sapiens (human) PKU-beta
CGGAAGAACCUAGGUCUGAUACUUCGAGUAGGUAACGUGGAAGGGACCCGGAGGGCCUACCGCCG
CACACUAAGUCGUCCCUGGACCGGCGGCGGCGGCUCGGGCCCCAACCUCUGCACCUCUCGACCCU
GGUUCUACCG
>EMBL_CDS:BAA24841 BAA24841.2 Homo sapiens (human) hypothetical protein
CCCUCUCCGCCUGCUACUGUAACAAGUACUUCUACAGUAACUCCCACCGUACCAGAGCCGAGAGG
G
>EMBL_CDS:BAA20783 BAA20783.3 Homo sapiens (human) KIAA0325 protein
ACACCCAUGACCUGGAACUUGAACAGCACUUCUCUGAGUUACAGUAGAAAGUGCGCGACCUAGUC
GACCCAGACCAUAAGCGC
>EMBL_CDS:BAA20840 BAA20840.2 Homo sapiens (human) hypothetical protein
ACUGAAACCGAGAACCGGAAGAGGGUCCCUCAAGGACCUUCUACGACUCGACCACAGUAGGUAUG
GGAGUAAGACCCUCCGGUUCGACGACUCCAAU
>EMBL_CDS:BAA20841 BAA20841.2 Homo sapiens (human) hypothetical protein
CCCCCCGCCGACCACGGUCGUUUCCGGACCCGUAAGAACUCCCACGUGCGUCUUCCAUGACACCG
AGGUCCGACGGGAGG

>EMBL_CDS:BAA25420 BAA25420.1 Homo sapiens (human) perilipin
UCCCCGACUGAGAAGGAACAGACGUCCCUCCUCCAUGAGGUGGUGGAAGAGUUACGACGGGUUCC
GGUUCAGCCGGGGA
>EMBL_CDS:BAA22962 BAA22962.2 Homo sapiens (human) hypothetical protein
GACGGACCGGCUCGACCAGUCUCUCGGUGUCGACGUACGCCUACUUCCCGAGGGCGGGGAAGACG
CAGUCGAACGAGGUACCGAAGCCGGCCCUGUC
>EMBL_CDS:BAA20793 BAA20793.2 Homo sapiens (human) hypothetical protein
CAACGGUAUUAAUUACGUUAUUAAUACGUAUAGAGGUGACAACAUACGUAAACCAAACCGUUG
>EMBL_CDS:BAA20760 BAA20760.2 Homo sapiens (human) hypothetical protein
CCAGGGCGAGGGGACUCCCGUCGGUGGGAGGACCAUAACACCCCACACGACCAAAGGCUACGACU
AGCUCAGAACGACAUCAGUCCCCCUCGGCGACGAAAGAGGGGGCCCCUCCCCGACGGGAGUACCC
CCUCGGACUGA
>EMBL_CDS:BAA20819 BAA20819.2 Homo sapiens (human) hypothetical protein
CAGUAAGGUAUCUAACCGGGAACCGUGGACUCUAAGUCCGAAAGAGGUCCACGGUACUACCCGGG
UCCUACCCGACAG
>EMBL_CDS:BAA23698 BAA23698.3 Homo sapiens (human) hypothetical protein
ACAAGACCACCCACCAGUGACUGACACUUGUAGGGUACGACAAGACCACCCACCAGUGACUGACA
CUUGUAGGGUGCGACAAGACCACCCACCAGUGGCUGACACUUGUAGGGUACGACAAGACCACCCA
CCAGUGACUGACACUUGU
>EMBL_CDS:BAA24932 BAA24932.1 Homo sapiens (human) hypothetical protein
GUGCCUGUGAGUCUGGCAAAGACACUCUAGACAAGACUCGGUAGAAACCGGUGUCGUCCACUAUU
GACGUAAACCGUUCCUCACAGGCAC
>EMBL_CDS:BAA20828 BAA20828.2 Homo sapiens (human) hypothetical protein
GUACUUCAAAAAGAACCUCCAUAGAUAAAACAUUUACAUUUAGAAGAUGUUGUCUUAGAGCUCCU
UCUCGAACCAC
>EMBL_CDS:BAA19943 BAA19943.1 Homo sapiens (human) scFv collagenase IV antibody
CUACCAGUUCCUUCGACACAAAGGACUCGACCUCGGUCUCUUAGCCAGACCCUAGGGACUCCCGG
CCAACAAAAAUGGUAUCUACUGUUCAUGUCCCCGGACAGGACCGAAGACGACCAUGGUCGAACGU
AUUAUCGAAGACUCCGACAG
>EMBL_CDS:BAA25666 BAA25666.1 Homo sapiens (human) hGLI2
CACCCCGACCCGAGCUCCCCUCCGGCCCCGACGUGAUCCGGUUUCGGACGACAUCGGUGGGGGUA
GCCCGGACAAACCCCGAGGUA
>EMBL_CDS:BAA20755 BAA20755.1 Homo sapiens (human) hypothetical protein
ACGACAAGAAGCAUGACGACGGCCAUUCGGCACAAUCACGAGUCUUCCACCCACACCAUUGAGAC
CAGCGACAUCGGUAUCCCCCCAUGUAUCCGUAUGACCAACAUCAUGUCCCUGACGU

>EMBL_CDS:BAA21573 BAA21573.2 Homo sapiens (human) hypothetical protein
GACCUAACUACUACGGUUUCUCGAGUAGUAAACACUGACGAAACCCUAACUCCAGUCAGGUC
>EMBL_CDS:BAA20770 BAA20770.2 Homo sapiens (human) hypothetical protein
UCGUCGAGGCAGAAAAAGUCCACAGACUACCUGUAAAGGUGUGACAUAGCGAACAUUUCUACGAC
GACGA
>EMBL_CDS:BAA32590 BAA32590.1 Homo sapiens (human) retina-specific amine oxidase
CCUGACGGGGAAACGGGUGAUUAUAUACCUACAGGUGGUAGCACCGAACUAUCCCCGUCAGG
>EMBL_CDS:BAA23712 BAA23712.2 Homo sapiens (human) hypothetical protein
ACAAGAGGCUGCCAGAACAGAACAAAAAGUCGCACAAGACAUACGAAAAGUCGUACCGACUCAGU
AACCUUAACUCCGAUUGUCUUGAAGGGACUGAGUGUCCUGACGAAAACCUCCUGU
>EMBL_CDS:BAA25535 BAA25535.3 Homo sapiens (human) KIAA0609 protein
CAGGUCGAGUCUGUCGACGAAGACCUCGAAAUCUAGUCUGUGCAGAGACAUCGUGACGAGCCUCG
CCCACACCAGACUGUCGACGUGUAAGGUCUUCGUCCCCUCGACCAUG
>EMBL_CDS:BAA36409 BAA36409.1 Homo sapiens (human) NBR13
CACGGAAGAGGAAGUCGCAGAGGCCACCUCCCGACCACACGUGCCACCCUAUGUGGAAGGGCGAC
GGGUGUCAGGACGGCCUACGGAGUCUACCCCUGGACCCUCCCCCGCG
>EMBL_CDS:BAA32313 BAA32313.2 Homo sapiens (human) KIAA0468 protein
GUCCGACCUACAAGCACCACCCGUCGUGGCGUCCACACCACCUGUGGCGGUCCCGAUGUAGACCC
GAC
>EMBL_CDS:BAA32312 BAA32312.2 Homo sapiens (human) KIAA0467 protein
CCGUGCGCCCCGUCCGACUACUCCCCCUGUGGGAGUCUUAGGAGACCCCUGGGUCCACGAACCCC
CGACCGACAGUCCCAGAGGGGAAGCUACGGAAGUCGGGACAGGAGUACUGACACGG
>EMBL_CDS:BAA25898 BAA25898.1 Homo sapiens (human) sodium bicarbonate cotransporter2
GACAUUGUGUCAAAUAGGAACCAUGACUUCUACUACUACUUCUUGUAUAAGGGGUACUAGAGAAG
UGCCUUUACCUACUUGACACAAUGUC
>EMBL_CDS:BAA32289 BAA32289.1 Homo sapiens (human) KIAA0444 protein
CACGGGGCCCCAGAAAGAGUAGGUACAUCGGGUCGACCCGAAGGUAAAACAGACCGUCCGGGUCG
CCCCGACCGUCCUCCACCCGUCCCGACCGCCCGUG
>EMBL_CDS:BAA24112 BAA24112.1 Homo sapiens (human) osteonidogen
CUACAAGGAAGAAACCACACUCCUAGGUUCCACCUUCUAAGGAGAUUUCCGAAACAUAGGUGAAA
CCUAACCUUCCUUUGUAG
>EMBL_CDS:BAA25530 BAA25530.2 Homo sapiens (human) KIAA0604 protein
CCGGGCAAACAUGAGGUGGUACAACGACGGUCGAGGGUCGAGGACGUCGCGCUGCAAGUACCACC
UCAGCCCGG
>EMBL_CDS:BAA25461 BAA25461.1 Homo sapiens (human) KIAA0535 protein
CCUCAACCUUAUCUUCACAAUCUCCUUCAAUCCCCACGUCUGAACAGGUCCUAAGCUAAAAAAAA
GUACGAAUUCAGGUUACACGGUAAAAGUAGGUGAAGAUAAAGCCGAGG
>EMBL_CDS:BAA25458 BAA25458.1 Homo sapiens (human) KIAA0532 protein
CCUAACGUCUGUCUAUCGACACCAAAUAUUUUAUAAACUAUACCACAUAAUAACUAAUAACAUCA

GAACAGAAGUUAGA
>EMBL_CDS:BAA25533 BAA25533.1 Homo sapiens (human) KIAA0607 protein
AAGGAACUAGUCCUAGAGGGCCCCGGGUAGAAGUUGCCAGUCCACCGUGGUCGGUCCGUCCUCCU
CGGCCUCUCGCAGAGGACCGGUCCACCCGGGACCCCACCACCCCCUC
>EMBL_CDS:BAA32319 BAA32319.3 Homo sapiens (human) KIAA0474 protein
AACCAGCACCCGUAGAGACCCCUCGAGACUCACCUACUCAAGAGGCUGAACCCGAGGACACUCUG
CACGGGCGACAGACC
>EMBL_CDS:BAA31199 BAA31199.1 Homo sapiens (human) natriuretic peptide A type receptor
CCUCGAGUCCCCGGCAGGUGUCGAAAAACCGUAUUUGCCACACAGGAACGAGACCCAGGUCCAGG
GACUUCGAGA
>EMBL_CDS:BAA25522 BAA25522.2 Homo sapiens (human) KIAA0596 protein
CCCUUCGGACUCCGGCUCCCCCGAAAAGUAAGACCGAUCACUCACUUCAGAGUUCCCGGAGGACG
GGUACGAAGGA
>EMBL_CDS:BAA25526 BAA25526.2 Homo sapiens (human) KIAA0600 protein
GGUGGAAGACAAAAUCGGAACUUGCGUGAAAGUUCAACCCAAGUCUCCGACAAAACGCCUCCCCC
>EMBL_CDS:BAA25510 BAA25510.1 Homo sapiens (human) KIAA0584 protein
ACAGACUACCCCGAAGUCCCCCUACCCGUCCAUCGGUAUCACGAGAGACAACGUCUCCAUGUAGA
CCUACGGACGAACGGACGACCUCUUCCGUUUCUGU
>EMBL_CDS:BAA25479 BAA25479.2 Homo sapiens (human) KIAA0553 protein
GACCCCGUAAGAAACCCACACAUCAUAAUAGUGGUAGGUAAACAAGUGACCGAGAGUAUUUUUCA
UCUUUCCCUUUUAAUCCAAAAUGAAACGUCACACGACCCCCCUACAUCACCAUUAUGAGUCCGAG
ACAUCGGGGUC
>EMBL_CDS:BAA82611 BAA82611.1 Homo sapiens (human) ganglioside sialidase
GGUACCACGGUACACUGGGGAUCCAGUAGUGACAUCUAGUAGUCUCUUACUCCGGACCAAAAUGU
ACCAUCGACC
>EMBL_CDS:BAA25482 BAA25482.2 Homo sapiens (human) KIAA0556 protein
CCACCAGGAAGACGUAGGACGUCACCCGACCGACCGACGAGCCGGAAAGACGACUCCUCGCGGGA
GACGUCCCGACGCAAGAGGUGG
>EMBL_CDS:BAA32136 BAA32136.1 Homo sapiens (human) aurora and IPL1-like
ACACGUACGCGGGGAGUUAGUAGAGACCCCCGUCCAUCAGGUCCCACGGUGUGUAACAGAAGGAG
GAGUCCCUCCCGCGUACGUGU
>EMBL_CDS:BAA25505 BAA25505.2 Homo sapiens (human) KIAA0579 protein
UACCACCACCACCACCACCACCAAGCCUAAGCUCCGUCCUUACAAAGGACACCUCGGCGGUGGCG
GUGACGACGAUA
>EMBL_CDS:BAA25467 BAA25467.2 Homo sapiens (human) KIAA0541 protein
ACCGUUAAGACUGUUAUUUAACCAAACCUAAGGAGGAGUUUAUAACCGAGUCAUAGGACGUACAG
UGAAUAAAGGCUCCAUUGUUAGGUCUGGAAGUCCUACGGU
>EMBL_CDS:BAA32295 BAA32295.3 Homo sapiens (human) KIAA0450 protein
CCAGGGGGUCGUACCUCAGCGCGCCGAACCCUUCGACGACCGGUGACUACUAGGCCUCGUGGUCG
ACGAAGAACUCGACGGGCCCGUCCCCCAGG
>EMBL_CDS:BAA25474 BAA25474.1 Homo sapiens (human) KIAA0548 protein

UAACUCGUCCCCGACACCUAUCUUUAAAGGACAUCCCCGAACGCUUCCGUAGAAGUGACCAGGAA
AUAUCCUUGGGAGAUACGUAAAGGGAAACGGUCA
>EMBL_CDS:BAA24863 BAA24863.2 Homo sapiens (human) hypothetical protein
GUCUUGUGGAGAUCUUAUGUGUCCUACUUCAAAUAAAUGUCACAGUAGUAGAACACAGGAGACUU
CCAGAC
>EMBL_CDS:BAA32404 BAA32404.1 Homo sapiens (human) Stem cell growth factor-alpha
UCCCCACCCUCUCCCCGACCCCGGCCUCCUACCCCAACCGCAACGAAGGAGGAGGAGAAGGAGGG
GGACCAGGAGGAGGUAGAGGGUCAGGA
>EMBL_CDS:BAA37096 BAA37096.1 Homo sapiens (human) hypothetical protein
UAGACCAGCGGGUCCAACACGUCGGUCGGCUACGCACUCAGGUACACACCGACGAGGUACAACGG
CUACCGCAUGGUCUA
>EMBL_CDS:BAA25519 BAA25519.2 Homo sapiens (human) KIAA0593 protein
GUCAAGGAGGUCCACAACAUAGGUAUGAUGGUAUAAGAAAUAAUAAGUAUAAGUAACCCCUUUUA
GGGUAUUAUACAACAAGGUUACUACCACCACAUCCUAUGUAAAAUACUUCUAGAC
>EMBL_CDS:BAA25478 BAA25478.2 Homo sapiens (human) KIAA0552 protein
CUCCACCGGUCCUCGAACCCACCCACCGACCCGAGUGCUAUGACGCCACCCCGAAACAGUGACGG
UGGUGACGACGACGACGGAG
>EMBL_CDS:BAA32298 BAA32298.3 Homo sapiens (human) KIAA0453 protein
CAUAGAACCCGAGUAGGUGACAACCUCUUCCUCCACCCCAGUUCCCUAUCAGGAACAGAACCUGA
AGGAAAACGAAAAAGGCCCUGCGUCUCCACCCGGAAACUAUG
>EMBL_CDS:BAB69038 BAB69038.1 Homo sapiens (human) kinesin-related protein
AACGAGUACGAGACCGGUUCCGACCAAAACGAGUAGCACAAAUUGAACCACCGACCUUGACCGAA
CCUGUCACCGUACCCACC
>EMBL_CDS:BAB19784 BAB19784.1 Homo sapiens (human) MOP-3
UACGACUAUUGGUCGGUAAUAGUUACCAAAGGAGUCUUUACCUACAAAACAUGGUUUGUAGUCCU
CCUCAUUGUGACCUAUUUCUCGACCAAAGGUGAUGUCGUA
>EMBL_CDS:BAA32695 BAA32695.1 Homo sapiens (human) hypothetical protein
UGGACGCAUGGAAACCCCCCCGUGACCUCAGUAUCGUCACGAGUCAGGGGUCUACCAGGUGUCCA
>EMBL_CDS:BAA31684 BAA31684.2 Homo sapiens (human) KIAA0709 protein
CGACGACCCCUGCCCGAGUCCCUCCCCAGGCACGGGAAGACCGUCUACUUCGGUACCCAGAGGAG
GCACGUCGAGGCUAGCAGGGUCGCCG
>EMBL_CDS:BAA31593 BAA31593.2 Homo sapiens (human) KIAA0618 protein
CGACGAGGGCGACGACCGCCAGACCCACCACCGAGGUUUCGGCCUCGACCACCGACGUGGCUUCU
UCUGUCGUCACCUCGGCGGUUUCCGCCCGACACUCGACCGCCCUCGCG
>EMBL_CDS:BAA31613 BAA31613.2 Homo sapiens (human) KIAA0638 protein
CCGGACCCGGGACAUCACUCUACAGAGACCGACGUCUCGGUUAGUGGCAGGAGGGAAGGAGGUCG
CCGUCCUCCCACUACCGACACGACUCAGCGGGUGACGGGUCCGA
>EMBL_CDS:BAA31675 BAA31675.2 Homo sapiens (human) KIAA0700 protein
AAGACGCGGACCGCCAUCUAGAAGUCGUCGGACCUCGUGUCCCAGACCACGUCCGCGUCCU

>EMBL_CDS:BAA31683 BAA31683.3 Homo sapiens (human) hypothetical protein
ACCGAGUCCCAGGAGUGACAUCGAGACGUCGUCGACAAGUCGCGGGGUCACCCUUACUCGGU
>EMBL_CDS:BAA74737 BAA74737.1 Homo sapiens (human) hypothetical protein
ACUCGGUCAAGGAAGACCAUCCGACGCUACAUCCCCUAGGGGAAGUACGUCUGGAGCAUCCCCC
GACCUAGU
>EMBL_CDS:BAA31685 BAA31685.2 Homo sapiens (human) KIAA0710 protein
AUAGGAGUAAGAGUAGGUCGUCCAUUAGUUUAUAUUACUCCGGGGGUGCCCGGUAUAUGAACUCC
AACAAGAACCACUCUUUUUCCUAU
>EMBL_CDS:BAA31649 BAA31649.1 Homo sapiens (human) KIAA0674 protein
ACCGGAAAGAAUCCGACAAAACCCCGACACAUGACCUUUUUAAGUAAAGGUACCGGUCGUCGACG
GACUAGGUCAGGUUUUUCACUCCGGU
>EMBL_CDS:BAA31616 BAA31616.2 Homo sapiens (human) KIAA0641 protein
GAGACCCGGACUCCACCGGAGCCGGAGUCGGAGCUCCAGACUCUUCAUCCGUCUCAGAGCCAUCC
CUAAGAAGAGCAACUCCGGUGACUCCCUCCACCUCUCGGCACAGAGCCCGGGCCCC
>EMBL_CDS:BAA31604 BAA31604.2 Homo sapiens (human) KIAA0629 protein
AGAAGGUGUACGUUUGGUAGAUGUGUCCAUUCACGACAUCUACUCUUUCAUACACUUACUCCU
>EMBL_CDS:BAA36753 BAA36753.1 Homo sapiens (human) huntingtin
CGGGACGGACCUCUAGGUCUGAGUCGCCCUAGACGAGUACCUAGUUUACGGUCCUGUCCCG
>EMBL_CDS:BAA31587 BAA31587.2 Homo sapiens (human) KIAA0612 protein
GUCCCUCUGUGACAGACGGAGGUCGAGGAGAUGGAGUGACCGACACAGGUGGAGUCCACAUCCCG
UACUGAACCCCGACAACACCUACCUGAGCCUCACCCUCCUCAACCUCUCUCUGAGACGGAAGAAC
>EMBL_CDS:BAA76877 BAA76877.1 Homo sapiens (human) LCAU-like lysophospholipase (LLPL)
UCUUCCUCAGACCACACCCCUGCGGUCACGGUAUCUCCGUCACGUCGACGUGCGGUCCACCGUAG
CACCGAAGGUGGUCGGGAAGA
>EMBL_CDS:BAA83718 BAA83718.1 Homo sapiens (human) RNA binding protein
AGAUCUCGCCGCGGCUUAUCGACCUCCACAGGCCCUGGAUCUCGCCGCCGCACAGUGACCACUCC
AAGCUCUCGCCCUGGAAGAAGCUCAAUGACCUCUACACGCUCUAGCUCUAGAGGAAGCCCACUGG
CCUCUACAAGACCUAGAACUAGAAGACGCUCACUAUCCGCUUCAAGACGAAGCUCUAGCGGAGGA
CCAAUGACCACCUCAGGACCU
>EMBL_CDS:BAA33713 BAA33713.1 Homo sapiens (human) HIP1R
CCGGACUGAACCACCUCCGGUGGUGUAACCGUCGGGAGAGUAACUGACACGCUCUUGUAAGGACG
UCCGCCGAGUCCACCCCCGACACGAACAACCGGAAGUGGAACCUCCGG
>EMBL_CDS:BAA31606 BAA31606.2 Homo sapiens (human) KIAA0631 protein
CUACGUGUCACAUCCCUUCGACACCCGUCGACCCCAGAUACGCGUCCGCGUGGGCGGGUAGCCGG
GCUCAGCAGGUGUCGCGGAGGAGACCUCGUAG
>EMBL_CDS:BAA34529 BAA34529.2 Homo sapiens (human) KIAA0809 protein
UCUCGACAGGGCCCUUGUCGUGACGACAGGUAUUCAACCCGGCCCCCCGGUCCGUCCCGCCGUAA
CCACGAACCCCUCGAGA
>EMBL_CDS:BAB61018 BAB61018.1 Homo sapiens (human) hypothetical protein

AUACUGUCGGUACAAAGGGUCGACCCCGUGUGGUCCCGGUCGGGGACCGUACCGGACAAGGUAAG
GAACGUACCAACAGUAC
>EMBL_CDS:BAA34515 BAA34515.1 Homo sapiens (human) KIAA0795 protein
GUCCACCGACACACCACCAACAUCACAAGGUGUGACGACUUCUAGACGUUUGGUAGUACCGGCGG
AC
>EMBL_CDS:BAA76776 BAA76776.2 Homo sapiens (human) KIAA0932 protein
CUCUAGGAGGUCUGCGUCACCCUCAUUCUACCCAUGGGUAAACCCUUUGGUCCACGUCUUUUCAA
AGGGCAACACAGGACGUCCCAGAG
>EMBL_CDS:BAA34517 BAA34517.2 Homo sapiens (human) KIAA0797 protein
CUCUAUCUCCUCCUCAUCCGACUAUCCAUGAUGUCACGGCAAAGACAACAUGAAAGGAGAAUUAA
AG
>EMBL_CDS:BAA74870 BAA74870.1 Homo sapiens (human) KIAA0847 protein
CUAUCCGAAAGAAAACCUCCUCGAAAAGAAAACGUCUUUGACCUCUCCUACUAAGGUUUCAACUC
AGAUAG
>EMBL_CDS:BAA74936 BAA74936.1 Homo sapiens (human) KIAA0913 protein
GAACGAGUCCCACAACCAUCGGUGGGUCCAGACUGCCGAGCACCUAUGGAACAAGAAGAACGGGA
ACUCCAGCCCACCCAACCGAUCGUCUAGGACCCGUUC
>EMBL_CDS:BAA76782 BAA76782.2 Homo sapiens (human) KIAA0938 protein
GGUUACGGACUCCCCCCGAAAAAGGGAAGUAGAGGUGACGAAAAAGGACGUAGACUUCCUCUAGA
AACAUCUCUACUAGGAAAAUCCCCUCGAAAAGGAAAGAGUCUUCGAAACC
>EMBL_CDS:BAA34445 BAA34445.1 Homo sapiens (human) KIAA0725 protein
UCGAAACCUAACACAUUUUAUUAUUAAAGAGACAACCCUCUAAGGUCAAAGAAAAAGGUAAGUAG
GUUUCAA
>EMBL_CDS:BAA75631 BAA75631.1 Homo sapiens (human) semaphorin W
CCGGUGUCAACCAACAUAAGUGAACACAGUGGAGUCAUGCCCUCGGUUGGUCCUCGGUCGACACC
AUGUUAAAGUACAAGAGUUGACCGACACCGA
>EMBL_CDS:BAA34523 BAA34523.1 Homo sapiens (human) KIAA0803 protein
CCGAAGAGAAACGUCAACGUAGAAACAUUAAUUUAGGAAAUCUCUACUCUAACAUAACUACAUUC
GA
>EMBL_CDS:BAA77349 BAA77349.1 Homo sapiens (human) hypothetical protein
AACAGUCUCAGUCGAUCUCUAAGGAGUAUAAAUUCUUACACGAGUUGAUCCAAUAGUUGUUCAAC
UCAUGUUCGAAAGAAGUGUCCAAAUAUACUCCUUAGGAGAAGUCUGAAGUUGUU
>EMBL_CDS:BAA34470 BAA34470.2 Homo sapiens (human) KIAA0750 protein
UGGUGUCCCGAAACCGAAAGGUCCACCAGUGGAACCUGAACUCGAACCUUAUUUUCAAAGACACC
A
>EMBL_CDS:BAA34436 BAA34436.3 Homo sapiens (human) KIAA0716 protein
CGUAACGACUCGACCUACCGACUCCAGCCGACCACUAGAGGUUAUACCAGGAGUCGUCUUAUAGG
UCGUUCGACCG
>EMBL_CDS:BAA76374 BAA76374.1 Homo sapiens (human) interleukin-18 binding protein
ACUGGUCCUCUCACUGCUGCACCCGUGUGUCCUCGUCCUGGGUGUUUCCCGACUCCAGACCACAG
GUCAACACAGAGUACCAGU
>EMBL_CDS:BAA78717 BAA78717.1 Homo sapiens (human) hypothetical

protein
UAACCCAUAACGUAAAAUAGUGUCAUCCACAUACUUUAUGAACGUAGCCUACUCAACGUCUGGGU
UA
>EMBL_CDS:BAA34508 BAA34508.2 Homo sapiens (human) KIAA0788 protein
ACAAAACUUCGGGAGUUUGGGUCGGACCCGUAUGAAACCGUCGAAAAGGUGACCUUCGUCAACAA
GAAGACUCGGUUUCCCGAACCCGAAGUCUCGU
>EMBL_CDS:BAA34467 BAA34467.1 Homo sapiens (human) KIAA0747 protein
CACGACCCUGUGGUCCUAGGGAUCGACACGAGAGUCAUCGUGGACGGGGACUGGUGACGACUCAC
AUUUGGUCGCCAGGUUCGUCUCGACCAGUCGGUGCUCCUCGAGACUCUCCCCGUCCCUAUUACUC
GGGUCGUG
>EMBL_CDS:BAA34480 BAA34480.2 Homo sapiens (human) KIAA0760 protein
AGGUCCGUCUAGACUGUACACACCGAGACGACCCCGAACAGGCGCACCUACCAGAAGUCCACCUA
GAGGUCGUGCCGGUCCGACAAUUUCAGGGCGAACCU
>EMBL_CDS:BAA74887 BAA74887.2 Homo sapiens (human) KIAA0864 protein
CCGGGCCCGACGGACACGGACGAGACUCGUCCUCCCGAGGGCCCAAACGGUCCUGAGGAACGUCU
CGACCUCGAGGAAGAGUAUAAGAUCGGCCUAAGACGUCCUGCGUAUCGUUUGGACCCGG
>EMBL_CDS:BAA76801 BAA76801.2 Homo sapiens (human) KIAA0957 protein
AGUGAAAGGGUAGGAACGGGGCCAUGUUACACAUCUCGACUAUGCGGGACUUGAGUACCCCACCC
CCACCCCUACU
>EMBL_CDS:BAA83592 BAA83592.1 Homo sapiens (human) BEC2
GCGGUGAGAGCCGGAGACACUACCUACCGUCGCAGAACAGACUCCUCCUCGGCUCCGAAAGACUC
GCCCCGACCCUCUCCGC
>EMBL_CDS:BAA34513 BAA34513.2 Homo sapiens (human) KIAA0793 protein
GAGGCGGUCGUAGUGACUCCUGUCCCGAGUCCUCACCCCUACUCGGGACAAGUCGACCCGGGUUU
CCGUGGACUUUACGUCCCGAGUCUGAGUCCCCCGAGAAGGCCGACCUCCCUC
>EMBL_CDS:BAA34482 BAA34482.2 Homo sapiens (human) KIAA0762 protein
AGACCGAUGAACUCACUACCUGGGUGGGAGACCCAGUAUCUUUCCUGAGACUCCUACCAGGUCCG
ACCAGUCCCCGGCCU
>EMBL_CDS:BAA77569 BAA77569.1 Homo sapiens (human) neuroD-related factor
ACGCUCUCAACUCGACGUCUGUCGGCCGGUGGUCUAACCACCACCCGACGCUGUCUGGGAACGU
>EMBL_CDS:CAB38381 CAB38381.2 Homo sapiens (human) immunoglobulin heavy chain variable
AGAGGGAGUACUGGACACGUGGACUAAGUGAGGUCAGGGAAGGGACCCCUCGACUCCCUAGGUCG
AGGUCGUCCUUCGUGACCAACACUACCUCUUCGUCUCUGUCGUGUCUACUCCCUCU
>EMBL_CDS:BAA76791 BAA76791.3 Homo sapiens (human) KIAA0947 protein
CUUUCAGAGCUCAGGUCUUCAACAGUGAAAUAAAUGAAGGGUACAUAAGUCUCGAACCUAUCGAA
GUACACUAAACCCGAAAA
>EMBL_CDS:BAA86871 BAA86871.1 Homo sapiens (human) Maf-related DNA binding protein
UGCGCCGUCGACCGCCGCAUCGGUGCCAAAAACUCACACGCCGCGGCGACGAACUCGGCACA
>EMBL_CDS:BAA75063 BAA75063.1 Homo sapiens (human) apg-1
AUCAACAGAUGUAUCCGAUGAGACCUAGCCAUCUAGUUAUGAAAACUGAAAAGGAAAAAAUUCAC
AGACUAAAUUAGCCAGAACAAACAGACUCUGUCGAC

>EMBL_CDS:BAB71953 BAB71953.1 Homo sapiens (human) male enhanced antigen-2
CGGCGGAGGUUCUACUAGAAGAGCAAGACGUCCUCCGUCUAGCACAGGAGGGACCUCCGCCG
>EMBL_CDS:BAA95162 BAA95162.1 Homo sapiens (human) vascular cadherin-2
CGGAGGGACACCCCGACGGACGUUGGAAGUCUCCGGACCUUGCACCGACCGGACACCGUCCGACC
CCGAGCCCUUCCAAGUCCAAGAGGGCCCUCCG
>EMBL_CDS:BAA86899 BAA86899.1 Homo sapiens (human) RECQL4 helicase
GAGAAGUCGGUGUCGGUCCACGACACGGUGCAGUGACCGUCACGCCGCACACCGACACCGACACU
C
>EMBL_CDS:BAA76854 BAA76854.1 Homo sapiens (human) KIAA1010 protein
CUCCGGGACCCGCAUGACCACCUUGAACCCGUCGAGUAGGUCGUCGACACGUAAGUCCCGGAG
>EMBL_CDS:BAB19280 BAB19280.1 Homo sapiens (human) protein tyrosine phosphatase HD-PUP
CGUCCGGGUCCACGAUGCCCGUAACGUACCCCGGGACCCCGGGACCCGACAUAGUCGACCCACCC
CGGGCUCAUCCAUGGUCCCCCGUUCCCCGGACUCUCUAUCACCCCAGGACCCGGACA
>EMBL_CDS:BAA86961 BAA86961.1 Homo sapiens (human) hypothetical protein
CCACUCUGUGGGGUUGUUUGAGACGAAGGUUGACCAUACACUCACGUUCGUGAACCUACAGGCAC
CGUCUCUCCGACCCCAUCAGAGGA
>EMBL_CDS:BAA78033 BAA78033.1 Homo sapiens (human) sodium channel
UAUGUUCAAAAGGGUUAUCAGGACCCAGUAUUCCGCUUUAUUACGGUUCUUCCGGGUCGAUUUUC
ACAGUUUUGAACACA
>EMBL_CDS:BAA86559 BAA86559.2 Homo sapiens (human) KIAA1245 protein
AGUAGUGAAAGUAAAAGACCCUAUUACACAUGUCGUAGGAGGGUAAGUUGUACUCUCCUACU
>EMBL_CDS:BAA86582 BAA86582.1 Homo sapiens (human) KIAA1268 protein
CUAGUGCACCACCAUCCCGUCGAAAGGACGGAACCUCUACCACCGUAACGGGCCAUCCUCAGACG
GGAGAGAGAAGUGAUAGACCAG
>EMBL_CDS:BAA86534 BAA86534.1 Homo sapiens (human) KIAA1220 protein
CCUGUCACGGUAUUCUGUAAAGGUGAACAAACCUGUCCAGGACCUUUCAGGAAUACCCCAACAGG
>EMBL_CDS:BAA83041 BAA83041.2 Homo sapiens (human) KIAA1089 protein
UCUUCCAGGCCCUCCUUUCGUAUAGGUGAGAGUAUGAGGAGGAGUAGGAGUGACCGGUCCGACUC
CAACCCACUCCUCCCGUCCAUCUUCUCCGUCUCCAACUUCAGGAGGUCCUGACUGA
>EMBL_CDS:BAA86524 BAA86524.1 Homo sapiens (human) KIAA1210 protein
CCGAGGUGCCUUAGCGAGACAAAAUAUCUUCACGGGUGACUUGACCACCCCUGAACAACGACUUG
UCAUCCUAAGUAUUACCCGA
>EMBL_CDS:BAA86560 BAA86560.1 Homo sapiens (human) KIAA1246 protein
ACGUCACGCCGACCACGACAACGACUCCACACCGUCGACCUGCUACCUCUGGAGGUGGUACCGGC
ACCGGAGAGGCCGUCGUAACCGUCGUUACGU
>EMBL_CDS:BAA86545 BAA86545.2 Homo sapiens (human) KIAA1231 protein
UCCGAGCGGGACCGGGCCCACUCGUGGCCGGCCAACCCCUAGCCCACGUUCCCCGGCACGAAGCC
UGUGUGACUUCGUACGCGCAAUAACGUGAGGAGUGUGGUGUUCAGCACACCCAGCUUCCCGGCCC
CGCCCAGA
>EMBL_CDS:BAA86526 BAA86526.2 Homo sapiens (human) KIAA1212 protein

AUCCUCCUCUACCCAGUAUUUAAACAUAACUAGGUAUUAAAAGAGAAGAUCAAAGAGGAAGAC
>EMBL_CDS:BAA86437 BAA86437.2 Homo sapiens (human) KIAA1123 protein
CCGAGGUGACAGACGAAAGUUCGUUCCCGACCUCUAGAACGUUCUCGAAACCGUCCGUGGACAUG
AGACACAGAGGUCGUCGAACACGUACCUCGA
>EMBL_CDS:BAA86592 BAA86592.1 Homo sapiens (human) KIAA1278 protein
GCACCCAGAAACCACUCGUCAGGGACGUCCUUCAAGAAUUUCGUCCCUGACUACCAACUCCCGGU
CGC
>EMBL_CDS:BAA83014 BAA83014.2 Homo sapiens (human) KIAA1062 protein
UCGGGGGAAGAACCGCCGGAGCGACCCGUCCUACAUCCUCUCGAGGCACGAACACAGACUCUGGU
CGUCCGUCCUCCGGUGUACGAACGCCUACUUGACCCUGUGGACCUCGAGCCUCGUCGACGAGUCG
CCCCGGGCUGGACCCCCCGA
>EMBL_CDS:BAA86561 BAA86561.2 Homo sapiens (human) KIAA1247 protein
GGCGAACCACUCCAAAGCCCCGACCCGCCGUAGUGGGUCCGGAUGCACCAUGUGGGACGGCAGGU
GGAGCUACCGGUGACUCGCC
>EMBL_CDS:BAA83045 BAA83045.2 Homo sapiens (human) KIAA1093 protein
GCCGUGACGGUGGGAAAACCGUAACAACGGUGGUAACUGACCACCGCUACCUCUCGACGGUUAAC
CCGACCGCCGGC
>EMBL_CDS:BAA82978 BAA82978.1 Homo sapiens (human) KIAA1026 protein
CGGCCCUCACCGUCCCCGAGUGGGACUGGCGAACGGCCAGGCGUCCCACUCCCCCCACACUCACC
AUCUCUCAGACAACUGAGACGGCCUACCGACGCCAGUCGCCCUCCAGGCGGACGUGGUGGGUGAG
GACCA
>EMBL_CDS:BAA86054 BAA86054.1 Homo sapiens (human) fatty acid coenzyme A ligase 5
CGGACUACUAUGGGAAAACUUCGAGAAACUUAAAUGACCUUUGUCGGUCGAAGUUGUUCUUGAAG
AAGUUCCCACAGAACCG
>EMBL_CDS:BAA92330 BAA92330.1 Homo sapiens (human) F-box and WD-repeats protein
CGGUCAAUCUAAGAUAUCAGAGAUAUAGGACCUAUUAGAAACCCAUAUUACUGGAUAUUUUACUU
AAACCUCCCG
>EMBL_CDS:BAA83019 BAA83019.1 Homo sapiens (human) KIAA1067 protein
GGUCGACGCGACCAUCCAGACCUAGACGACGAGUUACACGAGGGCCAUCCUCGCGAGUCGUCAGA
AGACACAGUGACGGUGGUCGACC
>EMBL_CDS:BAA83063 BAA83063.1 Homo sapiens (human) KIAA1111 protein
CGAAGAAAGGAACAGGUCCGAGAUUUCCGGUUCAAGUUCCGAAACGGUGGUACCUGGUCCAUCCU
CCG
>EMBL_CDS:BAA89345 BAA89345.1 Homo sapiens (human) hMYHgamma4
GGAGGGACCCCCCGAACGCCGACCGGAAAGACCCCUUCAACUGGUGAGGGUCCCAGACCAGGGUC
CCGAGGCUCCCUCC
>EMBL_CDS:BAA86228 BAA86228.1 Homo sapiens (human) endomucin-2
CAACAACCACACUGUUGUAAAAAGACAUUACUAAGACACAAACCACAACAAUAUCUACCAAAACA
ACAUCAUUGUUG
>EMBL_CDS:BAA88125 BAA88125.1 Homo sapiens (human) breast cancer associated protein
CUCAAGAAUGACCGAAGAGACCACCAAGACCUAAUCCUCUCUGGAGAUCCCUCCAGGAUCCUGAA

>EMBL_CDS:BAA83016 BAA83016.2 Homo sapiens (human) KIAA1064 protein
CCCGGCGGUCCCGGGUACCCACCCGGUCCUAAGUACGGUCCCGGGUAGCCGUACAGACGCACGUA
CAGACGCACGUACAGUCCCACGUACAGGCCCACGUACAGCCCCACGUACAGUCCUACGUAACCUG
GCGGGUAACCGGGACCCCCAGGGUACAACCCGGGUCCCGGGUAACCGGGACCCCCAGG
>EMBL_CDS:BAA82977 BAA82977.2 Homo sapiens (human) KIAA1025 protein
CCCUUCCACGGUUUAGACAAUGGACACAUUCCGAUGACCUCGGUCGAGACGGUCGUCGUAAUAGG
ACGGCCUGUAGACAAAAUCAUCGUCUUGACUUUUAUCUGUACCGUAAAAAGG
>EMBL_CDS:BAA83000 BAA83000.2 Homo sapiens (human) KIAA1048 protein
CUGGGACCCGGACUCAUCUUCCGCAGACGACACCUCACCCUCCUCGGACACCGACGAACCGGACU
CAAACGCCGUCUCCGACCGACCCACCCCGAACCCCAA
>EMBL_CDS:BAA86596 BAA86596.1 Homo sapiens (human) KIAA1282 protein
CCGUCGACGUCCUGGAGGAACACGUCCACGUACCGCUACAGACGCGCGUCGAGCAGUCCCUCCGA
GACGUCGUCGA
>EMBL_CDS:BAA86591 BAA86591.2 Homo sapiens (human) KIAA1277 protein
GUCGUGGCCCUACAUCCACACGACGGUCCGCUUCCCCAUCUAGUCGCGGUGGUAGCGCACCGUCG
UGAACCUGUCCUACGAC
>EMBL_CDS:BAA86516 BAA86516.1 Homo sapiens (human) KIAA1202 protein
CCUCGACAUCCGACUCAGUGCGACCAAUCCCAUGUACAAGACCAGUUAUCCGUUGUCUACCGAGA
GUAUCCACCGAACCGGACCAACGAGG
>EMBL_CDS:BAA86597 BAA86597.1 Homo sapiens (human) KIAA1283 protein
GUCCGUCGUCGGCAGGGGGAACCGUGAGUGUUUGUCGGGGCACCCCUCCGACCCCUAGCGACGAC
AGAC
>EMBL_CDS:BAA86525 BAA86525.1 Homo sapiens (human) KIAA1211 protein
CCGAGUGAACCGAGACCCAGGCCGGAGGGGCGAGCCGCUCUGUCGUCGAGAUGGUUCAUCCCGGA
CGAGUCAAACCUUUCACCCCUACGACCACCCCAGACUGACCACCCGA
>EMBL_CDS:BAA86603 BAA86603.2 Homo sapiens (human) KIAA1289 protein
UCGUUCAUUCAGAGGUACUAGAAAAAGUCCCACAUCAGAAAAUAUUCUUAGACAAAAACUAUCUG
ACCCCAGACAAUAUUGAGAACUAGGGUUAAAUUGUCAGUCUAGAUAACUCGCCAUUGGGACUCCU
UCAAAAGCCAGUAUAUCCUCUCAAUUUUCGA
>EMBL_CDS:BAA89212 BAA89212.1 Homo sapiens (human) bromodomain adjacent to zinc finger
UGACGAAAAAGGAAGGGAACUGAUACGUCCUACCCUCUCCGUCUUCCGUAAUGUCCACAGACUAA
CGGUCCCCAAGAAGAGGGUGAACAUGAGUCUAAAUGUCCACUCCUUUUUCCCCA
>EMBL_CDS:BAA86518 BAA86518.1 Homo sapiens (human) KIAA1204 protein
AGGAGUUAAAGGACCUACUCAGAAGAGAAAGGCCCGACGGGAAACUUUCGUCCUGACCUCAACUG
AGGAGGUCUCCUCAACCUCCC
>EMBL_CDS:BAA86595 BAA86595.2 Homo sapiens (human) KIAA1281 protein
CGCCACCGCCGUCUCCACGACCACGACCAUGAUAACGACAAACCCAACGGCGACGGCGGCGACG
>EMBL_CDS:BAA86489 BAA86489.1 Homo sapiens (human) KIAA1175 protein
ACGACCCCCUCCGUCUCGGGACCUCUAGGCAAAAUGACCUGAAGUACCCUCAAAACUGAAAGAGG
CCGU
>EMBL_CDS:BAA86579 BAA86579.1 Homo sapiens (human) KIAA1265 protein
CAAGAGCGGCACCACCAAUACACGUCGUCGUACCUCUAGUACGAGUACUUACCAUACAUUAGGUA
GUGACACUCUUA

>EMBL_CDS:BAB83129 BAB83129.1 Homo sapiens (human) BDG-29
GGACGUCCCCCGGGUGACCGUAUGACGAGUAACGUCUUUUCCGUUGAAACCACAACCGACACAUG
UCUCGUCAGGCCCCCAAAUAAUAACGUCC
>EMBL_CDS:BAA83004 BAA83004.2 Homo sapiens (human) KIAA1052 protein
AAGAAAACUCGAAGUCGGAGGUCCGACGACUUCACAAAGACGUCAACGACCGAGACUCGGACGUC
GUCUAGGUGAACCCUGAGGUCGAAGUCGAACGCCCGGACGUCGAGUAGUUCUU
>EMBL_CDS:BAA83025 BAA83025.2 Homo sapiens (human) KIAA1073 protein
UAUCUCUCCUAAUCACUUCAGAAGGUCGACCGACAAAUACAUUCUGGUGUCACUGUGUCAGGAAA
AUCCUUCUAAGAGAAAAGGAGAGACA
>EMBL_CDS:BAA86539 BAA86539.2 Homo sapiens (human) KIAA1225 protein
CUGAAAACCGACCAAUUAUCUCCAAAGUUAAUUACCACCAGCGUAGAGGCGGUAAAAGUAGAGAA
GUAGUCUUCUUUCAA
>EMBL_CDS:BAA86446 BAA86446.2 Homo sapiens (human) KIAA1132 protein
GACCGCGAGGGCCGAGUGCCACCGCCGACGCAACGACCGCUACGUACAUAUCAACGGCAACACGA
ACUCCCUCUGCGAUC
>EMBL_CDS:BAA82664 BAA82664.1 Homo sapiens (human) serine/threonine phosphatase 1
AGACAACUACGACCGUGUAAGUACCAAGGGAGACUCUUCUUUUUUUAAGAGUCCUAUAAAAUAAA
ACAUCCGGUCAUUCUCCGUCU
>EMBL_CDS:BAA83068 BAA83068.2 Homo sapiens (human) KIAA1116 protein
GAAAAAGAAGUCCGAGACGGACAAAUCAACCUCGUAAUUUUUGUAAAGGUCGACCGUCUCGCCUU
CAUCGUUC
>EMBL_CDS:BAA92681 BAA92681.1 Homo sapiens (human) KIAA1443 protein
UACGAGGUGACGGUCCACAGUAGUCCCUGAGGAAAUUAAAGACCACUGAGACACCGUCACCACCG
UA
>EMBL_CDS:BAA87415 BAA87415.1 Homo sapiens (human) cadherin-7
AGUAUCAGGUACCGUUUAAGUCCCCGCAAUAGUAACUACAGUUCAUAUCACUACCGGUGUAUCGG
AGAAGGAUGAACUCUACCUAAGACCGAGAGGUAACGUUCCUGACACU
>EMBL_CDS:BAA96028 BAA96028.1 Homo sapiens (human) KIAA1504 protein
ACCUCCACAGUAAAAGGGUGAGAAGGUACCGAGAACAUCCAAUUGACAUCUUCAAUUAUAUCUAC
CCUCUGCGGAGGU
>EMBL_CDS:BAA96063 BAA96063.1 Homo sapiens (human) KIAA1539 protein
GCGGGGUACCCUGGCGUCAGACAACCCAGGUUUUGUCCGAGAAACCCGGGUCCGUCGUCCACCGA
GGUCUUCCCAC
>EMBL_CDS:BAA96051 BAA96051.1 Homo sapiens (human) KIAA1527 protein
AGUCUUCUGGUUCUUCGUCUUGCUAUACUCCCAGACCUCACUCGUCUUCAUAAAGACCCAGGGUA
UAAAGACUGAAGGACCUAUGGAAGACC
>EMBL_CDS:BAA92540 BAA92540.3 Homo sapiens (human) KIAA1302 protein
AACGCGACCGCGGUGAGGUGGUCCAACUCCGGCAACAGCUACCCGUCGCACUGUAAGGAGAACGG
GUGCCACCCCAACUGCCACGGUCGGUCGUU
>EMBL_CDS:BAD74065 BAD74065.1 Homo sapiens (human) histone protein
CAGGAAGAAGACGCGGAAUCAGUGCCGAAAGAAGCUCGGGAAGAACCCGCGCCCUCGCCUG
>EMBL_CDS:BAA95992 BAA95992.1 Homo sapiens (human) KIAA1468 protein
CGGAAUAGAAGGUAAUUGUAAACAACGUUGUAACUUUCUUGGUUCCUCGAUGCCUAAAGAAAUCC

UUCCAUUCCA
>EMBL_CDS:BAA92641 BAA92641.1 Homo sapiens (human) KIAA1403 protein
ACGGAGGAGAGGUACCACCACGUCUGGGUCCACCCACCCGUCCAGGUGCAGUCCCCGGUCAUCAA
GAGGGACUUCUGACUUGGCUCGGACGUAGGAGAGGACACAGACGACGGCGGCCUGUCCCCCGU
>EMBL_CDS:BAA96094 BAA96094.1 Homo sapiens (human) intelectin
AGACCAUCUACGGUUUGUCUAAUACAGGGUCACAGACCUCCUUCGGUCACAGGCACGCCAUGGAG
UCGUCCCUCGACAAAGAGGUCACGACGUACCCCCUGAAUAACCCGUGCACGGUCU
>EMBL_CDS:BAA95984 BAA95984.1 Homo sapiens (human) KIAA1460 protein
CUAAGACCCCUACCAAGGAGGGUCGGACACCCAAGAAGAAGAAAACGACCCCGACCAUAUCCAGG
GGGGUCGGUCGGACAUGGUCCUCCAAACGGACAUCGACCACCCUAAGGGGUUCUAG
>EMBL_CDS:BAA96027 BAA96027.2 Homo sapiens (human) KIAA1503 protein
CUAGAAGUCCGGGACCGGAGGAAGGUACAAGAAUUAGGUAAAGUCCCGGACCCGGACUCCCAG
>EMBL_CDS:BAA95996 BAA95996.1 Homo sapiens (human) KIAA1472 protein
AGGCGGGUACUCCUGGAGCUAACGCACUUCUUCUUCUGGCGAACGAAGGGCGUCAGCUGUAUACC
UGGAGUCCUCGCCU
>EMBL_CDS:BAA92624 BAA92624.1 Homo sapiens (human) KIAA1386 protein
GAGAUCAAAGAAGUAGCUCCCGGUCGGGUCUGAGAACGUCACUAUUGACAUAACGUUCUUCAUAU
AACCCUAGCAGAAGUAGGUUCCUUGAGUCUC
>EMBL_CDS:BAA92654 BAA92654.1 Homo sapiens (human) KIAA1416 protein
GUGAAGGAGAGCUCCGGCGGCAGACGCGGCCAGUCAGAAGUAAUCCCGGAGAACGGAGUAGACAA
CGGAAAAUAUCGACGCGACUAUCCGUCAUUACUCGGCCGCGUCUGCACAUCAGUCCCAACUUCAC
>EMBL_CDS:BAA92671 BAA92671.1 Homo sapiens (human) KIAA1433 protein
AGCCCGGUCGACGAACCGCCGUCUCAACAGAUACUCCCACCCCCUAUUUAGGGACCUACCUCCCG
AGACAUCCGGUGACCGAACAACCAGGACU
>EMBL_CDS:BAA92626 BAA92626.1 Homo sapiens (human) KIAA1388 protein
AAGUGGUCCGGUGACACCCUCGACACCACGACACGGUCACUCCACUCAUCACUCUUCGGGUAGAA
CGUCUGGGGUGUCUGCUUCCCGAAGACGAGUCGGACGUGCGUGACCACGAGGUCGUCCAGUCACC
GGACCACCU
>EMBL_CDS:BAA96031 BAA96031.1 Homo sapiens (human) KIAA1507 protein
AGUCCUACUUCAUCAUAAAACACCAACUGAGGAAGUACCUCAUGUCGUUAAACAUAAGGAAGAAU
GACU
>EMBL_CDS:BAA92554 BAA92554.1 Homo sapiens (human) KIAA1316 protein
CUCCCCACCUUCAUUUUUACAUCUACGACGGUGGACUUAUAGUCCCCGUCAGUUCAGUCGUCGGU
CGAGAG
>EMBL_CDS:BAB16102 BAB16102.1 Homo sapiens (human) hypothetical protein
CGUAUGGUUCUGAACCCGAGAGGUUUCACCUCUAUUAAUUACGAUAUCUCCAGGUACAGAAGCCA
CACG
>EMBL_CDS:BAB17049 BAB17049.1 Homo sapiens (human) interferon-responsive finger
UCUAGACCUCGUACCUUAUGUCAGAAGUCAAAGAACCUUUGGUAAAAACCGAAAAAGUCGGAGGU
CUAGA
>EMBL_CDS:BAA92659 BAA92659.1 Homo sapiens (human) KIAA1421 protein
CGUCUCCGACUAUAACCGACCUUUUACAUAACUAUUAAACCCCCAUAUAAAGGUGAACAUUCUUA

AAGUAAAAAGUCGACUAGGUUUUAGACA
>EMBL_CDS:BAA92636 BAA92636.2 Homo sapiens (human) KIAA1398 protein
ACGGGACUCAUCACGGUCACCGACCACACAACGGGAACCGUGGGUGACCCCCGUGGUGGUGGUAG
CCGUGGAGGAACCCUCGU
>EMBL_CDS:BAA94075 BAA94075.1 Homo sapiens (human) neurexin II
CCCACCACGACAGGGAGUCACACCCCCUCGCCGGGGCCGCGCACCACCAUCACCGGUACCACCAC
CAUCAGAGGUACUACCACCACCGGUACAGUCGGUCGUCCCACCACCGGCACCAGAGGCGUGACUC
GUCGUGCCUGCCGGGGAGGG
>EMBL_CDS:BAA92662 BAA92662.2 Homo sapiens (human) KIAA1424 protein
CAAAGAAACAAAAGGAGGAGACAAAGGUAAAAGAAGUAGGAAUAUACUUUUAACUUAACGUCUGA
GACCCCCUAUUUGUUAUUUUA
>EMBL_CDS:BAA92655 BAA92655.2 Homo sapiens (human) KIAA1417 protein
AAGUCUCAGACUACAGUUGUCAAGACCUUCUCUAAGGUGUCUUAACCAGAAUGGUCUGAAAUUAU
UAGGACAUCUACUACCUGAGACUU
>EMBL_CDS:BAA92546 BAA92546.1 Homo sapiens (human) KIAA1308 protein
GUCGACCGAGACCUCGGACGACCUCGAGGCCUCGACCACGACCUCGAUGAAGAUCGAGACUUGGA
CCUCGACCACAACCUCGACCGAGACCGAGGCCCCGACCUCGGCCGUGACCCGACCCAC
>EMBL_CDS:BAA94480 BAA94480.1 Homo sapiens (human) muscarinic acetylcholine receptor m3
GUCGUCGUAGUAACAACAAGGUUGACAGUGACGACGACACCAGAACCAGGUAGACGAGCCUCGAC
CCAAAGGUCGAGAACCAACACUUGGUCUUCACCGUCGCCGGUAUGAAGGAGGACAACCUCGCAAA
GUACGAAACGACAAC
>EMBL_CDS:BAA92615 BAA92615.1 Homo sapiens (human) KIAA1377 protein
AGAGAAAACUUACGAGGUCUUCACCUCAGUCAACAGGAGUAUCACGAUCAAAGUCUCCAGAAAAG
UGUAAAAAAUAGAUCAUGUUUGUACCCACCCGAUCUAUGUUUCCCAUGACUUAAUAAUCGUCAUA
AACACCGAGAAAGGUGACAAGGUAAGUUUCCUCU
>EMBL_CDS:BAB16413 BAB16413.1 Homo sapiens (human) X11L-binding protein 51
ACACGUUCAGUCCGGGGAACCGGUCCAGGAGAAGUGCGUCCCCAAGGUCGGCCCCCCGGACACUC
CCGUGU
>EMBL_CDS:BAA94194 BAA94194.1 Homo sapiens (human) protein kinase PAK5
GACCCCAGAGGACCCGACUGGUGGUCGACGUCCCGGCGGGCUUUGACAAGUACCCUGUGGGACCU
CCCGACGACCAGCCUCCUCCUCGGGGUC
>EMBL_CDS:BAB83623 BAB83623.1 Homo sapiens (human) galectin-9
CGAGACGUGACACACCUACUGACAGACCCAGACCUUCGACUACAUCCUGUCGACGUGUCUCGGUA
ACUGCCUCUACCACAGGUGUGCCACCUUCCCG
>EMBL_CDS:BAA92556 BAA92556.1 Homo sapiens (human) KIAA1318 protein
AAACACCGUACCUGUAUGGGUACGGUGUAGGUCUCCGACACCGAGAACCAUCACCGUACCCGUAG
CUAGGUCUUU
>EMBL_CDS:BAA96025 BAA96025.1 Homo sapiens (human) KIAA1501 protein
CAUCCGACCUGUGGUGACGUAACAACGGCCCGUGAGCCAUUUACGGACACCUGAGGUCGGGAG
>EMBL_CDS:BAA92595 BAA92595.1 Homo sapiens (human) KIAA1357 protein
ACAGUGACGACCACGACGAGAAAGCCUACGAAGAGACUUAGCUGGGGUGAACGAAACCUCUCCGG

UCCGAACCCCGUCGCGGCCACCCACAGU
>EMBL_CDS:BAA96021 BAA96021.1 Homo sapiens (human) KIAA1497 protein
AACUUCAGGGGAAGAACCUAAAACACUUACCCCAAAAACAACUCCAGAUUCUUAAAGUUUAAACC
UUGAAAAACGUCCCGUUCAACUCCCUGAAAUU
>EMBL_CDS:BAA96024 BAA96024.2 Homo sapiens (human) KIAA1500 protein
GUUACUGACCUCCACCUAAAAGUCGUCCUCACUAGUACUACCAUUUAUAGACUAAUAGGUCUACC
AACACCAGGUACAGUCUCUGUCUCUUUAUCUUCGAUAGUCAUUGGUCUACUGGUAGUCAAAGUUU
GUGUCGACUCAUAGACUAAAAGAACAAUAAC
>EMBL_CDS:BAB13394 BAB13394.1 Homo sapiens (human) KIAA1568 protein
CACUGUGACAAGAAUCAUUGUAGUCACUGGACGCCAAACCUACCACCGGGACCGUCCAGUGAAUU
UAGUAUCAAAAAUGACUAACAACGAGGUCUGAGACAGUG
>EMBL_CDS:BAB13428 BAB13428.1 Homo sapiens (human) KIAA1602 protein
CGACGGUCCUAGGGGUCCAGCGGCAGGACUGGGGACCGACCCGAAGAACCUACUCAACGGACCCC
AGGACGCCG
>EMBL_CDS:BAB13433 BAB13433.1 Homo sapiens (human) KIAA1607 protein
CCCACUUCGUCCUCUACAACCGUUCUCUUCCCCGUCGUCGUCGAAGACGUGAACCGACUCCGAGA
AACGGCAGAGACUUGUAGCGACGUAGUGGA
>EMBL_CDS:BAB13456 BAB13456.1 Homo sapiens (human) KIAA1630 protein
UCAGAAAGAGUAGGACCGAGACUUCAACCCUCCAAAGUUAUCUUUAGACGGGUGUCAUCUAAACU
AACUCUAUCUGA
>EMBL_CDS:BAB13396 BAB13396.1 Homo sapiens (human) KIAA1570 protein
AGAAAAACCAUACAGAGUUAGUUCCGGAAAAGGAGUCAAAGAUACAAUUAUCUCAGACUUCUUAA
CUAGAGUAGUCCUAUAUGUCCUUCU
>EMBL_CDS:BAB62077 BAB62077.1 Homo sapiens (human) affixin
CGUGCAGUAAUUAGGUCAGCUCCUCCUGGAACUGGUCAAGGAACUUGAACCCCAGAAGGAACCUU
CACCCCAGUUAGUAGCACG
>EMBL_CDS:BAB13460 BAB13460.1 Homo sapiens (human) KIAA1634 protein
AGUCCGCCCCUCUAGGUAAGACACAACUUUACUUCCGGACUCUCGACAGCAGGAGCCCAACAAAA
AGAGGUAUCUUCUAGAAGGCAGACU
>EMBL_CDS:BAE71373 BAE71373.1 Homo sapiens (human) zinc finger protein
CGACGACGACGACAUCGACGGACCCCGGCGUGGUCCCGGAGGAAGGUCCCGAGGUCCGGUCCGA
GACCCGACUUCCGACGUUGACCCCGGGGGUCUUCCUCUCCCGACCACCUGUCCGAACAUUCCCGU
CGCCGUCG
>EMBL_CDS:BAB13392 BAB13392.1 Homo sapiens (human) KIAA1566 protein
AGUCGACACCUGAGGGCACAGUCUCCUUACCCUAGUCCUCGUUAACGACUAGAGUAGAGAAGACA
AAGGUGAGGACAGACGUCUACU
>EMBL_CDS:BAB13399 BAB13399.1 Homo sapiens (human) KIAA1573 protein
GGUGAAAGGUCGGAGACUGACGUGGACGACAACUGUGUGACCACUUCUCGUAGAACGUCCGGUCC
CAGUUAUCGACACAUACAGUGGCCGAGGACCACC
>EMBL_CDS:BAB13458 BAB13458.1 Homo sapiens (human) KIAA1632 protein
CCAGAGGAAACUUAAGUGUCCGAUCUCUUCCCUAAAGGACGAGAGACCUCCAAAAACCUUCCCUC
UGA
>EMBL_CDS:BAB39742 BAB39742.2 Homo sapiens (human) mosaic serine

protease
CCUCUACGGGCCGGACCUCCACAGGGUCGACCUCUACGGACCCGACCUCUACGAGGUCGACUUCC
ACAAGAACGACCUCUACGUAAGGGCACCGACAGGGAGAGG
>EMBL_CDS:BAB17658 BAB17658.1 Homo sapiens (human) gamma2-COP
CUAAAGUCUGUGUUUUCUUCUAUAGACGAAGUACUCGGCCAGGUGUGACGAGAGUGAAGGACAAA
ACUCCUCACAUCAUUACCGAUUCCAUCGUUACGAAGACAAACUCAGACACUAA
>EMBL_CDS:BAB39854 BAB39854.1 Homo sapiens (human) VUS20631
GACUGUGGACACGUCCGACCCGUCGAGGAGUUAAACUAACACUCUGUCAAGGUCCUGAGCCUCGG
ACCCGUCGACAACCGUGUAGGGGCUACCCUCGUCGGCCUACGGACGCGCCCAGUCCCAGUC
>EMBL_CDS:BAB59010 BAB59010.1 Homo sapiens (human) calmin
CCUUUGGAAACUCUCCCCACCCCUCCCGAACUUUAGGUCCGGAAGAAGCGACACUGAGGGUUCCC
AGAGG
>EMBL_CDS:BAA99542 BAA99542.1 Homo sapiens (human) 3alpha-hydroxysteroid dehydrogenase
CUCGUGACACCCGUUGGUCUUGUUACAGAAAACUGAACGUCUUUAGGUCGUCAAACGAGACCAAC
UCCAUUCCUACUGUAAGAUGGACCAACGUCUGUCCGAA
>EMBL_CDS:BAB13377 BAB13377.1 Homo sapiens (human) KIAA1551 protein
ACGGACCCAGUAGAGUUGUCGACUACAGAAACUUCACUCUUGAAAGUGUAGUACUCUACCCAGAC
CGU
>EMBL_CDS:BAB13442 BAB13442.1 Homo sapiens (human) KIAA1616 protein
AGACCAAGAAGACGGAGGAAGAGAACCUCCACCUAAAGGACCCGGACUCUGUAUAUCCCGUACAG
CAGAAACAAGAACUUGAGGUUCGACUCUUAACUUCCUCCUUCAUCUUAAGGGUCU
>EMBL_CDS:BAB39380 BAB39380.1 Homo sapiens (human) MAP/microtubule affinity-regulating
CGACACUGACCUCCCCCUCCGUCCCCCGUGGGCACCCCACGGACUCGACAAAAGAAAGGGUAAAC
CGUUGUCA
>EMBL_CDS:BAD74069 BAD74069.1 Homo sapiens (human) hypothetical protein
CCGAGGAAGAGGUUCAGGGUCCUCAGAUCGAGCAGGAGGACCCCGAACCCCCGUUCCCUACGG
>EMBL_CDS:BAB86289 BAB86289.1 Homo sapiens (human) CasL interacting molecule MICAL
AGCGACGGAAGGAAAAACACCAGACAGACCCCGUCCACGACCUCCGUCAUCUUUACAGGUAGAGG
ACCCACGACGAGUAUCGGUGGACCGGUGUCACACCGGAGUGUCCAUACCGUCGCC
>EMBL_CDS:BAB87181 BAB87181.1 Homo sapiens (human) psi neuronal apoptosis inhibitory
UCACCAAAAAAACUUAGUCAAAAGGAUACUGAACUCCCUAACUUGUAUAAAGAAACAUCAGUAGA
UUUUCCUUAUUCUGGACUAAGAAAUUGACGA
>EMBL_CDS:BAB13469 BAB13469.1 Homo sapiens (human) KIAA1643 protein
AGUGACCGUAUCAAGCAGACGUCGACCAGUCGGUGUGGACUUUACGUCUGCUUGUCCGCCACC
>EMBL_CDS:BAB19259 BAB19259.1 Homo sapiens (human) toll-like receptor 9
CACGUAACGGCGACUUCAGGUCCCGGGCGUCGCUGCAUGACGUCUCGACGACCCUGUGAACCGAC
ACCUACAACAACACCCGGUCCGACUCCACCGCGUCCCACGCG
>EMBL_CDS:BAB21774 BAB21774.1 Homo sapiens (human) KIAA1683 protein

GUAGAGUCAUCCGACUCUACUCAUCAGACCCGAACCCCUGUGAAGUCACCGGUGUCUAUCCAUCG
GACUCGAACCCCUGUGGAGUCACUCGACUCUACCCAUCGGACCCGAACCCCUGUGGAGUCACCGG
UGUCUAUCCAUCGGACUCGAACCCCUAUAGAGUCACCCGACUAUAC
>EMBL_CDS:BAB60824 BAB60824.1 Homo sapiens (human) Gi-coupled ADP receptor HORK3
AGUGUAAGAACAGAGAGCCGACGGACAACCAGUCUUAGUACAAUCCGUUUCUCUCAUUCUUGUAC
U
>EMBL_CDS:BAB21853 BAB21853.1 Homo sapiens (human) KIAA1762 protein
UUCUCUCCCCGACCCUUAGCCACGUCCAGGUUCCCUCCACCCCCUCCCCUGUCCUUUCCGUUAGG
UCUAUACUUCGGUGACCACAGUCCCGGGAAGAAGAGUCACGGGUGGAGGGGGAGACAGAGCGGAA
GGAACCCGAGAAGAACCGACGCAAGACCGGGAAGUUAGAA
>EMBL_CDS:BAB21813 BAB21813.2 Homo sapiens (human) KIAA1722 protein
UCGAAAAACCGAAAAUACGUCGAAUCCACCAGAUAAAGUAGAUCUAAUCCAUAUUCUCGAAGUUU
UCGA
>EMBL_CDS:BAB21776 BAB21776.2 Homo sapiens (human) KIAA1685 protein
ACCAGGGAGGUUGACUCCGCCGACGUCGUCGACGCCGCCGUCGCCGUCGACGACGGGAGACACGA
AACUGGU
>EMBL_CDS:BAB21777 BAB21777.2 Homo sapiens (human) KIAA1686 protein
UCCUUUCACCCUUGGAAAUUGUUGACUUCAAAGAUAGAGACUUUGUCCAAGACUAAAAAGA
>EMBL_CDS:BAC15606 BAC15606.1 Homo sapiens (human) FELE-1
ACCACAUCCGUCACCGUCGGAGUUAGACCCCGUGGUUGGGUACCACGACGUCGUUAAGGGCCUUC
UCCGACUUCCGUCCGUGGU
>EMBL_CDS:BAB21802 BAB21802.1 Homo sapiens (human) KIAA1711 protein
ACAAGACUUUAUGUACAUUAACGUAAGUCUCCGAAAUCUUCCGACCUAAAACAUUAAAACACACC
UUCUCGU
>EMBL_CDS:BAB47408 BAB47408.2 Homo sapiens (human) fibrillin3
CGGUGGUGACUGUUACGUGAGCAGGUGUAGGUGCGUACGGACGGACCACCCGCACCGGACCUUCG
GGCCCAUCGUGGCCGUCACCAUCCACGGCCCCUACAACUGCGUCAGUGGCACCACCG
>EMBL_CDS:BAB40803 BAB40803.1 Homo sapiens (human) epiplakin 1
CCCGGACGGCUCACGACAUGAACUACUUACCCCCAGGGACCCCGAGGGGAGGGGCCCCAGGGGGA
CUCUCACCGUCCUUCCGUUCGGG
>EMBL_CDS:BAB21818 BAB21818.1 Homo sapiens (human) KIAA1727 protein
CCCGCGCCGUCCAGAAUAAAAGGAGUCCGAAGACAGACCCCUCCAAGGACUCACCAAACCGUGAC
CCGAAGAACUUUCUUGGGGACCCGACGUCCCCUUGCAGGGAGGGGCCUGCCCACGACGACCUCGG
CGACGAGGG
>EMBL_CDS:BAD93351 BAD93351.1 Homo sapiens (human) BNIP2 motif containing molecule at
GGAAGGAGAAAUCGUUUACCGAACACAAGUCAAAGGAAAAGCAGAGGUCGUUUACAACCACUACA
CAUUCUCAGACUCCACCUUAUUAACCAUAGAUUCCCCCUUCC
>EMBL_CDS:BAB33339 BAB33339.1 Homo sapiens (human) KIAA1669 protein
GGACACCGGACCUCCCGACUCACGACCGACGACGCCCACGGAGGGGUCCUCGACUCCCCCCGUCC
AGGUUCAAUUACCAGACGUCGCGGUGGAGGUCCUCUCCCUGGUACGGUCAGACGUGCCUGCCGAG
UCGGGGACCCGGGUGUCC
>EMBL_CDS:BAB20900 BAB20900.1 Homo sapiens (human) KF-1 protein

GUCCUCAAGAUUCAUACAUACUACCGUAUAAUAUAUACGGUUAUAGACAGUAUAUUGAGAAC
>EMBL_CDS:BAB39359 BAB39359.1 Homo sapiens (human) HsRma1
CCUUCGGGGGCCAUAGUGGUUUACCGACCUUAGGGGGAGACGAGAGGCCUCGACCAGAGACCGGG
ACCCCCGCCCCACCUCAAAAAUUAGACCCUAGGACCCCGAAGA
>EMBL_CDS:BAB69021 BAB69021.1 Homo sapiens (human) hypothetical protein
AUACUUGUCUUAAAAGACGAACUCCUUCGACGCACUUAUGUACAAGAAAGGAAUCAAAGAAUUCG
UCAUAUAGACAUUCAU
>EMBL_CDS:BAB21780 BAB21780.3 Homo sapiens (human) KIAA1689 protein
GUACUCAGACACUUCUUUAGAACUUUACUGACUUCACUGAACUAUGAAGAAAAAGUCUUAAAGAA
AAAAGAAGAUGUCCACAUAC
>EMBL_CDS:BAB59128 BAB59128.1 Homo sapiens (human) chorein
UAACACAACAUUACUGAUUUCGUCCUCGUAGUAAAAAACAGUACAGUCGAUGUUUGUGUUA
>EMBL_CDS:BAB21863 BAB21863.1 Homo sapiens (human) KIAA1772 protein
AUGUCAAAGGGGACGUCCUUACCCGGACCCAACAUUUUAAGGUCAAUCACCCCAGACACCUUGAC
CCCGUCUAUUUUCCCCGAGGCAAGAACUUUCUCCCGCUUGACAU
>EMBL_CDS:BAB21770 BAB21770.1 Homo sapiens (human) KIAA1679 protein
GACAUAGCUCUGACAAAAUGACACCGUAAAGGACGAACCGUGUAGGAACCCGUCCCACAGGUGAG
UGACUUUCGUCAGUGUUAUGUC
>EMBL_CDS:BAB21846 BAB21846.1 Homo sapiens (human) KIAA1755 protein
CGUCUUCGAAGACCGUUCCGUCGGAAAAGGAGUCAGUCACCCGACCCCCGUCACCGACCCGGUCU
ACAAAACCA
>EMBL_CDS:BAB32867 BAB32867.1 Homo sapiens (human) NeshBP
AGACUAAACAGUAAACUUACCUGAACAAGAAGGGUGACUUACUGGGUAAAAGAGACCUAUAUUGA
AGUAUAAGUCACUGUCACAGUAAAUCACCGAAAAGGGUCAGGUUCUACUGUUUACUCC
>EMBL_CDS:BAB21816 BAB21816.1 Homo sapiens (human) KIAA1725 protein
ACUGGUCAAGACUUUAACGGAAACUGACAAGGAGUAGGUAACUUCUAAGAAGACUACGUACCCCU
CAUAAUGAGUUUCUCGACGAAGAGACCAGU
>EMBL_CDS:BAB21827 BAB21827.1 Homo sapiens (human) KIAA1736 protein
CGCCUGAUCGUCUCGACGAGUUGAGGUCGGACGACCUCGACCCGCAAACCGGGAGUCAGGCG
>EMBL_CDS:BAB21833 BAB21833.2 Homo sapiens (human) KIAA1742 protein
UCGCCAACCUCUCGUGUCGGUCUAUCAACGGGUCCCUGGUCGACCCCGUCGUGACCAACCGACGA
CAGGCGA
>EMBL_CDS:BAB33332 BAB33332.2 Homo sapiens (human) KIAA1662 protein
UCGGGUGUCGUCACCCACAGGGUCUCACGGUCCUAACACUCCUGAGACGCCCCGGAGGGGUCAAG
AGUCCCCCCGAGCACUCACCCGAAGAGAAAGGAUCAAGAGACCCCUCGAGUACUCACCCGA
>EMBL_CDS:BAB21848 BAB21848.1 Homo sapiens (human) KIAA1757 protein
CAUUGUAAACUUUGUCUCAACGAUUGAAGUCUAAGUCCCCGAAGUCAAAAACAGAGAAGUGGUUG
UCCUCAGAGACUCCACCAACAUCAUUAGAGAUGUAGUCUCAACG
>EMBL_CDS:BAB21861 BAB21861.1 Homo sapiens (human) KIAA1770 protein
CUCCUCCACAUCCUUCACCUUCGAGUCGGUCCCUUUGACGACGAAAAAAGAGUCGAAGGAAAAGA
AGAUUAAAGAAGAG
>EMBL_CDS:BAB21850 BAB21850.1 Homo sapiens (human) KIAA1759 protein
GGCCCUCCGGUCAGACGAGUCGGUUAAGUAGGAGAGGUACUCCGUCCGACCCGUCCUGACCGUCG

GUCUCC
>EMBL_CDS:BAC05722 BAC05722.1 Homo sapiens (human) seven transmembrane helix receptor
CAUCUACUACCCCAAGUAACCCCACUAGUGAAGUAUUCUGUUAGGUUACUAAAGUAGUCUACG
>EMBL_CDS:BAB91223 BAB91223.1 Homo sapiens (human) aquaporin 10
CGACCAUUCACCGACACGGUUGCCACCGGGGGUGGUCUCCCCGGUGGUGUCCGUGGGUGGUGGUC
GGUAAUGGUCG
>EMBL_CDS:BAB84316 BAB84316.1 Homo sapiens (human) RIN3
CUCGAGACCCUUACCCGUCACCGGACCCGUCCUCCCACGACUCAGGGUCGGACUCUUCGUCCCGA
AACCGGGAGAGACCCACCUGGGCCGCACCCACAGAGGUUCCGGUAGCGGACACACGUCUCGAG
>EMBL_CDS:BAB40969 BAB40969.1 Homo sapiens (human) kringle-containing transmembrane
GGUCGGGGGUACCCACCGCGGGUCCUUAACGAUGGACCCUGUCAGACUCCACCCACCGACC
>EMBL_CDS:BAB67831 BAB67831.2 Homo sapiens (human) KIAA1938 protein
CGGGGGCGUCAUGGUGUCCGACUCCGGCCCCGGCGAGUGAGACCGCCGAACCGACGGAAACCUAC
AACCCCA
>EMBL_CDS:BAB47435 BAB47435.1 Homo sapiens (human) KIAA1806 protein
CGGGCGUAAGGACCGUACGUAUCCCGAAGAACGGUGACACUCAGGAACGUACAAAGGACUCCCUA
CUCCCG
>EMBL_CDS:BAB67802 BAB67802.2 Homo sapiens (human) KIAA1909 protein
AGUACGAAACCCCCCGAGACCCGUUCCCCGCCAUUCGACCCCAGACCCCGUCGGAGUCUUUCCCG
GAGAAGGUCUGCCUCGGCCCAGACCCCCAUGACGUCGGUGUCCAGAACGACACUACGGGUGCCAA
AGGGGUCCCGUACU
>EMBL_CDS:BAB69067 BAB69067.1 Homo sapiens (human) signal recognition particle 14kD
AGUCGGGUAGGUACAAUCGAGAUUCCUCCAAACUUAUUCGGUAGACUUUGAAUAAGUGAAGGAAC
CUCGAGUGGUGUCACGACU
>EMBL_CDS:BAB47462 BAB47462.1 Homo sapiens (human) KIAA1833 protein
CAGGGGAUCCAUCCCCGACCUUCUGUGUUGGUUGUCAGAAGGCCAAUGUCGUAUCCCUCUG
>EMBL_CDS:BAC01892 BAC01892.1 Homo sapiens (human) immunoglobulin heavy chain VHDJ
CGGAAGGGACCCCCGACUGCCUAGGUCGAGUGUCUGUAAGGUGAUGACGACUCACUCUUGGGUCC
CUUCCA
>EMBL_CDS:BAB47465 BAB47465.1 Homo sapiens (human) KIAA1836 protein
ACGGGACCACGUACUUGACGAACCUCUUGUCGUCCGUGAAGAGAACAACGAGGUCUUCGUAAAAA
ACGUCGACCCGU
>EMBL_CDS:BAB93492 BAB93492.1 Homo sapiens (human) annexin A7
ACGAGGUGGACCACCCUGAGGUUUAGGAACCGGACCUCCUUGAGGCCCUAUCCUACCUCGAGGGG
GACCGACACCCCGU
>EMBL_CDS:BAB47436 BAB47436.1 Homo sapiens (human) KIAA1807 protein
GAUACACUCGACACGAAACCCGUUAUCCUGAACUUGAACUGAAGUAGUAAGAGACGAUUCUACCA
GAAGUAGAGGUCCGGGGCCAGUUUUCGUGUCCAGUGUACC
>EMBL_CDS:BAC05726 BAC05726.1 Homo sapiens (human) seven transmembrane helix receptor

GAGAGGUGGUAGGUCCCCAGAAGAGGAACGAGAUUGACCCUCUACUGGAAACCGAACCUUUUCUC
>EMBL_CDS:BAC06143 BAC06143.1 Homo sapiens (human) seven transmembrane helix receptor
CACGAAAAACAAAAGGGGGUCUACUCCUGUAGAAGGAAUACCUACUUCGUUAAGCACUUAUUCUC
UUUUACUAGGGUUCCUCCCCUUUUUGCGGGUCGUG
>EMBL_CDS:BAC05799 BAC05799.1 Homo sapiens (human) seven transmembrane helix receptor
ACACAACGGAACUCUCCUUUAACGAGUCGUAGAAACCUUACUACUGUCUUCACUCGUCAUAGAGA
UCCGUCCUGU
>EMBL_CDS:BAB93508 BAB93508.1 Homo sapiens (human) OK/SW-CL.108
CAACGACAAGGACUUCUGUUCGAUCUAGGUAGUAUACAGAUUAAACACGAAAGUUUAGAAACAAG
ACCUCCACGUUUACCACUGUUACUUCUAGAAAGAACUCAAGGAAACCUUAUACUG
>EMBL_CDS:BAC05849 BAC05849.1 Homo sapiens (human) seven transmembrane helix receptor
CUUCUCGACCCUGUACGUCGGACGUAUACUUUACCAUAAAAGACUACAAUCGUACCAGGGGUCGU
AGAAG
>EMBL_CDS:BAB47448 BAB47448.1 Homo sapiens (human) KIAA1819 protein
UCGCCCGCCGACGACGAGAGGCAAAUAGGGUGGUGACGGUGGUAAUAACGAUGACAAGUCGUCCA
CGACGACCACCACUACCACUAUCAGACCACCCCCGCCACCCCGACGACAACGACGA
>EMBL_CDS:BAB47484 BAB47484.1 Homo sapiens (human) KIAA1855 protein
CGAGAAGCGGGAGGAGCCCGUCACCGGCGUCGAGGAGCCCGAGGAGUAGGCACCACGGGCUACAC
GACCCGAGUCGGGGACCUCCCUUCAGGAACUCG
>EMBL_CDS:BAB47478 BAB47478.1 Homo sapiens (human) KIAA1849 protein
ACCCACACCGGUCGUCCAACUCCCACGUCCUCUCGAAGACCUUCUUCACGAGAGGUCGGCGGGGC
UUCGGCCGCGAGGCGUAGGU
>EMBL_CDS:BAC01580 BAC01580.1 Homo sapiens (human) immunoglobulin kappa light chain
UUAACCUACGUCGUAUCUAGUCGUCAAAUCCCCGAAAGGGACCAAACACAACUAUGGUUAA
>EMBL_CDS:BAB68399 BAB68399.1 Homo sapiens (human) WNU7B
CGGCGUCUGCCGACCGGGCGUGGUGGAGGUGGACGUGCCGGCGCAACAUGAAGAGGAAGUCGUCC
ACCGGGUGGAGAGCCUUGAACCCGUCGCACCAGGUCGUCCAAAACCACCACGUCCUCGGUCUGUG
CGGCACCG
>EMBL_CDS:BAB47466 BAB47466.1 Homo sapiens (human) KIAA1837 protein
GGGUCCUCCUGGGGUUAGACCGCCUACUUGUAGGGGAAGUCGGAGAGUCAAUCGACUGACUGCAA
CUAUAGGUUCUACUAGAGGUGGUCCAACAAAAAGGACCC
>EMBL_CDS:BAC05715 BAC05715.1 Homo sapiens (human) seven transmembrane helix receptor
CCACACCUCCACCCUCAGUCUCGACUGUCGGCCCUACGACUCGUCCAAGGAGUCGUGACACUGGU
CUAUGUACCUGUCCCUGUCCCUGUCGUUUCGCUCCUGGCCGACGUCAAGACCUAGGAGGCGCGA
>EMBL_CDS:BAB47446 BAB47446.1 Homo sapiens (human) KIAA1817 protein
CGUCGACCCCGAGACGUCUCGGUACCCCAUCCAUCGAGCCCCUUGGUCCGACCAGAGAAGCUCGA
ACGAAGAAAACCCACACGAGAGGUCGACG
>EMBL_CDS:BAC05977 BAC05977.1 Homo sapiens (human) seven transmembrane helix receptor

CCUGGUGUUAUGUCACCCUCCGUGUCCAGACUUUCCGAGACGCGGAGGGUAGACUCCACGCCUAG
G
>EMBL_CDS:BAC01621 BAC01621.1 Homo sapiens (human) immunoglobulin lambda light chain
ACUGAAACCCCCGAAACGGACCCUCGACAACCAUGGUCCUCUGUAUCCAUAUUCAUGGUUCCAGU
>EMBL_CDS:BAB67799 BAB67799.1 Homo sapiens (human) KIAA1906 protein
GGGGGAACUCCAGUGACACUCCAGUGAUCCUAAUAACCCCAGGCCACUGAAGGGAACUCACCCC
>EMBL_CDS:BAB79459 BAB79459.1 Homo sapiens (human) ribosomal protein L8
CGGUGGUGUGGUUGGUGUCGAGACAACCGACUCCUCUAUUGGAAGAACCUCGGCCUCCCGUCGAA
GUGUGCCCAGAAGAACCAGAGUCCCAACACCCUCUAUUGCCACCG
>EMBL_CDS:BAE45748 BAE45748.1 Homo sapiens (human) putative protein product of
AGUCUCCGACUUAGUUUUUGUAGAGGGAAAUUUAUAUACGAAUUCAGUCCCUACAAACUAAAGAA
GAGACU
>EMBL_CDS:BAD90695 BAD90695.1 Homo sapiens (human) CXADR like 1
ACGGGGGAUACAGACCUUCCAACAACUGGUCCGUGACCAUCCACGGUCACAGACUAUUGACUCAC
AAUAAUUACUUCUAUCUCUGUAACCAUCGACCGUACCACGGACAUUUAGGAUGGGAUGGCACCUU
GGCCCCCCGU
>EMBL_CDS:BAB85546 BAB85546.1 Homo sapiens (human) KIAA1960 protein
GGACCGGUCAAGAAACCGGAUUCGGUCAAGGUAUGCCGACCAGUCUCCGUACCUGUGACCGACCC
>EMBL_CDS:BAB85570 BAB85570.1 Homo sapiens (human) KIAA1984 protein
ACCCGAGUGUCUACGGUUCGGGAGCCCAACCCACCACCCUUUGGACGGGUGGCCUACGACCCCAC
UGACCUCGGGU
>EMBL_CDS:BAE45754 BAE45754.1 Homo sapiens (human) putative protein product of Nbla00360
CGAAUCGAGUCCCUGACCGACCAAGUCCUCACCUCUGGAGACAGAUCCGCACCAGAAGUGACGGA
CGUAGUACCACGACAAAGCUCCAUUCG
>EMBL_CDS:BAE45758 BAE45758.1 Homo sapiens (human) putative protein product of
UCUCCCUCGUCCCCCGGCUACAACUACCCGUUUCUUCGGAAGUUUCUAGUGUUUCAACAAGUGGA
CACCCUGACACCAGAGGUCUAACAUCACGAAUAAGUCCUUAAAGUCGUCGAAGACACUCCCAGCC
AGUUAACGGUCAACCACGCCAAGGUGAGCGAGGAAGA
>EMBL_CDS:BAB91138 BAB91138.1 Homo sapiens (human) CLCP1
ACGAGGUGGUACCACCUAGGUCACCAAUAUUACGGACAAUAAAAGAAAAGGAAUAAGUUUAGAUA
AACAUUGGUGACCAUAAGUAGUCACCGUUUUCGU
>EMBL_CDS:BAB85535 BAB85535.1 Homo sapiens (human) KIAA1949 protein
CAACCCCGACCCCCACCCCCUCUGUCUCCCCGACCACCACCCCCGACCUCGAGGUGACGAAGGAG
AAGGACGACAGAGUCCGGAGGUCAGGGUCG
>EMBL_CDS:BAB85544 BAB85544.1 Homo sapiens (human) KIAA1958 protein
GGAAUGAGUCGUGGGUGUCGAAGAGGAACUCAAAGAACCACCUCCUCGACUUCCACGACGACCAC
UACC
>EMBL_CDS:BAB85554 BAB85554.1 Homo sapiens (human) KIAA1968 protein
CCGGGAGACCGUUCCAAAGACGGUCCCGUCACCCGACCCCCGGUAGCCAGAGAGGAAGGGUCCAU

CUUAGUUCCGACAGUGAUCCCACCAGCUCCGAGUGACAGGGUGACGAGGUCCUGGAACGGCCCGA
>EMBL_CDS:BAD38642 BAD38642.1 Homo sapiens (human) putative protein product of
UCCUUACAAGACGGUAGAACCCUUCUGGUGACCAGGGUGAUCACUUCAUCUACCGUCUACGAAGG
A
>EMBL_CDS:BAE45765 BAE45765.1 Homo sapiens (human) putative protein product of Nbla10317
CUCGAACUACAACAGCCGCGCGUCCGACAGGUCGAGUGAGACGUCCCGGACCUCCCACACGACCC
GGUCCGCCAAGAGCCGGAGUUCGAG
>EMBL_CDS:BAE45713 BAE45713.1 Homo sapiens (human) nestin
AGAAGACCUCAGGUGUCACCACGAACUCAAAGACCUCUAAAGUCACAAAGGUCCAAGAGAACAGG
GCGUCUGAAGUCACU
>EMBL_CDS:BAC02434 BAC02434.1 Homo sapiens (human) p53-inducible RING finger protein
CACUACCCUCUGGGUGUAGGAAGAGCCUAACGGUCGACGUACAUGACAAAGUCCGUUCGACACGU
UUUCUACGUGACCGUAAGGACCUCACACCAGUAGAACAGG
>EMBL_CDS:BAD38647 BAD38647.1 Homo sapiens (human) peptidoglycan recognition protein L
GGUCCAAAGGUCGGUCCCACUGACGGUCCUCCGACAGGUGGUACCAGCCACCCCUGAACCGAAAC
CGUAGACCGUUCCUUCGAACC
>EMBL_CDS:BAE45716 BAE45716.1 Homo sapiens (human) putative protein product of
CGAGAGACAUCUGCCCCGACCGACGACCCAGGGACUUCCGAGUCACCGACCGCGUAGGGUCCGUC
CCCCA
>EMBL_CDS:BAB86816 BAB86816.1 Homo sapiens (human) Sialic acid-binding Ig-like lectin
CCUGGCCCUCCCUACACGGCAAGGGCUCCAAAAGGUCCUGACAGGACAAAGGAACCCUUUGGUAG
UGAGAGUCCAAG
>EMBL_CDS:BAB88873 BAB88873.1 Homo sapiens (human) SU7L isoform 3
CUCCUAAUUUAAGGUGUCGAGAUACUUAACGAAGGUGCCGUAAUUAAAGACGACACGAAUUAGGA
G
>EMBL_CDS:BAD06454 BAD06454.1 Homo sapiens (human) Grb2-associated binder 2-like protein
GCCUCCUUCGGAGUAAUAGGCGGAGUCUCGGACACUCCGACGGAGACCACACCGGUACCCUCCGG
UCCGAGGUCCCCUAAGACACCCACGACGGAGAC
>EMBL_CDS:BAB91442 BAB91442.1 Homo sapiens (human) KIAA0781 protein
CUACCUACCGAGUAAAAGAACGAGAACACCUAUCUCUUGUCCAGAGACCUGUCCUUGAAGAUACU
CGUAGGUAA
>EMBL_CDS:BAC16233 BAC16233.1 Homo sapiens (human) L-SOX5 transcript B
AACGUCGAUCGACCCGUCGUAUAUUGACGACGUCAACCUCACCCGGAUUCGGACCACAACGACGC
CGUCGUCGACGGUACCAUCAACCCU
>EMBL_CDS:BAC67014 BAC67014.1 Homo sapiens (human) Formactin2
CGAAGGCCAAGACGGAGACUAAGCCCAAGUCCUAGGGGCCGUGAACGAACCCCUACCACUCCCCA

AGGACCCCUCCUCCGUCUGUGGCACUCUCG
>EMBL_CDS:BAC01115 BAC01115.1 Homo sapiens (human) Di-Ras1
GACGGCCACCACAGACACUAGACGUCGCACGUGUGCGAGAACAGCGUCGACUAGUGGACGGCCAU
C
>EMBL_CDS:BAE96598 BAE96598.1 Homo sapiens (human) zinc-finger homeodomain protein 4
CCAGUGACUAUAUUCCACUGUCGAGAGGACGUCAUUCCUCCCUGUCCAGUAGUGGGUAAUCGACU
GG
>EMBL_CDS:BAC02703 BAC02703.1 Homo sapiens (human) KIAA1994 protein
CAACCACGUUGACCACACUAUGACAGUCUCGAAGGUCAACAUCUCUCAAAAGAUCUAUGACCCGA
ACUCCCACCGGGUGGUCAUUACCUACGUGACCG
>EMBL_CDS:BAC02711 BAC02711.1 Homo sapiens (human) KIAA2002 protein
CGGACUCUCUUCAACGAGUGAUAUUCGGAUCACCCUAUUCUACUCCACCCCGACCCGAGACGUCC
GGGUUUCGGAACCCGUCAGGGGGGUCCGACCAUCACCUGUUCAUCGUCCAAGAGAUCCG
>EMBL_CDS:BAD00088 BAD00088.1 Homo sapiens (human) chimeric MOZ-ASXH2 fusion protein
AUCCUGGACGAAACUCGAGUUGACACGGUAGUCGACGUGUUACUUGUCCACUCCGUGGACUCUAA
UCUCGUCCUGGAC
>EMBL_CDS:BAC67158 BAC67158.1 Homo sapiens (human) cytosolic phospholipase A2 delta
CCCCCUCUUGAGUUGGGUGAGGAACUUCAGGUCACAGAGGUCUAACAAGAGAAACUGUAACUCCG
AGUUCAUCUCCCCGUCUCCCAAGACUGGGG
>EMBL_CDS:BAC76999 BAC76999.1 Homo sapiens (human) homeobox transcription factor Nanog
CCACCAUCCUUCUCAUCUCCGACCCCAUCCAUCCACGACUCCGGAAGACGCAGUGUGGUAACGAU
AAGAAGCCGGUCAACAAAAAGACGGUGG
>EMBL_CDS:BAC45250 BAC45250.1 Homo sapiens (human) receptor protein tyrosine kinase
GAUGACUCUUUACUAGGCAUACCAAUUUCAGAAAACCUACCUCGUGACAGGGUAGGCCGAAGUGG
UCCCAGUAAAGAAGUAAUCAUC
>EMBL_CDS:BAD02449 BAD02449.1 Homo sapiens (human) N-acetylgalactosaminyltransferase
CUUGACCUCCACACCUAGGUGUCGACCUUACAAGGACGACCGGUCUCGGAACCGAUCAAGAGAGG
UCGACGGCAUGGAGGCCAAG
>EMBL_CDS:BAC54922 BAC54922.1 Homo sapiens (human) tripartite motif-containing 15
AUCUAGAAGAGCGGCACGAGGAGCGUCAUUCAAAGAGGGUCGCCCGGGUCCCCGUGUCCCCGGUA
CCCUCAGAGACGGACGAGGAGGAGAACCGUCUCGCCCGUCUCGUCCUAGAACGGGCUCCUAACCC
GGGGGUAGACCCUCUCGCGCCCCUCCGUCUCGGCCGUCUUCCACAC
>EMBL_CDS:BAD89397 BAD89397.1 Homo sapiens (human) carbonic anhydrase VI nirs variant
AUAUGUCACGGCAGUCGGUGACAGUACGCGUACCACCUCCCGUCCGACUAGACGUGACACAC
>EMBL_CDS:BAC23110 BAC23110.2 Homo sapiens (human) KIAA2014 protein
UCGAAGACACUUCUGAACUGCCUUCGCCUGCGGCGUUACUACCGUAACUCAACGCCACACUCGUA

GACGUCCAAUAGGACCUUCAA
>EMBL_CDS:BAC23107 BAC23107.1 Homo sapiens (human) KIAA2011 protein
CCGGUAGACGAGGUUGGACCCCACCCCGACCGCCGGGUACAUGUUCCAAGGCCGACGGUCCAUCC
CGGCUCGGAGGAGACCGCCCCUCACCCAGACUACACCUACGAAGGUCGGCAUUGAAGGCCAACCU
CGGUCCGG
>EMBL_CDS:BAC23124 BAC23124.1 Homo sapiens (human) KIAA2028 protein
AAACAGACUAUGUAAUUGUCGUUGAAAAGAGAUAGUCAGAGAGGUGCUCGAACGUGACUCUUUAU
AUCAGACAAAAACAUACAGUCUGCUU
>EMBL_CDS:BAC82348 BAC82348.1 Homo sapiens (human) scapinin
CCGACCCCUCGACCGGGUGCCUAGAGAGGUCUCCAGACCCUCCUGACAGGUCCCUCAGUCGGGGU
CGA
>EMBL_CDS:BAC98464 BAC98464.1 Homo sapiens (human) GDNF-inducible zinc finger protein 1
UGAGAACGGUGUACAAAGUGUGUAUUUUCCAGAAAGUGGACACACUGUGGAAAAUACUAUUUAGU
CGUACACCAAGACUCA
>EMBL_CDS:BAC23123 BAC23123.2 Homo sapiens (human) KIAA2027 protein
ACGAGAGGACCAAGAAGGAUUAACCCCUCCCACAUCCUAAUCGUCGACUAAGACACGGAGGUGAA
AGAACCCUAAAUCCUAACUCGU
>EMBL_CDS:BAF31273 BAF31273.1 Homo sapiens (human) LUB protein
CAGGAACGACCCGGACACGGGGCCCCAGCCGGCAGAGGCAAUGGUCAGGAGGGACUAGGACCCCG
UGAUUCCGGUCGUGUCGGUCCUG
>EMBL_CDS:BAD89424 BAD89424.1 Homo sapiens (human) interferon regulatory factor 1 nirs
AGUGACCACGACAGGCCGUGUUGAAGGUGACCCUACACGGUCAGCCCCUCUCACGACGACUGUCG
UGUGCCGCU
>EMBL_CDS:BAF31266 BAF31266.1 Homo sapiens (human) KIAA0170 protein
CCUAGAUAGACAAGAAGGGGAUCAUCGGACUCUGCAUCCGAGUCCCCAUUGUCCGACCAGA
>EMBL_CDS:BAD02451 BAD02451.1 Homo sapiens (human) KIAA1199 protein
CAACCUUACACGAGCCAACAAUAUCUUAAAAGGGUCACCUUACACGAGACUUAUUGGACCCCUCA
UGUAAGGGUG
>EMBL_CDS:BAC98376 BAC98376.1 Homo sapiens (human) KIAA2036 protein
GUCCCUUUCUCCGGUCCCGUGUCUCCGUCGACCCACCUCGGACGCCCGUGACACUCUACACAGGA
CGAGGCGAGGACAGGGAAGGACGGGAGAGGGAC
>EMBL_CDS:BAC84969 BAC84969.1 Homo sapiens (human) N-Acetylglucosaminyltransferase IX
CGCAUCGUCCCCAGUCUCCAGGUCGCCCGUGCGUAGGUGAGGUGGAAGGGACUCGUCAGUCCCUU
GAACCCCAGUGACCCACGGAGUGUGACCAGGGCGGCCGGAAGACUGUGGAACGACACG
>EMBL_CDS:BAC78211 BAC78211.1 Homo sapiens (human) hypothetical protein
UCCUUGAAUAGUCAGUACCGCUCCUCCAACGCAUCCCGCUACAGGUCAGCAGAGACCUGGAGCCC
CACCGAGUUCCGGACGAGGCCCCACUGUAGUAUACCCUCUACCGACGCGACGGGACAGACCCGGA
GUCAGACCAACUAGGUCAAGAA
>EMBL_CDS:BAF31253 BAF31253.1 Homo sapiens (human) HLA-G protein
CCGUCCCUUCUGACGAAGGUAGAGUCGUACUCCCCGAGGCCGUCGGGGAGUACGACGUGUACCGU

GCACAUAGAGACGA
>EMBL_CDS:BAC57496 BAC57496.1 Homo sapiens (human) hornerin
ACCCGGUGCCGACCUUCUUGCUGGACUCGGUCUGGGUACAACCGGCACCGACCUCCUCACGGGGU
UUGGCCUGGGUACAGCCGGCGCCGAUCCCCUGACCGGUCUAGGUCUGGGAACAGCCGGCACCGGG
C
>EMBL_CDS:BAC99057 BAC99057.1 Homo sapiens (human) KIAA2038 protein
CGGGAGAACGUAGACGUCGAGGUCGCCCGACGCUCACGACUUCGCCGGGACCGUGACUUUCCCGU
CCGAGUCCGAAAGCAACCACGCCACCAAGUACGUCUACUCCGUCCCG
>EMBL_CDS:BAD89015 BAD89015.1 Homo sapiens (human) extracellular matrix protein QBRICK
CUUACGUUCUCACACUAUGCUCCUCGACAAAACAGGUUAACCAGAUGGACUUACCACUUAUUUUG
UCCAAGAAGGGUGUGAGAGGGUAAG
>EMBL_CDS:BAD86792 BAD86792.1 Homo sapiens (human) diacylglycerol kinase kappa
CGGAGUCCCGUCUUGAGACCCCGGUCGAGACAGUGUCCAAGACCCCAGCCGAGACCCCGGUCGAG
ACCCCGUUGAAGACCUCAGUCGAGACCCCGGCCGAGACCCUGGCUGAGAUCCCGUCCAAGACCUC
AGCCGAGACCCCG
>EMBL_CDS:BAD66828 BAD66828.1 Homo sapiens (human) KIAA0596 splice variant 1
ACCUUGAGUGACCAUAGGUACCACCCCAACAGUGAGCCGAGGCCCAUCUGUUAUGGUAGAAGUUA
CCCACGUCCAAGGU
>EMBL_CDS:BAD52439 BAD52439.1 Homo sapiens (human) non-muscle myosin heavy
CCGGUCGCCUAACAACUACUUCAAAAAUAGGUGUAUCUCUAUGAAUAGCCGUCGAACGACACGG
>EMBL_CDS:BAD37141 BAD37141.1 Homo sapiens (human) serine/threonine kinase
CCAGGAGGAGUCCGGCGAAUAAAAGACGCAACAAUGAGACUCAGAAGAAUCUCAUUCUUAAUCUC
CACCCUUACCGUCCUGG
>EMBL_CDS:BAD52436 BAD52436.1 Homo sapiens (human) filamin A
AACCCCACCGACGGUCGAGGGUGUAAGUGGGACUUCCCCGACAACCCCUACGACUGCGGAGGGGU
C
>EMBL_CDS:BAH02288 BAH02288.1 Homo sapiens (human) liver X receptor beta
CUAGAGGACCUGACUCUACUACCGGUCGAGGCACUUCACCCGUUUCGCAACGACCGCCCGUAGAG
CCCUGACCCCCAG
>EMBL_CDS:BAG15901 BAG15901.1 Homo sapiens (human) UAF1 RNA polymerase II, UAUA box
CGUUCUACUACGGAAGAGGUAAAAGUCUGUGUGGUCAUUAUCUUAGGACCAGGAAUAGGAAGAAG
UAACCAGGGCCCCCACCUCCACCUCCUCCGUAGUUGAACG
>EMBL_CDS:BAF94759 BAF94759.1 Homo sapiens (human) U cell receptor alpha variable 35
GUCAAGGACGGACCAACACCCGUCGUGUCUUCAUCUACGGAUGUAGUGAUCCAUACCUACGAC
>EMBL_CDS:BAF94561 BAF94561.1 Homo sapiens (human) U cell receptor beta variable 27

GACCCAGAGGGCCGAGGGCGAUCUUGACGACCGUGUCUUCAUGUCUCUCCAGACCAACCCCGACC
CGCUGAGGUC
>EMBL_CDS:BAF63624 BAF63624.1 Homo sapiens (human) MHC class I antigen
UCUUUGGGUCCCGGGUCGUGGAGUCCCACCGGAGUACCAGUCUCUACUCCACCACCCAGUGCACA
CAGA
>EMBL_CDS:BAF94849 BAF94849.1 Homo sapiens (human) U cell receptor beta variable 3
AGACCUCUAAAUCCACUCUUCGCUAAUCCUCGACAAAGUAAAUAUUACUCGAGGAAUAAUAACAU
CGAUUUGUAAUAGAAGUCU
>EMBL_CDS:BAG71128 BAG71128.1 Homo sapiens (human) riboflavin transporter 1
CGGUAGUCCGGGUCGUCCUUCCGUGGUACCCGACUCUUGUCGACUACCUGGAGUCCCAGACCCGG
UCCCUACCA
>EMBL_CDS:BAG12377 BAG12377.1 Homo sapiens (human) N-acylsphingosine amidohydrolase 1
GUAUCGGUCGUUUCGAACUUCGGAACUUCUGUCAAAACAACAAAGAAACCUUUAGGUUUAAGUGA
CAAUUUCCAAAAUCAACGAGUCAAUAC
>EMBL_CDS:BAG48316 BAG48316.1 Homo sapiens (human) acetyl-Coenzyme A carboxylase alpha
GGCGUAAUGGUACGAGGCGUGCCUAAGGAGUUAGAAACUACCCAGGUACUAUUGGCACCCGUGUU
UCAACUCCUUCUACACCAAUGUCAGUCACGCC
>EMBL_CDS:BAG48312 BAG48312.1 Homo sapiens (human) collagen type V alpha 1
GGACCUCCAGGACCGUUUGGGACGUCGGGGUGACCGGGUCCCCCAGGUCCUUUCGGAGACCC
>EMBL_CDS:BAF63512 BAF63512.1 Homo sapiens (human) small G protein signaling modulator 2
GGGCCUCAGGGCCCAGGACGAAGGACGAGUCCAAAACCAGAACUUAGAAGUCCCAGGACCCCGAG
GCCC
>EMBL_CDS:BAF80583 BAF80583.1 Homo sapiens (human) MYO5A variant protein
CAAAACACAUUAAUUUCAAUAGACGUAAAGGAUUAGCAUCGUAAAAUUUAAGUCCAUUGAAACGA
CUCCGUGUUUCG
>EMBL_CDS:BAF63529 BAF63529.1 Homo sapiens (human) Sickle tail
GUCCGCCACCCCUACCCCAGGUCCGACUUCGUCCGAGGAGACGACCUAUAAAACAUCGAGACCAG
ACACCCGAACCAGGUCGUAGAAGUGACGAAC
>EMBL_CDS:BAG06714 BAG06714.1 Homo sapiens (human) MYO5B variant protein
UCGAUAGACCCCUCGGCACCGACCUCAAUAAGACGCACCGAACCAGCCGGUGCCGAAAGGAGUCG
A
>EMBL_CDS:BAG06722 BAG06722.1 Homo sapiens (human) DNAH10 variant protein
ACCCCUUCGGGAACCACCCCAGGCACCACUCGGUGUCCGCCUUCAGACCCACCCCGAACCACUAG
GAGAGGUCCCUGAAGAGGU
>EMBL_CDS:BAD92948 BAD92948.1 Homo sapiens (human) RAB40B, member RAS

oncogene family
CUCGAAGAGGACCUCUCUAAGGCGGCACAAAGAAAGUCACGGAAGGAAUUCUUUAAAACGUCGAC
AAAGACCACGUCAAAAACCCCCCCCGAGACCCCCCCCGCCUGCUCGAAGUGAAACGCCUCCGACG
AG
>EMBL_CDS:BAD92557 BAD92557.1 Homo sapiens (human) LEPREL2 protein variant
AGCCUCCUACUUCGCGACCUACAGGAGAGACCCAGGCUCCGGUCCAGAGCCGAGAGGGUCGACCC
GGACCAUGACCAAGUCUCGGGAGAACCGUCGGAGUAGGAGGCC
>EMBL_CDS:BAD93084 BAD93084.1 Homo sapiens (human) zinc finger protein 211 isoform 2
CCGACCUCGAAGCCGAUUUCCUAAAAGGUGUAAGUAACGUGAGUACUCCAGAAAGAGGUCACACU
UGAGAGACCACCAUUUAGUCCGACUUCAAAACCGA
>EMBL_CDS:BAD93143 BAD93143.1 Homo sapiens (human) tripartite motif-containing 38
UGCAGAAGGACUCCACUUCGGAAGUGUUGGGUUCUGUGUCCCCUUCCGUCAUUUAGAGGACCUUC
UACA
>EMBL_CDS:BAE06093 BAE06093.1 Homo sapiens (human) UNPO2 variant protein
UUGAACCCCUAGUACUACAACUCCCCGGACAACUCCCGCAGUGACAGGUCUUCGAGACUACUCAG
AAGUGUCUAGAAGACGUCCCGAGGUUUCCGAGGGAGUGUUCACAACAUUAGGAGGCUCAA
>EMBL_CDS:BAD92719 BAD92719.1 Homo sapiens (human) Diaphanous 1 variant
AGUUAGUAGUACAACCCUUGUCGUCCUAGGUACCGAGACUGGUCGUCAUCCUAAGGAAGAAGACA
GAGGUUGUACCAGAACU
>EMBL_CDS:BAD92976 BAD92976.1 Homo sapiens (human) Inositol 1,4,5-trisphosphate receptor
CCAUGAACGUGUCCCCCGACUACAGUAACUCCUAGUCACGCCACUACUACAGAUACGGUAACCUG
AGCACUCAGUGUUACGUUCAGACCAAGAGUACCGUCCCCGGGACCGUCAUGA
>EMBL_CDS:BAE06086 BAE06086.1 Homo sapiens (human) CSPG3 variant protein
CGUGACUUCGAGGACUACAAGGGACCCAGAGGUGGACUAGGAGACCGAGACCCACCCCAACCCUC
GAACUCCUCGGGUCACA
>EMBL_CDS:BAE06083 BAE06083.1 Homo sapiens (human) APC variant protein
CAACGACCCGUCCAGAACUUCCCCAGCUUAGAGAUCUAGGACUAGAUCUUCCACGAAAUGAACUG
GGUGGAUAAACCCUACAGACGGACCGUUG
>EMBL_CDS:BAD92747 BAD92747.1 Homo sapiens (human) EBNA-2 co-activator variant
ACCGUUACGUCUUGAGAGGGGACGCCCCCCGUAUCCUCGGGAGAUGUCCCCCCACUGACCGUUAC
AGU
>EMBL_CDS:BAD93123 BAD93123.1 Homo sapiens (human) MSH5 protein variant
CAGGUCACUACUGAAACUGGGAGGGAAACAGGGGUGGUGUAAGACACCUCAACCCGUGUUUCCAA
GCCCGUGUCUCAAGGUAGUCUCCUA

>EMBL_CDS:BAD92921 BAD92921.1 Homo sapiens (human) Synapsin-3 variant
GAGCCACCACCGCAUCCAAAACCGGUACCGCUGGUGCGACCACUACAGGACCUUCAGUUCGACCA
AAAGGUGAAACUAGAAAGGGUAAGGUCGCACCCGUACAGGGUCGAACUGAUGGUGGCCC
>EMBL_CDS:BAD93102 BAD93102.1 Homo sapiens (human) latent transforming growth factor
AGUCCGUAACCACCCCAACACCGUAAGGGACACUGGGUCCGUCCACGUACCCACUGUGACCAGCA
CUGGACCGGUCGGACU
>EMBL_CDS:BAD93140 BAD93140.1 Homo sapiens (human) LL5 beta protein variant
AGGUACGAUAGUCUCGAUAGCUCCGAGUCUCAUAGUUUCCGGACCAGGAACAUCGACACUAGACC
UAUUACCU
>EMBL_CDS:BAD92774 BAD92774.1 Homo sapiens (human) G protein-coupled receptor kinase
CUACCCCACCUGAAACAGAACCCCCAACCGAAAUGCGGCCGGCGAGACGUGAACACGCCCCAGGU
CGUCCCUCACGAGGGUCUACCUCAACCGGGGCAA
>EMBL_CDS:BAD92514 BAD92514.1 Homo sapiens (human) son of sevenless homolog 2 variant
UCCUCUAAAAUUCCUUUUUCAACUAAAGGAUCCUUUAGCUCCACCGACAAACGUCAAAGCUCCAA
GUUAAAGAUCACUGAACAACUUGUUUAUUAGACAUUUGAGAAAAAGUCUACGUGAAGGGUACCCC
AAUUCCAAAAGUUUCUUAGAGGA
>EMBL_CDS:BAD92702 BAD92702.1 Homo sapiens (human) hypothetical protein FLJ16665
CUAGGUACCGCUAGACUCGUUCCCUGUUCCUACCUUUACCCAGAGAAUAAUCGUAAACGAACUAG
UGGUACCCAA
>EMBL_CDS:BAD92714 BAD92714.1 Homo sapiens (human) anaplastic lymphoma kinase Ki-1
CGGGUUCGACCUCCUGACAGGGUAAGGUUGUUCACUUCCUCGAGACGUCCCGGUAGAACCUCGGA
CCCCUACAAGGAAGUGACGUCAAGAAGUCCCG
>EMBL_CDS:BAD92530 BAD92530.1 Homo sapiens (human) SWI/SNF-related matrix-associated
CGAGGAGAAGAGACACCGGUGCGAAACUGAAUCACAUUCCACAACUCCCUGGAAAGAAUCGUAAA
AAGAAGAAACGAAGACCCCAACCACUUCCUCUCCCCCUCG
>EMBL_CDS:BAD92651 BAD92651.1 Homo sapiens (human) A-kinase anchor protein 13 isoform 2
ACAACCUCGCCUUCUCUUGAGAUCCCGAUACACAACCCCUCGUUAGGAGACCCUCUCCCCACGAC
GUGAGUCGGUACAGGGCCUUUUAGUAGAGGAAGAGAAGGUUGU
>EMBL_CDS:BAD92907 BAD92907.1 Homo sapiens (human) protein tyrosine phosphatase,
CCAAGAAAGGUCAAGUUCUUCUAGAAUAAAAGUUUCCUACAAGGAACUUCUCUAAAAUGACCAUG
CCCUGG
>EMBL_CDS:BAD92640 BAD92640.1 Homo sapiens (human) iron-responsive element binding
GUCUUAGUGUUCUAGGAGCCGUCCAUCAGACCGGAGACGUCCCUUCGAAGAAUCCGACGUGAAAU
UCACCUCUCUCGAAAGGACGAAAGACGUCCAGUGGUGGAGGUCCUAAACCACGUAAGAC

>EMBL_CDS:BAE06113 BAE06113.1 Homo sapiens (human) C14orf159 variant protein
GGUCUCCCGUCGUCUUCGGUCGUACCCAUUGUGUUCCGUGACAACAGAACAUACGUGGGCGGACC
GACACUGGACGACCCAGAGAAGACC
>EMBL_CDS:BAD92895 BAD92895.1 Homo sapiens (human) integrin alpha 3 isoform b, precursor
AUCGGCUUCCACCGGUUGGGUCCGUCAGGGUCGAAGAGAGGUACCUAAUGGACGACCCCGACAGA
CUCCUCGGGGAAUCUCGAU
>EMBL_CDS:BAD92894 BAD92894.1 Homo sapiens (human) platelet-derived growth factor
CGUCCGUCUGGUCUACUACAAGCCGACCCCGUACGGGGCCGGUGCUGUCGCCUGACAGACA
>EMBL_CDS:BAE06120 BAE06120.1 Homo sapiens (human) UNC variant protein
GACCAAGAGUAGGAGACACUCUAGCUACCACCAUGCCAGAGGACCGUGCAGAAACUACGGCAUCC
AGUCGAGUUACGGUAGCUAGAGUCGGUCCCCGAACUUGGUC
>EMBL_CDS:BAE06087 BAE06087.1 Homo sapiens (human) MEF2D variant protein
UGGUCCACCCUGACAACGACUCCACCGACAACGCCGACGACUCCGACGACACCGACACCGACGAC
ACCGACGCCACCGACGACGACACCUCCGACACCGACGACGCCGACGACCCCGACGACACCGACAA
CGGUCCGUCACUGUAACGGAUCGCUGUCGGGGGGUCCA
>EMBL_CDS:BAE06094 BAE06094.1 Homo sapiens (human) DAB1 variant protein
CCAGCCCAAACGCCCUCCCGUGCCCGCCUCCGACCCGGUAGACCUUUAGGAAUUUGCAAAGAAAC
GGGUAAAAGACGGACCCGAACAGCCAGACACCUGAACUGG
>EMBL_CDS:BAD92901 BAD92901.1 Homo sapiens (human) Hypothetical protein DKFZp666D035
CACAUCCCAAAAAGAAGUCUUACUCAAGAGAAUACCAAUCUUUCCGAACUCGUUCAAGACUUCUG
GAAGGGUGUA
>EMBL_CDS:BAD92880 BAD92880.1 Homo sapiens (human) smoothelin isoform b variant
CCCGUCGUCCCCAGAGAAACCCCGGUCUGUCUUCGAACAACUGGUGGACCGGGAGUCACGACCCC
UCGCAACCUCUGAGGACUCCGAGUCCCGACCACCAGAACCCACCCGACCCCCCACCGAGACCCGA
CGGA
>EMBL_CDS:BAD92987 BAD92987.1 Homo sapiens (human) RE1-silencing transcription factor
CAAAAGAAAUCAAAGUGAAAACUCUGUAGGUAACAAAAUAAUCCUGUUCAUCCUACGAAUCUAAA
CUUCACUAUGACAUCUAAUG
>EMBL_CDS:BAD92820 BAD92820.1 Homo sapiens (human) phospholipase C, beta 2 variant
CGUGGACCAGUCCGGCACGACCGCCCUUGUCGUCCCUCAAUUCGGCCCUCAGGGCGACAAAGACC
AACUACUUAAACCAGUCCACG
>EMBL_CDS:BAE06070 BAE06070.1 Homo sapiens (human) FASN variant protein
CCCGUCCCCGACCAGGUGGUGGACGUCGGCGGGUAGGUUGUCGCGACCCUAGAGUCCCAACCCCG

AUACCUUCACGUCCAACCCCCGGGUCUCGGGCACGAG
>EMBL_CDS:BAD92549 BAD92549.1 Homo sapiens (human) DNA-repair protein complementing XP-G
AGGACACCACUCUCUCCAGGGGACGACUUACGAAAUAAUCGAAAUUGUCCCCAACGAAGCUUCGG
UUAACCU
>EMBL_CDS:BAD93125 BAD93125.1 Homo sapiens (human) natural killer-tumor recognition
AAAUCUUGCAAAUUGAAAACCGGAGGAGAAUACAAAGGAGGAAGACGAAGAGAGUACAAGUUCAA
GUGAAAGACUUCUACUAAGACUUCUUCUCCUCCUUAACCUUCCCCUUAGGCUCGGAAGACUU
>EMBL_CDS:BAD92735 BAD92735.1 Homo sapiens (human) low density lipoprotein-related
CGUAGACCCCCCCCGCAACCUCUCCCGUGUCCGUGCGUACACGGCAACAGGUCGGCGAAGGGUAA
CCCUGUCCACG
>EMBL_CDS:BAD92858 BAD92858.1 Homo sapiens (human) zinc finger, MYND domain-containing
GGACGGUGAACGGAGACAAAAGAGCGAGGUCGGCGAGGGUCUAAAGACCCUAGAUCCUCUCUCUU
CACCUCUCACCGUCC
>EMBL_CDS:BAD92580 BAD92580.1 Homo sapiens (human) elongation factor, RNA polymerase
AUCGUUCAUCGAGGCCAAAGAACAUCCGGAAGUCCCGGUCAUUCACUUAGUGGGACAGGGACAUA
CCGGAGACUCUCUACCACGACGACGAU
>EMBL_CDS:BAE96739 BAE96739.1 Homo sapiens (human) downstream of tyrosine kinase 7
GGUCCGACGACGGGCGCAUCCUGCUCUCCGACGACCUCAUUCUCCUCACCGACGGUCACCGCUAC
GGCGACAGGCUCCUCGAGACCGCCGGUUGGAGGACGUCGACGGCGCCUGCGUCGCCGAACC
>EMBL_CDS:BAE44474 BAE44474.1 Homo sapiens (human) hypothetical protein
CGUAACCCCCGGUCCUGUUUCCCCGAGACGCCCCGCACCCGGAGACCAUCCCGUCCCAGACCUCG
AGACGUCUUCAGGACCACGACCUACA
>EMBL_CDS:BAE17137 BAE17137.1 Homo sapiens (human) hypothetical protein
CUUCCUACACCACGAUGUCCCGAACCACUGAAAGCCAACCCCUCGUGGUUCAAGACACGAAAGAA
A
>EMBL_CDS:BAE96351 BAE96351.1 Homo sapiens (human) mammalian diaphanous homologue
GUGUUUUAAGUCAUAUAGUCCCAUGAAGAGAAGUGUAUAAAGAUGGUCCUUUACUUCAUCGCAAC
AAAAGACUAGACGACUAAAACAC
>EMBL_CDS:BAG70269 BAG70269.1 Homo sapiens (human) MLCK protein
GGUGUAACUGGGACGGCCCACGACCUCGGUUAAGGUCCGGUCAGGACUCCAAGAGCGACCCACAG
AGUCCUCGGAACCGAAGGUGGAAACACCCGUCCCCGUCCACC
>EMBL_CDS:BAD92391 BAD92391.1 Homo sapiens (human) Uyrosine-protein kinase transmembrane
GGAGGUACUUGGAGUGGCGUCUGUCCGACUAGAGAAACUCAAACCGGACAAACACGACCAAUUAC
UCC

>EMBL_CDS:BAD92390 BAD92390.1 Homo sapiens (human) formin 2 variant
AAGACAUCAUUAAGGUCAAACGCUACUUAACUCGUUUACGAAAGACUUUUACACCUGACCUUGUC
CU
>EMBL_CDS:BAD92083 BAD92083.1 Homo sapiens (human) Apolipoprotein B variant
UUAGUAUGAAUUUAAGGUACCUCCUUCUCUGUCAUCCAAAACUGAACCAUAAAGGUAAUUCAAGA
ACGAACUUUAAGUAAGAGUUUUAACCUUAAACCCGAAAACACCAA
>EMBL_CDS:BAD92195 BAD92195.1 Homo sapiens (human) protocadherin gamma subfamily A, 7
AUAACUAAAAAGAUUAAAUUUUCCAUCCAAAAGACCUUACCACGUCCAGUGGAGUGGUAAGAAAG
GUCUUAGAGACAGAACAUCCAUCUUUUCUCGUUAC
>EMBL_CDS:BAE53437 BAE53437.1 Homo sapiens (human) C8orfK32 protein
UCCCCAAGUCGAGACCUCCCCAAGAAUAACGAUCUAAAAUGCAGUUACAUCCAGUCGUGACCCCG
GACAGGUAGGAGACCUGGACCUGAGGA
>EMBL_CDS:BAD92137 BAD92137.1 Homo sapiens (human) hepatocyte growth factor isoform 1
AACAUAAAGAAGAAAAGGAAACAGGGAGACGUAUCCCCUACCGCUACCCCUCGUCCUCCUCUACG
UC
>EMBL_CDS:BAD92305 BAD92305.1 Homo sapiens (human) ash1 (absent, small, or
CAACAGUGAAAGUCUAAGAUCGAACGAACCUGAAAGUCACCGUUGUUUACCUCGGUGACUGUCAU
GUCCACCGAAGUAGAACUGACCACUGGAG
>EMBL_CDS:BAD92062 BAD92062.1 Homo sapiens (human) poly(rC)-binding protein 2 isoform
UCCACCAGGGAGGUCCAGUCCCAACUCCGUGUACCUGCCCCACCACACCCCUUUCGAGCGUGACA
GCGACGGACACGACAUGGACAGGACUGGUGGA
>EMBL_CDS:BAD92359 BAD92359.1 Homo sapiens (human) glypican 1 precursor variant
CCAAGAGGAGGUAGAGCGACCACGUCGUCCACAUCGGGACCCCUGUCUAGGCGUCCACGAGUGGA
CACCCCUUUCCGGUCACACUCCCUCUCCCCGA
>EMBL_CDS:BAD92458 BAD92458.1 Homo sapiens (human) hypothetical protein FLJ41407 variant
CCGAGGAGGUCCACGAAGACAUCUGUGUCCACUCUCCAUUGUAGGGUCCGGGACCCUACCACCAG
AAGGGACCCGUCACUGGACACUCCCGG
>EMBL_CDS:BAD92438 BAD92438.1 Homo sapiens (human) WD repeat-containing protein 3
CUCUAUGACGGUAUUCGGUAAUCCUACCCCAACGACCCUUCACCUUGGAGAAAGGGACGUCGAAA
UGUUUACCGAAACACAAGGAAGUAAAAACGUCAAAGAA
>EMBL_CDS:BAE16982 BAE16982.1 Homo sapiens (human) connexin43-interacting protein of 150
CAUCAUAAACCUGGUAGUCUAAAAGAAAGUUAUGACAUUUCCGUAUAACUUGUCAGACGAGAUUU
AUGAUA
>EMBL_CDS:BAD92206 BAD92206.1 Homo sapiens (human) KIAA0182 protein variant

AGGAGGAGAAGGAGGAGGAGGCUCAGGGCCAGGUUGAGGACCCAACCCCCCGGGAAGGAACGUCA
CCCCCCGAGGACGACGGGUAACACGACCCCACCUCCCCGCGCCGACCUCCU
>EMBL_CDS:BAD92022 BAD92022.1 Homo sapiens (human) phosphoribosylglycinamide
AAGGUCGACGUUCUGGUGACCGGAAGUGUUGGUUUCGUCCCUUCAGACGUGAGUUUUACUUCGAC
GUCCGAAGAAGUCCAAACCACUUUCGGAAGGUAACACGCCAACCCU
>EMBL_CDS:BAD92171 BAD92171.1 Homo sapiens (human) plexin C1 variant
CGACACUCUGAAAGUAAGAACAGCAACCGCAGAGACAAUACGUAUAGAAGUCACUUCUACACCCA
CUUCGGUGGACUGAGUCCCUUCUUUCAAGAGUCUCG
>EMBL_CDS:BAE46995 BAE46995.1 Homo sapiens (human) PPAR gamma DBD-interacting protein1
CCGCACCUACCCCAACGGGAACCGGUGCAUCUUCACGGCGCUCUACUUCGACUGUUACCGCAGGC
ACGUCGCCAUCAAGAGCACCUUCUGGUCCGCCGAGACGGCGCGG
>EMBL_CDS:BAF43710 BAF43710.1 Homo sapiens (human) hypothetical protein
CCUCCGACAGAGGACGUCUAGUACCUCCUUAGACGACAAGUGAAACUACUAUAGUAGUUACAAAC
UCUUGUCCUGUAGGUCUAGAGGGACGCCGUUGACGACCUCCGCCGAGG
>EMBL_CDS:BAD92025 BAD92025.1 Homo sapiens (human) Retinoblastoma-associated protein
AAGUGUUUCACAUAAAUCGGCCUCUAUCCGAUCGGCUAUGUGAAAAAAUAUUUUGUCACUU
>EMBL_CDS:BAD92410 BAD92410.1 Homo sapiens (human) transducin beta-like 3 variant
AGGACACUCGGACACCGGUCGUCGAACAGCAACCCCCGUUAUCGGUGCGACAACUACAGGAAUAG
UACCGUCGCGACUCACCAGACCCGGACGUCCU
>EMBL_CDS:BAD92113 BAD92113.1 Homo sapiens (human) axin 1 isoform a variant
CGACUCCCGGCCGGGUCCCCACGAGUUCCCUGUUCCCAGACCUCAAGAGUACCCCGACACCGAAG
GAGCAGGGGCUUCUGGGCACCGGCCAGGACGCCA
>EMBL_CDS:BAD92250 BAD92250.1 Homo sapiens (human) sorting nexin 1 isoform a variant
UCCUCCUUCUUCAGUAACAACCGGACAAGGACCACCUCGACGAGUUAAAAAAGAAGGUGGA
>EMBL_CDS:BAD92184 BAD92184.1 Homo sapiens (human) chordin isoform a variant
CACGUGGAGAGGUCACGGGGGGAACGUCCACGUCUGUCGUGUGAAUUAAUUCGGUUUCCCCCCGU
GUUGCCCCACGG
>EMBL_CDS:BAD92413 BAD92413.1 Homo sapiens (human) calcium channel, voltage-dependent,
GUAGAGGGUCUGACCUUGACCUCAAUAGCGGACUAAACGACCUCGGCUCCGCCAAGACCGCGUCG
AAGACAAGGACGUCGUCCGCUUCCGACAGGUCAAACUGCCCCGACCCUUAC
>EMBL_CDS:BAD92392 BAD92392.1 Homo sapiens (human) serine/threonine kinase 23 variant
GGUGUGUGUCGUUCUACAAGAGCCCGAACUACAGGCACACCUACUAGAACGUGAACCACACC
>EMBL_CDS:BAD92189 BAD92189.1 Homo sapiens (human) colony stimulating factor 1 isoform a

GACCCCCCACACUAAGGUCAGGACCGGUCACCCGGAGUGCCCCGGGUGGGACCCGUUCCGACAUG
GUCAAUGUAGACGGCC
>EMBL_CDS:BAD92109 BAD92109.1 Homo sapiens (human) SUMO-1 activating enzyme subunit 2
UGAAAAAGGCCCCGUAGUGGUUGUUGAAGUUUAAGUUGCAGGAAAGGAUCCAGAAGUGAUACUUC
CUACAACUAGUUAUUUCAUAUCAGGACCUCCUUCA
>EMBL_CDS:BAD92020 BAD92020.1 Homo sapiens (human) Oviduct-specific glycoprotein
CCCAGUGUCUGACUACUGGGUGUCCCCAGUCCCAGAAAAGGGGUCCCCAGUGUCUGACUAUUGGG
UGUCUCCAGUCCCAGAAGACAGGUCACCAGUGUCUAACUACUGGGUGUCCCCAGUCCCGGAAGAG
AGGUCCCCAGUACCUGACUACUGGG
>EMBL_CDS:BAD92212 BAD92212.1 Homo sapiens (human) nuclear transcription factor Y,
GACUAUGUCCGACCCGAUUCGCCUAUAUGACGUCGACCGGCCGUAAGUCGACGUGGCCCUAGACG
ACCUAGAGAGGACACAAUC
>EMBL_CDS:BAD92489 BAD92489.1 Homo sapiens (human) chapsyn-110 variant
GUUGUUCGUACACGAAACCUUAACCACUCAUGGAAGGACCUCUCUACCCACCGUCCCUCCAAAAU
AUAAGAUUUCACGGUAACAAC
>EMBL_CDS:BAD92053 BAD92053.1 Homo sapiens (human) calpastatin isoform a variant
CGACAUCCGCUGGGUGUCCACUUCAGUCUACUGUUCCGCAGAUAUCGUAGUAGACCGGGAUACCC
AAAUCGGUUAUCA
>EMBL_CDS:BAD92377 BAD92377.1 Homo sapiens (human) son of sevenless homolog 1 variant
GUCUCACUCCAACCACCAUACAGCUUAGUAAAAGUCUUCUAAUCCUCUCAAUUAAAGAAACAGAA
GAAAACAGGUGUGUGAAGGUUGAUUAAGAC
>EMBL_CDS:BAD92110 BAD92110.1 Homo sapiens (human) PUPL1-associated RhoGAP 1 variant
CUCAGUCACAAGAAAUUGUCGUGCAACAGAUCAAGAUUUCCCCUUUUUAAAGGAAAAAGUGACCU
ACUCAGGUGACUAAG
>EMBL_CDS:BAD92177 BAD92177.1 Homo sapiens (human) hypothetical protein DKFZp434B216
GUCUUCUAUUUAUGACUCCACCUUUUCAACUUCGAGGUUUCCGGUACAGAAGUCAUAUAUAAAAG
AC
>EMBL_CDS:BAD92356 BAD92356.1 Homo sapiens (human) eukaryotic translation initiation
AUCCGAAGGUCCUAAGACCAAAUUUACCUUUACAUUGGGGUAGUGGUCUUGAUAGACACCUCGGA
C
>EMBL_CDS:BAD92300 BAD92300.1 Homo sapiens (human) Polyhomeotic-like protein 1 variant
GAUAGACCCCGGCUCUCGACGGGGGACUCGAUCUUGAGGGUAACCACCUUAACCUCAAGACGAGC
GAGAGUCAGAGGUACUAUC
>EMBL_CDS:BAD92412 BAD92412.1 Homo sapiens (human) collagen, type V,

alpha 3
GUUACACCGGGAAAAGGGGGAACCCCGGUCACAGAGGGAAGUCGGGACCCUCCGGUCUCCCGGGG
UCCCCCGGUCCUCCCGGGUAGAC
>EMBL_CDS:BAD92396 BAD92396.1 Homo sapiens (human) Uhyroglobulin precursor variant
UGGUACAUCGUCGUCGGAGCUGCGCCCGUAGACUCAGGAGCGGUGGGUCUCUUAAGACGUCACGG
ACCAUUUUCCGACAAAACGAUGACCA
>EMBL_CDS:BAD92334 BAD92334.1 Homo sapiens (human) KIAA0367 protein variant
AAUCAACAAACGUAAAGGAUAAAGACUCAAAGGGUGAACCACACACAAAUAAUUCACUCGAUCGU
CUUCACUGAACUACAUUCGUUCACU
>EMBL_CDS:BAD92066 BAD92066.1 Homo sapiens (human) G2/mitotic-specific cyclin F variant
CAGGUCCCCAACGUCCCUAGACCCACAGGUCGCGGUCACGGACACCCAGGACCCCAGGGAACAGG
AGACCUG
>EMBL_CDS:BAD92357 BAD92357.1 Homo sapiens (human) AUP-binding cassette, sub-family C,
CCUGGACAUUAUACCAAGGAGGUCGACUGUAGGUUUUUCCUACAAAAGAGCCUCUCUUAGUAAGA
CUUAGG
>EMBL_CDS:BAD92101 BAD92101.1 Homo sapiens (human) flightless I homolog variant
GCGACCCACGAGGCGUCCACGUCCCAGACGUCCCGGCAGUAGCGACCCUCGACGGCCUCGACACG
UACGUCGUCCCCCAAAGGUAACUCGUGCUCGCAGACGUCCACGUGGUCCGCCGCGUAGACGC
>EMBL_CDS:AAC04305 AAC04305.1 Homo sapiens (human) R29144_1
UGUCGCUGUAGUGGUGGUGGUCGUCGUGCAUCAACAGGUCCUACCCCAGACCGAGACGGCACCCC
GCAGGUCACCGUAACAGGGUGCUGCAGCAGUCCCGAUGACACCGUCACCACCACCACCACCACCA
CCGCCGACA
>EMBL_CDS:AAC24313 AAC24313.1 Homo sapiens (human) Unknown gene product
CCCUACAACACUCCCUCGUACUCCUACGACUCCCGGGCCCCGGUCACGAGUUACAUACCCUAGUC
GUAAGAGUCAGACCAAAGGUGACGUAGGG
>EMBL_CDS:AAS07423 AAS07423.1 Homo sapiens (human) unknown
UCCCUUGAGGUGGUCGAUGUUCUUCGAAAACGACAGCGUAAACAAGAAAGUUAUUAUUUACGACG
UCCUCUCGGACCACCGACGACAACCCACCGAAAGGGA
>EMBL_CDS:AAC07983 AAC07983.1 Homo sapiens (human) P1.11659_4
CUCCCGAGGUAUAUGUGGGUACCGGACUCGGUGGUACGACCACUGUAGCGGUCCCAACCUCCCGU
CAUCCUAUCACAACCUCAGGAA
>EMBL_CDS:AAS07390 AAS07390.1 Homo sapiens (human) unknown
GGGACUAUAAGUCCUACUACCACUCGUCCACGACCCCGUCCUCCACCACCAAGAGAUCGUGGACU
UAAACUCAGUAGGACUUAUCCC
>EMBL_CDS:AAC08453 AAC08453.1 Homo sapiens (human) KIAA0324
AGGUCUAAAGUUGUAUCCUGAGCGACUCUUGAGUGGAGAUAGAACAGAAUAACCUCUAGACCU
>EMBL_CDS:AAB94785 AAB94785.1 Homo sapiens (human) pNORF1
ACGUAGGACAGUUUCCUCCAGCUGAAGGUGUGUUUUAGGUGGACCUUCAACACUCAGUGGAGGUG

UCCACGU
>EMBL_CDS:AAC32592 AAC32592.1 Homo sapiens (human) APXL
UCCAGAGGACCCAACCCCAGGUUCAGCGCCGCGAACUUCCUGCACCGGGAGUCCUGGGCCCGAAC
CCGGAGAAAGUCCACCAGA
>EMBL_CDS:AAC15789 AAC15789.1 Homo sapiens (human) Notch 3
GUCCUGGGCCCGAUCCUGCCGUGACCGGAGGUACGACCGUACCGACCCUGAGCGUCCCUCUGUCC
UGUCAGACUGUCGCUCCUGGACUCUUCGGUCGUACUGUUUCCGUUGCCUUUGGAGGCGGACCCAG
GAC
>EMBL_CDS:AAC08455 AAC08455.1 Homo sapiens (human) R30385_2
ACACUUUCCUCACCACUUUUGCUUUCCGUAAGGGUGUACCGAAUGUACAUAUACGGAAGACAGAU
GUAAGGACUAUAAGUGU
>EMBL_CDS:AAC08447 AAC08447.1 Homo sapiens (human) CBP
CCCAAACACCGGGGGUCCCUUCAGCCUUCUCGACUAAGACUUUAGUUCAAACCCCCGACGACUAG
A
>EMBL_CDS:AAC27426 AAC27426.1 Homo sapiens (human) Uranslation initiation factor
CGAGCCCCAAGACGAACAUCGAACACUAUACCCUGAGCUUUAGUGCCAACAUAAAAGCCUAGAAG
ACUGCGUUCCACGAGUCUCGGAACCG
>EMBL_CDS:AAD03158 AAD03158.1 Homo sapiens (human) immunoglobulin-like transcript 10
CGGGUCUCCGACCCAAACCCCUCCCAGGGACGUUCACGAACCCGAAAGACCGGGUCUGAGUCGGA
CUCCG
>EMBL_CDS:AAC97987 AAC97987.1 Homo sapiens (human) SN24_HUMAN
CCGCCUCCCCGUCACACCCGCUUCGACCGUGACGGCGACGGGCACGACGUCACAGACGAGAGUAG
GACGAGGAGCACGA
>EMBL_CDS:AAS02034 AAS02034.1 Homo sapiens (human) unknown
AGACUGCCGUUCCGUUUACAUGUUCUGACGACCUUCCCGGUCCUGGGCCUCGAGUCGUGAACUUA
CGACUUUAGGUUCAGAUCCCCAAGACUCAAACAGUUCACGAAAAACUUAACGAGCAAGUCU
>EMBL_CDS:AAS46897 AAS46897.1 Homo sapiens (human) unknown
GACCCGACGCACACAAGGCGACGUGGUCCGACGACCCGACGUAGGACAAGUCUACCUGGAGUCAC
UGGUCCUCCUUCUGUAGACAGUCCCAGUAGCACGUCAGCCCGUCGACACCACUCGGAAGUUUCCG
UCGUGCC
>EMBL_CDS:AAC33490 AAC33490.1 Homo sapiens (human) F20887_1, partial CDS
CCAAAGACCUCGGUGACUCGGUAAAUCGAGGUACACCCAACCGGGCAAAAACCACCGGCACCGAG
GUCGUUGG
>EMBL_CDS:AAQ96886 AAQ96886.1 Homo sapiens (human) unknown
CGGUAGACCCGGUAGUCUGGACCGACGGCCCCGCGUCGUCGUCUUCUCGGUUGACGUCUACCCCC
ACGGCGACGACCACCGACUCGCUGACCACGACCGGACCCACGUCGAGAGUAGACUCCCCGGGCCG
ACCG
>EMBL_CDS:AAC62835 AAC62835.1 Homo sapiens (human) R33374_1
CCGAGCUCACUAAGGUCUGGUCGUCGAACAACAGCAACGGGGGGCUCCGCUCCUCGACCACCAGA
CACCUGGUGAACUCGG
>EMBL_CDS:AAC24489 AAC24489.1 Homo sapiens (human) H53_GS1

GUCCCUGAACGUCAGUUCCGACCGGUAAGACCACAAGGACAGGUUGAAGGGUUUCCCGGACUACG
GAGAAACCCCAUGUCAGUGCUGUCCCGACUACGAGAGUCCUGGUCGGACAGGAACGUUCGGGAC
>EMBL_CDS:AAX82033 AAX82033.1 Homo sapiens (human) unknown
CCAGGCGGAACCUAGGGUACUCCUACUCCUCCGGGAACGUAAGGAGACCCUGGUCCACCAGG
>EMBL_CDS:AAC33801 AAC33801.1 Homo sapiens (human) R26984_1
CCUGUCGAGACCGUCUCCGAUCGGCGACGAGGAGUAAGAGACACGAGCCCUACUUGUCCCGGCCC
CCCUCCUCCUGCUCGACCUCGGUGACGACCCCGGCCAGG
>EMBL_CDS:AAC25582 AAC25582.1 Homo sapiens (human) ubiquitin-52 amino acid fusion
CCGUUCGACCGCCUCUCUUCCGAGUUAUUACGGUGGAGCGUCCGCGUUGUGGUCCACGUCCCACC
UGAGAAAGACCUACAACAUCAGACUCUCUCACGCCGGUAGGAGGUCGACAAACGG
>EMBL_CDS:AAC23434 AAC23434.1 Homo sapiens (human) hypothetical protein
GACGGGAGAGGGGAAGUAGACAAAGGCGACGAGGAAGACCCGGACGAACCCACCGAACCCGAACC
GAAACCUCGGUCCCUCCCCCCGUAGAUCCCCGUCCGCCUACCUACCACUUCCAACAUCCCGUC
>EMBL_CDS:AAD30180 AAD30180.1 Homo sapiens (human) hypothetical protein
GGUCCGUCCCCGACCGUAGUCCUCCGGAACGCCACACCCUUCCAAGACGAAGGAGAAGGAGAGAG
CCCGGAAGAAACCCGUCGCACCCGACUGGGGUCGAACGCGUAACGGACCGUGGUCUCCUAAGCCC
CUCUCUGUGUACGGAGCCUGUUACCAGAGGUGAGACCGGGAGAAACGGACC
>EMBL_CDS:AAC62840 AAC62840.1 Homo sapiens (human) R33083_1
CCCCGCGGAACAACUGUUACUCCGGGCUCAUGAACGCCGACGGCACCUACUACGAGGCCUCGGGC
AGGGUGCUCACGAAGACGUGGACCUUCGCCCGCCGGCCCAGUAGACACCGCCGACGCAGCGGAGC
CAGCCGGCAACCCCAGGG
>EMBL_CDS:AAS00377 AAS00377.1 Homo sapiens (human) unknown
AAGUCCUUUUCCUAACCCCUACCGGCCGUCUCUACUCCAAGAUUUGACGACCAGAAGGAGGACUU
>EMBL_CDS:AAC71655 AAC71655.1 Homo sapiens (human) hepatocyte growth factor
AGUCCGUUCUUAAACACGGCCACACCACAGACUACUAGGGUCGCGACUGUUUAGAACGGACU
>EMBL_CDS:AAD11988 AAD11988.1 Homo sapiens (human) BC85722_1
CGGCGACCCUCGGCGACGGAACCAGGGUCCCCGACCACGACCCCGUCCACGGUGGACUUCGACGA
CCCCGUCGCACCCGUCCUACAUGACAUGGACCCACUAAGGGGGUCGGAACGGGUCCCGUGGACCA
ACGUCCUACGGGUCCCCA
>EMBL_CDS:AAC62261 AAC62261.1 Homo sapiens (human) neuronal apoptosis inhibitory
AAACCUCUUUACCUGUUAACCCUUUUGUAGACCACACUAGCAGAUUACCCAGUAGUGGAAGGACG
GUAAAGAGAUUU
>EMBL_CDS:AAC24305 AAC24305.1 Homo sapiens (human) R31449_3
ACCCGUUUUGAGCCCCAGUGACCAGAGUACGAGGUUCCGGGGCACUCGUCCGGUCCACCCCCCUA
GGGGCGACGCCCCUUUACCUGGGGUGGUCGGUCGUGCCCCACUCAACCCCCGGUCAGGGACGUCU
CACACCCACAGGU
>EMBL_CDS:AAC18916 AAC18916.1 Homo sapiens (human) Acetolactate synthase
UUGGACGACGCCCCUUCGGACCCCCUACAGGUCCCCGUCGCCAGGGAGCCCGACUCCGAGGGUCC

GAGGACGUUUCUCCAA
>EMBL_CDS:AAX88848 AAX88848.1 Homo sapiens (human) unknown
UCUAAACAUCUCAACUAUCAAGGCUCUCUUAAAAGACUUCAGGUACUGUCAACCUUCAACUCUAA
GAAAGUCUCGAAGAAAGUGAUUGAAAAAGUCUAGA
>EMBL_CDS:AAX88914 AAX88914.1 Homo sapiens (human) unknown
AGGACCCGCCUCUGCGCCAGAGACCCUUUCGGAAAAUCGUAGACCCUCCGGUCCAGAAGUCGAAG
UCCU
>EMBL_CDS:AAX93134 AAX93134.1 Homo sapiens (human) unknown
CCACGCACAUAAACUCGUCGAGCCGGUCGACGUCUCCCAUGAACGUCUAGAAGACGUGGCCUCAG
UCGUCCUUCGGUAGGUCCCUCUACAGUUAGUAGAAGACGUCGUCGGCCGUCCGGAGCUUCUUCAU
GUGCAUGA
>EMBL_CDS:AAD23604 AAD23604.1 Homo sapiens (human) DYN2_HUMAN [AA 474-870]
CGGCUGAGGAGGUACUCCGACGAGACCAGACGGCUCCUCAUAUCCAUCCGGUCGUCGAGCACCAC
CUACUUCCG
>EMBL_CDS:AAD19817 AAD19817.1 Homo sapiens (human) Human homolog of Mus musculus wizS
CGGGUCCAUCGGUCACCGACUCCCGAACCGGGGUCACUACCCGCAGUCCGACUCGAGUGCUCCUU
GGACCCG
>EMBL_CDS:AAD21217 AAD21217.1 Homo sapiens (human) Putative map kinase interacting
GUCCAGGAGCAGCACGACCCGGUCGACGGCCAAGUACCGUUACCGGAGUCGGCGCUUCCUGCAC
>EMBL_CDS:AAY24351 AAY24351.1 Homo sapiens (human) unknown
CUCCACGUCCUGGUUCUAUUAGUCCUAAUAGUGUACAAGGUUAUAUAUCGUUAUGUGACUUACCA
AAACAACACCCUAGAAAACCUAUUUUUUACUGACAACACCAAGACAUCGGAG
>EMBL_CDS:AAX93195 AAX93195.1 Homo sapiens (human) unknown
CUCAGAACCAACUACAUUAUAUACUCAGAACUUCCGUCACACUAAAGAAAGACGUAACGUAACGA
CAUUGACUAUAAUGACGACGAUAAGACAACGUUACAUAACAGAAGUAGACGGUCCUGAG
>EMBL_CDS:AAD39266 AAD39266.1 Homo sapiens (human) Human Fc gamma BP [AA 1-2843]
CACCGGGACACCCCGACCUCUCCUCGGGUGUCUUGAGGAGGAAGACUAUGAAGAAGAGGUCGAGC
CCUCCUGUG
>EMBL_CDS:AAX93216 AAX93216.1 Homo sapiens (human) unknown
CACCCAGACCAACCGGAGAGUGAACCACAGAACUUUUCAUCUCCCGAUUCUUUUGGGUCCACUCU
CCUUUGUAUAGGUA
>EMBL_CDS:AAY14753 AAY14753.1 Homo sapiens (human) unknown
GACCUCGGUCCUUCCAAUGGAGGCUGGCACCAUCCUGACCGUUGACCCGUCGGUCCCGAGAAGAA
CGGUGACUCUCCCACCGGAGAGGGACGAGAUC
>EMBL_CDS:AAX81988 AAX81988.1 Homo sapiens (human) unknown
ACCCAAACAAAAGGAGAUCUCAUUAUUUAAGAUAAUGGGCCCAUUAUCCUAUAACGAACGGGU
>EMBL_CDS:AAX88939 AAX88939.1 Homo sapiens (human) unknown
UUUCACUAAGUUUUCUCCACAGAAGGAGUAGAGACGUGUCAGGAGAACCACACUGUCUACACUAC
CUACCACGUGGAGAUAAAUAUAGUAGAAA
>EMBL_CDS:AAY14651 AAY14651.1 Homo sapiens (human) unknown

CACGUGUAUCAGUAUCACCAUCUGUCGCAGUGAAUCGACAAAGGGUGAAAACUGUCGGACGUG
>EMBL_CDS:AAF03518 AAF03518.2 Homo sapiens (human) unknown
CAACCGAGGUUCUACGGUUCGGGUACGGUGGGACAAAGACCCCAUGAGACCUCCCGGUGACGAAU
UCCGAACCGAGAGACCCGGGUCUUUUACCGUCCCGUCACCGGAGACGACCACCCG
>EMBL_CDS:AAY14751 AAY14751.1 Homo sapiens (human) unknown
UCGACCACGUCCUGUACCAGGUGUCUCCCGUGGACCGACUUCAGUCGUGGGUCGAGGUCCCACGC
CCGGGUCGAGUCCCGGGGACGUGUUAGAGGGACCGUGGACCGGAGGACGUCGUCGA
>EMBL_CDS:AAD23606 AAD23606.1 Homo sapiens (human) R31665_2 [AA 1-673]
UAGGGUCACAAGGGGAUUGUCUCUCAUGAGGUCUAAUAUCGAACACUCAGAGAGACAAUAGUGAC
GGACCCUA
>EMBL_CDS:AAX93132 AAX93132.1 Homo sapiens (human) unknown
AUCGCACCCUACAUGUUUGAAGGACAUGUCCUCCGACCUCGGAAAACACAUCCCUCUGUCGUCGG
UCCGAU
>EMBL_CDS:AAC68599 AAC68599.1 Homo sapiens (human) cell cycle-regulated factor p78
CCCGACCCACCGUGUCCUCACGACCCCCGAAACCUAUGGAAGAAGAAGAGUGACCUCCUCCCAAG
UGAGGGGCUUGUCGCGGGACCAAGGUGGGGUGACCGGG
>EMBL_CDS:AAC64294 AAC64294.1 Homo sapiens (human) PKY protein kinase
CUUUAUUUACGACUUACACCCUAUAACCAUAAGACAUUACUUAAUGAGGUUGACUUUCGUAAAUA
AAG
>EMBL_CDS:CAB22795 CAB22795.1 Homo sapiens (human) hypothetical protein
AGUACACGAACUACUACUAAAAAGAACGGGUUCCAACACCUGUUCCUUCUGUUUCUCACGUACU
>EMBL_CDS:AAC97080 AAC97080.1 Homo sapiens (human) lambda 1 immunoglobulin light chain
AAACCCCCGGCGAGGACCCUCUACGACCAUGGUCCAAUGUCAUAAUGAAGGCUACAACCUCGACG
AAGGUCU
>EMBL_CDS:AAD17540 AAD17540.2 Homo sapiens (human) nectin-like protein 1
CGAGGUUUAGGACGAGGACGGUCAUCGGCAGGACCCUCUCCAACCGGGGCGGCCCGCGGGUCGUC
GUCCGCUUGUCGUCCUCGUCCUCGUCCUCG
>EMBL_CDS:AAC16813 AAC16813.1 Homo sapiens (human) immunoglobulin lambda light chain
UCACCGACACAGGGGUCUCUACCUCGGUCUCUUAGCGAGUCCCUAGGGACUCCCGGCGAACAGUA
AAACUAUCUACUGGUAGUAUCCCCUGACCGGACCGAAGACAACUUUGGUCAUUCAUAAAAAUAGG
AGGUUAAAUAGAGGACUCGUCCACUACGACCGACAGACAGGACCCCUGUGCCUGUGA
>EMBL_CDS:AAC39660 AAC39660.1 Homo sapiens (human) retinal pigment epithelium-specific
AACUGCUCCGGGACUUUUCUCUUGAAGUCCGAGGUCGGUCUAUCAGAGCAGUGACGUGUCUUAAC
GUCACCGUCAACAUAACCCCUCGCACUGAUUUAAGAACGGACACAGUCGGAACAGUU
>EMBL_CDS:AAC39892 AAC39892.1 Homo sapiens (human) RNA polymerase I 40kD subunit
AUGUCAAGCAACCCCAGUCUCCUUAGAAAUCGUCGUACCCCCAAGGCUCACGUAGACUGGACCUC

UGCUUUGACAU
>EMBL_CDS:CAB23081 CAB23081.1 Homo sapiens (human) hypothetical protein
AGUAGACACCCCCUGUGUCCCCGCCGGAGUCUCAGUGGGCAUCGCCCCUACCCCGUCGGAGUCAC
AGUGGGUUCCACC
>EMBL_CDS:AAC18086 AAC18086.1 Homo sapiens (human) diphthamide biosynthesis protein-2
CCCGUGGGAGUAGGACUCAGAAGGGACAUAGACCUGUCGUCCUCCUCUUCCCAACUGGACCACGG
G
>EMBL_CDS:AAC79696 AAC79696.1 Homo sapiens (human) MRP6
CAACUCCUACAACUCUUGACACUCUCAGUGUUUCCGAAAGAGACGUAAGUAUCGUAAGAGCCG
>EMBL_CDS:AAL86699 AAL86699.1 Homo sapiens (human) alpha 1 type XXI collagen precursor
CUAGGACCUUGUGGACCACAGGGACGUUAAGGGACUAUAGGUCCGAAUGGUAGAACUGGUCCAAC
AGGUCCCAUCGGUCCCAAGGGUCCGUCAGGUCCCAG
>EMBL_CDS:AAQ05538 AAQ05538.1 Homo sapiens (human) Ig heavy chain variable region, VH3
CCCGGCACAGAAGCCGAAGGUCUGACAAGUCAACGUCUAUGUCAUACAAGGACCUUAACAGAGAC
CUCCACCACUUGGCCGGG
>EMBL_CDS:AAM43838 AAM43838.1 Homo sapiens (human) U-cell activation WD repeat protein
CUGUUCUUCACCGAGAGAUACCUCUUUACAGUCAGGCCGUUAACGACAUCUCACACGUAAAGAGU
AAACCAACUAAUUAAAAAACAG
>EMBL_CDS:AAL67806 AAL67806.2 Homo sapiens (human) esophageal cancer associated
CUUAAACAAUGUAAAGUCCUACCUACGGAGGUGCAUUUUAGAACCUUACUCAAGGGACUACCUCC
GUUAGAACUUUUUGGUUAAA
>EMBL_CDS:AAK27221 AAK27221.1 Homo sapiens (human) junctional adhesion molecule 3
UAGGCACCCGUCACCAUGUAGUAACGCUAUGGUCGACAUCACUCCGGCCCCCACCGGGAGUGAGA
GGACCGUCACGUCACAACGGUAGAACGGAUGACCA
>EMBL_CDS:AAM77566 AAM77566.1 Homo sapiens (human) IL-17D
CGGGACAAACAGCUACGACCUCAACUACGACAGACGCAGGAAGAGGCCGAGCCCCUGCGUCCACG
>EMBL_CDS:AAK74120 AAK74120.3 Homo sapiens (human) mucin 16
CCAUCUCUCGGACCACUAGUGAAGUCACCUAUACAGAACACUGUAACACCUGACUAGUCCCGAUC
CAGGAGACGA
>EMBL_CDS:AAK68033 AAK68033.1 Homo sapiens (human) DNA methyltransferase 2f
ACGGUUCUCUCCAACCUCUAUUAUAUUUGAGAACCAUGACUUUCGGUGUUAAAAGAUAACAAACA
UAGUUCUCCAGAAAGUUAGCAGAACCGU
>EMBL_CDS:AAP97715 AAP97715.1 Homo sapiens (human) excision repair protein ERCC5
AAAAGUGAAACCACAGUACUCCUAAUCCAGAAUAGAAACCUAAAAAAACUCGAAGUACGGUGGUA
AGACUCUU

>EMBL_CDS:AAM12641 AAM12641.1 Homo sapiens (human) regulator of G protein signalling 3
CGGGGUGAACUCCCGAAGGAGACUCCACCCGAACUUACUGAAGUAGUAAAACAGACGGAACGGGC
GCCCUCCCCG
>EMBL_CDS:AAK96045 AAK96045.1 Homo sapiens (human) testicular soluble adenylyl cyclase
CAGAAGUAAAAACAAUUUUGCACCAGUUUCUUUAAGUUCUAGAAAAGAGAAUAAAGAAGUCCUGA
CGCUAAAAGUCCUUUUUUACGUCUA
>EMBL_CDS:AAL77058 AAL77058.1 Homo sapiens (human) multivalent protease inhibitor
AGUCCGUGAGCCGGCGGCCCUUUCGGCACGGUAACCCCGACGAGAGACUCCACCGACGUCGGACU
>EMBL_CDS:AAL08625 AAL08625.1 Homo sapiens (human) hematopoietic zinc finger protein
CCCCUACCCCUCGGUCCGACAAAGAGACCCCUAGGACCCCGACCGGGGUCAGGUAAGAGGUACCU
UUGACCUGCCCCACGAUGUGGUAGGGG
>EMBL_CDS:AAL65133 AAL65133.2 Homo sapiens (human) ovarian cancer related tumor marker
CAACAGUGACUCAGUCGAUCCUGUCUCCUUCCUCUAAGUACACAUAUUGGACUCCAAUGGUGUAA
CCAGUAGAGGUCAAAGAGACAUAGACAUCACUGAAGUCACCACCG
>EMBL_CDS:AAL24502 AAL24502.1 Homo sapiens (human) deleted in azoospermia 2
GUCCGUUGACUAUAGGUCACCACUGGACUUUACCACUUGAACCUAUUCGUCCUUUACGGACUAUU
AAUAUAUGUCCGUUGACUAUAGGUCACCACUGGACUUUACCACUUGAACCUAUUCGUCCUUUACG
GACUAUUAAUAUAUGUCCGUUGACUAUAGGUCACCACUGGACUUUACCACUUGAACCUAUUCGUC
CUUUACGGAC
>EMBL_CDS:AAK27358 AAK27358.1 Homo sapiens (human) Bcl-Rambo
CCGAAGUCUUUAAAGAAACAGGUCUCUAAAAUCGAGAAGAAGUUAAAGUCAAAAUUGAAAAUUAU
UUUAAAGAACUAGGUCUCUAACACUUCGA
>EMBL_CDS:CAC15059 CAC15059.1 Homo sapiens (human) putative protein kinase
GACCCAACGACCAUGACAUCAACAACUACAACUAGACGGAGUCCAAGAAGUCCAAGAUGAACACA
UCUUUGACUUCGACUUCACCAUCUUCGUCAUCCAUACGGCCACGAUCGUCUUC
>EMBL_CDS:CAC10401 CAC10401.1 Homo sapiens (human) CDC2L5 protein kinase
UCGUACCCUCCGUUUCCAUUGCCACCAUUACAUCUCCACUGAUUUCGAUGUACAACGAGGAAGAG
GAACCGAGGAUACGA
>EMBL_CDS:CAB65596 CAB65596.1 Homo sapiens (human) peroxisomal branched chain acyl-CoA
GAAUGACGGUGUACACGGGUCCGGCACCACAUCUCGAGGCCCGUCUCCUUCAGCCGACGGGAGAC
CCGACCUGUGGACCGGUCCAGUCCACGCCACUC
>EMBL_CDS:CAA06213 CAA06213.1 Homo sapiens (human) integral membrane protein, Ump21-I
CCAGGAACACUUAGAGGAGUGCCUCCGUGAACGCUCUCAAUUACCCGUCUACCUUCCUCUACCGU
UCCUGG

>EMBL_CDS:AAO62630 AAO62630.1 Homo sapiens (human) transforming acidic coiled coil 2,
CCGUCACGAGUCAGACUUCCUCCGGUGUUGAGUUUCAUCGAAGAGGUCCAAAGAGGCCUCGGACG
ACGAAAGGACCCAGAGGACGUGGUCGCGACGG
>EMBL_CDS:CAC00640 CAC00640.1 Homo sapiens (human) rhomboid-related protein
CAGGUCCCCGUCGAGUACCGACACGUGGUCCGGUCACUUCCACAGGCGUGGCUACUUCGGACACA
AGAGCCCCAGGUCGACGACGAGGACCUA
>EMBL_CDS:CAC34245 CAC34245.1 Homo sapiens (human) U-cell receptor beta chain VJ
ACGACGACAGAAGUCCCGAGUACAACGAGUGUCAGUCUCAACUCUUACAAUCCAAACCCGCCAAC
UACCCUUUGAACAGUUACUGUUUAGGUGAGAGUAUACACCGGAGUCUCGGGACUAAACGUCAACG
C
>EMBL_CDS:CAC17609 CAC17609.1 Homo sapiens (human) importin7
ACCGCUAGAAGAAGACCUUAUCAUAUUCCCCUAUAUAGGGGACCACGACAAAGAGCUAGUCCGGU
>EMBL_CDS:CAB65100 CAB65100.2 Homo sapiens (human) NICE-4 protein
CAUCGUUGACUCGAAGCUCAGCUCCGACACUGUCACCUCAACGACGGAUCCGACUCCGGGUCCGA
UG
>EMBL_CDS:CAD24023 CAD24023.1 Homo sapiens (human) putative alpha 1,3-fucosyl
ACACCCGAGACCCGUUGAGUCCACCCAUAGAAGACGGAGGGUAGAAAACCCACCAUUCGGGAAAA
GGACUUCGGACUAUAAUCGGGUGU
>EMBL_CDS:CAD58724 CAD58724.1 Homo sapiens (human) NOSURIN protein
ACGUCAAAGAAGGUAAUGUCGGACCUAUAGAAAAAGUUACAGUUAGAACGACUACCGUGUCACUU
AGACGCACACCGUACACCAGUCCCAAACCGGUUUUUCUCUUUAUACAACCGACAUGACCAAAUUC
AAUAACGU
>EMBL_CDS:CAF25192 CAF25192.1 Homo sapiens (human) Xin A
CCACCCGGACCCCCGGCACCACGACUCCGGUACAACAUUCGACCCGACCGAUACAGGUGACCCUG
UAGACCGGUCUCCAUACACCGGGACCAGAUGUCAUCACCCUUGGAACCGACCGA
>EMBL_CDS:BAC04859 BAC04859.1 Homo sapiens (human) hypothetical protein
AUACAUACACACGUGUCCACGGACACGUACACAUGCACACGCGUAUCUGGACAUACAUGUAGACA
GAGGUACAUGCAUACAGACACACACCGUGUCUACGCUAUGACGCCCACGUAGACGUAUACACACG
UGUCCACGGACACGUACACAUGUAC
>EMBL_CDS:BAG53993 BAG53993.1 Homo sapiens (human) hypothetical protein
CCAAAGGAAUCAACAUAUGUCACGACUUCAAUACCAUUAGUAUCAUAAAUAUUAUUGACCACUUU
GG
>EMBL_CDS:BAC85326 BAC85326.1 Homo sapiens (human) hypothetical protein
CCGGGACAGAGGGUUGGGAACAUGGUCACGACCCGAGUCUGGGACCAUGUCCGGACCCCCUGUCC
CUGG
>EMBL_CDS:BAB55198 BAB55198.1 Homo sapiens (human) hypothetical protein

CAGUGACUGAGCCCCAAACCCAUCGAGGAGUAAAAGGCUUAGUGACCGGACCAAGGAACCCCCUA
GGAGUGAAAGGCUUAGUGACUAAGCCCCAAACCCUUCGAGAAGUAAAAGACUCAGUGACCG
>EMBL_CDS:BAB15388 BAB15388.1 Homo sapiens (human) hypothetical protein
CGACCACAACGUCCCGAAGAGCACACAUACGACGACGUCGGAGACCCGACCGUGGUCGUGGUCCG
ACUCGGACAGGAACCCGUGGUCGUCGAAGGUCCUCUUCAGUACGUCGAGGUGUCCG
>EMBL_CDS:BAC11516 BAC11516.1 Homo sapiens (human) hypothetical protein
CCCACUCCGCGGGAACAUGUGUGACAACGAGUCGCGGAGAGGCGCGACGUCGACCUUCUUCGACA
ACUCUAUCGACCUCACGUCCCCGCUCGGG
>EMBL_CDS:BAG53483 BAG53483.1 Homo sapiens (human) hypothetical protein
UACAUUGAGUCUCGUCAGAGGUUCAAAUGAUGGAAGAAGACUUCCCAAACCUAAACACUAGAGAC
GACCUGUUCAACGUA
>EMBL_CDS:BAG52312 BAG52312.1 Homo sapiens (human) hypothetical protein
CCCUCUUACUGUCCACCAGGGACGAGGACUGUGAAGACACCUCCUACCGCCCCUACCGUGGUAGA
GGA
>EMBL_CDS:BAG51468 BAG51468.1 Homo sapiens (human) hypothetical protein
CCUCGAGGGACCCUCACGGGGUCUGGGUUGGGUCGGUAGUGACCCCGAGACCCCUGUGACAACGG
ACCCUGACCCGAGG
>EMBL_CDS:BAC87399 BAC87399.1 Homo sapiens (human) hypothetical protein
CGGAAAAGGUGUAAGUGAUGUAUAUAUUCCAAAAAGAGGACAUACUCAAAAGACUACACAUCACU
CUGACUUGAAACAUCUCUUCCG
>EMBL_CDS:BAG54552 BAG54552.1 Homo sapiens (human) hypothetical protein
AACGAUGAGGUACCCCACCUAAAAGUACCGCUGCGGAACAAUAGAACCAAUAACAACGGUAUCCU
GAAAGGUUUCGUGGUGUACCAAACCAUU
>EMBL_CDS:BAG51912 BAG51912.1 Homo sapiens (human) hypothetical protein
CCCCUCUCUAGUAACCGUCGUUCUAACGCCGUUGUCGGUCAGACUUCGUUGCGAUCACAGAAAGG
GG
>EMBL_CDS:BAB15349 BAB15349.1 Homo sapiens (human) hypothetical protein
CCGAGGGACCAGUUGAACCCUACGGGUCUGGUGACACCCGACCCCCUUGGUAGCCCCUCGG
>EMBL_CDS:BAB14766 BAB14766.1 Homo sapiens (human) hypothetical protein
CAGGAGGAGGUCAUGGACCUACAUCCCCGACGAGGACAGGUCGAAGGGGAGGACCUGCCUCCGGA
GGUCGAGGACGUGGGCCCGGACCUCGUCCCG
>EMBL_CDS:BAB15157 BAB15157.1 Homo sapiens (human) hypothetical protein
CGCACACACACACGUACACACGUCAUCUUCGAGUAUGCAGAGAGACGACACGUAUGUGCACACA

>EMBL_CDS:BAC86835 BAC86835.1 Homo sapiens (human) hypothetical protein
UUCGUACCCCAUAGGACGUAUAAAUAUUCCGACAUUUAGAAGAAAUACUGCCAACUUCUCCUACG
AGACACAAA
>EMBL_CDS:BAG53471 BAG53471.1 Homo sapiens (human) hypothetical protein
GAACGGCACUUACCGCGGCUGGUACGCGAGUUAGAGGAAUCCCCUCUGUCCGGAGUAGGACGAGA
CGAGGACCUCCUCCAACCACUGCACGCACCACUUGAGUGCUGUCC
>EMBL_CDS:BAC86126 BAC86126.1 Homo sapiens (human) hypothetical protein
AACCACGUAGGGAGCCAAAAGGACCGGUCCCGUUACCGACGCUACGGACCAUCCGUCUACCGACC
GGGCUACCGGUCCUCCCCCUACAAAGGGUU
>EMBL_CDS:BAG51766 BAG51766.1 Homo sapiens (human) hypothetical protein
UUAUAACGUAAGAGGAGGAUUAAGGUGGGUCCCGUGACACUCCUAUCUGGAAGGUAUCGGUAACC
CCUUACACUCUUACAUUACAA
>EMBL_CDS:BAC87609 BAC87609.1 Homo sapiens (human) hypothetical protein
CCUCUACGUCGGCCCUCCUUCUCGACCCGGGCCUCCCCCGCGGCCCUCCGACGUUCACCCAGACU
CUCCGGUUGAACUCCUCCGGACCGGAGACGGAGGGCGUAACGGGUCGACAAGGAGGACCGACGUA
GAGG
>EMBL_CDS:BAG51223 BAG51223.1 Homo sapiens (human) hypothetical protein
GUCUCGUCAUUCUUCUCGUUGGUAGAGACUCAGAGGUCUCGAGGAGAGACGUCCUUCCAGACCCA
GGAGCCCGUGACCCUGGAGCGAGAGGACGUCCCAGACCUCGAGGAGAAGGACCACACCUGCGACG
AGAC
>EMBL_CDS:BAB70820 BAB70820.1 Homo sapiens (human) hypothetical protein
AAGACCCUAUGUAAGACCAACAUUUACACGGGUGACUACAAAGAUAUUCCACCUGUCUACGUAAA
CGUGGCCUAUAGAAGUCUU
>EMBL_CDS:BAA92130 BAA92130.1 Homo sapiens (human) hypothetical protein
UCUUCAACUCCCAUUUUGUACAAGUAGUAAAAACAGAUUCAGUGAUUGUCUACAAUUCUUACCGU
UUACAAGUGGGAGUUAGAGA
>EMBL_CDS:BAG54675 BAG54675.1 Homo sapiens (human) hypothetical protein
AGGAGGUCCCUGUCCUAUAGUGAGAAGAGGAAGAGACCGACCCGCUCGUCAAACGCCUUGUGUAA
GGACACAGGCACCCGACGUCGGACCGACUUUCUCAAGUAUAGUGGGACCCCCC
>EMBL_CDS:BAD18708 BAD18708.1 Homo sapiens (human) FLJ00261 protein
CUCCUCUGGUCCCGGAAGGCUCCGACGGCGCCUCUCGAUCCGGGCCCACAGAGCCUGUCCGAGAG
GUCGGCGACGACCGAGACCUAGAGGAA
>EMBL_CDS:BAC11282 BAC11282.1 Homo sapiens (human) hypothetical protein
CCGAGGUUCACGACGACUCCAACCUCGUGUCCCUUACCCUCCUCCGUGUCCGGAACUCCGUCCAC

GGUGGGACCCGGGCGACGUCGACUUGGAGGUCGUCUCGGAACCACGG
>EMBL_CDS:BAG54709 BAG54709.1 Homo sapiens (human) hypothetical protein
ACAGUGAGGCUCGACCUCCGUCCCCACGUGGGUGGAAUCCUCCUUCCAACAGGCACCGUCAGAGG
UUCCUUGACCCCGUGACGACCGAGGUCCUACCCCCACCGU
>EMBL_CDS:BAC05039 BAC05039.1 Homo sapiens (human) hypothetical protein
AGUCUCGACGGUCAAUCUUCUACCAAGGGAACUCCCAACUUUCUUUAGAAACUUUAAAGUGGAAG
UACUGGAAGGUCAAACCGUUACGUAAAAGAACACCGUCGUGACU
>EMBL_CDS:BAB15527 BAB15527.1 Homo sapiens (human) hypothetical protein
UUGAAGUGUUCCUACUAAACCGACGACGGUUCCUGUCAGACCUUCACUUGUCCGAAAAGGCCUUC
ACGACCGCACCGAAAGUCUAUCUCGAGUAUAACCAUCAUUGGGUUCAGUCACAACACGUUUCAA
>EMBL_CDS:BAB15113 BAB15113.1 Homo sapiens (human) hypothetical protein
ACCCCGACGUCCGUAACAGACCCACUCGGAGGUGUUCCUUGGGGACCUCCGGGAGGACCGAGUUC
CAUCGGGGU
>EMBL_CDS:BAG53044 BAG53044.1 Homo sapiens (human) hypothetical protein
CUCGUCAGAAUUUACGUCUAAACUGGUUAAAUCACCCUGUGUAAUACAAAACCGAGUGAUUUCCA
UAACAAAGUACUUACCUGACAAG
>EMBL_CDS:BAC04529 BAC04529.1 Homo sapiens (human) hypothetical protein
GUUUGAAAGGAGUAUAAGUCUUGAAAGUAUCCUAAAGAGAGGACACACGUAAAAGACCACACAUU
AUUCCAAAC
>EMBL_CDS:BAG54259 BAG54259.1 Homo sapiens (human) hypothetical protein
UCGACCCUCCAGUCCAUCCGCUACCACGACCGGGCGACGAGGUCGAGCGAUCUGUCGUCCCAGUC
GAACACUGAGAACCGGAAGUCGA
>EMBL_CDS:BAD18751 BAD18751.1 Homo sapiens (human) hypothetical protein
CCUGGGUCUCCGUAGGGACGGGUCUGUAGGUGUGAGUCGAGUCCCAAGACACCGGACACACACCU
UAACAGACGUCGACCCCUACGUCGGGCAGG
>EMBL_CDS:BAD96865 BAD96865.1 Homo sapiens (human) solute carrier family 27 (fatty acid
AGUGCCAGUAAACCAAAGACACCACUCAACGUAUUUAUGGCUUCGUCAAGUGGCUAUAUGACUUA
CUGUCACU
>EMBL_CDS:BAD96221 BAD96221.1 Homo sapiens (human) hypothetical protein FLJ11848
GACUUCGACAUUGGAGACCCUACGGUAGAUCCGGUUAAGGAACUGUUCAGUCCGACGGUAUGUUU
CUACCAAAGGGACCCCCUACUGUCGAGUC
>EMBL_CDS:BAD97219 BAD97219.1 Homo sapiens (human) monoamine oxidase A variant
GACCAGUUGCACUCACUGUCCUACCAAGUCGAAGUGAACCAGAGGCUCCUCCAGGUAAUAGGCAA

GCGAGUGAACUGGUC
>EMBL_CDS:BAD97254 BAD97254.1 Homo sapiens (human) tektin 3 variant
AGGUCAGACCAGACUCGGUGGAAGUCCUUCCGUCAGAACAGGAACUACCGGAAGAACUACCUAAG
AUACCAGUAAAGUCAGACCU
>EMBL_CDS:BAD96196 BAD96196.1 Homo sapiens (human) aspartyl-tRNA synthetase variant
AACCCAGUCCGUACCAUAUCUUUCCAGAAUGUCGGUUACCUAUAAAUAGUUCUUAUAUUUUUAGA
CAUAGUAUGAAAAGGAAAUGGUUUACUGGGUU
>EMBL_CDS:BAD97122 BAD97122.1 Homo sapiens (human) sarcosine dehydrogenase variant
CGGGGUGAGUACGGGUCGAACCGGUCCACCAUGUCCCAGACCGUCGGGUUCGACGGAGGUGGUUA
CUGGUGGUGCAACCGGCACGACCCGUCCCCG
>EMBL_CDS:BAD96931 BAD96931.1 Homo sapiens (human) carbohydrate (chondroitin 4)
GUCGACGACGGAGGUCCGGUCCCCCUAGAACCGCUUGGUCAGGAGGAGGGUCGACGACCGCCAGG
ACAAGGCCAUCGAGCCCCCCUUCGCCUCGACGGCCAGGUGGACCUCAUCGACGUCGUCGAC
>EMBL_CDS:BAD96995 BAD96995.1 Homo sapiens (human) AU rich interactive domain 1A
CGUGUCCCCGUCUCCCUGGUAUACCCCCGACCGCACACGGUACCCUUCCGGUGCUCGGAAGCACC
AACCGGGAGACUAGACACACG
>EMBL_CDS:BAD97289 BAD97289.1 Homo sapiens (human) Na+/K+-AUPase alpha 3 subunit variant
CCCCCGUGGGAAGUAGUGGUCGUCCAUAGCCAACAGCAACCCCAGGAGCCAGAGUACCUACCUCU
CGACCAUAAACAACCACCUUAACUUCCCUUAGAG
>EMBL_CDS:BAD96814 BAD96814.1 Homo sapiens (human) aminopeptidase-like 1 variant
GUAGACCCCACACCGACUCCUGCCGGUCCCGACCCCCUACGUCCCGCCGAAACGGUUGGGGUAUC
UAAGGAGGUUUAGGAGAGCAGAAGUCAAGGAGUAGGGCCUAC
>EMBL_CDS:BAD97332 BAD97332.1 Homo sapiens (human) pinin, desmosome associated protein
CCGACCCUGACCUCAUGACCAACCCUGACCCUAACUUCGACUUCGACCCCGACUCUGACUCCGAC
UCCAACUCGGUGUCCGAGUCCAACCCCGAGUCUAAGAAAAAGAAGAAAAAGGAGUCUGAGGAAAA
GGUACAG
>EMBL_CDS:BAD96423 BAD96423.1 Homo sapiens (human) calcium/calmodulin-dependent
CAACCGUCUGGGUGCCCACGAAGUCCCGGAGUACCCGACACUACGCGAACCGUCCCAACUACCAG
UCG
>EMBL_CDS:BAD96332 BAD96332.1 Homo sapiens (human) PHD finger protein 10 isoform a
GACCGAAGUUGAAAGACUCAUAAGACAACAACGUAAACCCUUAUGAGAAAUAUUACCAGACAUUA
AGCAACUGCAAGAAAAAGAACAUCUUAUUGUCUUAUGAGUCGUACAAACCGACC
>EMBL_CDS:BAD97166 BAD97166.1 Homo sapiens (human) piggyBac transposable element
GGACCAUCGUCUUCACGGCUUUCGCGUCCGCCGUUUAGAGGACUCACACCCUCGACGGGAGGACA

CUCAACGUGUGGACGAGGGUCC
>EMBL_CDS:BAD97228 BAD97228.1 Homo sapiens (human) phosphoserine phosphatase variant
GGUCGAGGGAAUACGGCCCCCAGUCCACACCCCCAACGAGACGAUACUCAGAGACGUGGACGAGG
GACCUCCCGACC
>EMBL_CDS:BAD96811 BAD96811.1 Homo sapiens (human) galactosidase, beta 1 variant
AGGUCUGGACCGGGGACCAGGUAGGUCCUUUGACCUAUUUCCACAGGACCCCGUUCAGACCC
>EMBL_CDS:BAD96436 BAD96436.1 Homo sapiens (human) RNA binding motif protein 28 variant
CGGGUGGUCGGAGUAAGAGACCUCUUCGUCGAUCUUCCGUAAAGACUCGACGAAGAACUCAGUAC
UUGACCCG
>EMBL_CDS:BAD96821 BAD96821.1 Homo sapiens (human) carcinoembryonic antigen-related cell
UGACACUAGCAGCACUGACACCAGGAUAACUCCGGUCACAGACUCAAUACCCGAACCGUGCAUAU
CCUAGGUGAUAAUAAGUGUCA
>EMBL_CDS:BAD96726 BAD96726.1 Homo sapiens (human) 2',5'-oligoadenylate synthetase 1,
ACCCCAAAAAUUUCAGUAUUAUGAAACAGGUCAUCUACGUCUCAACGACCAUCAAAUACUGAUUA
AGGU
>EMBL_CDS:BAD97042 BAD97042.1 Homo sapiens (human) splicing factor 3b, subunit 4 variant
CUGUAGGGACCUACCCCGUAGGGUGGGCCACCCUUACCCACUCCUACACUCACAGGUACUGGUCC
UACAG
>EMBL_CDS:BAD97300 BAD97300.1 Homo sapiens (human) DKFZP586B1621 protein variant
GAGGACCUAACGAGACCGGCGUGCCGACACCCACCACGGUGUCAGCGGCAGUGGUCGUCGGGCCA
GGUCCCGUAAGUCCAC
>EMBL_CDS:BAD97305 BAD97305.1 Homo sapiens (human) glycine amidinotransferase
UCCUCUACUAUACGUGAGACAUCAGACCUCGUUCUACGGAUGCGUAGGUAAGUUACGGAUCCAUC
AAACAUUGGACCGAAGAGACACGUUUUUAUAGAGAA
>EMBL_CDS:BAD96710 BAD96710.1 Homo sapiens (human) RAN binding protein 3 isoform
AGAGACAGUCUUAGAAGAGGCGGAAGUCCUGACCUCGGCGGUCGACUGGAGGGACGGUCAAGAGC
GCCUUUUCCUCCUUCGACCCGAAGUCCUCGCCCUCCGCCUCCUCACGACCGACUCC
>EMBL_CDS:BAD96976 BAD96976.1 Homo sapiens (human) nibrin variant
UCGAAAAAACCUUCGUAGAGUGAUAGUAGGACUUCAAACAGUAACAAUCUAUAAAGAAAUCGACU
GGUAUCACUCAGAAGGAACUCAA
>EMBL_CDS:BAD96686 BAD96686.1 Homo sapiens (human) PDZ and LIM domain 3 variant
ACCUGAGACAGGGUUUCGGCAGUUAUCGGUCCUACUGUAGAGGUCCUGUGUCCAACCGUCGACGG
CGGAACGAAGGACCACAUUAGGACCACUACUGGUUUCCGACCAACUUCAGAU
>EMBL_CDS:BAD96624 BAD96624.1 Homo sapiens (human) connector enhancer

of kinase
CCGAAACGUCCAAGAGACAGACAUCGGACCUCGAGUCCCGGACCUCGACAAGGUGGGGCGGGUCC
UACUCGAGGACCACAGGGUCUCUGGCGUGUCGG
>EMBL_CDS:BAD97292 BAD97292.1 Homo sapiens (human) inositol hexaphosphate kinase 3
CCCAACUUCGAGAAGGAGAGUUGGACCAAAGGCGACCACAGAAGGUGGUCGCUCUUCCGACCUCA
CAACUCCACCCCGAGCCUGGA
>EMBL_CDS:BAD97230 BAD97230.1 Homo sapiens (human) cytochrome P450, family 2,
UUGUCAUUUAAGUCUGACAACUAACACGAAAAGGACAAGGUAAAACUAGUCCUUUGUUAGUUAUU
UCAGGGCUCGUGACAA
>EMBL_CDS:BAD97047 BAD97047.1 Homo sapiens (human) integral membrane protein 2A
AGGACCCACCGUUGAGCCUCGAGGAACGGCCAGUCAUAGACUCAAGACUGGCACGCCGAGUCCU
>EMBL_CDS:BAD97004 BAD97004.1 Homo sapiens (human) mutS homolog 2 variant
GGUGACCAAUUGUUCAGAAACAGGAACUCCCCAAAAUGUGAAUAAGUCGUUCCGUCGGUCUCUGA
CUCUCGGUCACC
>EMBL_CDS:BAD96438 BAD96438.1 Homo sapiens (human) fibroblast growth factor receptor 1
CGUCCUCUACUCCUUCCGGGGACACGUUAUCUACUACUAGAGGUCCAUGUCCCCGCUCCAGUAGU
GACG
>EMBL_CDS:BAF82658 BAF82658.1 Homo sapiens (human) hypothetical protein
AGUUUCCGUCACACCAGCCGGACCUUUAUGGUAGAGGGAGUCCUCAGGAACGGGAAAGUGAGACA
GAAACU
>EMBL_CDS:BAF83734 BAF83734.1 Homo sapiens (human) hypothetical protein
GUCGCAGACUAACCGACCAGGGACCUCCUACUGUCCUCCGUCGGGUAGGUUUCUCCGAGGAAAAU
AGAGGUCGUCCCGGAACCGGUCCUACGAC
>EMBL_CDS:BAF83800 BAF83800.1 Homo sapiens (human) hypothetical protein
GCCUAGGAGGACCCUCCUCUACACAGACUCAGUACUGCUCGGGGAGACUCCCCUCUUCCUAGGCC
CCACAGUACUGCCCGGGAGGAUCCUCUACCUAGGC
>EMBL_CDS:BAF83741 BAF83741.1 Homo sapiens (human) hypothetical protein
GGUCACUCCUCCACCCACUCUACCAUGGUCGACUACCGUGUAUCAGGUUCGGGACGCAUCGAAGU
CAUGCCCUCCUUCCCUACGAACACUGACACAGUUCGUCGUCCCUCGAGAACGGACAGAGGAGACA
GACC
>EMBL_CDS:BAF82320 BAF82320.1 Homo sapiens (human) hypothetical protein
AAAUUCGGUUCAAGGUGUUUCGUAAGAAAGUUAAGUCCCUACAGAAAAUGAAAGUCACCUUGAAC
UUAAUUU
>EMBL_CDS:BAF83693 BAF83693.1 Homo sapiens (human) hypothetical

protein
AGACCAACCCGGAACGGCCCGUGAGACCGAAGGCCUCCGAGACCUCCGGGUUCACGGACUCUUCC
AAGGUCU
>EMBL_CDS:BAF83474 BAF83474.1 Homo sapiens (human) hypothetical
protein
GGCGGUACAAGUGUCCCUGAACCCGGAACGACCCGGUAGUCUGUGAGCAGGUCCAGGACCACCGU
CCACC
>EMBL_CDS:BAF82652 BAF82652.1 Homo sapiens (human) hypothetical
protein
CCCAACAGACACUAUUUACCUUAGACCUCCUGAAGUGGAAGUAAAAGUGUAAAACGUUGUCUUGG
A
>EMBL_CDS:BAF82401 BAF82401.1 Homo sapiens (human) hypothetical
protein
ACAUCUCUUCGACUUCGGCCAUCACUUAAGGCAAUAACCAGAAAAGCGACAACCGAAGAAAAAGG
AAGAGGUGC
>EMBL_CDS:BAF82503 BAF82503.1 Homo sapiens (human) hypothetical
protein
CGGUAACGACCGAGAGAGAGGACCCAUCAGGUUCCUUAGGCACUCUGGUGACCCCACUCCCGGUA
GCCACCG
>EMBL_CDS:BAF82429 BAF82429.1 Homo sapiens (human) hypothetical
protein
UUAGACCGGUACAAGAAACUUCCUCUCAAACUACAUCCUCAAGAAUGAUGAGACCUUACCCGGUC
CAA
>EMBL_CDS:BAF82111 BAF82111.1 Homo sapiens (human) hypothetical
protein
CGCAGAUAGUAUAAACCAAAACUACUAAGUAGUCCUAAUAUUCACUCAACCUAACGAAAAUAGUC
GACAAACUGAAGACUACAAAACAAAAGUUGAACUACAUUCUACA
>EMBL_CDS:BAF83032 BAF83032.1 Homo sapiens (human) hypothetical
protein
AGAGGAAAGAGGCAACGGUCAAGGAGGUAGAGACCACAACCGAGUAGAAGGUCCUUCCCUAACCA
UCUACCCCCACCUCU
>EMBL_CDS:BAF83617 BAF83617.1 Homo sapiens (human) hypothetical
protein
CCUUCGACUCCAGAAGCACCACUUACCGGAGCAUUAGUAUGUCCCGUUGGUGCUACUACAGGAGU
CUCGG
>EMBL_CDS:BAF83614 BAF83614.1 Homo sapiens (human) hypothetical
protein
AGUGUUUAUGGUCCUCGUACAUCACCAAGGAGAACACCCUCGGCUACUUGUGACGGUAUAACGAC
AACCAUCCACU
>EMBL_CDS:BAF82421 BAF82421.1 Homo sapiens (human) hypothetical
protein
UCAGGUUUCUUGACAUAAGAUUCUCCAGUAAGAAUAUCUGUCAAACUUCUAUCAAGGGACAAUCC
AUCUCAAAGAAACCUGA
>EMBL_CDS:BAF82282 BAF82282.1 Homo sapiens (human) hypothetical

protein
CGGACCCCUACCGGCUCCCUUACCCGUCAACUGACCUUCUAAAUCGUCGACGAGGGACGCCGGCC
CCGUCCG
>EMBL_CDS:BAF82871 BAF82871.1 Homo sapiens (human) hypothetical protein
CCUGACGAAACGUACGACUCUCACGACGAAGAAGAGGUCCCGACAGUCGUACGGGGGGUUCAGG
>EMBL_CDS:BAF82241 BAF82241.1 Homo sapiens (human) hypothetical protein
GAACACCUGUAACGUACACCGGACCUCGACCCACCUUUGGGUCAGGUACAAGGCCAGUACUAGUU
ACACGUACCC
>EMBL_CDS:BAF83705 BAF83705.1 Homo sapiens (human) hypothetical protein
GCGUCGGUUCAGGAAUACGUGUUUCGCCAACAACCUGUCCACGAGGUACGGGUCCCGAUGGACCC
ACGUAUCCCGGUGGAAGACGAACCACGACUCCCCGACAC
>EMBL_CDS:BAF82092 BAF82092.1 Homo sapiens (human) hypothetical protein
AAGGUAUUUAGGACACGAGGACCUGUACCUCGCCUCCUACUUGAGCUGACGUAAGAGUUACUAGA
GACACGGGUCCUCCGAAUCCUUAAACCCCUU
>EMBL_CDS:BAF83622 BAF83622.1 Homo sapiens (human) hypothetical protein
CUCCUACUUAAAACGUCGCACCUAAGACGGAACCUUCUACACAAAAAGUAGAAACCUCCAUAUUA
GGAAGACGUUCAAAAGAAAUAAAAUCAGAAG
>EMBL_CDS:BAF82365 BAF82365.1 Homo sapiens (human) hypothetical protein
AGGAAUUUCUGACCCACCAACAGUAAACAUAGUCAUUGUGAAUGAACGUUUUAUCGUCAUGACCU
AGAAACUCCU
>EMBL_CDS:BAF83187 BAF83187.1 Homo sapiens (human) hypothetical protein
ACUACCAAAAACGACCUUGUGACCUCAAACAAACUCAGAACCCACGACGACCUUCGUUAUUUCCG
AACCCGACUUAACCUCCAAAACUUAAGUGGACUUAAAAAAAGAAACCUCAGUCAGGUAAAAGUCA
GAACAAGGAUCUUGGUCACUGUCCGAAUAGAAGGAAGUCACCGAGGCCACUUCUCUCUGAUAAU
>EMBL_CDS:BAF83448 BAF83448.1 Homo sapiens (human) hypothetical protein
UCUUGUCACGUCUCCCAAGGGGCUGUCCUGGUCAAGGACAUUCCUCUCCGGGACGUGUCAAAA
>EMBL_CDS:BAF82979 BAF82979.1 Homo sapiens (human) hypothetical protein
GUCUCUCAGCUACUUCUCUACUUACCUCGCGAGCACCCUCGUGAAGUGAUCGACCAGGUGUAGAG
GUUCAGGAGAGAGAC
>EMBL_CDS:BAF83152 BAF83152.1 Homo sapiens (human) hypothetical protein
AAGGUCCAGUCUAGACAUAGACAUGUCUCAAAGUUGAUCUCAUCUUACUCGGAGAACAUGUCCAA
AAAGUUACGCCUAUAAGACCUU
>EMBL_CDS:BAF83331 BAF83331.1 Homo sapiens (human) hypothetical protein

CGUUAAGACCCUCGACGGAAUAAAACAACAGUGCGUCCUGUUCGAGAGCUUCAACUCCGUCAAAA
GCCUUACG
>EMBL_CDS:BAF83608 BAF83608.1 Homo sapiens (human) hypothetical protein
CCCUAGGCACAUUAGGUCCCACGACAAAAGAAAAGAGAUAUGUCUGCCAUGAGGACCCAGAGGUC
CUAGGG
>EMBL_CDS:BAF82394 BAF82394.1 Homo sapiens (human) hypothetical protein
AAGACGUCGAAGGUCUACGAGGUCGACAGUCACCGCUGUGACAACUGCCAGAGCGAGUACUCCCG
CUACUCGUACAACUCGUCGUCCU
>EMBL_CDS:BAF82417 BAF82417.1 Homo sapiens (human) hypothetical protein
ACCACGCAAGUCUUGAUGACCGACUACACGAACGUAUGACCGGUCAUAGACGACUCGAGUGAU
>EMBL_CDS:BAF82583 BAF82583.1 Homo sapiens (human) hypothetical protein
CGGUCACUCUGUCUCCCCUGUCGCUCCCGACACCUCGGUCUAGAUGUCAGUUUAGGGUAAAGUGA
CCG
>EMBL_CDS:BAF82265 BAF82265.1 Homo sapiens (human) hypothetical protein
CACGACGAGUCGCGGCCGACGGGGGUCAGGGUGUCAAGAUACCCUUGGUUCAUCCCCCGUACCGC
CCCACCUCGUCCCG
>EMBL_CDS:BAF82223 BAF82223.1 Homo sapiens (human) hypothetical protein
CCUGGAGACACGGUAGGAAGAGCUACUCGCGGGCGUGUUUCCAGUCGCUGACGAGCUCCCGUACC
CACAGGGACCUCGUCCACAGGUCUUACGCCCGCGGGUCGCACUACAAUCCGUCGUCCAGG
>EMBL_CDS:BAF82500 BAF82500.1 Homo sapiens (human) hypothetical protein
AGGAGAAGUAGCCCAAGGGGUCAGGAGACGACGACCAACUUGAACGUGGCCGUAGAAGACGAGUC
GUGCUACUCCUACGGGUCCUACUUCUCCU
>EMBL_CDS:BAF83533 BAF83533.1 Homo sapiens (human) hypothetical protein
CAGAAGUCCUACUUCGACGUCUCGACCCCUUGGAGGUCGAGUUGGAACGUCAACUAGGACCACCG
>EMBL_CDS:BAF82409 BAF82409.1 Homo sapiens (human) hypothetical protein
CCGCGACCGACGUCUACUUCCUACAGGACCACCCUCCUACCCGGGACACCGGCUCCAAGAAGAAA
GAUCUCUAAGGACUGCGACGACGACCACGA
>EMBL_CDS:BAF82680 BAF82680.1 Homo sapiens (human) hypothetical protein
CCAGAACGUCGGUCAACUCCCUCGAGAGCCAGAACCACCCCGCUUCCGAAACCGAGGCGCUCUAC
CGGCCCAGUAAGGGAACCUUCGACGACUGA
>EMBL_CDS:BAF82658 BAF82658.1 Homo sapiens (human) hypothetical protein
UGACUGUCUGGCCAUCCAGACAUACCAGAGAUCGACCAGAUUUGUGGUCAGAUUAAGGAGGUCGC
AAAGGUAACGAUUAAAAACUAUUCUAAGAAGAACACCCAACUGAGACCUCUGUCCUCCGACUGAU

CGUCGACCCCUGGAGAACAAUCUACAUGGUACUUCCAAACAGUCA
>EMBL_CDS:BAF83039 BAF83039.1 Homo sapiens (human) hypothetical protein
CCGUAAGGAGGAACACCUCUCAGACAGACGCGCGACGACCAUGGCUUUCGUCAAAAAAACCACAC
GUGGAACGCCACGAGUUAAUCUGAAAGAAAUAAACCUUUAUCCUUCGG
>EMBL_CDS:BAF82999 BAF82999.1 Homo sapiens (human) hypothetical protein
GUCAGGUCUAGAAGUCCAAGAAGGACCCAUCCUGACUCUCGACUCAGGACCUUCCGAGGACCCUA
CCCUGAC
>EMBL_CDS:BAF82997 BAF82997.1 Homo sapiens (human) hypothetical protein
CUUCUAUGACAACAGGGUGGUUGAACUCCAUAAGGAGUUACCUAAACCACCGUUAUCACAAGAG
>EMBL_CDS:BAF82867 BAF82867.1 Homo sapiens (human) hypothetical protein
GACCUCCACCCCGGCCACUCCUGACGACCCGGAGAGCCAAAACCCUGUCCACGUCGGGUAACCGA
CAGUCGUCACGACGUAAGAGGAAAUCUAUUUGGUGGUCACCAUGGAGAAGGAGGAGUGACCGGUG
GAAGAGGUC
>EMBL_CDS:BAF82568 BAF82568.1 Homo sapiens (human) hypothetical protein
UCACGAAGUAGGUCUCCGAGUCAUUCCACCCGGGACCACUCGUAGACGGAGUCCGCGUGCGCCAG
CCGCCUACGAGACCGGUCCCGGAGGAAGAGGUACCGGAAGACGUCGUCGUGA
>EMBL_CDS:BAF82458 BAF82458.1 Homo sapiens (human) hypothetical protein
CCCCGCCCCUCAGGACUCGGUACGGAGGGUACUACUCGUCCUCGUCGUCUCCGUCCCUUGGGGAC
GGGG
>EMBL_CDS:BAF83157 BAF83157.1 Homo sapiens (human) hypothetical protein
CCUGAGAGACCGAGACGUAGUGGAAAGAAACCUAAUAACACAGGUCCCCUAUGUUCGACUCUCAG
G
>EMBL_CDS:BAF82714 BAF82714.1 Homo sapiens (human) hypothetical protein
UAGACCUUUGAGACACAUCUACCUCCCGCACUCCACCGUGUCCUCGGCGUGUAGCAGGAGGACCG
UCCGGAAGUACAGGUAGUAGUCCGUCCACACGUCUA
>EMBL_CDS:BAF82285 BAF82285.1 Homo sapiens (human) hypothetical protein
CCGACUUCCGGGGUCCAACUACAAGGGUAACCGGGACCUCCCGACACCUGAACCCCGGGGUCGA
>EMBL_CDS:BAF83711 BAF83711.1 Homo sapiens (human) hypothetical protein
CCGUCGAUCCCCUCUUCGUCGAGGUUUUAUAGGUAACCAACGUCGUCAUCGUCGUCGGUCCGAUG
GAUUACGUUUACAGGGGCGGCAUGGUGGUCGACACGACGGUGACCGACUCCGAAGUGACCGUAUC
CACCGGUUAUUUAACCGACGACGACGACGACCCAACCGUGACCGUCGACGACGUCGGUUUCGGGG
ACAACGACGA
>EMBL_CDS:BAF82494 BAF82494.1 Homo sapiens (human) hypothetical protein

CGAAAGGAGAACGGGGCCAAGAACGAUCAGUACAAAAAGGACCUGACUAGUAGUCAUAGAAAUGA
ACCACAUCAGACCCUGUUUCUACCUGAACAUCUUCCCAGAGGUCUCCUUUCA
>EMBL_CDS:BAF83528 BAF83528.1 Homo sapiens (human) hypothetical protein
AACUUCCUUAUUUUGCACAGUCUCGGUUGGCAGAAUCACCGAAGUCAAUACGCCAUGUUAUGUUU
GACCAACAAUGAAACCAAAACUGACAAUAUAAAGAAGUC
>EMBL_CDS:BAF83123 BAF83123.1 Homo sapiens (human) hypothetical protein
CGACCUCUAGACUCUACUCGCACGACCGUAGGACGGAGACGACCUGGUCGACCAGACAUCGACGA
CGGUCCAACAGGUCGUGUAAGUGGAGUGGAAGGAGGUCG
>EMBL_CDS:BAF82992 BAF82992.1 Homo sapiens (human) hypothetical protein
AGUAACACCAAAGGUACUUGAAGAAGACCUUAAGGACUAAAAGUAGGAACUUCCUCACGAGGUAC
UUCUAACGUUACC
>EMBL_CDS:BAF83005 BAF83005.1 Homo sapiens (human) hypothetical protein
CUACCGAGAAAGUAGUUAACAUAGACCAAGUCUAUUCAACUAGUAAAACGUUUCUUCCUACGGUA
G
>EMBL_CDS:BAF83714 BAF83714.1 Homo sapiens (human) hypothetical protein
CCUAGAACUAAAAAUUUUUGAACUGAGAACCGAAAACUCUCAGGUCAACGGUUCAAUGAAACAAA
AAAGACUUCUAGG
>EMBL_CDS:BAF83249 BAF83249.1 Homo sapiens (human) hypothetical protein
ACAGGUAAAAGGUAGAAGUGUUGCAUAAAGACGACUGGGUACCUAGAGUUUUAAUUCAAGUGUAA
GAUAUACUUGAUAAAGAGGUACUGCUAUUCAACAACUUACACAAACUUAAAACCUCAAAUACCCG
UCUCCACCUCCUCCUGU
>EMBL_CDS:BAF82907 BAF82907.1 Homo sapiens (human) hypothetical protein
GACGAGGGUCUUCGUGGGGGUCUCGAAGACGGGUCAAGAGACCCCCGACUACGACCAAGACCCUG
UC
>EMBL_CDS:BAF83287 BAF83287.1 Homo sapiens (human) hypothetical protein
GUAGGACCCCAAACAGAGUCAAAAAGUCCGACGAAAAGACUAUGACGUCAGUAACUUUUAUGGAG
UCCCAC
>EMBL_CDS:BAF82366 BAF82366.1 Homo sapiens (human) hypothetical protein
AUUCUUUUGGAAAAACUAAAAGAAAUAGUCUAGGACUUUGGUCACUAAACAUCUCUGACCUUCAU
CCCAACUAAAAU
>EMBL_CDS:BAF83418 BAF83418.1 Homo sapiens (human) hypothetical protein
CACCACCGCCACCGACGACCCCACCACCGACACCACCGCCUCCGACGACACCACCGCCGACAACG
ACCCCUCGGGCCAACAUCGACGGAGACAACAACCUCAACAACAAUAGGGCCAACAACAAUGGGGU
CGCCAAGACCGAUAUCGGUGGUG

>EMBL_CDS:BAF83738 BAF83738.1 Homo sapiens (human) hypothetical protein
UCGUCUCCGUUCCGACCGGUGGAGUAGUAGUACCAGUGAAACGAGUCGUGACCGUAGGACCGACA
GUGCAACGACGUCGUCCACUAUACCACCAGCCACCUUCCGGUCCGAGAACACGA
>EMBL_CDS:BAF83681 BAF83681.1 Homo sapiens (human) hypothetical protein
CAGACCCCAACAGUCACACGAGACGGAAAAAGAAUCCCGUCUCGUGAAAUGACGGAAACUGUCUG
>EMBL_CDS:BAF83419 BAF83419.1 Homo sapiens (human) hypothetical protein
UACGAAAGUCACACAGGCAACGAGGUCGACACCGAUCCUCAGACCCACUGUCAUCACCUCACUAC
CCGACACCUUCAUA
>EMBL_CDS:BAG56880 BAG56880.1 Homo sapiens (human) hypothetical protein
CUAGGUCUACCGACCUGCGAAUGACGGGGGGCACGACUUGAAGUCGUGCAUUUACCCUGCGGGAA
GUAUCCACAACGGUUAGACCUAG
>EMBL_CDS:BAG58264 BAG58264.1 Homo sapiens (human) hypothetical protein
UGACAACCACACCGACUUCGUGAAAUUUCCAAGGACGUCGACGCCGACGUCGACGUCGACGUCGU
CGACGUCGCACGACGACGUUUUGUCA
>EMBL_CDS:BAF84207 BAF84207.1 Homo sapiens (human) hypothetical protein
GGUCCUCACAACCACGUACAACUUAACUACCCCGUGUAAAGUCACGUAAGUAGUUAUAGAAGUGU
GCUACGAAACUCUGGGACC
>EMBL_CDS:BAG58070 BAG58070.1 Homo sapiens (human) hypothetical protein
UCCUUCGAUUUGGGGUAGUAAUCUAUACUCCCUCCUUCUUUUAAGAAAAAAGGAGAAUAGUACUU
CGAAGGA
>EMBL_CDS:BAG58030 BAG58030.1 Homo sapiens (human) hypothetical protein
CAAGGUCUAGUAGGUGACUCUAUUUGUGUAGCAACGACUACCACCUCAGAGCCACCUACUCCGCG
ACGUG
>EMBL_CDS:BAG58082 BAG58082.1 Homo sapiens (human) hypothetical protein
UUGGAAGGACGGGUACUCUAAGAAGACCUUCUCCGAGAAAGAACGAGACGAGAAAGAAAAGGUCU
UUCAGAGUAGGAACCGACCUACCCAA
>EMBL_CDS:BAF85421 BAF85421.1 Homo sapiens (human) hypothetical protein
UACAAGGGGGAGAGAUCGAGACAGACGUCCGUACACAGGACAGGGUCGUUCCCCCGGUGGUACAG
UAACCACUGUCCGUCGAACGACCCGUUCGAUCUCUACGUCACCUCGUA
>EMBL_CDS:BAF84376 BAF84376.1 Homo sapiens (human) hypothetical protein
ACCAAGAUUGUGUAAUUAAAGAUAAAAUGAGAACACUUAAAGUGAUCUUAGUCUACUGAACCUUG
GU
>EMBL_CDS:BAG57257 BAG57257.1 Homo sapiens (human) hypothetical

protein
CCACCACCCAACAGCACCAGUAUCACCAUGUCCACCAUGAGUUAUGGUGACAUUGAGAACAUCUG
GAAAGCCGUCCAGAGGUGG
>EMBL_CDS:BAF84679 BAF84679.1 Homo sapiens (human) hypothetical protein
GUAUAAAGGACAUAAACACACUUACUUCUUACCUCCUUUAAGAAGGGUAUGAAUAAUCUUUAUAC
>EMBL_CDS:BAF85202 BAF85202.1 Homo sapiens (human) hypothetical protein
CCGCAAGACAAACGUACUCGGGUCGAACAGGUCCGACCCAAAGAAACAAUUAGCUCUUACGA
>EMBL_CDS:BAG57591 BAG57591.1 Homo sapiens (human) hypothetical protein
AGACGAAGAGGGUACAACAUUGGGUACCUCUAGUCCGUCCGCGCCUUUAGUAGGCACAGGUAGUA
CGGACAGAAGAAGGCCAGUUUCACCAACUUCCUCC
>EMBL_CDS:BAF84216 BAF84216.1 Homo sapiens (human) hypothetical protein
GUCUAGGGCGUUUUUCCGUGGGUGCAGAUGUCGAGCCGCCCGACCUCCAGACGCCUGGUUCGGUC
CGGAGACGUGACUGACACCGACUUCUCCACACCGUUUCCAGUCCAACUCCCUAGAC
>EMBL_CDS:BAG56677 BAG56677.1 Homo sapiens (human) hypothetical protein
CCUUGCAAAACAAACCCCCAGAGAGUAAUGGUGUGUUAGACACUCACACGGGGUAAAGAGUUUUA
CAAGA
>EMBL_CDS:BAG57781 BAG57781.1 Homo sapiens (human) hypothetical protein
ACCGUCCGACUCCGUCCUCUUAACGAACUUCGGUCCUCCGCCUUCAACGUCACUCGUCUCUUACG
UGGUGACAUGAGGUCGGACCCGUUGUCUCGCUCUAAGGUAAAGAAGUCGUCGGU
>EMBL_CDS:BAF85170 BAF85170.1 Homo sapiens (human) hypothetical protein
CUCCUCAGAAACUUCAUCUUAGACUUUUAGAAUUUGAUAGAAGUCAUAAAGAACAUCUAAGGAG
>EMBL_CDS:BAF84864 BAF84864.1 Homo sapiens (human) hypothetical protein
ACGAAAGUCCAUACUCAGGUCCCAUGACAGGUUGACCUACGACGGGACCACCGACUUCCGU
>EMBL_CDS:BAF85240 BAF85240.1 Homo sapiens (human) hypothetical protein
CCGGAACCUAAGCGACGAGUACGUCAACGACCUCGUCUACCGUCGACACCCAUCGUUGUCCCAG
>EMBL_CDS:BAF84836 BAF84836.1 Homo sapiens (human) hypothetical protein
GUUACCAUAGACAAUAAGACCGAAAAUAAACCGAAAAGGAUCGUCGACGAAGUGAUCGUCAGUAG
CCAAGUCCUUCUCGACUUCCUUAUCUUCUUAAUAAC
>EMBL_CDS:BAF84347 BAF84347.1 Homo sapiens (human) hypothetical protein
GGCCGAGUCCGCGUCUCGGUCGACGUCGACGUCCAGGAGCAGGGACACCCUCCGACCCCCCUGUC
CGAAGAGACGGAGGAGUGCCGAGUACCGCUCCCGGUCGACGUCGACGUCAAGGAGAAGGGGACUG
CAGACUCCGGCC
>EMBL_CDS:BAG57215 BAG57215.1 Homo sapiens (human) hypothetical

protein
GGACCAUUUAUAGGACCCGUCCCGGAGGUCCGGGACCCCCGUGGACCAUGUCCAGACACCGGCCC
>EMBL_CDS:BAG57077 BAG57077.1 Homo sapiens (human) hypothetical protein
CCGACGCAUCGGAGGUAUUUAGAGUCGUGGUAUUUUAGGUAGGACUAUGUGCUGGCGAGGCAUUC
AGCCCUACCCCCCACCCCCACCGCGUCGG
>EMBL_CDS:BAF85419 BAF85419.1 Homo sapiens (human) hypothetical protein
AACUAAAACAUUUGGAGGUAACAGAAGUAAGAACUCAUCCCACCCUAACCGUCAAAGGGAAACCC
CUGACUUCUCUAGUUGACUUGACGUAACGGUUCUUACCAACCACUAUUUAACCAGUACCAAAAAC
GUUCUAGUU
>EMBL_CDS:BAG58600 BAG58600.1 Homo sapiens (human) hypothetical protein
CAGCGUCUCGGCACGUAUCUGACGACCCCAGUCCACUGACCGGUACGUCAACAACACGAAAGCCU
CCAGACCCAUG
>EMBL_CDS:BAG58480 BAG58480.1 Homo sapiens (human) hypothetical protein
GACACCGACCACACCGAGAGGGUAGUCACCGACCUCAUCGCGAGGACAGUCACCGACCCCACUGU
GAGGGUAGUCACCGACCUCACUACGAAGGUAGCCACCGACCCCACUGUGACGGUAGUC
>EMBL_CDS:BAG57966 BAG57966.1 Homo sapiens (human) hypothetical protein
CGACGAACUCCCGGUCCUCUGUCCGGUACGUAGCCAUUGCCUUCGUACCAAAGAGUCCGACCUCG
AGUCGUCG
>EMBL_CDS:BAG57341 BAG57341.1 Homo sapiens (human) hypothetical protein
UGGUAACAGGACGUCGAGACCGGUCUCGAAGUCGGCCACCGUGCGACGGACGUCCGCUGAGUCAC
CUGCCUCUACGUCCUGUACCA
>EMBL_CDS:BAG57707 BAG57707.1 Homo sapiens (human) hypothetical protein
UAGGAGGACAUCCUUCGACGACCGGACAUCUCCGCUAAUGUAUCGACCCGACGACCCGGCCGACA
CCGUAUUCCCUCCUA
>EMBL_CDS:BAF85245 BAF85245.1 Homo sapiens (human) hypothetical protein
CUCCGAAUUUCAAGUACAGAAACUAAGACUAUUAACCUAAAAGGGGUUAGUAGUCUUAGAGAUCU
AAACCUUCUCGUAG
>EMBL_CDS:BAG56840 BAG56840.1 Homo sapiens (human) hypothetical protein
CCUCGGGCAGAAAGGGUGGACACCCCCGGGACAGUCGUCCUCGGCAGCCCCCACCUUCAAAGCCC
ACGG
>EMBL_CDS:BAF85698 BAF85698.1 Homo sapiens (human) hypothetical protein
CACUUCAAAACAACACUAAUAAACACAGUGUACUUAAAUCCACCCUGAAUGUUUAUAGGUAUUAU
ACUUUUGUCCUGAAAUG
>EMBL_CDS:BAF85766 BAF85766.1 Homo sapiens (human) hypothetical

protein
AGAAGUCUCACCGUAGGCGAAGUACGUCAGAGAGGACUUCUCAGACGUCUCGAACUAAGACGUCC
GACAAUGGAAGUGAAACUAGGACGUAAUAAGCACUAAAAAGUGAGACAUUCC
>EMBL_CDS:BAF85338 BAF85338.1 Homo sapiens (human) hypothetical protein
UGAUAUGUCCCGGACGACUCCAACACUUAUAACGACAACAUUACGUCGACGACGUCGACGCCGAA
CAACGACGUUACGUCGUCAAACACAAUUGACUAUCAAACGACCUCAAACACACCA
>EMBL_CDS:BAG58200 BAG58200.1 Homo sapiens (human) hypothetical protein
GUGGGAGGACGGACCGUCCUCUCGAGUGACCCGGGUGUCCCCAAGAGGCACCGUAACUUCCGGUC
CAUCCACCGAC
>EMBL_CDS:BAF84446 BAF84446.1 Homo sapiens (human) hypothetical protein
CCGGAGGGUAAACUCAUCCUAGUCGGUAAGGUUUCAAUUCGACUCCGUAAAGAAGUCAGAAACGA
AAGAACCCCGAACCGUUCAGAGAACUUCUCAGUCUACCCUUCCGG
>EMBL_CDS:BAG57305 BAG57305.1 Homo sapiens (human) hypothetical protein
AAGACGUCGAAUCUACCAUCAAAACAUCAUUAUAGUAGAUCAGGUAGAUUCGUAAGGUGUCUU
>EMBL_CDS:BAG57209 BAG57209.1 Homo sapiens (human) hypothetical protein
CCUCGGCAGGUCAAGGGGUCCCUUACCCCCACCAUACCUCGGGCGUCCCCGUCUGGCGAGGGUUA
GGAACCUCCCGAGG
>EMBL_CDS:BAF84645 BAF84645.1 Homo sapiens (human) hypothetical protein
AAGGACACCUCGACUCUGACAAAAGUGGAAAUCAGGGGACCUCCCAAAACCAAAGGUCUAUAUUC
ACUAGUCCUGGAAAAUUCUGUUCCGCUUAUACAGAUGAAGGUAUCCCU
>EMBL_CDS:BAF85023 BAF85023.1 Homo sapiens (human) hypothetical protein
AGACAAGGGACCGUUAUGGAGGGCUUCACCGACCAUCCCGACCGGUGACCUCAAACGAUACCGAC
GUCC
>EMBL_CDS:BAF84376 BAF84376.1 Homo sapiens (human) hypothetical protein
CAAAGAAGACUUCUAUCCGUAAAUUGAUCAAGACGUAAAAAUCAUCCACUAAAUCAUCCUCUUCU
GUUCUAAUAUCAACCGUCCAAAAGAGGUAAUUAUGAUCUUUGUCGUGACCAUUCAAGUCAUAGAC
UCCUUUG
>EMBL_CDS:BAF84474 BAF84474.1 Homo sapiens (human) hypothetical protein
CGUCUCUCAACCGUCCACGGAGACGGGGUGACCUCGUCUACUAUCCGAGCCGGACCAGUGAGAGA
CG
>EMBL_CDS:BAG56859 BAG56859.1 Homo sapiens (human) hypothetical protein
UCGACGAUCUCGACCUCGACGCCCUCCACGACCUCGAAGGACUCGCCGACCUACGACUCCUUCUC
GACCUCGGUGACGAACUCGGUCACGACCACGA
>EMBL_CDS:BAG58495 BAG58495.1 Homo sapiens (human) hypothetical

protein
GAGUCCCACCAGGAGAGUGUGGUCAAGUGAGGAGAACCGACCCACUCCAACACCUUCUCCUGGUA
UAGGAGACCC
>EMBL_CDS:BAG58417 BAG58417.1 Homo sapiens (human) hypothetical protein
GGACCGGACGGGAGGAGGAGUUACAGGUCGACGAGACCGAACUCCGACAGUCUUCGGCUUCAGCA
CCGGUUCCUUUCGUCGGGACCUCACCCUCCAUGACCGUCCGGUACC
>EMBL_CDS:BAF83866 BAF83866.1 Homo sapiens (human) hypothetical protein
ACCGUCCUUCUACCGUGACCGGUCCAACGGCCACUGUGGUGAGUCGUCCAUCUACUACCGAUACG
CCACGGUCGGACGGU
>EMBL_CDS:BAG57315 BAG57315.1 Homo sapiens (human) hypothetical protein
CAGAGCUGUACCGAUAAAAGUUCUUUCGGACUAAAUUAUUUCAUAUUCCACCACAACUUGGUACA
CUCCG
>EMBL_CDS:BAF84089 BAF84089.1 Homo sapiens (human) hypothetical protein
CCAUCACGAAAAGGCACACCUACUUGACCAUCGGGCAGUGUAUCCGACGGUCCUACGGUGGGUCG
UGACGG
>EMBL_CDS:BAG56931 BAG56931.1 Homo sapiens (human) hypothetical protein
CGAACAACCUCCCACUCAGGAACGGGUCGAACACGAGUCUCCACCUCGUCCUCAGGUACUUCCAG
ACCCGGUGGUGGCUGUUCG
>EMBL_CDS:BAF84102 BAF84102.1 Homo sapiens (human) hypothetical protein
CUUAAGAGACUACAACUUAUAUCGAACCGAAAAACGAUUUCCAUAAGGACACAAAGAAUUGAAGU
CUCGUAAA
>EMBL_CDS:BAG58077 BAG58077.1 Homo sapiens (human) hypothetical protein
GGUACACUUCUGUCUAUCUCGUCAGGUCCAGGUAUGACAGACGAAACAUGAAAAAACAUACC
>EMBL_CDS:BAF84735 BAF84735.1 Homo sapiens (human) hypothetical protein
CCUCCCGCUCCACCCCCUACACUCCGACUCGAAAGUAUAGAGGCGUAGUACAUUAUAACGAGAAG
>EMBL_CDS:BAG56779 BAG56779.1 Homo sapiens (human) hypothetical protein
CUGUCGUCGAGCGACCACCACGACUCCGACAGAAGUAACUCCGACCUCCCACUCCUGCCGUCUGA
GUCGACUCGGUCGUCGUCGUACAG
>EMBL_CDS:BAF85554 BAF85554.1 Homo sapiens (human) hypothetical protein
CAGGGGAUGUCUACCCACAAAUCUAUCACGGAAGUUUUAUAGGACACAUCGACCGUCAUAGACUC
CUACACAGUACUCCCCUG
>EMBL_CDS:BAF83895 BAF83895.1 Homo sapiens (human) hypothetical protein
GACCCGACUUUAGGUCAGGGGGAUGAAACCGACCCCCGCACUCGGAGGUCCUAAGUCCCCAAGAC

CCCUCCUGUCUAAACGAGCC
>EMBL_CDS:BAG57690 BAG57690.1 Homo sapiens (human) hypothetical protein
CGACCCGGGGGGUGGUUCGGAGACGAACCCCAGGAAGAAUUCGUCGGGUCGUUCGUCCCUGAACC
GGAGCCCCGACUCG
>EMBL_CDS:BAF85101 BAF85101.1 Homo sapiens (human) hypothetical protein
CCCUGAACACACAGAGGACGACACGUAGGUAUGACCCACGAAAUCUUUGCCGUCCGUCUGGUCUU
CGGG
>EMBL_CDS:BAF84857 BAF84857.1 Homo sapiens (human) hypothetical protein
CCCCUCAAAAAGAGUCUCCCUAAUCGACCAAGUGUCAACAUAUACAAUUAUAUACCACACACUCG
AGAUUAUUUUAAGUCUGAGAGG
>EMBL_CDS:BAF84433 BAF84433.1 Homo sapiens (human) hypothetical protein
CGAAAUCGGACGACACCGACGCAGUCCUCGAAGGAGUCCUGCAAGGACACCGGCACCCGUCGACU
CACCGACUUCG
>EMBL_CDS:BAF85661 BAF85661.1 Homo sapiens (human) hypothetical protein
UCGGUGUGGUUGUCUCUACCCGACUACGGACGGGACCUAUGGUGUCCACAAACGGGUAAGAUUCC
UCCACCGA
>EMBL_CDS:BAF85008 BAF85008.1 Homo sapiens (human) hypothetical protein
CCGUCUUUUAGCUAAGUCUACCUCGUGCUCCUAAGCUCCCUUAGAAACAUGACAGUUGAAAAACC
AUGAAGCUCAAAAGACGG
>EMBL_CDS:BAG58141 BAG58141.1 Homo sapiens (human) hypothetical protein
CCUCCUGGAUUUUGUCUUCCUAAAGGGUGUAAGCGACGUAAGUAUCCCGAAGAGUGGACACACUC
AAGAGACCACGACCUAGUCACCCCGUUAAACCAACUUCCAGGAGG
>EMBL_CDS:BAG58003 BAG58003.1 Homo sapiens (human) hypothetical protein
CCCCGACAUCGUGCUCCGCGACAUCGUGCUCGACGACAUCGUCCUGCACGACAUCGUGCUCCUCG
ACAUCGUGCUCCCCGACGUCGUCCUGCGCAGUGUCGGGAGCGUUGACACCAGGG
>EMBL_CDS:BAG57805 BAG57805.1 Homo sapiens (human) hypothetical protein
AGCAAUCACACCCAAAGGAGGUGUUCGCAGGUGUCGAACGACUCCUUUCGGCCGUGCAAGCU
>EMBL_CDS:BAG57303 BAG57303.1 Homo sapiens (human) hypothetical protein
CUCCUUCGGUACGGACCGUACCAGGUACCAUGAACGACACCGGUCCAUGAACCUAGGCACGGAGA
CCCUCCCAGAGAAG
>EMBL_CDS:BAF85323 BAF85323.1 Homo sapiens (human) hypothetical protein
CGCGUGGCGACGGAAGACCCCCUCCGAGACCUACAGAGAUUUCUCCAGGACCCGGAGUCGGAGCU
UCUCGGCCGCCAACAGGCACA

>EMBL_CDS:BAG57928 BAG57928.1 Homo sapiens (human) hypothetical protein
GUACUCGCAGGAGGUCUCGACCUACCGUAGGAACACCCGUUCCUACAUCCGAGCCUAACCGGGCG
AAGUAC
>EMBL_CDS:BAG57970 BAG57970.1 Homo sapiens (human) hypothetical protein
UCUCAACCGUCAAGAAACCGGAAAAGAAACAGUGAUCGAAGAGAUCGGUGACAUCUCGGUCAACC
GAACCCUUUUCACUUUCACCGAAAAUCUAACCGGUAGAAA
>EMBL_CDS:BAF85130 BAF85130.1 Homo sapiens (human) hypothetical protein
ACCGUACAAAGUCUUCUUACCAAAACAUGACGAUAAAGAACCAUGAGUCUUUUUAAGAGGACUAU
AUACAAU
>EMBL_CDS:BAF84054 BAF84054.1 Homo sapiens (human) hypothetical protein
AACCACUGGACGUGCAAGUCCAUUAAUAACAGCUACUCGCCGGUCCGCGGGACGUGGAACGUCCA
GACCUUCGCCAGCCACUUUCAGGACUCCACACCCAGGGGCU
>EMBL_CDS:BAG57942 BAG57942.1 Homo sapiens (human) hypothetical protein
CCUUCUCGUGUGCCUACCCCCGACCGACAUGGGACUACCGCGGGAUCCUCCUGGGAGUACAUCUC
CCUCGCCCCCCAUGCGGUCAGGAGGUUAUGACUAGACGAGAGA
>EMBL_CDS:BAG57811 BAG57811.1 Homo sapiens (human) hypothetical protein
UCUUCUGCUGACACUCCGGUGUGAUGAAACACCGUCCUUCAGUCGAGACGAUCGACUCGUAGAAC
GACGUCCCUGACCCAGAUUGUCGAAUAGUAGA
>EMBL_CDS:BAG58536 BAG58536.1 Homo sapiens (human) hypothetical protein
UCGAGGAAUACAAAUCACUCCUGACACGAUACUUAUUUCCUGAAUGGUGUUAGGAACGUAAGUAU
CUUACCAUAUCGAGGAAACUCCCCGA
>EMBL_CDS:BAG57899 BAG57899.1 Homo sapiens (human) hypothetical protein
ACCAGACCAUCAACAUCACGACGAAGACGACUGAGUCCUAGACGUCAACGUCCUUGUCGACGACG
ACGACCUCGU
>EMBL_CDS:BAG57602 BAG57602.1 Homo sapiens (human) hypothetical protein
CGACUUCCAUAUCGUCAGGCCCUAGAGUAAUAACUACUCCGAGACCUCCAAUUACCAGAAGUCG
>EMBL_CDS:BAF85257 BAF85257.1 Homo sapiens (human) hypothetical protein
AUAAUCCAGAAGACCCAAAAACGGAAAGUCGACGAAAAAUCGGUCCCGUCGGGUAAGACCCACUU
CCCCGACAACGACCACAUCUUCAGCAACGACAUACUCGGACCUCGGACUAC
>EMBL_CDS:BAG58444 BAG58444.1 Homo sapiens (human) hypothetical protein
AGGACCAUGACGACCAAUAAGAGUCUCUCGUUUCGGACGACUCCACCUCCCACCGACUGUCACGG
UCCU
>EMBL_CDS:BAG58021 BAG58021.1 Homo sapiens (human) hypothetical

protein
GGACCCUCCGUUCUCGUCCGGGACGUCUCCGACAAGAGUUCAGUUUCGACCCGGACAAGUACGGU
GGCCC
>EMBL_CDS:BAF85237 BAF85237.1 Homo sapiens (human) hypothetical protein
CUCGAUCCCUACGGUGAACGCAUUCGCGGACAGUUCCUCGAGUACCUUCUCGAAGCCCUAGAGAA
UAAGUUCACCAGUAGACUCGAG
>EMBL_CDS:BAF84119 BAF84119.1 Homo sapiens (human) hypothetical protein
AAGACCAAAAACUUUCUCAAUUGUCUCACCAGAAAAAAGUGAUGAGAGAAAAAGAGAACAUGGUU
UCCGACCCU
>EMBL_CDS:BAG58492 BAG58492.1 Homo sapiens (human) hypothetical protein
GUUUACGGACAACUACUCCCCAACAACAUCACUGAGGUCCUGAUCUGGGGUCGAGUCGGCCGUAA
AC
>EMBL_CDS:BAF85447 BAF85447.1 Homo sapiens (human) hypothetical protein
GUCGGUCAGGAGUAACUAGAACCCGAACCCCAGUGACAUCACGUAGCUGUGGAAGACCGGGUCCU
ACAACUCCCUCACGAC
>EMBL_CDS:BAF85025 BAF85025.1 Homo sapiens (human) hypothetical protein
UCCUUUGUCGUUUCCUGAAAGAAAUUCCACCGACGAUAAAACUUCUUUCAGUAUGGUGACCAAAG
GA
>EMBL_CDS:BAG57874 BAG57874.1 Homo sapiens (human) hypothetical protein
GUCAAGAAAUUACAGAUGUAGUCGUCUCUCAUUGACUACGGGAGAGUAAGAACGACACACCUACA
CUUCUUAGAC
>EMBL_CDS:BAG58442 BAG58442.1 Homo sapiens (human) hypothetical protein
AGGAUCGUGUGGCAACCCCAUCACGAGACGGAGGUAGAGGUAGAGUGACAGCGACACCCCUCCCC
AACUCCACCCUCAACCGGGUCCCCCACACCAACCU
>EMBL_CDS:BAF85424 BAF85424.1 Homo sapiens (human) hypothetical protein
CUUCUUGUCUUUUUCCUCUGUUUCUUCCCGCAACACUAUAAAACGAGAAGCUUUACAGAGUCGUC
UACGAGAACGAAAAAGAACGAAACUCGAUAAGAAA
>EMBL_CDS:BAF84536 BAF84536.1 Homo sapiens (human) hypothetical protein
GUUUUCUGUACCGUUAAAUAAACUUAGAACGACAUAACAGAACCAUCUCAUUCAAAAUGUAAAAA
CACGGUUUUAAAGAAAAC
>EMBL_CDS:BAG57445 BAG57445.1 Homo sapiens (human) hypothetical protein
CAUAUAAUGAGGUCACAGGAGUGGUCGUCAAGACAUUAACGUCCCCACGUACCCUCUCCUCGACC
CUAUCCCUG
>EMBL_CDS:BAG57264 BAG57264.1 Homo sapiens (human) hypothetical

protein
CACCCGGGCCAGUCACGCGUCAUGUAGGAACUCCUGGCACGACGCCUACUUAAGGUCCAGGAGUC
CCAAGCGAGCGACCCGGGUG
>EMBL_CDS:BAF85124 BAF85124.1 Homo sapiens (human) hypothetical protein
CCGAAGCCCUAUCCCCACCCCUCCCCCCUCGACGCAUCGGUUGAAGAACUUUCACGAGACCGGAG
UCGACGGAGACAGGACAUCGG
>EMBL_CDS:BAF83938 BAF83938.1 Homo sapiens (human) hypothetical protein
CCGACCUGUUGCUACAGUCCUCCCAAAAACUGUAGUGUCUAUAUCCACGGUAACACAUAUUUGAC
GUAUAAAAGAUACAACUAACUACGUAGAAAGAACAGUUCCAGAGGUCGG
>EMBL_CDS:BAF83918 BAF83918.1 Homo sapiens (human) hypothetical protein
GCGGACGCCUCCAUCGUCGCAACCGCCAGGGAGUCCGAGAUCGAACUGGCGGUGGAGGCAGACAA
GGAGGCGGUCGC
>EMBL_CDS:BAF85253 BAF85253.1 Homo sapiens (human) hypothetical protein
AGGUCCCAAAAUCUCCACUCGUCACACCCGAACAUCGACUUCUACAGUUAGAGUCACGGGACCU
>EMBL_CDS:BAF84759 BAF84759.1 Homo sapiens (human) hypothetical protein
ACGGUUUUCACAGUUGGAAAAGACCAAUCCUUGGUGUUUUGACUAGUGUAUCCUGACUAGUGAAA
ACCGU
>EMBL_CDS:BAG57486 BAG57486.1 Homo sapiens (human) hypothetical protein
UCGACACCAAUCUCGGUCCGCUCCUCACUAUUAAGCGAGUUACCAACCCAGUAAUCGGUUCAA
>EMBL_CDS:BAF85387 BAF85387.1 Homo sapiens (human) hypothetical protein
GUUUCCUCCCGUUCUUGAGCCGGUGAAAAGACCCCGAACUAAAAGGACUUCGUUACGAGGUCCCU
CCAUGUCGAACUCAAGACCUAGAAGGUAAC
>EMBL_CDS:BAF83900 BAF83900.1 Homo sapiens (human) hypothetical protein
ACCGUGACCCUGAAGACCAGGACGUCGUGCCACCACUACACCCUGGUCAACACGACCCGGU
>EMBL_CDS:BAF84318 BAF84318.1 Homo sapiens (human) hypothetical protein
CACCGGGUCGAGUACACGGUCCCAGUGCCGACGUGGUUCCCCUAACAGACUUACCAGGUACUGUU
AAGGGGGUCUGACCAGACGGCACGUGUACGAGUACUAACCCCGGUA
>EMBL_CDS:BAG58534 BAG58534.1 Homo sapiens (human) hypothetical protein
CGGGGGAGGAGUAGGUUCCCGGGUCUCGACCCCCAGAGUCCGGACGCCUUCCGUUGAACCUACCC
UUCCCCG
>EMBL_CDS:BAG57202 BAG57202.1 Homo sapiens (human) hypothetical protein
UUCCAGGAAAACGUCACACUAGAGCGACUACUAAUGACUUCGACUGUAUAGACGAUUUCCUAAAA
>EMBL_CDS:BAG60413 BAG60413.1 Homo sapiens (human) hypothetical

protein
CGAGAAUGUAGAUAGUCGGUCAUCAGGACGACGACAGACGGUAGAGACUACCUACACUACCCG
>EMBL_CDS:BAG61320 BAG61320.1 Homo sapiens (human) hypothetical protein
ACUAUGUCAAUAAACACGACAACCGACUCCUCAUCUGUCCGAUUCCGAACAUCCGUCACGUUGUC
GACCGACACAAU
>EMBL_CDS:BAG61920 BAG61920.1 Homo sapiens (human) hypothetical protein
GGCUCUCCUAGGUCCGUGUACCAUAGGUCCGAACGUAAACAUAAACGUCUUCCUCGGGUGGAACA
CGAAUAAGAGAGCC
>EMBL_CDS:BAG61669 BAG61669.1 Homo sapiens (human) hypothetical protein
AACGGGUGGUAGUGACAAGGGAAUCCUUAGACUUAUCACUAAAACCCUUACCCACCACACCGUU
>EMBL_CDS:BAG59189 BAG59189.1 Homo sapiens (human) hypothetical protein
GAGGGUGUCGGGAGGAGUGUCUGACUUCUCACCUCCAAGGGAGAAACCCAGAAGGUUUUAGUGUC
GUAUUACCACUCCUCUCCCCACCCUC
>EMBL_CDS:BAG59510 BAG59510.1 Homo sapiens (human) hypothetical protein
UAACUUUUGCAAACGGGACAAGUUGAAGAUCUUUCCAAUUAAGACUGUUUUAUCCGAAAAGACCU
UGUACCCAUUAAAAGUUA
>EMBL_CDS:BAG63066 BAG63066.1 Homo sapiens (human) hypothetical protein
CGGCGACGACGACGACAACAACCACCACUACCACUACUACCACGACUCCGUUGUCUCCGUCGUCG
ACCUUAGACUGGGCGUCGUCGACCUCUUUGUCGUCGUCGUCAUCGUCGCCG
>EMBL_CDS:BAG62663 BAG62663.1 Homo sapiens (human) hypothetical protein
CAAAGAGUCGGUAAUAGUAAUCGAGUCCUCCUUUAUAGUCAAGGACCCAACCUAGGUUACCGUCU
UUG
>EMBL_CDS:BAG60917 BAG60917.1 Homo sapiens (human) hypothetical protein
UCGGUGUGUCUUGACCCCCCAGGUCCUUGGUACUUCGAACCGACACCAGAUCCCUCGGUCCUUAG
ACCUGUCACAAGACCCAGUAUCGGUCCUAAGACCUUAACAUCAUAGACACGUAACGA
>EMBL_CDS:BAG61437 BAG61437.1 Homo sapiens (human) hypothetical protein
CACACCGACCACUCCUCGGACCAGUGGAGGCCCUUGUGGACCAAGGGGUAGUGAGACACGUCGUC
UCCCACCGCCCUGUACAACGAGUCCUCCCUCCGAGGCCUGGCGUGGUGUA
>EMBL_CDS:BAG61141 BAG61141.1 Homo sapiens (human) hypothetical protein
ACUUUAAAUCGAAGGUAGUAAAAACAAAAGUACAGGUCGAGAAAGAGUGCAUAAGCUCUCUCUUU
UACUAGUAGUACCUUUGACGACCUAAAAGAGUCCGACUUAAAGC
>EMBL_CDS:BAG61361 BAG61361.1 Homo sapiens (human) hypothetical protein
GGACACGACCCGUCCGGACGUGGCCCUUCAGGACGUGACCCGUCAGGGUGUGGCCCGUCAGGGCG

UAACCCGUAAGGACCUUGACGUCCUAAUGGACCGUGUCC
>EMBL_CDS:BAG62184 BAG62184.1 Homo sapiens (human) hypothetical protein
ACUCCACUCAAAAGACCCACCCACAUAACGGGAAUCUUCAGGACUGGACAUAUCUAUGUGAGACG
AGUCUUCGAUAGCGGAGU
>EMBL_CDS:BAG62251 BAG62251.1 Homo sapiens (human) hypothetical protein
GAUAAAGAAAAAGAAGAAGAAACCACCAAGUAGCAGUCUCGACGAUAGACACGACCACGAUGACG
AUGACCUUCUCGACCUUAGACUCAGACUUAGUCUCCUCCUCCUUCUCCAACACC
>EMBL_CDS:BAG61188 BAG61188.1 Homo sapiens (human) hypothetical protein
AGGAGAAAUGACAGUGGUUCUCGACGAAAAGGUCUACGACCGAGGAGGAACGAAAAGAGAAACGA
GAGCAUGUAGAAUGCUUAGUGACUCCAUCCCCAUUUCCUUCCUCUUUCGUACGAACACUACCUCC
GUCGUCCCCGUCCCCUUCUCCU
>EMBL_CDS:BAG62653 BAG62653.1 Homo sapiens (human) hypothetical protein
ACCCCGGACUAGGAACGGGCUUCCGUCGAGACGGGUCUCGGACCCACCGCAAGAGCUUCGGGUCC
CGGGCCGUCUGCUGCGACCGUAGUCAGUCCAGGGU
>EMBL_CDS:BAG58852 BAG58852.1 Homo sapiens (human) hypothetical protein
GACGAGAGUACGUUUCCUCUAAGGAGGACGACCAGAACCCGUGAGAGUCUACCCGUCACCUCCCC
CAAGAAGCUGCCCCGUACCCCCGCC
>EMBL_CDS:BAG62270 BAG62270.1 Homo sapiens (human) hypothetical protein
CCCCGUCACUAUAUCCCACGCAACAGUAAGAGCAGGUCGUGUAACUACCACUGUGGUUGCAUUCG
ACUCGCUCCACCCUGCGGUGGUAGGUGACGGAA
>EMBL_CDS:BAG60629 BAG60629.1 Homo sapiens (human) hypothetical protein
CCCCAGACCGAGUCACAACGAACCCCUGGAGACAGAGGUCACCGUCGUAGAUCCAGGGGACGUCG
CGAAGGAAGGGG
>EMBL_CDS:BAG59726 BAG59726.1 Homo sapiens (human) hypothetical protein
CCUUUCGAUCGAAGGAGGCCCUGACGGACCCCGGAGACCGAGGGAAGGUCCUCCCUGAACUCCAC
GACGUCGAGAACCCAGACGUCCUCCAACCAUAAGG
>EMBL_CDS:BAG62080 BAG62080.1 Homo sapiens (human) hypothetical protein
CGAGACCCAGGAACCUCCACGCCUCCAAUUUGGAGUGUCUACCAAACUGGAGUCAGACACAUCGA
AACCCAAACACCUCG
>EMBL_CDS:BAG58688 BAG58688.1 Homo sapiens (human) hypothetical protein
CCCCGUGCCUCCGAAACCCUUUCCACCCCCCGUCCGUUAUCGACCCGGGGUAGGCGAGGGAGUCC
GGGGCAAGGAG
>EMBL_CDS:BAG60605 BAG60605.1 Homo sapiens (human) hypothetical protein

GAGGCUCCGAUAUCCUUGGUUCCGGUCCAGUAACCUCCGUCCCCGAAGGUCUUACAUCGACCGCA
CCGGGACCCCGUACCCGAAGACCUC
>EMBL_CDS:BAG60014 BAG60014.1 Homo sapiens (human) hypothetical protein
AGUAGAGGUGAGAAGACGUCUCCGGAACAAAAAGCUCCAUCGCGACCUCCCCACGGCUCUACU
>EMBL_CDS:BAG62491 BAG62491.1 Homo sapiens (human) hypothetical protein
CCCUAAAGUCAAGGUCUCUCACUCAGAUAGGGCAGAUCGGCCCCUAGAAAUGAGAGUUAGAAAUC
CGUGACUUUAGGG
>EMBL_CDS:BAG62745 BAG62745.1 Homo sapiens (human) hypothetical protein
GACGACCUAGUCGUAGGCGGUGGCACGUCCCAGGUCUUCCAGGAACAUGCCCCUCUACACGCACA
GCCCGCC
>EMBL_CDS:BAG58970 BAG58970.1 Homo sapiens (human) hypothetical protein
UCCACCUCCCCCUCCACUUCCACCUCCCCGACCUCCCCGACCUCCUCGACCACAGUGACCCCCCG
UCCCACGUCGAUGACGACACCGGACUCGGUUGACGUAGUCCAGAAAAAGAAAGAAUUAAAAGUCC
AGAGAGAAGUCGAGAAGGACGUGAA
>EMBL_CDS:BAG62696 BAG62696.1 Homo sapiens (human) hypothetical protein
AAAGAUGUCCGAAGGUCUCUAAGGUACAUGUAAGAGAAUCACUACUACCUCAACAGAUCCUCCGG
AAGUUUCUUC
>EMBL_CDS:BAG59694 BAG59694.1 Homo sapiens (human) hypothetical protein
CCCACCCCAGAGGAACCCCGGGGGAUCGAAGGGGAACCAAUAGAACUCACCCAGACCGAAACCCC
CAAACCCCCAAAGUAGG
>EMBL_CDS:BAG60617 BAG60617.1 Homo sapiens (human) hypothetical protein
CCACCCCUACUUCCACAAAAACGCGUUGUGUCAAUGGUUGUCCCUACCCUGACACUACGAACAUC
CGUCGGAAGGAGAGACGG
>EMBL_CDS:BAG60390 BAG60390.1 Homo sapiens (human) hypothetical protein
UAGGACCCUGAAAUACACCAGUCGUCUUAAUGUAAGAAAUUACAGUCUCAGUAGCAAACCUCAGG
GUCCUA
>EMBL_CDS:BAG62105 BAG62105.1 Homo sapiens (human) hypothetical protein
GGAGGAGCGGGAACGAGACAUGGUCCAGGUCUUAAAACGCCUGGAACAGUCCGACCCACCCCGUC
C
>EMBL_CDS:BAG59014 BAG59014.1 Homo sapiens (human) hypothetical protein
UCGGACCGGUGUCGGACGUAGUAGAGUAGGGUCUCCGCCUCGUGUCCGAGCCCCAGGAAGUCCCG
GACUUCCAAACACCAACCGUGACAGUCCUACUACAACAGGCACCGGUCGA
>EMBL_CDS:BAG62713 BAG62713.1 Homo sapiens (human) hypothetical protein

UCCCCAAAGAACUCUUCGUCACUGAAGAAGACCUGAAACCAAGAGAAGAAACAGUCGGGAAAAAG
GAACCUCGGUCACAGGUGCUUCUUCUCAAAAAGUAAACCCCGGAGA
>EMBL_CDS:BAG59538 BAG59538.1 Homo sapiens (human) hypothetical protein
AGGGACGACGACCUCGUGUCCGACCGACUGUCCUACUGGUCUCCGUCCUAUAGGUCGUCAUCCCU
>EMBL_CDS:BAG62640 BAG62640.1 Homo sapiens (human) hypothetical protein
GAGCGACCAUCAGAACUGCUGCCCUCAACCAUCGUGUGUCCACUCGACGGUCGGUAUCCGGCGGU
CGCUC
>EMBL_CDS:BAG59148 BAG59148.1 Homo sapiens (human) hypothetical protein
UCGUUAUCCGAGGACUAAACAAUUUGAUAAACUACAGACAGGUUAUCAUACUUUUAUCUUCAGUC
CUUGAGAACGA
>EMBL_CDS:BAG59956 BAG59956.1 Homo sapiens (human) hypothetical protein
CCAGUGACCCCGAGAAAGUCACGACCGACGUCACUUCUGUGACUACGGCGAUGACUUCGACCUCA
GUGUCCUUGG
>EMBL_CDS:BAG61877 BAG61877.1 Homo sapiens (human) hypothetical protein
CACCUCUCCCACGGACGACCCCCUGACCCCGGAAGCCGUGUCCGGGGAACAAUAGGGUCCACGGU
UACAGGUA
>EMBL_CDS:BAG60351 BAG60351.1 Homo sapiens (human) hypothetical protein
UACCGAGGUCACACCCCACGACCGGUCCGGUCAAGCACCCCUGCAUGUACCCGACCCCACCUCGG
GGGUCGGAGAUGGGGACCCCUCCUACCUCUGUGACCUUCAAAACCGUGACCUCGGCA
>EMBL_CDS:BAG61210 BAG61210.1 Homo sapiens (human) hypothetical protein
CCCGACCCCCUACCACCUCUGUGACUCCGGACGUCACGGGAGACGUCUACUCGGGCGAGGGAGAC
GAGGACCCCGAGCCCGACUAACGCGUGACGACUCCCACCGGGGUCGAGGAGGUGAAAGGGGAAGA
CGGA
>EMBL_CDS:BAG60577 BAG60577.1 Homo sapiens (human) hypothetical protein
CCUCUGGGGCACUCCGCGUCCCAAGGACCCCCGACCUAGUACAGAGACUCGUCGGAGACCGAGAC
AAAGAACUCCCCAGACG
>EMBL_CDS:BAG58638 BAG58638.1 Homo sapiens (human) hypothetical protein
GUCUCGAGGGGGGUCAACCCGGGCACCUCACUCCCGAGGUCCGGACGACGUGCCCCUACAAGAC
>EMBL_CDS:BAG60983 BAG60983.1 Homo sapiens (human) hypothetical protein
CCACAGAGCUCUAUGAACAGUUGCCGUAGUCCCCAUGACCGACGACAACAUCCUUGAGCUUGGUG
G
>EMBL_CDS:BAG62808 BAG62808.1 Homo sapiens (human) hypothetical protein
CGACACGUAGACCCGGCCCUACUUCCGCCCGGCGGGCAAGUAGAACCGGUCCCGCAUGUCG

>EMBL_CDS:BAG59077 BAG59077.1 Homo sapiens (human) hypothetical protein
AGAAGGAACGGAGGAUUUUGUGGUGACGAAGGGACGAGGUAACCGACUCCGACUAGGUCCGACAG
UCCACCACCACCACCACCGUUUCUUCU
>EMBL_CDS:BAG61752 BAG61752.1 Homo sapiens (human) hypothetical protein
CCGAGACCCGAGCCGACUCCUUCAUCGACCGGUCCUUCGGGUCGAACGGAGGUGUGGCCCAGAAC
UCGG
>EMBL_CDS:BAG59247 BAG59247.1 Homo sapiens (human) hypothetical protein
CGACACCGGCGGUGAAGCCGGUUUAGGACCCCGGGAGUGCCGUUCGUGUCCAGGGUCCACGUCGA
CACCGCCGUCGUCG
>EMBL_CDS:BAG59760 BAG59760.1 Homo sapiens (human) hypothetical protein
UAAGUACUGAAAGUACAUCUUUAUUCCUUUGUCGGUUUAUUGGAUGAGAAAAAAGAUUCUCCAAA
GGAACAACCCGAUUGAACUAACGUACUUA
>EMBL_CDS:BAG62203 BAG62203.1 Homo sapiens (human) hypothetical protein
GGCAUCAACAAGACCCGCCCCUUUCGUAAGUGACCCCUCUGAGGUACCUUCGGUAGGAGUCGAAA
CAGAGGGAACCCCUUGAAAGGGACGGUGUCCAACCACGCC
>EMBL_CDS:BAG59795 BAG59795.1 Homo sapiens (human) hypothetical protein
AGAGGUCAGAUCCCAGACUACGAGGAACGACGUCAUUAUACGAAACACCGUAGACCUCGUGCAAA
ACCCCGGAUUUGUCGGUGUUUGGGACGUCUCUACUCGUGGUCUGAAUUCGACCUCU
>EMBL_CDS:BAG60566 BAG60566.1 Homo sapiens (human) hypothetical protein
ACAAUUCUGUUACGUGACCCCGGACUUCAAAAAAUGCACCAGAAGGUGACACCAAUUAAGGUCUC
GUAACAGAUUUCGU
>EMBL_CDS:BAG59668 BAG59668.1 Homo sapiens (human) hypothetical protein
AAAAACCCCUAUGCACGAAGACCCCCGAGUGAUAUAAAUUACUGAUGUGGGGCCUUUCACAACCA
CAGGAGUUCUU
>EMBL_CDS:BAG59450 BAG59450.1 Homo sapiens (human) hypothetical protein
AGACGUCGGAACCUCCCCGAACCCCGGAUCCGUUCCGGACCGGAUGACCGGGAAAGUAGACACCU
>EMBL_CDS:BAG61934 BAG61934.1 Homo sapiens (human) hypothetical protein
CGGUCCACAAAAGUCAGAAGGGUCUACGAGAAAGGUACUAACUUGGGUAUAGAAGGAGUCCUUUA
UACCUUUUAGGAAACCUUGCAUAAACCGUCAAACCUCCGAAUAUCGGACCG
>EMBL_CDS:BAG59929 BAG59929.1 Homo sapiens (human) hypothetical protein
CCGGUCCGACCCCGACCCCGGCCACCCUCCUGACUUCCUUUUCUUCCGACCUCCGACACCCCCGG
GUCUCCGAAACCCGUCGACGAGACAGGUACGGGCGUCCGGAAGCAUUCAGACCGA
>EMBL_CDS:BAG62150 BAG62150.1 Homo sapiens (human) hypothetical

protein
AGUCCUUCCCUAUAGGUUGAAAUGUACACUAUAAUACUAAUCUAAGUGUUGACAGAAAAACCUAU
CGGAGUUAAGGACU
>EMBL_CDS:BAG59519 BAG59519.1 Homo sapiens (human) hypothetical protein
CGGGAACCUUCCCCACAGGUCGAACUCCGGUGGGUCGACAGGCUCAGGUGCGUCCUCGACACUGC
CAAGGAGCAACAAGUCGUCCCGAGAACCGAGUCCGACCUCUCUUGGAAGAGGUACUCCG
>EMBL_CDS:BAG59120 BAG59120.1 Homo sapiens (human) hypothetical protein
ACUAAAAUUUCAGGAAACCUGACUAUAUAGAUAUGACAGGACAGGUGUCCCGAAAGUACUAAU
>EMBL_CDS:BAG62911 BAG62911.1 Homo sapiens (human) hypothetical protein
CCGUCGAGAAAAACCUCCCCCUGUCGAAGGACUCCCUCUCCCCCUGGGUGAAGGAAAAGUCGUCC
CUCAACCCGUCCAUCGUCGAAAAGGACAACCUCUCCCCACCGGAGGAACUGAGACAGGGAGUUUC
CCUCCCGG
>EMBL_CDS:BAG61567 BAG61567.1 Homo sapiens (human) hypothetical protein
GCCUCCCGACGAGUGGUGGUCGACCACGGAGGACCCUAGGAAAUGUUAGGUGGUCAAUAACUGCA
GAAACUUCCCCAUUGUCCAGUGGACAACGACCACCCUCGACGGAGCCGGC
>EMBL_CDS:BAG61509 BAG61509.1 Homo sapiens (human) hypothetical protein
GGUUUCGGUCCCAACCACUAACCACGUGGUGACUUCGUUCGUGACCACGACUGUCGUGGUGACUU
CGACCGUGACCACGACUGCCGUGUCGGUUUCGACCGUAACCACAACUCACGUGGUGGUUUCGGUC
GUGACCACGACC
>EMBL_CDS:BAG62941 BAG62941.1 Homo sapiens (human) hypothetical protein
AGGAACCCCAGGACCCCACGGUCACCGAGGAACGAAGGGACCUCGUGGGACCCCAGGAACCU
>EMBL_CDS:BAG62171 BAG62171.1 Homo sapiens (human) hypothetical protein
AUCGGAUCCUCGAACUGAUGCCCCACUGGCGGUCCCCGUCGAAACUGUCGAGGUGCCGACGGAAG
ACAGAGUAUCACCCGGGCCACCACCCGAGAUCCGAC
>EMBL_CDS:BAG63132 BAG63132.1 Homo sapiens (human) hypothetical protein
UCUAGGGGACUUAGAGUCCGUCCGUAAAGAGACACCUUCCCGACAUGAAGACUCUUUCCCCUGAG
A
>EMBL_CDS:BAG59555 BAG59555.1 Homo sapiens (human) hypothetical protein
CCUCAGGCCCUCAGACCAGUAGGGCCCCUACGUGCGUCCUCGAAGGCCCGUACCGCAACCCGUAC
AGGACGAACAACCGUUUAUGGUCGUCGUGACGUAGGGCGUCGAGCAGGAGGACGUCGUAGAAGAC
CUCAAGUAGUCGUCUAAGAACCUGGG
>EMBL_CDS:BAG60174 BAG60174.1 Homo sapiens (human) hypothetical protein
CUCGUGUUGACAAAGGACACACACUACACAACAAGAGACCGACACGUGAAGGGUCACGGUUGCAG
GUGUAUCCUGUCCCACACGAA

>EMBL_CDS:BAG61723 BAG61723.1 Homo sapiens (human) hypothetical protein
UUGGGAACAAACUCGACUUCUUCGUCAAAGGCUCACCUACGACGGUAAGACCGUGUUCUUGUCCC
CAA
>EMBL_CDS:BAG62260 BAG62260.1 Homo sapiens (human) hypothetical protein
GAGACCAUAUCGUCCCAGUAACCCCGGCGUCUCUUGACUCCGAAGGGUAAUUACUGUAACCAGAC
CUACGGUCUC
>EMBL_CDS:BAG59789 BAG59789.1 Homo sapiens (human) hypothetical protein
AAACCAGUACAACAUCGAAGUUUGGAACAUCGAGUACCAAAAGUUCAACGUUGUACAGGUCU
>EMBL_CDS:BAG59777 BAG59777.1 Homo sapiens (human) hypothetical protein
CUUCCGUUGACAGGUCCCCCUAAACAACCCAUAAAACCUUAAAAACUUUGACCUUCUCAACGGAG
UCGUUUAAACACCUAUAACAGAAG
>EMBL_CDS:BAG62660 BAG62660.1 Homo sapiens (human) hypothetical protein
ACAUGUCCGACCAACCCCCCCACAGGAGACACUACGUCACAAAGGAAACGGAGGCUCCUGGGUGG
GACGGACCGGGGACACGU
>EMBL_CDS:BAG61680 BAG61680.1 Homo sapiens (human) hypothetical protein
CUACUUUCAACCCGACCGGUCACGGUACGUCCACCUUCCUGACGACCCACCGUGACAGUGGUUAC
GACAGUAG
>EMBL_CDS:BAG61884 BAG61884.1 Homo sapiens (human) hypothetical protein
AACUUCGGCUGUCGACACACGACUCCCCGGAACGCUUCCCGCAACCAGUAGCAAGAGGCCCGGAG
ACUCAUGAGGUCGACCUACCGAAACU
>EMBL_CDS:BAG63104 BAG63104.1 Homo sapiens (human) hypothetical protein
CGACGUCACCGAACCCGCAGUCCUCCGAGUGACUCCCCCGGUGUACUGGGGUCGGUCACUGUCA
>EMBL_CDS:BAG59267 BAG59267.1 Homo sapiens (human) hypothetical protein
CCUUGUGUAACAACUCUAACAGUAACUGCUACUCGUCGAGGAGACAGUCGACGAGUAACCGCUAG
ACUCCCUACUCGAGG
>EMBL_CDS:BAG59479 BAG59479.1 Homo sapiens (human) hypothetical protein
UUCUCUCUUCCCUAAACUUUACCUCAACCAGGGCUGCCAUCUGAUGAGCCCGAGCUCAGAGAAGA
UAAAGAA
>EMBL_CDS:BAG62102 BAG62102.1 Homo sapiens (human) hypothetical protein
UCCUAGACUUCCGUUAUUUAUGACCCCUCGGUAGCCCGAAAAGUAUAAAGGUAAACGGUUUGUAC
UCUAAGA
>EMBL_CDS:BAG61695 BAG61695.1 Homo sapiens (human) hypothetical protein

GUCGCUACAACCACCCGGUUUCGGGUCGGGACUUCUUCCUCCCCGACCCCGGUGUCGUGGCGAC
>EMBL_CDS:BAG62265 BAG62265.1 Homo sapiens (human) hypothetical protein
CAUGAGUAACUAUAACCACAAGUGCAGGACCUCCCGUCUCUGUCGGGAGACUUCCUGUCCUAUCC
GUGACCGUAGUCCCUGACACAAGAGGUCCUAAAAGACGUCGUCGUCACCCAUG
>EMBL_CDS:BAG61782 BAG61782.1 Homo sapiens (human) hypothetical protein
UAGAGGACCUAUCAGAAAACCCCGUGUGCCAGGAGGUCGGUCCCCUACCACGCGGCCUAGGUGAG
GUCCUCCA
>EMBL_CDS:BAG62806 BAG62806.1 Homo sapiens (human) hypothetical protein
GUCGUGGUCGAGGACGUCGUCGGUGGACCAGCGGCAGUUCUUCCGUCUUUAACCCGACUGGUCCC
CGACCCGAC
>EMBL_CDS:BAG60749 BAG60749.1 Homo sapiens (human) hypothetical protein
CCUAACAACAGUCAAGUAAGUACCCCUAAAAGUUCUACCAGGAGAACUACUUGAGUCGUUGUCAG
A
>EMBL_CDS:BAG60166 BAG60166.1 Homo sapiens (human) hypothetical protein
ACUCUUUGGUCGACACACCACCCCAAAGGUCUCUUCUCCUUUAAGGAAGAAAGGACCUCUCGCAC
GUCGACAUUGAGU
>EMBL_CDS:BAG62068 BAG62068.1 Homo sapiens (human) hypothetical protein
CGGACCGGAACCAGGAAGACCGGACUUCUCUCGAGCGCCCGGGCCCGGAAAUAUUCCGACGUCAU
GUGGUCCCGGUCCG
>EMBL_CDS:BAG58809 BAG58809.1 Homo sapiens (human) hypothetical protein
AGGACCCCAUAGACGAAGGUGUUACUUAGGUCAUUUCCGAAAUAAUCGUCCUCGAAAUCCU
>EMBL_CDS:BAG61318 BAG61318.1 Homo sapiens (human) hypothetical protein
CUCUAAACUUGACUCUAAACGUCGGUAGUUUAAUGACAAUCAAUCUAACUACCCACCGUAACAGU
GUCUUGGAGGCGUUACACCUAAAAGAAAGUUCGGUAAUGUCAAUCACGACCCUAACUAUCCCAAA
CCACCAAGUUACCAAUGUUCAAAA
>EMBL_CDS:BAG59722 BAG59722.1 Homo sapiens (human) hypothetical protein
GGAACCGGCCCCGAUAAAGACAAAGACCCUCAACUCGGUACAGGAACUGACCCCGGUGGAGACGA
AGAUUUCCAUCACGAAAUAGGAACCAGUUCUAGUGCAACCAAAGGCCCCCGUCACAGUACAGGGA
CCGGUCCC
>EMBL_CDS:BAG61783 BAG61783.1 Homo sapiens (human) hypothetical protein
CCCGAAGAGGACGGCCAACUACUUGAACUACCGGAGCAGGUGCGAGACGUGCAAGUGCUACCCGU
CCUACCCGGG
>EMBL_CDS:BAG61220 BAG61220.1 Homo sapiens (human) hypothetical protein

CGAGGUAGGUGUAAGACCUUUGCUACUCCCGUCACGGAAGUGACGGGACUUGAACCAGUCCUACA
CACUUCUCA
>EMBL_CDS:BAG59869 BAG59869.1 Homo sapiens (human) hypothetical protein
CGACCCUUCGGGUCCUAGAUCCAGAGGUAGGUCCGAAAGGUCGAGACCCACCUUCAGAAGGAGAA
ACGGGUCG
>EMBL_CDS:BAG61084 BAG61084.1 Homo sapiens (human) hypothetical protein
AACAGGUGUCGAUCGACAUCCUUAAGUAGGUCGACGACGGGACGUCCCACGAACAACUCCUCAGG
ACGUUUCGUGAACCGUCGUCGCCACCUUCUUUUCCCCGUCGACACCGUC
>EMBL_CDS:BAG59325 BAG59325.1 Homo sapiens (human) hypothetical protein
AGGUCCCUCAAACCCCUUCUCAACAGAAGAUUUCUUACGAGGAAGAGUCUUUAAGAGAGUACCU
>EMBL_CDS:BAG61661 BAG61661.1 Homo sapiens (human) hypothetical protein
ACCAAAAUAAAACUUCGAGAAACCUUUCUUAAGGACAUUGUGAGUCGUGAGCUAUGUCCUUAAAG
UUUGUUUUCUGUUUAAGUUUAAACCUUUGGU
>EMBL_CDS:BAG58962 BAG58962.1 Homo sapiens (human) hypothetical protein
CCACCGACUGAUCCUCUGUUUAACGGUAGAGGUCAUCGACUACUUUGUCUCCUGAGUCCGAAGAG
UCGAAGACGACGUCCCUCACCUCCUCCGACUCAUCCGACGAAAUCCAGUAUCACGAAAUUAAGAC
CUUCACGUAAUUAUAGAGGACCUGUUAGUAG
>EMBL_CDS:BAG62164 BAG62164.1 Homo sapiens (human) hypothetical protein
AAACCCGUCACGUCCACCCGAGCGUCAAGAGGCGAAGAACCGGAGUGAUAAACACGACGGGUUU
>EMBL_CDS:BAG60980 BAG60980.1 Homo sapiens (human) hypothetical protein
AACACCACCCCGACGAGGUGUAGGAGUGUUGGCACGACCAGGACCCACCCUCACUUCGGGAGUAG
CGUC
>EMBL_CDS:BAG59420 BAG59420.1 Homo sapiens (human) hypothetical protein
AUACCCCGGAUAUAUAAAGGUAAAAAGACAUAUUCGGUAUUAAAUUGUCGUUCAAUACCCAGUAC
GCCUAAGAUACUCCGAAGUAC
>EMBL_CDS:BAG62711 BAG62711.1 Homo sapiens (human) hypothetical protein
CGGACUGGGGUCCUGGACCAAAAGAGACUUCUUCGUCUUGACCUCAACUUGUAGGUUGGAGACAC
CUCUCUCGGCCCAGAGUGUAAAGUCCG
>EMBL_CDS:BAG61734 BAG61734.1 Homo sapiens (human) hypothetical protein
AGUGUCCCCGCCAGACCCACCAGCGUCCCAACCAUCAACAUGUGAACACACCUUCUCAAUAGAAA
GAAGUCGACUUAGAAGGACGUCACACGUCGGGUGUUAGACACGAAAGAAAAGAAGAAGACACU
>EMBL_CDS:BAG60578 BAG60578.1 Homo sapiens (human) hypothetical protein
GACCUCGUCCCAGGGAACCGGUGUUCACGACCCUACGAGACAACCAAGAACGACUGGGACUACGA

CCACGACCCGGACCCAUCCCCCGGAACGACGGUC
>EMBL_CDS:BAG61098 BAG61098.1 Homo sapiens (human) hypothetical protein
GGGUUCCAGAGUCGAGGUCAUAUCCUCCGUCUCGACCUUUUGAAAACGUCCAGGUCGAGACACGU
AAGCUACCCCGACACCUGGGUCACCC
>EMBL_CDS:BAG59084 BAG59084.1 Homo sapiens (human) hypothetical protein
CCGACUACAGUCGUCGUACCUCGUCAUCUCUGUCCCGGAACCCGAACAGUUAGGAGAGGUCGACA
ACGUUCCGA
>EMBL_CDS:BAG62127 BAG62127.1 Homo sapiens (human) hypothetical protein
AGUAGUCCUCUCAAGGAACUCGGUAAGUGGACCGAUCCCAAUGUAAGGUAAAACUACCACUGGGA
CCGACCCCCAACUCUGUCGGUUAGGACGACU
>EMBL_CDS:BAG64836 BAG64836.1 Homo sapiens (human) hypothetical protein
ACAUGUCUUUAGACAGGGUAAUUAUCGUCAACAUCAUAUACGUAAGACCAACUCUACCUCGUCCA
UAAACAGU
>EMBL_CDS:BAG65121 BAG65121.1 Homo sapiens (human) hypothetical protein
ACCUCUACUCCAUCGACGGUCCACGGUACCCCCAUAUCCACUGCGCCAUCGGGAGUCCCGUGAGG
ACCGUUAGGUAGAAGU
>EMBL_CDS:BAG64320 BAG64320.1 Homo sapiens (human) hypothetical protein
GAGACUUCCACCCAGCGUGACGACCCAGACGGAGGGUCGUAACGACGUAGUGAACCAUGUCUC
>EMBL_CDS:BAG63685 BAG63685.1 Homo sapiens (human) hypothetical protein
CUCGAGUAUCAGAAGGACCGACGACCGACAGUCACUACCCCUGCGUAACUGGAGGUCCUCGGUGC
CGAACUCCAG
>EMBL_CDS:BAG36645 BAG36645.1 Homo sapiens (human) hypothetical protein
CCGACCUACAACACGAACUACGAAAGUAAUGAAGACAGUUGUUGUCGACCACUACUCCUCGGCAA
AUAAUGUUGAAGUCGA
>EMBL_CDS:BAG64189 BAG64189.1 Homo sapiens (human) hypothetical protein
CACGGGAACGGUAGGUCACCCUGAUACCCUAUUACCGUCAAGAGUGUGUGUAGCUACAAAAGGUA
GGUCAGUAGAAGGCCCACACUCGACGUCGGUGUCGGGUACCAGGGACUCCCUUACCUUCUCCCCC
G
>EMBL_CDS:BAG64538 BAG64538.1 Homo sapiens (human) hypothetical protein
AACCCAGGACCAUUACUAGCGUACAUGAGGUGUGUGGUGACACCUUAGACACAACAAAAUGGUCU
GGGUC
>EMBL_CDS:BAG63986 BAG63986.1 Homo sapiens (human) hypothetical protein
UCCUACCGUUGUGGUGAAGAUUUCGUGUCUUCUUGAAGUCCGAAACGAAGCCUUCACCGUGGUAC

GAUAGAA
>EMBL_CDS:BAG35483 BAG35483.1 Homo sapiens (human) hypothetical protein
CGGGUUAAAAGAGGUAGUCCGACAGAGACUAACACGACUGACACAGAAGUAGUGUAAGGUCGUCC
GAAGCCUGUUCAGACAAUAUCAUAGACAACCUCUUCACUAACCCG
>EMBL_CDS:BAG64566 BAG64566.1 Homo sapiens (human) hypothetical protein
GAACCGUUGGUACACCCCCGAGUGUCUCGACAGCUGAGUCCCGGUCAGAGACUUUCGGAAGUGGU
ACCAGUCACGACUC
>EMBL_CDS:BAG36692 BAG36692.1 Homo sapiens (human) hypothetical protein
GGUCCUUAUUCUUUCUCCUACUCAUUGUCCUUACACACGGUCCUGACGACGUAUUGUAGUAAACC
GUCAUCUUUCGCCGUGUAUAAGCGCCAGACCAACAAUAAAAACC
>EMBL_CDS:BAG35778 BAG35778.1 Homo sapiens (human) hypothetical protein
CCGACAACUACAUCCAGAACCUCAGGUUCCAGUCUCAGUGAAGACUAUGAACUGGAACCUAGACG
CUCCAGAAUAACUCCAG
>EMBL_CDS:BAG64784 BAG64784.1 Homo sapiens (human) hypothetical protein
GUUUCCACAGGGAAUACCGGUCCAGACCCGACACCUUCUAGACUCUCUGACUCCACUGUGGAAAC
>EMBL_CDS:BAG34957 BAG34957.1 Homo sapiens (human) hypothetical protein
CCCGUCGUAACUGACGGAAAACCGACCGUCGUGACCAAGACAAAACCACCCGUCGUAACUAACGG
AAAACUGACCGUCGUGACCAAGACGACACCACCCGUCGUAACUAACGGA
>EMBL_CDS:BAG63386 BAG63386.1 Homo sapiens (human) hypothetical protein
ACGGGGCUCAGGUCGGAGAGGAGUCCCAGGCCGUGUGAGUCAAACGUCGACCCCUCUCCCUACCA
CGCCCCGU
>EMBL_CDS:BAG36091 BAG36091.1 Homo sapiens (human) hypothetical protein
GGCCCUCUGAAACCCACCUCCGUCGGGUUAUAGUUAGAAGAGAAACCCUAGACAGAAGAGGGGCU
UUAUGAGAGCC
>EMBL_CDS:BAG65367 BAG65367.1 Homo sapiens (human) hypothetical protein
CGGACGUCGGUGAAGAAGGACUCCUGAGUCCCAGUACCGGGACGGUCGGUGGUCCACCAACCAAC
CCCGACACGAGAGUCCUCGUCUCCACACGCGUGUCCA
>EMBL_CDS:BAG64234 BAG64234.1 Homo sapiens (human) hypothetical protein
CUCCCCCGCCUCCGUCGACCCAACCCGAAGGAAAUCCGUCCCCGGUCCCACCCGUACCAACGGAG
GACACGGGAG
>EMBL_CDS:BAG65426 BAG65426.1 Homo sapiens (human) hypothetical protein
GAGGUGGUUCUUUCGACCCCUCUCCCGUUCCAGUCUCCCCGUUUCUGAAAAGAGGUCCCUACGAA
AGAACCCCCUC

>EMBL_CDS:BAG65387 BAG65387.1 Homo sapiens (human) hypothetical protein
UCUAUCAAACUUGGAAAAACCUCCACAGAAAGACUCUUAGUAGGAAGAGCAAAAAUAGGACCUAU
ACCAACUCCGAUAGA
>EMBL_CDS:BAG63854 BAG63854.1 Homo sapiens (human) hypothetical protein
CUGGAAGACAUCGUGGGUCGGUACCUUCUACCGUGAGAGUAAGUGUCACACGAGUGGGUACCGUC
CCCCGUCGUCACCCAG
>EMBL_CDS:BAG64988 BAG64988.1 Homo sapiens (human) hypothetical protein
CCUAGUCGUAGUAGGUACCGCCACGGUCACCACCACGGCAGGUGUCACCCCGACCGUGACGACCG
UGACAGCACCAGG
>EMBL_CDS:BAG63267 BAG63267.1 Homo sapiens (human) hypothetical protein
GAGGGCUCGGAGACCCCAGAAACUCUGGAGACACCGGAAACUCUGGGGUCUCCGAAAACUC
>EMBL_CDS:BAG63399 BAG63399.1 Homo sapiens (human) hypothetical protein
AGACUCACGUUCGUACCGGACCCCACUGGUACAACCCGAAGUCGUUCCACGGAAGGUCCAUCUAC
ACCACCAGUGAGUCU
>EMBL_CDS:BAG35659 BAG35659.1 Homo sapiens (human) hypothetical protein
CCUUGUUCCAAUAUCUCUACUUUGAAGUUUCGAAGUGAGAAGUCAUUCGAUAUUCGGUAGAGACA
UUCUAACUAAGG
>EMBL_CDS:BAG35102 BAG35102.1 Homo sapiens (human) hypothetical protein
GUGGAAGAAACGGGUAGUCGUUCUGAUCGCAGAACAGUCAGAACCACUGUCACUGUAACUUCCAC
>EMBL_CDS:BAG64453 BAG64453.1 Homo sapiens (human) hypothetical protein
CCUUCCCUUCUCCGGGUUCUCCGAAGGACAGGGGAACCCCCGUCGUCUCGGUUUACGAACCCGUG
CCCGGGUUCGACGGACGGGACGUCCCCAGGUCCCUCCAGGACUACUCAGGACGGGGAAGGGAAGG
>EMBL_CDS:BAG63864 BAG63864.1 Homo sapiens (human) hypothetical protein
CACGUAGUUAAUAUUUACCCAUAACUCUGUUUUCCAGAAAUUCAGGUUCUUCUCGUGAAACAAAU
UGGGGUCGUAACAUCACUACGUA
>EMBL_CDS:BAG64667 BAG64667.1 Homo sapiens (human) hypothetical protein
CUCCACCAAUCACAAAUAAAGUCCGUAGUAAUGUUUUACAGACUAGACAAGAUCUUGUGACUAGU
CGGAG
>EMBL_CDS:BAG36301 BAG36301.1 Homo sapiens (human) hypothetical protein
CCGGAGGACCGUUCCGGAGACCCGUGACGUAGUCUCGGACACGGGGAACAGAACAGGUCUCCGG
>EMBL_CDS:BAG65215 BAG65215.1 Homo sapiens (human) hypothetical protein
AGAAAGACCUCGUUAUUUUCCAAGUACCAAGGCAAAUAAGGGUGAAACCUUCGUUCUUGGAAACU

ACAGAAGUCGUUCC
>EMBL_CDS:BAG64492 BAG64492.1 Homo sapiens (human) hypothetical protein
UCGAAGAAGUCGAUAGGCAACCCUUACACAAAGAGGUCCUUCAAAAGGGCCGCUAUAGUAUCGAC
GACUCCGA
>EMBL_CDS:BAG63777 BAG63777.1 Homo sapiens (human) hypothetical protein
ACGACCCCAGCGUCUGUCCCAGUUUCGGCAGGUUACCCGUCCUCGUCCGGAGGUACGUCGAACCU
CUGUCGAGGUCGGGUCCCACCCGUGACCAGACCACACACUACGGGCGU
>EMBL_CDS:BAG35982 BAG35982.1 Homo sapiens (human) hypothetical protein
CUCUGGUAAAGGGGUACACUUUGUAGACCGAACGCUGUCCACUAAAAAAGUGUCCAUCCUCCCGA
ACCAGAG
>EMBL_CDS:BAG36045 BAG36045.1 Homo sapiens (human) hypothetical protein
GUCCUCGACCCCGACCUUGAAGAAGACCGUAAACGACAAGUAAGUGUUGACGGUAUAAUCCUCGA
AGUCGGUAGUCAACAAC
>EMBL_CDS:BAG64969 BAG64969.1 Homo sapiens (human) hypothetical protein
GGACCUGUCGGGACGCGACCCAAUUCCUUACACAGAACGUACAGUUUCUGGCCCACGUCCCGAAG
AACC
>EMBL_CDS:BAG63766 BAG63766.1 Homo sapiens (human) hypothetical protein
GGGUCACAAGUCGAACCCGAACAACAACCCACUCUACCAUUGUUCGGAGGCCUACUGUGACCC
>EMBL_CDS:BAG65551 BAG65551.1 Homo sapiens (human) hypothetical protein
CUUCCCCCACAACCCCCUCGGUAGGUUCCGGGGUGACACCGACACGUCGUCGACGGCUCGUAGGA
CCUACCGAAACCACUGUCGCAGGGGAAG
>EMBL_CDS:BAG65101 BAG65101.1 Homo sapiens (human) hypothetical protein
UGAGUCCCAGUCGCGGUAAGAAGAGGAGGUCCUACCUGUGGUCUAACCGGGUCCCUCCUCGUGGC
CACCACGACCGGUGUCUCA
>EMBL_CDS:BAG64658 BAG64658.1 Homo sapiens (human) hypothetical protein
GAAGAAGGACAAGAACAAAGGAAAAGGACACAAAUUUUUACUUGACCCUAACCUUAAGAAGUUAU
GUACUCCUUC
>EMBL_CDS:BAG63325 BAG63325.1 Homo sapiens (human) hypothetical protein
CGGCACUAGGACACGAAGAGCAGCGGAGCGAGGUCCUCGCCCUACUCGUCCAUCUCCUACGCCG
>EMBL_CDS:BAG36590 BAG36590.1 Homo sapiens (human) hypothetical protein
CCGAGGGACUCCUGAGACCCCUCCUGGACGACUGAGUCGUCGUCCGUGAAGGAGGUCCCACGGUC
CCUGG
>EMBL_CDS:BAG63870 BAG63870.1 Homo sapiens (human) hypothetical

protein
UGUCGGUCUUCGAGGUGUCCGAGGCACAUCAGGACCCCGAGGACCUACCUUUGGGCCCGACGCGC
CGAGAGGGACACAGGACGCGACA
>EMBL_CDS:BAG36246 BAG36246.1 Homo sapiens (human) hypothetical protein
GUGCGGCUUCCCGACGUGCGGACCUCUUCCCCCACUCAUAAAAAAAUAGCCACCCGACCUUCUUC
CGUACGAGUUAGGUCCGCUCCGAGAGGCCACAC
>EMBL_CDS:BAG35665 BAG35665.1 Homo sapiens (human) hypothetical protein
AUAUCGUAAAGUCAACGGACACGUUCUUGAACCCCAUCUCUCGGUCCAACUUCAAACGUGUCAAG
UUACUCCUCACAACAC
>EMBL_CDS:BAG35966 BAG35966.1 Homo sapiens (human) hypothetical protein
CCGAACCUGAAUAACAUGUGGUCGAGGUACGAGAGGACACCUCAACUCUCGUACAGGUCCGA
>EMBL_CDS:BAG64463 BAG64463.1 Homo sapiens (human) hypothetical protein
AAACCUUUGGUCACUUUUAGUUAUCCCAUUUAUAGUAGUCCUUCGAAAUGAUAAACUCAACAAAG
UGACCAAUUGUUC
>EMBL_CDS:BAG36729 BAG36729.1 Homo sapiens (human) hypothetical protein
CCCACCCCCGACCCCCAACCCUUUUCGGUAAAGGACUCUUCACAGGCCGAGGACCCAGAGUGAG
>EMBL_CDS:BAG36728 BAG36728.1 Homo sapiens (human) hypothetical protein
GACGAGGGGAUACUCGUGGUGAGCCAUCAUCCACAAGGGACUGAGUCUCCUCUACUGACUCAGAC
ACCUCAGUCUCCUGGUCGAAGCCGCCGUCACCAGGAGUCCCCAUCGUC
>EMBL_CDS:BAG36475 BAG36475.1 Homo sapiens (human) hypothetical protein
AAACUACUUCCUCUUCCACUUCCUCUUCCCCGUCUUUCACAAAGAAGAACAAGUUAGAGAAAAAG
GUCGAAAUAGUUU
>EMBL_CDS:BAG36715 BAG36715.1 Homo sapiens (human) hypothetical protein
CGAACUGUCCUACACAGGUCCUCCGGCAGGAAAGGAGAAAAAUCCUUCCCCCGAAGGAGCUACCC
GUGUAAGAAGAAGACG
>EMBL_CDS:BAG64621 BAG64621.1 Homo sapiens (human) hypothetical protein
GACGUCUCAGACCAACCGACAGAGGUCUUCGAGGAGCCUCGAGAGGUCCCGGGCGCACGUCCGUC
GGUCGACCAAGACGUC
>EMBL_CDS:BAG64291 BAG64291.1 Homo sapiens (human) hypothetical protein
GACCACGCAAAGAAGGAGGAUGCCGAGUUCGGGAGUUAAGGACCUCUUAAGUCCCGUGAACCCGA
CCUCUCCCUGCUUAGUC
>EMBL_CDS:BAG35520 BAG35520.1 Homo sapiens (human) hypothetical protein
CCCAGACCAAGAACACAUAUUCGACCGGCCGGUGGAGGUCCCAGCGUCUCGUCCUCUUCGACGCG

GGCAACAGGAGACCCCACCGGAAGUCGUCCUCGACCCGGG
>EMBL_CDS:BAG65220 BAG65220.1 Homo sapiens (human) hypothetical protein
AAACUUACACUAGUAAUUCCGUACUCCUUAAAGAUUUCCUAGAAGGUGUAAGAAAUGUAAGUUU
>EMBL_CDS:BAG35534 BAG35534.1 Homo sapiens (human) hypothetical protein
CGGACCCGACACCAUUCAUUACCUCGACACUCAAGGACCUACCACUGUGCCAGGUCCUAGAACUU
UGAGCACAGGUCAAAGUCCCCGACCGACGUCGCGACCG
>EMBL_CDS:BAG63921 BAG63921.1 Homo sapiens (human) hypothetical protein
CCAUAACAUCCACGUUGUAUACGUUCGGAUCAAGGAAAUGAUAGAAAGAGGAAAAGAAAUAAUAG
GUUUCUCCUCCUUUCAGACCCUGAACUUCGACUUGCACAUUGUGGACGUCAUAG
>EMBL_CDS:BAG35780 BAG35780.1 Homo sapiens (human) hypothetical protein
AACCCAGUCUCAAACACUCGAACUUCUCGCAAGUGAUUUACAGAGGGUGACGAGUGACGAGACUA
AGUC
>EMBL_CDS:BAG64635 BAG64635.1 Homo sapiens (human) hypothetical protein
CAUGAGACAGUGGUACUAUAAAAGUCCCGGCGCGUGUGUCUGCGGUACGUGCUUCCGGUCCACGC
ACACCCUCUGGACCGAGUACUCCGACCGACAGAGCAUCUUCCGGUCCCGCUACAGUACCACUGAU
CCCAGG
>EMBL_CDS:BAG63996 BAG63996.1 Homo sapiens (human) hypothetical protein
CCCCGAGGACCAUCGACCCGUCCUAGAACUCCUACUGUGACGGUGACCGUAACGCGUCCUCGAGG
>EMBL_CDS:BAG63418 BAG63418.1 Homo sapiens (human) hypothetical protein
GGUACGUCGAGCAAGAGGCACGCCUCGGUCAGGUCGUCGCCGAACUUCAUCGAGUUGUACCGGCU
CCGCGAGUACAACCCGACCGGGCACUAGUCGACGUACC
>EMBL_CDS:BAG36359 BAG36359.1 Homo sapiens (human) hypothetical protein
GAGGAAAAAACAGACACCAAAAUAAAGAAGACAUAGACUAUUCUGUCAAAAUCAAAGAAGUACAC
GUCAUAAUAAUCCUAGUUACUCUGAGACUCCUC
>EMBL_CDS:BAG36248 BAG36248.1 Homo sapiens (human) hypothetical protein
CCUUCCCGAAGGAGGUGACCCUAUAUUCUUACGACUUGAUACUCGAAGACAAGAAAAAGGUACCU
CUCCAAAAAGG
>EMBL_CDS:BAG65567 BAG65567.1 Homo sapiens (human) hypothetical protein
GGUCGUGGAGGGGCUAGGGAAGCAGUCUCGAGGUCGACGACGCCGCCCGCAAGACGAACUACCCC
UACCACGACC
>EMBL_CDS:BAG36687 BAG36687.1 Homo sapiens (human) hypothetical protein
CCCAGAGCCCAAGGUGGACUGAAAAGGUCAACAGACCUUCCCCGUCCACCACCAACCCCGGA
>EMBL_CDS:BAG65615 BAG65615.1 Homo sapiens (human) hypothetical

protein
UAGGCGACGUAGUAGACCCGCACGAGCCACUGGUCAACGGCGUCCAGUCGGUAGAAGACGUCGUC
GAACCCCUUCUCCAUGACCCGUAGUCCCACCAACCGGACGUCCACCUUAAGCUCCCGUGCGUCCU
ACCACAGGACCUA
>EMBL_CDS:BAG63827 BAG63827.1 Homo sapiens (human) hypothetical protein
AACGUCCCGACUUGUCCCAGGUUAUGUCAUAUCUCUCGUUAAACUGGUGUUCCUGGGACUCAAGU
CUGAACGUU
>EMBL_CDS:BAG65558 BAG65558.1 Homo sapiens (human) hypothetical protein
GGUCGAGGGGUCUCCACGACUCGAGGCGUCUCGGUAAGUCGUAGACACGUGACCCCUCGACC
>EMBL_CDS:BAG35671 BAG35671.1 Homo sapiens (human) hypothetical protein
GACGCCGACUUCAACGCGUACGUCUAGGCUGUGACCUUCCCGAAUACGGGUCACACCUAAGCCUA
CACGGCACAGUCGAGCAGUC
>EMBL_CDS:BAG65475 BAG65475.1 Homo sapiens (human) hypothetical protein
CCUACGACACGAACCCGACAGGUCCCAGAAAGAGGAGGAAGGACCUCCAGAACUCUAAGACCUUA
GAGACGUCCGCAAG
>EMBL_CDS:BAG36498 BAG36498.1 Homo sapiens (human) hypothetical protein
GCGCGCCCAACUCUGACCGGGCGACGAGCUUUCCCCGAAGCUUCAGCAACCGGGGUCUUGUCACC
CCGCAGAACAGGUCGCGC
>EMBL_CDS:BAG35884 BAG35884.1 Homo sapiens (human) hypothetical protein
AGUGUCUCGAAUUAUAAAGGUGAAGACAGGUCUUCUUAUAACCUUCCAAACUAUAGAGACACU
>EMBL_CDS:BAG36104 BAG36104.1 Homo sapiens (human) hypothetical protein
CUGCAUCUUCUCCUUUCCCAGACACGGUAUCAUGUCACAGAAUACCAGUUCCUUUGUGAACCGGU
UUUCUGUGUCCAAGACCCUUAUUGAGAACAAGAAUGCAG
>EMBL_CDS:BAG36383 BAG36383.1 Homo sapiens (human) hypothetical protein
CGUCGACGGUGUCAGAGAAGGAGUCGGGUUCCCCAACCAAAACCCAAGUAACUCAUACUCUAACA
CCCGUCAAG
>EMBL_CDS:BAG35761 BAG35761.1 Homo sapiens (human) hypothetical protein
GGACGUCAAAGACACUGAAGGAGGGGACCUAACAAGACCCGACGAAGACCCUGUUGAAGGUUUCU
ACUCCUCCCAUCUUCGACCCCC
>EMBL_CDS:BAG64651 BAG64651.1 Homo sapiens (human) hypothetical protein
CUCGUUGACCCAGAGGUACCCGAGCGACUACGACCAUGGUCCUCAUCCGGGACAGUCGUCCCAAC
CACUCGUGACACUACUAGAACCGAG
>EMBL_CDS:BAG63310 BAG63310.1 Homo sapiens (human) hypothetical protein

GCACAUCCCGGAGAGCGGUCACACCCAAGCGACCACUUUAACCUCCGAUUAUUCGACGUAC
>EMBL_CDS:CAD92497 CAD92497.1 Homo sapiens (human) pseudogene similar to part of
AGUCGCAGAACGAGAGGAGGACCAUUGUCGUCGGUCUACGAGAAGUACUCCAGCCGACGGUCCUA
CUUCAAGGACUGCGACU
>EMBL_CDS:BAG37573 BAG37573.1 Homo sapiens (human) hypothetical protein
GCCUACCGUAGGUCAUACUAUGAAGGAAGAAACGACUUAUUCUUGUCGUACCCCUAGAACUACAG
UAGAC
>EMBL_CDS:CAQ07637 CAQ07637.1 Homo sapiens (human) MAGE (Melanoma Associated Antigen)
CCACGAACUUCAGACGAGGAGAAGAACCUACGACCUCAGAAGUAGUCCACACGAGGUGCUUCAUC
UCCUCCUCCUCGUCCUCGAACCUCCUGA
>EMBL_CDS:CAI42087 CAI42087.1 Homo sapiens (human) melanoma antigen family C, 1
GACUCAAGAGAGUCUCUUUGAGACCUUUUCUGAGUCAUUUCACCUCCUCCUCCUCCCCGAGUGUC
CUUAGACCUCUCCUCUGACCCUCAGGAGGGGGAGUCCUGUUGAGACUCCUGAGAGUCUCCUUGAG
ACCUCUUCUGAGCC
>EMBL_CDS:BAG38101 BAG38101.1 Homo sapiens (human) hypothetical protein
CGUCUUCGACGUUUACGGAGAACCCUCCUUCUACUCUUUAGUCACCUGAGGGACCCCGACUUCAU
CAACGACG
>EMBL_CDS:BAG37649 BAG37649.1 Homo sapiens (human) hypothetical protein
UACAAAGUACCGGAGAAGUCGACUUUGGGAAAAGAAUCUAAGUAUGACUUCUUUAAAACAAUAGU
UGAGUUAAGGAAGUCUCAGAUUGGACCCACGCACCAAUUAAUCUCCGACACCAAAUA
>EMBL_CDS:CAQ07541 CAQ07541.1 Homo sapiens (human) MICAL-like 1
GUCACCGCUACCAUGUCGGACGGCAGUCGGUCCAUCGCGACGUGGUUCACGUGUACGACGACCGU
CCGACGCGUGCACGACGAC
>EMBL_CDS:CAQ09093 CAQ09093.1 Homo sapiens (human) structural maintenance of
AAUUGAUACAAAAGUACUACUCUGUUUCUGAGAGAAAACUGUGAGUUUAGGGAUAAGUGUACGAG
AUUGAACCACAAGUCCUCUACUUAGAAAAAGAGUAAUACCAUAUCAACU
>EMBL_CDS:BAG37533 BAG37533.1 Homo sapiens (human) hypothetical protein
CAAGACAAGGUACUUCUCAGUGCACUCAGAAGACUCAAUUUAACCGUAGGACAGGACCAAAAUUC
GGUCGUCACUCGAAGUGUAGAGACUAAACCCAGAAAAGUACCCUGACUUG
>EMBL_CDS:BAG37303 BAG37303.1 Homo sapiens (human) hypothetical protein
CAGGUGUUCUAGAGGGACCGAUGCUCUAGAGAAACUCACUGGUACUCUAGAGAGACUCACUGGUA
CUCUAGAGAGACUCACUGGUACUCUAGAGAGACUCACCGGUACUCUAGAGAGUCUG
>EMBL_CDS:CAI42601 CAI42601.1 Homo sapiens (human) melanoma antigen family C, 3
CGGAGUGGAGGGAGUCUACGGGUUCUACUUUCCGUCUUAUCAGGAAUAGACACCGGAAAAAGGCC

CCGACCCCGACUCCACCCUCG
>EMBL_CDS:CAI22918 CAI22918.2 Homo sapiens (human) meiosis inhibitor 1
ACAGACCACUUGGACGUUUGACGGGAACACGACGACCCGUCUCUUCCUCCGUCACUCGUCGGUCCGU
>EMBL_CDS:BAG36941 BAG36941.1 Homo sapiens (human) hypothetical protein
CUGGAACCACAGUUUGACACCGUCUACCACCUAUUUCUGACAUGUUGGGCGACAGGUACCAAGAUGGUCCCAG
>EMBL_CDS:CAI21838 CAI21838.1 Homo sapiens (human) RASD family, member 2
CCGCCGUGUCGAGCGGCACCAGCAAGAACAACGGUGUCUACUGGUACCCGUCGAGGCGGCGG
>EMBL_CDS:CAA15949 CAA15949.1 Homo sapiens (human) E2F transcription factor 2
GAGGUCGUCCGACAGUCAUCGGAGGUUCCCCUGGUCCCUACCUCCGCCACCCCGGACGACCCC
>EMBL_CDS:CAI22034 CAI22034.1 Homo sapiens (human) DEAH (Asp-Glu-Ala-His) box
UGGUAAAUCUCAGGAAUAACACAAACUAUUUACGAAGUAUAUAUAACUCGUAUCACUCCACCA
>EMBL_CDS:BAG37899 BAG37899.1 Homo sapiens (human) hypothetical protein
CGGAAACGGAGACGAACCCGGACUGGGAGCUCGAAGCCCCCUAUCACCUCCAACUCGUUGACCAUUUCCG
>EMBL_CDS:CAA16670 CAA16670.1 Homo sapiens (human) hypothetical protein
GGGUCCCCGUAGCCCGCAAGACCUCUCCCCGGACCGAGGACCCUCUUCGGCUCACGACCCUGUCCCGAGGACGUGUGCCUUUGAAGGUAACCGACGAGGAGUCAGGAGGGCCACAGUAAGACCC
>EMBL_CDS:CAB36861 CAB36861.1 Homo sapiens (human) POM121 membrane glycoprotein-like 2
ACUCGGGGUCCUCACCUCUGAAGAGAAGUCGCCGGGUCCGGACCCGUGUUUUCUUCACUCCCAAGGUAAAAAGUAGUCCCCGAAU
>EMBL_CDS:CAQ07960 CAQ07960.1 Homo sapiens (human) cadherin, EGF LAG seven-pass G-type
CGGGUGUCCGGACAGGAGUGACUGUGAGUGACACAUUACCCUCGAGACUUUCUGUCCGGACACCCA
>EMBL_CDS:CAI22773 CAI22773.1 Homo sapiens (human) chromosome 1 open reading frame 32
UCCCCUACCUCAUUCCCGGCCCCUGUGGCCUCUGUCUCCCUCCCAUGGGCCGGAAACGACGAAAGGGA
>EMBL_CDS:BAG36963 BAG36963.1 Homo sapiens (human) hypothetical protein
GGGUCCUCCCGGUCCAGGUGCCCCGGCUCGGGCGAGAACCCCCUCGCGAAGGAGAAAAAGACGUCCCCGAAGGACGACAGGACCGAGAAGGACCC
>EMBL_CDS:CAQ10440 CAQ10440.1 Homo sapiens (human) BAI1-associated protein 2-like 2

CCUCCAGUACCUCCAGUACCCCCAGUGCCCCCAGUACCCUAAGUGCCCGGGGAGGAGGUCUCGGA
AG
>EMBL_CDS:BAG37475 BAG37475.1 Homo sapiens (human) hypothetical protein
AGGGUCACUUUAUCUCUUGUGGUGUCAUUAUGACUAGAAACCCAUAGUUGGACUAUAGUUAAGAA
GACACACCUAUAAGAAAAAGUAGUAAAAAUGACCCU
>EMBL_CDS:BAG36854 BAG36854.1 Homo sapiens (human) hypothetical protein
UUCCUCACAAACGCCCCUCCCGGGCUCCAGCGACACAGGCCCCGUCUCGCCGGCCAAGCAGGGCU
CAGACGCGAGAAA
>EMBL_CDS:BAG37025 BAG37025.1 Homo sapiens (human) hypothetical protein
CGACUCCUGACCCUCCUAUUCAGGAGUCUUAGACCAAAAUCGAGGCGUCCGAGUAGGAAGCCCAG
GAGUCA
>EMBL_CDS:BAG37096 BAG37096.1 Homo sapiens (human) hypothetical protein
CGUCCGUUUAAUCUCCCUCCAUUUCCUCAAAGGAACUAAUGAAACUAAAAGAGAGAUUUGAGGAC
G
>EMBL_CDS:CAD92504 CAD92504.1 Homo sapiens (human) Fanconi anemia, complementation
CUCCUCGGGUGAAAGGCCGUCGCUACCGGCUUGCCGGUAGUCGUCCCUGUAGUCCAAGGAG
>EMBL_CDS:BAG38131 BAG38131.1 Homo sapiens (human) hypothetical protein
GGUCUCCGUUCCUUUCGUCCGCGAGUUGGUGGAACCCAACUGCGAACUACAGGACAUUGUAACAG
UGACC
>EMBL_CDS:CAQ10327 CAQ10327.1 Homo sapiens (human) crystallin, beta B2 (CRYBB2)
CCAUCGGUACCCGCACCUUCGACCCAUGUAGCAGUAGAUACUAAAGGUAGAAGAAGGGCCACUUC
AACCCCAAAAGUAUCUCCUACUAGAACACGAGAACCGACAGGUGAAACUACCCGG
>EMBL_CDS:BAG37516 BAG37516.1 Homo sapiens (human) hypothetical protein
GACCUCAAGUAAUCCUAAAACCAAAAGAGAAAACUCAGUAGGAGAAAUAACUCUUAUCGUAUCAA
GACCUGAAGUC
>EMBL_CDS:BAG36983 BAG36983.1 Homo sapiens (human) hypothetical protein
CGGUACAACGGGUCUUCCCCUGGUCAUGAUUGGUCACGAGUGACCAAUCCUGGUCUGCAAGGAAG
GACCCAUCGAGGACCG
>EMBL_CDS:BAG38048 BAG38048.1 Homo sapiens (human) hypothetical protein
UAGUUGUAUCGAACAAAUUAAAGACGUAUAAAUAGAAGUUCUACAAAGUACAUAAAUCACUCUUG
UAGAUCUAAGUACCUAAGAACUUCUCAGUACGGACCUUAUCCAAAACUUCCUA
>EMBL_CDS:BAG37908 BAG37908.1 Homo sapiens (human) hypothetical protein
GAAGAAGGUCAGUAGGACUCCAUCUAUAGUAUGUCCUUACGACCCCGGAAUACCGAAUGUCGUUA

GGUCAUUACUAUAUUUUCUACUAACCUCCAAUC
>EMBL_CDS:BAG37745 BAG37745.1 Homo sapiens (human) hypothetical protein
GACUCCGUGACCGACCACUGCGAGACCCCUCGGGUCCAGGACCCUCACGCUCAGCCACAGACUC
>EMBL_CDS:BAG37560 BAG37560.1 Homo sapiens (human) hypothetical protein
AUAUCUCUAGGACCAGAUUUAUGACUCCCUCUUCGAAACGGUUCUAGAACCGACAUAAAAGUGAG
UCAAGUCUUCCGAAAUAU
>EMBL_CDS:BAG37871 BAG37871.1 Homo sapiens (human) hypothetical protein
UACCCGAAAGAGAUACAACACACAAACGACUACAAAUUCCUUAACACUGAACCACGACUACCCAA
AAGAUGAAAGUCCCUUAAGUUAAAGGCAUCCGUAAACAAGUACUAAUAGUACCUCUUUUCGAGGU
A
>EMBL_CDS:CAI21422 CAI21422.1 Homo sapiens (human) guanosine monophosphate reductase
GCACUUCCACGCAAGUUCUAGGUGGAGCCGAGCCGAGAACUCCGACGAGGCGAAUCCAGCCUCGU
CCUGUAGGAACUUCAGCUCGAACUCCAGGCGUAGAUACGC
>EMBL_CDS:BAG37070 BAG37070.1 Homo sapiens (human) hypothetical protein
UAGUAAUUUCAUCGGUCGUAGUAAUCGUCGAAAGGUAGAGACGACACUGACGUCCCGUAUAAACA
GUGUAAGAAGAGACGAAUAAAACACAAAAUGUCGUUCUAAGAGACGUCAGUAACGGUACCCCUCG
ACCUGUACGACUAGACAAUUACUA
>EMBL_CDS:BAG36757 BAG36757.1 Homo sapiens (human) hypothetical protein
UCACCGUAACCCUUGGUGGUACCGACCUUAACGAGAUCGGUACUUGUCGUGACACCAAUGAGACU
CGGUGA
>EMBL_CDS:BAG37688 BAG37688.1 Homo sapiens (human) hypothetical protein
GACCACCGCGAAGACGACCGCCAUGUACGUCGUCUCCGACUCCCACUGGCUCGGCAACGUCUCCU
UCUACCGUCGGUGGUCGUCCCGGUGGUC
>EMBL_CDS:BAG37378 BAG37378.1 Homo sapiens (human) hypothetical protein
CAACCGAGUCUCUCGACCCCCUGUGUCCCGUAGAGCCCAAGAUGAAGGAGGUCCACAGACCGGUC
G
>EMBL_CDS:BAG37793 BAG37793.1 Homo sapiens (human) hypothetical protein
UGAGAAAGGUCGUUGAGAGAAACUUAAUGACAAAGGUAUUAUCACGGUUUCCGACCUUAACACCG
UUAGGACUGUCUCUAAAGUCCCAGUGACCUCUCUCA
>EMBL_CDS:CAA16432 CAA16432.1 Homo sapiens (human) paired box 7
CGACAUCGGCCACCACGACAUCCACCCACCCGUCAUCCGGACCCUCCACCCGUCCGACAGAGGAC
CGAACUACCUCAGCCGGGCGACCGACGUCG
>EMBL_CDS:BAF98780 BAF98780.1 Homo sapiens (human) hypothetical protein
ACGUAGGUGCCCACCUUCGACGGAGUCAAGUCCCAACGAUAAGUCGACCGAAUCUCUGUUAGACU

ACGACAACGUCCAAAGUGAACUACCUACGU
>EMBL_CDS:CAI23623 CAI23623.1 Homo sapiens (human) zinc finger protein 33B
UUCCGAAAAGAACACAUACCCAAGAGACUACAUGUUAAUCUCGACUGAAUACCGUCUUUCGAAA
>EMBL_CDS:BAG37550 BAG37550.1 Homo sapiens (human) hypothetical protein
GGUCCACUUUGACGAAGUCCAUUCACAACUAAUUAAGAGCUUGACUAAACUAUAGAAAACCAAGG
UAACCAGAGUUUGGACUUCAGAAACAUUGUAGACC
>EMBL_CDS:CAI42154 CAI42154.1 Homo sapiens (human) odz, odd Oz/ten-m homolog
CAUACACAUCCACCUUACAAAGAAGACGGUAUUUAAGGGUCGUCUCUCAGGAACCGAUUUCUUUA
UAACUUGAAGUCUAUA
>EMBL_CDS:BAG37064 BAG37064.1 Homo sapiens (human) hypothetical protein
UCCAAAAUGGUCAAAUUCAGGCCUUACUUGUACCAAUGUUAAUUUUAGUGUGAAGUAAAACUGUC
UUUCCCAUCUUGAA
>EMBL_CDS:BAG36994 BAG36994.1 Homo sapiens (human) hypothetical protein
UCCCGACGGAGGGUCCAUCAGCAGGUCGACACACCAUAUAUACCGACGCGUCGAAACGAGUAGGA
CCCUCCUCAGGA
>EMBL_CDS:CAQ08480 CAQ08480.1 Homo sapiens (human) kinesin family member 4A (KIF4A)
AUCCUUGGUCUGAACAUCGACAACAUGGACAACGAAAUCUACUAAUUCAAGUUGAUAGACCGCUA
GUCAUAAUUGUUGUCCAAACAAGGAC
>EMBL_CDS:CAO03520 CAO03520.2 Homo sapiens (human) novel transcript
AAGAAGUCCAUGAAGACCUGCCGCAACAACGUGUCCACCUACCGCGACAGGUCGAACAGGUCCCU
CUU
>EMBL_CDS:CAI42270 CAI42270.1 Homo sapiens (human) tubulin beta 4 (UUBB4) pseudogene
CGGGCCUUGUGCCACCGGUACCAGUCCAUCGCCGGUACAGCGCCCAACGUGUACCGGUAGUACAA
GAACCG
>EMBL_CDS:CAI42047 CAI42047.1 Homo sapiens (human) collagen, type IV, alpha 6
GACCAGCGGGCCCCAGUGGUCCGACAGGACGAUACUCACUCCCGUUCCUCUACGGACCCCGUGGC
CCCUCUGGUC
>EMBL_CDS:CAB42440 CAB42440.1 Homo sapiens (human) dJ597B2.1 (transcription factor 20
CCGAAACGUCCCCGUCGAACCACCGUCGCAAUCAUAUGACGUAGUGUACGACUCUUCCCACAACA
ACAACGACGCCAACGACGACGACGACGGGGACAACCCAG
>EMBL_CDS:CAI19007 CAI19007.2 Homo sapiens (human) novel protein
CCGGACCCCUACCAUGUGUCCAUCCUGUCAUCGAACCACUGGAGACUUUGACACGUGGUACCACG
GAGGGUCCGG
>EMBL_CDS:CAI19407 CAI19407.1 Homo sapiens (human) v-myc myelocytomatosis viral oncogene

GGGUGGUCCCGAACGUUCAUAAGGUUCCGGAACGAAUCCUAGUGAUGAAACCCCGGAACCUCGU
CGACCGGUCCCACCC
>EMBL_CDS:CAA22684 CAA22684.1 Homo sapiens (human) hypothetical protein
ACACCUUGAACUCCUAAGACAACAAAAGCAUAACCACAUACUUAAGGUUUGUAAGAAGUUUACAA
GAUAU
>EMBL_CDS:CAI21973 CAI21973.1 Homo sapiens (human) phospholipase C, beta 1
GAACGAGAACAAAAUCUCCACCUAUUUCAUAAAGUAAUUCGGCUCCUAGAGCGACGAAUUCCAAC
UAUUUUAGGUAGUAGACUAGUUGCCAUUC
>EMBL_CDS:CAB51755 CAB51755.1 Homo sapiens (human) novel C2H2 type Zinc Finger protein
UCCAGACUAACCAUUGAUUUCCGAAAAAGUGUAAGUAACGUAAAUGUUCCAAAAGAAACACUCUU
UUCAGAAACCACAAAUUAAUUCAGA
>EMBL_CDS:CAB36555 CAB36555.1 Homo sapiens (human) chromobox homolog 7
CCCGAGGGCGACGGCUCACCCGUGCAGUCCCUCUUCGUCUCGAAGAGGAACGGGAACCGGAACAC
CCUCGAGG
>EMBL_CDS:CAI21952 CAI21952.1 Homo sapiens (human) exonuclease pseudogene
AACCCACCUCAAGACACCACGUAAGAAGACUCCUACUAAGAAGAAUCUCCUAAAGAAGGCCCUCG
GGAAGAAGACCCUCACCCAACCCACCGAUAAUUAGAAACCUUCCCGUGAGGUCUAGAGGGGGUU
>EMBL_CDS:CAI20281 CAI20281.1 Homo sapiens (human) ankyrin repeat and sterile alpha
CCCCCCUGGGCAGACCUACCGAGACCGGUCGCCAUGUCUCGUCGACCUUUCCCAGGACGGGG
>EMBL_CDS:CAI42978 CAI42978.1 Homo sapiens (human) lipin 3
GAGGUACUGAACCUCCGUCCUCCCUCCGGGUACAGGGGUCUCGAGACUCAGAACGAAAGGAGACA
GAGACCCCACACCUC
>EMBL_CDS:CAB90289 CAB90289.2 Homo sapiens (human) collagen, type IV, alpha 5 (Alport
CGGAAAAAGCGGACCUCCGGGACCAACGGGUCCUACUGGAGGUGGUGGAUGUCCUGGUCCAAGAG
GACCUUCCGGCCCCAAGGGUCCUCCCG
>EMBL_CDS:CAI23620 CAI23620.1 Homo sapiens (human) poly(A) binding protein, cytoplasmic
AUGUUUGUCGUCCUCAUAGACAUCAUCGUCAUCGCCGUUGACGUCCUACACGAGGAUAACAGACA
C
>EMBL_CDS:CAI43140 CAI43140.1 Homo sapiens (human) collagen, type IV, alpha 6
UCCGGAACAACAGGGCCAUUAGGUAGGAACGGAAAUAGAGGUCCUUCGGGUGCCCCAGGUCCUUC
AGGACCAUUGGGUAAAGGACCCUCCGGUCCACCUGGUCCUACCGGA
>EMBL_CDS:CAI23618 CAI23618.1 Homo sapiens (human) pseudogene similar to part of
CCACGCGUCGGGCGCGUCCGACGGUUACCUCUACGGCUACUCGAAGACCUCGACGGACGUCCCGA
CGUCGCGG

>EMBL_CDS:CAB43181 CAB43181.1 Homo sapiens (human) FUL (ferritin, light polypeptide)
CCAGUGGGUAGAAGAACUACUCGAAGUGAAGGAGUAGAUCCUUCACUCAGAGGUCCUUCAGUGUC
UCUACCCCCAGG
>EMBL_CDS:CAB76865 CAB76865.1 Homo sapiens (human) ubiquitin-conjugating enzyme E2
UCCAAGUUCAGAAGGAAGUAGUGAGUCAAACGAAACACUGAAGUCCAUUCUGCGAACUUGGA
>EMBL_CDS:CAA21453 CAA21453.1 Homo sapiens (human) gamma-aminobutyric acid (GABA) B
GGUGUUUAAGGACCCUACAGUACAACCUUUACGACGCCCAUAACCGUCCUAAGUCGUACUGUUAG
AGUCAACACUACACC
>EMBL_CDS:CAI42323 CAI42323.1 Homo sapiens (human) melanoma antigen, family E, 1,
CCUUAUUUUUCACCUUUCUCCUUCUCCUUUACCUCCUCCGAAGGAGGAGGAGGAGAAGACACCUU
AGGACACGUCUAAGA
>EMBL_CDS:CAI42433 CAI42433.1 Homo sapiens (human) dynein, axonemal, heavy chain 8
UUUUUGUGGUCUAGUUCUCUACAAGGAGAUUACUAAAGUAACUUGACAGAGACCAUUUUGACCUC
AAAAA
>EMBL_CDS:CAI19790 CAI19790.1 Homo sapiens (human) exocyst complex component 2
CUAAAAGAGGGGACUAACACUGGAAGCAGGUACCAUAGGGAAGUAAACCUCUCUACGGCCAGUGU
UCCCCCCCAACAG
>EMBL_CDS:CAI20309 CAI20309.2 Homo sapiens (human) meningioma (disrupted in balanced
UCGCGUCGGUCCGUCGAGACCGGCUUGUCCAACAGGAACCUCGACCCGAACAACUUCCUCGGGUC
GCUCUCGCGCGGGUUAAAUGACCGGCUCCACGCGACCGACCCGA
>EMBL_CDS:CAQ07680 CAQ07680.1 Homo sapiens (human) pseudogene similar to part of novel
CCACUACCGAGACCGACGGGUCUUCGACACCCACCCCGUUCCCGACCAGAACAGGACUCCGGGAC
ACCGGGGUCCACCCGUCUCGGUCCGGUAACUCGG
>EMBL_CDS:CAA22267 CAA22267.2 Homo sapiens (human) collagen, type IV, alpha 5 (Alport
CCCAAGUGGUCCCAUUGGUCCUAGUGGUCCGUUUGGUCCUUAAGGACCAACCGGUAAAAGAGGUA
GAAACGGACCAAAUGGA
>EMBL_CDS:CAI23555 CAI23555.1 Homo sapiens (human) cadherin, EGF LAG seven-pass G-type
GGGUCCUCAUCUCCCGGCCAGUCUAGGUCCCUGAAGAACCUCGGCCAGACUCACGGGACCC
>EMBL_CDS:CAM26476 CAM26476.1 Homo sapiens (human) fumarylacetoacetate hydrolase
UCGCGAACGGGUAGUGGUGCGGGUCGAGGUCGAGCACCACGUCCAACGCUCACAUGCGCCCGUAC
UCCUACCCGCUCGGGA
>EMBL_CDS:CAI95580 CAI95580.1 Homo sapiens (human) PUUAUIVE novel protein similar to

CCCUGUCGCAAGGUCCGUCCACGUCAACCGGGGGACGUGUUUAGGCGGACGAUCACGUCGUAACC
GGUCGCAGUCCUACCGUGGACAGCCCUUGAGAAAGGG
>EMBL_CDS:CAI42184 CAI42184.1 Homo sapiens (human) chromosome 20 open reading frame 94
UAGUGAAGGUUCUCUUUGAGAGGGCACCACCAGCUUCAGAAGGCGUCUCGACGUCAAGGACGCAA
AGACAACCCAAAACCGGUGAGCACCUGAAACAAAACCUGUACCACUA
>EMBL_CDS:CAI42909 CAI42909.1 Homo sapiens (human) UUP14, U3 small nucleolar
AGAGAGAAGCUCGGAACCGGAGUAUCAUCCUGACGUCUCGGGCUCGGGAGACUUCGAGACGAGCA
GCGUAGAAACGGAGAAGAUCCGAGUACCGAGCCUCUCUCC
>EMBL_CDS:CAI42503 CAI42503.1 Homo sapiens (human) solute carrier family 13
ACAGAGGACCCGGAAGAAGGUCCAGUCGUUCCCGAGACAGAGACACAACCCUCGAAACUUCAGUU
UGGUGGUGAACUCUCUCCCGGAAACCCUGCCCUUCUUCUUGUUCUACCACUGU
>EMBL_CDS:CAC03411 CAC03411.1 Homo sapiens (human) histone 1, H2bf
CCCGUCGUCGUCCGCAUGCCGGCAGACCUAGAGGGACCUCCACUACCACCUCGCGAACAACAUUA
CGCGG
>EMBL_CDS:CAI20551 CAI20551.1 Homo sapiens (human) zinc finger CCCH-type containing 12A
GCCGACGAAGGACACGAGGUUUCACUCACCGAAGAAUGCGUCCUUCAACAGGUCCGACCCGGGCA
CGGCCGGGUCACCCAGUAGUCCCCCGUAUUUGAACAGUAACUGCUUCCUCAUGUACUCGUCGGC
>EMBL_CDS:CAM21580 CAM21580.1 Homo sapiens (human) G protein-coupled receptor 112
ACAGUCUACAGUCUUUACAACUAAAACUGUGGAGAUGACAUCUCCUUCAGUAUUGUACUCCCCGU
AAUCUAU
>EMBL_CDS:CAB51071 CAB51071.1 Homo sapiens (human) hypothetical protein
CUUACCUACCCGCAAGUCCGAGUCUCCGGGUACUUCCUCCACCCGGAGAAGGAGCUUCCGGAAAC
AAACAAA
>EMBL_CDS:CAB45738 CAB45738.1 Homo sapiens (human) hypothetical protein
CGCGAACAGGACGUCGUGUCCGAGGAAGUAGUCGACGACCUAGACCGUCGACGACUUUUCCCCGU
CAAAUGACUACUAGACGGCCUGUCACG
>EMBL_CDS:CAD92528 CAD92528.2 Homo sapiens (human) transcriptional regulating factor 1
CUCCCGCCGCCGCCCUACUUCUCUCCAAGGCCGGCCACGGCCUUGAAAAAGAAGAGGACGUGCCA
GAA
>EMBL_CDS:CAI21445 CAI21445.1 Homo sapiens (human) chromosome 6 open reading frame 94
AACAGACUCAAAACGAGGUCUUCGUCCCAGGUAUCGAUUAGGACUGAACCAACCCUGAAGUAAGC
ACCGGUGGUCCUGGGACAAGACGUCGUUUCGAGGGUGUC
>EMBL_CDS:CAO72109 CAO72109.1 Homo sapiens (human) novel transcript
CGUAGUGGACCCCUCCACCGGUUCCCACCUUCUGGACCCGGCAAUCCAGGGCCUCCUAGAAGGGA
UCGGGUGCUACCCGAGGACUACAGAGCCCACCACG

>EMBL_CDS:CAB42442 CAB42442.1 Homo sapiens (human) hypothetical protein
UUCGAAACGAAGUUCCUAGACGUACUCGACGUACCUCUAAAGUAUUUAGAGAGACAACCACAGUC
UCCGGAACUUAUCUUGUUCCAAA
>EMBL_CDS:CAI43080 CAI43080.1 Homo sapiens (human) androgen receptor
CGACGACGACGACGACGGACCCCGAUCAGAGAACGACGACGACGACGACGACGACGACGACG
ACGACGACGACGACGACGACGACGACGACGACGUCGUCGUCGUUUGACCGCGGCCCUCCACG
ACGCGAGCG
>EMBL_CDS:CAB46715 CAB46715.1 Homo sapiens (human) small nuclear ribonucleoprotein
CCGGGUCGUUCAGGACGUCCUCGGACCCCGUACCCUUGGGGUCGACCCUAAGGAGACGGUCGUCG
GGACGGCUAGGGACCCGG
>EMBL_CDS:CAB43225 CAB43225.2 Homo sapiens (human) hypothetical protein
CAUAGUAAGAGACUUGAACAAAGUGACUUUGAAAAGGUGGCAAGAAAACGAGAACUCUCCUUCUA
AAGUAUUUCAAGUCCUCACUAUA
>EMBL_CDS:CAI42283 CAI42283.1 Homo sapiens (human) spectrin repeat containing, nuclear
CACCAGGGGCCUCCAUAAAAACUAACGUAAAAGACACUCAAAAUGUAUCCGGAGUCCCGAAAGCC
CCUAGUA
>EMBL_CDS:CAI23231 CAI23231.1 Homo sapiens (human) actin, gamma pseudogene 9
CGAUCGUACUCCAUAUACGUAAACGGUCCCCGUUUAAAUAUGAAGACUUAAUUGGGUACGUCGUU
UACGAUACGUAGACGAGCG
>EMBL_CDS:CAI19089 CAI19089.1 Homo sapiens (human) novel protein
CGGAGUCCGACCGACCACUACCUUCUCGACCACAGGUCUCUGGGACGUUAGGUCAGUAUCGGGUC
CAUACCGGACUCCG
>EMBL_CDS:CAM28081 CAM28081.1 Homo sapiens (human) collagen type XII alpha 1
CACUGAGGACCCCUCGGACCCGAAGGGCCGCCGGGACCACCCGGGGACCCAGGGUAACGUGGCAG
AAAAGGUCACUUCGGAGACAGAGGACCUCCAGGGUAACCAGGACCACCCGGUCCAUUAGGACCCC
AAGGACAAGCCGGACCUUCAGGACCGAGAGGUCGUAGUG
>EMBL_CDS:CAI19192 CAI19192.1 Homo sapiens (human) REV3-like, catalytic subunit of DNA
UCACUGUCAGAUAUUUUGGUAGACACCCACAAAAUAAAAGUUUUCGAGGUGCUCACCAAAAUCAA
UUUAGUGA
>EMBL_CDS:CAI21950 CAI21950.1 Homo sapiens (human) zinc finger protein 697
CCUUCCCGAAGAGCGGCCACACGUGCGCGUCCACCAAGUGGUCCACGCUCAAUGCGGGCUUCGGG
AAGG
>EMBL_CDS:CAB46377 CAB46377.1 Homo sapiens (human) hypothetical protein
UCCGUCUACGUGCCUGUUAGAGACGGGUACGGAAGAGUCUGUCGGUAACCCCUCGACCUAACCCC
AUCCCCAAUAGGUCACGCUACGGA

>EMBL_CDS:CAQ07672 CAQ07672.1 Homo sapiens (human) chromosome 11 open reading frame 41
CGUCUCAGUAAACGUCACCGUCUCCGAUCGAAAGACGACCAGUAAAGAUACCGGGGACUAAGACG
>EMBL_CDS:CAI95767 CAI95767.2 Homo sapiens (human) collagen type IX alpha 3
GACGAGCGGAAACAUCGGUCACCCAGGGAACUUGGGACCGUAAGGUCCCCCGGGACCCCCUGGAC CGCCCGUC
>EMBL_CDS:CAI95769 CAI95769.2 Homo sapiens (human) death inducer-obliterator 1
CCCCGGAGACGACAGGGGUAUUCCUCCACCGGGUACAACGGGGCUCUUAUUCCUUCCCUACCCGG GGAAGACCCGAGGCUUAGACCGACCGCCUCCGGGA
>EMBL_CDS:CAB53713 CAB53713.1 Homo sapiens (human) hypothetical protein
AGGUUGGGACUAAGUCCCCUCCGGUACCGAAAAUGCUACCGUCGGUACAGUGACACCUACCUGUU CACCUUCAGCAGAUCCGCGAGACACAGUCCCUACCU
>EMBL_CDS:CAI22564 CAI22564.1 Homo sapiens (human) chromosome 1 open reading frame 134
CGGGUAAAAAACAACAAACAAAACAAAACAAAAACUCUAUCUCAGAGUGAGACAACGGGUCCGAC CUUCGUUCUGGGACAGAGAUUUUUUUAUUCUUCCCGUAGUCGUUCUCCCG
>EMBL_CDS:CAI21775 CAI21775.1 Homo sapiens (human) replication protein A2, 32kDa
CACCACUUGAGUAAGUAUAGGAGGUCCCCGUACUAGAAUUUCCGAUGGUCCGAGAAAAACAAGAC UUUUCUAGAGUCCACCGGACGGUGAAAGUGUAUACAAAGACCUCCUUGGUGUCACAAAAGUGACG
>EMBL_CDS:CAI19682 CAI19682.1 Homo sapiens (human) ribosomal protein S4, X-linked
GGAACCGAACAACGGUACCAAACACGGUACCAAACAACGGGAACGGUUAUUGUUUUUUACAACCU UUCGGUCCACCGUUACGACAACGGCAACCGCAGAAAGUGCACUUGGUACCGUUUUAUAGGUCCC
>EMBL_CDS:CAI22711 CAI22711.1 Homo sapiens (human) potassium voltage-gated channel,
UUCUAGAAGGGACGUUAGCAGAAUCCGUGGUACAGAGGCAUAGGGUCACACCAGUACCACUGUUA CCACAUGGUUUUGCUCCGUCCCUACGAA
>EMBL_CDS:CAI42134 CAI42134.1 Homo sapiens (human) forkhead box H1 (FOXH1) pseudogene
CUCCACAGGACGUCAUCUCACCUCUCUGGUCCGGGAUCCGAGACACCUCUCCCAGCUCCCGGGUC ACCGAGGGACGACCCUUCGGAG
>EMBL_CDS:CAB81612 CAB81612.1 Homo sapiens (human) EPH receptor A8
CCCCCCACUACGUCUCCGUCCUUCUGUCCACCCCCACGGACAGGUAAGACUAUCACGUAGAAGAG GAG
>EMBL_CDS:CAB43361 CAB43361.1 Homo sapiens (human) hypothetical protein
GGAGAGGGGGUAUAGGUCAGGGGGGCCUCCGGAGAAGACGUGGGGGUGAGUCGCCCCUCGGUGUC CUCCGACUCGCCGCCACUGGAGCUCUUCCGGAUCCCCUCCUCCGACCACCCCUCUCC
>EMBL_CDS:CAQ10064 CAQ10064.1 Homo sapiens (human) cyclic AMP phosphoprotein (ARPP-19)

UUCCUUCGACCCGGGGGUCCGGAAACAGGAUCCGAACCUAUAAACCGGAAAUCGAAAAGGAA
>EMBL_CDS:CAC00585 CAC00585.2 Homo sapiens (human)
UUCUCUCGGGGCAGGAGUCCUAGUAACGACAUCAGUAGGUGGUUCUACUAAAGAAAAUACUCUAC
CCCGCCCGAAAAGAA
>EMBL_CDS:CAQ76415 CAQ76415.1 Homo sapiens (human) olfactory receptor, family 2,
GAUUACCAGAAAAGACCAGGGGCCUCCAACUGGUCGUAGACACCCUGUCACGACCACCAUAUCGU
CUCCAGGUCUUUCCUAUCUAAUC
>EMBL_CDS:CAI20272 CAI20272.1 Homo sapiens (human) arginase, type II (ARG2) pseudogene
AGAGGAUCUAGUCUUAUGCGAUAUGAACCCCUCCUCUAUGUUCCAAACAGGUCCCUUUAGGACCU
UCAAGACCAUGGAAAGGGACAUCAAGACACUCCUCU
>EMBL_CDS:CAB81608 CAB81608.2 Homo sapiens (human) rearranged L-myc fusion
UCUACUAAGGUUUAGACAAAGUCUUGAUGUAAGUAGGAAAAAUCCUACUGUCACACCAAAGAGUC
UCAAUGAUGACCCUAGUGGA
>EMBL_CDS:CAI43017 CAI43017.1 Homo sapiens (human) transient receptor potential cation
CCCCGACCCCUACUACAACUUUCCUCCACCGUUCCACGGUGGAAGUAGUUUCAUUGAGUAGGUCU
CGAAG
>EMBL_CDS:CAB43293 CAB43293.1 Homo sapiens (human) hypothetical protein
CGCCGACGGCUGAGUCGGUCCCUGAGAAAGAGUCCCCACCGGAGGACCCUGCUCCUGAGUCUGGG
ACUGAGCCGUCGACG
>EMBL_CDS:CAB92770 CAB92770.1 Homo sapiens (human) glutathione S-transferase A2
UUGAAGAAGUGACACCCGUCCAAUGACUAAGACCAAAAGUCCCGGAAGUCGUCUCCCUUCGACCU
UUAUUCCGAUCUCAGUUCGAGAAGGUGCAUCAUCUCUUCAA
>EMBL_CDS:CAB45747 CAB45747.2 Homo sapiens (human) hypothetical protein
AACGAAGUUCUCCACCUCCUCGAACACACCCUUGGGGUGGUUCACAGACAUUUAUACCUUCGUC
>EMBL_CDS:CAB65797 CAB65797.1 Homo sapiens (human) olfactory receptor, family 5,
CUUUCUUCUACCACUUAUCAUUUUUGACGUUAAGUAAAUCCAACCUCUUAGGGUUCUACUACUUA
AGUCAAUAUCGACAAACUAAGAAAGAAAG
>EMBL_CDS:CAI22044 CAI22044.1 Homo sapiens (human) dystonin
UGAUAGUCACAGACAAGGACACAAUAAGACGACUAGUAAGUCGUCACUGCAAAGUAUCAGUGAUC
GUGACUACCA
>EMBL_CDS:CAI23037 CAI23037.1 Homo sapiens (human) E1A binding protein p300
CCGGACGACGGAAUGGGACCCUGUGUCCCGGUCGUCGAACUCAGGACCCGUUCAUCCCACCGUAC
GA
>EMBL_CDS:CAO03467 CAO03467.1 Homo sapiens (human) myo-inositol oxygenase

CUGGACUGCACAUCAAGGCCUUCGACCGGAACAGAAACCGGUGGAGACCCAGGUGUAGUCCAG
>EMBL_CDS:CAI43095 CAI43095.1 Homo sapiens (human) zinc finger protein 64 homolog
ACCGACCUCAACGCCGACCGCAUCCCCGUCUAGACCGUAAACUUCCCGGCGAGCAGGCUCACCUA
GGAGUCCACGAACAACUCCGACGACGACAGCCGUCGCCGCAUCAGU
>EMBL_CDS:CAI22113 CAI22113.1 Homo sapiens (human) sex comb on midleg homolog 1
UCGACAGAACCUACCCCGUCUCUACCCUACCCUUUAAUCCCAGAACCCACAGGCCGGGGCGAAGA
AAGGACCAAAUGCGGGGGAGACGGGACCAACUACAAGGUUCUGUCAA
>EMBL_CDS:CAI43016 CAI43016.2 Homo sapiens (human) chromosome X open reading frame 45
CUACUUAAGGACCCGUAGGGUAUAGGGGCUGGAACAGAGCCUCUACCUACGACUCCUACUUUUAC
AAGUAA
>EMBL_CDS:CAI19918 CAI19918.1 Homo sapiens (human) KH domain containing, RNA binding,
CCACCACCCCCUCCUCCUUACCGUGGGGGUGCCGGGGAACUUCCUCGACACCCUCGAUAAGACUA
GGGAGACGGAGA
>EMBL_CDS:CAC33877 CAC33877.1 Homo sapiens (human) zinc finger, NFX1-type containing 1
CAACUUCAAGAACCGGUCUAGUAUGUGCAACCGUGACCCCGCGUCGACGACCACCAGGGGUUAGU
UUUACUCCACGACCGUUCGAAACGAGUUACACCGUUACCAUACCCGGAGUUCCUGAAGGCG
>EMBL_CDS:CAB88112 CAB88112.1 Homo sapiens (human) chromosome 6 open reading frame 72
UCUAUGACCGAUCACCAAACGUCACUUGAUUGUGAAAGAAAAAACCUCUCCAAUCCUUCCCAUUU
AUUUUACAGUGACAGA
>EMBL_CDS:CAB59178 CAB59178.1 Homo sapiens (human) hypothetical protein
CUAGGGACCCGAGGACUCUCGGUGACCCGACCCCAACCUGUACUCGUACAGGGAAGAAUUGGGCC
GGGAAGAAUGAGUCGAACCGGACCCCAG
>EMBL_CDS:CAI19914 CAI19914.2 Homo sapiens (human) soluble adenylyl cyclase (SAC)
GAAGAACAUCGGAUUGAAACAGAAGAAGAGAAACCAAGGUAGUUACUACAGCUAUCUCCUUCCGU
AAUUAUACCCGAGUCGUCCUUC
>EMBL_CDS:CAB55978 CAB55978.1 Homo sapiens (human) hypothetical protein
UGCUUAUUUAGAAAUUGAAGAUCUCCACCCAACGUGAAAAGAUCUACAUCUAAAUAGGUAGCA
>EMBL_CDS:CAI40128 CAI40128.1 Homo sapiens (human) actin filament associated protein
ACAUUCACAACCCGAGACUCUUGAAAGAGACGUAGUCAUUCUCUCUCAAAAACCGGCGUGAGUGU
>EMBL_CDS:CAB55985 CAB55985.3 Homo sapiens (human) hypothetical protein
GACCGUGACCAUCGUCCCCAUCCCCUCCGUGGUGUCCUGAGAAGGGGUCGUCGACGGAGACGAAA
GAGGCCCGGUC
>EMBL_CDS:CAB96723 CAB96723.2 Homo sapiens (human) CD83 molecule

AGUCCCUUAUCCCGGAAAGUAACCCCCGCAGCUUUCUUGGUAAAACGGGGAAGACUACUAUCACG
ACAGGGGACU
>EMBL_CDS:CAI22736 CAI22736.1 Homo sapiens (human) sperm associated antigen 17
UCUUUAGAGACCAAAACCUUCUGAUCCGUGUGAACUCCUAAAGACAAGACUUUGGAAUACACCGA
ACUUUAGAUCUAAUUCUAGUCUACAUAGA
>EMBL_CDS:CAB55920 CAB55920.1 Homo sapiens (human) hypothetical protein
GGGUCCCCCACCGUACGGGUGGACCUUCCGGACGUGCAUGUCGUCCUACGACUCCCGACCCCGGA
AGAGACCGAGUCGGUAGAAAACCC
>EMBL_CDS:CAH10712 CAH10712.1 Homo sapiens (human) hypothetical protein
GAACCGGUUCUAUAUGAGGGAACGUCACCGGUCCGACGAGGAAACGACUCGGUAUCUGGUGACGA
ACAUCCAUAACCGGUCC
>EMBL_CDS:CAI19330 CAI19330.1 Homo sapiens (human) chromosome 20 open reading frame 32
UACCCCCCGUGCCGGACAGACGUCGCUGGAGGCACUCCUAAACCUCCGCCAACCGUCCCCGGUCC
GGAACGGAGGGUA
>EMBL_CDS:CAB57341 CAB57341.1 Homo sapiens (human) hypothetical protein
CCCGCGACUCCUGUCUACCCGACUGUAUCUUCGGUACCAAUCCUAGACCGCACAUGAGCCAGGUG
AAGUCGUCGCGGG
>EMBL_CDS:CAI21960 CAI21960.1 Homo sapiens (human) fatty acid amide hydrolase
CACAUCGACUGCCGGGGACACCGGGACGGACCCCGUAAGUUCAGGUCUCGUCCCCGGUCGUACCC
CCAGUCGUGGUG
>EMBL_CDS:CAB56033 CAB56033.1 Homo sapiens (human) hypothetical protein
GGUCCAUACCCCGACAACCCGGACCAAGUCCCCUGGGAUGAGGGGCCGGGGAAUCGGGUAGACC
>EMBL_CDS:CAH72269 CAH72269.1 Homo sapiens (human) chromosome 20 open reading frame 59
CUACCCGUACGACCUCGCCCGCGUCAUGUCUUCCGUACACGGGUCGUCGUCGCAGGGGCAG
>EMBL_CDS:CAB81623 CAB81623.1 Homo sapiens (human) novel protein
AACCGAAAAAGUGAGUCGUUUGAGCACUCUGACCCCUCCAGGUCCCGCGGACCUAACGAAUCACG
GAGAAGAAAGAGAUCCCGACUCGACACUUUUCCGACC
>EMBL_CDS:CAM28319 CAM28319.1 Homo sapiens (human) bone morphogenetic protein 7
CUCCCAUGCCGACAGCUCGUCCUUCUCUAGGCUAAGGGACGGUACAGGAGAGGAGACGACCUUGC
ACUCGAAGGUCUCCCGUCCCUGAGCACAGACCAAGCAAGACCAGCUACGGGAG
>EMBL_CDS:CAI12515 CAI12515.1 Homo sapiens (human) golgi-specific brefeldin A resistance
AGUCAUUCCAGAAGGAACCUUCACCAAACCCUCCUCUUAAGGUCCGGUGACAGACAUCUCCCUGA
CUGGUGACGACU
>EMBL_CDS:CAI22343 CAI22343.1 Homo sapiens (human) chromosome 1 open

reading frame 164
ACACGUCCCCUACUGUAGUCGGAGACACAUAACACCGAAGGGUAUGAGGUCUCACAGUAGGGGUA
GGUGU
>EMBL_CDS:CAC44024 CAC44024.1 Homo sapiens (human) SLC2A4 regulator
CCGUCACCCCCACUGCCCCACCUGCCUCUCCUGACCAGUGACCGGUCCAGGGUAGGGGUCAGAGG
UGACAACGACGACGACGA
>EMBL_CDS:CAB55896 CAB55896.1 Homo sapiens (human) hypothetical protein
CCGUGGGAACCUCCUCAGACGUCCUCACUCCCCGGAGACGACGAGAGACUCCGACAGUCGCGAAC
GUCCCUCCUCACCUCCGAGAGUGUUUACCCAGACCGAGAAGGUCACGG
>EMBL_CDS:CAI15269 CAI15269.1 Homo sapiens (human) immunoglobulin superfamily, member 8
CCGGCCCUUGCCCGUGACCGACGAAGUGCUUCGGCCCAGGGUCUGGGAGCUUGUAUCCGAAACCG
CUCCGUCGCCAUCCACGGGCGUAGUGGUCCGG
>EMBL_CDS:CAI14947 CAI14947.1 Homo sapiens (human) zinc finger protein 37A
UCCAGAAGGGUGUAAGUACUGUAAGUAUCCCAAAAGGGGGACACACACAAGAGACUACAGAACAC
ACCCGACUGAA
>EMBL_CDS:CAQ09650 CAQ09650.1 Homo sapiens (human) poly(A)-binding protein pseudogene
CACCAUUCAUUGGACUUCCUCUCCACAGGACAUCUUUAACGUCCCACCUGUGUGGAGAGUGAAGA
CAAUAAAAGACG
>EMBL_CDS:CAQ10609 CAQ10609.1 Homo sapiens (human) par-6 partitioning defective 6
UCAAACCAAGUUAGACGACACCAAUCCGUUCUUCCGAUAAUAGUCGUCUAACCGACCUCGA
>EMBL_CDS:CAI17853 CAI17853.1 Homo sapiens (human) hemicentin 1
CUUCAUAACUCGUCAUGUAUCCAUCCAUAAGGAACGUGACCAGACGACUGACAAAAAAACUUCAG
GUCCCACCCGAAAGCUCUUUUCGGUCGAGCCUUUAGUUAUCAUGACUCCGGUCAUUACGCUUGUC
UCACGGAAGGUGACGAACUCCAGAAG
>EMBL_CDS:CAB99331 CAB99331.2 Homo sapiens (human) collagen type XIX alpha 1
CCCCUUGGCACUUGUGGACCUUCAGGAGAAAGUGGGAAACCGGGUGCACCUGGUCCACGAGGUCC
UAAAGGUAGAUUAGGACCAAGGGG
>EMBL_CDS:CAM13070 CAM13070.1 Homo sapiens (human) shroom family member 4
CGGAGUAACUCACCCAACCUUAGGUGCGACCUUCGAGACAGUGUCCGAAAACCCCUAGGUUGUCG
AAACCAACUCCG
>EMBL_CDS:CAI16056 CAI16056.1 Homo sapiens (human) chromosome 9 open reading frame
AAGUAAAUUUCCAUCGUGUAAUCCGUUACGAAGUUAUCAUCCGAAGUUUCACGAUGAAAAAUUCC
UU
>EMBL_CDS:CAI23423 CAI23423.1 Homo sapiens (human) leucine rich repeat containing 7
GACUCUACUAAAGAAGAAGUGGAGCCUUUGUAGCCGUACCGUGGUCUGCCGGCUACUAAAAGGCA

AACCACCAGUAGAGGUC
>EMBL_CDS:CAH61508 CAH61508.1 Homo sapiens (human) ribosomal protein L12 pseudogene 3
CACGAAUUACACGAAGACGAAAAGAGACAGACUCACCAGGAAACUGUCGAAACUACCAGUUCCGU
CUCCGUCUUCCAUG
>EMBL_CDS:CAI22170 CAI22170.1 Homo sapiens (human) chloride channel, calcium
GACACUUGACGAACCACAUACCAAGUAGAGAUGGACCCUCACAUCCACCACGUUGAUAUUGUAGU
CGUACAAAUACC
>EMBL_CDS:CAC35071 CAC35071.1 Homo sapiens (human) novel protein similar to serine
UAGUCGGGAUAGCAAUAGUUUAGGACUCUUCACCUAGUAAGGUGGUCCGUAAAAGAACUAGCAAC
CCGACUA
>EMBL_CDS:CAB55911 CAB55911.1 Homo sapiens (human) hypothetical protein
AAGGCCAAAGUGAACUCCCUCCGGAAAAACAGCUGCAAGAGAGAGAAAAAGAGCUAGGGCAGAAA
GAACUCUAGCUCUAGCCCCUCUUACAGCCGCAGAUAGGGACCUGGCUCUAGCUCUU
>EMBL_CDS:CAQ07060 CAQ07060.1 Homo sapiens (human) staufen RNA binding protein homolog
GCCGGAACUGAUAACAAAAUCCCGCAAAAAAUUUUUUAUUAAAACGGAAAAGGUGGUGUCCUUCU
CCACCUUCAAAAAAUUCGAGGACAUUCUGCCACCAACGUCGUGC
>EMBL_CDS:CAH70743 CAH70743.2 Homo sapiens (human) centlein, centrosomal protein
GCCCUCUUUGGGAACAACCGUCUAUACAAAAGACUGUCAAAAAGUAAACGAUAGAAAUCAAAGAG
GAC
>EMBL_CDS:CAC40711 CAC40711.1 Homo sapiens (human) 1060P11.4.4 (killer cell
AGUGAGCCCAGACUGGUGAGCAUCCCUCUAGGUACCUUUCUCGGCUUCGUAGACAUCCAGAGAGG
CACCCACCGUCCUGGGUCUCCCU
>EMBL_CDS:CAI19614 CAI19614.1 Homo sapiens (human) ADP-ribosylation factor guanine
CCCACCUUAGGUCCGCGAGGAGGACCACGUCCUUAACCCGAUACAGAAGUUGACUGCAAGGGUCG
UACGGGACGAGGACCUCUUUGACCUAGGGGG
>EMBL_CDS:CAH71770 CAH71770.1 Homo sapiens (human) transcription factor 7-like 2
CCUCCUCGACAAAACUAAGCAAAGACUAAGUAACUGAUCUCUGCUAAACUGUAGUCGAUUUAGG
>EMBL_CDS:CAI14921 CAI14921.1 Homo sapiens (human) zinc finger, CCHC domain containing 6
CGACAACAUUAUCCAUUCUACAAAGAGUCUGGACUUACAAAAGACUCCUGAGAACGAUUAAGAAG
UUAUCA
>EMBL_CDS:CAI19272 CAI19272.1 Homo sapiens (human) ubiquitin protein ligase E3 component
GGAACAACCAGGACGACGUCCGAGUCCCGUUACCGAUCAAGUUGGUACCAGAGCAGUAGACGUAG
UCCACCCUACAGGUCGUACCCCCCCUUCGGGAGACGCGGACGGUCGUCCCGGAGGUCCC

>EMBL_CDS:CAI16208 CAI16208.1 Homo sapiens (human) nucleolar protein 8
CCAACACCGUCAUAAAACUCCCUAGUAUAAACUACUAAAAUGCAAAUACAACUAAAAAUUCCACU
UCUUGUCCAUUCUGAGAAGGUUUAAACGAGUGUUGG
>EMBL_CDS:CAI12398 CAI12398.1 Homo sapiens (human) collagen type XVII alpha 1
CAGUCGUAACAUCCGUCGAACGAAAAGAAAAAAAUCCCACAGAAGACUUUUUCUUCAACGUCGCU
ACGACCG
>EMBL_CDS:CAI20000 CAI20000.2 Homo sapiens (human) chromosome 6 open reading frame 170
ACAGAAACCGUCAAAAGAUAGGUAAGUCAAACAAAUAGUCUAUAAAACUCCGUAUUAUUUUCGAA
CAGUUCUAGUAACAGAACAAAAUAUGGUCGUAAAGAACAUUACAGACUGUCUAUCGUCAAACCGU
UACCUAUACUACUUUUGUAACCGGUUCCUAU
>EMBL_CDS:CAB70833 CAB70833.1 Homo sapiens (human) hypothetical protein
CCGAACACCCGAGGGCUCGGAGGACACCCCUGGUGACCCUGAAAGGUAAGACCCUCCGGGGUCAC
ACUAGGGACAGUUAUCCGAACCCUGGUAACCCGG
>EMBL_CDS:CAM17621 CAM17621.1 Homo sapiens (human) potassium channel regulator
AUGGUCGGACUGAGGUCAAACCAAUAGUCAUCACGACAGUCUUGGUGUGACCUUUUGGUCCAGUA
CCAU
>EMBL_CDS:CAB70855 CAB70855.1 Homo sapiens (human) hypothetical protein
GGUUAUAGUAACAGGUAUAGUAGACCCUUACCUUCCAGUUGUUUGUAUAGUAGUAACAACCCCGA
UUUGGGUAGAAGUAACCCCAUCAACCGACCUCGGUGGACCUACCAACUACUACAACC
>EMBL_CDS:CAB63722 CAB63722.1 Homo sapiens (human) hypothetical protein
ACCCGGGUUCCUGUCACGAAUGUCACCGCUCCCCAGUCCUACAGUGAACGAGAAGCGGGACGGAG
ACCGACUAGGACCCCUGGGC
>EMBL_CDS:CAI39480 CAI39480.1 Homo sapiens (human) LIM domain 7
AACACUAGCGGAAAAAUUCAAAAAGUUUCUUUACCUAAGUCUACGAAAACUAAGUUUUCGUAACG
AAAGUAGUACUUAAGGUAACUAAAGAAACCUAAGUUAUAAUCUUGAGACUUCCGAAAGCCUC
>EMBL_CDS:CAH73836 CAH73836.1 Homo sapiens (human) chromosome 10 open reading frame 4
UCUCAAAGAUUCAUCUCUUAGAAGUCUAAAGAAACUUCUACUUACAGGAAAUAGAAAAAAGAACC
UCCGGAGAAGACGUCUUCUAUUAGACCUAAAAAAUACACUACUGAGA
>EMBL_CDS:CAH71682 CAH71682.2 Homo sapiens (human) chromosome 13 open reading frame 18
GAGACAGGUCGAGGAACCGAAAGCGUAUGUCCGAAACCGGCUACGAGUCGUUUAACUUUUACCCG
ACCACGGUAUACGACAGCUCGUCGACAAACCUC
>EMBL_CDS:CAH73304 CAH73304.1 Homo sapiens (human) dachshund homolog 1 (Drosophila)
GGAGGAGGUCUCUCCCCCGUCCACUCCCCGAUAGUCCUUGUGCGAGGAAUUAUUGACUACAGAGU
UGAGACCUACCCCC

>EMBL_CDS:CAI23362 CAI23362.1 Homo sapiens (human) zinc finger protein 644
GAGAUGUUCGUAUACCGGAACCCUGUAAUUCUACCGCUUUCACUGAUAAGGGUAGAUUUACUAUG
UAGUAUACGGACACCUC
>EMBL_CDS:CAI20328 CAI20328.1 Homo sapiens (human) novel protein containing CH (Calponin
ACGACUUAGGUUACUCCAUUUAAAGCUUUUUUUAGGAAAAACCGUAAAAGACUAACUAACUCGU
>EMBL_CDS:CAI12722 CAI12722.1 Homo sapiens (human) ash1 (absent, small, or
ACGCUUCCUCUUAAACCGUUGUUUCAUCCUCCUCUAUUGGCGGAGGAGGGGAAACUCCGGAAGAA
GUAACGU
>EMBL_CDS:CAB66599 CAB66599.1 Homo sapiens (human) hypothetical protein
UCCUGUCGGAAAAGAGGUAGAUUAAAACAAAACGGUAUGUUUAAAUCCCCUCGACCAUACAGAA
>EMBL_CDS:CAH70098 CAH70098.1 Homo sapiens (human) haloacid dehalogenase-like
AUACGGACGGACUUCGGAACAAGGUCCCGACUCCCGAGGUGGAGGUCGGGUACCCGGGCCCGGAA
CCACCGUAU
>EMBL_CDS:CAQ10575 CAQ10575.1 Homo sapiens (human) capicua homolog (Drosophila) (CIC)
GGGACUCCCCGGGUCCCGAGGAGGAGGUACCACCCUCCACUCGAAAAUGGUCCAAGGUGGUGGGG
GUUUCACACACCCCAACGCCCGGGACCCGAAAGUCCC
>EMBL_CDS:CAI23173 CAI23173.1 Homo sapiens (human) centaurin, beta 5
CGAGCCGACGGUCGAAGAAGUACAUCCCCAGGUCGACCACGUCCUCCGACAUCGGGACGACCUUC
UUCGACCUGACCCG
>EMBL_CDS:CAB63754 CAB63754.1 Homo sapiens (human) hypothetical protein
CCCCUCUUUAGUCACAGGUCAGACCGAGUCCCGAACCACCUGACCUAUCGCUUUCGAGGGG
>EMBL_CDS:CAI95132 CAI95132.1 Homo sapiens (human) transforming, acidic coiled-coil
CCCCAAUGGAGGGGACGGAGGCCCCGGCACCAUUCUCCGUCGCAAAAGGAGGGCUGUCCCCCACU
CAAGACGUGAGACGGUGGAGGUCUUCGGCCCCCCUGUUGGGG
>EMBL_CDS:CAD13328 CAD13328.1 Homo sapiens (human) tyrosinase-related protein 1
ACUUCCACACGUCCUCCUGUUUUUAUCCUAGUAAACCUCUGUUUACCCAAACAGGGGGACAAGGU
AAGU
>EMBL_CDS:CAI12716 CAI12716.2 Homo sapiens (human) ash1 (absent, small, or
AUCAUGAAUCCGAAAGUCGUUGAACCGUCACAAACAGACUACGUCUAUGACGUGGGUCAAAAUUU
UUUCCGAAUAAUGAU
>EMBL_CDS:CAB66750 CAB66750.1 Homo sapiens (human) hypothetical protein
GGUCCCGAGGAUCCUUCUAGACGGAGUCCACCUCCCGGGAACUCACACCUCCGAAGGCCCCCUGA

CC
>EMBL_CDS:CAI14765 CAI14765.1 Homo sapiens (human) MAP kinase interacting
GACAUCAGGGUCGAUGAACCCUCCGACUCCACCCUCCUAGUGACCUCGGGUCCCUCCAACCCCGA
CGUC
>EMBL_CDS:CAH73308 CAH73308.1 Homo sapiens (human) novel transcript
CCGGACCACAAUUAGAAGAACCUGCUUAGUUUACUCGGAAUACAAACAGUAAUACGAAUAAUUUU
UACACAGCCUCCUCUAAGCUGGCCCAA
>EMBL_CDS:CAI41479 CAI41479.1 Homo sapiens (human) ubiquitously transcribed
CCGUUCCCUCCCAUACCACUUCUACCACCAGAACCUCCACCUGUAAAUAGGUUGUUUUACGAUGA
CAAUCUAUUCGGUAAAAAUGGAUCUAAGGACGUACGG
>EMBL_CDS:CAI15524 CAI15524.1 Homo sapiens (human) Smg-5 homolog, nonsense mediated mRNA
GGACGUCGGGAUCCACCCCGGACGAGGGACGGGACCUUCGAUCUGUCUCCCUCUGUGUUUCGUCU
UCACCCCGUUACCCUACACGUCGGGGACGAAAACCCGACAUCC
>EMBL_CDS:CAH71353 CAH71353.1 Homo sapiens (human) calcium channel, voltage-dependent,
GGUGUAGUAUGCCUCCUCACACGAGACAGAAGAAUUUCUUCCCGUAAGAAAAGAGAAACCUCACC
>EMBL_CDS:CAB70793 CAB70793.1 Homo sapiens (human) hypothetical protein
AAGAAAGGACGGGAGCCACCGUGCGACGUAGGCCGGCUACCAAACCAAGUCCUACCCCACCCGUG
GGUCCUCUCUU
>EMBL_CDS:CAB70826 CAB70826.1 Homo sapiens (human) hypothetical protein
CCAGAACCGUACCUCCUCUUCUCGACGACGUCCCGGAGAGCCCCCUCCCCAACCGGUUCAUCCGU
AAGUGGUCGACGUACUAGAGAAGGUGGACCCCCGAGACGUCCUCCUCGACCGAGAGAGGGUGGGA
GUUCCGG
>EMBL_CDS:CAI19076 CAI19076.1 Homo sapiens (human) DNA cross-link repair 1B (PSO2
CUUUCCGUGACGGAACAAGGUAAGAAACGAUAUCUCUAGACCCUUGUUGACGAAGAACUAGGCGU
UUCCUACCACCCUUCCGACGAAAAG
>EMBL_CDS:CAI22995 CAI22995.1 Homo sapiens (human) dedicator of cytokinesis 7
CUCCCAUAUACAUCUCUUCUAAAGACAUUCAUCACAAACCUUGUAUUUGUAGGGAACGCCCUUUG
AGAUUCUAAAGAG
>EMBL_CDS:CAI41083 CAI41083.1 Homo sapiens (human) interleukin 15 receptor, alpha
CGACGACAACACCGGCGACACAACAAACUCGACCCUCUACUUCGACGCCCGAGGGCGCAGCGGCC
GCCGGCCUCGUCGUCG
>EMBL_CDS:CAB66695 CAB66695.1 Homo sapiens (human) hypothetical protein
CGCCCGAUAGACCCGGGCGUCCAUCAACCGGAGCAACGGGCCCUUCCCACCCAUCGACUGCUGAC
CGCACAGGUCCAUCGGACA

>EMBL_CDS:CAH72822 CAH72822.1 Homo sapiens (human) polycystic kidney disease 2-like 1
GACAUACGGCAGAGGACCCCAAGUAGAAGUCCGAAGAACCCAACCCCGACCUCUCCGGGGCACGA
CCUCUACCACGUC
>EMBL_CDS:CAH70925 CAH70925.1 Homo sapiens (human) v-abl Abelson murine leukemia viral
CCCACUCUAUUCCUGACCGUGAAACACCAACACUCACCAACCCUCCUGCCCCCGUCGGAACCGAC
CUCUUCCGGUCGGGCCGACGAGAGGUAGGAGACCUUGAGGUCGGUAGGGUUCAGCACGGGGUGGU
AAGAAGAGAAAUGGG
>EMBL_CDS:CAI19765 CAI19765.1 Homo sapiens (human) small proline-rich protein 2C
GACGACUCCACCCGUCCCGACACCUGUGAAACCACCACCCGUCCCGAGUCCCGUGAAGUCCCCAC
CUGUACCGAGACCUGCGAAACCGUC
>EMBL_CDS:CAB66751 CAB66751.3 Homo sapiens (human) hypothetical protein
UACAUACAGUUGGUACUGACGUAAGACGACCCCACCUACUCGUCGUCCUACAUCGUCUGCCCCUC
UAUGUA
>EMBL_CDS:CAI23384 CAI23384.1 Homo sapiens (human) chromosome 1 open reading frame 84
AAGGACAAGUCUGACUUCGUCGUAGAGGACCCCACACCGGACGUGUCAAACCACGUCUACUGACU
CCUUGGUUCGGUCCGCUUGUAAAGCCCUCUAAGCUAUUAGGAAAAAGUAAUUGUCCUU
>EMBL_CDS:CAQ52599 CAQ52599.1 Homo sapiens (human) spastic ataxia of
GGAUUUUCUGAGUUUGUUGAGUUCUUGGAAGACGAAUAACAUCUCCAAUGAUGAUUCAACACUUC
CAACAUCC
>EMBL_CDS:CAB66709 CAB66709.1 Homo sapiens (human) hypothetical protein
GAGGGAAUCGACUACCUCUGACUUGAAGUCGUCCUCGGAGAAGACGUCUUCAGAGGAGUCUAUCA
CCUC
>EMBL_CDS:CAB66530 CAB66530.1 Homo sapiens (human) hypothetical protein
CCAGGACGAGGACAAACCUCGACGACGACGCGUCCGACGCGGCCUACUACGAGUCGUCGCCGAGG
UCGUCGAGGUCCUGG
>EMBL_CDS:CAI20013 CAI20013.1 Homo sapiens (human) molybdenum cofactor synthesis 1
AGCGUCCACUAGGGCGUCCCUAUGGAGUCUCAAAGGUUUCUCCGUCUGGAACUCCAAGGGUAGUC
GACACU
>EMBL_CDS:CAI16092 CAI16092.1 Homo sapiens (human) potassium channel, subfamily U,
ACGACUUAAGAGAAGAAACCAUUAUAAUUAUAUUUUCGUAUACAGACAUCUUAAGUAUUACAUAG
CUCCUGGACCUAAGUCGU
>EMBL_CDS:CAI15169 CAI15169.1 Homo sapiens (human) POU class 2 homeobox 1
CGGACACGGACACGGACGACCUCAUCAUUGACAACGACCCGUCCGCAGUUUCAUUCGGGUCAAUA
GACAGGAGGUCGAUCGUAUUCGACCCA

>EMBL_CDS:CAB66825 CAB66825.1 Homo sapiens (human) hypothetical protein
CCGCUCAAGUAGGUCCCUCGGCUUCCACCACCCCCGGCGCUCGAGGUUCCGACAGACCCUCUUGA
ACGG
>EMBL_CDS:CAC19507 CAC19507.1 Homo sapiens (human) novel protein
GUCGUAGAGGAGACGGACCCCUCUUCCAAAAGCCAUUUUCACGAAUUCGACAAGUAUCCCGUCUC
CCUCGACGAC
>EMBL_CDS:CAI22864 CAI22864.1 Homo sapiens (human) Fc receptor-like 5
UACCAUGGUAAAACAAAAGACACCACUCAUCUUCGCUUUAGGGAACGUUCACUCCCAGUGAGAGA
GAGGAACCUUCUGACACCAGGUA
>EMBL_CDS:CAI95133 CAI95133.1 Homo sapiens (human) novel protein
CGGUCCCGAAAGUCCAUCUCGAACCGGUUCCAGAGUCUGACUUUUUCGACCCCGACUACCUCGAG
GGUAAACUUCGGGUCCG
>EMBL_CDS:CAB63721 CAB63721.2 Homo sapiens (human) hypothetical protein
GACCAUAAGGACUCACUCCACCCUCGCGACCCAGUGUGUCAUGUGCAUAGAGAGGUCCUGUAUGG
UC
>EMBL_CDS:CAI23448 CAI23448.2 Homo sapiens (human) hypothetical protein
UCGUCCUGUGUACCAUCAUGAUGGUCUGGUCUACCCUACACAACACAACCGUAUAUCUAGAAAAG
AUUAUAUUCGUAGUGUAGAGUACCCACUGUACCUUCACGAUAGAACUCACGGUGACGA
>EMBL_CDS:CAB70899 CAB70899.1 Homo sapiens (human) hypothetical protein
UACGCCGGAGCACGUCGCCGUCGGUCGGGAGGAACAACCCGAGACGCUCUAAGAAGGGAAGGAAC
UAGUACCAGAAGUUCCGGAGAAGUAGCGGUAGGAACUACCGGAAAUAGUUCCCCAGGCGUCCGCG
GUCGACCCUCCAGAACUUGUUCGACGACGACAUAAAGACGUA
>EMBL_CDS:CAH73567 CAH73567.1 Homo sapiens (human) A kinase (PRKA) anchor protein 2
UCGCCCAUACGGGACGUCGUCGGUAAAGAGGGCUUCGUGAAACCUGAACCUUACGACGUCGUCUU
CAUAGACGA
>EMBL_CDS:CAI21524 CAI21524.1 Homo sapiens (human) RAE1 RNA export 1 homolog (S.
CAAAACAAGACCGUAGAAACAGGGUCUUCGACUUAGAUGGUAGUCUAGGGUGUCAACGUUCCCAC
GGUACUUGUCCUA
>EMBL_CDS:CAH73982 CAH73982.1 Homo sapiens (human) human immunodeficiency virus type I
CGUCUCCCAGGCAAUGAGGAAGGUCUCAGAACAAGACACGACCCAUUGGAAGACCGUUUCCCUCA
ACCACCUGAGUGACA
>EMBL_CDS:CAB70832 CAB70832.2 Homo sapiens (human) hypothetical protein
GUCGUCGCAGACAAGACACCUCGAGACCCGGACUAUCGCAAACGGCAACCACGGGUGCGUGGUGU
GAAGGGCGACCUCGUCCCCGACGAC
>EMBL_CDS:CAH71782 CAH71782.1 Homo sapiens (human) Nedd4 family

interacting protein 2
GAGAAGGAGGACUUAAGAAACUCUACAAAGACGACGACGUCGACGGUAACGUCGUCGAAAUCGGA
AGAGUCGAAGUAGCAUACAUCCUUCUC
>EMBL_CDS:CAH70133 CAH70133.1 Homo sapiens (human) zinc finger, MYM-type 2
ACCACACUCUCUCAUCUAGGAGAAAAUUGAAAUGACUAAAUCUUCUAAAAUUGAGUAGAUUAUGG
U
>EMBL_CDS:CAH72509 CAH72509.1 Homo sapiens (human) guanylate cyclase type protein
CGCUGGAGGAGCCACAAACCAUCUGAACAAAGGUUUUCGGAAGCAGAGGUGCCGGAAAAGACAUC
CUCGACUCCCGUUACUCUUCGUCACUGGGUAUGUGGAAAACCUCCACG
>EMBL_CDS:CAB70719 CAB70719.1 Homo sapiens (human) hypothetical protein
GUACCGCACCCUACCACACAGAACUCUAUCGUAUAGAUCCCUAACGUAUAGAACCCUACAGUACA
GUAC
>EMBL_CDS:CAC05478 CAC05478.1 Homo sapiens (human) progestin and adipoQ receptor
CUUCUCCUAGACCUCCCACACCAUGGUCCGACGAACGAGGACCGUCGGUCGGUCCACUGUCUCGC
GGUGUGCCACACGGUGUCCCGACUACAGAUCCUAUUUUCGGUCUGGACGACCGUGGUGAACUGUC
UAGAAGGAG
>EMBL_CDS:CAB66731 CAB66731.1 Homo sapiens (human) hypothetical protein
ACAAGAAGGGUGAGACCUUCAGAACUAAAAUUGACAAAUUUUAGUAUACCGACCCAGUAUUAUCC
UUCUUAU
>EMBL_CDS:CAH72176 CAH72176.1 Homo sapiens (human) novel pseudogene
CUCAUGUGUGUCGACCCACCUUCUCUUGUGUCAAGACCAGUAGACAAAGAACUGGUCCUGAAACA
GAAGGAAAAAACGCCACGAG
>EMBL_CDS:CAB66803 CAB66803.3 Homo sapiens (human) hypothetical protein
GGAACUGGAGUCGAUGCUGCCUCCCGAACCGACUCCCCGCACCGGAGAAGACUCCGACCGAGAGA
CCCAAGAGGACGACCCUAGCUCC
>EMBL_CDS:CAB70673 CAB70673.1 Homo sapiens (human) hypothetical protein
CGGUAAAAGAACUAAAUAUCUACUUACAAGUCGAAAGAAAAGUCCUCUUGGACUUACUCUUUACU
UUCCUUGUCUUGGUUUUCCUAGAAGACCUCCUACCA
>EMBL_CDS:CAI23454 CAI23454.2 Homo sapiens (human) hypothetical protein
ACGCAGAUUCGUCGACGCCGCCCCGAUCUUGGCGUCACUCGAGGAGCGUCGACGACGACGCGU
>EMBL_CDS:CAI23380 CAI23380.1 Homo sapiens (human) myeloproliferative leukemia virus
CGACCGAGGAGGGUCGACUAGACUUCAAGGGGACCGACCGAGGGUGGGUACCGGAACUACUAUGA
CCCCCCUCGGCCG
>EMBL_CDS:CAQ08768 CAQ08768.1 Homo sapiens (human) ribosomal protein S26 (RPS26)

CUCCAACCACCCGUCGUGGGUGUCCAGAUUUAGUCCCAACCCCACAAACCAGGAAUGGCCGAAG
>EMBL_CDS:CAB66720 CAB66720.1 Homo sapiens (human) hypothetical protein
GUCUCCGUCCCGGUCCGAGGACCUCGACCCGGAACUGGGCCCUGAGGUGGAACAGCAUGAACCCA
CUCGCGGUGUCUCGAAAGUCCCCGGACCCGACCGGGAC
>EMBL_CDS:CAI16091 CAI16091.1 Homo sapiens (human) potassium channel, subfamily U,
CAUCAUUUGGUAACCUUUGGUUGUCUAACGUAGGUCUUUUACGUAUAGACCGCCCAAUAGGUUAU
CGUCAUGAUA
>EMBL_CDS:CAH69976 CAH69976.1 Homo sapiens (human) muscle, skeletal, receptor tyrosine
CGGCUCCGAAGAAGAAACUCGUAGAAAUGACGGUGGUAUCACUUUCCAAGUAUCCCUUCAUUCGG
ACCA
>EMBL_CDS:CAI22140 CAI22140.1 Homo sapiens (human) Rho guanine nucleotide exchange
CCUAUACGGAGGCCUGGUGGACGACUCCCGAGUCCGGGAGACCGUACCCCGGGUCGAGACCCCGA
CCCCCGAACUACAGG
>EMBL_CDS:CAI41303 CAI41303.1 Homo sapiens (human) transmembrane protein vezatin
CCUAUGACCUUAGUUCAUCGAGUCACUCGAGGUGUAGGAGAACUUUGUCCUGAAGGACAACGUCC
UACCAUACAUUACUCAACUCAGCCUUAGGUUAUAGG
>EMBL_CDS:CAM28254 CAM28254.1 Homo sapiens (human) RAE1 RNA export 1 homolog (S.
GGCCGUUCCAACCACCCGAUUUUCUGUCUGUUGGUUACGAUAGUAGUCCUCUACUACAAUGAAGU
UAUAGGAAGUACCCUAACACUAACAGACAUCAACGUGACGGUUUGUACGACC
>EMBL_CDS:CAI12437 CAI12437.1 Homo sapiens (human) ankyrin repeat and sterile alpha
CUCCCCACACGAAGAAUCACAACGUCGAGUCCUCCAACCCCACACGUAAUUCCUCGUAAAAGUGG
UGGAG
>EMBL_CDS:CAI95134 CAI95134.1 Homo sapiens (human) transforming, acidic coiled-coil
CGGCCGUGACCAACGACGGACGGAAGGACCCAAAGUAGUGUACGGAGACAGAGACCCCGUAGGAC
CCACCCACACUGAGGCCCCGACCGCCCGCGGUCCCGACCG
>EMBL_CDS:CAI22280 CAI22280.1 Homo sapiens (human) KIAA1324
CAACAGUGGGGUUAUUCAGCUAGUCGUUCCGACUGUCCGACACUCCUUUGGGGCCGGAACAUCGG
ACAGUGGAGACCCCCCUACUACUG
>EMBL_CDS:CAB70809 CAB70809.1 Homo sapiens (human) hypothetical protein
ACGACCGUUCCACGAGGAACCUCCACUAUUAAACCAGGUACAGUCAAGCCUACUGCCGGACGUAC
CGGCCGACGUGGACGACGUGGAACCUGUCGU
>EMBL_CDS:CAI16917 CAI16917.2 Homo sapiens (human) novel pseudogene (KIAA0563, FLJ34306)
GGAUGACAAGGAGACCACGCCAAUUUCGGAAUAAAACCCGCAUUGAAAGUCUGACGUGGUCACUU
AGGACUCAUCC

>EMBL_CDS:CAI22406 CAI22406.1 Homo sapiens (human) latrophilin 2
AACUACGGGUCUCCUUUAAAUUUCAGAACCUAGACAGGAAGACAUGACUCAUGCCGUUGAAGGUC
CUGUUAUAAAAGACACCCGUAACU
>EMBL_CDS:CAI13041 CAI13041.1 Homo sapiens (human) chromosome 9 open reading frame 3
AACUACUGGUACAGAGGUAGAAGUAAAGACAGGUCGUAGGGUGACGUUAACACUUCCACCUCCGA
CCGUAACC
>EMBL_CDS:CAM15931 CAM15931.1 Homo sapiens (human) novel protein
ACGACUCAGGACACGUGCAGAACCGGCAAAACCGCAUACAUCCUACACGUCACGUAAGUCGU
>EMBL_CDS:CAQ09540 CAQ09540.1 Homo sapiens (human) novel pseudogene
ACUCUCCUUACUCGUCGUCGUAACAGUACCAUCUCUUUCUGAGAGACCACUUCGAGAAGGUCCUC
AAAAAGACGAUUUUGAAAACGAUCGAAAGAGUUUUACGAGAGU
>EMBL_CDS:CAI40484 CAI40484.1 Homo sapiens (human) NADH dehydrogenase 1 (MUND1)
AAAUACCAUAUCUCAAGACCACUUAUAUACCAUACAUCACGAGGAUCCUUCUAUCACCAUCAAUC
CCGUAAAUAAUACUAUUAUAAGUACAUGAGACGAUAUUU
>EMBL_CDS:CAH72050 CAH72050.2 Homo sapiens (human) collagen type IV alpha 2
CCGGGUCAGGACCCCCCGGGUAAAGAGGACCUCAUGGGAAGCAAGGUCCAUAAGGACCUAUCGGA
CCCAGGGGACCUCCCGG
>EMBL_CDS:CAI22896 CAI22896.1 Homo sapiens (human) complement component 1, q
CCAGAGGUCGGUCGGGACCUUCGGGAAAGAGAGGAAAAUAGGGACCCCAGGGUCCAACCGGUAGC
CCCGGUCCACAGGGUCCCUAUGGGCCCUACGGUCCCUACCGACCCCCGG
>EMBL_CDS:CAQ10023 CAQ10023.1 Homo sapiens (human) novel pseudogene
CGAUUCAGCGAAAACCUAAUAACUUACCCCCAAUAUUAGUGGGUCUUUUAAAUUUCCGCCUCUCG
>EMBL_CDS:CAI19463 CAI19463.1 Homo sapiens (human) peroxisomal biogenesis factor 6
GGGUGUCGGACCCCCCGGGGCAUCUUCCUGUGACGAUCAAGGACAGUCGUCCCGUGGGGGACCGA
CCUCC
>EMBL_CDS:CAI39429 CAI39429.1 Homo sapiens (human) DCN1, defective in cullin
CGGCGAGGUCGAAGAAGAACAGGUGUCGCAAGGCGUACCUGAGGGACACCUCGCUCAGACCCAAA
ACCUUCUUCGACAGGCACCG
>EMBL_CDS:CAH73425 CAH73425.1 Homo sapiens (human) mitochondrially encoded AUP
UCGACACCCUUUUGAUUAUCGAUUAUCAUGAUCACACAGAGGACGUUAAUUUACAUAAUCAUCUA
CUGACUGAUAUUACAACCGACAAUUAACGUGUCGA
>EMBL_CDS:CAC88170 CAC88170.1 Homo sapiens (human) ubiquitin specific peptidase 20
CCUCGUGUGCAUGUCCACCAAGUGCCGCCCCGGUGGGGGCUUAGACAACAUGUCCACGAGG
>EMBL_CDS:CAH70819 CAH70819.1 Homo sapiens (human) olfactomedin 1
GACGGGAGCGACAGGACCCGUCUCGACAUGUGGACGGUCGAGAGGAGUCCCAACCACCCGUC
>EMBL_CDS:CAI40281 CAI40281.2 Homo sapiens (human) chromosome 9 open

reading frame 102
CCGACGUUUGAACUCUUUAAAACAACCAUCUUAGAAAUUUACUGUUCCAAAGUCUUAGACUGUCG
GAACCAAAAUAUUCUCUAAGAGAAAAUAGUCUAAAGAAGGCCCACGCGA
>EMBL_CDS:CAI13431 CAI13431.2 Homo sapiens (human) CUAGE family, member 5 (CUAGE5)
GUCAAAAAGAAGUAAGUAAACUUUAGGGAACUUCCAGUAAAACCGAAGUCAAUCAUCACUAAACU
CCCUAAAACUGAGUAGAAGAUCUCUGAC
>EMBL_CDS:CAI16234 CAI16234.2 Homo sapiens (human) novel pseudogene
GCCUCUAGAGGGUUACCGAUUGGUAUCCGACGGCGAGGAACGACAGGUCGCCGACGAGGACCACG
ACCUCUCUAGAGGC
>EMBL_CDS:CAM27610 CAM27610.1 Homo sapiens (human) obscurin, cytoskeletal calmodulin and
GAGCGGACCCUCCGACUGGGACCCCUUGACGUCCCCGGCCCAAAACCGGAGGGUCCUUCUCUC
>EMBL_CDS:CAO03600 CAO03600.1 Homo sapiens (human) likely orthologue of Mus musculus
CCCUACUCCGAUACGAGUAGGCAGGGAGCACUAUUUCCAUUCCUAGUCGUAUCUUUGGGAGUAAA
G
>EMBL_CDS:CAI95112 CAI95112.1 Homo sapiens (human) PHD finger protein 19
AACUCCUCUACACCAGGAGCUAGACAGUGACCCCACGACCACUCGAACGGUUCGACGUCCUCGAG
GGUCACCACCAACUGUUUCCGGUCUUAGAGGAGUU
>EMBL_CDS:CAQ10511 CAQ10511.1 Homo sapiens (human) novel pseudogene
ACACACUUCCAUUCUCGGUGGAUUAAGACCGUAGUCAUUCCUUCUUCAGUUUUGACGAGUCUUCC
ACCUCGAACCGAAGUAUCC
>EMBL_CDS:CAI15983 CAI15983.1 Homo sapiens (human) CD274 molecule
ACCGAGGGUCUUAAUGGUUCACUCAGGAAAGUAAACCUCCUACACGGUCUCCAUCAAGACCCUAC
UGGU
>EMBL_CDS:CAI16895 CAI16895.1 Homo sapiens (human) ring finger protein 38
UCCCUCUACCACUAUCAAAAGUAGAACUGAUUACCUUAAUACAGCUCUGUCUGCUAGGGAAGAAG
GA
>EMBL_CDS:CAB90482 CAB90482.1 Homo sapiens (human) human type XVIII collagen
CCUCCAGGUCCCUUCGGAGCUCCUGGGGCGUCCCGGAGGUCUAGCGGGGGCUUCGGUCUAGGGAG
GUACAGUUACUUCCAGUCGAACAGCACAGACUUCCUCCCAGGACCCCCGGGACCUCCGGGAACCC
CAGGACCCCCGGGAGAGAGAGG
>EMBL_CDS:CAQ09916 CAQ09916.1 Homo sapiens (human) pseudogene similar to part of
UCCUCGUUAUCACCUUCCGUCGAAUCGUAACGGACUUAAUUCUUUUUUCUGUUCGAAAGAAAAUU
AAUUAAGGUCUCAUUACACUAAAGGUACAACAGGA
>EMBL_CDS:CAI23559 CAI23559.1 Homo sapiens (human) single stranded DNA binding protein
CGACGGCAUUAAGACACCCGGCCCUGGGUACCCGGGGUACGGAGCCCCUCCCAAGUAAGAGACGU
AACUAGGAGGGUACAACCCCACCGGAACAACAGCACACCCUAGGUAUCUUAACCCGUCG

>EMBL_CDS:CAB82417 CAB82417.1 Homo sapiens (human) hypothetical protein
CAACCGGAAAGACUUCCGAUAUUACAGUCCUAGCAGGACUUUCAGAACUCCAAGACGGUUG
>EMBL_CDS:CAI15920 CAI15920.3 Homo sapiens (human) chromosome 9 open reading frame 48
GGGUAGGACCUGGUCGACGAGGCUCCCUCCUCUGGGAAGCAACAGACCCAAGAGGUCCUCCC
>EMBL_CDS:CAH72907 CAH72907.1 Homo sapiens (human) interferon pseudogene
AGAGUACCUCUUAUAACCACCGGACUCGGAAAACCUUAAACCACGGUAGGUAGACGAGGUCCUCU
>EMBL_CDS:CAQ07755 CAQ07755.1 Homo sapiens (human) interleukin 6 receptor (IL6R)
UCCGACGACCCCCUGUGACUUCCCUCUACUCUCCUUGUUCGUGACCCCACCAAGAUCCAGAGAGA
UCCUGGUCGGUGGGGUUUCUUGUGUUGGUCACGUAGGACUAGAAUAGAAGAAAGUCUGGGACGUU
GAA
>EMBL_CDS:CAI14820 CAI14820.2 Homo sapiens (human) novel protein
GGACUGUACGAACAGUGGCUACCGUACCACCGCUUCGGUCCAGAAGGAACCCUUUGCGACGAGAC
GGGUCCACUGUCCACAUUAUGUCAACCC
>EMBL_CDS:CAQ10338 CAQ10338.1 Homo sapiens (human) NHP2 non-histone chromosome protein
GAAGUGUAUCCUCGACCUACACAUCUUUCUAGAGGUUCUAAACACCGGUAGUGCUAUUAAAACCU
CCACAGAAAAAUUUUCCAGAGUAACCGAGGAAAUGUUUC
>EMBL_CDS:CAQ07400 CAQ07400.1 Homo sapiens (human) novel pseudogene
CGAGACCCCGCGAGGGCAUCGAACCCUCCGAAACCGGAGUCUUCGUCCAUCCCCCACAAUGACCC
G
>EMBL_CDS:CAI14818 CAI14818.1 Homo sapiens (human) tubulin folding cofactor C
ACGAAAGUUGACGGCCCCCCUACGGUCCUCGGCGCAGAUGAAACCAUCUGCUUCGUCGUAGGAAA
GGGGCCCAGAACUUUCGC
>EMBL_CDS:CAI10944 CAI10944.1 Homo sapiens (human) protein prenyltransferase alpha
AGAAGAUCUUCUACCCCUUCUAAUUAGGAACCAAGAAGACAACUUUGACGACGAAGAAGUAGAAA
ACCUCCU
>EMBL_CDS:CAH70960 CAH70960.1 Homo sapiens (human) far upstream element (FUSE) binding
CGACCCCUUGGGGGGACUACCACUUAGAGGCGUCCAACCCCAGCGACAACCCUCCCCCCAAGGAG
ACUUCG
>EMBL_CDS:CAH72910 CAH72910.1 Homo sapiens (human) interferon, omega 1 (IFNW1)
UACUUCAGGUCACACCUCAACCAGGUCCUCCCAGCACAAGGUCCGUCGUCUCCUCGCGAGACACA
CCUUCUCCGACUUCUAGACGACGUCGUAGAGUACCUCCUGUCUGUCCCGGACCCGGACGACGUUG
ACCGACAGUA
>EMBL_CDS:CAH74092 CAH74092.1 Homo sapiens (human) KIAA1614
AGACAGACCAGUGGGUCGGACUCGUCAGGUCGGUCCCGACGGAAGGACACUCUGUGACCGCUCCA
CCAAAAGGAGCUUCCCCCUCCCCAACCGCGGGUCGGAAGUCCUGUGUUCCUUCGACAACCGACGG

UCCGAACCACACCCCGUCU
>EMBL_CDS:CAC16001 CAC16001.2 Homo sapiens (human) CGI-121 protein (CGI-121)
UAAACGACUCUAUGGUGUAAAAAAGUUACGGAGACUUUAUAACAAUAAACCCCUUUCCAACUUUU AUUAAAGUCAUCUA
>EMBL_CDS:CAQ06692 CAQ06692.1 Homo sapiens (human) olfactory receptor pseudogene
AGGGACAAAGAGUCCGACAUCUACUUCCCCAAGUCGUACCCCCACUGGUGGCACAUGUAGUGACU CCGGUGACGUCGGAAAGAGCCCCU
>EMBL_CDS:CAI39663 CAI39663.1 Homo sapiens (human) myeloid/lymphoid or mixed-lineage
CUUAAUAGUCCAUUUUUCGACAAAGGAUAUACUCCCGAGAAUGACGACAAGUUCCUUAGUCAUCA UCCUCGUCAGACACGACUUUUACACACAUAAACUACUCUG
>EMBL_CDS:CAQ09825 CAQ09825.1 Homo sapiens (human) hypothetical protein
CCCGUCAUCCCCGACACGAGACCGUCCGUCCGGUACCUCAGUGUCCUGAGGUUCCAGACGUUCAC CUUCCUCGAGAGGACCAGGACGUACGGG
>EMBL_CDS:CAB90455 CAB90455.1 Homo sapiens (human) hypothetical protein
UCGACUUCAACAAAGUUUUUAACCAAAAGACAUUCCUACCGAAGGUUAAAUACUACGUAUAGUCG A
>EMBL_CDS:CAH74053 CAH74053.1 Homo sapiens (human) solute carrier family 12,
GUCACCGGUACGUCCACUCGUGUGUCCACGGCAUUUGUGCGUACAACAAGUCGUACCGACGAC
>EMBL_CDS:CAI22065 CAI22065.1 Homo sapiens (human) ryanodine receptor 2 (cardiac)
CAACCUAAAGAACCAAGCCAGUAGGUCUUCUCACAUCCCUUGUUCCGCUCCUAAAGAAGACAAGA AAUGCAGGACAACCUACGGUAUUCAGGUCG
>EMBL_CDS:CAQ08581 CAQ08581.1 Homo sapiens (human) novel pseudogene
AGGAGACACCCCAUCUCGUCCAGGUCUAUCUGGAGAGAGACCAACUGGUUCCGGAAGGGGUAACU CCU
>EMBL_CDS:CAQ07592 CAQ07592.1 Homo sapiens (human) 60S ribosomal protein L12 (RPL12)
ACAACCCUUUACAACUGUUAGAGUAGUUUUCACUAGAAAAGUGAUACAAACUACAAGACAAAGAA AGAGGAUCGU
>EMBL_CDS:CAI15578 CAI15578.1 Homo sapiens (human) G protein-coupled receptor 111
UGAGAGAAACAGUUCUCUUCGAACGAUGUUUAGUGUCCGACCCGAGAACAUACCUACUCUCA
>EMBL_CDS:CAH72236 CAH72236.1 Homo sapiens (human) thymidylate synthetase (UYMS)
CUUCGGUCCCGUCUUACACGAACUAGAAGAGACCCAUGACGUCAACCGGAGUCUCUACGUCGGGA CCGAAG
>EMBL_CDS:CAI20310 CAI20310.1 Homo sapiens (human) erythrocyte membrane protein band

CGCCCAGACACGAACCCACGCCGGUGAUAUCGCUUUAAACCUGGGGUUCCAGUCCUUGAACCGAA
AACCACCGACGAGACCUCUUUGUUCGGACA
>EMBL_CDS:CAM14169 CAM14169.1 Homo sapiens (human) ankyrin repeat and sterile alpha
CCGACGAAAAAAGGAGACCCGUCGCUAAACCCUGUUCUGUAACAGAUACGACGGUGGACUCGCCA
AUUCGUCGA
>EMBL_CDS:CAQ08002 CAQ08002.1 Homo sapiens (human) ribosomal protein S3A (RPS3A)
UCUGUAGUACCUCUCCUGUUUGUCUAACCGUUCGGAAAAAUACAGAAAAGGUUACGACAGA
>EMBL_CDS:CAI12548 CAI12548.2 Homo sapiens (human) collagen, type XV, alpha 1
CAGACGGAGGACGUCGGGUGACCAGAGAGGUCUACGGCUCCUGAGAAGUCCCAGUGUGUCGAGGA
GCCCUCAGGACCCCAGCCCCACGUGCCACUCGACGACCUCCCUCGG
>EMBL_CDS:CAH73281 CAH73281.1 Homo sapiens (human) chromosome 20 open reading frame 45
UACAACCGGUCCGACCAGAGCUUGAGGACUAGAGUCCACUAGGAGGACGGAGCCGGAGGGUUCCA
CGACCCUAAUGUCCUCACACAGUGGUACGGACCGGACGUA
>EMBL_CDS:CAI13397 CAI13397.1 Homo sapiens (human) chromosome 6 open reading frame 190
GUUCUUCACAGAAAAUCCUUUUAACGAAGAUUAAAGACUUCGAUUCUAAGAACUACGGACCAUGA
AAAAC
>EMBL_CDS:CAM21543 CAM21543.1 Homo sapiens (human) protocadherin 15
CCGUUUCACCUCUUACUCUUCACUCCGGACCCUUUCGUUUUACUUCUCAGACUUCUCUCUCUAAA
GUUGACAAGACAAGGAAGAUAGUAGUCACAAAGUGGAACGGAAUAAAGGAGAAAGAGACAGUUUA
AACGG
>EMBL_CDS:CAM22929 CAM22929.1 Homo sapiens (human) chromosome 10 open reading frame
ACAUAAAAACAAGGAACUUGCAAAAAACCUUCGUUGACGAAGGUCUUUGAAAGGAAACUUAUCAG
UUUUUUAUGU
>EMBL_CDS:CAI16805 CAI16805.1 Homo sapiens (human) chloride intracellular channel 5
UGGGAAGGUAACUCUACGGGGACAGGAGGACCCCGAGUGGACCUACUCGGAGGACCUCAAGUCUC
AUAUAGAGUAGUCCGUCCUCCA
>EMBL_CDS:CAI16482 CAI16482.1 Homo sapiens (human) hypothetical protein
CAUCGACGACAAGUAUAAAACCCGUAAUUCGUACACGUACAGGUGGAGGAAGUACCUGUACACCG
GGGACUUGUACCGUCGAUG
>EMBL_CDS:CAM14602 CAM14602.1 Homo sapiens (human) thrombospondin, type I, domain
GACGAGCGAGUCGAGCGUCUAUAAGAGGAGCAGGCUCAAGAAGGCCUUCGACCCCCUCACCUACC
UCAACACAGCUCGUC
>EMBL_CDS:CAC40698 CAC40698.1 Homo sapiens (human) dJ1104A8.1 (novel protein)
GUGAACGUAAAGACUAAAACUGUAACUCAGAACUACGGAUUCAUAUUCUAAAAGUAAACCAAGAA

AUAGUCCUCACCACUCAC
>EMBL_CDS:CAI22937 CAI22937.1 Homo sapiens (human) ataxin 7-like 2
CGACCCGGGACGGGUAAGUGAUGUACCGUAAAGACUCUCGACCGUCCCCGACCUCCACCCCCACC
CCGGGCCG
>EMBL_CDS:CAI15464 CAI15464.1 Homo sapiens (human) novel protein
CUGAAGGAGGUCAAGGCCGCCUAGGACCGUGAAAAGAAGGAUCUCACGUCUAUGACGAGUGACCU
CCGACAG
>EMBL_CDS:CAH71701 CAH71701.1 Homo sapiens (human) DEAH (Asp-Glu-Ala-His) box
UCACAGUCCUCAUAGUCAUUCACCCCCGCCUCUACGAUGGGGUUUUCGACCUUGAAGAAGUGA
>EMBL_CDS:CAH72941 CAH72941.1 Homo sapiens (human) platelet endothelial aggregation
GGAACCCGGGGUGGACCGGCGAGUCCCAAGACGUCCGACCGUUUCUCGCCCGGACCUUGGAACAA
UCCCCCACCCCCCAAACCCC
>EMBL_CDS:CAH72281 CAH72281.1 Homo sapiens (human) tudor domain containing 3
AAACCCUCAGAGAAACUUUUGCUCCGCCAAAAAGUAAAGAACCGACGGACCAUAGUAAAGCUCCU
CCUACAGAGUCU
>EMBL_CDS:CAH72748 CAH72748.1 Homo sapiens (human) novel pseudogene
ACAGACAAAGAGUCCAAAUAGGUAAUGUGUCAAUACACUCCUUUACCUGAACAGUAUAUCUGU
>EMBL_CDS:CAH72940 CAH72940.1 Homo sapiens (human) Rho guanine nucleotide exchange
AAGAGUUUCACGACUUACUACAAGGAGUGUAACGGACCAAACUGAAGGUGUCCCCUGUUCCCUUA
UACUUUACACGAAACUCUU
>EMBL_CDS:CAH72663 CAH72663.1 Homo sapiens (human) calmodulin binding transcription
AACGAAGGACUAUAAACCGAGAAGGUCUGAGUCACGUCACCCUUCGUCGAAGAGGACGGAACAGA
CCUUCAUGAGUCCCAAGUU
>EMBL_CDS:CAI12394 CAI12394.1 Homo sapiens (human) poly (ADP-ribose) polymerase family,
GGUCAACAGAAGACUUCGAGUCAUCAGCUGUGGUAACAUGCUUUAAGAAAGAGAACUUAAUCACG
UGUGUCUCAACGGACACACGUCACUCCUUACUUAGGUAUCUGUUCCUCAGCUAGUAACUCUUUGU
UCCUGCCGUGGACC
>EMBL_CDS:CAQ06897 CAQ06897.1 Homo sapiens (human) DEAH (Asp-Glu-Ala-His) box
UUCCGUCUUACUUCCAGUGGAGGUACAUUCUCUCGUAACCAGCACUGUAGAAGUUACAGAA
>EMBL_CDS:CAI15500 CAI15500.2 Homo sapiens (human) ADAMUS-like 4
CCCGGCCGUCAAGUCAAGUGUCGCGACCCUCGGGACCUGAAGUCACUUCCCGAGGGUGACUAUGU
CGACCGGG
>EMBL_CDS:CAH73232 CAH73232.1 Homo sapiens (human) ankyrin 3, node of Ranvier (ankyrin G)
AAGUAGAACGUCCAGUAUCGGUAGGUCUCUCAGCUAGAGACACCGUAGGCACAGUACUCUUUUGA
GACGACACCACCGUUACCUUGUGAGACACUACCU
>EMBL_CDS:CAI21793 CAI21793.1 Homo sapiens (human) potassium channel,

subfamily K,
ACACUAGCUCCUCUUCCUGUCAACCAGUACGAGAUACUACACGUGGACUAGGAGCAGGAACAGGA
AGAAGUGU
>EMBL_CDS:CAH73228 CAH73228.1 Homo sapiens (human) transmembrane protein 206
CCUCCGCCUGAGCGACAGGUCCGGACCAUUCUAUGGGCCUGGAACCUGAGACUGGCAGAGG
>EMBL_CDS:CAI12685 CAI12685.1 Homo sapiens (human) chromosome 9 open reading frame 86
AGGAGGUGUGAGACCUGGCAACGACCCGGGAGACGCCGGCCCUGACCGAGACCUCCACCUCCC
>EMBL_CDS:CAI14117 CAI14117.1 Homo sapiens (human) BUB (POZ) domain containing 14A
GCUCGACGAGCGACCGGCAGUACCACUCGGACGGGCACAUCGUCUUCCUGUCCUAGACGACCUUC
GUCCGCCCGCCGUGCCUCGGCCCGUCGAGC
>EMBL_CDS:CAI22394 CAI22394.1 Homo sapiens (human) mutS homolog 4 (E. coli)
AAACUCCUAUUAAUCAUAGAGCACGUCUUCUACUCGAACGUGGUUUUCAUUUUAAACUACGUUGA
CACAAAAGACGUAUUCGAGAAAACAAAGGUUU
>EMBL_CDS:CAI14911 CAI14911.1 Homo sapiens (human) sprouty homolog 2 (Drosophila)
CCGCACGUCCGGGUUUAGAAGGAACGAGUCACCGAAUUCGAGUGGACCGAACUCGAGUCUAAACC
CAACGUGGG
>EMBL_CDS:CAQ08176 CAQ08176.1 Homo sapiens (human) eukaryotic translation initiation
CAGGAGUCGAUCUUUCAGACAAUCCCUCUAUCAAAAGGAGAAGAAUAAGCAGAAAAAACGACUCC
GG
>EMBL_CDS:CAI16862 CAI16862.2 Homo sapiens (human) AU-hook transcription factor
ACCCCACCGUGCGGUCCCGGACCUCCCCGACGGAGUCGGAACGGGUCCGGACUGUACACGGGCAG
AGAU
>EMBL_CDS:CAI16233 CAI16233.1 Homo sapiens (human) collagen, type XXIV, alpha 1
AAGGUUCCUCAGGGAAACCAGGAAGUCCCGGCAAAACAGGUCCUUAGGGUUAUAGAGGACCUUUU
GGGAGACCAGGACCUCCCGGUCUACCAGGAACCUC
>EMBL_CDS:CAI13215 CAI13215.1 Homo sapiens (human) misato homolog 1 (Drosophila)
AUCCCUAUUCACCAAAACCUCAACAUCGCCACCCCUCACCACUCCUUGGAAACACACACCACUCG
AAGGGGACGGUACGAUAACGUCGUAGGUUGACAAACAGGGAC
>EMBL_CDS:CAH71957 CAH71957.1 Homo sapiens (human) potassium channel, subfamily U,
AAUCUCGGAGAGAGGGCAAGAAAGAAAAGGUCAAAACUUUCUCGUUCUCUCAUUGUCAGAAACCG
AGACU
>EMBL_CDS:CAI19369 CAI19369.1 Homo sapiens (human) DENN/MADD domain containing 2D
GCCCCUACUUCGAGAACUCUCACUGUCAGAAGGGUCCUCGUCCCUUCCGACGGAGAGCCUCCGGG

AC
>EMBL_CDS:CAI41373 CAI41373.1 Homo sapiens (human) ankyrin 3, node of Ranvier (ankyrin
CACAGAGGCAUCAGUUACAAAAUUACCCACUGUCGUUCCGACGACGACUAAAACAUUUACUUAAU
CUCAGACCUUCAAAGAAAUUACGACCAAGACCGUUUUGUAACUGAUGUCUCAUAUG
>EMBL_CDS:CAM23326 CAM23326.1 Homo sapiens (human) ring finger protein 17
AUACAAGAAUCUCUUCCGCUAUUAACAUUACGAGAACGAUACAAGUAUCGACGUAAGUUUAAUAA
AAAAAAGAGAAGUUACAU
>EMBL_CDS:CAC14920 CAC14920.1 Homo sapiens (human) solute carrier organic anion
CGCCGCGAGGUCCCUCCACUACUGACACAACUACUUCGGUAAGUGUCAGUAGGGGACGUCCUUAC
GCCGGCG
>EMBL_CDS:CAQ08463 CAQ08463.1 Homo sapiens (human) mitogen-activated protein
UGGUAGUCAACUCACGUCUCGUCGGGUAAUGUGUCUCUAAGUCAGUUUAACCCAAAGUAGACGAG
GGGUGAGAGAACUACCA
>EMBL_CDS:CAH73892 CAH73892.1 Homo sapiens (human) novel pseudogene
CCGACCCUCCAGCCCGUGAAAUAGGUGGACGAGAAACCAUGACCAGUAGACCUCCACCCACGUCU
CCACCAGGUCGGUAAGGUCAACAAGUGCAGUGGUAUCCACGUGGCCCAUGAAGAACUCGUAACCC
UACUAGACGAAGUGGAGGUCAG
>EMBL_CDS:CAQ10739 CAQ10739.1 Homo sapiens (human) cytochrome P450 4Z1 (CYP4Z1)
CUCUAGACGAAGUCUCUUCAGAAACCACAAAAGUGAAAACCGUGAGUUUUCAUACAGGUCUUUUA
GGACCGCAGAAAAGACUCAUCAUAGAA
>EMBL_CDS:CAI13563 CAI13563.1 Homo sapiens (human) nuclear distribution gene C homolog
CUGUCCCAGACCCAGGUCGCCAUUAACCCGUCCAGACGGGGCAACGGAUCCAACCCGAAGUCAAA
AGGAAACAG
>EMBL_CDS:CAQ07786 CAQ07786.1 Homo sapiens (human) thioredoxin 2 (UXN2) pseudogene
GUGACCGCAGAGUGACAACCAGAACUUUCAGUCCAGGUAGGACCUAUAAUUACCAGUAACUCCUC
UACGACCAC
>EMBL_CDS:CAI40867 CAI40867.1 Homo sapiens (human) DEAD/H (Asp-Glu-Ala-Asp/His) box
ACCAACCCACCGUAUUCUAGAGGACAGUAUAAAUAUUCGAGACUACCUCGUCUAAAACUGUCCUU
CGGUAGUCCACACUAGACUGGU
>EMBL_CDS:CAI23224 CAI23224.1 Homo sapiens (human) EGF-like-domain, multiple 9
GUCUUGAACGGACGGGUCGGUGACACCGUCUACGUGACGGUACCGACCACCGUCCAUGGCACGAC
>EMBL_CDS:CAI12104 CAI12104.2 Homo sapiens (human) discs, large homolog 5 (Drosophila)
CCUGUCCGGACCCCCGUCUGGACGUUCCCAGGGGGUCCCUUACCGAAUACGUCUUUCCUCGACGA
CGGAGGACCCCCCGGACCGAGGAAGGACAGAAGGUCGGACAGG

>EMBL_CDS:CAI41410 CAI41410.1 Homo sapiens (human) interleukin 13 receptor, alpha 1
GAGUCCUAGUGGAAGACCCCCACUCUACGUAAAAAGUUGGUUUUACGAUCCGAAGAGUGAGAGUA
ACCACGAUGUGACCC
>EMBL_CDS:CAH72952 CAH72952.1 Homo sapiens (human) laminin, alpha 2 (merosin, congenital
ACGACAGUGAAACCAUCGGGACCAGUCGUCGAGCAAGUAAAGCCAGUGCUCACAUAAGUCUAACG
GGAGACGGUCGACUUAUUCGGAGAGACCCCGGGCGACUCCACUGUCGU
>EMBL_CDS:CAH73851 CAH73851.1 Homo sapiens (human) KIAA1797
AAGAACGAAGUGUUCACUACAUUUUUUAAGGAGGUUUCGACCAUUUUGGUUUCCCCCUCACUCAC
UGUUGUCAAACUCUAUCGUUCUC
>EMBL_CDS:CAH71812 CAH71812.1 Homo sapiens (human) mutS homolog 4 (E. coli)
CGUCAUAAACUGUAAUAAUAAAGGUUUCCACUAUUUUAAAAUUCAAAUCACUAGUGGAAACGUAU
ACAACACAACAGACG
>EMBL_CDS:CAI12711 CAI12711.1 Homo sapiens (human) novel protein
CAAACUCGUCAAGAUAUAGGAAGAAGUGGCAGUACCCUGAAUUUGAAUAAAGAUUACUUUAUACA
AAAUCUCUUACUAACUCCUCCACGUCUAGGUUAACGAAUUCG
>EMBL_CDS:CAI15628 CAI15628.1 Homo sapiens (human) AUP synthase, H+ transporting,
CACGAACUCGGAAGAAACGGUCGUCGAAAUCCAGGCGUUACGUGAACCGUUAACAGAGGAAAAGG
ACGACACACCUCUACGAAACGUG
>EMBL_CDS:CAH72158 CAH72158.1 Homo sapiens (human) DAB2 interacting protein
GGAGUCCGCCCAUGACCCCGGGUGGGUUAGUCCACUCCGGCUGCGGUCCCAAGGCGAACUCC
>EMBL_CDS:CAI13226 CAI13226.2 Homo sapiens (human) chromosome 6 open reading frame 170
CGUGGACAUCCUGAAAGAAGUCUUCUCAAGUAUAAACGAGGUAUUUCCUCAUUUUAUUCGGGAAG
AAGAUGUCUACG
>EMBL_CDS:CAI13318 CAI13318.1 Homo sapiens (human) pancreatic polypeptide receptor 1
CUCCGUGUACUCCUUCAGUCUCUUCCGGUCCAACCGCUAUUCGUCCAACCAGUGCAACCGAAAGA
GGAAGACGGAG
>EMBL_CDS:CAC09445 CAC09445.1 Homo sapiens (human) hypothetical protein
AACAGUUUGGUCUAAGAUUAAACGAAAAAAGAAGAAGUCAUCGAACUUCGAGACCGUGAUUCUUU
AGGAGACCAAACUAUU
>EMBL_CDS:CAH71575 CAH71575.1 Homo sapiens (human) DEAD\/H (Asp-Glu-Ala-Asp\/His) box
GACAAGGUAAGGACCCGUACUUGAAAUUGAAUUGUUGCUCAAGUUAUCGUCCCUACUCUUGUC
>EMBL_CDS:CAQ10164 CAQ10164.1 Homo sapiens (human) actin, beta (ACUB) pseudogene
CUCCGCAUGUCCCUGGUGUUCCGGACCUACCGGUGCAUAUACCGACCCCACAACUUCCAGAGUUU
GUACUAGACCCAGUAGAAGAACGUCAACCGGAACACCAAGUACCCCCGGAG

>EMBL_CDS:CAC36039 CAC36039.1 Homo sapiens (human) oviductal glycoprotein 1, 120kDa
GAUCUCGAUGUCACACGAAAGGUUCCCUAUGUCAAAGGAAACAUCCCCAGUGUCAACAUGGAGAU
C
>EMBL_CDS:CAH73027 CAH73027.1 Homo sapiens (human) protein tyrosine phosphatase,
CGGUUGGUGGUGGAACUACACCCUCCGUAAUUACAUAGGACACAAUAAAAGAAACCAACCA
>EMBL_CDS:CAC09446 CAC09446.1 Homo sapiens (human) hypothetical protein
CGACUAUCACACAGACCUAAGACGUAGACGACGUCCUUCCGACCCUCGACACCGGCGUAGACGAG
GACGUCGACUCCUAAACGUCAUUUGUCGCCCGAUAAGUCG
>EMBL_CDS:CAI40860 CAI40860.1 Homo sapiens (human) suppressor of fused homolog
CACAUCGAGAAGUCACGUCUGUGGUUGCUAGACCUCCUUCCAUUGAUGGGGUUUCCCACAGACGU
GCCCGACGUA
>EMBL_CDS:CAI23469 CAI23469.1 Homo sapiens (human) neuroblastoma breakpoint family,
CCAACCGGUCCCUCAUCCGUCAUCGAACUCAAUAUUCCUUAAAGAGAAACUCCAGAGCCUUGACG
ACUAACGAGAGACGGUCGA
>EMBL_CDS:CAQ07609 CAQ07609.1 Homo sapiens (human) novel pseudogene
GAUCCUAAAGUAGACCACGUAAAACAUUUUGGAAGUAAUUUCCAAAAGUGUCUCACACUAGGAUC
>EMBL_CDS:CAQ10534 CAQ10534.1 Homo sapiens (human) solute carrier family 9 member 3
GACCGGUCGUAGAAGUGCCACAUCACGUACCACAACCGGCUGACGAGGUUCUACAACCGGAAGUA
CAUGAAUACUGUCGUCUACGGUGUCUUCUAUUACCGGUC
>EMBL_CDS:CAI95313 CAI95313.1 Homo sapiens (human) KIAA2026
AUCGACCUUACCUUUGUCUUCAACCACAAACCGAUUUUCCACUUUCUGAUCUACAUCAUCAACAA
GGUCGAU
>EMBL_CDS:CAI14157 CAI14157.1 Homo sapiens (human) NHS-like 1
CCUAGGUCGAAGAAGUAUCACGGUGAAGGAAGUCAUCUUCAUGAUCUUCUUUCUCUUCACCUGCU
ACUCCUUUACCUAUGA
>EMBL_CDS:CAQ10280 CAQ10280.1 Homo sapiens (human) retinoblastoma binding protein 8
ACGGUCACCGAAGACCGACCGUCCCAGAAGAUACAAUCCGAAUCACAGUAAUCUCCUUGAAAAGC
GUUUUCUCCAGAGACCGU
>EMBL_CDS:CAH70298 CAH70298.2 Homo sapiens (human) RAB GUPase activating protein 1
AACGAUAAAGUCACAAACAGACUCCUAGAUUCUCAGAGUGUUAAGGAAGUCUGUGUAAGCCGUGA
CUUUCCCAUU
>EMBL_CDS:CAB99097 CAB99097.2 Homo sapiens (human) hypothetical protein
CGACCCCCGGAACCUCGACCGUCGACAACCCCCUCUCGAGUCCCCGACUCAGAGUGAGCGUCGGA
GGACUCGGAGGGGUAGGGAAGAGCCGAGUCCGGAGUCA
>EMBL_CDS:CAQ07669 CAQ07669.1 Homo sapiens (human) peroxisomal

biogenesis factor 5
CCCUCCCGGGAGAGGACUUCAGCGACGUCGGGAAGAAACUUUCCGACUCCCACUAGUGCGUCCCC
CAAGAGGA
>EMBL_CDS:CAH72120 CAH72120.1 Homo sapiens (human) H2.0-like homeobox
CGCCUCCGACAACGACGACGCCGACAACGACGACGACAACAACAACGCCCACUACCACCACCAUC
CGCCGACUCUCGCCUCUGUCGGCGACG
>EMBL_CDS:CAH74130 CAH74130.1 Homo sapiens (human) collagen type IV alpha 1
CCCGGAGGUCCUUUCGGACGAACAGGAAACAGUGGUUACCCUGGUCGUCCUGGUAUAGGACCUCC
GGG
>EMBL_CDS:CAH72225 CAH72225.1 Homo sapiens (human) leucine rich repeat containing 8
CGUCUACAGGGUUUCCUUUACAGCAAACAACAGGCGGGUAGAACCAGAACCAACCACCGACGAAG
GUAGAAACCCUACAACCACCACUGGAGCCGUAAAGGACCACCACAUACACG
>EMBL_CDS:CAI16937 CAI16937.1 Homo sapiens (human) component of oligomeric golgi complex
ACGUACUUCUGAGUUCGAUAACUUCUUAUAAAAAAACGAAUCGUUUACGUAAAGAAGUUACCAUC
AGUUAUUACGU
>EMBL_CDS:CAI14459 CAI14459.2 Homo sapiens (human) zinc finger protein 318
GACAACAGAAACCCUUAGGGUCCUUCCCCUGGGGUAACCAUCUCCAACGGGACUCCAAGGAAGUU
CCGUCAGAGAUGGUAUCAGGGGAACAAAAGUUAGAGACUCCGUUUUC
>EMBL_CDS:CAC21660 CAC21660.1 Homo sapiens (human) hypothetical protein
CAGGAAGGCACGACAUCAGGCCCCGUGCCGUUCCACACCCAGGAGACCACGGGAUCCAGCCCCCG
CCGCUCAGUCUCCAAGAGGGCACGGAACCAGAUCCCGCACCUUCCUG
>EMBL_CDS:CAI13266 CAI13266.1 Homo sapiens (human) zinc finger and BUB domain
UGUGCCCUAGAGGUCGUCCGCCCGUCGGACCUCGUGUCGACCGUACAACCGCGACGACCACCAGU
CACA
>EMBL_CDS:CAI14933 CAI14933.1 Homo sapiens (human) tudor domain containing 7
CGGAAACGUGACCUUACAGAAACUUACGAAGUAAAGGUUCUCAACACACAUCGUAUAUUCCUCUG
UAAAGUCGUAGUCCG
>EMBL_CDS:CAI41701 CAI41701.1 Homo sapiens (human) RNA binding motif protein 10
CGGAGAGGGGUAGGGUCAUGUCCAUGACGACCGAGACUCGUAACAUCAUUAUGACCCUCAACCCC
AGUAUCAUCUCCG
>EMBL_CDS:CAQ08432 CAQ08432.1 Homo sapiens (human) zinc finger protein pseudogene
ACACUCAUGAGACUACUUGUUCCUCCAAACCUAACAGUCUUUUCCGAGAAUCAACAAGUAAUAUA
AGUGU
>EMBL_CDS:CAH70855 CAH70855.2 Homo sapiens (human) ribosomal protein L12 (RPL12)

GGACUCCCGAAACUACUAGUCCCGUCUCCGUCUUCCGUGGUGGAGUUAGACCCGGACAGACAAGA
CUUACCAGUCAAAGUGACAUUAGGAGUCC
>EMBL_CDS:CAH71826 CAH71826.1 Homo sapiens (human) FRAS1 related extracellular matrix 1
CGGACCUCUUAUGUCAAUGUUCCAACGGGUCCAUAACAAGGGACGUUCGAACCCGUCGGCGUAAG
GUCCA
>EMBL_CDS:CAH74069 CAH74069.1 Homo sapiens (human) pseudogene similar to part of NADH
GUCUUUUUAACUCGAACAAUACUAAUAAGGAGUAUCCCUAUCAUACUCCCUCCUUAUUCGAUAUU
UGAGAC
>EMBL_CDS:CAI14932 CAI14932.1 Homo sapiens (human) amylase, alpha 1C (salivary)
CCAACCAUAGAAAGGGUGGUUCCAGACUUUCCCAACACUUACCGUUGUAAAAGUAAACCACCUCU
CUGGACUUGG
>EMBL_CDS:CAI13272 CAI13272.1 Homo sapiens (human) DC-SUAMP domain containing 1
UCGUGGUGGUCGUCGUCGUCUUACCCGUCACAGAGGUCCUCGAGGGAGUGGUGUAACGAGUAAAG
ACUCCGGACCUACCACCCGA
>EMBL_CDS:CAH71185 CAH71185.1 Homo sapiens (human) CDC42 binding protein kinase alpha
UCAUGCAGACGGAAACGAUCGACCUCAUGUCCUACCUCUCGCAAGUUUAACUAGGUCUCGUAGCC
AUCCGCAUAA
>EMBL_CDS:CAI14348 CAI14348.1 Homo sapiens (human) chromosome 9 open reading frame 126
AAGUCGUUCCGCUAAGCUACACUAUUGUUACCAUAAUAGUAGUAAAUUAUGGUAAUGUCCUAGGC
GGAGUUACUU
>EMBL_CDS:CAI14438 CAI14438.1 Homo sapiens (human) surfactant, pulmonary-associated
ACCUGAAGGGACCCCUGGGUACCGAGGUCGUCUGGGACGACGGGGACACAAAGGUCCCUGAGGU
>EMBL_CDS:CAC21674 CAC21674.1 Homo sapiens (human) hypothetical protein
GACGGACCAGGUCUCCAAGACGCCAGGUGUCGACCUUUUCCCGGAGGUGCCCGGCCCAGUC
>EMBL_CDS:CAI14538 CAI14538.1 Homo sapiens (human) human immunodeficiency virus type I
UACCCUUUGCAACCCCGAGACGAGACUCCGACUGGAGGCGUAGAGAGCCCGUACACCCACCACCG
ACCCCUGUCAGUUGUACACCCACGGGUCCCCAAACCCCGCGUACAGUCUACUCCCUGACCCCCGG
AGCCGAAAGGGUA
>EMBL_CDS:CAI14682 CAI14682.1 Homo sapiens (human) serine/arginine repetitive matrix 1
UCUUCCUCUGGCAGCGGAAGACCACCCACCACCCCUACCUGAAGAGGAGGCAGAUGGUGCUCCCC
UUCAGAGAAAAA
>EMBL_CDS:CAH70858 CAH70858.1 Homo sapiens (human) spen homolog, transcriptional regulator
CCCCAGUCGUUCGAAGAACAAACACCACUGUUACGAACCCCACUCCUGGUCGACCAACACUUGGU

CGUGUCCCCCACACAACUAGGG
>EMBL_CDS:CAI15846 CAI15846.1 Homo sapiens (human) CD52 molecule
UCGUCUUCUCCACCUAAUACCGUAACCGGUGCUUCUUUUCCUUUUACGGAGGCGAAUACAACGA
>EMBL_CDS:CAH74000 CAH74000.1 Homo sapiens (human) tudor domain containing 3
CUACUAAGAUCAUAAAAAAGACCGAAUUAACCGGGUCUAGAAGCAAACCCGGGUGAUAACAUUUU AUAAAAUAGUAG
>EMBL_CDS:CAD13322 CAD13322.2 Homo sapiens (human) gamma-aminobutyric acid (GABA) B
CCAGCUCCUUCAUCCCCGCGUCCUCACUGAGCAACGCCUAGACGAGCUACCGGUCAAGGUGCCGC CCCUCGUGUGGCGCGGGCUACGACGGGAACCGG
>EMBL_CDS:CAQ07191 CAQ07191.1 Homo sapiens (human) novel pseudogene
GUAAAGAACUAGGAACAUCUCCCCCUAGAAAUAACUCCCCUCUUUAUGGAAACCAUAAUCUAUGU UGUUCCUAGACUUAUAC
>EMBL_CDS:CAQ09904 CAQ09904.1 Homo sapiens (human) diacylglycerol kinase pseudogene
CCCAGGUCCUAUAUUUAGAGUAGGACACGCUAGAGUCAGUGUAUCAACUCCACGAGGACCCGAG
>EMBL_CDS:CAI10965 CAI10965.1 Homo sapiens (human) solute carrier family 31 (copper
UGGUGGACCUACUACACGUCGUGACAAACGUCCUCCACUCCUUUCGAGUCGUAGACAACGGGUUG UCAAAACACACAGAGGUAUUCCUACCA
>EMBL_CDS:CAI14296 CAI14296.1 Homo sapiens (human) eyes absent homolog 3 (Drosophila)
UAGUAAAGAAGGUAACAAUUUGGACUCGGUUAGUGAUGACAACCCAGGAAAGGUAUAAAGACCCG UAUCCUAGGUCAUUCUUCACUCACCUUCUACUACUA
>EMBL_CDS:CAI22608 CAI22608.1 Homo sapiens (human) MORN repeat containing 1
GACACGGCACCCACCACAGGGGGACCCCACGUCCGGCGGAGACCUAGACGGCGGACGGAAGAACA CCUCCGACAGGUCCUUGUCCUUCCCCACCAUGGGUCCGCCCCCGCCGGGGUCCUCCUGUAGACAC UUGAGCCGUGUC
>EMBL_CDS:CAH73652 CAH73652.1 Homo sapiens (human) olfactory receptor, family 10,
CUCUUUUUGGUGUUUCUCUCACUACACUACAAACACCUUAGACCUCUUAGGGUCCUUUUGUUUGA GACAAUGGAGACACUCCAAGAAAGAA
>EMBL_CDS:CAO72141 CAO72141.1 Homo sapiens (human) chromosome 1 open reading frame
CAGGCCGCCUUUCCCACAGGUCCUUUUCCAGAACCCAGGCUGUCACUUCUGAAGUCACCCGAGGC CCGGCGUGGACCCGAGGGCGGGCGGCCCG
>EMBL_CDS:CAQ08914 CAQ08914.1 Homo sapiens (human) actin related protein 2/3 complex,
AUAUAGUUCCAGGACAGUCGAAGUAAGAAUUAAAGUAUCAAAAACUUCUUCUGUAACCGGAACUU CAU
>EMBL_CDS:CAI13832 CAI13832.1 Homo sapiens (human) CDKN1A interacting zinc finger

CCCCGCCCCCUGUCCACAGUCCCCAUGUCACGAUUUCCGGGCCGACAGUGACGAGACGUCUUACC
ACUGGAAGUCCCUCGGGGGCCGCGGGGUAUGGGGACCGUAGAGAGAGCGAGA
>EMBL_CDS:CAI41249 CAI41249.1 Homo sapiens (human) immunoglobulin superfamily, member
UAUCAUUUUUGUCUCCAUUACAGGUCAUAGAACCUAGAAGUUUCUGACCUUCUUCUUUAAACGGA
GUAAAAAAUAACA
>EMBL_CDS:CAI12482 CAI12482.1 Homo sapiens (human) v-ski sarcoma viral oncogene homolog
GCGUCGAAGAGGAAGAACCGGUGCGCAUCCUUGAGGUCGAGGACCACCUCCGACGCGAACCGGAC
GUCCCGACGC
>EMBL_CDS:CAI12125 CAI12125.1 Homo sapiens (human) heparan sulfate proteoglycan 2
CUGGUCACCUACCACCCGCGCCCCGACGGACACCGACUCGUCGCGUUCCACCGGCACACAGGUCG
GAGGUGUCAG
>EMBL_CDS:CAI15178 CAI15178.2 Homo sapiens (human) ribosomal protein S24 (RPS24)
CACUUCCUGUAGAUGCUGACAAACACGAGGGACUCCAUCAACUAACCAGUACUUGAAGGAUCAUG
UUUAUCAAUAACACAGUAAGUA
>EMBL_CDS:CAI12649 CAI12649.1 Homo sapiens (human) myotubularin related protein 11
UGGGACCGGUCGGAACGAGUCCCAAUAGGGGCUUAUGACAACCCGAACUAACGAGACUCGAGACC
GAACCUGUUACCGGUACCA
>EMBL_CDS:CAI12547 CAI12547.1 Homo sapiens (human) aryl hydrocarbon receptor (AHR)
UACCUUAGGAAUAAAACUCGAUUAAACCAUCGUCUCAUAAAAGGUCAUCACGAUGAUAAUCAGGA
GUAAUCAUUCUCAAGGUA
>EMBL_CDS:CAI14505 CAI14505.1 Homo sapiens (human) chromosome 9 open reading frame 50
CCGGAAGAAGGUACUUGUCUUCCCGUCCGACGACAAGUACCUCCACAGGAGUCUUUCCAUCGGGA
CACAGAAGACCCACCUGUCCGG
>EMBL_CDS:CAI17060 CAI17060.2 Homo sapiens (human) pseudogene similar to part of NADH
UCCCAAAUUAUGACAAUUAUAUCGUACAUGUACAAGGAGCAACAUAACACUGUUCAUAUAUUUCC
CUUAUUUCCCGACACCAAUUAUAUAAUUCAGGA
>EMBL_CDS:CAH73128 CAH73128.2 Homo sapiens (human) protein tyrosine phosphatase,
UCGGUCUGCCUUUAUCAUAUUCGACAAACCAAAGUCAGGAACGUCGGAUAUACUACAGGGACCGA
>EMBL_CDS:CAQ10461 CAQ10461.1 Homo sapiens (human) NADH dehydrogenase 5 (MUND5)
AGGAUCAUAUAAUCUCUUCAACUUACAGACAUUACCCUUGGAAUCAAAUUCUAAUAAUCAAUACU
CUAAUUCGAGAUAACGAUCCU
>EMBL_CDS:CAI40039 CAI40039.1 Homo sapiens (human) chromosome X open reading frame 59
GUCAAAAGACGACAACGUCAUUGACCUUCACUUUUGUGGUCAAAAAAGACAGUAGUGUCUUUUCU

UCUAAC
>EMBL_CDS:CAQ11044 CAQ11044.1 Homo sapiens (human) mitochondrial AUP synthase 6
CGGUUACUGAGAAAACAAAACACCACCUUCAGUCACUAUUGACGAGAACCAAUAUCACCCUAUAG
AUAUAAAUCUAAAUAACUAUUGACUCAAUAACCG
>EMBL_CDS:CAI41100 CAI41100.1 Homo sapiens (human) melanoma antigen family H, 1
AACGGGUACUACUUCUACUCGCGAUUAUAUCUGUAGUCCUCUGAAAAGAACCACGGGACUUUUAA
CGAGGCCCCGUGACUUCCGAAAACACGAACCCGUCACGAGCUCCGAAGAAGUCCCCACC
>EMBL_CDS:CAH74086 CAH74086.1 Homo sapiens (human) hematopoietically expressed
AACCUCUUAGAGUGGACCGGCGGAAAGGAAAAUACGUCUCCGGAGACGUUCUUCCCCGAGGUCUC
AUCUCCAAACGGGUC
>EMBL_CDS:CAH73166 CAH73166.1 Homo sapiens (human) peptidyl arginine deiminase, type
CCUCCUGUUCAAACACCAGUAGUUUCUCCCGACGUCCCGGGACGCAGGCGUCCUGGUACUGUCUG
UACAGAAGG
>EMBL_CDS:CAQ06495 CAQ06495.1 Homo sapiens (human) UAF1 RNA polymerase II, UAUA box
GACCCACUACUUUACCGGUUCUUAGACCUUGACGGUAGUAAAAGACCCACUGUUACAACAGGUCU
UACUUUCUUUUACGGUGAACCAGUAGUAGUAGGUC
>EMBL_CDS:CAH73905 CAH73905.1 Homo sapiens (human) novel tudor domain containing protein
GGGACAAGAACUAAGCAGUUCUUAGAGUUAUUGUAUAACUACUAGGACAAAUAGUUCUUAUACC
>EMBL_CDS:CAI17217 CAI17217.1 Homo sapiens (human) tetratricopeptide repeat domain 13
UUGAAAGACGAAGUUUCGAAGAAACUUCCUAAGUUAUCGAAGGAAGUCUUCAGGAGACACUUUCU
UUCAA
>EMBL_CDS:CAI40666 CAI40666.1 Homo sapiens (human) novel protein
ACACCCAUGUCCGGCCCGACCACGGGAGGUAUGCCGCCGACAGGGCUCCCUCCCGGAACCGGACC
UAUGACGACGGGUAU
>EMBL_CDS:CAQ06806 CAQ06806.1 Homo sapiens (human) nicolin 1 (NICN1) pseudogene
ACCUCCUCACGUCCAACGAGUGUCCCGUGACCCACCCUCUCGGUGAACCCCUUCUAGUACCUCCC
CGAGAAACCAGGGACGACUAUCUAGACGUCGAGGAGGU
>EMBL_CDS:CAQ10659 CAQ10659.1 Homo sapiens (human) novel pseudogene
CAGUUCGGAAGGAAAUUUCGAUACUACAUCAACUAGAAGUCGUUUUUAGACCUUAACUUCUCCAA
ACUA
>EMBL_CDS:CAM13086 CAM13086.1 Homo sapiens (human) ADAMUS-like 1
CCGAAGUCCUCGCACAGGACCACGAGGUCCACCGACGCCUUCUAGAGGACCCGGUCGACCCGGUG
GUCCACGAACGACAUCUCCAG
>EMBL_CDS:CAI12265 CAI12265.1 Homo sapiens (human) adipose differentiation-related
AAGACGUCGACGGGGAACGAUCUUCACUCCUCCGACAGUCUGUGAAGAAAUUUCCUCCGUCGUAA

CGCCUU
>EMBL_CDS:CAH70693 CAH70693.1 Homo sapiens (human) stathmin-like 2 (SUMN2) pseudogene
GGGUGGAGCCGGAGGGUUUCACGACCCUAAUGUCCGCACUCGGUGACACGGGUCGGACUAAACGA
AGUGAAAGUAAAGUUAAUUCAUAUCUACAACUACAACGUUCUAAGGUCCAUUUUUGUUCUCGUCC
CGGUGUGGACCACCAAGUGUGGAUAUUAAGAUCGCGUAACUCUCCGACUCCACCC
>EMBL_CDS:CAI15376 CAI15376.1 Homo sapiens (human) poliovirus receptor-related 4
CGACACGAGUCACCAGUCACCCCCUUUCGGGUUUCACAGGGGUAGGUGAGCAUGGGGUGACCCGU
CUCCGGGUAGGUCGGCACAGGUCAACAUACUCCCUCCCCCGACGGGAAGUGAGUCCGUGAACUCG
UAUCG
>EMBL_CDS:CAI13541 CAI13541.1 Homo sapiens (human) dual-specificity
GACAAGAUGGAAGUAGAGUGGAGGUAGGUUUUUCACGACUCAUACUAGAGGACAUUUGACGAGUC
ACCAGUAUAAAUCAGAAGACCCACCUCCACCAAGUCUUCCUAACGUC
>EMBL_CDS:CAH70273 CAH70273.1 Homo sapiens (human) pseudogene similar to part of
AACUGUGUUCCACAAAGUUACCACAACUUCUCUUUAAGGGAAAGAAACCGUAGAAAAUACAUUCA
CAGUU
>EMBL_CDS:CAI40469 CAI40469.1 Homo sapiens (human) melanoma inhibitory activity family,
UCGAAACCGUCGAACAUCACUCAACGCCAGUAGAAGGUUAAAGAAAUGGACCAGAAGGUCCAAUG
UAAAUCACCUGUGCCUCCGAGAAUCGAAUUCGACAUUUUCGA
>EMBL_CDS:CAI16384 CAI16384.1 Homo sapiens (human) tropomodulin 4 (muscle)
GUCACACAUGUACAGGUCUUAACGACGUUACAGUGUGUAAAGUCGUAGACACCGUACACGGUCAC
>EMBL_CDS:CAI16380 CAI16380.1 Homo sapiens (human) LysM, putative
AGGAGAGGUAGAAAGAGAAGGAGUCUCAGGUUUGGUAACUUGUCCAGAGACCCGAGACAGUCCUA
CCCCUACAUCUCCC
>EMBL_CDS:CAI13935 CAI13935.1 Homo sapiens (human) small nuclear RNA activating
CCCCUAGCCGUCCUAGGUUCAGGAGUCUUCCGUCACUUAGACGAAGUCUUAGACUGAGCUCUGAA
CUAAGACUCUAGAGG
>EMBL_CDS:CAI39693 CAI39693.1 Homo sapiens (human) novel (FLJ20483) pseudogene
ACGGUAUAAAAAAUUUCACAACAGACACAAAAGAAUCUCCGUAAGGACGAGCUUUUCUACACCAU
>EMBL_CDS:CAI39581 CAI39581.1 Homo sapiens (human) HECU, UBA and WWE domain containing 1
CCGAGAAGAUCCUAAGGAGCCCUUAGACGAGGUCUUUACCGGUGCCGUCUUAGAGACCACCUUCU
CGA
>EMBL_CDS:CAI16184 CAI16184.1 Homo sapiens (human) cholinergic receptor, nicotinic,
GGUGUAGACCCUCGGUCUACACAAACCUUCCCUCGGCUUGAAAGAAGUACAACAGUUUGAGAAGU
CCGAAGGUCCACUCC
>EMBL_CDS:CAI13006 CAI13006.1 Homo sapiens (human) polo-like kinase

3 (Drosophila)
CCACUCGACAGAAGUCCACGACAGAGAUGGUCCGACUGUCCCCGACCCGGUCUUCGACGACCUCG
GAGACCGGACCGUAGGACUACCGGUUGCCUACACGCGUACUCCGGCGAGUGG
>EMBL_CDS:CAH69928 CAH69928.1 Homo sapiens (human) inositol polyphosphate-5-phosphatase,
GUGACUCGACUGUCCGAACACCAGUGACCAGAAGUCCCGGUACACCGAGACCAUUGAGUCGACUC
AC
>EMBL_CDS:CAI40037 CAI40037.1 Homo sapiens (human) cylicin, basic protein of sperm
AAGUCACAAACAGAAGAACCUUAAGAAACUUAGAAAGAAUAGAAAAACACUUGUUAAAAAACUCU
GACAGAAUCUUAAAACCCUAAGACCUAAUCAAAGACUU
>EMBL_CDS:CAI18907 CAI18907.1 Homo sapiens (human) carnitine palmitoyltransferase II
AGUACCACCGUAGUUUGAAAAGGAAUCGUCGACACUACGGUCAGAAAUUCCGUAGUCAGUCGAGC
UUCAACUCAAAGACGUGGCACU
>EMBL_CDS:CAQ09180 CAQ09180.1 Homo sapiens (human) mitochondrially encoded cytochrome
UGUUAUCCUUAAAGAUAACCCUCUUAAUGAGCUAACAGUUGAAACUCCUCAGCAUCAAGAGGACC
AAAAUUCAGACAACA
>EMBL_CDS:CAI18063 CAI18063.2 Homo sapiens (human) collagen type XI alpha 2
AGGACCCCCUGGAACACCCGGUAGUCGCGGUAACCCAGGUCGACCUGGAGCUCCAGGACCCCCCG
GUCCACCAGGGUAUCCU
>EMBL_CDS:CAI16169 CAI16169.1 Homo sapiens (human) potassium large conductance
AUCAAGUCACUACUACCCCUACAACUGGGGUCAACACUACCUACCAACUGCAUUGUAGGGCACGU
GACCCGAC
>EMBL_CDS:CAI17779 CAI17779.1 Homo sapiens (human) HLA complex group 4 pseudogene 5
CUUCCGCCACAUACCUAACCCCUCAGGGUCGGAACCCCUAAGGGGUUGAGGCGUCAAAGAAA
>EMBL_CDS:CAI17611 CAI17611.2 Homo sapiens (human) collagen, type XI, alpha 2
UCCUGGGAAGAGAGGUACUCCCGGCUACCGUGGGACCCCGGGCCCUUCUGGGACCCAGGGUCCCC
ACGGGACAACAGGUCCUCCAGGA
>EMBL_CDS:CAI18234 CAI18234.1 Homo sapiens (human) poly(A) binding protein interacting
UACGAAGAACCAUUAGGCCUAGUCGACGUCACUUUCCUUGUGAUACUUUGUGUUAUUGAUACACU
UACUGGUGAUAUUUUACAACCAAGUAAGUGUAUCAUUAACCCUAGAAAAAACCGACAACAUAAAG
ACAUACUACUUCAACGUA
>EMBL_CDS:CAI10948 CAI10948.1 Homo sapiens (human) jumonji domain containing 1C
CGGUUUCCAUGAACUCAAAAGGACUCGGUGGAAAAGAUUUAAUCACAGUAAACAUUAUAGUUAUU
GUGUAAAACCACACCCACCAGACCUAUGUUUGAAGAGGAAACCA
>EMBL_CDS:CAI95083 CAI95083.1 Homo sapiens (human) tripartite

motif-containing 46
GGAGCGAAGGAGGGUCCCAUACAGGGUUUCGGACUGAAUUCGUCGGCCAGGUCCGCCAGACCGAA
CCCCUCUCAAGACGCCCUCUCCGCCCC
>EMBL_CDS:CAI40878 CAI40878.1 Homo sapiens (human) potassium large conductance
AAAGACGGUACACAGAAGGAGUUAGAAAUACUUACUGGAGUACCUCUUCUCCAACCGUUCGUACC
ACCUCU
>EMBL_CDS:CAD38974 CAD38974.2 Homo sapiens (human) hypothetical protein
AGCUCUAGAUCUUGGUCUAGAUCUAGCGGAGGAAAAACCACUGGACCUACGACUGGAUCUAGAAC
U
>EMBL_CDS:CAI18078 CAI18078.1 Homo sapiens (human) tenascin XB
GUCGGUGUCGGUCACCUUCCCCCGUCCAAUUACGCCACCCACUUAUGGUGGACGAAGAGGGGAGG
AAGGUGACACACGAGCAUUUCGAC
>EMBL_CDS:CAD38738 CAD38738.1 Homo sapiens (human) hypothetical protein
CACCGACCCCCGUCCCGGUCCUGUGGAGGAGGAAACCCCAGACGUCAAAGGUCGACCGUCCGGCC
GACUGUCUGACGACCAAGAACUACCCUCCGUCACAGAACAUGAAGGAGCGUCCAAGUG
>EMBL_CDS:CAD39100 CAD39100.1 Homo sapiens (human) hypothetical protein
UCCCGUCACCCCCAUUGAGACGUAAACCCGGUACCCCCGGGUUCUCGUGAGACAGGAGGUGCCGA
CGAGA
>EMBL_CDS:CAD38978 CAD38978.2 Homo sapiens (human) hypothetical protein
CCACGUCGAACUACACCGUAGACCCGGGUCACGGGAGUUCACGGUGGUAGAGUCCGACGCGA
>EMBL_CDS:CAD38583 CAD38583.1 Homo sapiens (human) hypothetical protein
CUCAGUCUAAGACGUAGUCUAAGACUCAGUCCAAGACUAACCGUAAGCCUAAACCCAAGACUGAG
ACUAAGACUAAGCCUAAGACCGAG
>EMBL_CDS:CAD89960 CAD89960.1 Homo sapiens (human) hypothetical protein
CGAACAUCCCGUUUCUCUCUCCGACGUAGACACCGACAGAGUCUACCCCGAACACAAUAACCGGA
AUUCA
>EMBL_CDS:CAD38951 CAD38951.1 Homo sapiens (human) hypothetical protein
CCGCCCCUAGACGAGUAUCUGGUCGAGUUUCCGGCAGUCGGCCCGAGGGACCAACAUCCCGGCCC
AGAGCUCGUGUCAGGGCGG
>EMBL_CDS:CAD39091 CAD39091.1 Homo sapiens (human) hypothetical protein
CCAUGAGAUCAAUCUUUACCCGACCUAAAAACUCCGUGUCUUAAGUCGAGACAAGGUUGUAAUCU
CCCGG
>EMBL_CDS:CAD89901 CAD89901.1 Homo sapiens (human) hypothetical protein
CGAGUUAGAAGAGGUCUUUCGUUGGAGGGUACAUCCUCGUGACGACCCUCCCGUCAAACUUUGAC

CUUUACCCUAACUACG
>EMBL_CDS:CAD39157 CAD39157.1 Homo sapiens (human) hypothetical protein
ACCUCACGAACCAGGGUUCGUCGAAGAGAAUGUCCUCCACUCCCUCCGACACUCGGUAGAGGU
>EMBL_CDS:CAH74027 CAH74027.1 Homo sapiens (human) laminin receptor 1 (ribosomal
UCUCGUCCGACGACGAUUUUUUCGACUCCGAUUCCUCCUUAAAGUCCCACUUCCCUGACGGGACC
GAGGACUCACAUGACGAUGAGUCGGACUCCAACGUCGGACGAAA
>EMBL_CDS:CAH10602 CAH10602.1 Homo sapiens (human) hypothetical protein
CGGCCCCGACGUCGUCCACUCUUCACGUCCUCGACCCUCAACGACGGUGACGGUCGUCGUCG
UGGUCGUGGACGUCACCGUUACCGCCCCCGACACCG
>EMBL_CDS:CAD39047 CAD39047.1 Homo sapiens (human) hypothetical protein
UACCGAAAAGAGACUCCCUGACACUACAGAAGAGAGUCUCCCUGACGCUACAGAAGAGAGUCUCC
CUGACGUUACCGAAGAGAGUCUCCCUGACGUUACCGAAGAGAGUCUCCCUGACACUACAGAAGAG
AGUCUCCCUGACACUA
>EMBL_CDS:CAD91151 CAD91151.1 Homo sapiens (human) hypothetical protein
AGACGGUAGACCCGAAAGUACCGGAGGAACCGGAAGGAAUGUCGGUCGUUCGAGUGGAGGUAGAC
CAGUGGGUUCACGGCCUACGACCGGACGAGCCGGAGGUCCCUCCGGUUCCUCGGGACCUACCGAC
U
>EMBL_CDS:CAM25754 CAM25754.1 Homo sapiens (human) HLA complex group 22
AGUGACGACACUGACUUCAUCAAGUGUCUUCCACUCACAGUGGUACAAAUCACCAAAGUAGAAGA
CACCAGGGACCCAGACGUCACU
>EMBL_CDS:CAD38816 CAD38816.2 Homo sapiens (human) hypothetical protein
ACUUCACCCGCCGGCUCCCCGACAACACGGCCGAGUGGGACGUCCCGUCCCGUCGGUGCCACCGC
AUCAAGU
>EMBL_CDS:CAD38604 CAD38604.1 Homo sapiens (human) hypothetical protein
UCGUCGUAGAAAAAGAAGUCUGAACCCUCGACCACGGUGGUGUCGGGGCAGACUUCUUCCCACGA
GGA
>EMBL_CDS:CAD38784 CAD38784.1 Homo sapiens (human) hypothetical protein
CGAAACAGAAGGUCCUCGAUUUUUUCCAGAAAGACCAACUGAUUUUUAUAGAGGACCUCCUUUUC
CA
>EMBL_CDS:CAH10604 CAH10604.1 Homo sapiens (human) hypothetical protein
GACUUUUUAGGAGAAAACCCUCUACUGAAUCUAACACAUUCACCGUUUCUUUCAACAGCAACUAC
AAACUUCGCGUAAGGAAAGGCAACCGGAAACAGGUCAAAACCGAAAAGGUGUAUUUCUCCCAAAA
ACUC
>EMBL_CDS:CAD38994 CAD38994.1 Homo sapiens (human) hypothetical

protein
GGGACGACCAGACCUGUUACGGGUGUCACCGGUCAUCCGGCGUCGUACCAUAAGAUUCACCGACC
CUCCGGUCCUCCACCCCGAAUGCGACACACGUCUUCUCAGGUCAGGUCGGUCCC
>EMBL_CDS:CAI18564 CAI18564.1 Homo sapiens (human) Notch homolog 4 (Drosophila)
GAAGACGGGAGUGUGGGGAACCGAGGCCCAAAGACUGUGGCCCCGGGACCUCUCGACACUCCCGU
CCUC
>EMBL_CDS:CAM25804 CAM25804.1 Homo sapiens (human) collagen, type XI, alpha 2
CAGGUCCUCCAGGACCGAGAGGGACCCCAGGGUUCGAGGGAAACCCCGGGACCCCUGGUAGGUAC
GGAGCCUG
>EMBL_CDS:CAQ10080 CAQ10080.1 Homo sapiens (human) zinc finger protein pseudogene
UAUUACUUAUUCUGAAUCGGAGAUUAACUUCCGAAUAAGUAAUGUCCAUGUCUCACAGACAGGUG
AUACUUAGACGUCUACUACUUGUUCCUAACUAAAGACAAACCUCCAAAAACAUAUAAGUAACA
>EMBL_CDS:CAQ09244 CAQ09244.1 Homo sapiens (human) mitochondrial ribosomal protein
AACUCUAACCACCUCACCUGGAAGAGUAAUGAGUAGUAGAUCUCCUUCCAGGAGUAGUAGAGAUG
AAGAGUU
>EMBL_CDS:CAH71081 CAH71081.1 Homo sapiens (human) rearranged L-myc fusion
ACGUAUCAAAGUCUCACUAGAUCACACAGUUAAUCGAGAAGUAAGUAACUUCGACUUCUCGAUAC
UGAAUUGACUAGUGAUCUUAAUGACAGU
>EMBL_CDS:CAJ20055 CAJ20055.1 Homo sapiens (human) Cox18Hs1 protein
AAGGUGGAACCGGUCCUACAUCACGACCAUCCGACGGUUUCCGUCGCACUGUCGUGGGGCAUUCC
GGUGCCACCUC
>EMBL_CDS:CAL06519 CAL06519.1 Homo sapiens (human) immunoglobulin heavy chain variable
AGUCCGACCAGUAAACGUUUAUGUCGCACCUGAACCUUAACAGAGACCUCUACCACUUGGCCGGG
AAGUGCCUCAGACGCAUCAUACACGAUACUGGUCAUACU
>EMBL_CDS:CAJ57189 CAJ57189.1 Homo sapiens (human) immunoglobulin variable kappa
AAUCCUCGAAUCCUCGAAAGGGACCAAAGACGACUAUGGUCCGAUUUCGUGAUGAUUACGGGACU
>EMBL_CDS:CAJ84703 CAJ84703.1 Homo sapiens (human) cytochrome P450 1A1
GCACGAAUAGUCCUGGAGUCGGAGGAACGAGUGUACGAGAAGGUCCAUCGUCCUCCAACUCCUCC
GACCCAGUCUCCGUUAC
>EMBL_CDS:CAJ75539 CAJ75539.1 Homo sapiens (human) immunoglobulin lambda light chain
CGAAGACGACCCUGGUCGAACCUAUCAGUAAUAACCACUGACGAGGUCACGACCUUCGUGUCCA
>EMBL_CDS:CAJ75485 CAJ75485.1 Homo sapiens (human) immunoglobulin heavy chain
CACGAAGUGCACCGUACAGUGCCUGAACGGCCGAUCCGUGACACACCGGCCGUCCCAGUCGACCG
ACGAGCACCACAUG

>EMBL_CDS:CAK50629 CAK50629.1 Homo sapiens (human) immunoglobulin A heavy chain
CCAGGUACAUCCGACACCAACGCCUACACAGGGACCAUUCCCACUGAGACGGGACCUUAAAGACA
CUUAUAAAACACAGUGGUGACGGUCGCACCUAG
>EMBL_CDS:CAK50713 CAK50713.1 Homo sapiens (human) immunoglobulin A heavy chain
GUCCAGUCACACUCCCAGACACACCCGAAGUCACGACACCCUGGUCUGGGAGCUCCAACUGGAC
>EMBL_CDS:CAM34545 CAM34545.1 Homo sapiens (human) autogenous vein graft remodeling
CGCGGGUGGUGGUGCGGGUCGAUUAAAACAAAACAUAAAAAACUCUACCCAAAGGACACAAUCGG
UCCUACCAGAGCUAGAGGACUGGAACACUGAUGUGCCACUUUGAGACGGAGAUGAUUUUUAUGUU
UUUUAAUCGGCCCGCACCACCACCCGCG
>EMBL_CDS:CAQ37746 CAQ37746.1 Homo sapiens (human) putative ankyrin repeat and sterile
AUCCCUCGGUGGUCACCACAUUCACGAAACCGAUUCACUACCUCUGUUGACUCCUCCUCACACUA
AUGGUGACCCAAGGGAC
>EMBL_CDS:BAA95561 BAA95561.1 Homo sapiens (human) liver-type 1-phosphofructokinase
GAGGAACCCCCCGAAGACCACCCGCUCCUCGAACAUUUACAAGGUCAACAAGAGCUUCGAGGGUG
GUGCCUC
